# Identification of a regulatory pathway inhibiting adipogenesis via RSPO2

**DOI:** 10.1038/s42255-021-00509-1

**Published:** 2022-01-13

**Authors:** Hua Dong, Wenfei Sun, Yang Shen, Miroslav Baláz, Lucia Balázová, Lianggong Ding, Mona Löffler, Bradford Hamilton, Nora Klöting, Matthias Blüher, Heike Neubauer, Holger Klein, Christian Wolfrum

**Affiliations:** 1 Institute of Food, Nutrition and Health, ETH Zurich, Schwerzenbach, Switzerland; 2 Global Computational Biology and Digital Sciences, Boehringer Ingelheim Pharma GmbH and Co. KG, Biberach/Riss, Biberach, Germany; 3 Institute of Experimental Endocrinology, Biomedical Research Center at the Slovak Academy of Sciences, Bratislava, Slovakia; 4 Department of Animal Physiology and Ethology, Faculty of Natural Sciences, Comenius University in Bratislava, Bratislava, Slovakia; 5 Helmholtz Institute for Metabolic, Obesity and Vascular Research (HI-MAG) of the Helmholtz Zentrum Munchen at the University of Leipzig and University Hospital Leipzig, Leipzig, Germany

**Keywords:** Metabolism, Biochemistry, Fat metabolism, Gene expression analysis

## Abstract

Healthy adipose tissue remodeling depends on the balance between de novo adipogenesis from adipogenic progenitor cells and the hypertrophy of adipocytes. De novo adipogenesis has been shown to promote healthy adipose tissue expansion, which confers protection from obesity-associated insulin resistance. Here, we define the role and trajectory of different adipogenic precursor subpopulations and further delineate the mechanism and cellular trajectory of adipogenesis, using single-cell RNA-sequencing datasets of murine adipogenic precursors. We identify Rspo2 as a functional regulator of adipogenesis, which is secreted by a subset of CD142^+^ cells to inhibit maturation of early progenitors through the receptor Lgr4. Increased circulating RSPO2 in mice leads to adipose tissue hypertrophy and insulin resistance and increased RSPO2 levels in male obese individuals correlate with impaired glucose homeostasis. Taken together, these findings identify a complex cellular crosstalk that inhibits adipogenesis and impairs adipose tissue homeostasis.

## Abstract (web summary)

Dong and Sun et al. show that adipogenesis regulatory cells in murine white adipose tissue inhibit adipogenesis through RSPO2, which regulates maturation of early progenitor cells via Lgr4.

## Main

Obesity is an increasingly prevalent condition that develops when energy intake exceeds expenditure by depositing the excess energy within adipose tissue in the form of triglycerides^1^. Even though adipose tissue is viewed mainly as a lipid storage organ, white adipose tissue (WAT) contains only 20–30% of mature lipid-filled adipocytes, whereas adipocyte progenitor cells (APCs)^2^ are contained in the stromal vascular fraction (SVF). One process that influences adipose tissue plasticity and function, tightly linked to metabolic health, is adipose tissue expansion, which occurs via two processes: the recruitment and differentiation of APCs by hyperplasia and the hypertrophy of mature adipocytes^3,4^. The latter has been linked to the development of metabolic disorders likely due to insulin resistance in the adipose tissue^5^, whereas hyperplasia has been shown to protect against obesity-associated comorbidities^6–8^. Taken together, these data demonstrate the importance of adaptive adipogenesis, which drives adipose tissue plasticity and might be an unexplored avenue to develop strategies to treat obesity-associated comorbidities.

Many studies in recent years have tried to delineate the adipogenic response to better understand the cellular and molecular events driving this process^4,9–11^. This is illustrated by the heterogeneity of APCs in different depots of the adipose tissue, which have been identified using single-cell RNA sequencing (scRNA-seq). A study from Merrick et. al.^4^ revealed the hierarchy of distinct populations of APCs in mouse and human adipose tissue by scRNA-seq. Similarly, previous work^9^ from our laboratory on adult inguinal WAT (ingWAT) demonstrated the presence of three clusters of APC populations. Two adipogenic populations were marked by Lin^−^Sca1^+^CD55^+^ (population 1, P1) and Lin^−^Sca1^+^VAP1^+^ (population 2, P2), while a third subpopulation, which is characterized by Lin^−^Sca1^+^CD142^+^ expression, was shown to suppress adipogenesis in vitro and in vivo. The aim of the present work was to delineate the cellular and molecular mechanisms that control adipogenesis to regulate adipose tissue homeostasis.

## Results

### Integration of APC scRNA-seq data reveals heterogeneity of adipocyte progenitor cells

**Fig. 1:**
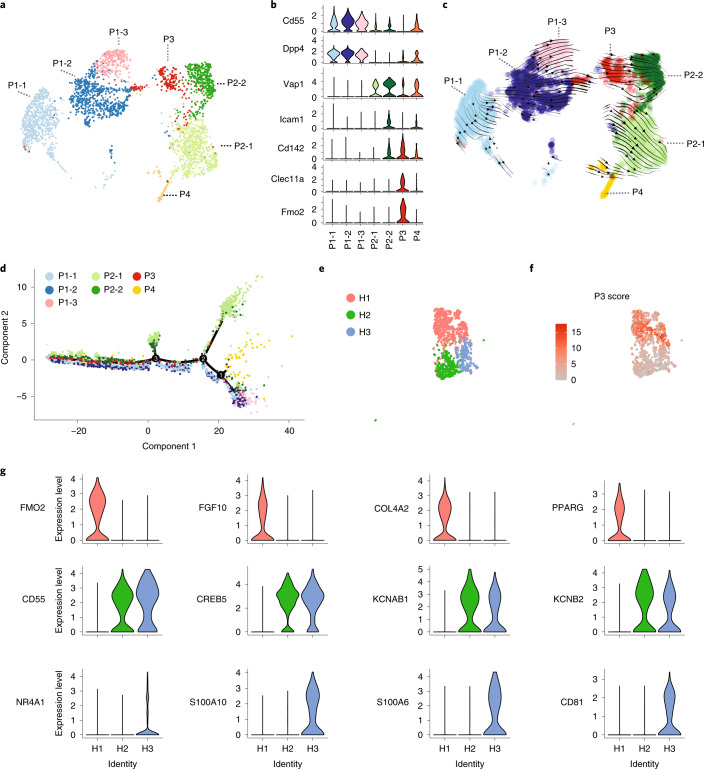
Integration of different scRNA-seq data further reveal the heterogeneity of adipocyte progenitors. **a**, Uniform Manifold Approximation and Projection (UMAP) two-dimensional map of cells derived from 10X dataset in our previous study^9^ shows several distinct clusters, including *Cd55*^+^ progenitor cells (P1-1, P1-2 and P1-3), two subpopulations of committed pre-adipocytes (P2-1 and P2-2), P3 cells and dividing cells expressing cell-cycle genes of S phase (P4). **b**, Violin plots showing the expression of marker genes. *Cd55* and *Dpp4* (marker of cluster P1-1–P1-3); *Vap1* and *Icam1* (marker of cluster P2-1–P2-2); and *Cd142*, *Clec11a* and *Fmo2* (marker of cluster P3). **c**,**d**, Cell trajectory analysis of Lin^−^ cells by Velocyto and scVELO (**c**) and Monocle 3 (**d**). **e**,**f**, Single-nucleus RNA-seq (snRNA-seq) of human deep neck adipose tissue. Unsupervised clustering of pre-adipocyte populations shown as UMAP plot (**e**). P3 score (**f**) calculated as the sum of *F3*, *CLEC11A*, *FMO2*, *GAS6*, *CYGB*, *PPL* and *STEAP4* for each cell. **g**, Feature plots of H1 (*FMO2*, *FGF10*, *COL4A2* and *PPARG*), H2 (*CD55*, *KCNAB1*, *KCNB2* and *CREB5*) and H3 (*NR4A1*, *S100A10*, *S100A6* and *CD81*) markers in preadipocyte nuclei of human deep neck adipose tissue.

**Extended Data Fig. 1:**
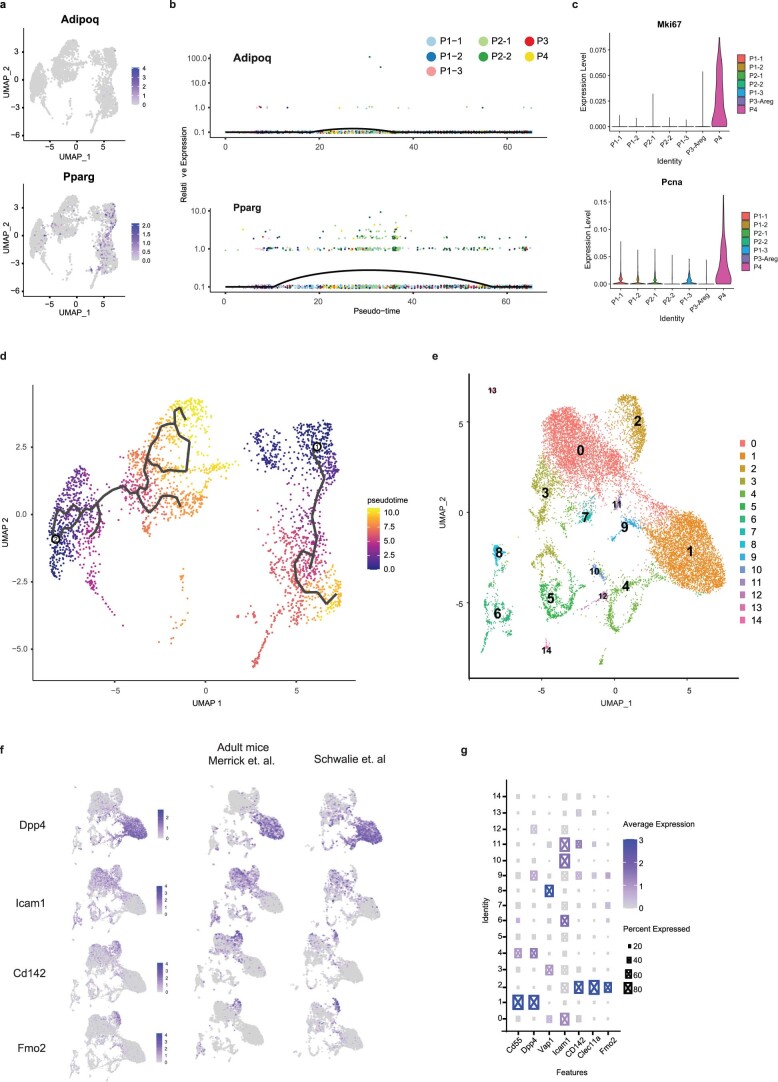

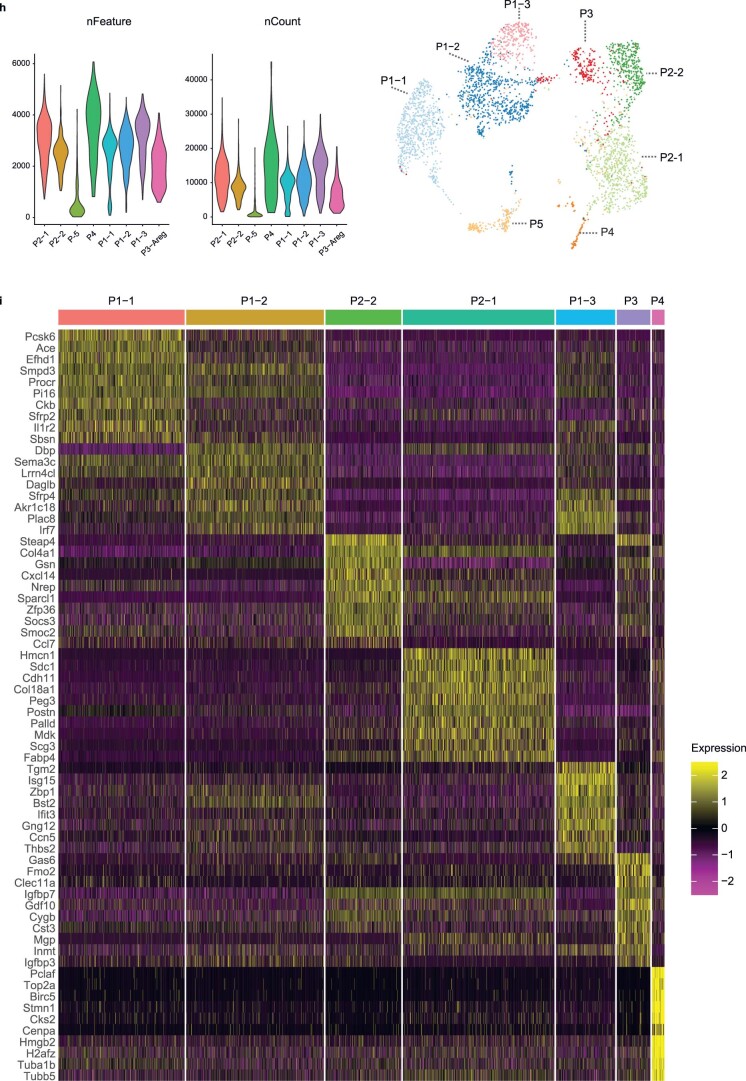

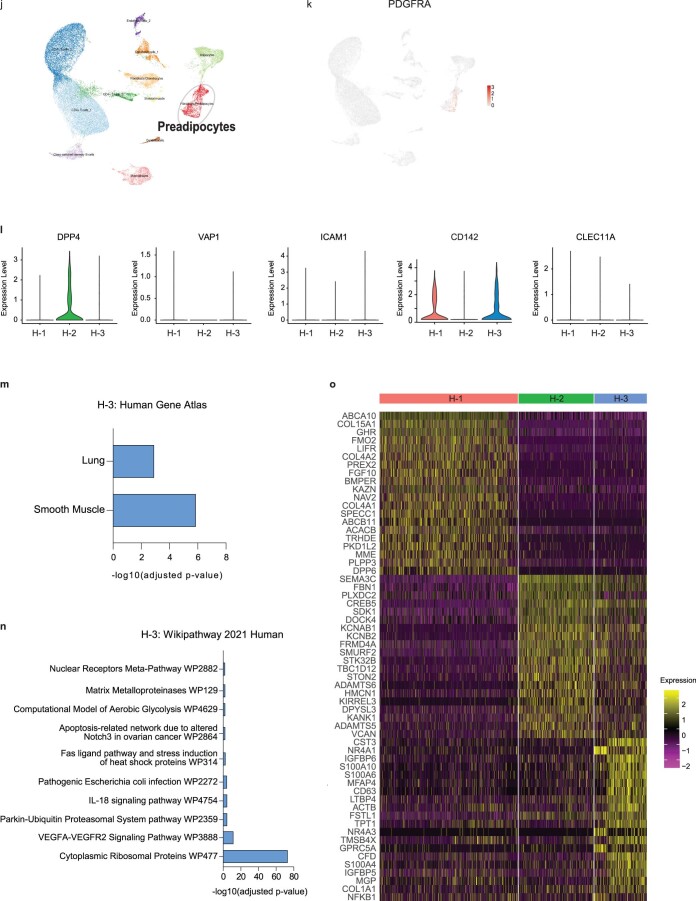
Integration of two scRNAseq of mouse Lin- cells from ingWAT and delineation of the heterogeneity of preadipocytes in human adipose tissue by single-nuclei sequencing. **a**) Feature plots of *Pparg* and *Adipoq* in mouse preadipocytes clusters. **b**) *Adipoq* and *Pparg* relative expression along the pseudo trajectory. **c**) Cell cycle related genes expression of mouse preadipocytes clusters. **d**) Cell trajectories reconstruction. **e**) UMAP of aligned cells derived from study Schwalie et. al^9^ and Merrick et. al^4^. Cluster 0: Cd142+ committed preadipocytes; Cluster 1: Cd55+ progenitors; Cluster 2: Aregs; Cluster 3: Vap1+ committed preadipocytes. **f**) Feature plots of gene expression (log2 CPM) of marker genes for progenitor cells: *Cd55, Dpp4*; for committed preadipocyte: *Vap1, Icam1*; and for Aregs: *Cd142, Clec11a* and *Fmo2*. **g**) Integrated dot plots of gene expression (log2 CPM) of marker genes for progenitor cells: *Cd55*, *Dpp4*; for committed preadipocyte: *Vap1*, *Icam1*; and for Aregs: *Cd142, Clec11a, and Fmo2*. **h**) Violin plots of number of genes and reads detected in the unsupervised clustering of mouse SVF cells^9^ (Methods). **i**) Heatmap of signature genes expression across mouse preadipocytes clusters. **j**) Unsupervised clusters of preadipocytes from human deep neck adipose tissue. **k**) Feature plots of *PDGFRA* in nuclei isolated from human deep neck adipose tissue. **l**) Violin plots showing the expression of *DPP4, VAP1, ICAM1, CD142, CLEC11A* in the clusters colored in Fig. 1a. **m**) Cell type analysis was performed by Enrichr on significant genes of H3 cluster. Bars were sorted by p-value ranking. P-value was computed using the Fisher exact test. **n**) WikiPathway (WikiPathway 2021 human database) enrichment analysis was performed by Enrichr on significant genes of H3 cluster. Bars were sorted by p-value ranking. P-value was computed using the Fisher exact test. **o**) Heatmap of signature genes expression across human preadipocytes clusters H1-H3. This figure is related to Fig. 1. Source data

In a previous study^9^, we defined Lin^−^Sca1^+^CD142^+^ APCs as adipogenesis regulatory (A_reg_) cells and demonstrated that these cells are both refractory toward adipogenesis and control adipocyte formation of APCs through paracrine signaling. In contrast, Merrick et. al.^4^ observed that Lin^−^CD142^+^ cells could differentiate into adipocytes. To study these seemingly inconsistent observations, we re-examined 10X scRNA-seq of Lin^−^ cells with the most recent computational algorithms^12–17^, which by unsupervised clustering of the transcriptomes revealed seven distinct subpopulations (Fig. 1a). The newly identified clusters P1-1, P1-2 and P1-3, which express *Cd55* and *Dpp4* (Fig. 1b) represent an early progenitor population and resemble the previously identified cluster G1/G4^9^/Group 1 (ref. ^4^). The previously identified G2^9^/Group 2 (ref. ^4^) cells, which represent committed APCs, could be divided into two *Pparg*-expressing subgroups (Extended Data Fig. 1a,b) P2-1 (*Cd142*^−^) and P2-2 (*Cd142*^+^) based on their *Cd142* expression (Fig. 1b). The newly defined cluster P3 represents a subset of the previously identified A_reg_^9^/Group 3 cluster^4^, with specific expression of *Cd142*, *Clec11a* and *Fmo2* (Fig. 1b) and which is separated from the P2-2 cluster. The proliferating P4 cluster expresses high levels of cell-cycle genes (Extended Data Fig. 1c).

To define the relationships between these cells, we next predicted the cellular trajectories of these cell clusters through dynamical modeling of RNA splicing events by Velocyto^12^ and scVELO^13^ (Fig. 1c). The models suggested that P1 cells can transit to P3 cells and further to either P2-1 or P2-2 cells. Another trajectory analysis using Monocle^16^ (Fig. 1d and Extended Data Fig. 1d) inferred that P1 cells can transition through the first branch point to become proliferating P4 cells or through the second branch point to become P2-1 cells. Some P1 cells continue transit through the third branch point to form the cluster of P2-1, P2-2 or P3 cells (Fig. 1d). These data are consistent with previous experimental findings^4,9,18^ that demonstrated that P1 cells define a group of early adipocyte progenitors, whereas *Pparg-*expressing P2 cells (Extended Data Fig. 1a,b) represent committed preadipocytes.

Given the newly identified separation of Cd142^+^ APCs into P3 and P2-2 cells, we hypothesized that the discrepancies reported by us and Merrick et. al. could be due to the fact that these two populations were analyzed as mixtures. To address this, we aligned data from the two mouse datasets from Merrick et. al. to our own data using canonical correlation analysis^15^ (Extended Data Fig. 1e) and performed clustering based on the aligned results. Indeed, we observed two distinct clusters of *Cd142*^+^ cells in the integrated analysis (cluster 0 and 2) (Extended Data Fig. 1f,g). Cluster 2, but not other *Cd142*^+^ cells, express *Cd142*, *Clec11a* and *Fmo2*, similar to the newly defined P3 cells. Cluster 0 was similar to P2-2, which expresses *Cd142*, *Icam1* and *Vap1* (Extended Data Fig. 1f,g).

We next wanted to extend our analyses to the human situation, as little is known about the presence of either early or late adipocyte progenitors. Therefore, we resolved the adipocyte heterogeneity in human deep neck subcutaneous adipose tissue (SAT), which allowed us to define 12 subpopulations^19^ (Extended Data Fig. 1j). The pre-adipocyte cluster featured by the pre-adipocyte marker *PDGFRA*^20^ (Extended Data Fig. 1k) could be further subdivided into three subsets, termed H1–H3 (Fig. 1e). We failed to correlate the mouse P3 cluster with either H1–H3 clusters, as the overlaps were not statistically significant (Extended Data Table 1). Alternatively, using the P3 score as a sum of mouse P3 signature genes (Fig. 1f) and *Cd142* expression (Extended Data Fig. 1l) indicated that mouse P3 cells were enriched in cluster H1 and H3. Based on these findings, it would be worthwhile to investigate, whether H1 or H3 cells are functionally similar to mouse P3 cells. The enrichment of *PPARG* in the H1 cluster, suggests that these cells might constitute the committed pre-adipocytes within human SAT (Fig. 1g). Enrichr analysis of the H3 signature denotes this cluster as a smooth-muscle-cell-like population (Extended Data Fig. 1m) with enriched pathways such as VEGFA–VEGFR2 signaling or the matrix metalloproteinase pathway, which might regulate adipose tissue microenvironment (Extended Data Fig. 1n) and the expression of known adipogenesis regulatory genes such as *NR4A1* (ref. ^21^) and *FSTL1* (ref. ^22^) (Extended Data Fig. 1o). Taken together, these data suggest that H3 might constitute a regulatory cell type within human SAT; however, more studies will be needed to delineate the function of H3 cells.

### In-depth functional analysis of the different cell populations within mouse adipose tissue

**Extended Data Fig. 2:**
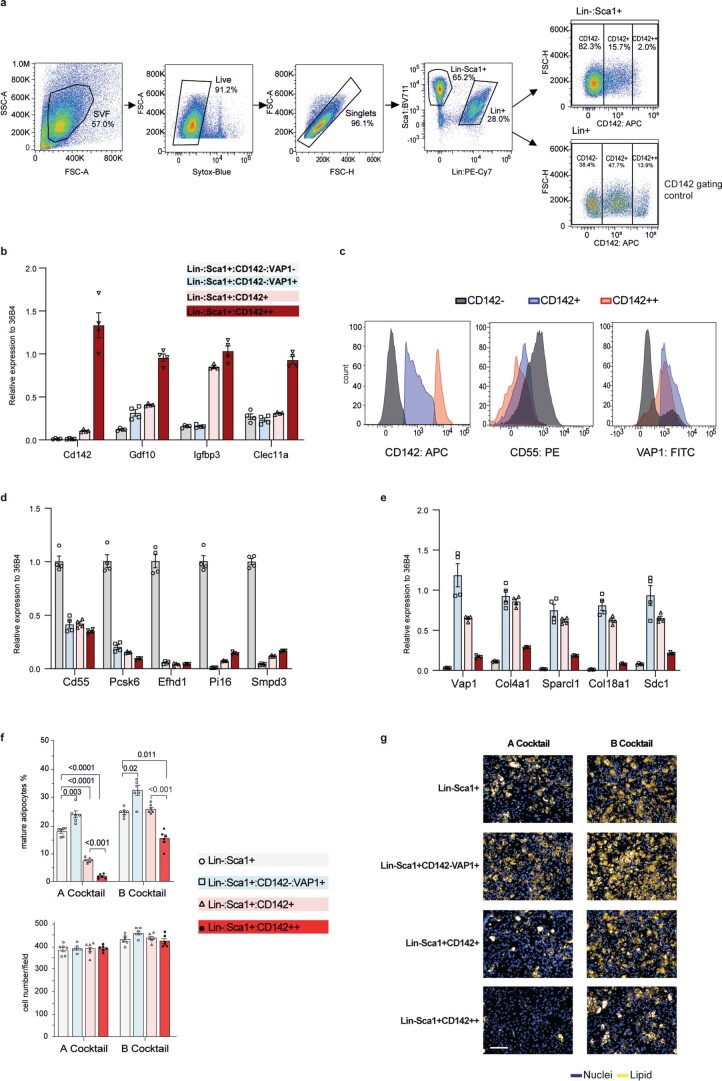
Discrepancies of the two studies^4,9^ regarding the sorting and culture of Aregs. **a**) Representative flow cytometry dots plots showing the gating strategy used to identify CD142-, CD142 + , and CD142 + + cells. **b**) Relative mRNA levels of P3 specific marker genes (*Cd142, Gdf10, Igfbp3, Clec11a*) in different cell populations. n = 4 biological replicates. Data show the mean ± SEM. **c**) Histogram plots shows expression level of CD142, CD55, and VAP1 in CD142- cells, CD142 + cells and CD142 + + cells. **d**) Relative mRNA levels of P1 specific marker genes (*Cd55, Pcsk6, Efhd1, Pi16, Smpd3*) in different cell populations. n = 4 biological replicates. Data shown as mean ± SEM. **e**) Relative mRNA levels of P2 specific marker genes (*Vap1, Col4a1, Sparcl1, Col18a1, Sdc1*) in different cell populations. n = 4 biological replicates. Data shown as mean ± SEM. **f**) Cells were sorted from ingWAT. Adipogenesis was induced by A Cocktail (1 μM dexamethasone, 0.5 mM isobutylmethylxanthine, 1 μM insulin) or B Cocktail (1 μM dexamethasone, 0.5 mM isobutylmethylxanthine, 125 nM Indomethacin, 1 nM T3, and 20 nM insulin). Quantification of adipogenesis (top) and cell numbers (below) in culture well on differentiation day 6. Data shown as mean ± SEM. *F*(3,30)= 14.78, *P* < 0.0001 by two-way ANOVA. For Cocktail difference, *F*(1,10)=290.5, *P* < 0.0001. For cell type difference, *F*(1.929,19.29)=148.8, *P* < 0.0001. **g**) Microscopy images of different cell populations shown on differentiation day 6 in f. In all panels, nuclei were stained with Hoechst 33342 (blue) and lipids were stained with LD540 (yellow). Scale bars, 100μm. This figure is related to Fig. 2. Source data

Caution needs to be used when employing CD142 as a marker to isolate P3 cells, as adipogenic CD142-expressing P2-2 cells will also be collected. This fact might explain the divergent findings regarding the adipogenic potential of P3 cells^4,9,23^. In our previous study^9^, we observed a continuum of CD142-expressing cells within the Lin^−^Sca1^+^ (enriched pool of APCs) cell fraction (Extended Data Fig. 2a). This is supported by the finding that Lin^−^Sca1^+^CD142^++^ cells are more similar to P3 cells compared to Lin^−^Sca1^+^CD142^+^ cells based on P3 signature gene expression (Extended Data Fig. 2b). Furthermore, abundant P1 and P2 cells are admixed to the Lin^−^Sca1^+^CD142^+^ fraction, while fewer are observed in the Lin^−^Sca1^+^CD142^++^ population (Extended Data Fig. 2c), which was confirmed by the analysis of P1 and P2 marker gene expression (Extended Data Fig. 2d,e). Thus, for gating of P3-like Lin^−^Sca1^+^CD142^++^ cells, CD142 staining within the Lin^+^ population could be used as a reference control (Extended Data Fig. 2a). Based on this strategy we next examined the adipogenic capacity of the following cell populations with different cocktails (Extended Data Table 2): Lin^−^Sca1^+^ cells, Lin^−^Sca1^+^CD142^−^VAP1^+^ cells (P2-1), Lin^−^Sca1^+^CD142^+^ (P2-2) and Lin^−^Sca1^+^CD142^++^ cells (P3). We observed Lin^−^Sca1^+^CD142^++^ cells were refractory toward adipogenesis similar to the previously described A_reg_ population^9^ upon adipogenic cocktail induction, whereas Lin^−^Sca1^+^CD142^+^ cells could form adipocytes with previously used induction strategies^4^ (Extended Data Fig. 2f,g).

**Fig. 2:**
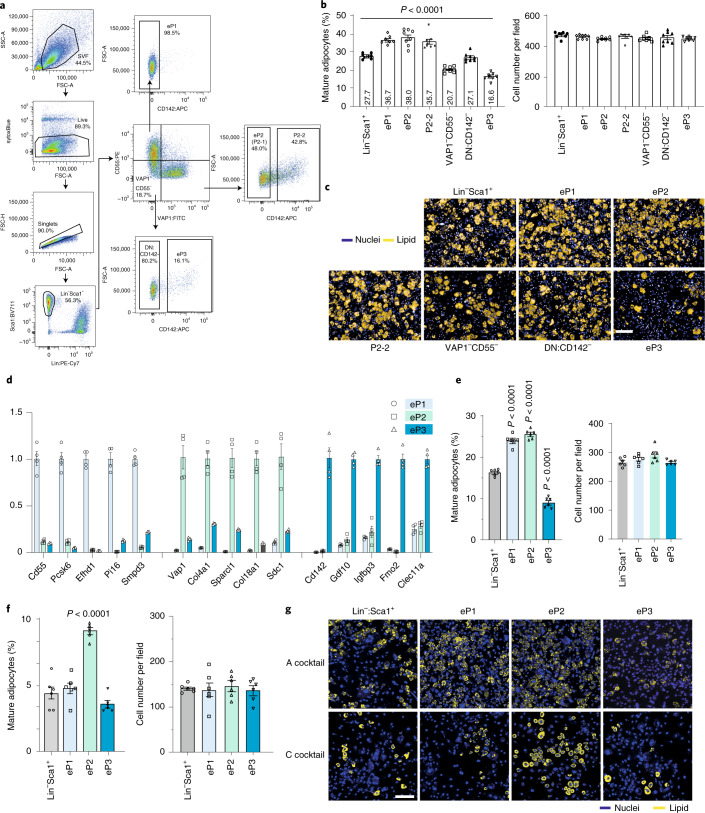
Classification of different cell populations within the adipose tissue. **a**, Flow cytometry dot plots show the new gating strategy used to sort eP1, eP2 and eP3. **b**, Quantification of adipogenesis (left) and cell number (right) of Lin^−^Sca1^+^ cells, eP1 cells, eP2 cells, VAP1^+^CD142^+^ APCs, VAP1^−^CD55^−^ cells, DN:CD142^−^ cells and eP3 induced by A Cocktail (1 μM dexamethasone, 0.5 mM isobutylmethylxanthine and 1 μM insulin). Data are shown as mean ± s.e.m., *n* = 8 independent wells. Data were analyzed with one-way analysis of variance (ANOVA); *F*(6,49) = 69.7002, *P* < 0.0001. **c**, Microscopy images of different cell populations shown in **b** on differentiation day 6. Experiment was repeated twice. **d**, Relative mRNA levels of P1 marker (*Cd55*, *Pcsk6*, *Efhd1*, *Pi16* and *Smpd3*), P2 markers (*Vap1*, *Col4a1*, *Sparcl1* and *Sdc1*) and A_reg_ cell-specific marker (*Cd142*, *Gdf10*, *Igfbp3*, *Fmo2* and *Clec11a*) genes in different cell populations; *n* = 3 biological replicates. Data show mean ± s.e.m. **e**, Quantification of adipogenesis (left) and cell number (right) of Lin^−^Sca1^+^ cells, eP1 cells, eP2 cells and eP3 induced by A Cocktail. Data show mean ± s.e.m.; *n* = 6 independent wells. Data were analyzed with one-way ANOVA; *F*(3,20) = 280.8, *P* < 0.0001 (left); multicomparison with Lin^−^Sca1^+^ group was performed by two-stage step-up method with false discovery rate (FDR) = 0.05. *F*(3,20) = 2.838, *P* = 0.064 (right). **f**, Quantification of adipogenesis (left) and cell numbers (right) of Lin^−^Sca1^+^ cells, eP1 cells, eP2 cells and eP3 induced by C Cocktail (1 μM insulin). Data are shown as mean ± s.e.m., *n* = 6 independent wells. Data were analyzed with one-way ANOVA. *F*(3,20) = 48.27, *P* < 0.0001 (left), multicomparison with Lin^−^Sca1^+^ group was performed by two-stage step-up method with FDR = 0.05. *F*(3,20) = 0.189, *P* = 0.903 (right). **g**, Microscopy images of different cell populations shown in **e** and **f** on differentiation day 6. In all panels, nuclei were stained with Hoechst 33342 (blue) and lipids were stained with LD540 (yellow). Scale bars, 100 μm. Source data

To isolate P3 cells more reliably and to further investigate the function of cell populations defined in our combined analysis (Fig. 1a and Extended Data Fig. 1e), we established a new FACS strategy to purify the different subpopulations. As shown in Fig. 2a, enriched-P1 (eP1) composed of P1-1 to P1-3 cells, were isolated using a Lin^−^Sca1^+^CD55^+^VAP1^−^CD142^−^ gating strategy. VAP1^+^ cells were further separated into P2-1 and enriched-P2 (eP2) cells, which are (Lin^−^Sca1^+^CD55^−^VAP1^+^CD142^−^) or P2-2 cells (Lin^−^Sca1^+^CD55^−^VAP1^+^CD142^+^), whereas enriched-P3 (eP3) cells were isolated using a Lin^−^Sca1^+^CD55^−^VAP1^−^CD142^+^ strategy. Upon adipogenic cocktail induction, eP1, eP2 and VAP1^+^CD142^+^ (P2-2) cells showed an adipogenic capacity and we furthermore observed that removal of eP3 cells from Lin^−^Sca1^+^VAP1^−^CD55^−^ (or VAP1^−^CD55^−^) cells, Lin^−^Sca1^+^VAP1^−^CD55^−^CD142^−^ (or DN:CD142^−^) showed markedly increased adipocyte formation, reminiscent of the fact that eP3 cells are refractory toward adipogenesis (Fig. 2b,c). Moreover, gene expression analysis demonstrated that P3-specific genes are enriched in the eP3 population (Fig. 2d). In conclusion, we were able to isolate eP1, eP2, P2-2 and eP3 cells, which represent the P1, P2-1, P2-2 and P3 cells identified by the 10X scRNA-seq approach (Fig. 1a). EP1 cells, when treated with a minimal adipogenic cocktail (C cocktail; Extended Data Table 2) exhibited a lower adipogenic potential compared to eP2 cells, whereas no differences in adipogenesis were detected using robust adipogenic conditions (A cocktail) (Fig. 2e–g). These data imply that eP1 cells, which express low levels of *Pparg* (Extended Data Fig. 1a,b), are at an earlier stage of adipogenesis and might have not committed to the adipocyte lineage.

### Identification of Rspo2 as a new marker of P3 cells

**Fig. 3:**
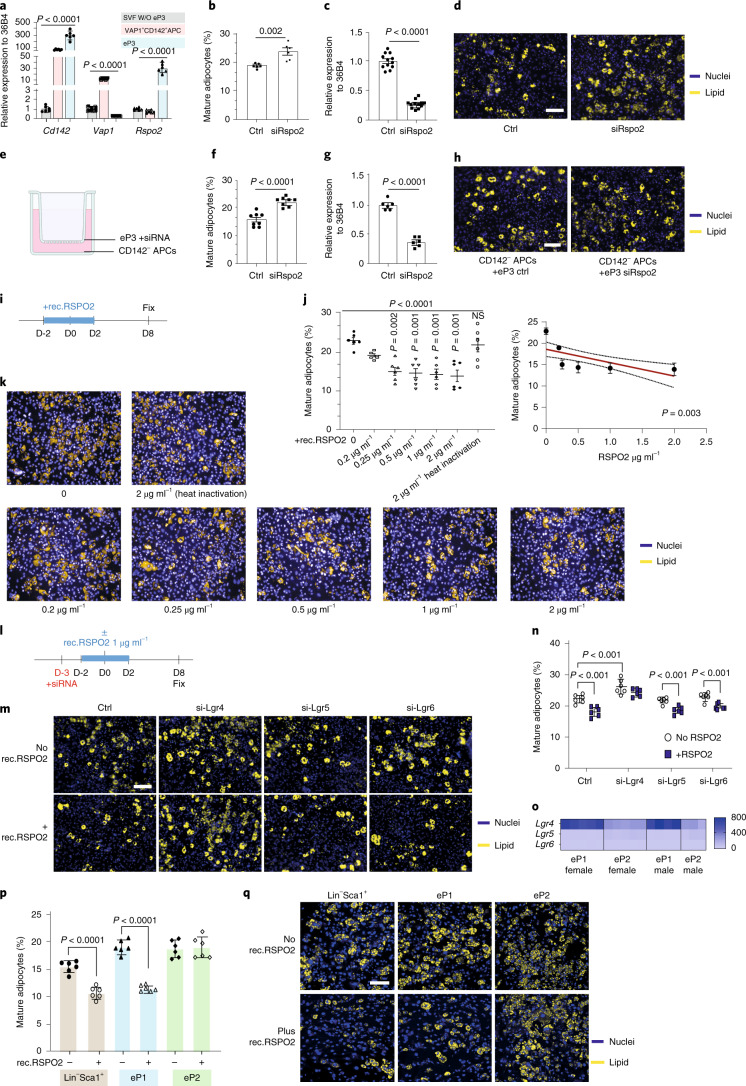
Identification of Rspo2 as a new marker of P3. **a**, *Cd142 Vap1* and *Rspo2* expression in eP3-depleted SVF, VAP1^+^CD142^+^ APCs and eP3. Data show the mean ± s.e.m., *n* = 6 biological replicates. *Cd142*: *F*(2,15) = 88.9; *Vap1*: *F*(2,15) = 686.4; *Rspo2*: *F*(2,15) = 79.5 using one-way ANOVA. **b**–**d**, The ratio (**b**) and representative images (**d**) of adipocytes after knocking down of *Rspo2* in ingWAT SVF. *Rspo2* mRNA expression (**c**) 48 h after transfection. Data show mean ± s.e.m., analyzed by two-tailed Student’s *t*-test. *n* = 2 biological replicates (**b**), *n* = 4 biological replicates (**c**). Ctrl, control. **e**–**h**, Scheme of Transwell co-culture experiments (**e**). The ratio (**f**) and representative images (**h**) of CD142^−^ cells on differentiation day 8. *Rspo2* mRNA levels (**g**) in siRNA-transfected eP3. Data show mean ± s.e.m., analyzed with two-tailed Student’s *t*-test, *n* = 2 biological replicates (**f**,**g**). **i**–**k**, Experimental scheme (**i**) for rec.RSPO2 treatment experiment. The ratio (**j**) and microscopy images (**k**) of adipocytes in SVF-treated ± rec.SPO2. Data shown as mean ± s.e.m., *n* = 6 independent wells. Data were analyzed with one-way ANOVA followed by Tukey’s multiple comparisons test. *F*(6,35) = 10.18. Spearman *r* correlation between RSPO2 level in medium and adipocyte ratio (**j** right). NS, not significant. **l**–**n**, Experimental scheme (**l**) for knocking down of *Lgr4*, *Lgr5* and *Lgr6* in ingWAT SVF treated with or without rec.RSPO2. Representative images (**m**) and the ratio (**n**) of mature adipocytes per well. Data shown as mean ± s.e.m., *n* = 6 independent wells. *F*(3,30) = 1.07, *P* = 0.377 using two-way ANOVA. Multicomparsion between groups was performed by two-stage step-up method with FDR = 0.05. **o**, Heat map of *Lgr4*, *Lgr5* and *Lgr6* expression in eP1 and eP2 cells, *n* = 3–5 biological replicates. **p**–**q**, Ratio (**p**) and representative images (**q**) of adipocytes in cells treated ± rec.RSPO2. Data shown as mean ± s.e.m., *n* = 6 independent wells. *F*(2,20) = 26.22, *P* < 0.0001 using two-way ANOVA. Multicomparison between groups was performed by two-stage step-up method with FDR = 0.05. In all panels, nuclei were stained with Hoechst 33342 (blue) and lipids were stained with LD540 (yellow). Scale bars, 100 μm. Source data

**Extended Data Fig. 3:**
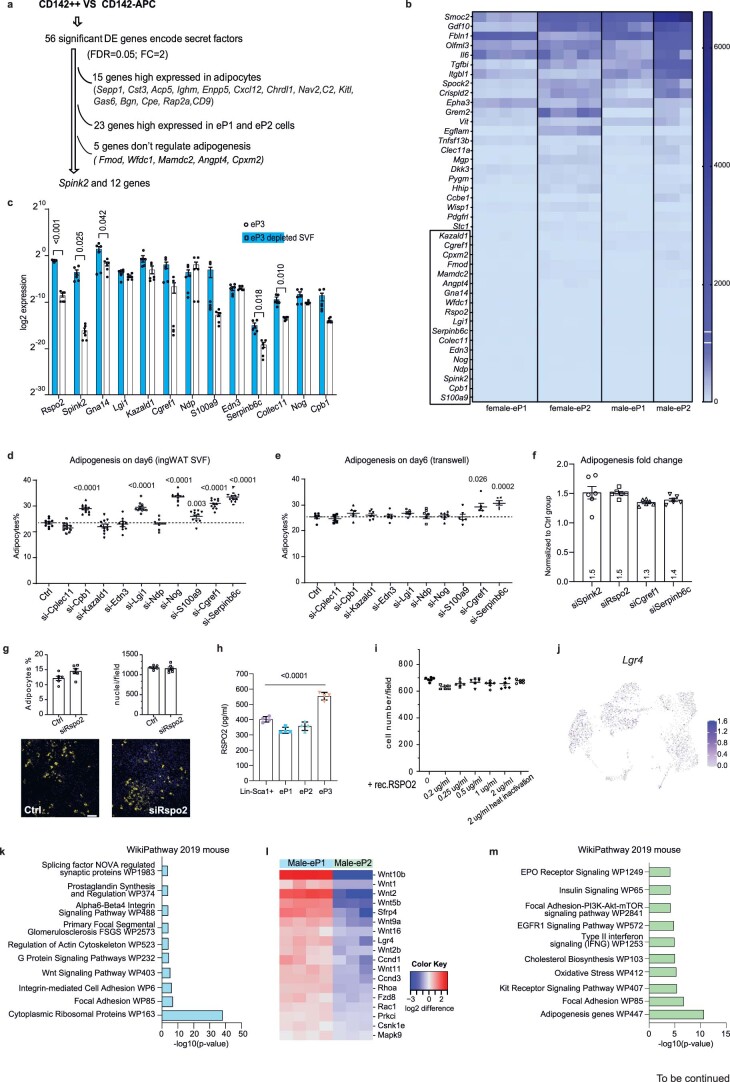

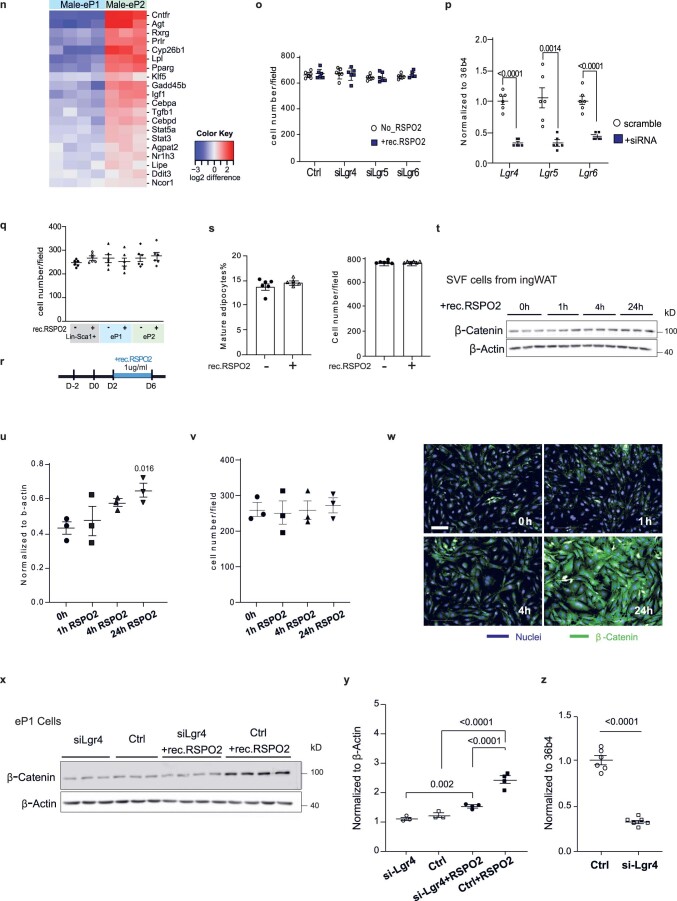
Selection and identification of Aregs’ effectors. **a**–**c**.) Scheme of identification of Aregs marker genes candidates (**a**), and their expression in eP1 and eP2 cells bulk RNAseq data, n = 3-5 biological replicates (**b**). mRNA expression in eP3 and eP3-depleted SVF (**c**). n = 6 biological replicates, data show the mean ± SEM., and analyzed by two-tailed Student’s *t*-test. **d**) Adipocyte ratio in ingWAT SVF after knocking down eP3 marker genes by siRNA. n = 3 biological replicates; data shown as mean ± SEM. and analyzed by two-tailed Student’s *t*-test (compared to Ctrl group). **e**) Adipocyte ratio in CD142- cells co-cultured with eP3. n = 3 biological replicates; data shown as mean ± SEM. and analyzed by two-tailed Student’s *t*-test (compared to Ctrl group). **f**) Comparison of adipogenesis of CD142- cells after knocking down of *Spink2*, *Rspo2*, *Cgref1* and *Serpinb6c* in eP3 cells in transwell co-culture experiments. Adipocyte ratio normalized to ctrl group. n = 3 biological replicates; data is presented as mean ± SEM and analyzed by one way-ANOVA test. *F*(3,20)=2.025, *P* = 0.143. g) Adipocyte ratio in eP3 after knocking down *Rspo2* by siRNA, n = 3 biological replicates. Representative images of adipocytes on differentiation day 7. **h**) RSPO2 conc. in cell culture medium, n = 4 biological replicates. Data is presented as mean + /- SEM and was analyzed by one way-ANOVA test. *F*(3,12)=75; *P* < 0.0001. **i**) Quantification of cell number in Fig. 3j. n = 6 independent wells; data show the mean ± SEM, analyzed by one way-ANOVA test. **j**) Feature plots of *Lgr4* in 10xscRNAseq of ingWAT Lin- cells^9^. **k**) Pathway enriched in eP1 cells by Enrichr analysis. P-value is computed using the Fisher exact test. **l**) Heatmap of log2 fold changes of Wnt signaling related genes in eP1 and eP2 cells. Each row represents 1 gene; each column represents one replicate. **m**) Pathway enriched in eP2 cells by Enrichr analysis. P-value was computed using the Fisher exact test. **n**) Heatmap of log2 fold changes of adipogenesis related genes in eP1 and eP2 cells. Each row represents 1 gene; each column represents one replicate. **o)** Quantification of cell number per field in Fig. 3n. Data show the mean ± SEM, n = 6 independent wells. **p**) *Lgr4-6* mRNA level in cells in Fig. 3n. Data shown as mean ± SEM, n = 6 independent wells. Statistical analysis was performed by two-tailed Student’s *t*-test. **q**) Quantification of cell numbers per field in Fig. 3p. Data shown as mean ± SEM, n = 6 independent wells. **r**) Experimental scheme of treatment cells with rec.RSPO2 during adipogenesis day3 to day6. **s**) Quantification of adipocytes per well (left) and cell number (right) ± rec.RSPO2 during day3 to day6. Data shown as mean ± SEM, n = 6 independent wells. **t**–**w**) SVF cells treated with 0.5ug/ml rec.RSPO2 for 0-24 h. Western blot images (**t**) and quantification (**u**) of beta-Catenin and beta-Actin in SVF. Data shown as mean ± SEM, n = 3 biological replicates. *F*(3,8)=3.85, *P* = 0.057 by one way ANOVA. Multiple comparison between groups was performed using Tukey test with FDR = 0.05. Quantification of cells number/field (**v**) and microscopy images (**w**) in each well treated with rec.RSPO2. Data shown as mean ± SEM, n = 3 biological replicates. Data was analyzed by one way-ANOVA. Experiment was repeated twice. **x**–**z**) After knocking down *Lgr4* by siRNA, eP1 cells were treated with rec.RSPO2 (0.5ug/ml) for 24 h. Western blot images (**x**) and quantification (**y**) of Beta-Catenin protein in eP1 cells. Beta-actin protein levels were used as loading control. *F*(3,10)=52.68, *P* < 0.0001 by one way ANOVA test. Multiple comparison between groups was performed with Tukey FDR = 0.05. *Lgr4* mRNA level (**z**) in eP1 cells 48 h post siRNA transfection. Data shown as mean ± SEM, n = 3-4 biological replicates (**x**, **y**), n = 6 independent wells (**z**). Data analysis was performed using two-tailed Student’s *t*-test (**z**). Nuclei were stained with Hoechst 33342 (blue). Scale bars, 100μm. This figure is related to Fig. 3. Source data

We previously could show that A_reg_/P3 cells regulate adipogenesis in a paracrine fashion through Spink2 and Rtp3 (ref. ^9^). To characterize P3 cells in more detail, we compared bulk RNA-seq of A_reg_ (Lin^−^Sca1^+^CD142^+^^+^) cells to Lin^−^Sca1^+^CD142^−^ cells from mouse ingWAT^9^. A total of 216 differently regulated genes, of which 56 encoded secreted proteins, were enriched in A_reg_/P3 cells (Extended Data Fig. 3a). The list was reduced to 41 genes after exclusion of candidates expressed in mature adipocytes or other cell populations. When filtered for factors expressed in eP1 and eP2 cells, 13 candidates remained (Extended Data Fig. 3b), which were exclusiverly enriched in eP3 cells (Fig. 3a and Extended Data Fig. 3c).

To test the functional relevance of the 13 candidates, we first used siRNA to decrease their expression in ingWAT SVF. We observed increased adipogenesis of SVF after knockdown of *Rspo2* (Fig. 3b–d), *Cpb1*, *Lgi1*, *Nog*, *S100a9*, *Cgref1*, or *Serpinb6c* (Extended Data Fig. 3d). Next, to test which candidates could modulate the inhibitory potential of P3 cells in a paracrine manner, we co-cultured eP3 cells with Lin^−^Sca1^+^CD142^−^ APCs in a Transwell system (Fig. 3e). We noted that short interfering RNA (siRNA)-mediated ablation of *Rspo2* (Fig. 3f–h), *Cgref1* or *Serpinb6c* (Extended Data Fig. 3e) in eP3 cells significantly increased adipogenesis of CD142^−^ APCs in the other compartment. Furthermore, ablation of *Rspo2*, *Cgref1* or *Serbinb6c* lowered the paracrine inhibitory potential of eP3 cells, comparable to the previously identified Spink2 (ref. ^9^) (Extended Data Fig. 3f). Therefore, P3 cells likely regulate adipogenesis in a paracrine fashion through the potential effectors Rspo2, Cgref1, Serbinb6c or Spink2.

### Recombinant RSPO2 protein inhibits adipogenesis through Lgr4 in primary SVF cells

RSPO2 could be detected in eP3 cell culture medium at 500–600 pg ml^−1^ (Extended Data Fig. 3h). To test whether RSPO2 inhibits adipogenesis in vitro, we added recombinant RSPO2 (rec.RSPO2) in an SVF differentiation assay starting from 2 d before until 2 d after cocktail induction (Fig. 3i) and observed that increasing amounts of RSPO2 led to a progressive decrease in adipocyte formation (Fig. 3j,k) without affecting cell numbers (Extended Data Fig. 3i). When rec.RSPO2 was inactivated by heat its inhibitory effect on adipogenesis was lost (Fig. 3j). Among the three receptors of RSPO2 leucine-rich repeat-containing G protein-coupled receptor 4–6 (LGR4–6), we found that only *Lgr4* was expressed at high levels in Lin^−^ cells (Extended Data Fig. 3j). Depletion of *Lgr4* expression induced more adipocyte formation in SVF, whereas knockdown of either *Lgr5* or *Lgr6* did not alter adipogenesis (Fig. 3m,n). Moreover, SVF adipogenesis was not inhibited by rec.RSPO2 when *Lgr4* expression was ablated (Fig. 3m,n).

Rspo2 is an enhancer of the Wnt signaling pathway, which plays a key role in regulation of adipocyte commitment. As single-cell trajectory analysis (Fig. 1c,d) suggests a transition of P1 to committed P2 cells, we next aimed to identify mechanisms that regulate this transition. Enrichr^24,25^ analysis of differentially expressed genes of eP1 and eP2 cells suggest an enrichment of the Wnt signaling pathway in eP1 cells (Extended Data Fig. 3k,l) and adipogenesis genes in eP2 cells (Extended Data Fig. 3m,n). Notably, Rspo2 receptor Lgr4 was enriched in P1 cells compared to P2 cells (Fig. 3o and Extended Data Fig. 3j), which suggests that P1 and not P2 cells might be the target of RSPO2. In accordance with our hypothesis, when exposed to rec.RSPO2 during adipogenesis (Fig. 3i), eP1, but not eP2 cells, exhibited less adipocyte formation (Fig. 3p–q). Because eP1 cells are at an earlier stage of adipogenesis compared to eP2 cells, we assumed that RSPO2 might affect adipocyte commitment and late-phase adipocyte formation. This was confirmed by the finding that adipogenesis was unaltered when cells were exposed to rec.RSPO2 during differentiation from day 3 to 6 (late phase) (Extended Data Fig. 3r–s).

We found that rec.RSPO2 upregulated Wnt signals by inducing β-catenin levels in a time-dependent manner, independent of cell number changes (Extended Data Fig. 3t–w). Similarly, rec.RSPO2 upregulated Wnt signals in eP1 cells 24 h after treatment (Extended Data Fig. 3x,y) and the effect was blunted after ablation of *Lgr4* by siRNA (Extended Data Fig. 3x–z). Collectively, these data demonstrate that RSPO2 inhibits P1 commitment during adipogenesis, possibly by regulation of the Wnt/β-catenin signaling pathway through Lgr4.

### Rspo2 inhibits adipogenesis of eP1 cells in vivo

**Fig. 4:**
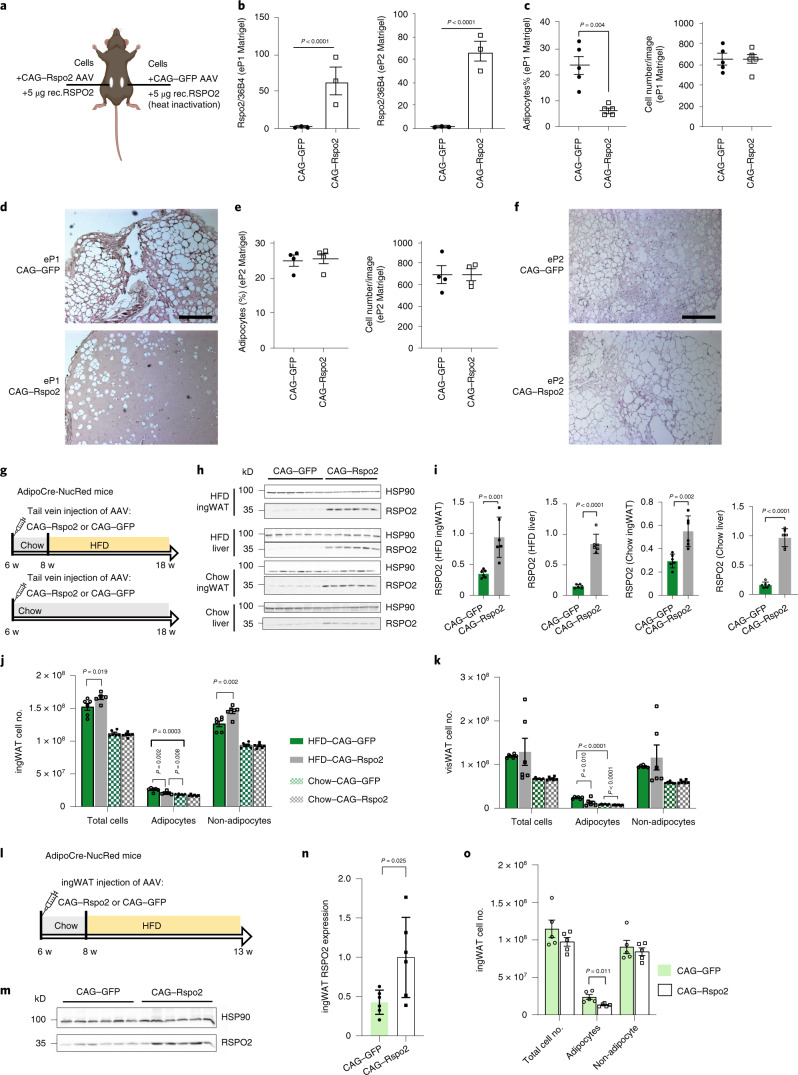
Rspo2 inhibits adipogenesis of eP1 cells in vivo. **a**–**f**, Experimental scheme (**a**) for cell transplantation in Matrigel. *Rspo2* expression in eP1 Matrigel plugs and in eP2 Matrigel plugs (**b**). Quantification of adipocytes and cell number in eP1 Matrigel plugs (**c**) and eP2 Matrigel plugs (**e**). Representative hematoxylin and eosin (H&E) staining of eP1 Matrigel plugs (**d**) and eP2 Matrigel plugs (**f**). Data show mean ± s.e.m., *n* = 3 biological replicates (**b**), *n* = 5 biological replicates (**c**,**e**). Data analysis was performed using a two-tailed Student’s *t*-test. Scale bar, 100 μm. **g**–**k**, Experimental scheme for overexpression of RSPO2 in AdipoCre-NucRed mice fed with HFD or chow diet. Western blot images (**h**) and quantification (**i**) of RSPO2 protein in liver and ingWAT; HSP90 bands were used as loading control. Quantification of adipocyte numbers in ingWAT (**j**) and visWAT (**k**) of mice shown in **g**. Data are shown as mean ± s.d., *n* = 6 mice. Data analysis was performed by two-tailed Student’s *t*-test (**i**) and one-way ANOVA (**j**,**k**). In **j**, Total cell number, *F*(3,20) = 14.4, *P* < 0.0001; adipocyte, *F*(3,20) = 15.50, *P* < 0.0001; non-adipocyte, *F*(3,20) = 14.1, *P* < 0.0001. In **k**, total cell number, *F*(3,20) = 14.4, *P* < 0.0001; adipocyte, F(3,20) = 15.50, *P* < 0.0001; non-adipocyte, F(3,20) = 14.1, *P* < 0.0001. **l**–**o**, Experimental scheme (**l**) for overexpression of RSPO2 in ingWAT by injection of AAV into ingWAT of AdipoCre-NucRed mice. Western blot images (**m**) and quantification (**n**) of RSPO2 protein in ingWAT of mice shown in **l**. HSP90 bands were used as loading control. Quantification of cell numbers by quantitative PCR in ingWAT (**o**). Data shows mean ± s.d., *n* = 5–6 mice. Data were analyzed using a two-tailed Student’s *t*-test. Source data

**Extended Data Fig. 4:**
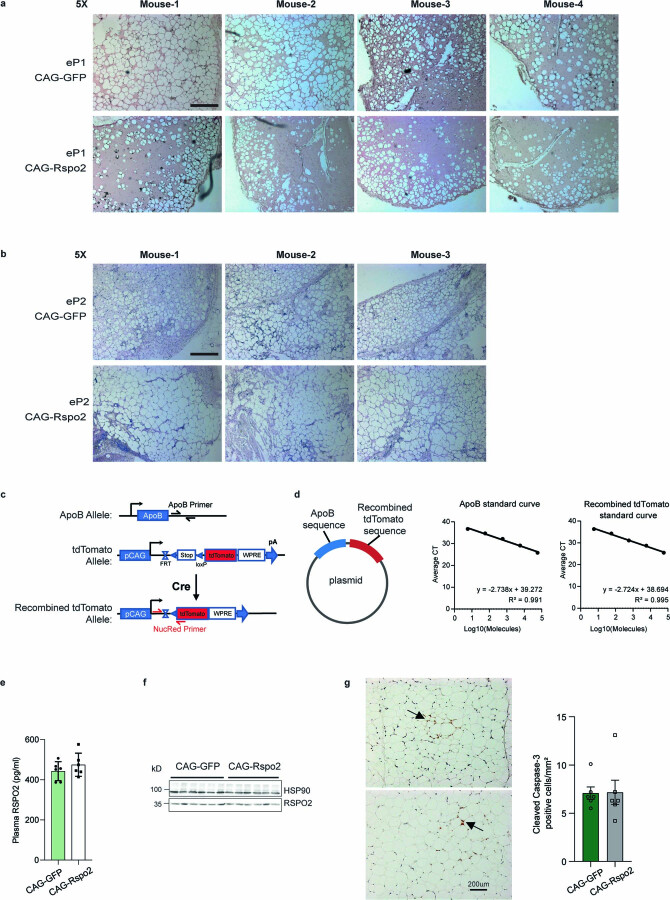
Rspo2 inhibits adipogenesis of eP1 cells *in vivo*. **a**, **b**) Microscopy images of H&E staining of matrigel plugs with eP1 cells (**a**) and eP2 cells (**b**) without RSPO2 overexpression (top) and with RSPO2 overexpression (lower). Scale bar, 100 μm. Experiment was performed once. **c**, **d**) pUC57 plasmid insertion with ApoB and recombined tdTomato sequence. ApoB and recombined tdTomato standard curve generated with plasmid (d). e) Plasma RSPO2 level in CAG-GFP and CAG-Rspo2 ingWAT infection mice, n = 6 mice. Data are presented as mean values + /- SEM. Data analysis was performed using two-tailed Student’s *t*-test. **f**) Western blot images of RSPO2 in liver of ingWAT AAV infection mice. HSP90 served as a loading control. **g**) Immunohistochemistry staining of cleaved caspase-3 in ingWAT. Data are presented as mean values + /- SEM, n = 6 mice. This figure is related to Fig. 4. Source data

To extend our data to the in vivo situation, we first generated an adeno-associated virus (AAV) system to express RSPO2 under the chicken β-actin promoter (CAG), while pAAV–CAG–GFP was used as infection control. Next, eP1 or eP2 cells from ingWAT were resuspended in Matrigel, which contained either pAAV–CAG–Rspo2 or pAAV–CAG–GFP and was transplanted subcutaneously into mice (Fig. 4a). In addition, rec.RSPO2 was supplemented into the Matrigel to ensure that cells were exposed to RSPO2 during the initial phases of adipogenesis as AAV-mediated expression requires at least 5 d^26^. Mice were exposed to a high-fat diet (HFD) for 4 weeks to induce adipocyte formation after transplantation. pAAV–CAG–Rspo2 significantly increased *Rspo2* messenger RNA levels in Matrigel plugs (Fig. 4b) and reduced adipocyte formation of eP1 cells (Fig. 4c,d and Extended Data Fig. 4a). In accordance with the in vitro data, RSPO2 did not inhibit adipogenesis of eP2 cells in mice (Fig. 4e,f and Extended Data Fig. 4b).

To investigate adipocyte formation within ingWAT during obesity, 6-week-old AdipoCre-NucRed mice received either pAAV–CAG–GFP or pAAV–CAG–Rspo2 by tail-vein injection and 2 weeks after infection, mice were switched to HFD or continued on a chow diet for another 10 weeks (Fig. 4g). By using AdipoqCre-NucRed transgenic mice we quantified adipocyte numbers using qPCR^27^ (Extended Data Fig. 4c,d). At 12 weeks after AAV infection, higher RSPO2 level were detected in liver and ingWAT (Fig. 4h,i). At 10 weeks after HFD, adipocyte numbers significantly increased in ingWAT (Fig. 4j; HFD CAG–GFP group versus chow CAG–GFP group). Meanwhile, a reduced number of adipocytes was detected in RSPO2 overexpression mice (Fig. 4j; HFD CAG–GFP group versus HFD CAG–RSPO2 group). Higher levels of RSPO2 in chow-diet-fed mice did not alter adipocyte numbers in ingWAT. Moreover, in visWAT, reduced adipocyte numbers were detected in RSPO2-overexpressing mice under both HFD and chow diet conditions (Fig. 4k).

Tail-vein-mediated delivery of AAVs led to RSPO2 overexpression in ingWAT, but also in the liver, circulation and possibly other organs. Thus, to achieve RSPO2 overexpression within ingWAT, AAVs were injected directly into ingWAT (Fig. 4l), which led to a twofold increase in RSPO2 protein levels in ingWAT (Fig. 4m,n) with a minimal increase in circulating RSPO2 (Extended Data Fig. 4e). RSPO2 expression in the liver was not affected (Extended Data Fig. 4f). As a result of RSPO2 overexpression, HFD-induced adipocyte formation was decreased within ingWAT (Fig.4o).

To evaluate whether Rspo2 increased adipocyte apoptosis, which in turn might reduce adipocyte numbers, staining for the apoptosis marker cleaved caspase-3 (ref. ^28^) was performed in ingWAT. We did not observe any significant difference of adipocyte apoptosis between HFD CAG–GFP mice and HFD CAG–Rspo2 mice (Extended Data Fig. 4g). Collectively, these data suggest that Rspo2 inhibits adipocyte formation in HFD-induced obesity.

### Rspo2 inhibits transition of P1 cells to P2 cells in vivo

**Fig. 5:**
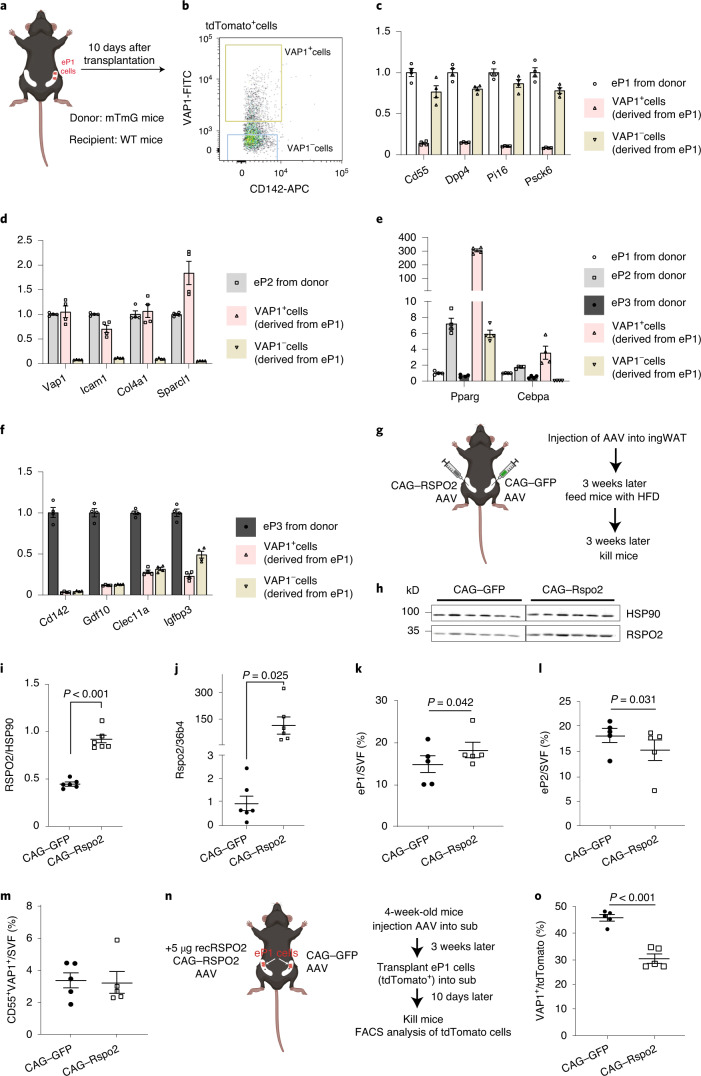
Rspo2 inhibits transition of eP1 cells to eP2 cells. **a**–**f**, Experimental scheme (**a**) for transplantation of tdTomato^+^ eP1 cells into inguinal adipose tissue of wild-type (WT) mice. FACS analysis (**b**) of VAP1 and CD142 expression in tdTomato^+^ eP1 cells 10 d after transplantation. Expression of P1 marker genes (**c**) (*Cd55*, *Dpp4*, *Pi16* and *Psck6*), P2 marker genes (**d**) (*Vap1*, *Icam1*, *Col4a1* and *Sparcl1*), *Pparg* and *Cebpa* (**e**) and P3 marker genes (**f**) (*Cd142*, *Gdf10*, *Clec11a* and *Igfbp3*) in eP1 cells (from donor mice), eP2 cells (from donor mice), eP3 cells (from donor mice), VAP1^+^ cells (derived from implanted eP1 cells) and VAP1^−^ cells (derived from implanted eP1 cells). Data are shown as mean ± s.e.m., *n* = 4 biological replicates. **g**–**m**, Experimental scheme (**g**) for injection of AAVs into ingWAT for overexpression of RSPO2. Western blot images (**h**) and quantification (**i**) of RSPO2 protein and *Rspo2* mRNA (**j**) in ingWAT. FACS analysis of eP1/SVF (**k**), eP2/SVF (**l**), CD55^+^VAP1^+^ (**m**) in ingWAT. Data are shown as mean ± s.e.m., *n* = 6 mice (**h**,**i**), *n* = 5–6 mice (**j**), *n* = 5 mice (**k**–**m**). Data were analyzed using two-tailed Student’s *t*-test. **n**,**o**, Experimental scheme (**n**) for transplantation of tdTomato^+^ eP1 cells into RSPO2 overexpression mice. FACS analysis of (VAP1^+^:tdTomato^+^) cells in tdTomato^+^ eP1 cells (**o**). Data are shown as mean ± s.e.m., *n* = 5 biological replicates. Data were analyzed using a two-tailed paired Student’s *t*-test. Source data

Single-cell trajectory analysis (Fig. 1c,d) as well as previous work^4,9^, suggests that P1 cells can transition to P2 cells. To establish a model to study the transition of P1 to P2 cells in vivo, we isolated tdTomato^+^ eP1 cells from ROSA^mT/mG^ mice and transplanted them into ingWAT of wild-type mice (Fig. 5a). Ten days after transplantation, flow cytometry analysis demonstrated that approximately 23% of implanted eP1 cells had transitioned into eP2 cells (Fig. 5b). A careful evaluation of VAP1^+^ P2 cells, derived from eP1 cells, showed that they lost expression of P1 markers (*CD55*, *Dpp4*, *Pi16* and *Pcsk6*) (Fig. 5c) and acquired expression of P2 markers (*Vap1*, *Icam1*, *Col4a1* and *Sparcl1*) (Fig. 5d) as well as committed pre-adipocytes markers such as *Pparg* and *Cebpa* (Fig. 5e). However, neither VAP1^+^ nor VAP1^−^ cells derived from eP1 cells, expressed P3 markers (Fig. 5f). These experiments validate our model system as a tool to study the P1 to P2 transition in vivo.

When pAAV–CAG–Rspo2 was injected into one depot of ingWAT in mice, while the other depot received pAAV–CAG–GFP as a control (Fig. 5g), RSPO2 protein levels were twofold higher (Fig. 5h,i), whereas mRNA levels were around 100-fold higher than pAAV–CAG–GFP-injected depots (Fig. 5j) 6 weeks after infection. FACS analysis revealed higher numbers of eP1 cells (Fig. 5k) and lower numbers of eP2 cells in the RSPO2-overexpressing ingWAT depot (Fig. 5l), even though CD55^+^VAP1^+^ cell numbers did not differ (Fig. 5m) and the eP1/eP2 ratio suggests that Rspo2 inhibited eP1 to eP2 conversion. As eP1 or SVF cell numbers did not change when treated with rec.RSPO2 in vitro (Extended Data Fig. 3i,s) we hypothesized that the increase of eP1 cells might not be due to elevated proliferation. Next, we transplanted eP1 cells from ROSA^mT/mG^ mice into the ingWAT in which RSPO2 expression was modulated by AAVs (Fig. 5n). Ten days after cell transplantation, flow cytometry analysis of transplanted tdTomato^+^ cells showed that approximately 45% of eP1 cells acquired P2 marker gene (*Vap1*) expression, demonstrating that they had transitioned to P2 cells, whereas only 30% of eP1 cells acquired P2 marker gene expression in RSPO2-overexpressing ingWAT (Fig. 5o). Taken together, cellular crosstalk of different subpopulations within the adipose tissue SVF fine-tunes adipocyte formation by regulating maturation of early progenitor cells to committed preadipocytes through paracrine RSPO2-mediated changes in the Wnt signaling pathway.

### Rspo2 leads to unhealthy adipose tissue expansion and insulin resistance in vivo

**Fig. 6:**
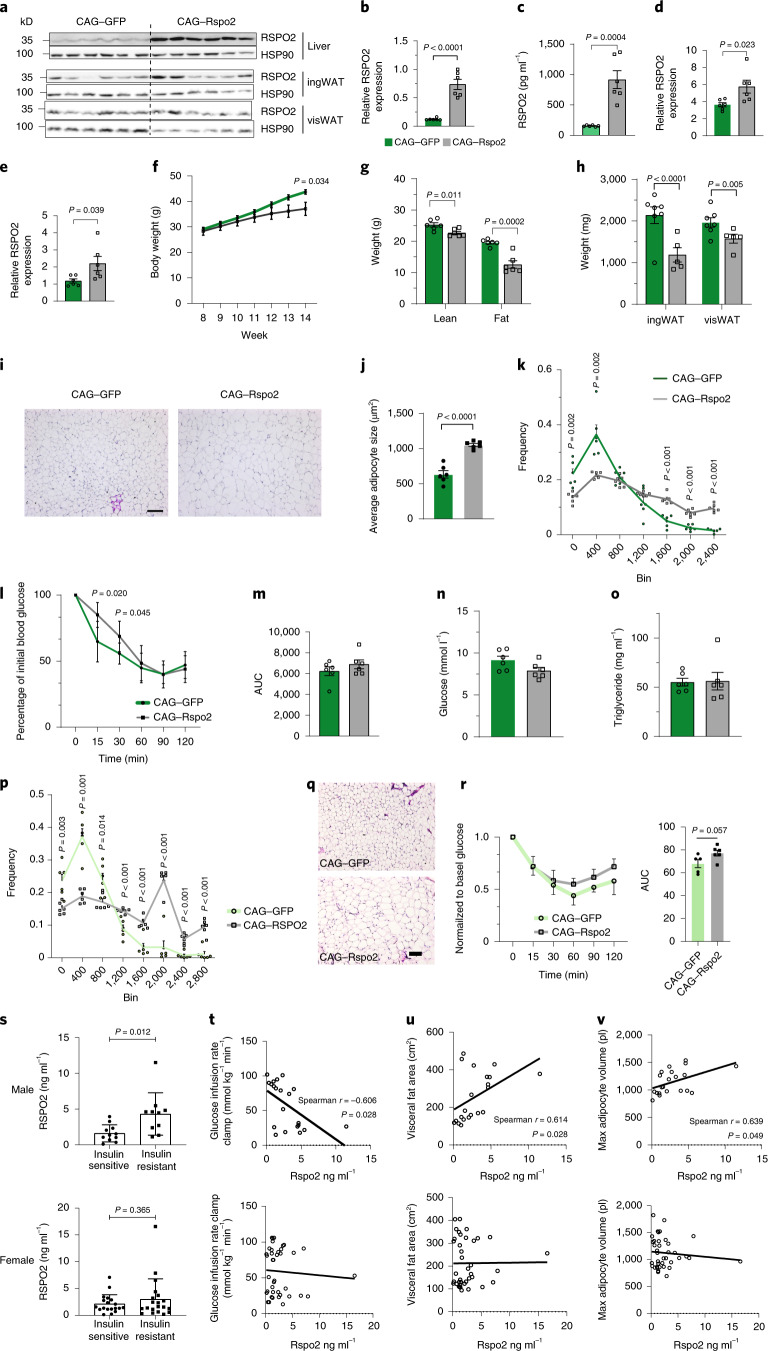
Circulating RSPO2 leads to unhealthy expansion of adipose tissue and insulin resistance in vivo. **a**–**o**, RSPO2 overexpression in mice by tail-vein delivery of pAAV–CAG–Rspo2. Representative immunoblots (**a**) and quantification of RSPO2 and HSP90 in liver (**b**), circulation (**c**), ingWAT (**d**) and visWAT (**e**) in RSPO2-overexpression mice. Body weight curve (**f**), lean mass and fat mass (**g**) and ingWAT and visWAT tissue weight (**h**) of AAV-infected mice. Representative H&E staining images (**i**), average of adipocytes size (μm^2^) and adipocyte size frequency distribution of ingWAT. Blood glucose normalized to initial blood glucose after insulin injection (**l**) in ITT and area under the curve (AUC) was quantified as shown in **m**. Fasting blood glucose (**n**) and triglycerides (**o**) in AAV-injected mice. Data are shown as mean ± s.e.m., *n* = 6 mice. Data were analyzed using a two-tailed Student’s *t*-test. Scale bar, 100 μm. **p**–**r**, RSPO2 overexpression by injection into ingWAT. Adipocyte size frequency distribution (**p**) and representative H&E staining of ingWAT. Data are shown as mean ± s.e.m. Glucose levels in blood in ITT and glucose was normalized to time point 0 (**r**). Data are shown as mean ± s.d. Comparison of AUC (**r**, right) in ITT. Data are shown as mean ± s.e.m., *n* = 5 mice (CAG–GFP), *n* = 6 mice (CAG–Rspo2). Data analysis was performed using a two-tailed Student’s *t*-test. **s**, Circulating RSPO2 levels in insulin-sensitive and insulin-resistant individuals. Data are shown as mean ± s.d., *n* = 11 (male, insulin sensitive), n = 10 (male, insulin resistant), *n* = 18 (female, insulin sensitive), n = 21 (male, insulin resistant). Data analysis was performed using a two-tailed Student’s *t*-test. **t**–**v**, Spearman correlation coefficient analysis of circulating RSPO2 and glucose infusion rate (**t**), visceral fat area (**u**) and max adipocyte volume (**v**). *P* values are corrected by two-stage step-up method of Benjamini, Krieger and Yekutieli with an FDR = 0.05. Source data

**Extended Data Fig. 5:**
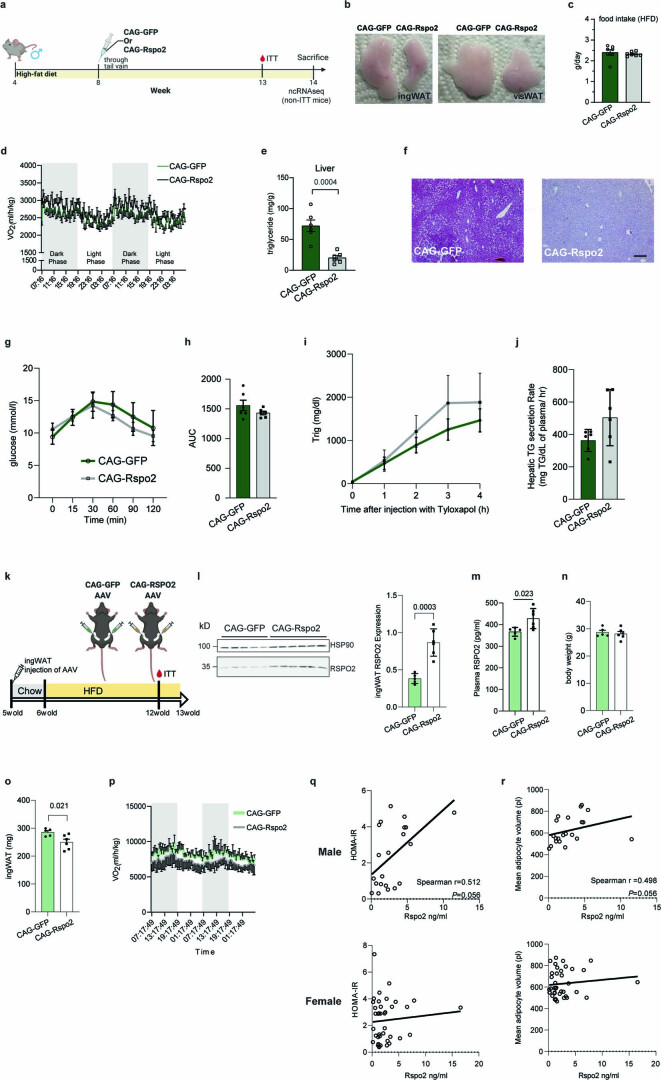
pAAV–CAG-GFP and pAAV-CAG-Rspo2 infection mice on HFD. **a**–**j**) Overexpression of RSPO2 by tail vein delivery of AAVs in mice (**a**). Images of ingWAT and visWAT (**b**), food intake per day (**c**), Time-resolved oxygen consumption (d), quantification of triglyceride per gram of liver (**e**), representative images of H&E staining of liver (**f)** from pAAV-CAG-GFP and pAAV-CAG-Rspo2 infection mice. Blood glucose (**g**) and area under curve (AUC) (**h**) shown by intraperitoneal pyruvate tolerance test. Triglyceride levels in blood (**i**) and hepatic triglyceride secretion rate (**j**) after tyloxapol injection. Data shown as mean ± SD (**i**, **j**), n = 6 mice. Data analysis was performed using two-tailed Student’s *t*-test. **k**–**p**) Experimental scheme (**k**) for overexpression of RSPO2 in ingWAT by injection AAV into ingWAT. Quantification of RSPO2 in ingWAT by western blot (**l**) and in circulation (**m**). Body weight (**n**), tissue weight (**o**), and time-resolved oxygen consumption (**p**) of pAAV-CAG-GFP and pAAV-CAG-Rspo2 ingWAT infection mice. Data shown as mean ± SD, n = 5 mice (CAG-GFP); n = 6 mice (CAG-Rspo2). Data analysis was performed using two-tailed Student’s *t*-test. **q**–**r**) Spearman correlation of serum RSPO2 level with HOMA-IR (**q**), mean adipocyte volume (**r**) in male and female subjects. The correlation coefficient was calculated using a Spearman’s Correlation Test. This figure is related to Fig. 6. Source data

As Rspo2 inhibits adipocyte formation in vivo, we next investigated, whether Rspo2 influences adipose tissue expansion during obesity. Therefore, pAAV–CAG–Rspo2 was injected into 8-week-old diet-induced obese mice (Extended Data Fig. 5a), which led to a fivefold increase in RSPO2 protein levels in the liver (Fig. 6a,b) and plasma (Fig. 6c) and a twofold increase in ingWAT (Fig. 6d) and visWAT (Fig. 6e). We observed decreased weight gain in pAAV–CAG–Rspo2-infected mice (Fig. 6f), accompanied by reduced fat mass (Fig. 6g) both in ingWAT and visWAT (Fig. 6h and Extended Data Fig. 5b) independent of food intake (Extended Data Fig. 5c) or energy expenditure (Extended Data Fig. 5d). In addition, RSPO2 overexpression not only reduced adipocyte formation (Fig. 4j) but also led to adipocyte hypertrophy (Fig. 6i–k). Notably, even though RSPO2 overexpression reduced weight gain, higher levels of RSPO2 exhibited a worsened insulin sensitivity during an insulin tolerance test (ITT) (Fig. 6l,m), without affecting fasting blood glucose (Fig. 6n) or hepatic glucose secretion (Extended Data Fig. 5g,h). Fasting triglyceride (TG) levels were unaltered between the two groups (Fig. 6o), whereas less TG accumulated in the livers of RSPO2-overexpression mice (Extended Data Fig. 5e,f) independent of any changes in hepatic TG secretion (Extended Data Fig. 5i,j). These data suggest that adipocyte hypertrophy due to increased RSPO2 levels might be one factor contributing to the worsened metabolic phenotype.

Therefore, we next investigated whether RSPO2 overexpression might impair insulin sensitivity in mice whose ingWAT was targeted by pAAV–CAG–Rspo2 to increase intra-tissue RSPO2 levels (Extended Data Fig. 5k). RSPO2 overexpression in ingWAT (Extended Data Fig. 5l) did not affect body weight (Extended Data Fig. 5n) but slightly decreased ingWAT tissue weight (Extended Data Fig. 5o) without altering energy expenditure (Extended Data Fig. 5p). Furthermore, an increased number of large adipocytes was observed in ingWAT in RSPO2 overexpressing mice after 7 weeks of HFD feeding (Fig. 6p–q). However, the observed hypertrophy of the ingWAT did not impair insulin sensitivity (Fig. 6r). Taken together, we show that Rspo2 might affect systemic insulin sensitivity, possibly in part by regulation of de novo adipocyte formation and adipose tissue expansion.

### Serum RSPO2 correlates with insulin resistance in individuals with obesity

Given the fact that higher circulating RSPO2 in obese mice led to insulin resistance, we queried this association using serum from obese metabolically healthy and unhealthy individuals. Sixty patients (body mass index (BMI) = 45.6 ± 5.6 kg m^−^^2^) were divided into an insulin-sensitive group (HOMA-IR = 0.9 ± 0.4) and an insulin-resistant group (HOMA-IR = 3.8 ± 0.9). In men, RSPO2 levels were significantly higher in the insulin-resistant group and we observed the same trend in women (Fig. 6s). Similar to the mouse study, circulating RSPO2 levels exhibited an inverse correlation with the glucose infusion rate (Fig. 6t) in men but not women. In line with the observation from mice, we noted that circulating RSPO2 levels correlated with the visceral fat area (Fig. 6u) and maximal adipocyte volume (Fig. 6v) in men but not women.

### Single-nucleus sequencing revealed Rspo2 inhibit adipocyte formation in vivo

**Fig. 7:**
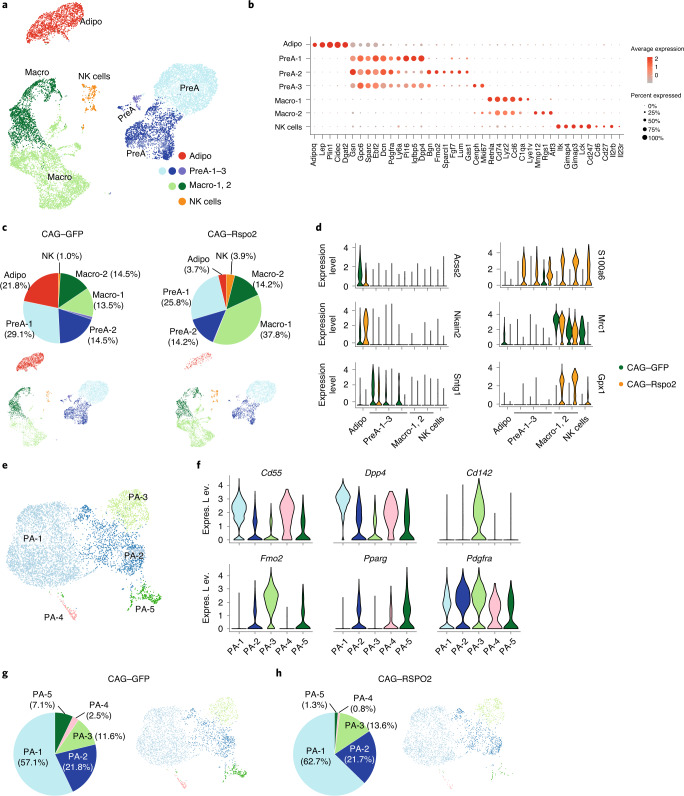
snRNA-seq reveals Rspo2 reducing adipocytes number in vivo. **a**, Integrated analysis of snRNA-seq, including 14,303 nuclei from ingWAT in mice fed on HFD with chronic expression of GFP or RSPO2 by AAV, yielding 2,218 genes (median). Unsupervised clustering shown as a UMAP plot, seven populations were identified, including adipocytes (adipo) (red), pre-adipocytes (PreA) (blue), macrophages (macro) (green) and natural killer (NK) cells (orange). **b**, Dot plots for representative markers of each cluster. Expression level (indicated by red color) refers to the log normalized ratio of gene expression reads, normalized to the sum of all reads within each nucleus. Percent expressed refers to the ratio of cells within each cluster that express the genes listed in *x* axis. **c**, Cluster compositions in CAG–GFP (*n* = 7,190 nuclei) and CAG–Rspo2 (*n* = 7,143 nuclei) conditions. **d**, Violin plots for *Acss2*, *Nkain2*, *Sntg1*, *S100a6*, *Mrc1* and *Gpx1*, which are differentially expressed between CAG–GFP and CAG–Rspo2 conditions. **e**, Subclustering analysis of preadipocyte populations. Unsupervised subclustering of 6,411 preadipocyte nuclei from ingWAT, yielding 2,577 (median) genes. Five subpopulations of preadipocytes (PA-1–PA-5) were identified. **f**, Feature plots for *Dpp4*, *Pparg* and *Fmo2*, shown as separated plots by conditions. **g**,**h**, Pre-adipocyte cluster compositions in CAG–GFP (*n* = 3,539 nuclei) and CAG–Rspo2 (*n* = 2,872 nuclei) conditions.

**Extended Data Fig. 6:**
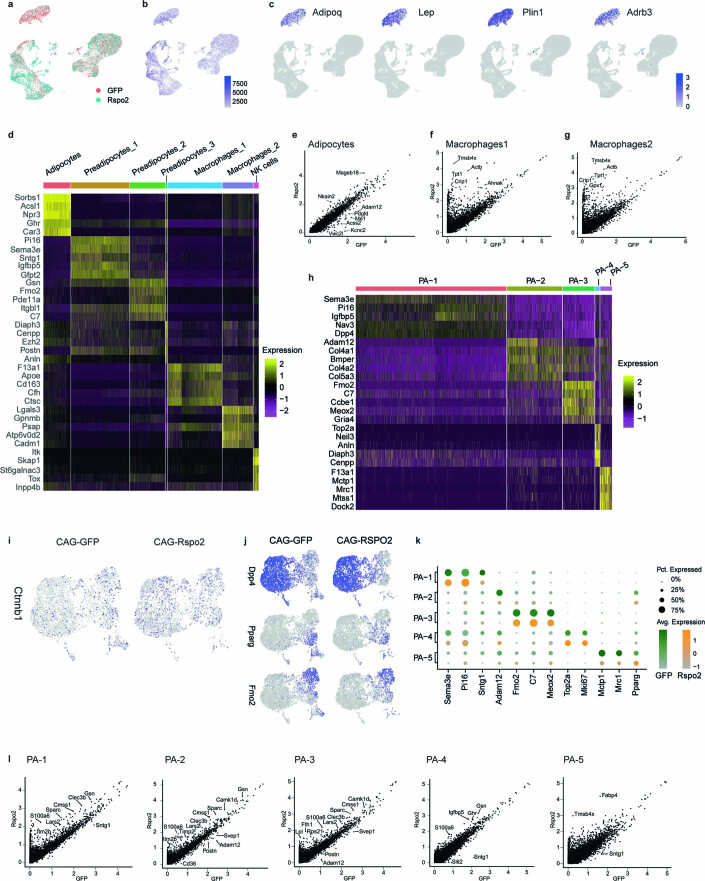
snRNA-seq of ingWAT in mice with HFD and pAAV-CAG-GFP or pAAV-CAG-Rspo2 AAV. **a**) Feature plot for the origin of pAAV-CAG-GFP or pAAV-CAG-Rspo2 conditions. **b**) Feature plot for the number of genes expressed in each nucleus. **c**) Feature plots for adipocyte markers *Adipoq, Lep, Plin1*, and *Adrb3*. **d**) Heat map of signature genes for each population. **e**–**g**) Scatter plots for gene expression analysis between pAAV-CAG-GFP and pAAV-CAG-Rspo2 within adipocytes (**e**), macrophages-1 (**f**), and macrophages-2 (**g**). **h**) Heat map of signature genes for preadipocytes population PA-2-PA-5. **i**) Feature plots for *Ctnnb1* in preadipocytes clusters in pAAV-CAG-GFP and pAAV-CAG-Rspo2 ingWAT, separated by conditions. **j**) Feature plots for *Dpp4*, *Pparg*, *Fmo2* in preadipocytes clusters in pAAV-CAG-GFP and pAAV-CAG-Rspo2 ingWAT. **k**) Dot plots for representative markers of each cluster PA1-PA-5, separated by conditions. **l**) Scatter plots for gene expression analysis between pAAV-CAG-GFP and pAAV-CAG-Rspo2 within PA-1-PA-5. This figure is related to Fig. 7.

To comprehensively evaluate the effects of Rspo2 on different APC populations we performed 10X snRNA-seq on nuclei isolated from ingWAT of pAAV–CAG–GFP- and pAAV–CAG–Rspo2-infected mice. Unsupervised clustering identified seven clusters of cells (Fig. 7a), which were annotated on the basis of known cell marker genes (Fig. 7b). Among all clusters, adipocyte markers (*Adipoq*, *Lep*, *Plin1*, *Cidec* and *Dgat2*) were found in the adipocyte clusters (Fig. 7b and Extended Data Fig. 6c), while pre-adipocyte markers (*Ly6a* and *Pdgfra*) were found in the APC clusters (Fig. 7b). 10X snRNA-seq analysis revealed reduced adipocyte numbers (Fig. 7c) in pAAV–CAG–Rspo2-infected ingWAT, which underscores our previous findings and suggests that RSPO2 can inhibit adipocyte formation. Besides reducing adipocyte numbers, RSPO2 facilitated macrophage recruitment into ingWAT (Fig. 7a,c), which might contribute to the observed insulin resistance. RSPO2 overexpression also affected many of the identified clusters with regards to their gene expression profile (Fig. 7d and Extended Data Fig. 6e–g).

Next, we clustered pre-adipocyte nuclei into five subpopulations named PA-1–PA-5 (Fig. 7e). PA-1 represent noncommitted APCs (P1) with expression of P1 marker genes (Fig. 7f and Extended Data Fig. 6h), *Sema3e*, *Pi16* (Extended Data Fig. 6h) and low *Pparg* expression (Fig. 7f). PA-2 represents the P2-2 population. Even though P2-2 marker *Vap1* or *Icam1* were barely detectable in PA-2 nuclei (data not shown), the committed preadipocyte marker *Pparg* was highly expressed in PA-2 nuclei (Fig. 7f). Similar to P2-2, we observed that some PA-2 nuclei expressed P3 markers such as *Fmo2* and *Cd142* (Fig. 7f and Extended Data Fig. 6h). The PA-3 population represents the P3 population based on expression of marker genes *Cd142*, *Fmo2* and *Meox2* (Fig. 7f and Extended Data Fig. 6h). The PA-4 population defines a cluster of proliferating cells with high levels of cell-cycle genes such as *Top2a* and *Mki67* (Extended Data Fig. 6k). PA-5 represents another cluster of committed pre-adipocytes, which expresses *Pparg* (Fig. 7f and Extended Data Fig. 6j). Overexpression of RSPO2 led to more active Wnt signaling indicated by higher expression of *Cttnb1* (Extended Data Fig. 6i) and led to a higher proportion of adipocyte progenitors (PA-1) and reduced committed pre-adipocytes PA-5 (Fig. 7g,h). These data are in line with our cell transplant experiments, which suggest that RSPO2 inhibits P1 transition into committed preadipocytes (Fig. 5n,o). Collectively, our data suggest that Rspo2 inhibits adipocyte formation during obesity, which leads to adipocyte hypertrophy and macrophage infiltration into adipose tissue. We propose that a combination of these factors contribute to the development of insulin resistance in RSPO2-overexpressing mice.

## Discussion

Adipogenesis follows a highly ordered process that initiates during embryogenesis and persists throughout life^1^, requiring coordination of multiple regulatory signaling pathways^29,30^ and cell types. Recent studies using scRNA-seq have reported mouse and human adipose tissue cell-type composition^4,9–11,19,31^.

Notably, P3 cells similar to P1 cells express *Lgr4*, the target receptor of RSPO2 (Extended Data Fig. 3j), suggesting that the failure of P3 to give rise to adipocytes might stem from an autocrine inhibitory mechanism, similar to a stem cell niche regulating its own stemness. However, we observed that loss of *Rspo2* in P3 cells did not increase adipocyte formation (Extended Data Fig. 3g), which suggests that the stemness of P3 cells can be maintained by other signals.

While our study mainly focuses on SAT, adipogenesis regulatory cell populations were also reported in muscle^32^ and visWAT^11^. A P3-resembling cell population was identified in muscle and it was shown that these cells were capable of inhibiting adipocyte formation from precursor cells in the context of muscular dystrophy^32^. Hepler et. al.^11^ furthermore identified an anti-adipogenic PDGFRβ^+^ population within visWAT, which exerts a proinflammatory phenotype named fibroinflammatory progenitor cells (FIPs). Shao et. al.^7^ recently described the adipogenic differences of PDGFRβ^+^ cells derived from ingWAT and visWAT, which indicate that both depots utilize different cellular and molecular mechanisms to regulate adipogenesis. A study on spatially resolved transcriptional profiling with scRNA-seq of human WAT^31^ suggests the presence of P3-like cells (C09 cluster), which were enriched in areas close to macrophages and linked to fibrotic/vascular structures, similar to the mouse P3 population^9^, suggesting that other regulatory cell types exist that might share some characteristics of ingWAT P3 cells.

De novo adipocyte formation within ingWAT is associated with age and gender. ^15^N-thymidine-labeled newly formed adipocytes were detected in both ingWAT and visWAT in mice fed with HFD from 4 weeks of age^33^. Robust adipogenesis was reported in ingWAT of female mice exposed to HFD, but not in male mice. When discussing these data, we should keep in mind that newly formed adipocytes might be considered as existing adipocytes due to incomplete tamoxifen washout or newly formed adipocytes derived from precursor cells, which did not proliferate possibly due to a cell conversion from P1 to P2, which might not rely on proliferation. Therefore, other methods to examine adipogenesis within ingWAT during obesity will be required. Quantification of the absolute adipocyte numbers of the complete depot by qPCR in this context could pose an unbiased strategy to investigate adipocyte formation during adipose tissue expansion^27^.

**Extended Data Fig. 7:**
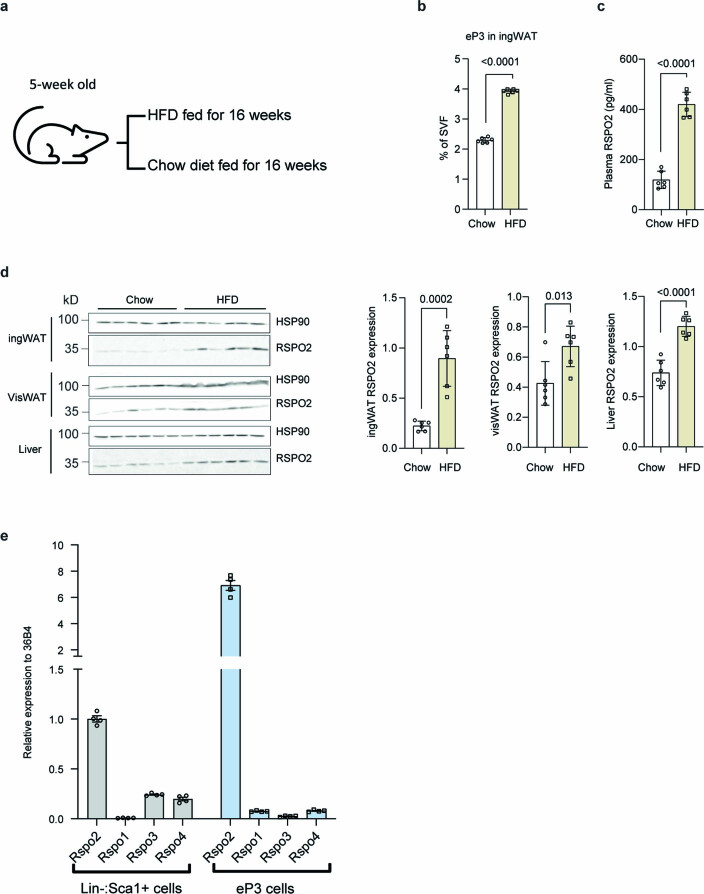
other markers expression in subpopulations of adipogenitors. **a**) Scheme for HFD induced obesity. **b**) eP3 percentage in ingWAT quantified by FACS. Data show as mean ± SD, n = 6 biological replicates. Data was analyzed using two-tailed Student’s *t*-test. **c**) Circulating RSPO2 levels in chow and HFD fed mice. Data shown as mean ± SD, n = 6 biological replicates. Data was analyzed using two-tailed Student’s *t*-test. **d**) RSPO2 expression quantified by western blot in ingWAT, visWAT and liver. HSP90 served as loading control. Data show the mean ± SD, n = 6 biological replicates. Data was analyzed by two-tailed Student’s *t*-test. **e**) *Rspo1-4* mRNA levels in Lin-Sca1+ cells and eP3 cells of ingWAT. Data are presented as mean + /- SEM, n = 4 biological replicates. Data was analyzed by one-way ANOVA, in Lin-Sca1+ group, F(3,12)=602, *P* < 0.0001; in eP3 group, F(3,12)=326, *P* < 0.0001. Source data

HFD mice have more eP3 cells (Extended Data Fig. 7a,b) in ingWAT and express higher levels of RSPO2 in circulation and adipose tissue (Extended Data Fig. 7c,d), suggesting that eP3 or RSPO2 might regulate adipose tissue expansion during obesity. Another member in the R-spondin family, Rspo3 (ref. ^34^), which has also been detected in ingWAT (Extended Data Fig. 7e), was shown to be associated with reduced lower body fat, enlarged gluteal adipocytes and insulin resistance in humans and regulates adiposity in zebrafish, which is in line with our observations. However, these data have to be treated with caution as the findings from Rspo3 as well as our work on Rspo2 are based on changes in circulating levels, which could be due to other adipose tissue-independent mechanisms, even though we did not observe changes in liver function.

Overexpression of RSPO2, specifically in ingWAT, confirmed our finding that RSPO2 can inhibit adipocyte formation and cause adipocyte hypertrophy, with a trend toward development of insulin resistance. As RSPO2 is a secreted protein, expressed also in non-adipose tissue^35^, loss of RSPO2 in WAT might be compensated by circulating RSPO2.

Circulating RSPO2 correlated with insulin sensitivity and fat distribution in men but not in women. The detected sex differences may reflect the previous notion that adipocyte size and function are distinctly associated with parameters of metabolic health in women and men^36,37^. In this context, it has been suggested that SAT of premenopausal women has a greater capacity for adipose expansion via hyperplasia and hypertrophy; although larger, these gluteal–femoral adipocytes remain insulin sensitive^38^. Moreover, sex differences have been reported in adipogenesis and metabolism^39–41^.

Inherently a study such as this has many limitations. Besides the ones outlined above it should be noted that without a transgenic mouse model that can be used to deplete P3 cells or induce loss of RSPO2 specifically within adipose tissue, it will be difficult to determine to which degree ingWAT derived P3 cells or RSPO2 contribute to metabolic adaptations. Also in human studies, RSPO2 expression within adipose tissue and its relation to metabolic diseases will require a fractionation of adipose tissue in larger patient cohorts. Last, while our in vitro experiments demonstrate that RSPO2 inhibits adipogenesis of P1 cells through Lgr4, a model with specific loss of Lgr4 in P1 cells will be required to delineate the effects on metabolism.

In conclusion, here we delineate additional regulatory mechanisms that control adipose tissue plasticity governed by a cellular and molecular crosstalk of different cell types within the SVF of adipose tissue. We identify RSPO2 as a new functional effector of the P3 population that controls adipocyte formation by modulating maturation of P1 to P2 cells and might therefore play an important role in regulating adipose tissue plasticity.

## Methods

### Animals

C57BL/6 mice were obtained from Charles River Laboratories and C57BL/6J:ROSA^mT/mG^ mice (stock no. 007676), AdipoCre mice (stock no. 028020), NucRed mice (stock no. 026006) were obtained from JAX. Mice were kept on a 12-h/12-h light/dark cycle and 20–60% (25 °C) humidity in a pathogen-free animal facility of SLA ETH Zurich. The HFD used contained 60% (kal%) fat (diet no. 3436, Provimi Kliba SA). All animal experiments were approved by the cantonal veterinary office Zurich.

### Human cohort

Sixty individuals were selected from the Leipzig Obesity Biobank to define age-, BMI- and sex-matched groups of insulin-sensitive (*n* = 30) and insulin-resistant (*n* = 30) individuals with obesity. Definition of the metabolic healthy obese participants was based on the glucose infusion rate (GIR) during the last 30 min of the steady state in euglycemic-hyperinsulinemic clamps (insulin-sensitive, GIR > 70 μmol kg^−1^min^−1^; insulin-resistant, GIR < 60 μmol kg^−1^min^−1^)^42^. All individuals fulfilled the previously reported inclusion and exclusion criteria^43^. BMI was calculated as weight divided by squared height. Waist circumference was measured at the midpoint between the lower ribs and iliac crest. Percentage body fat was measured by bioimpedance analysis. Abdominal visceral and subcutaneous fat areas were calculated using computed tomography or magnetic resonance imaging scans at the level of L4–L5. Insulin sensitivity was assessed using the euglycemic-hyperinsulinemic clamp method^42^. The study was approved by the ethics committee of the University of Leipzig (approval nos. 159-12-21052012 and 017-12-23012012) and all participants gave written informed consent before taking part in the study.

### Re-processing scRNA-seq datasets

The datasets from Schwalie et.al.^9^ and Merrick et.al.^4^ were used. Briefly, we first used STARSolo (2.7.3a) to map reads and de-multiplex cell barcodes with parameters: ‘–winAnchorMultimapNmax 2,000–outFilterMultimapNmax 2,000–outSAMprimaryFlag AllBestScore–outSAMmultNmax 1–limitOutSJoneRead 10,000–limitOutSJcollapsed 3,000,000–outSAMattributes NH HI nM AS CR UR CB UB GX GN sS sQ sM–soloType CB_UMI_Simple–soloCBwhitelist 737K-april-2014_rc.txt–soloCBlen 14–soloUMIstart 15–soloUMIlen 8–soloBarcodeReadLength 0–soloStrand Forward–soloFeatures Gene’. The whitelist (737K-april-2014_rc.txt) of the cell barcode is available in CellRanger package. We then kept unique mapped reads (by NH:I:1 flag) and assigned reads to gene loci using featureCount. After parsing featureCount results, we obtained a raw gene count matrix. We used DropletUtils to differentiate cell barcodes mainly containing ambient RNAs with actual cells and generated a filtered count matrix.

We used the Seurat package (v.3.1.2) for downstream quality control, cell clustering and generating two-dimensional UMAP cell plots. We removed cells with <500 or >6,000 genes detected and with more than 30% of Unique molecular identifiers (UMIs) derived from mitochondrial RNAs. Raw UMI counts were normalized and log-transformed per 10,000 UMIs. When scaling the normalized count for calculating principal components (PCs), the number of UMIs per cell and the percentage of mitochondrial RNAs were regressed out. We selected top 30 PCs for graph-based clustering with resolution parameter 0.3 (snn), which has been implemented in the FindNeighbors and FindClusters functions of the Seurat package. We used the RunUMAP functions of Seurat package to generate UMAP with n.neighbors = 50. For data integration, we first pooled individual datasets from both studies and selected top 2,000 most-variable genes from each dataset for canonical correlation analysis (CCA). The top 40 dimensions in CCA were used for identifying anchors (FindIntegrationAnchors). With these anchors, datasets were integrated with IntegrateData function. We scaled the integrated data and computed top 50 PCs for cell clustering (with resolution 0.3) and UMAP plot.

For re-clustering of mouse ingWAT cells (Fig. 1a)^9^, we applied unsupervised clustering using sctransform^44^, based on the 6,500 most-variable genes. Cluster P5 was removed from subsequent analysis owing to its low gene expression (Extended Data Fig. 1h). The top ten markers for each cluster (logfc.threshold = 0.5) were plotted on the heat map (Extended Data Fig. 1i).

### Single-cell trajectory analysis

Cellranger v.3.0 (by 10X Genomics) was applied to map the spliced and unspliced transcripts, Velocyto was applied to reconstruct the RNA state trajectory^12^, scVelo^13^ was applied to model the cellular dynamics and Monocle 3 (ref. ^16^) was used to estimate the pseudotime trajectory.

### Gene overlapping analysis between human and mouse preadipocyte

The R package biomaRt v.2.48.2 was used to map the overlapping genes between human and mouse single-cell data based on positive cluster markers. Significance of overlaps was calculated based on hypergeometric distribution and simulations using the R packages stats v.4.1 and purrr v.0.3.4.

### snRNA-seq of adipose tissue

Nuclei were isolated from mouse ingWAT infected with either pAAV–CAG–GFP or pAAV–CAG–Rspo2, following a previously established protocol^19^. Around 10,000 nuclei were loaded to 10X Genomics Chromium and libraries were prepared with Single Cell 3′ (v3) RNA kit. Sequencing was performed with a Novaseq 6000 (Illumina). Raw reads were mapped to GENCODE Release M26 (GRCm39). Cellbender^45^ was applied to remove ambient RNA and empty droplets; scrublet^46^ was applied to remove doublets. Seurat was applied for unsupervised clustering and CCA^15^ was applied for integrative analysis.

### Mouse SVF isolation and culture

For SVF isolation, adipose tissues were minced with scissors and incubated in 1 mg ml^−1^ collagenase (C6885-1G, Sigma-Aldrich) in collagenase buffer (25 mM NaHCO_3_, 12 mM KH_2_PO_4_, 1.2 m MgSO_4_, 4.8 mM KCl, 120 mM NaCl, 1.4 mM CaCl_2_, 5 mM glucose, 2.5% BSA and 1% Pen/Strep, pH 7.4) for 50 min at 37 °C under agitation. An equal volume of DMEM (61965026, Gibco) (supplemented with 10% FBS and 1% Pen/Strep) was added and samples were centrifuged for 5 min at 300*g*. The SVF pellet was resuspended in 2 ml erythrocyte lysis buffer (154 mM NH_4_Cl, 10 mM KHCO_3_ and 0.1 mM EDTA, pH 7.4) and incubated for 4 min at room temperature. Samples were diluted with 10 ml DMEM and filtered through 40-µm cell strainers. After centrifuging for 5 min at 200*g*, the pellets were resuspended in FACS buffer (PBS with 3% FBS, 1 mM EDTA and 1% P/S). Cells were then centrifuged at 200*g* for 5 min and cell pellets were resuspended in FACS buffer for antibody staining or in culture medium for seeding. Lin^−^:Sca1^+^, CD142^−^, CD142^+^ and CD142^+^^+^ populations were FACS purified (gating strategies shown in Extended Data Fig. 2a) and cultured in either DMEM (supplement with 10% FBS and 1% Pen/Strep) or DMEM/F12 (supplement with 10% FBS and 1% Pen/Strep). At 48 h after cell confluence, adipogenesis was induced with the following cocktails: cells were exposed to A induction cocktail for 48 h, which contained 1 μM dexamethasone, 0.5 mM isobutylmethylxanthine and 1 μM insulin in DMEM, then changed to maintenance cocktail (1 μM insulin). B induction cocktail contained 1 μM dexamethasone, 0.5 mM isobutylmethylxanthine, 125 nM indomethacin, 1 nM T3 and 20 nM insulin in DMEM/F12. Some cells were exposed to B induction cocktail to induce adipogenesis and changed to maintenance cocktail contained 1 nM T3 and 20 nM insulin in DMEM/F12 two days after induction. C induction cocktail contained 20 nM insulin in DMEM/F12 medium and cells were exposed to C cocktail during induction and maintenance. Maintenance cocktail was refreshed every 48 h.

### Fluorescence-activated cell sorting

SVF was isolated from ingWAT and resuspended in FACS buffer to 0.5 × 10^7^ cells ml^−1^. Cells were incubated with purified anti-CD16/CD32 antibody (1:50 dilution, BioLegend, 101302) for 10 min on ice before immunostaining. The following fluorophore-conjugated antibodies were added to samples: anti-mouse CD31–PECy7 (1:600 dilution), anti-mouse CD45–PECy7 (1:600 dilution), anti-mouse TER119–PECy7 (1:600 dilution) (all BioLegend, 102418, 103114 and 116222, respectively), anti-mouse Sca1-Brilliant Violet 711 (1:600 dilution) (BioLegend, 108131), anti-mouse CD55–PE (1:700 dilution) (BD BIOSCIENCES, 558037), anti-mouse VAP1 (1:200 dilution) (Abcam, ab81673) and anti-mouse CD142 (1:700 dilution) (SinoBiological, 50413-R001). Anti-VAP1 or anti-CD142 antibody were conjugated with fluorescein (FITC) (Expedeon, SKU:707-0005) or allophycocyanin (Expedeon, SKU:705-0030) following the manufacturers recommendation. SVF was stained for 20 min on ice, protected from light, followed by two washes with 10 ml FACS buffer. After the final wash, the SVF pellet was resuspended in FACS buffer to 1 × 10^6^ cells ml^−1^. SYTOX blue (1:2,000 dilution; Thermo Fisher, S34857) was used for dead cell staining. Compensation setup was performed with single stains of AbC Total Antibody Compensation Bead (Thermo Fisher, A10497).

### RNA extraction

RNA extraction of sorted cells: cells were collected in RLT+ lysis buffer (QIAGEN, 1053393) and flash-frozen on dry ice. Cell lysates were homogenized with QIAshredders before RNA isolation using the RNeasy Plus Micro kit (QIAGEN, 74034). Reverse transcription was performed using the QuantiTect whole transcriptome kit (QIAGEN, 207045) following the manufacturer’s recommendations for a standard-yield reaction (2 h of amplification time).

### Bulk mRNA-seq for eP1 and eP2 cells

All samples passed RNA integrity number >9 determined by testation. The Illumina Truseq kit was used to generate libraries. Single-read sequencing was performed at the Functional Genomics Center Zurich on an Illumina HiSeq 4000 platform. Raw reads were trimmed using Trim Galore (v.0.4.4) and mapped to the mouse GRCm38 genome assemblies using STAR. Transcripts were defined using the Ensembl annotations over protein-coding mRNAs. Differential expression analysis was performed using DESeq2 (ref. ^47^) and EdgeR^48^. Differentiated regulated genes were used for biological pathway prediction based on Enrichr^24,25^.

### siRNA-mediated knockdown experiments

A pool of 3–4 individual siRNA probes were used to knockdown targets at a final concentration of 100 nM. siRNA probes were dissolved in 1.5% Lipofectamine RNAiMAX (Invitrogen, 13778150) in Opti-MEM I reduced serum medium (Invitrogen, 31985062). For 96-well plates, 100 μl primary cells (25 × 10^4^ ml^−1^) were reverse-transfected with 20 μl 100 nM of corresponding siRNA. At 48 h after transfection, cells were changed to culture medium without siRNA. For the Transwell co-culture experiment, eP3 cells were cultured in inserts with corresponding siRNA for 48 h with a blank receiver plate. At 48 h after transfection, the inserts were washed with warm PBS twice and co-cultured with Lin^−^Sca1^+^CD142^−^ cells growing on receiver plates until the end of the experiment. For the β-catenin experiment, 48 h after transfection, eP1 cells were changed to culture medium supplemented with 0.5 μg ml^−1^ recombinant RSPO2 for 24 h.

### In vivo differentiation of SVF

A total of 200,000 sorted cells (eP1 or eP2 cells from male mice) were resuspended in 200 µl of Matrigel (Corning, 356234) supplement with AAV and 5 µg rec.RSPO2 (heat inactivation for control group), then injected subcutaneously in the abdomen of the same 4-week-old mouse. After 4 weeks of HFD, Matrigel plugs were excised and fixed in 4% paraformaldehyde overnight, dehydrated and embedded in paraffin. Sections of 5 μm were stained with H&E and examined. From each plug, pictures of at least three full sections were taken and adipocyte numbers as well as the number of nuclei were determined automatically with Cell Profiler software.

### AAV production

Adenoviral particles carrying overexpression constructs under control of the CAG promoter to express either GFP or RSPO2. The coding transcript sequence for Rspo2 was obtained from OriGene (MR216699) and insert into vector pAAV–CAG–GFP (Addgene, 37825) to replace GFP. The direction of insertion and nucleotide sequences of the insert was verified by sequencing analysis (Microsynth). Virus was produced in HEK 293A cells (Invitrogen) and purified with AAVanced Concentration Reagent (System Biosciences, AAV100A-1) following the manufacture’s protocol.

### Adipocyte number quantification by qPCR

AdipoCre-NucRed mice were used for quantification of adipocyte numbers in adipose tissue. Adiponectin^+^ adipocytes express tdTomato as a result of Cre-recombination of tdTomato allele (Extended Data Fig. 4c)^27^. Briefly, a known quantity of plasmid carrying ApoB sequence and recombined tdTomato sequence was used to build standard curves in a qPCR assay (Extended Data Fig. 4d). To quantify adipocytes (Adipoq^+^), genomic DNA was extracted from ingWAT or visWAT^19^ and the number of recombined tdTomato alleles and ApoB alleles (total cell number) were quantified by qPCR.

### Mouse surgery

For cell transplantation, tdTomato^+^ eP1 cells were isolated from ingWAT of ROSA^mT/mG^ mice by FACS. eP2 cells from ROSA^mT/mG^ mice were collected in RLT+ lysis buffer for RNA extraction. The eP1 cell pellets were resuspended in Matrigel for injection. The 3–4-week-old mice were anesthetized with isoflurane flow and abdominal hair was removed. A small opposing Y-shaped cutaneous incision was made following the abdominal midline to expose inguinal fat pads. A total of 100,000 cells in 20 µl of Matrigel was injected along the edge of the ingWAT in several depots. Ten days after injection, injected ingWAT was dissected and cells were isolated as described above. Cells were stained with VAP1–FITC antibody and analyzed by Sony SH800 cell sorter. tdTomato^+^VAP1^−^ cells and tdTomato^+^VAP1^+^ cells were collected in RLT+ lysis buffer for RNA extraction. For AAV injections, 40–50 µl of AAV were distributed in each side of ingWAT. Three weeks after surgery, mice were fed on HFD for another 3 weeks. IngWAT was dissected for FACS analysis and protein extraction. To investigate whether RSPO2 inhibits eP1 to eP2, mice were first infected with pAAV–CAG–Rspo2 or pAAV–CAG–GFP in ingWAT. Three weeks after AAV infection, a suspension of 100,000 tdTomato^+^ eP1 cells (in 20 µl of Matrigel, with or without 5 µg rec.RSPO2) was injected into ingWAT. Ten days after surgery, the injected ingWAT was dissected and cells were isolated for FACS. Cells were stained with VAP1–allophycocyanin antibody and analyzed by Sony SH800 cell sorter.

### Protein extraction and western blot

Protein samples were extracted from adipose tissue and in vitro cultured cells with RIPA buffer (50 mM Tris-HCl, pH 7.5), 150 mM NaCl, 1 mM EDTA, 1% Triton X-100, 0.1% SDS and 10% glycerol) supplemented with protease inhibitor cocktail (11697498001, Sigma-Aldrich) and Halt Phosphatase Inhibitor (78420, Thermo Fisher). Protein levels were quantified using the DC Protein Assay (Bio-Rad). For immunoblotting, protein samples were separated by SDS–PAGE on 12% polyacrylamide gels and transferred onto a nitrocellulose membrane. Membranes were probed using the indicated antibodies (anti-RSPO2, 1:1,000 dilution, Biorbyt orb185986; anti-HSP90, 1:1,000 dilution, Cell Signaling 4877S; β-catenin, 1:1,000 dilution, Abcam ab223075; and β-actin, 1:1,000 dilution, Cell Signaling 4967). Chemiluminescent signals of the HRP-conjugated secondary antibodies (1:5,000 dilution, Calbiochem) were detected by a LAS 4000 mini-Image Quant system (GE Healthcare). Band intensity was quantified using ImageJ.

### Adipogenesis quantification

Differentiated cells were fixed with 4% formaldehyde before staining with LD540 (2 μM) for lipid droplets, Hoechst 33342 (4 μM) for nuclei and Syto60 (5 μM) for cytosolic staining (all Invitrogen). Twenty pictures per well were taken with an automated microscope imaging system (Operetta, PerkinElmer). Pictures were analyzed using Harmony software or Cell Profiler software.

H&E staining images of Matrigel plugs were analyzed by Cell Profiler software v.3.1. The pipeline was modified from online pipeline file ‘AdipocyteSize.cp’ from Cell Profiler Forum (https://forum.image.sc/t/adipocyte-h-e-cell-profiler-pipeline/12490). Briefly, by adjusting diameter of objects the pipeline could identify cell membrane and export ‘Count_Membrane’ which equals cell numbers. By adjusting diameter of adipocytes, the pipeline could identify all the adipocytes and export count number.

### β-catenin staining

SVF cells was isolated from ingWAT as described above. A total of 20,000 cells were seeded in a 96-well plate (Greiner Bio-One, 655090) per well. At 24 h after SVF cells attachment, 0.5 μg ml^−1^ rec.RSPO2 was added in medium for 0–24 h. Cells were washed with PBS before fixed with 4% formaldehyde. Cells were incubated with 5% donkey serum supplement with 0.1% Triton in room temperature for 1 h, followed with anti-β-catenin antibody (1:200 dilution, Abcam, ab223075) incubation overnight at 4 °C. Cells were washed three times with PBS and Alexa Fluor Plus 488 donkey anti-rabbit IgG secondary antibody (1:500 dilution, Invitrogen, A32790) for 1 h at room temperature protected from light. Nuclei were stained in parallel using Hoechst 33342 (1:10,000 dilution, Cell Signaling, 4082). Cells were washed three times with PBS, before imaging.

### Adipocyte size quantification

IngWAT paraffin blocks were sectioned at 6 µm and stained with H&E. IngWAT sections were examined by light microscopy using AxioPhot microscope equipped with AxioCam MR (Zeiss). Adipocyte size was determined using the Fiji Adiposoft plugin^49^. At least six fields of view were analyzed for each sample. Adipocyte size frequency distribution was calculated using GraphPad Prism 8.

### Insulin tolerance test

Mice were fasted for 8 h and then injected i.p. with human insulin (Actrapid Penfill, Novo Nordisk) at 0.25 U kg^−1^. Blood glucose levels were measured by a blood glucometer (Accu-Chek Aviva, Roche) before and at 15, 30, 60 and 120 min after insulin injection. For data analysis, glucose levels versus time after injection were plotted using GraphPad Prism 8. Area under curve was calculated as a surrogate of insulin sensitivity.

### Pyruvate tolerance test

Mice were fasted overnight (>12 h) and then injected with pyruvate (1 g kg^−1^ body weight; i.p.; Sigma-Aldrich, P5280). Blood glucose was measured before and 15, 30, 60 and 120 min after pyruvate injection. For data analysis, glucose levels versus time after injection were plotted using GraphPad Prism 8. AUC was calculated to estimate hepatic gluconeogenesis.

### Hepatic triglyceride production rate

After 6 h fasted mice were injected with Triton (Sigma) in saline (400 mg kg^−1^ i.v.). Immediately before injection and at 1, 2, 3 and 4 h following injection, blood samples were collected in heparin capillary tubes and TG concentrations in plasma were determined. The TG production rate was calculated from the difference in plasma TG levels over a given interval following Triton injection and was expressed as mg TG dl^−1^ plasma h^−1^.

### In vivo overexpression of RSPO2 by tail-vein delivery of pAAV–CAG–RSPO2

pAAV–CAG–GFP and pAAV–CAG–RSPO2 (5 × 10^9^ vg kg^−1^) were administered into the tail vein of mice at 8 weeks of age. Mice were fed with HFD from 4 weeks of age until they were killed. ITT was performed in mice at week 13 after 8 h fasting.

### Triglyceride measurement

Total lipids were extracted from up to 50 mg tissue using chloroform:methanol (2:1) mixture and normalized to tissue weight. TG in plasma and liver was measured by Cobas TRIGB kit (Roche/Hitachi) following the manufacturer’s instructions.

### RSPO2 ELISA

Plasma RSPO2 levels were analyzed by using the Mouse R-spondin-2 ELISA kit (Cusabio Technology, CSB-EL020551MO) and Human R-spondin-2 ELISA kit (Cusabio Technology, CSB-EL020551HU).

### Oligonucleotides

Primer and siRNA oligonucleotide sequence are attached in Extended Data Table 9.

### Statistical analysis

Statistical methods were not used to predetermine sample size. The experiments were not randomized and investigators were not blinded in experiments. Results are given as mean ± s.d. or s.e.m. described in detail in legend. Statistical analyses were performed using two-tailed Student’s *t*-test or ANOVA.

### Reporting Summary

Further information on research design is available in the Nature Research Reporting Summary linked to this article.

## Supplementary information

Reporting Summary

Supplementary Tables Extended Data Table 1: Overlapping genes between mouse scRNA-seq clusters and human snRNA-seq clusters. Extended Data Table 2. Cell culture medium used in Fig. 2 and Extend Data Fig. 2. Extended Data Table 3: Marker genes of each cluster in murine Lin^−^ cells. Extended Data Table 4: Marker genes for human pre-adipocytes clusters from human deep neck adipose tissue. Extended Data Table 5: Pearson coefficient correlation of male participants between RSPO2 (ng ml^−1^) in plasma and following parameters. Extended Data Table 6: Pearson coefficient correlation of female participants between RSPO2 (ng ml^−1^) in plasma and following parameters. Extended Data Table 7: Marker genes for each cluster identified in 10X Genomics snRNA-seq of ingWAT CAG–GFP and CAG–RSPO2. Extended Data Table 8: Marker genes for pre-adipocyte clusters identified in 10X Genomics snRNA-seq of ingWAT CAG–GFP and CAG–RSPO2. Extended Data Table 9: Sequence of oligonucleotides.

## Data Availability

RNA-seq data has been deposited in ArrayExpress (www.ebi.ac.uk/arrayexpress), E-MTAB-6677 for scRNA-seq of ingWAT Lin^−^ cells, E-MTAB-5787 for bulk RNA-seq of CD142^++^ cells and Lin^−^CD142^−^ cells, E-MTAB-9827 for bulk RNA-seq of eP1 and eP2 cells, E-MTAB-11104 for single-nucleus RNA-seq of mouse ingWAT with Rspo2 or GFP AAV injection. The datasets can be explored interactively at https://batnetwork.org/. For further bioinformatic information, please contact wenfei-sun@stanford.edu. Source data are provided with this paper.

## Source data

Source Data Fig. 2 Statistical source data.
Source Data Fig. 3 Statistical source data.
Source Data Fig. 4 Statistical source data.
Source Data Fig. 4 Uncropped western blots.
Source Data Fig. 5 Statistical source data.
Source Data Fig. 5 Uncropped western blots.
Source Data Fig. 6 Statistical source data.
Source Data Fig. 6 Uncropped western blots.
Source Data Extended Data Fig. 1 Statistical source data.
Source Data Extended Data Fig. 2 Statistical source data.
Source Data Extended Data Fig. 3 Statistical source data.
Source Data Extended Data Fig. 3 Uncropped western blots.
Source Data Extended Data Fig. 4 Statistical source data.
Source Data Extended Data Fig. 4 Uncropped western blots.
Source Data Extended Data Fig. 5 Statistical source data.
Source Data Extended Data Fig. 5 Uncropped western blots.
Source Data Extended Data Fig. 7 Statistical source data.
Source Data Extended Data Fig. 7 Uncropped western blots.

## Notes

**Editor recognition statement** Primary handling editor: Isabella Samuelson. **Reviewer recognition statement** *Nature Metabolism* thanks Qiong Wang and the other, anonymous, reviewers for their contribution to the peer review of this work.
