# Supplementary material for: Identification of a regulatory pathway inhibiting adipogenesis via RSPO2
Source: Nat Metab. 2022 Jan 13;4(1):90–105. doi: 10.1038/s42255-021-00509-1 (PMC8803606; doi:10.1038/s42255-021-00509-1)
Supplement: Supplementary file 1 — Reporting Summary [file 42255_2021_509_MOESM1_ESM.pdf]

nature research

Corresponding author(s): Christian Wolfrum

Last updated by author(s): Oct 25, 2021

Reporting Summary

Nature Research wishes to improve the reproducibility of the work that we publish. This form provides structure for consistency and transparency in reporting. For further information on Nature Research policies, see our Editorial Policies and the Editorial Policy Checklist.

Statistics

For all statistical analyses, confirm that the following items are present in the figure legend, table legend, main text, or Methods section.

n/a	Confirmed	
☐	☒	The exact sample size (*n*) for each experimental group/condition, given as a discrete number and unit of measurement
☐	☒	A statement on whether measurements were taken from distinct samples or whether the same sample was measured repeatedly
☐	☒	The statistical test(s) used AND whether they are one- or two-sided<br>*Only common tests should be described solely by name; describe more complex techniques in the Methods section.*
☐	☒	A description of all covariates tested
☐	☒	A description of any assumptions or corrections, such as tests of normality and adjustment for multiple comparisons
☐	☒	A full description of the statistical parameters including central tendency (e.g. means) or other basic estimates (e.g. regression coefficient) AND variation (e.g. standard deviation) or associated estimates of uncertainty (e.g. confidence intervals)
☐	☒	For null hypothesis testing, the test statistic (e.g. *F*, *t*, *r*) with confidence intervals, effect sizes, degrees of freedom and *P* value noted<br>*Give P values as exact values whenever suitable.*
☒	☐	For Bayesian analysis, information on the choice of priors and Markov chain Monte Carlo settings
☒	☐	For hierarchical and complex designs, identification of the appropriate level for tests and full reporting of outcomes
☐	☒	Estimates of effect sizes (e.g. Cohen's *d*, Pearson's *r*), indicating how they were calculated

Our web collection on statistics for biologists contains articles on many of the points above.

Software and code

Policy information about availability of computer code

Data collection	BD FACSAria III software and Sony SH800 cell sorter software was used to collect from flow cytometry; Zen 2012 software were used to collect HE staining data; Operetta software 3.0 was used for in vitro adipogenesis imaging; LAS 4000 mini Image Quant system software were used to collect western blot images.
Data analysis	Flow cytomertric analysis were performed with FlowJo analysis software (FlowJo10); Cell Profiler software (CellProfiler 3) was used for quantify adipogenesis in vivo ; Harmony 3.5.2 was used for quantify adipogenesis in vitro; statistic analysis were performed with the GraphPad Prism 8.0 software. We used Seurat package (V 3.1.2) for downstream QC, cell clustering and generating 2D UMAP cell plots. Cellranger V3.0 (by 10x genomics) was applied to map the spliced and unspliced transcripts, Velocyto was applied to reconstruct the RNA states trajectory, scVelo was applied to model the cellular dynamics. Monocle 3 was used to estimate the pseudotime trajectory.

For manuscripts utilizing custom algorithms or software that are central to the research but not yet described in published literature, software must be made available to editors and reviewers. We strongly encourage code deposition in a community repository (e.g. GitHub). See the Nature Research guidelines for submitting code & software for further information.

Data

Policy information about availability of data

All manuscripts must include a data availability statement. This statement should provide the following information, where applicable:

- Accession codes, unique identifiers, or web links for publicly available datasets
- A list of figures that have associated raw data
- A description of any restrictions on data availability

The authors declare that the data supporting the findings of this study are available within the paper[and its supplementary information files].
RNA sequencing data has been deposited in ArrayExpress (www.ebi.ac.uk/arrayexpress). Accession codes E-MTAB-6677 for scRNAseq of ingWAT Lin- cells.

Accession codes E-MTAB-5787 for bulk RNA seq of CD142++ cells and Lin-CD142- cells. Accession codes for Bulk RNA seq of eP1 and eP2 cells is E-MTAB-9827. Accession codes single nucleus RNAseq of mouse ingWAT with Rspo2 or GFP AAV injection is E-MTAB-11104. Please address correspondence and requests for materials to C.W. and requests for bioinformatic information to W.S. (Wenfei-sun@ethz.ch or Wenfei-sun@stanford.edu). The datasets can be explored interactively at https://batnetwork.org/. Source data are provided with this paper.

Field-specific reporting

Please select the one below that is the best fit for your research. If you are not sure, read the appropriate sections before making your selection.

☒ Life sciences ☐ Behavioural & social sciences ☐ Ecological, evolutionary & environmental sciences

For a reference copy of the document with all sections, see nature.com/documents/nr-reporting-summary-flat.pdf

Life sciences study design

All studies must disclose on these points even when the disclosure is negative.

Sample size	No statistical method was used to predetermine sample size; the sample size are determined based on previous working experience.
Data exclusions	No data was excluded from the analysis
Replication	All attempts at replication were successful, and the details was provided in corresponding figure legends.
Randomization	All studies were performed on animals or tissues collected from animals. Animals of each sex and strain were randomized into groups of equivalent weight prior to the beginning of the in vivo studies
Blinding	Investigators were blinded to group allocation during data collection and analysis.

Reporting for specific materials, systems and methods

We require information from authors about some types of materials, experimental systems and methods used in many studies. Here, indicate whether each material, system or method listed is relevant to your study. If you are not sure if a list item applies to your research, read the appropriate section before selecting a response.

Materials & experimental systems

n/a	Involved in the study	
☐	☒	Antibodies
☒	☐	Eukaryotic cell lines
☒	☐	Palaeontology and archaeology
☐	☒	Animals and other organisms
☐	☒	Human research participants
☒	☐	Clinical data
☒	☐	Dual use research of concern

Methods

n/a	Involved in the study	
☒	☐	ChIP-seq
☐	☒	Flow cytometry
☒	☐	MRI-based neuroimaging

Antibodies

Antibodies used	anti-CD16/CD32 (Biolegend, clone 93, Cat# 101302, Lot# B282595), anti-mouse CD31-PECy7 (BioLegend, clone 390, Cat #102418, Lot# B264590), anti-mouse CD45-PECy7 (BioLegend, clone 30-F11, Cat #103114, Lot# B246760), anti-mouse TER119-PECy7 (BioLegend, clone TER-119, Cat #116222, Lot# B251241), anti-mouse Sca1-Brilliant Violet 711 (BioLegend, clone D7, Cat #108131, Lot # B241586), anti-mouse CD55-PE (BD BIOSCIENCES, clone RIKO-5, Cat #558037, Lot# 9121503), anti-mouse VAP1 (Abcam, clone 7-88, Cat #ab81673, Lot# GR3232949-2), anti-mouse CD142 (SinoBiological, clone 001, Cat #50413-RP01, Lot# HA10OC2402-B), anti beta-catenin (Abcam, clone IGX4794R-3, Cat #ab223075, Lot# GR3178277-1), anti RSPO2 (Biorbyt, Cat# orb185986, Lot# BR5703), Alexa Fluor 488 donkey anti-rabbit IgG secondary antibody (Invitrogen, Cat # A32790, Lot #UL293143); anti-HSP90, 1:1000, Cell signaling #4877S; HRP-conjugated secondary antibodies (1:5000, Calbiochem).
Validation	These are commercial antibodies with validations available in the manufacturers' website.

Animals and other organisms

Policy information about studies involving animals; ARRIVE guidelines recommended for reporting animal research

Laboratory animals	C57Bl/6 mice were obtained from Charles River Laboratories, and C57BL/6J:ROSAmT/mG mice (stock: 007676), AdipoCre mice (stock: 028020), NucRed mice (stock: 026006) were obtained from the Jackson Laboratory. Mice were kept on a 12-h/12-h light/dark cycle and 20-60%(25°C) humidity a pathogen-free animal facility of SLA ETH Zurich. The HFD used contained 60% (kal%) fat (diet No. 3436,

	Provimi Kliba SA). The age and sex of the animals were described in method part in detail.
Wild animals	This study did not involve wild animals.
Field-collected samples	This study did not involve samples collected from field.
Ethics oversight	All animal experiments were approved by the Animal Ethics Committee of Zurich.

Note that full information on the approval of the study protocol must also be provided in the manuscript.

Clinical data

Policy information about clinical studies

All manuscripts should comply with the ICMJE guidelines for publication of clinical research and a completed CONSORT checklist must be included with all submissions.

Clinical trial registration	For the purpose of this study, we selected 60 individuals from the Leipzig Obesity Biobank to define age-, BMI-, and sex-matched groups of insulin sensitive (n=30) and insulin resistant (n=30) patients with obesity. The study was approved by Ethics committee of the University of Leipzig (approval numbers: 159-12-21052012 and 017-12-23012012).
Study protocol	The study was approved by the ethics committee of the University of Leipzig, and detailed protocol could be found in Leipzig Obesity Biobank and related sutdy.
Data collection	Definition of the MHO subgroups was based on the glucose infusion rate (GIR) during the last 30min of the steady state in euglycemic-hyperinsulinemic clamps (IS: GIR > 70µmol/kg/min; IR: GIR < 60µmol/kg/min). All individuals fulfilled the previously reported inclusion and exclusion criteria.BMI was calculated as weight divided by squared height. Waist circumference was measured at the midpoint between the lower ribs and iliac crest. Percentage body fat was measured by bioimpedance analysis. Abdominal visceral and subcutaneous fat areas were calculated using computed tomography or MRI scans at the level of L4–L5. Insulin sensitivity was assessed using the euglycemic-hyperinsulinemic clamp method as described in method
Outcomes	The correlation of RSPO2 level in plasma with different clinical measurements listed in paper was calculated by Pearson's Correlation Tests in Prism 8.0.

Flow Cytometry

Plots

Confirm that:

☒ The axis labels state the marker and fluorochrome used (e.g. CD4-FITC).

☒ The axis scales are clearly visible. Include numbers along axes only for bottom left plot of group (a 'group' is an analysis of identical markers).

☒ All plots are contour plots with outliers or pseudocolor plots.

☒ A numerical value for number of cells or percentage (with statistics) is provided.

Methodology

Sample preparation	For SVF isolation, dissected adipose tissues were minced with scissors and incubated in 1 mg/ml collagenase (#C6885-1G, Sigma-Aldrich) in collagenase buffer (25mM NaHCO3, 12mM KH2PO4, 1.2m MgSO4, 4.8mM KCl, 120mM NaCl, 1.4mM CaCl2, 5mM Glucose, 2.5% BSA,1% Pen/Strep, pH=7.4) for 50 min at 37°C under agitation. Equal volume of culture media (high glucose DMEM medium (#61965026, Gibco) supplement with 10%FBS and 1% Pen/Strep) was added and samples were centrifuged for 5 min at 300 g. The SVF pellet was resuspended in 2 ml erythrocyte lysis buffer (154 mM NH4Cl, 10 mM KHCO3, 0.1 mM EDTA, pH 7.4) and incubated for 4 min at room temperature. Samples were diluted with 10 ml culture media and filtered through 40 µm cell strainers. After 5 min of centrifugation at 200 g, the supernatant was removed and the pellets were resuspended in FACS buffer (PBS with 3% FBS, 1mM EDTA, 1% P/S). Centrifuge cells at 200g for another 5 min, then remove supernatant and resuspend cell pellets in FACS buffer for antibody staining.
Instrument	BD FACS Aria III, and Sony SH800 cell sorter
Software	FlowJo
Cell population abundance	After sorting, a aliquot of collected cells were run through the same sorter, and the same gating strategy was applied to check the purity. A general purity of higher than 95% were achieved for all the sorted population.
Gating strategy	The gating strategies were described in Fig. 2a, Fig. 5b, extended data Fig. 2a. FSC/SSC gating was use to display all the events detected. FSCA/FSCH was used to exclude doublets, and Sytox Blue staining was used to exclude dead cells.

☒ Tick this box to confirm that a figure exemplifying the gating strategy is provided in the Supplementary Information.
