# Supplementary material for: Identification of a regulatory pathway inhibiting adipogenesis via RSPO2
Source: Nat Metab. 2022 Jan 13;4(1):90–105. doi: 10.1038/s42255-021-00509-1 (PMC8803606; doi:10.1038/s42255-021-00509-1)
Supplement: Supplementary file 2 — Extended Data Table 1: Overlapping genes between mouse scRNA-seq clusters and human snRNA-seq clusters. Extended Data Table 2. Cell culture medium used in Fig. 2 and Extend Data Fig. 2. Extended Data Table 3: Marker genes of each cluster in murine Lin− cells. Extended Data Table 4: Marker genes for human pre-adipocytes clusters from human deep neck adipose tissue. Extended Data Table 5: Pearson coefficient correlation of male participants between RSPO2 (ng ml−1) in plasma and following parameters. Extended Data Table 6: Pearson coefficient correlation of female participants between RSPO2 (ng ml−1) in plasma and following parameters. Extended Data Table 7: Marker genes for each cluster identified in 10X Genomics snRNA-seq of ingWAT CAG–GFP and CAG–RSPO2. Extended Data Table 8: Marker genes for pre-adipocyte clusters identified in 10X Genomics snRNA-seq of ingWAT CAG–GFP and CAG–RSPO2. Extended Data Table 9: Sequence of oligonucleotides. [file 42255_2021_509_MOESM2_ESM.pdf]

Extended Data Table 1: Overlapping of genes between mouse scRNAseq clusters and human snRNAseq clusters

**Human cluster 1 (H-1)**			
Mouse cluster	numOverlappingGenes	% overlaps (human)	% Overlaps (mouse)
P1-1	3	0.949	3.488
P1-2	6	1.899	7.692
P1-3	3	0.949	2.632
P2-1	14	4.430	14.433
P2-2	15	**4.747**	13.274
P3	8	2.532	16.667

**Human cluster 2 (H-2)**			
P1-1	21	**5.185**	24.419
P1-2	18	4.444	23.077
P1-3	3	0.741	2.632
P2-1	3	0.741	3.093
P2-2	6	1.481	5.310
P3	0	0.000	0.000

**Human cluster 3 (H-3)**			
P1-1	20	2.946	23.256
P1-2	18	2.651	23.077
P1-3	13	1.915	11.404
P2-1	21	**3.093**	21.649
P2-2	18	2.651	15.929
P3	9	1.325	18.750

Extended data table 2

Cell culture medium used in Figure 2 and Extend data figure 2

	**Induction** (day 0 - 2)	**Maintenance** (from day 2)
**A** cocktail	DMEM<br>1μM dexamethasone<br>0.5mM isobutylmethylxanthine<br>1μM insulin	DMEM<br>1 μM insulin
**B** cocktail	DMEM/F12<br>1μM dexamethasone<br>0.5mM isobutylmethylxanthine<br>125nM Indomethacin<br>1nM T3<br>20nM insulin	DMEM/F12<br>1 nM T3<br>20nM insulin
**C** cocktail	DMEM<br>1μM insulin	DMEM<br>1μM insulin

Extended data Table 3

Marker genes of each cluster in murine Lin- cells

gene	p_val	avg_log2FC	pct.1	pct.2	p_val_adj	cluster
Isg15	9.2E-133	2.385	0.935	0.428	1.3E-128	P1-3
Tgm2	9.9E-141	1.969	0.619	0.099	1.4E-136	P1-3
Plac8	2.5E-107	1.861	0.997	0.931	3.6E-103	P1-3
Ccn5	5.2E-85	1.783	0.66	0.189	7.5E-81	P1-3
Ifit3	7.8E-105	1.762	0.773	0.243	1.1E-100	P1-3
Thbs2	2.8E-63	1.678	0.897	0.664	4.0E-59	P1-3
Bst2	4.2E-107	1.588	0.993	0.885	6.1E-103	P1-3
Pi16	6.7E-134	1.516	0.997	0.87	9.7E-130	P1-1
Il1r2	9.8E-96	1.510	0.776	0.455	1.4E-91	P1-1
Zbp1	2.8E-117	1.476	0.9	0.341	4.0E-113	P1-3
Sbsn	2.1E-83	1.464	0.733	0.383	3.1E-79	P1-1
Gng12	1.7E-98	1.439	0.979	0.783	2.4E-94	P1-3
Gas6	3.3E-21	1.410	0.55	0.307	4.8E-17	P1-3
Smpd3	2.2E-83	1.393	0.905	0.594	3.2E-79	P1-2
Ifit1	8.8E-97	1.386	0.732	0.202	1.3E-92	P1-3
Gpx3	1.3E-63	1.383	0.993	0.905	1.8E-59	P1-3
Tmem158	5.3E-53	1.382	0.928	0.701	7.7E-49	P1-3
Sfrp2	6.0E-106	1.356	0.986	0.772	8.6E-102	P1-1
Akr1c14	1.3E-89	1.349	0.773	0.261	1.8E-85	P1-3
Daglb	2.0E-100	1.342	0.628	0.25	2.9E-96	P1-2
Irf7	3.1E-83	1.336	0.928	0.519	4.5E-79	P1-3
Pgr	4.3E-231	1.322	0.612	0.037	6.2E-227	P1-3
Akr1c18	7.6E-88	1.304	0.736	0.378	1.1E-83	P1-2
Ifi211	2.4E-99	1.291	0.993	0.806	3.5E-95	P1-3
Ifi205	2.7E-105	1.277	1	0.962	3.9E-101	P1-3
Akr1c18	2.5E-55	1.270	0.814	0.422	3.7E-51	P1-3
Procr	3.1E-135	1.263	0.94	0.631	4.6E-131	P1-1
Ifi27l2a	3.6E-97	1.255	1	0.963	5.2E-93	P1-3
Ifitm3	3.7E-122	1.236	1	0.999	5.3E-118	P1-3
Pcsk6	7.8E-170	1.216	0.81	0.273	1.1E-165	P1-1
Ly6a	1.4E-102	1.199	1	0.97	2.0E-98	P1-3
Efhd1	6.0E-148	1.184	0.876	0.407	8.7E-144	P1-1
Ace	1.5E-167	1.180	0.973	0.69	2.2E-163	P1-1
Spon1	2.4E-77	1.178	0.869	0.368	3.4E-73	P1-3
Smpd3	1.5E-147	1.175	0.989	0.579	2.2E-143	P1-1
Ckb	4.3E-125	1.162	0.852	0.481	6.3E-121	P1-1
Il4ra	2.1E-99	1.157	0.907	0.385	3.0E-95	P1-3
Plod2	7.4E-89	1.149	0.876	0.386	1.1E-84	P1-3
Anxa3	1.4E-75	1.142	1	0.82	2.0E-71	P1-3
Trim30a	1.7E-88	1.127	0.828	0.327	2.5E-84	P1-3
Sema3c	4.4E-117	1.100	0.919	0.534	6.4E-113	P1-2
Lurap1l	2.1E-139	1.058	0.758	0.264	3.0E-135	P1-1
Igfbp6	2.5E-77	1.057	1	0.989	3.6E-73	P1-3
Oasl2	5.5E-90	1.052	0.808	0.287	7.9E-86	P1-3
St3gal1	1.7E-76	1.049	0.935	0.611	2.4E-72	P1-3
Ifi204	1.0E-88	1.042	1	0.888	1.5E-84	P1-3
Ccn1	1.7E-36	1.025	0.918	0.787	2.5E-32	P1-3

Cd164	2.1E-80	1.019	0.969	0.864	3.1E-76	P1-3
Ngfr	1.1E-134	1.016	0.574	0.075	1.6E-130	P1-3
Pcolce2	1.7E-143	1.012	1	0.901	2.5E-139	P1-1
Serpinb6a	2.8E-90	1.001	1	0.991	4.1E-86	P1-3
Sfrp4	4.7E-40	0.998	0.948	0.684	6.8E-36	P1-3
Irf7	5.5E-53	0.990	0.747	0.503	7.9E-49	P1-2
Cpz	1.3E-95	0.978	0.731	0.349	1.9E-91	P1-1
Cadm3	3.1E-132	0.976	0.853	0.391	4.5E-128	P1-1
Pi16	1.9E-94	0.975	0.997	0.867	2.8E-90	P1-2
Ly6e	4.4E-75	0.974	1	0.96	6.4E-71	P1-3
Cd320	1.2E-75	0.973	0.708	0.258	1.8E-71	P1-3
Greb1	2.0E-175	0.968	0.598	0.058	2.9E-171	P1-3
Gan	8.5E-27	0.967	0.564	0.308	1.2E-22	P1-3
H2-T23	4.1E-62	0.960	0.935	0.579	5.9E-58	P1-3
Mndal	2.5E-58	0.959	0.804	0.407	3.7E-54	P1-3
Igfbp4	1.6E-134	0.954	0.998	0.916	2.4E-130	P1-1
Anxa3	2.1E-98	0.947	1	0.794	3.1E-94	P1-1
Plac8	6.9E-86	0.936	0.996	0.92	1.0E-81	P1-2
Serpine2	1.1E-123	0.936	0.67	0.208	1.7E-119	P1-1
Dbp	4.3E-118	0.933	0.925	0.574	6.2E-114	P1-2
Znhit6	1.1E-86	0.926	0.619	0.16	1.6E-82	P1-3
Ecm1	5.3E-109	0.924	0.987	0.877	7.7E-105	P1-1
Scara5	2.4E-120	0.918	0.995	0.746	3.5E-116	P1-1
Sfrp4	1.1E-88	0.915	0.947	0.639	1.6E-84	P1-2
Stmn4	6.5E-151	0.913	0.514	0.078	9.4E-147	P1-1
Lrrn4cl	6.9E-107	0.911	0.963	0.654	1.0E-102	P1-2
Igfbp5	3.7E-52	0.911	0.931	0.753	5.3E-48	P1-1
Uqcc2	6.7E-132	0.909	0.987	0.885	9.8E-128	P1-1
Mfap5	4.3E-108	0.907	1	0.981	6.2E-104	P1-1
Sparc	1.4E-146	0.898	1	1	2.0E-142	P1-1
Phf11d	8.6E-75	0.881	0.746	0.263	1.2E-70	P1-3
Nova1	2.0E-97	0.880	0.936	0.726	2.9E-93	P1-2
Ackr3	2.2E-94	0.879	0.984	0.729	3.3E-90	P1-1
Tmem100	1.6E-90	0.876	0.937	0.615	2.3E-86	P1-1
Cd55	5.8E-89	0.869	0.824	0.473	8.4E-85	P1-2
Cldn10	1.3E-83	0.865	0.618	0.254	1.9E-79	P1-1
C4b	9.3E-42	0.864	0.856	0.516	1.4E-37	P1-3
Csf1	6.4E-43	0.857	0.893	0.654	9.2E-39	P1-3
Hspa1a	2.3E-18	0.853	0.608	0.408	3.3E-14	P1-3
Lgals9	1.6E-54	0.851	0.883	0.581	2.3E-50	P1-3
Qpct	7.8E-94	0.847	0.931	0.702	1.1E-89	P1-1
Adh7	1.5E-33	0.844	0.674	0.351	2.2E-29	P1-3
Oas1a	1.5E-102	0.840	0.636	0.137	2.2E-98	P1-3
Gfpt2	2.4E-78	0.837	0.842	0.546	3.5E-74	P1-1
Dpp4	1.6E-99	0.833	0.938	0.58	2.3E-95	P1-2

Mustn1	2.7E-90	0.824	0.758	0.335	4.0E-86	P1-1
Aldh1a3	2.9E-81	0.818	0.581	0.217	4.2E-77	P1-2
Pla1a	2.4E-24	0.812	0.911	0.788	3.5E-20	P1-3
Fbln2	4.4E-31	0.812	0.945	0.845	6.3E-27	P1-3
Ifi35	3.3E-61	0.810	0.78	0.366	4.9E-57	P1-3
Prss23	7.4E-79	0.805	0.978	0.844	1.1E-74	P1-2
Ifi203	1.9E-45	0.802	0.814	0.466	2.7E-41	P1-3
Rtp4	2.8E-49	0.797	0.708	0.315	4.0E-45	P1-3
Mpp6	3.6E-42	0.795	0.677	0.317	5.2E-38	P1-3
Adh1	1.5E-19	0.792	0.684	0.461	2.2E-15	P1-3
Psmb8	8.0E-45	0.788	0.89	0.648	1.2E-40	P1-3
Edn1	9.3E-56	0.782	0.575	0.26	1.4E-51	P1-2
Kif2a	1.8E-82	0.775	0.733	0.391	2.6E-78	P1-1
Irf4	8.2E-72	0.771	0.629	0.181	1.2E-67	P1-3
Gas7	8.2E-45	0.769	0.821	0.64	1.2E-40	P1-2
Plpp3	2.9E-91	0.764	0.998	0.983	4.2E-87	P1-1
Aspn	9.9E-69	0.758	0.957	0.782	1.4E-64	P1-1
C3	4.1E-59	0.757	0.919	0.73	5.9E-55	P1-2
Dact1	1.9E-82	0.752	0.691	0.318	2.7E-78	P1-1
Fndc1	2.4E-76	0.750	0.968	0.789	3.5E-72	P1-2
Camk2n1	2.2E-79	0.749	0.902	0.655	3.2E-75	P1-1
Apod	6.5E-30	0.748	0.916	0.815	9.5E-26	P1-2
Fbn1	3.9E-89	0.748	1	0.961	5.7E-85	P1-1
Gpc3	7.2E-66	0.747	0.953	0.734	1.1E-61	P1-2
Gcsh	4.3E-33	0.746	0.674	0.382	6.2E-29	P1-3
Clec3b	2.5E-125	0.741	1	0.972	3.6E-121	P1-1
Sp110	1.2E-52	0.741	0.704	0.301	1.7E-48	P1-3
Phf11b	1.0E-82	0.740	0.533	0.114	1.5E-78	P1-3
Dpp4	7.5E-88	0.735	0.958	0.583	1.1E-83	P1-1
Rab38	1.9E-100	0.734	0.541	0.153	2.8E-96	P1-1
Xaf1	2.4E-52	0.733	0.701	0.297	3.5E-48	P1-3
Tgfbr2	9.0E-46	0.728	0.986	0.863	1.3E-41	P1-3
Cd248	9.1E-97	0.726	1	0.946	1.3E-92	P1-1
Bst2	6.3E-50	0.725	0.962	0.875	9.2E-46	P1-2
Arl4d	4.1E-83	0.717	0.564	0.204	6.0E-79	P1-2
Aldh1a1	5.9E-35	0.717	0.866	0.614	8.5E-31	P1-3
Zbtb16	1.6E-65	0.715	0.773	0.471	2.4E-61	P1-2
Scd2	3.8E-72	0.714	0.831	0.53	5.6E-68	P1-1
Errfi1	6.2E-32	0.712	0.942	0.75	9.1E-28	P1-3
Col14a1	7.3E-75	0.711	0.987	0.814	1.1E-70	P1-2
Rnf213	9.0E-57	0.707	0.694	0.267	1.3E-52	P1-3
Slc16a9	1.9E-101	0.706	0.531	0.138	2.8E-97	P1-1
Lgals3bp	8.6E-49	0.702	0.986	0.894	1.2E-44	P1-3
Ly6a	1.3E-91	0.699	1	0.965	1.9E-87	P1-2
Pcolce2	6.8E-72	0.696	0.999	0.898	9.8E-68	P1-2

Jpt1	1.3E-81	0.695	0.968	0.822	1.9E-77	P1-1
Ogn	1.8E-50	0.694	1	0.945	2.7E-46	P1-3
Cxcl13	8.9E-30	0.693	0.677	0.359	1.3E-25	P1-3
Usp18	1.7E-40	0.692	0.584	0.245	2.4E-36	P1-3
Ackr3	3.1E-59	0.686	0.944	0.735	4.5E-55	P1-2
Fndc1	2.7E-84	0.684	0.992	0.786	4.0E-80	P1-1
Glul	1.2E-69	0.680	0.882	0.653	1.7E-65	P1-2
Lgals3	4.7E-24	0.679	0.787	0.547	6.8E-20	P1-3
Cd34	2.9E-94	0.678	1	0.961	4.2E-90	P1-2
Man1a	1.6E-79	0.675	0.963	0.756	2.3E-75	P1-2
Wnt2	4.2E-58	0.674	0.675	0.353	6.2E-54	P1-1
Anxa3	7.3E-68	0.674	0.99	0.792	1.1E-63	P1-2
Uchl1	9.4E-70	0.665	0.554	0.205	1.4E-65	P1-1
Anxa1	8.1E-82	0.659	0.999	0.977	1.2E-77	P1-2
Mustn1	1.6E-59	0.656	0.69	0.345	2.4E-55	P1-2
Gm26771	8.1E-58	0.652	0.517	0.207	1.2E-53	P1-1
Samhd1	9.3E-63	0.647	0.744	0.426	1.3E-58	P1-1
Tmem100	1.3E-26	0.647	0.89	0.661	1.8E-22	P1-3
Il33	1.7E-41	0.646	0.513	0.252	2.4E-37	P1-2
Iigp1	6.6E-41	0.644	0.687	0.309	9.6E-37	P1-3
Ifi205	1.0E-72	0.644	0.997	0.956	1.4E-68	P1-2
Ackr3	2.6E-32	0.643	0.973	0.762	3.8E-28	P1-3
Nampt	1.1E-44	0.643	0.704	0.325	1.6E-40	P1-3
Errfi1	4.2E-39	0.642	0.888	0.733	6.1E-35	P1-2
Car8	2.5E-62	0.640	0.616	0.28	3.7E-58	P1-2
Akr1c14	6.7E-39	0.639	0.51	0.252	9.7E-35	P1-2
Emilin2	8.1E-72	0.637	0.981	0.835	1.2E-67	P1-2
Arhgap20	3.7E-79	0.636	0.576	0.216	5.3E-75	P1-1
Marcks	7.2E-85	0.636	1	0.983	1.0E-80	P1-1
Tspo	2.0E-35	0.635	0.983	0.931	2.9E-31	P1-3
Ahnak2	2.0E-50	0.635	0.851	0.639	2.9E-46	P1-2
Igfbp5	6.7E-24	0.635	0.88	0.763	9.6E-20	P1-2
Serping1	3.0E-76	0.634	1	0.985	4.3E-72	P1-1
Zfp385a	2.8E-64	0.632	0.791	0.458	4.0E-60	P1-1
B2m	1.2E-62	0.632	1	1	1.7E-58	P1-2
Heg1	3.6E-63	0.630	0.926	0.72	5.2E-59	P1-1
Prdx5	3.6E-30	0.626	0.976	0.93	5.3E-26	P1-3
Il18	1.3E-64	0.625	0.734	0.37	2.0E-60	P1-1
Car8	2.5E-72	0.624	0.66	0.276	3.7E-68	P1-1
Tmem45a	3.0E-31	0.624	0.729	0.438	4.3E-27	P1-3
Mgll	1.4E-70	0.620	0.707	0.321	2.1E-66	P1-1
Tmem100	1.3E-52	0.620	0.9	0.619	1.9E-48	P1-2
Ugdh	1.3E-44	0.618	0.919	0.761	1.9E-40	P1-2
Timp2	1.9E-95	0.617	1	0.997	2.7E-91	P1-1
Tek	2.9E-72	0.616	0.671	0.308	4.1E-68	P1-1

Scara3	2.4E-54	0.614	0.876	0.647	3.5E-50	P1-2
Inmt	1.0E-09	0.613	0.725	0.628	1.5E-05	P1-3
Lamb2	1.7E-55	0.612	0.815	0.58	2.5E-51	P1-1
Timp3	2.3E-20	0.611	0.955	0.889	3.3E-16	P1-3
Metrnl	2.6E-55	0.610	0.963	0.867	3.8E-51	P1-2
Cd34	2.5E-43	0.608	1	0.966	3.6E-39	P1-3
Scara5	3.0E-63	0.608	0.97	0.747	4.3E-59	P1-2
Ankrd46	9.9E-47	0.608	0.646	0.268	1.4E-42	P1-3
Gbp3	8.3E-41	0.606	0.632	0.27	1.2E-36	P1-3
Gpx3	8.5E-22	0.604	0.968	0.898	1.2E-17	P1-2
Aff3	8.3E-51	0.604	0.55	0.179	1.2E-46	P1-3
Il17ra	7.0E-32	0.597	0.629	0.319	1.0E-27	P1-3
Slc43a3	4.2E-49	0.597	0.95	0.76	6.1E-45	P1-1
Sema3c	3.7E-60	0.593	0.87	0.556	5.4E-56	P1-1
Nid1	2.6E-99	0.593	1	0.994	3.8E-95	P1-1
Emilin2	1.5E-68	0.591	0.987	0.837	2.2E-64	P1-1
Cfb	1.3E-24	0.589	0.756	0.475	1.9E-20	P1-3
C4b	2.3E-29	0.587	0.706	0.503	3.3E-25	P1-2
Dbn1	3.2E-51	0.586	0.845	0.612	4.6E-47	P1-1
Anxa1	1.4E-33	0.583	1	0.98	2.1E-29	P1-3
Igfbp6	1.9E-66	0.583	1	0.987	2.8E-62	P1-2
Ogn	4.1E-60	0.582	0.997	0.937	6.0E-56	P1-2
Sp100	2.9E-37	0.581	0.66	0.317	4.2E-33	P1-3
Basp1	2.6E-48	0.581	0.924	0.777	3.7E-44	P1-1
Fgfr1	7.7E-66	0.578	0.962	0.826	1.1E-61	P1-2
Ppa1	2.0E-31	0.577	0.694	0.377	2.9E-27	P1-3
Plpp3	3.6E-62	0.574	0.999	0.983	5.1E-58	P1-2
Il13ra1	2.3E-64	0.573	0.588	0.254	3.4E-60	P1-2
Ppp1r14b	1.0E-57	0.571	0.929	0.715	1.5E-53	P1-1
Ebf2	7.9E-34	0.571	0.858	0.738	1.1E-29	P1-2
1700019D03Rik	4.2E-52	0.571	0.731	0.434	6.1E-48	P1-2
Fbxw17	2.3E-61	0.571	0.56	0.16	3.3E-57	P1-3
Csrp2	2.8E-51	0.571	0.993	0.957	4.0E-47	P1-2
Uap1	1.0E-38	0.570	0.866	0.711	1.5E-34	P1-2
Islr	9.7E-68	0.568	0.991	0.887	1.4E-63	P1-2
Adgrd1	1.0E-54	0.568	0.829	0.566	1.5E-50	P1-1
Gbp2	1.2E-29	0.567	0.632	0.327	1.7E-25	P1-3
Psme1	7.8E-29	0.564	0.876	0.679	1.1E-24	P1-3
Tgfb2	7.3E-26	0.557	0.601	0.313	1.1E-21	P1-3
Adamts5	1.4E-55	0.557	0.994	0.905	2.1E-51	P1-1
Hspa5	1.1E-38	0.555	0.976	0.932	1.6E-34	P1-1
Ifi27l2a	4.6E-38	0.554	0.997	0.957	6.6E-34	P1-2
Lims2	1.5E-48	0.554	0.657	0.373	2.2E-44	P1-2
Axl	2.5E-61	0.551	0.99	0.918	3.6E-57	P1-2
Pamr1	9.1E-41	0.548	0.66	0.405	1.3E-36	P1-2

Axl	4.2E-63	0.547	0.992	0.92	6.1E-59	P1-1
Thbd	7.3E-13	0.547	0.66	0.473	1.1E-08	P1-3
Ar	2.0E-50	0.546	0.976	0.852	2.8E-46	P1-1
Creb5	3.7E-46	0.544	0.871	0.638	5.3E-42	P1-1
Gm31378	2.2E-38	0.544	0.521	0.273	3.2E-34	P1-2
Ifi207	4.7E-23	0.543	0.835	0.631	6.9E-19	P1-3
Stat1	1.5E-44	0.543	0.57	0.21	2.2E-40	P1-3
Mgll	1.3E-54	0.542	0.656	0.327	1.9E-50	P1-2
Ccdc80	2.5E-34	0.542	1	0.986	3.6E-30	P1-1
Il6ra	5.1E-54	0.541	0.549	0.25	7.4E-50	P1-2
Lbp	3.3E-14	0.540	0.725	0.526	4.8E-10	P1-3
Sema3b	1.4E-49	0.540	0.614	0.32	2.0E-45	P1-1
Clec2d	2.3E-24	0.538	0.863	0.669	3.3E-20	P1-3
Col1a1	2.9E-39	0.537	1	0.997	4.3E-35	P1-1
Ugdh	1.2E-42	0.536	0.926	0.763	1.8E-38	P1-1
Npc2	1.1E-37	0.536	1	0.994	1.6E-33	P1-3
Ccl11	5.5E-22	0.535	0.889	0.842	7.9E-18	P1-2
Marcks	7.8E-51	0.534	0.996	0.984	1.1E-46	P1-2
Hspb8	8.8E-42	0.532	0.675	0.421	1.3E-37	P1-1
H2-K1	4.3E-35	0.531	0.985	0.956	6.3E-31	P1-2
Bmp1	5.1E-54	0.530	0.944	0.811	7.4E-50	P1-1
Creb5	1.9E-45	0.529	0.864	0.634	2.8E-41	P1-2
Poglut3	1.3E-43	0.528	0.738	0.487	1.9E-39	P1-1
Lrrn4cl	2.6E-52	0.527	0.923	0.673	3.8E-48	P1-1
Il6ra	1.4E-39	0.526	0.66	0.282	2.0E-35	P1-3
Steap4	4.4E-161	1.525	0.86	0.244	6.4E-157	P2-1
Fn1	5.4E-149	-2.076	0.922	0.994	7.9E-145	P2-1
Col4a1	4.3E-147	1.780	1	0.787	6.2E-143	P2-1
Gsn	1.7E-140	1.517	1	1	2.4E-136	P2-1
Cxcl14	1.8E-121	2.110	0.688	0.179	2.5E-117	P2-1
Igfbp6	1.3E-113	-1.617	0.97	0.993	1.9E-109	P2-1
Jund	2.4E-113	1.115	1	0.997	3.5E-109	P2-1
Rplp1	8.4E-113	-0.654	1	1	1.2E-108	P2-1
Col4a2	1.3E-111	1.323	0.992	0.676	1.9E-107	P2-1
Nrep	2.4E-110	1.482	0.93	0.53	3.5E-106	P2-1
Sparcl1	2.6E-110	1.523	0.863	0.345	3.8E-106	P2-1
Zfp36	5.6E-106	1.423	0.995	0.875	8.1E-102	P2-1
Col15a1	1.3E-103	1.342	0.944	0.476	1.9E-99	P2-1
Rps24	2.5E-97	-0.580	1	1	3.6E-93	P2-1
Col5a3	7.3E-95	1.170	0.978	0.773	1.1E-90	P2-1
Jun	2.9E-94	1.102	1	0.994	4.2E-90	P2-1
Junb	9.7E-93	1.113	0.997	0.974	1.4E-88	P2-1
Egr1	1.3E-92	1.014	1	0.976	1.8E-88	P2-1
Anxa3	6.8E-91	-2.185	0.602	0.872	9.9E-87	P2-1
Socs3	1.1E-89	1.457	0.984	0.753	1.6E-85	P2-1

Rps20	2.1E-88	-0.557	1	1	3.1E-84	P2-1
Pi16	3.0E-86	-3.103	0.769	0.916	4.3E-82	P2-1
Rps12	1.3E-85	-0.648	1	1	1.9E-81	P2-1
Pcolce	8.1E-85	1.027	1	0.969	1.2E-80	P2-1
Smoc2	2.9E-83	1.353	0.86	0.469	4.2E-79	P2-1
Igfbp7	1.5E-82	0.996	1	0.939	2.2E-78	P2-1
Col6a2	3.0E-82	0.959	1	0.983	4.4E-78	P2-1
Col6a1	1.1E-80	0.775	1	0.995	1.5E-76	P2-1
Btg1	1.5E-78	1.080	0.978	0.755	2.2E-74	P2-1
Gask1b	4.5E-78	0.962	0.728	0.308	6.6E-74	P2-1
Pla1a	5.8E-78	-1.529	0.489	0.846	8.4E-74	P2-1
Rps10	9.2E-78	-0.579	1	1	1.3E-73	P2-1
Tmsb4x	1.9E-77	-0.813	1	1	2.8E-73	P2-1
Slfn5	1.3E-76	1.060	0.987	0.875	1.9E-72	P2-1
Eef1b2	2.4E-76	-0.733	0.978	0.998	3.4E-72	P2-1
Rps15	3.3E-76	-0.526	1	1	4.8E-72	P2-1
Islr	3.7E-76	-1.060	0.726	0.938	5.4E-72	P2-1
Fosb	1.0E-75	1.137	0.968	0.832	1.5E-71	P2-1
Anxa1	3.7E-74	-1.070	0.93	0.99	5.4E-70	P2-1
Sfrp4	5.6E-74	-1.879	0.371	0.759	8.1E-70	P2-1
Gpc3	3.2E-73	-1.250	0.457	0.832	4.7E-69	P2-1
Plac8	3.4E-70	-2.023	0.844	0.951	4.9E-66	P2-1
Mfap5	6.1E-70	-1.193	0.97	0.987	8.9E-66	P2-1
Csrp2	7.9E-70	-1.068	0.914	0.972	1.2E-65	P2-1
Igf1	1.3E-68	1.163	0.944	0.733	1.8E-64	P2-1
Dpp4	6.3E-68	-1.316	0.309	0.714	9.1E-64	P2-1
Insig1	2.0E-67	1.034	0.642	0.255	3.0E-63	P2-1
Itm2b	5.4E-61	0.618	1	1	7.8E-57	P2-1
Hmgcs1	5.8E-61	1.182	0.626	0.274	8.5E-57	P2-1
Rpl31	1.7E-59	-0.510	1	1	2.4E-55	P2-1
Ier2	3.3E-59	0.952	0.995	0.935	4.8E-55	P2-1
Dbi	1.2E-58	0.859	1	0.949	1.7E-54	P2-1
Efemp1	2.0E-58	-0.975	0.898	0.959	2.9E-54	P2-1
Cygb	2.3E-57	0.800	0.968	0.729	3.4E-53	P2-1
Emilin2	1.3E-56	-0.938	0.683	0.895	1.8E-52	P2-1
Pcolce2	4.1E-55	-1.218	0.89	0.926	5.9E-51	P2-1
Fst	9.0E-55	1.048	0.922	0.75	1.3E-50	P2-1
Axl	1.9E-54	-0.873	0.887	0.942	2.7E-50	P2-1
Gsc	1.4E-53	0.721	0.519	0.185	2.0E-49	P2-1
Cdkn1c	1.4E-53	-1.425	0.352	0.718	2.0E-49	P2-1
Enpp2	7.5E-53	0.908	0.551	0.206	1.1E-48	P2-1
Cd63	8.8E-53	0.801	1	0.998	1.3E-48	P2-1
Smpd3	9.4E-53	-2.318	0.414	0.702	1.4E-48	P2-1
Dbn1	1.7E-52	-0.879	0.336	0.708	2.5E-48	P2-1
Rpl38	3.0E-52	-0.520	1	1	4.4E-48	P2-1

Plpp1	3.1E-52	0.898	0.642	0.32	4.6E-48	P2-1
Nfib	2.2E-50	0.653	0.995	0.917	3.2E-46	P2-1
Scara3	3.2E-50	-0.868	0.401	0.743	4.6E-46	P2-1
Tmem158	5.5E-50	-1.197	0.473	0.76	8.0E-46	P2-1
S100a11	3.0E-49	-0.600	1	1	4.3E-45	P2-1
Col3a1	1.7E-48	0.575	1	1	2.4E-44	P2-1
Tuba1a	8.6E-47	0.755	0.997	0.993	1.3E-42	P2-1
Dbp	1.6E-46	-1.044	0.384	0.694	2.3E-42	P2-1
Sema3c	1.7E-46	-1.181	0.339	0.664	2.5E-42	P2-1
Plat	8.8E-46	-0.781	0.575	0.852	1.3E-41	P2-1
AW112010	1.1E-45	0.882	0.871	0.593	1.7E-41	P2-1
Fos	1.5E-45	0.667	1	0.994	2.2E-41	P2-1
Ptgis	6.3E-45	-0.795	0.212	0.605	9.1E-41	P2-1
G0s2	1.9E-44	0.892	0.608	0.306	2.7E-40	P2-1
Selenop	2.0E-44	0.629	1	0.963	2.9E-40	P2-1
Myl6	2.0E-44	-0.599	0.992	0.998	3.0E-40	P2-1
Mt1	4.1E-44	1.081	0.997	0.975	6.0E-40	P2-1
Htra3	1.0E-43	0.720	0.992	0.963	1.5E-39	P2-1
Gas7	1.7E-43	-1.152	0.419	0.72	2.5E-39	P2-1
Il17d	3.3E-43	0.567	0.556	0.232	4.8E-39	P2-1
Creb3l1	1.2E-42	0.747	0.788	0.534	1.8E-38	P2-1
Efhd1	1.6E-42	-0.969	0.185	0.553	2.4E-38	P2-1
Col14a1	2.1E-42	-0.914	0.704	0.876	3.0E-38	P2-1
Fbn1	2.8E-42	-0.835	0.949	0.972	4.1E-38	P2-1
Sfrp2	6.6E-41	-1.886	0.704	0.833	9.6E-37	P2-1
Creb5	7.4E-41	-0.802	0.401	0.729	1.1E-36	P2-1
Adam19	9.0E-41	0.626	0.57	0.268	1.3E-36	P2-1
Rhoj	1.7E-40	0.615	0.949	0.806	2.5E-36	P2-1
Il1r1	2.2E-40	0.661	0.624	0.334	3.2E-36	P2-1
Pamr1	6.5E-40	-0.805	0.148	0.51	9.4E-36	P2-1
Il11ra1	1.3E-39	0.592	1	0.968	1.8E-35	P2-1
Tmem100	2.1E-39	-1.161	0.481	0.713	3.1E-35	P2-1
Serpinb6a	3.6E-39	-0.640	0.981	0.993	5.2E-35	P2-1
Nid2	1.3E-38	0.585	0.511	0.228	1.8E-34	P2-1
Nav1	1.4E-38	0.628	0.898	0.733	2.1E-34	P2-1
Cd248	1.7E-38	-0.709	0.914	0.964	2.4E-34	P2-1
Loxl1	2.0E-38	-0.523	0.989	0.991	2.9E-34	P2-1
Akr1c18	3.3E-38	-1.638	0.177	0.502	4.8E-34	P2-1
Sash1	9.4E-38	0.673	0.745	0.493	1.4E-33	P2-1
Neat1	1.8E-37	0.800	0.968	0.9	2.6E-33	P2-1
Ftl1	2.1E-37	0.527	1	1	3.1E-33	P2-1
Metrnl	2.4E-37	-0.706	0.758	0.908	3.5E-33	P2-1
Irf7	3.0E-37	-1.304	0.298	0.597	4.4E-33	P2-1
Adam12	3.1E-37	0.502	0.567	0.241	4.5E-33	P2-1
Rarres2	5.6E-37	0.548	1	0.993	8.2E-33	P2-1

Plpp3	7.2E-37	-0.803	0.995	0.985	1.0E-32	P2-1
Lrrn4cl	5.7E-36	-0.821	0.5	0.758	8.2E-32	P2-1
Ubc	2.1E-35	0.641	0.995	0.986	3.1E-31	P2-1
Auts2	8.7E-35	0.543	0.726	0.399	1.3E-30	P2-1
Hspg2	1.7E-34	0.582	0.941	0.859	2.5E-30	P2-1
Fyn	2.0E-34	-0.603	0.567	0.81	2.9E-30	P2-1
Mmp3	2.9E-34	0.816	0.855	0.615	4.1E-30	P2-1
Errfi1	5.4E-34	-0.904	0.578	0.797	7.8E-30	P2-1
Ackr3	9.3E-34	-1.097	0.68	0.798	1.4E-29	P2-1
Bmper	9.7E-34	0.544	0.578	0.278	1.4E-29	P2-1
H2-D1	9.7E-34	0.709	1	0.989	1.4E-29	P2-1
Uqcc2	1.9E-33	-0.660	0.823	0.918	2.8E-29	P2-1
Ccl11	2.1E-33	0.571	0.976	0.835	3.0E-29	P2-1
Osr1	3.7E-33	-0.701	0.457	0.738	5.4E-29	P2-1
Procr	4.1E-33	-0.927	0.484	0.728	5.9E-29	P2-1
1700019D03Rik	5.1E-33	-0.713	0.228	0.542	7.3E-29	P2-1
Nfia	6.8E-33	0.544	0.952	0.855	9.9E-29	P2-1
Tgoln1	1.0E-32	0.581	0.825	0.638	1.4E-28	P2-1
Nmt2	1.8E-32	-0.688	0.43	0.702	2.7E-28	P2-1
Cadm3	2.6E-32	-0.725	0.215	0.529	3.8E-28	P2-1
S100a10	2.7E-32	-0.514	1	1	4.0E-28	P2-1
Acaa2	3.5E-32	-0.563	0.513	0.788	5.0E-28	P2-1
Ugdh	1.2E-31	-0.762	0.618	0.824	1.7E-27	P2-1
Ly6a	2.0E-31	-0.839	0.997	0.97	2.9E-27	P2-1
Dnajb1	2.1E-31	1.296	0.68	0.475	3.0E-27	P2-1
Cd34	4.4E-31	-0.614	0.987	0.967	6.4E-27	P2-1
Timp2	9.8E-31	-0.559	0.997	0.998	1.4E-26	P2-1
Cxcl1	8.1E-30	0.907	0.806	0.588	1.2E-25	P2-1
Samhd1	1.3E-29	-0.626	0.226	0.533	2.0E-25	P2-1
Gadd45g	1.4E-29	1.081	0.847	0.696	2.1E-25	P2-1
Svep1	1.5E-29	0.565	0.669	0.405	2.2E-25	P2-1
Ablim1	1.6E-29	-0.620	0.519	0.747	2.3E-25	P2-1
Gnpnat1	2.9E-29	-0.582	0.196	0.503	4.2E-25	P2-1
Ifrd1	4.8E-29	0.830	0.704	0.503	6.9E-25	P2-1
H2-K1	7.0E-29	0.628	0.995	0.958	1.0E-24	P2-1
Tnfaip6	1.1E-28	0.751	0.927	0.787	1.6E-24	P2-1
Igfbp5	1.6E-28	0.592	0.957	0.766	2.3E-24	P2-1
Tgfbr2	2.0E-28	-0.668	0.782	0.889	2.9E-24	P2-1
Zbtb16	2.6E-28	-0.636	0.285	0.578	3.8E-24	P2-1
Cd55	5.2E-28	-0.828	0.336	0.586	7.5E-24	P2-1
Igfbp4	9.3E-28	-0.768	0.938	0.932	1.4E-23	P2-1
Lgals1	1.5E-27	-0.646	1	0.999	2.2E-23	P2-1
Ifi27	2.3E-27	0.599	0.925	0.813	3.4E-23	P2-1
Poglut3	2.3E-27	-0.563	0.293	0.576	3.4E-23	P2-1
Adamts5	3.2E-27	0.566	0.973	0.916	4.6E-23	P2-1

Abca1	5.7E-27	-0.556	0.331	0.617	8.2E-23	P2-1
Ppp1r15a	9.0E-27	0.566	0.806	0.611	1.3E-22	P2-1
Postn	9.8E-27	-1.084	0.965	0.979	1.4E-22	P2-1
Mycbp2	1.2E-26	0.520	0.772	0.561	1.7E-22	P2-1
Plod1	3.8E-26	0.590	0.597	0.374	5.5E-22	P2-1
Mmp23	1.6E-25	-0.534	0.747	0.877	2.4E-21	P2-1
Tgfbr3	1.8E-25	-0.527	0.645	0.809	2.6E-21	P2-1
Flrt2	2.2E-24	-0.580	0.425	0.654	3.2E-20	P2-1
Scara5	2.6E-24	-0.682	0.704	0.812	3.8E-20	P2-1
Fndc1	1.6E-23	-0.724	0.788	0.836	2.3E-19	P2-1
Vgll3	2.2E-23	-0.543	0.234	0.502	3.2E-19	P2-1
Frmd6	2.4E-23	0.515	0.839	0.657	3.5E-19	P2-1
Cotl1	6.6E-23	-0.537	0.551	0.751	9.5E-19	P2-1
Atf4	7.4E-23	0.515	0.863	0.772	1.1E-18	P2-1
Adgrd1	1.3E-22	-0.511	0.401	0.654	1.9E-18	P2-1
Atp6v1e1	1.6E-22	-0.517	0.554	0.747	2.3E-18	P2-1
Hk2	2.7E-22	0.934	0.715	0.52	4.0E-18	P2-1
Nbl1	1.7E-21	-0.551	0.868	0.925	2.5E-17	P2-1
Ppp1r14b	2.4E-21	-0.570	0.621	0.78	3.5E-17	P2-1
Bst2	1.4E-20	-0.980	0.863	0.9	2.1E-16	P2-1
Nr4a1	4.3E-20	-0.767	0.277	0.516	6.2E-16	P2-1
Apod	5.0E-20	-0.789	0.739	0.852	7.2E-16	P2-1
Nova1	1.5E-19	-0.614	0.677	0.788	2.2E-15	P2-1
Aldh1a1	5.2E-19	-0.514	0.465	0.665	7.5E-15	P2-1
Jpt1	6.6E-19	-0.527	0.763	0.866	9.5E-15	P2-1
Heg1	2.0E-18	-0.514	0.648	0.78	3.0E-14	P2-1
Scd2	1.8E-17	0.544	0.731	0.574	2.6E-13	P2-1
Gng12	7.0E-17	-0.554	0.704	0.817	1.0E-12	P2-1
Il1r2	1.7E-15	-0.941	0.382	0.544	2.5E-11	P2-1
Ccn1	2.0E-15	-0.698	0.685	0.816	2.8E-11	P2-1
Cebpb	2.1E-14	0.501	0.952	0.908	3.1E-10	P2-1
Ifitm3	3.6E-14	-0.525	0.997	1	5.2E-10	P2-1
H2-T23	1.5E-13	-0.514	0.478	0.634	2.2E-09	P2-1
Mt2	1.3E-12	0.584	0.919	0.865	1.9E-08	P2-1
Lgals3	1.1E-11	-0.541	0.441	0.59	1.6E-07	P2-1
Phlda1	1.4E-10	0.635	0.841	0.765	2.0E-06	P2-1
Ccl7	1.2E-08	1.481	0.505	0.413	1.8E-04	P2-1
Cd9	1.9E-08	-0.566	0.621	0.679	2.7E-04	P2-1
Hmcn1	1.3E-303	1.898	0.693	0.054	1.8E-299	P2-2
Sdc1	4.9E-277	1.745	0.752	0.115	7.1E-273	P2-2
Cdh11	3.9E-269	1.476	0.787	0.141	5.7E-265	P2-2
Clec3b	4.3E-249	-1.484	0.926	0.995	6.2E-245	P2-2
Ly6a	3.1E-235	-1.840	0.903	0.997	4.5E-231	P2-2
Col18a1	3.7E-209	1.808	0.826	0.31	5.4E-205	P2-2
Frem1	1.1E-205	0.902	0.513	0.042	1.5E-201	P2-2

Serping1	3.9E-204	-1.351	0.964	0.996	5.7E-200	P2-2
Ptger3	1.9E-196	1.000	0.595	0.085	2.7E-192	P2-2
Peg3	2.3E-191	1.698	0.761	0.242	3.3E-187	P2-2
Scara5	4.6E-189	-1.521	0.489	0.904	6.7E-185	P2-2
Postn	1.3E-187	1.750	0.993	0.972	1.9E-183	P2-2
Lum	1.7E-185	1.219	0.996	0.989	2.4E-181	P2-2
Angptl2	1.3E-183	1.448	0.979	0.842	1.9E-179	P2-2
Palld	1.3E-180	1.497	0.774	0.273	1.9E-176	P2-2
C3	7.1E-178	-1.508	0.427	0.892	1.0E-173	P2-2
Gpx3	1.0E-177	-2.092	0.752	0.969	1.5E-173	P2-2
Mdk	1.6E-177	1.481	0.702	0.193	2.3E-173	P2-2
Smpd3	2.7E-177	-2.914	0.286	0.795	3.9E-173	P2-2
Scg3	2.0E-172	1.606	0.584	0.109	2.9E-168	P2-2
Pcolce2	9.3E-171	-1.516	0.777	0.971	1.4E-166	P2-2
Col15a1	1.1E-170	1.248	0.926	0.402	1.6E-166	P2-2
Gsn	2.9E-168	-1.257	1	1	4.2E-164	P2-2
Ackr3	5.1E-164	-1.630	0.478	0.887	7.4E-160	P2-2
Anxa3	7.1E-164	-2.007	0.644	0.904	1.0E-159	P2-2
Timp2	2.0E-163	-0.972	0.995	0.999	2.9E-159	P2-2
Slc43a3	2.1E-160	-1.252	0.479	0.91	3.0E-156	P2-2
Pi16	1.2E-159	-2.888	0.766	0.941	1.7E-155	P2-2
Cd34	1.8E-159	-1.083	0.906	0.991	2.6E-155	P2-2
Lgals1	3.0E-157	1.009	1	0.999	4.3E-153	P2-2
Tmem100	4.4E-157	-1.615	0.325	0.806	6.3E-153	P2-2
Ogn	3.0E-156	-1.222	0.842	0.988	4.3E-152	P2-2
Sfrp4	2.0E-155	-1.943	0.38	0.823	2.9E-151	P2-2
Fth1	5.7E-154	-0.728	1	1	8.2E-150	P2-2
Rbp1	1.4E-153	1.184	0.914	0.52	2.0E-149	P2-2
Ldhb	2.6E-152	1.140	0.612	0.153	3.7E-148	P2-2
Dpp4	1.4E-148	-1.386	0.303	0.785	2.1E-144	P2-2
Col4a2	4.0E-146	1.155	0.97	0.629	5.7E-142	P2-2
Nxn	5.2E-139	0.908	0.523	0.108	7.5E-135	P2-2
Sh3bgrl3	9.8E-138	1.094	0.983	0.882	1.4E-133	P2-2
Srpx	1.4E-137	1.102	0.965	0.812	2.0E-133	P2-2
Fndc1	3.8E-137	-1.195	0.587	0.913	5.6E-133	P2-2
Plpp3	2.9E-136	-1.165	0.968	0.993	4.2E-132	P2-2
Col5a2	4.7E-135	0.849	0.996	0.99	6.8E-131	P2-2
Fabp4	3.9E-132	2.803	0.797	0.444	5.7E-128	P2-2
Ifi27l2a	2.4E-130	-1.411	0.898	0.989	3.5E-126	P2-2
Slc9a3r2	9.3E-130	0.799	0.604	0.165	1.3E-125	P2-2
Adam12	1.1E-126	0.949	0.607	0.171	1.6E-122	P2-2
Txndc5	1.7E-123	0.875	0.992	0.909	2.5E-119	P2-2
Lrrn4cl	2.9E-122	-1.181	0.447	0.82	4.2E-118	P2-2
Prss23	6.8E-121	-1.138	0.674	0.943	9.9E-117	P2-2
Igfbp5	3.0E-120	-2.033	0.577	0.863	4.3E-116	P2-2

Tcf4	2.7E-119	1.279	0.966	0.869	3.9E-115	P2-2
Ar	1.5E-117	-0.952	0.686	0.943	2.1E-113	P2-2
Col4a1	9.1E-116	0.942	0.984	0.756	1.3E-111	P2-2
Sema3c	9.7E-114	-1.304	0.307	0.73	1.4E-109	P2-2
Arid5b	1.1E-112	0.846	0.682	0.261	1.5E-108	P2-2
Igfbp7	4.3E-111	0.894	0.997	0.929	6.3E-107	P2-2
Serpine1	4.3E-109	1.306	0.55	0.163	6.2E-105	P2-2
Procr	5.7E-109	-1.356	0.455	0.779	8.2E-105	P2-2
Cd9	2.4E-107	0.998	0.899	0.595	3.5E-103	P2-2
Ecm1	2.3E-106	-1.022	0.791	0.937	3.4E-102	P2-2
Prelp	2.9E-105	-0.915	0.303	0.734	4.2E-101	P2-2
Angptl4	5.4E-105	1.115	0.758	0.379	7.8E-101	P2-2
Man1a	1.1E-104	-0.888	0.566	0.884	1.6E-100	P2-2
Cadm3	1.2E-101	-0.994	0.166	0.599	1.8E-97	P2-2
Gsto1	2.2E-100	0.722	0.556	0.179	3.3E-96	P2-2
Col27a1	5.5E-100	0.682	0.556	0.168	7.9E-96	P2-2
Aspn	5.1E-99	-0.971	0.583	0.899	7.4E-95	P2-2
Itga2b	2.5E-98	0.973	0.54	0.182	3.6E-94	P2-2
Fscn1	4.3E-98	0.791	0.67	0.277	6.2E-94	P2-2
Adamts12	9.1E-98	0.804	0.714	0.333	1.3E-93	P2-2
Nova1	1.8E-96	-0.959	0.557	0.848	2.6E-92	P2-2
Sparcl1	3.9E-96	1.009	0.732	0.302	5.7E-92	P2-2
Mgll	5.6E-96	-0.871	0.078	0.514	8.1E-92	P2-2
Gfpt2	6.1E-96	-1.009	0.319	0.707	8.9E-92	P2-2
Axl	1.4E-95	-0.840	0.859	0.961	2.0E-91	P2-2
Eid1	2.4E-95	0.833	0.877	0.639	3.5E-91	P2-2
Ppp1r14b	4.9E-95	-0.919	0.549	0.832	7.1E-91	P2-2
Il18	5.5E-95	-0.942	0.134	0.554	8.0E-91	P2-2
Itm2a	6.6E-95	0.918	0.981	0.93	9.6E-91	P2-2
Pgrmc1	4.3E-93	0.876	0.878	0.676	6.2E-89	P2-2
Tshz2	9.4E-92	-0.797	0.561	0.877	1.4E-87	P2-2
Mustn1	2.3E-91	-1.032	0.111	0.532	3.4E-87	P2-2
Anxa5	4.5E-91	0.739	0.992	0.941	6.5E-87	P2-2
Tmem119	2.3E-90	0.766	0.73	0.355	3.4E-86	P2-2
Abi3bp	3.4E-88	-0.781	0.63	0.906	4.9E-84	P2-2
Qpct	2.4E-87	-0.868	0.523	0.828	3.5E-83	P2-2
Timp3	1.2E-84	-0.713	0.718	0.957	1.7E-80	P2-2
Sgk1	6.5E-84	0.937	0.699	0.351	9.4E-80	P2-2
Tpbg	2.9E-83	0.696	0.581	0.233	4.2E-79	P2-2
Plac8	4.4E-83	-1.684	0.878	0.958	6.4E-79	P2-2
Tsix	6.3E-83	0.651	0.69	0.3	9.2E-79	P2-2
Sh3pxd2a	9.1E-82	0.672	0.58	0.23	1.3E-77	P2-2
Snhg18	1.0E-81	0.641	0.985	0.912	1.5E-77	P2-2
Mafb	1.4E-81	1.145	0.579	0.23	2.0E-77	P2-2
Cthrc1	1.5E-81	1.113	0.541	0.205	2.2E-77	P2-2

Blvrb	4.3E-80	-0.690	0.695	0.895	6.3E-76	P2-2
Gna12	2.1E-79	0.600	0.525	0.192	3.0E-75	P2-2
Gadd45g	3.5E-79	-1.472	0.483	0.794	5.1E-75	P2-2
Dpt	1.7E-78	-0.507	0.996	0.998	2.5E-74	P2-2
Eva1b	3.2E-78	0.669	0.895	0.672	4.6E-74	P2-2
Emilin2	9.0E-78	-0.814	0.777	0.899	1.3E-73	P2-2
1810058I24Rik	2.1E-77	0.713	0.968	0.871	3.0E-73	P2-2
Tceal9	7.5E-77	0.604	0.993	0.944	1.1E-72	P2-2
H2-D1	1.5E-75	-0.698	0.966	0.998	2.1E-71	P2-2
Gstt1	2.7E-75	-0.691	0.525	0.848	3.9E-71	P2-2
Cd55	5.9E-75	-0.953	0.295	0.643	8.6E-71	P2-2
Fkbp9	4.9E-74	0.583	0.987	0.956	7.1E-70	P2-2
Gadd45b	1.9E-73	0.832	0.609	0.268	2.7E-69	P2-2
Cald1	2.4E-73	0.689	0.98	0.917	3.5E-69	P2-2
Scara3	1.4E-72	-0.781	0.497	0.769	2.0E-68	P2-2
Tgfbr2	1.7E-72	-0.748	0.741	0.921	2.5E-68	P2-2
Prrx1	3.3E-72	-0.676	0.803	0.94	4.7E-68	P2-2
Akr1c18	6.1E-72	-1.568	0.204	0.548	8.9E-68	P2-2
Ace	6.4E-72	-0.870	0.556	0.816	9.3E-68	P2-2
Bmper	1.8E-71	0.651	0.564	0.232	2.6E-67	P2-2
Il1r2	9.5E-71	-1.372	0.282	0.606	1.4E-66	P2-2
Cotl1	2.7E-70	0.765	0.879	0.674	3.9E-66	P2-2
Ifitm3	2.7E-70	-0.740	0.999	1	3.9E-66	P2-2
Lamb1	5.4E-70	0.680	0.871	0.65	7.8E-66	P2-2
Auts2	6.5E-70	0.694	0.689	0.356	9.4E-66	P2-2
Hspg2	7.6E-70	0.617	0.953	0.841	1.1E-65	P2-2
Cfb	2.2E-69	-0.922	0.235	0.594	3.2E-65	P2-2
Heg1	3.5E-69	-0.745	0.58	0.826	5.1E-65	P2-2
Phlda3	5.1E-69	0.587	0.621	0.28	7.4E-65	P2-2
1700019D03Rik	5.2E-68	-0.710	0.224	0.597	7.6E-64	P2-2
Gda	9.4E-68	-0.690	0.24	0.6	1.4E-63	P2-2
Efhd1	2.1E-67	-0.955	0.268	0.587	3.1E-63	P2-2
Cryab	7.8E-67	-0.775	0.385	0.713	1.1E-62	P2-2
Abracl	1.6E-66	0.658	0.889	0.711	2.3E-62	P2-2
Thbd	5.7E-66	-0.845	0.238	0.578	8.3E-62	P2-2
Mfap2	4.3E-65	0.601	0.988	0.973	6.2E-61	P2-2
Cd248	7.7E-65	-0.682	0.923	0.969	1.1E-60	P2-2
Emilin1	8.0E-65	0.694	0.658	0.37	1.2E-60	P2-2
Timp1	1.3E-64	0.938	0.64	0.339	1.9E-60	P2-2
Col5a3	1.7E-64	0.651	0.932	0.754	2.5E-60	P2-2
C1qtnf12	2.2E-64	0.541	0.529	0.214	3.3E-60	P2-2
Tmem158	5.2E-64	-1.032	0.549	0.783	7.6E-60	P2-2
Tagln2	9.0E-64	0.750	0.903	0.735	1.3E-59	P2-2
Tusc3	3.3E-63	0.692	0.769	0.526	4.8E-59	P2-2
Sod3	3.5E-63	-0.606	0.685	0.894	5.1E-59	P2-2

Atpif1	7.3E-63	0.517	0.988	0.95	1.1E-58	P2-2
Sbsn	1.4E-62	-1.515	0.228	0.535	2.1E-58	P2-2
Fgfr1	2.0E-62	-0.615	0.73	0.9	2.9E-58	P2-2
Anxa1	3.7E-62	-0.718	0.98	0.983	5.4E-58	P2-2
Creg1	5.0E-62	0.668	0.879	0.689	7.2E-58	P2-2
Uqcc2	1.2E-61	-0.686	0.839	0.929	1.7E-57	P2-2
Igfbp6	1.4E-61	-0.774	0.991	0.99	2.1E-57	P2-2
Rcn3	3.4E-61	0.518	0.996	0.991	5.0E-57	P2-2
Mmp19	5.9E-61	0.625	0.603	0.303	8.6E-57	P2-2
Apbb1ip	8.2E-61	0.687	0.703	0.431	1.2E-56	P2-2
Serpinb6a	1.6E-60	-0.583	0.988	0.993	2.3E-56	P2-2
Irs2	2.2E-60	0.658	0.588	0.283	3.2E-56	P2-2
Ptprs	2.9E-60	0.525	0.583	0.278	4.2E-56	P2-2
Txn1	4.7E-60	-0.524	0.991	0.999	6.8E-56	P2-2
Myl12a	6.4E-60	0.538	0.999	0.991	9.2E-56	P2-2
Fkbp10	8.5E-60	0.616	0.809	0.581	1.2E-55	P2-2
Emp3	2.6E-59	-0.591	0.907	0.972	3.8E-55	P2-2
Lims2	1.0E-58	-0.662	0.197	0.521	1.5E-54	P2-2
Marcks	1.2E-58	-0.623	0.979	0.989	1.7E-54	P2-2
Adamts5	1.7E-58	-0.704	0.839	0.952	2.5E-54	P2-2
Xdh	4.7E-58	-0.619	0.384	0.69	6.8E-54	P2-2
Gstm1	7.8E-58	-0.559	0.89	0.978	1.1E-53	P2-2
Adam23	9.8E-58	0.683	0.679	0.403	1.4E-53	P2-2
Zfp385a	3.8E-56	-0.680	0.31	0.603	5.5E-52	P2-2
Rarres2	9.9E-56	-0.537	0.984	0.997	1.4E-51	P2-2
Ccdc50	1.1E-55	0.596	0.736	0.499	1.6E-51	P2-2
Ahnak2	1.3E-55	-0.643	0.463	0.765	1.9E-51	P2-2
Rcn1	6.7E-55	0.526	0.981	0.927	9.7E-51	P2-2
Tle5	9.8E-55	-0.539	0.723	0.89	1.4E-50	P2-2
Glul	2.4E-54	-0.633	0.53	0.766	3.5E-50	P2-2
Tpm4	3.5E-54	0.622	0.917	0.783	5.0E-50	P2-2
Ugdh	7.2E-54	-0.756	0.656	0.846	1.0E-49	P2-2
D630033O11Rik	4.7E-53	0.613	0.514	0.238	6.9E-49	P2-2
Mgp	4.9E-53	1.359	0.519	0.242	7.2E-49	P2-2
Samhd1	5.5E-53	-0.647	0.272	0.569	8.0E-49	P2-2
Nbl1	6.5E-53	-0.516	0.817	0.952	9.5E-49	P2-2
H2-K1	7.6E-53	-0.624	0.901	0.984	1.1E-48	P2-2
Ech1	8.7E-53	0.630	0.874	0.719	1.3E-48	P2-2
Thy1	1.5E-52	0.721	0.934	0.811	2.2E-48	P2-2
Ccnd2	6.5E-52	0.720	0.871	0.698	9.5E-48	P2-2
Ifngr2	1.9E-51	0.544	0.613	0.335	2.7E-47	P2-2
Uap1	2.3E-51	-0.656	0.596	0.798	3.4E-47	P2-2
Fam102b	4.1E-51	0.631	0.757	0.547	5.9E-47	P2-2
Pofut2	4.6E-51	0.528	0.82	0.635	6.7E-47	P2-2
Jpt1	5.2E-51	-0.661	0.777	0.878	7.5E-47	P2-2

Fgl2	6.9E-51	-0.714	0.442	0.727	1.0E-46	P2-2
Selenop	3.5E-50	-0.606	0.921	0.984	5.0E-46	P2-2
Ebf2	1.1E-49	-0.658	0.621	0.814	1.6E-45	P2-2
Ctsk	1.8E-49	0.619	0.966	0.916	2.7E-45	P2-2
Lamb2	9.9E-49	-0.595	0.443	0.693	1.4E-44	P2-2
Maf	1.1E-48	0.630	0.906	0.791	1.6E-44	P2-2
Dpysl3	1.3E-48	0.637	0.789	0.615	1.9E-44	P2-2
Fbn1	2.0E-48	-0.606	0.941	0.979	2.8E-44	P2-2
C4b	4.6E-48	-0.896	0.352	0.617	6.7E-44	P2-2
Csf1	8.1E-48	-0.596	0.493	0.741	1.2E-43	P2-2
Pmp22	1.9E-47	-0.561	0.805	0.931	2.8E-43	P2-2
Errfi1	2.1E-47	-0.833	0.636	0.814	3.0E-43	P2-2
Lbp	2.4E-47	-0.716	0.349	0.613	3.5E-43	P2-2
Osr2	2.7E-47	-0.649	0.501	0.734	3.9E-43	P2-2
Pdlim2	3.8E-47	0.538	0.83	0.62	5.6E-43	P2-2
Ddah2	8.8E-47	-0.502	0.827	0.927	1.3E-42	P2-2
Rhob	1.6E-46	0.618	0.811	0.606	2.3E-42	P2-2
Camk2n1	1.7E-46	-0.640	0.572	0.753	2.4E-42	P2-2
Fhl1	2.4E-46	-0.606	0.348	0.622	3.5E-42	P2-2
Ifi211	5.1E-46	-0.676	0.713	0.863	7.5E-42	P2-2
Ttc3	6.5E-46	0.510	0.758	0.531	9.4E-42	P2-2
Pde8a	2.1E-45	-0.547	0.246	0.529	3.1E-41	P2-2
Ddr2	8.7E-45	-0.511	0.817	0.927	1.3E-40	P2-2
St3gal1	3.7E-44	-0.597	0.458	0.707	5.4E-40	P2-2
Bst2	8.6E-44	-0.937	0.813	0.923	1.3E-39	P2-2
Penk	4.3E-43	0.935	0.626	0.39	6.2E-39	P2-2
Prrx2	5.3E-42	0.569	0.626	0.381	7.7E-38	P2-2
Ugp2	6.6E-42	-0.520	0.464	0.69	9.5E-38	P2-2
Lysmd2	9.2E-42	0.545	0.863	0.726	1.3E-37	P2-2
Slc25a5	9.6E-42	0.525	0.945	0.882	1.4E-37	P2-2
Triobp	2.1E-41	-0.540	0.385	0.619	3.1E-37	P2-2
Col14a1	3.9E-41	-0.511	0.702	0.906	5.6E-37	P2-2
Klf4	2.4E-40	-0.596	0.906	0.98	3.4E-36	P2-2
Mfap5	2.7E-40	-0.670	0.979	0.987	4.0E-36	P2-2
Sox4	2.3E-39	0.580	0.671	0.432	3.3E-35	P2-2
Gng11	5.4E-39	0.602	0.766	0.57	7.8E-35	P2-2
Cdkn1c	9.5E-39	1.122	0.783	0.633	1.4E-34	P2-2
Klf3	1.6E-38	-0.557	0.435	0.659	2.4E-34	P2-2
Fkbp11	1.9E-38	0.518	0.718	0.503	2.7E-34	P2-2
Sat1	3.4E-38	0.637	0.942	0.89	5.0E-34	P2-2
Creb5	5.1E-38	-0.569	0.533	0.739	7.4E-34	P2-2
Btg2	2.2E-37	0.642	0.956	0.878	3.1E-33	P2-2
Oaf	3.1E-37	0.585	0.95	0.904	4.5E-33	P2-2
Slc38a2	2.9E-35	0.503	0.87	0.755	4.2E-31	P2-2
Ddx3x	6.7E-35	0.504	0.82	0.701	9.7E-31	P2-2

Pcolce	2.4E-34	-0.650	0.965	0.976	3.5E-30	P2-2
Ifi205	2.4E-34	-0.639	0.913	0.984	3.5E-30	P2-2
Lgals9	2.9E-34	-0.502	0.446	0.667	4.3E-30	P2-2
Phlda1	6.1E-33	-0.600	0.642	0.82	8.9E-29	P2-2
AW112010	6.2E-33	-0.592	0.468	0.683	9.0E-29	P2-2
Ckb	3.6E-32	-0.637	0.423	0.606	5.2E-28	P2-2
Scd2	3.4E-27	-0.541	0.468	0.637	5.0E-23	P2-2
Apod	5.4E-26	-0.638	0.753	0.867	7.8E-22	P2-2
Irf7	1.1E-25	-0.794	0.435	0.601	1.6E-21	P2-2
Smoc2	1.5E-23	-0.560	0.37	0.569	2.2E-19	P2-2
Neat1	7.6E-20	0.552	0.942	0.897	1.1E-15	P2-2
Mt2	5.2E-19	-0.617	0.791	0.899	7.6E-15	P2-2
Gas7	2.9E-18	-0.611	0.608	0.707	4.3E-14	P2-2
Cxcl1	3.5E-11	-0.566	0.544	0.641	5.1E-07	P2-2
Igf1	6.8E-11	-0.544	0.726	0.771	9.8E-07	P2-2
Hk2	3.2E-09	-0.521	0.47	0.57	4.7E-05	P2-2
Ggt5	3.0E-29	0.523	0.698	0.364	4.3E-25	P1-3
Prelp	4.3E-48	0.520	0.836	0.568	6.2E-44	P1-1
Lamc1	1.0E-56	0.519	0.984	0.944	1.5E-52	P1-1
Nmt2	2.0E-39	0.518	0.824	0.621	2.9E-35	P1-2
Sulf2	7.1E-49	0.517	0.617	0.317	1.0E-44	P1-1
Sdf2l1	4.2E-43	0.517	0.554	0.286	6.2E-39	P1-1
Gda	1.6E-22	0.514	0.753	0.481	2.3E-18	P1-3
Triobp	3.1E-28	0.513	0.828	0.53	4.5E-24	P1-3
Timp2	1.1E-68	0.513	1	0.997	1.6E-64	P1-2
Pik3ip1	5.6E-51	0.509	0.535	0.254	8.1E-47	P1-2
Vegfd	1.1E-21	0.507	0.787	0.556	1.6E-17	P1-3
Psmb10	6.0E-21	0.506	0.732	0.525	8.7E-17	P1-3
Ddah2	7.2E-58	0.505	0.981	0.881	1.1E-53	P1-1
Ablim1	1.1E-38	0.505	0.851	0.679	1.6E-34	P1-2
Snx18	3.1E-48	0.504	0.628	0.334	4.5E-44	P1-1
Npc2	6.3E-31	0.503	0.996	0.995	9.1E-27	P1-2
Fhl1	3.0E-25	0.500	0.677	0.515	4.4E-21	P1-2
Tnfaip6	2.1E-11	-0.500	0.747	0.821	3.0E-07	P1-1
Pcolce	1.7E-25	-0.503	0.959	0.977	2.5E-21	P1-2
Ltbp4	1.1E-25	-0.513	0.308	0.51	1.6E-21	P1-1
Zbtb16	1.9E-21	-0.515	0.412	0.575	2.7E-17	P1-1
Slc25a5	6.3E-25	-0.516	0.842	0.913	9.1E-21	P1-1
AW112010	3.1E-09	-0.516	0.564	0.646	4.5E-05	P1-1
Cd63	3.8E-17	-0.517	1	0.998	5.5E-13	P1-3
Fos	7.6E-12	-0.517	0.99	0.995	1.1E-07	P1-3
Eva1b	4.8E-29	-0.517	0.609	0.761	6.9E-25	P1-1
Egfr	1.0E-34	-0.517	0.666	0.815	1.5E-30	P1-2
Col5a2	8.8E-44	-0.518	0.987	0.993	1.3E-39	P1-2
Rrbp1	4.9E-50	-0.520	0.979	0.995	7.1E-46	P1-2

Angptl2	1.4E-07	-0.520	0.857	0.883	2.1E-03	P1-2
C4b	2.5E-08	-0.522	0.493	0.565	3.7E-04	P1-1
Apbb1ip	1.4E-26	-0.525	0.338	0.544	2.1E-22	P1-1
Anxa5	8.0E-17	-0.528	0.921	0.958	1.2E-12	P1-3
Socs3	6.9E-16	-0.529	0.718	0.799	1.0E-11	P1-1
Rhob	2.5E-12	-0.530	0.509	0.674	3.6E-08	P1-3
Ly6e	3.2E-24	-0.532	0.937	0.971	4.7E-20	P1-1
Adam23	3.2E-27	-0.541	0.304	0.518	4.6E-23	P1-1
Fbln2	1.9E-23	-0.542	0.783	0.875	2.7E-19	P1-1
Akap12	1.3E-22	-0.544	0.378	0.654	1.9E-18	P1-3
Jun	1.6E-23	-0.547	0.994	0.995	2.3E-19	P1-2
Tcf4	3.7E-11	-0.547	0.883	0.897	5.4E-07	P1-2
Fosb	2.4E-17	-0.550	0.783	0.869	3.4E-13	P1-2
Smoc2	1.3E-05	-0.554	0.436	0.528	1.8E-01	P1-3
Creb3l1	1.6E-33	-0.555	0.391	0.618	2.4E-29	P1-2
Gas1	2.0E-26	-0.556	0.814	0.888	2.9E-22	P1-2
Tubb5	1.4E-23	-0.556	0.878	0.936	2.0E-19	P1-1
Abca8a	6.7E-16	-0.560	0.344	0.565	9.7E-12	P1-3
Nupr1	5.4E-23	-0.563	0.918	0.963	7.8E-19	P1-3
Postn	1.5E-07	-0.563	0.99	0.974	2.2E-03	P1-2
Hspg2	1.1E-19	-0.563	0.811	0.876	1.5E-15	P1-3
Fam102b	2.7E-19	-0.565	0.388	0.623	3.9E-15	P1-3
Hk2	3.5E-13	-0.567	0.436	0.577	5.0E-09	P1-2
Fn1	2.7E-15	-0.567	0.997	0.983	4.0E-11	P1-3
Slc25a5	1.1E-33	-0.569	0.846	0.913	1.5E-29	P1-2
Ckb	2.5E-11	-0.571	0.423	0.574	3.6E-07	P1-3
Ctsk	1.9E-14	-0.571	0.893	0.933	2.7E-10	P1-3
Hspa5	3.2E-37	-0.575	0.901	0.953	4.6E-33	P1-2
Smoc2	6.6E-17	-0.576	0.397	0.555	9.6E-13	P1-2
Junb	4.2E-24	-0.585	0.968	0.98	6.1E-20	P1-1
Snhg18	3.4E-23	-0.588	0.876	0.937	5.0E-19	P1-3
Btg1	1.7E-27	-0.591	0.657	0.817	2.5E-23	P1-1
B2m	9.1E-35	-0.594	1	1	1.3E-30	P1-1
Ctsk	1.9E-29	-0.603	0.895	0.938	2.8E-25	P1-1
Neat1	3.8E-11	-0.605	0.825	0.918	5.5E-07	P1-3
Aebp1	3.8E-25	-0.610	0.993	0.993	5.5E-21	P1-3
Fosb	2.5E-09	-0.611	0.821	0.852	3.7E-05	P1-3
Anxa5	2.2E-44	-0.615	0.91	0.968	3.3E-40	P1-2
Fkbp9	3.4E-31	-0.616	0.911	0.969	5.0E-27	P1-3
Srpx	7.4E-18	-0.616	0.857	0.849	1.1E-13	P1-1
Jun	7.2E-12	-0.617	0.986	0.995	1.0E-07	P1-3
Zfp36	1.0E-15	-0.618	0.849	0.902	1.5E-11	P1-1
Eva1b	6.7E-24	-0.628	0.526	0.751	9.7E-20	P1-3
Fmo1	3.9E-44	-0.629	0.222	0.522	5.6E-40	P1-1
Fst	1.4E-14	-0.630	0.625	0.788	2.1E-10	P1-3

Cd63	2.3E-49	-0.635	0.998	0.998	3.3E-45	P1-1
Ccnd2	1.2E-19	-0.648	0.564	0.762	1.7E-15	P1-3
Fosb	1.3E-24	-0.669	0.8	0.862	1.9E-20	P1-1
Auts2	1.3E-43	-0.671	0.225	0.505	2.0E-39	P1-2
Col1a1	1.7E-54	-0.673	1	0.997	2.4E-50	P1-2
Adamts5	1.5E-21	-0.676	0.887	0.928	2.2E-17	P1-3
Ifi27	8.8E-46	-0.677	0.713	0.862	1.3E-41	P1-2
Ccnd2	4.8E-40	-0.688	0.612	0.781	7.0E-36	P1-2
Psap	1.3E-44	-0.692	0.976	0.992	1.9E-40	P1-3
Col1a2	4.6E-71	-0.695	1	1	6.7E-67	P1-2
Col5a2	2.9E-34	-0.696	0.979	0.993	4.3E-30	P1-3
Itm2a	5.6E-21	-0.705	0.89	0.949	8.1E-17	P1-3
Sparc	1.4E-72	-0.706	1	1	2.1E-68	P1-2
Igf1	1.5E-21	-0.711	0.557	0.782	2.2E-17	P1-3
Col6a1	1.3E-45	-0.713	0.99	0.996	1.9E-41	P1-3
Tcf4	9.0E-17	-0.717	0.814	0.903	1.3E-12	P1-3
Angptl2	1.7E-22	-0.718	0.868	0.879	2.5E-18	P1-1
Col3a1	2.6E-78	-0.719	1	1	3.7E-74	P1-2
Bst2	9.8E-11	-0.725	0.902	0.894	1.4E-06	P1-1
Col1a2	7.4E-37	-0.726	1	1	1.1E-32	P1-3
Ccl11	1.8E-25	-0.732	0.815	0.863	2.6E-21	P1-1
Igf1	5.2E-27	-0.735	0.66	0.789	7.5E-23	P1-2
Col5a3	1.0E-16	-0.736	0.694	0.81	1.5E-12	P1-3
Ifi207	2.4E-51	-0.741	0.436	0.708	3.4E-47	P1-1
Lum	2.9E-17	-0.742	1	0.99	4.2E-13	P1-3
Col5a3	9.4E-30	-0.742	0.731	0.817	1.4E-25	P1-1
Lgals3bp	8.1E-49	-0.743	0.849	0.918	1.2E-44	P1-1
Thbs2	5.1E-24	-0.746	0.568	0.719	7.4E-20	P1-1
Plod2	7.5E-49	-0.747	0.188	0.501	1.1E-44	P1-1
Egr1	2.1E-22	-0.747	0.962	0.981	3.0E-18	P1-3
Thy1	2.3E-46	-0.751	0.686	0.885	3.3E-42	P1-1
Tcf4	1.5E-30	-0.782	0.841	0.908	2.2E-26	P1-1
Tmem119	3.1E-60	-0.787	0.172	0.525	4.5E-56	P1-1
Irf7	8.9E-10	-0.794	0.507	0.573	1.3E-05	P1-1
Col5a3	1.7E-41	-0.798	0.696	0.83	2.5E-37	P1-2
Col3a1	1.2E-43	-0.801	1	1	1.8E-39	P1-3
Col1a1	3.3E-37	-0.803	1	0.997	4.8E-33	P1-3
Isg15	8.0E-15	-0.815	0.361	0.51	1.2E-10	P1-1
Sparc	9.8E-45	-0.818	1	1	1.4E-40	P1-3
Jun	2.6E-48	-0.833	0.994	0.995	3.8E-44	P1-1
Sgk1	5.9E-50	-0.840	0.187	0.507	8.5E-46	P1-1
Neat1	1.5E-37	-0.843	0.849	0.926	2.2E-33	P1-2
Klf9	7.9E-78	-0.847	0.907	0.971	1.1E-73	P1-1
Srpx	2.5E-52	-0.860	0.77	0.875	3.6E-48	P1-2
Rbp1	5.5E-24	-0.925	0.402	0.644	7.9E-20	P1-3

Nrep	1.0E-49	-0.939	0.375	0.643	1.5E-45	P1-2
Lgals1	1.5E-108	-0.940	1	0.999	2.2E-104	P1-1
Adh1	1.4E-57	-0.946	0.224	0.553	2.0E-53	P1-1
Cd9	3.8E-24	-0.950	0.436	0.698	5.6E-20	P1-3
Tnfaip6	2.5E-17	-0.958	0.646	0.822	3.6E-13	P1-3
Cd9	2.3E-44	-0.961	0.488	0.721	3.3E-40	P1-1
Igfbp5	3.9E-16	-0.971	0.619	0.809	5.6E-12	P1-3
Ifi205	5.4E-63	-0.972	0.974	0.963	7.9E-59	P1-1
Mt1	1.4E-16	-0.973	0.973	0.978	2.0E-12	P1-3
Sh3bgrl3	5.2E-68	-0.978	0.816	0.932	7.6E-64	P1-1
Col6a2	1.1E-53	-0.983	0.962	0.988	1.6E-49	P1-3
Cygb	2.6E-52	-0.983	0.591	0.805	3.7E-48	P1-1
Gas1	6.2E-39	-0.984	0.622	0.898	8.9E-35	P1-3
Inmt	1.6E-22	-0.992	0.514	0.671	2.3E-18	P1-1
Angptl2	8.4E-35	-0.995	0.708	0.895	1.2E-30	P1-3
Mmp3	1.1E-46	-1.016	0.425	0.704	1.6E-42	P1-1
Cxcl1	1.1E-11	-1.019	0.45	0.634	1.6E-07	P1-3
Angptl4	5.9E-74	-1.044	0.166	0.559	8.6E-70	P1-1
Junb	4.0E-37	-1.079	0.938	0.981	5.9E-33	P1-3
Cdkn1c	9.0E-24	-1.082	0.426	0.698	1.3E-19	P1-3
Rbp1	3.2E-71	-1.094	0.346	0.694	4.6E-67	P1-1
Socs3	4.3E-30	-1.151	0.557	0.807	6.2E-26	P1-3
Col4a2	1.2E-21	-1.164	0.581	0.731	1.8E-17	P1-3
Srpx	1.3E-53	-1.187	0.588	0.88	2.0E-49	P1-3
Zfp36	6.0E-28	-1.192	0.794	0.901	8.7E-24	P1-3
Spon1	1.7E-76	-1.249	0.1	0.503	2.5E-72	P1-1
Ifi27	6.3E-74	-1.293	0.443	0.87	9.2E-70	P1-3
Col4a1	2.2E-18	-1.298	0.742	0.822	3.2E-14	P1-3
Nrep	9.5E-49	-1.301	0.175	0.626	1.4E-44	P1-3
Col15a1	7.2E-26	-1.309	0.302	0.561	1.0E-21	P1-3
Igfbp7	4.4E-65	-1.330	0.922	0.954	6.5E-61	P1-2
Dbp	3.0E-127	-1.413	0.279	0.756	4.3E-123	P1-1
Col4a2	5.3E-86	-1.516	0.516	0.776	7.7E-82	P1-2
Lum	3.9E-165	-1.547	0.969	0.997	5.6E-161	P1-1
Col15a1	2.7E-82	-1.569	0.263	0.617	3.9E-78	P1-2
Igfbp7	2.5E-89	-1.594	0.897	0.96	3.6E-85	P1-1
Col4a2	2.6E-96	-1.639	0.47	0.782	3.8E-92	P1-1
Igfbp7	4.5E-47	-1.646	0.89	0.953	6.5E-43	P1-3
Col4a1	5.6E-74	-1.683	0.709	0.846	8.1E-70	P1-2
Sfrp2	1.0E-39	-1.714	0.574	0.844	1.5E-35	P1-3
Col15a1	2.6E-108	-1.766	0.156	0.637	3.7E-104	P1-1
Postn	1.1E-61	-1.770	0.918	0.984	1.6E-57	P1-3
Col4a1	8.6E-110	-1.946	0.614	0.868	1.2E-105	P1-1
Fabp4	1.9E-20	-2.491	0.326	0.557	2.7E-16	P1-3
Fabp4	1.8E-36	-2.655	0.388	0.578	2.7E-32	P1-2

Fabp4	3.5E-49	-2.748	0.327	0.59	5.1E-45	P1-1
Gas6	2.8E-58	1.955	0.8	0.303	4.1E-54	P3-Areg
Fmo2	2.2E-72	1.928	0.63	0.147	3.1E-68	P3-Areg
Gdf10	2.4E-52	1.839	0.83	0.415	3.4E-48	P3-Areg
Mgp	3.3E-37	1.828	0.673	0.291	4.8E-33	P3-Areg
Inmt	3.0E-31	1.665	0.885	0.623	4.3E-27	P3-Areg
Igfbp7	3.9E-55	1.517	0.994	0.944	5.7E-51	P3-Areg
Clec11a	3.1E-70	1.451	0.576	0.118	4.5E-66	P3-Areg
Igfbp3	2.1E-24	1.422	0.515	0.214	3.0E-20	P3-Areg
Cst3	5.9E-40	1.402	0.994	0.995	8.5E-36	P3-Areg
Cygb	7.4E-46	1.371	0.982	0.746	1.1E-41	P3-Areg
Nmb	3.9E-39	1.355	0.533	0.171	5.6E-35	P3-Areg
F3	1.8E-43	1.327	0.509	0.133	2.6E-39	P3-Areg
Steap4	3.2E-42	1.200	0.733	0.298	4.6E-38	P3-Areg
Tgfbi	1.7E-24	1.181	0.624	0.306	2.5E-20	P3-Areg
Cd9	4.7E-28	1.170	0.958	0.655	6.8E-24	P3-Areg
Gadd45g	6.3E-14	1.155	0.867	0.706	9.1E-10	P3-Areg
C2	3.6E-28	1.058	0.63	0.301	5.2E-24	P3-Areg
Abi3bp	7.4E-36	1.035	0.976	0.828	1.1E-31	P3-Areg
Igf1	1.8E-17	1.022	0.927	0.749	2.6E-13	P3-Areg
Mmp3	9.3E-14	0.982	0.879	0.631	1.4E-09	P3-Areg
Sfrp4	7.1E-08	0.969	0.903	0.699	1.0E-03	P3-Areg
Vit	3.5E-72	0.961	0.545	0.095	5.0E-68	P3-Areg
Ndrg2	1.4E-24	0.883	0.521	0.227	2.0E-20	P3-Areg
Gsn	6.2E-22	0.837	1	1	9.0E-18	P3-Areg
Selenop	6.6E-30	0.833	0.994	0.966	9.6E-26	P3-Areg
Pmp22	3.7E-03	0.819	0.939	0.896	1.0E+00	P3-Areg
Nfib	3.0E-22	0.710	0.976	0.924	4.3E-18	P3-Areg
Osr1	3.0E-11	0.708	0.848	0.693	4.3E-07	P3-Areg
Cav1	7.0E-19	0.703	0.636	0.354	1.0E-14	P3-Areg
Serping1	4.1E-25	0.698	0.994	0.988	6.0E-21	P3-Areg
Il11ra1	3.0E-23	0.686	0.994	0.971	4.3E-19	P3-Areg
Sned1	2.5E-06	0.676	0.503	0.396	3.6E-02	P3-Areg
Apod	1.4E-06	0.666	0.952	0.831	2.0E-02	P3-Areg
Junb	1.2E-15	0.659	0.994	0.976	1.7E-11	P3-Areg
Socs3	1.1E-13	0.632	0.958	0.772	1.6E-09	P3-Areg
AW112010	6.3E-09	0.629	0.782	0.619	9.2E-05	P3-Areg
Cryab	9.0E-13	0.624	0.842	0.617	1.3E-08	P3-Areg
Fth1	1.8E-33	0.624	1	1	2.6E-29	P3-Areg
Nrp1	1.1E-09	0.609	0.545	0.374	1.6E-05	P3-Areg
Deptor	4.7E-21	0.594	0.515	0.225	6.8E-17	P3-Areg
Tmeff2	7.3E-09	0.582	0.903	0.803	1.1E-04	P3-Areg
Rbp1	1.8E-15	0.576	0.873	0.605	2.6E-11	P3-Areg
Cd81	9.3E-21	0.563	0.994	0.997	1.3E-16	P3-Areg
Ecm2	3.9E-10	0.555	0.527	0.356	5.7E-06	P3-Areg

Cd302	9.2E-15	0.538	0.988	0.942	1.3E-10	P3-Areg
Jun	4.1E-15	0.538	1	0.994	5.9E-11	P3-Areg
Rpl22l1	8.1E-16	0.501	1	1	1.2E-11	P3-Areg
Nmt2	2.4E-09	-0.503	0.485	0.679	3.5E-05	P3-Areg
Jpt1	2.0E-09	-0.513	0.812	0.855	3.0E-05	P3-Areg
Ccnd2	2.5E-08	-0.515	0.636	0.749	3.6E-04	P3-Areg
Lbp	3.2E-08	-0.515	0.345	0.558	4.6E-04	P3-Areg
Pdpn	4.4E-11	-0.516	0.485	0.691	6.4E-07	P3-Areg
Nfkbia	4.3E-04	-0.517	0.927	0.904	1.0E+00	P3-Areg
Ifi27l2a	1.8E-08	-0.525	0.964	0.966	2.6E-04	P3-Areg
Rps12	7.9E-27	-0.527	1	1	1.2E-22	P3-Areg
Neat1	9.3E-05	-0.533	0.939	0.907	1.0E+00	P3-Areg
1700019D03Rik	7.0E-12	-0.533	0.23	0.518	1.0E-07	P3-Areg
Ext1	4.2E-14	-0.538	0.206	0.52	6.2E-10	P3-Areg
Procr	2.6E-05	-0.539	0.661	0.699	3.8E-01	P3-Areg
Loxl2	8.5E-15	-0.540	0.818	0.897	1.2E-10	P3-Areg
Meg3	2.8E-09	-0.540	0.594	0.736	4.0E-05	P3-Areg
Fstl1	6.7E-21	-0.541	0.988	0.997	9.7E-17	P3-Areg
Bst2	3.0E-04	-0.548	0.915	0.894	1.0E+00	P3-Areg
Lsp1	1.0E-11	-0.550	0.715	0.812	1.5E-07	P3-Areg
Fndc1	3.5E-08	-0.551	0.855	0.828	5.1E-04	P3-Areg
Gfpt2	3.1E-06	-0.556	0.521	0.614	4.5E-02	P3-Areg
Metrnl	4.2E-12	-0.561	0.794	0.895	6.1E-08	P3-Areg
Tgfbr2	1.3E-10	-0.568	0.885	0.875	1.9E-06	P3-Areg
Plpp3	3.5E-11	-0.573	0.988	0.986	5.1E-07	P3-Areg
Ptgis	6.7E-14	-0.578	0.285	0.571	9.8E-10	P3-Areg
Klf2	5.9E-06	-0.597	0.891	0.88	8.6E-02	P3-Areg
Zfp385a	3.1E-13	-0.600	0.267	0.544	4.5E-09	P3-Areg
H2-K1	4.1E-11	-0.610	0.945	0.964	6.0E-07	P3-Areg
Ier5	1.4E-07	-0.616	0.364	0.528	2.1E-03	P3-Areg
Npr1	1.1E-19	-0.620	0.152	0.537	1.6E-15	P3-Areg
Cotl1	1.6E-13	-0.623	0.545	0.737	2.3E-09	P3-Areg
Thy1	2.4E-10	-0.630	0.818	0.844	3.4E-06	P3-Areg
Nova1	2.9E-11	-0.635	0.691	0.779	4.2E-07	P3-Areg
Tcf4	1.1E-07	-0.636	0.891	0.894	1.5E-03	P3-Areg
Prss23	2.1E-12	-0.638	0.848	0.876	3.0E-08	P3-Areg
Irf7	3.2E-03	-0.643	0.521	0.561	1.0E+00	P3-Areg
Plat	3.4E-16	-0.645	0.703	0.823	4.9E-12	P3-Areg
Axl	1.7E-15	-0.647	0.909	0.936	2.4E-11	P3-Areg
Itih5	5.5E-26	-0.663	0.861	0.976	8.0E-22	P3-Areg
Lamb1	1.6E-14	-0.664	0.527	0.717	2.3E-10	P3-Areg
Ugdh	3.4E-10	-0.683	0.77	0.799	5.0E-06	P3-Areg
Il1r2	6.8E-05	-0.687	0.424	0.529	9.8E-01	P3-Areg
Angptl2	2.4E-10	-0.692	0.848	0.878	3.5E-06	P3-Areg
Basp1	3.5E-14	-0.699	0.709	0.814	5.1E-10	P3-Areg

Tnfaip6	8.5E-05	-0.701	0.782	0.806	1.0E+00	P3-Areg
Col5a3	4.3E-11	-0.704	0.679	0.806	6.2E-07	P3-Areg
Timp3	2.0E-17	-0.707	0.855	0.898	2.9E-13	P3-Areg
Dbn1	1.7E-16	-0.719	0.448	0.674	2.4E-12	P3-Areg
Islr	1.3E-19	-0.720	0.83	0.916	1.9E-15	P3-Areg
Anxa1	2.3E-18	-0.753	0.97	0.983	3.3E-14	P3-Areg
Oaf	1.1E-22	-0.769	0.794	0.923	1.5E-18	P3-Areg
Creb5	6.5E-17	-0.783	0.509	0.698	9.4E-13	P3-Areg
Cd55	1.8E-12	-0.789	0.339	0.567	2.6E-08	P3-Areg
Igfbp6	1.8E-17	-0.797	0.988	0.99	2.6E-13	P3-Areg
Aspn	6.5E-18	-0.799	0.673	0.827	9.5E-14	P3-Areg
Maf	6.1E-23	-0.802	0.642	0.831	8.9E-19	P3-Areg
Mfap5	5.4E-17	-0.814	0.994	0.984	7.9E-13	P3-Areg
Thbs2	5.2E-14	-0.835	0.497	0.699	7.5E-10	P3-Areg
Adamts5	1.3E-22	-0.853	0.83	0.929	1.9E-18	P3-Areg
Lrrn4cl	8.7E-15	-0.859	0.624	0.732	1.3E-10	P3-Areg
Sema3c	3.5E-09	-0.861	0.545	0.627	5.1E-05	P3-Areg
Loxl1	6.2E-40	-0.875	0.945	0.993	9.0E-36	P3-Areg
Emilin2	6.1E-24	-0.881	0.758	0.875	8.9E-20	P3-Areg
Fbn1	4.0E-24	-0.884	0.927	0.971	5.8E-20	P3-Areg
Gas7	3.4E-09	-0.888	0.612	0.686	5.0E-05	P3-Areg
Tmem100	4.8E-10	-0.937	0.636	0.686	6.9E-06	P3-Areg
Cdkn1c	1.7E-09	-0.944	0.552	0.678	2.4E-05	P3-Areg
Dpp4	5.2E-16	-0.945	0.521	0.671	7.6E-12	P3-Areg
Ccn1	5.0E-08	-0.966	0.8	0.799	7.2E-04	P3-Areg
Efhd1	7.6E-20	-0.973	0.182	0.526	1.1E-15	P3-Areg
Nid1	1.4E-43	-0.979	0.97	0.997	2.0E-39	P3-Areg
Fn1	5.3E-29	-1.109	0.982	0.985	7.6E-25	P3-Areg
Cd248	8.4E-39	-1.133	0.855	0.964	1.2E-34	P3-Areg
Igfbp5	4.5E-06	-1.166	0.836	0.788	6.5E-02	P3-Areg
Efemp1	2.7E-38	-1.208	0.83	0.959	3.9E-34	P3-Areg
Marcks	4.1E-42	-1.215	0.964	0.988	6.0E-38	P3-Areg
Ackr3	7.0E-21	-1.329	0.727	0.786	1.0E-16	P3-Areg
Smpd3	4.9E-09	-1.669	0.642	0.667	7.1E-05	P3-Areg
Anxa3	1.5E-22	-1.680	0.788	0.841	2.2E-18	P3-Areg
Pla1a	1.6E-39	-1.707	0.545	0.816	2.3E-35	P3-Areg
Postn	1.6E-44	-1.955	0.939	0.98	2.3E-40	P3-Areg
Pi16	2.1E-20	-2.392	0.915	0.896	3.0E-16	P3-Areg
Pclaf	0.0E+00	3.167	0.968	0.014	0.0E+00	P4
Top2a	0.0E+00	2.903	0.903	0.017	0.0E+00	P4
Birc5	0.0E+00	2.685	0.935	0.019	0.0E+00	P4
Cenpf	0.0E+00	2.566	0.823	0.008	0.0E+00	P4
Ccnb2	0.0E+00	2.343	0.774	0.01	0.0E+00	P4
Prc1	0.0E+00	2.278	0.839	0.007	0.0E+00	P4
Mki67	0.0E+00	2.193	0.935	0.006	0.0E+00	P4

Tk1	0.0E+00	2.108	0.887	0.011	0.0E+00	P4
Pbk	0.0E+00	1.838	0.806	0.004	0.0E+00	P4
Ccna2	0.0E+00	1.796	0.79	0.006	0.0E+00	P4
Tpx2	0.0E+00	1.774	0.758	0.008	0.0E+00	P4
Mxd3	0.0E+00	1.692	0.774	0.008	0.0E+00	P4
Hmmr	0.0E+00	1.484	0.661	0.002	0.0E+00	P4
Ckap2l	0.0E+00	1.473	0.79	0.009	0.0E+00	P4
Pimreg	0.0E+00	1.449	0.694	0.003	0.0E+00	P4
Sgo1	0.0E+00	1.233	0.742	0.008	0.0E+00	P4
Ska1	0.0E+00	1.077	0.597	0.001	0.0E+00	P4
Knl1	0.0E+00	1.030	0.597	0.002	0.0E+00	P4
Ccnb1	2.9E-307	1.682	0.613	0.003	4.2E-303	P4
Sgo2a	1.9E-302	1.094	0.645	0.005	2.7E-298	P4
Plk1	7.8E-301	1.106	0.613	0.004	1.1E-296	P4
Cdca3	6.6E-293	2.365	0.855	0.016	9.6E-289	P4
Spc24	1.3E-287	1.837	0.871	0.018	1.8E-283	P4
Asf1b	1.5E-259	1.261	0.661	0.009	2.2E-255	P4
Melk	8.1E-258	0.860	0.532	0.003	1.2E-253	P4
Nuf2	1.2E-255	0.946	0.548	0.004	1.7E-251	P4
Cdca8	1.8E-253	1.976	0.903	0.025	2.7E-249	P4
Kif11	7.5E-249	1.069	0.581	0.006	1.1E-244	P4
Mis18bp1	4.0E-246	0.910	0.532	0.004	5.9E-242	P4
Kifc1	6.3E-245	1.001	0.613	0.008	9.1E-241	P4
Cenpe	1.1E-244	1.588	0.581	0.006	1.6E-240	P4
Ncaph	2.0E-244	0.766	0.548	0.005	2.9E-240	P4
Cenph	5.4E-244	0.857	0.565	0.006	7.9E-240	P4
Kif20a	5.8E-239	1.228	0.645	0.01	8.5E-235	P4
Rad51ap1	5.1E-238	0.970	0.645	0.01	7.5E-234	P4
Ckap2	8.3E-235	1.540	0.726	0.015	1.2E-230	P4
Aurka	4.3E-231	1.038	0.532	0.005	6.3E-227	P4
Kif22	7.8E-227	0.976	0.581	0.008	1.1E-222	P4
Cdc20	3.9E-225	2.007	0.742	0.017	5.6E-221	P4
Shcbp1	6.8E-225	0.803	0.548	0.006	9.9E-221	P4
Cdk1	1.6E-220	2.439	0.839	0.026	2.3E-216	P4
Racgap1	2.9E-218	1.606	0.71	0.016	4.3E-214	P4
Cenpm	1.4E-217	1.756	0.823	0.025	2.0E-213	P4
Anln	1.4E-201	1.274	0.694	0.017	2.1E-197	P4
Cdkn3	1.7E-201	1.340	0.597	0.011	2.4E-197	P4
Bub1b	1.1E-199	0.761	0.532	0.008	1.6E-195	P4
Mad2l1	3.6E-190	1.129	0.823	0.03	5.2E-186	P4
Lockd	2.0E-189	2.032	0.855	0.035	2.9E-185	P4
Clspn	6.5E-185	0.862	0.548	0.01	9.5E-181	P4
Aurkb	7.3E-184	1.408	0.613	0.015	1.1E-179	P4
Rrm2	3.5E-179	1.537	0.613	0.015	5.1E-175	P4
Arhgap11a	4.0E-174	1.024	0.581	0.014	5.9E-170	P4

Diaph3	2.8E-172	1.048	0.645	0.018	4.0E-168	P4
Uhrf1	9.6E-165	1.150	0.613	0.017	1.4E-160	P4
Cip2a	1.6E-164	0.906	0.581	0.015	2.3E-160	P4
Cenpn	3.9E-153	0.712	0.532	0.013	5.7E-149	P4
Cenpw	2.8E-149	1.370	0.855	0.048	4.1E-145	P4
Stmn1	2.8E-149	3.824	0.984	0.075	4.1E-145	P4
Gmnn	1.7E-146	1.303	0.71	0.031	2.4E-142	P4
Cenpq	1.7E-146	1.141	0.742	0.034	2.5E-142	P4
Nusap1	1.3E-145	1.956	0.758	0.038	1.9E-141	P4
Cks2	2.6E-142	2.784	0.952	0.072	3.7E-138	P4
Incenp	4.0E-126	1.264	0.661	0.032	5.8E-122	P4
Fbxo5	3.4E-111	0.940	0.532	0.022	5.0E-107	P4
Cenpa	5.0E-110	2.578	0.629	0.036	7.2E-106	P4
Hells	2.4E-107	1.009	0.548	0.025	3.5E-103	P4
Dbf4	1.6E-106	1.060	0.661	0.04	2.3E-102	P4
Ncapg2	1.1E-103	0.977	0.661	0.041	1.6E-99	P4
Ncapd2	1.9E-103	0.937	0.597	0.032	2.8E-99	P4
Pmf1	5.2E-95	1.305	0.79	0.071	7.6E-91	P4
Smc2	3.9E-93	2.065	0.984	0.133	5.6E-89	P4
Nrm	1.0E-89	0.940	0.677	0.052	1.5E-85	P4
Lmnb2	1.1E-87	0.712	0.548	0.032	1.6E-83	P4
4930579G24Rik	1.2E-82	0.770	0.629	0.047	1.7E-78	P4
Tyms	3.0E-79	1.624	0.871	0.114	4.3E-75	P4
Nsd2	6.8E-73	0.717	0.565	0.042	9.9E-69	P4
Tcf19	1.7E-72	1.491	0.71	0.078	2.5E-68	P4
Mcm5	1.6E-69	1.070	0.516	0.039	2.3E-65	P4
Zfp367	1.5E-68	0.929	0.532	0.041	2.2E-64	P4
Pkmyt1	6.8E-66	0.724	0.565	0.049	9.8E-62	P4
Hmgb2	2.5E-65	3.150	1	0.26	3.6E-61	P4
Syce2	4.0E-65	0.800	0.548	0.046	5.8E-61	P4
Rrm1	5.9E-65	1.356	0.806	0.12	8.5E-61	P4
Rfc5	1.0E-62	0.872	0.677	0.077	1.4E-58	P4
Ezh2	8.6E-60	1.199	0.774	0.112	1.2E-55	P4
Lig1	1.3E-59	1.534	0.71	0.098	1.8E-55	P4
Tmpo	1.9E-57	2.025	0.968	0.251	2.7E-53	P4
H2afx	2.3E-57	2.172	0.903	0.198	3.3E-53	P4
Lmnb1	7.8E-53	1.475	0.839	0.163	1.1E-48	P4
Smc4	6.1E-52	2.138	0.887	0.219	8.8E-48	P4
Hmgn2	9.4E-49	1.394	0.855	0.177	1.4E-44	P4
Atad2	1.1E-48	1.021	0.613	0.084	1.6E-44	P4
Rfc4	1.1E-48	0.800	0.629	0.085	1.6E-44	P4
Dnajc9	8.1E-48	1.215	0.839	0.173	1.2E-43	P4
Gins2	2.6E-47	1.007	0.677	0.106	3.7E-43	P4
Pold1	3.9E-47	0.629	0.516	0.057	5.6E-43	P4
H2afz	1.5E-44	2.760	1	0.595	2.2E-40	P4

Mcm3	3.0E-44	0.967	0.565	0.076	4.4E-40	P4
Haus4	3.7E-44	0.871	0.661	0.105	5.4E-40	P4
Haus8	7.5E-44	0.641	0.516	0.062	1.1E-39	P4
Mcm7	1.3E-43	0.989	0.581	0.084	1.9E-39	P4
Bok	3.0E-43	1.012	0.629	0.099	4.4E-39	P4
Hist1h1e	3.5E-43	1.068	0.565	0.08	5.0E-39	P4
Ppil1	5.7E-43	0.990	0.79	0.165	8.3E-39	P4
Selenoh	8.1E-43	1.697	0.919	0.31	1.2E-38	P4
Dhfr	2.3E-42	0.870	0.516	0.065	3.3E-38	P4
Tuba1b	4.7E-41	2.622	1	0.928	6.9E-37	P4
Tubb5	4.3E-39	2.629	1	0.922	6.3E-35	P4
Ran	6.4E-39	1.925	1	0.821	9.3E-35	P4
Rangap1	6.6E-39	1.123	0.726	0.154	9.6E-35	P4
Rpa2	1.5E-38	1.012	0.629	0.114	2.2E-34	P4
Cdkn2c	6.2E-38	1.786	0.823	0.232	9.0E-34	P4
Usp1	1.7E-36	1.264	0.839	0.242	2.5E-32	P4
Mis12	2.8E-36	0.581	0.516	0.074	4.0E-32	P4
Mcm6	1.6E-35	1.046	0.597	0.109	2.3E-31	P4
Cks1b	6.8E-35	1.476	1	0.608	9.8E-31	P4
Dek	8.0E-35	1.903	1	0.612	1.2E-30	P4
Ncapd3	1.5E-34	0.789	0.548	0.09	2.1E-30	P4
Ptma	3.1E-34	1.523	1	0.994	4.6E-30	P4
H2afv	3.5E-34	2.007	0.984	0.801	5.0E-30	P4
Cdkn2d	4.8E-34	1.654	0.79	0.228	6.9E-30	P4
Hirip3	6.4E-34	0.641	0.516	0.08	9.2E-30	P4
Anp32b	7.0E-34	1.573	1	0.794	1.0E-29	P4
Nucks1	1.6E-33	1.749	0.968	0.637	2.3E-29	P4
Tubg1	2.0E-32	0.712	0.613	0.117	2.9E-28	P4
Rad21	3.4E-32	1.345	0.871	0.343	4.9E-28	P4
Mis18a	8.0E-32	0.667	0.548	0.097	1.2E-27	P4
Dnmt1	8.3E-32	0.800	0.581	0.11	1.2E-27	P4
Mthfd2	2.2E-31	0.712	0.548	0.096	3.2E-27	P4
Fam111a	3.0E-31	1.171	0.823	0.262	4.4E-27	P4
Hmgb1	1.4E-30	1.547	1	0.886	2.1E-26	P4
Tubb6	2.3E-30	1.869	0.968	0.71	3.3E-26	P4
Dut	3.5E-30	1.377	0.823	0.273	5.0E-26	P4
Itgb3bp	2.0E-29	0.838	0.758	0.203	2.9E-25	P4
Tubb4b	2.7E-29	1.894	0.919	0.454	3.9E-25	P4
Fxn	3.0E-29	0.575	0.581	0.11	4.4E-25	P4
Cmc2	6.8E-29	1.135	0.839	0.28	9.8E-25	P4
Lsm2	1.3E-28	1.156	0.887	0.346	1.9E-24	P4
Ccdc34	1.4E-28	1.506	0.887	0.405	2.0E-24	P4
Rfc2	2.0E-28	1.236	0.79	0.264	2.9E-24	P4
Snrpd1	2.5E-28	0.981	0.855	0.289	3.7E-24	P4
Rfc3	4.7E-28	0.768	0.516	0.098	6.8E-24	P4

Dctpp1	5.4E-28	1.243	0.839	0.311	7.8E-24	P4
Hat1	2.1E-27	0.947	0.677	0.184	3.1E-23	P4
Ssrp1	4.6E-27	1.249	0.919	0.448	6.6E-23	P4
Hjurp	1.3E-26	0.986	0.677	0.185	1.9E-22	P4
Hnrnpab	1.4E-26	1.213	0.968	0.682	2.0E-22	P4
Dtymk	1.8E-26	0.971	0.806	0.28	2.6E-22	P4
Lbr	4.9E-26	0.733	0.548	0.117	7.2E-22	P4
Anp32e	6.5E-26	1.120	0.855	0.364	9.4E-22	P4
Slfn5	7.6E-26	-1.732	0.274	0.903	1.1E-21	P4
Pcna	2.4E-25	1.894	0.774	0.311	3.5E-21	P4
Tagln2	6.5E-25	1.345	0.984	0.773	9.4E-21	P4
Cdca4	1.5E-24	0.566	0.532	0.111	2.1E-20	P4
Hint1	1.5E-24	0.877	1	0.988	2.2E-20	P4
Ywhah	1.3E-23	1.319	0.919	0.573	1.9E-19	P4
Alyref	4.0E-23	1.076	0.823	0.347	5.7E-19	P4
Rpa1	4.8E-23	0.805	0.548	0.133	6.9E-19	P4
Cox7a2	6.1E-23	0.947	1	0.966	8.8E-19	P4
Tpm4	8.0E-23	1.174	1	0.813	1.2E-18	P4
H1fx	9.5E-23	0.766	0.629	0.168	1.4E-18	P4
Calm2	1.1E-22	1.363	1	0.955	1.5E-18	P4
Tipin	1.8E-22	0.984	0.806	0.318	2.6E-18	P4
Ppia	2.1E-22	1.015	1	0.989	3.1E-18	P4
Mrpl18	2.3E-22	0.964	0.919	0.553	3.3E-18	P4
Terf1	6.8E-22	0.543	0.548	0.13	9.9E-18	P4
Banf1	8.3E-22	0.948	0.935	0.514	1.2E-17	P4
Ogn	9.1E-22	-1.748	0.806	0.954	1.3E-17	P4
Psip1	1.4E-21	1.029	0.871	0.403	2.0E-17	P4
Gemin6	1.8E-21	0.567	0.516	0.118	2.7E-17	P4
Hpf1	2.2E-21	0.941	0.726	0.273	3.2E-17	P4
Exosc8	5.9E-21	0.766	0.726	0.247	8.5E-17	P4
Nxt1	6.3E-21	0.912	0.677	0.217	9.2E-17	P4
Ranbp1	6.4E-21	1.425	0.952	0.815	9.3E-17	P4
Nap1l1	1.4E-20	1.014	0.968	0.619	2.0E-16	P4
Ncaph2	1.9E-20	0.888	0.758	0.3	2.8E-16	P4
Wee1	2.4E-20	0.803	0.726	0.243	3.5E-16	P4
Vdac1	3.9E-20	0.971	0.968	0.574	5.7E-16	P4
Nudt21	4.7E-20	0.806	0.871	0.415	6.9E-16	P4
Bub3	6.1E-20	1.006	0.839	0.416	8.9E-16	P4
Nasp	1.1E-19	0.930	0.597	0.183	1.7E-15	P4
Srsf3	1.2E-19	0.970	0.968	0.794	1.8E-15	P4
Tuba1c	1.3E-19	1.475	0.613	0.212	1.8E-15	P4
Srsf7	1.3E-19	0.917	0.871	0.475	1.9E-15	P4
Cbx5	1.8E-19	0.948	0.823	0.383	2.5E-15	P4
Acot7	2.1E-19	0.684	0.581	0.157	3.0E-15	P4
Snrpg	3.0E-19	0.669	1	0.975	4.4E-15	P4

Mrpl51	3.0E-19	0.891	0.887	0.596	4.4E-15	P4
Eri1	3.4E-19	0.578	0.532	0.136	4.9E-15	P4
Stub1	3.7E-19	0.865	1	0.742	5.3E-15	P4
Larp7	6.8E-19	0.772	0.823	0.33	9.9E-15	P4
Pcnt	8.4E-19	0.645	0.565	0.158	1.2E-14	P4
Rbbp7	9.8E-19	0.842	0.935	0.574	1.4E-14	P4
Bex3	1.5E-18	1.044	0.774	0.341	2.1E-14	P4
Ssna1	1.5E-18	0.846	0.887	0.473	2.2E-14	P4
Hmgn1	1.7E-18	0.797	1	0.966	2.4E-14	P4
Gjc1	1.8E-18	0.847	0.629	0.205	2.6E-14	P4
Cycs	2.4E-18	0.891	0.823	0.425	3.5E-14	P4
Nsmce1	2.5E-18	0.777	0.823	0.363	3.6E-14	P4
Fxyd6	3.1E-18	0.665	0.581	0.163	4.6E-14	P4
Ckap4	3.4E-18	1.086	0.952	0.853	4.9E-14	P4
Sephs1	5.4E-18	0.503	0.532	0.142	7.8E-14	P4
Dpep1	1.3E-17	-1.147	0.823	0.977	1.9E-13	P4
Fmr1	1.3E-17	0.556	0.661	0.21	1.9E-13	P4
Cep57	2.6E-17	0.592	0.565	0.167	3.8E-13	P4
Siva1	2.7E-17	1.222	0.806	0.405	4.0E-13	P4
Kpnb1	3.1E-17	0.806	0.79	0.366	4.5E-13	P4
Prdx4	3.7E-17	1.034	0.903	0.608	5.4E-13	P4
Lsm5	4.4E-17	0.835	0.887	0.478	6.3E-13	P4
Vars	9.9E-17	0.645	0.565	0.176	1.4E-12	P4
Pradc1	1.1E-16	0.579	0.548	0.166	1.6E-12	P4
Xpo1	1.4E-16	0.590	0.597	0.195	2.1E-12	P4
Pold2	1.5E-16	0.589	0.532	0.158	2.2E-12	P4
Rnase4	1.7E-16	-0.839	0.919	0.997	2.5E-12	P4
Smc6	1.8E-16	0.925	0.903	0.687	2.6E-12	P4
Rnaseh2c	2.7E-16	0.761	0.871	0.464	4.0E-12	P4
Tmx2	3.1E-16	0.505	0.581	0.18	4.4E-12	P4
Hdgf	3.5E-16	1.070	0.984	0.741	5.1E-12	P4
Tmem107	3.9E-16	0.737	0.726	0.308	5.7E-12	P4
Arpin	4.9E-16	0.555	0.532	0.157	7.1E-12	P4
Gpsm2	5.0E-16	0.723	0.597	0.202	7.2E-12	P4
Timm50	6.6E-16	0.558	0.677	0.235	9.5E-12	P4
Rnaseh2b	7.8E-16	0.592	0.581	0.184	1.1E-11	P4
Lsm6	8.4E-16	0.818	0.871	0.569	1.2E-11	P4
Jade1	9.5E-16	0.610	0.516	0.151	1.4E-11	P4
Tfdp1	1.1E-15	0.754	0.645	0.25	1.5E-11	P4
Phgdh	1.2E-15	0.736	0.565	0.19	1.7E-11	P4
Nde1	1.2E-15	0.593	0.516	0.158	1.8E-11	P4
Snrpa1	1.4E-15	0.657	0.742	0.31	2.0E-11	P4
Ddx39b	1.6E-15	0.802	0.871	0.464	2.3E-11	P4
Snrpb	1.6E-15	0.710	1	0.895	2.3E-11	P4
Orc6	2.5E-15	0.661	0.613	0.224	3.6E-11	P4

Gstm1	2.6E-15	-1.073	0.71	0.961	3.8E-11	P4
Ppm1g	2.9E-15	0.714	0.694	0.3	4.2E-11	P4
Timp2	3.5E-15	-0.958	0.984	0.998	5.1E-11	P4
Lmf2	3.8E-15	0.682	0.645	0.248	5.5E-11	P4
Nudc	4.2E-15	0.880	0.887	0.58	6.1E-11	P4
Naa50	4.5E-15	0.846	0.806	0.426	6.5E-11	P4
Uchl5	4.8E-15	0.538	0.565	0.181	7.0E-11	P4
Fabp5	5.7E-15	0.872	0.532	0.168	8.2E-11	P4
Set	8.4E-15	0.867	0.887	0.596	1.2E-10	P4
Gm1673	1.5E-14	0.765	0.677	0.296	2.1E-10	P4
Lgals1	1.7E-14	0.693	0.968	1	2.4E-10	P4
Hsp90aa1	1.7E-14	0.857	1	0.909	2.5E-10	P4
Ptges3	1.7E-14	0.717	0.919	0.584	2.5E-10	P4
Idh2	2.2E-14	0.754	0.758	0.367	3.1E-10	P4
Slc25a5	2.3E-14	1.069	0.984	0.896	3.4E-10	P4
Ube2e3	2.4E-14	0.669	0.855	0.441	3.5E-10	P4
Cbfb	2.4E-14	0.735	0.71	0.321	3.5E-10	P4
Acp1	3.3E-14	0.674	0.871	0.475	4.7E-10	P4
Dpt	3.4E-14	-0.821	0.935	0.999	4.9E-10	P4
Hspa8	4.2E-14	0.688	1	0.993	6.2E-10	P4
Itm2b	4.5E-14	-0.670	0.984	1	6.5E-10	P4
Plp2	5.8E-14	0.865	0.774	0.441	8.4E-10	P4
Pcbp2	5.8E-14	0.644	1	0.952	8.4E-10	P4
Pgp	5.8E-14	0.748	0.758	0.415	8.5E-10	P4
H3f3a	6.7E-14	0.707	1	0.996	9.8E-10	P4
Snrpe	6.9E-14	0.794	0.952	0.857	1.0E-09	P4
Gins4	7.2E-14	0.573	0.629	0.238	1.1E-09	P4
Actn4	9.8E-14	0.759	0.839	0.502	1.4E-09	P4
Snrnp40	1.0E-13	0.657	0.613	0.24	1.5E-09	P4
1810037I17Rik	1.2E-13	0.969	0.968	0.838	1.8E-09	P4
Smchd1	1.3E-13	0.542	0.726	0.3	1.9E-09	P4
Smc1a	1.5E-13	0.779	0.806	0.443	2.1E-09	P4
Cox5a	1.5E-13	0.682	0.984	0.815	2.2E-09	P4
Nsmce4a	1.7E-13	0.710	0.806	0.427	2.5E-09	P4
Cdk4	1.7E-13	0.788	0.968	0.744	2.5E-09	P4
H2-Q7	1.7E-13	0.767	0.565	0.19	2.5E-09	P4
Ctsl	1.8E-13	-0.791	0.952	0.997	2.7E-09	P4
Serping1	2.0E-13	-1.074	0.887	0.99	2.9E-09	P4
Impdh2	2.1E-13	0.659	0.887	0.582	3.1E-09	P4
Rpa3	4.0E-13	0.714	0.742	0.371	5.8E-09	P4
Anapc5	4.1E-13	0.768	0.903	0.671	6.0E-09	P4
Snrpf	4.3E-13	0.653	1	0.905	6.3E-09	P4
Ybx1	5.9E-13	0.513	1	1	8.5E-09	P4
Errfi1	6.2E-13	-1.379	0.435	0.776	9.0E-09	P4
Nubp1	7.7E-13	0.514	0.532	0.186	1.1E-08	P4

Dcn	8.3E-13	-0.743	0.968	1	1.2E-08	P4
Nfyb	8.7E-13	0.520	0.532	0.179	1.3E-08	P4
Tcof1	1.0E-12	0.543	0.581	0.212	1.5E-08	P4
Pole3	1.1E-12	0.644	0.758	0.385	1.6E-08	P4
Clec3b	1.1E-12	-0.991	0.903	0.979	1.6E-08	P4
Arpp19	1.4E-12	0.777	0.952	0.794	2.1E-08	P4
C1s1	1.8E-12	-0.769	0.887	0.978	2.6E-08	P4
Hnrnpd	2.0E-12	0.805	0.758	0.429	2.9E-08	P4
Nop56	2.0E-12	0.504	0.613	0.235	3.0E-08	P4
Srrt	2.1E-12	0.517	0.581	0.217	3.0E-08	P4
H3f3b	2.3E-12	0.967	1	0.997	3.4E-08	P4
Eny2	2.5E-12	0.779	0.903	0.688	3.7E-08	P4
Mapre1	2.9E-12	0.790	0.952	0.688	4.2E-08	P4
Emp2	3.4E-12	0.658	0.565	0.217	4.9E-08	P4
Ypel3	3.6E-12	-0.841	0.597	0.875	5.2E-08	P4
Nelfe	3.6E-12	0.598	0.581	0.226	5.3E-08	P4
Nupr1	4.2E-12	-0.915	0.742	0.963	6.0E-08	P4
Tceal9	4.9E-12	0.701	1	0.956	7.1E-08	P4
Nono	6.4E-12	0.627	0.823	0.47	9.3E-08	P4
Clic1	7.5E-12	0.721	0.952	0.922	1.1E-07	P4
Lsm8	7.9E-12	0.730	0.742	0.424	1.1E-07	P4
Htra3	7.9E-12	-0.831	0.742	0.971	1.1E-07	P4
Mzt1	8.6E-12	0.520	0.597	0.242	1.3E-07	P4
Anapc11	8.7E-12	0.744	0.903	0.782	1.3E-07	P4
Fcgrt	9.4E-12	-0.729	0.823	0.975	1.4E-07	P4
Myh9	9.4E-12	0.631	0.661	0.305	1.4E-07	P4
Cfl1	1.1E-11	0.662	1	0.97	1.6E-07	P4
Maz	1.1E-11	0.638	0.629	0.287	1.6E-07	P4
Klf4	1.4E-11	-1.172	0.774	0.965	2.1E-07	P4
Arl6ip1	1.5E-11	1.747	0.839	0.725	2.1E-07	P4
Srsf1	1.6E-11	0.625	0.677	0.336	2.4E-07	P4
Tpm3	1.6E-11	0.708	0.935	0.716	2.4E-07	P4
Stip1	1.8E-11	0.540	0.597	0.237	2.6E-07	P4
Rbbp4	2.1E-11	0.680	0.806	0.47	3.0E-07	P4
Cebpd	3.1E-11	-1.048	0.613	0.929	4.5E-07	P4
Zfp91	3.2E-11	0.661	0.823	0.501	4.6E-07	P4
Ptms	3.8E-11	0.754	0.984	0.933	5.5E-07	P4
Raly	3.9E-11	0.507	0.565	0.218	5.6E-07	P4
Lyar	4.5E-11	0.655	0.677	0.349	6.5E-07	P4
Fkbp2	5.2E-11	0.774	0.871	0.698	7.5E-07	P4
Lmna	5.4E-11	0.697	0.903	0.632	7.8E-07	P4
Eif1ad	5.5E-11	0.530	0.548	0.216	7.9E-07	P4
Rarres2	6.5E-11	-0.678	0.935	0.995	9.4E-07	P4
Map1lc3b	9.5E-11	-0.672	0.887	0.967	1.4E-06	P4
Hnrnpu	9.7E-11	0.573	0.726	0.361	1.4E-06	P4

Ak6	9.9E-11	0.501	0.581	0.242	1.4E-06	P4
Ahnak2	1.0E-10	-0.945	0.29	0.697	1.5E-06	P4
Trim28	1.4E-10	0.551	0.758	0.383	2.0E-06	P4
Hp1bp3	1.4E-10	0.741	0.758	0.463	2.0E-06	P4
Aldoa	1.4E-10	-0.708	0.758	0.956	2.1E-06	P4
Selenop	1.5E-10	-0.865	0.855	0.97	2.2E-06	P4
Eloc	1.5E-10	0.629	0.935	0.71	2.2E-06	P4
Mxd4	1.6E-10	-0.721	0.5	0.872	2.4E-06	P4
Erh	1.8E-10	0.715	0.774	0.48	2.6E-06	P4
Hnrnpr	1.8E-10	0.653	0.774	0.46	2.7E-06	P4
Ipo5	1.9E-10	0.529	0.629	0.278	2.7E-06	P4
Brd8	1.9E-10	0.537	0.597	0.25	2.7E-06	P4
Ppp1ca	2.3E-10	0.552	0.984	0.925	3.3E-06	P4
Tra2b	2.8E-10	0.634	0.919	0.725	4.0E-06	P4
Actb	2.9E-10	0.855	1	0.999	4.1E-06	P4
Cd302	2.9E-10	-0.749	0.742	0.949	4.2E-06	P4
Atpif1	2.9E-10	0.588	1	0.959	4.2E-06	P4
Ptbp1	3.0E-10	0.554	0.79	0.413	4.3E-06	P4
Cnot6l	3.2E-10	0.526	0.71	0.352	4.7E-06	P4
Mmp2	4.0E-10	-0.686	0.823	0.971	5.7E-06	P4
Mrps25	4.3E-10	0.530	0.758	0.38	6.3E-06	P4
Tcerg1	4.4E-10	0.511	0.597	0.27	6.4E-06	P4
Pih1d1	5.3E-10	0.556	0.661	0.308	7.7E-06	P4
Aarsd1	5.3E-10	0.701	0.629	0.307	7.7E-06	P4
Csrp1	5.3E-10	0.680	0.613	0.29	7.8E-06	P4
U2af1	5.7E-10	0.694	0.839	0.55	8.2E-06	P4
Cnih4	5.9E-10	0.594	0.806	0.479	8.5E-06	P4
Gkap1	6.5E-10	0.597	0.645	0.303	9.4E-06	P4
G3bp1	7.3E-10	0.754	0.823	0.543	1.1E-05	P4
Zbtb20	9.0E-10	-0.553	0.935	0.999	1.3E-05	P4
Nid1	1.0E-09	-0.652	0.952	0.997	1.4E-05	P4
Srsf2	1.2E-09	0.676	0.903	0.76	1.7E-05	P4
Gtf2a2	1.2E-09	0.587	0.855	0.54	1.8E-05	P4
Dazap1	1.2E-09	0.517	0.774	0.437	1.8E-05	P4
Nans	1.2E-09	0.575	0.677	0.364	1.8E-05	P4
Dpy30	1.3E-09	0.558	0.71	0.4	1.9E-05	P4
Psma4	1.4E-09	0.604	0.887	0.764	2.0E-05	P4
Ndufb8	1.6E-09	0.569	0.935	0.78	2.3E-05	P4
Pcolce2	1.6E-09	-1.055	0.726	0.926	2.3E-05	P4
Aspn	1.7E-09	-0.982	0.581	0.824	2.4E-05	P4
Hnrnpul1	1.7E-09	0.512	0.758	0.419	2.5E-05	P4
Actg1	1.7E-09	0.646	1	0.98	2.5E-05	P4
Tnxb	1.9E-09	-0.771	0.71	0.921	2.7E-05	P4
Crip1	1.9E-09	0.548	0.984	0.998	2.8E-05	P4
Rbm8a	2.0E-09	0.507	0.855	0.605	2.8E-05	P4

Pfn1	2.0E-09	0.539	0.984	0.985	2.9E-05	P4
Serpinf1	2.1E-09	-0.622	0.952	0.998	3.0E-05	P4
Ubald2	2.2E-09	0.697	0.661	0.355	3.2E-05	P4
Klf9	2.3E-09	-0.785	0.887	0.959	3.3E-05	P4
Cisd2	2.3E-09	0.578	0.823	0.509	3.3E-05	P4
Islr	2.3E-09	-0.792	0.71	0.916	3.4E-05	P4
Ccdc88a	2.5E-09	0.590	0.71	0.384	3.6E-05	P4
C3	2.7E-09	-1.050	0.516	0.779	3.9E-05	P4
Ciao2a	3.3E-09	0.510	0.742	0.409	4.8E-05	P4
Atp5o	3.4E-09	0.505	0.984	0.925	5.0E-05	P4
Fkbp4	3.5E-09	0.578	0.758	0.466	5.1E-05	P4
Mettl9	3.8E-09	0.566	0.855	0.575	5.6E-05	P4
Plpp3	3.9E-09	-0.871	0.839	0.99	5.7E-05	P4
Eif4e	4.2E-09	0.532	0.629	0.319	6.1E-05	P4
Cntln	4.3E-09	0.599	0.581	0.278	6.3E-05	P4
Zfp36l1	4.8E-09	-0.873	0.645	0.89	6.9E-05	P4
Prdx6	4.9E-09	-0.703	0.742	0.914	7.1E-05	P4
Mrpl14	5.1E-09	0.591	0.839	0.666	7.4E-05	P4
Cby1	6.1E-09	0.515	0.532	0.244	8.8E-05	P4
Acadl	6.2E-09	0.502	0.758	0.423	9.0E-05	P4
Fth1	6.3E-09	-0.538	1	1	9.1E-05	P4
Txnl1	6.9E-09	0.622	0.79	0.54	1.0E-04	P4
Lims1	7.0E-09	0.606	0.774	0.468	1.0E-04	P4
Gapdh	7.2E-09	0.588	1	0.931	1.0E-04	P4
Meg3	7.4E-09	-0.828	0.435	0.735	1.1E-04	P4
Inmt	7.4E-09	-1.707	0.339	0.644	1.1E-04	P4
Tomm40	9.6E-09	0.573	0.613	0.327	1.4E-04	P4
Actr3	1.0E-08	0.627	0.871	0.627	1.5E-04	P4
Scara5	1.1E-08	-0.895	0.452	0.806	1.6E-04	P4
Smarca5	1.3E-08	0.616	0.726	0.47	1.8E-04	P4
Timp3	1.4E-08	-0.795	0.677	0.901	2.0E-04	P4
Tcp1	1.4E-08	0.560	0.887	0.598	2.1E-04	P4
Txndc12	1.4E-08	0.550	0.661	0.368	2.1E-04	P4
Phf5a	1.6E-08	0.535	0.726	0.404	2.2E-04	P4
Calm3	1.7E-08	0.561	0.823	0.608	2.5E-04	P4
Ywhae	2.0E-08	0.527	0.984	0.943	2.8E-04	P4
Ppp4c	2.0E-08	0.504	0.694	0.406	2.9E-04	P4
Tpr	2.0E-08	0.552	0.903	0.684	3.0E-04	P4
Prdx5	2.2E-08	-0.635	0.839	0.937	3.2E-04	P4
Sult5a1	2.3E-08	-0.853	0.274	0.613	3.3E-04	P4
Lsm4	2.6E-08	0.589	0.855	0.623	3.8E-04	P4
Uqcr10	2.7E-08	0.506	1	0.961	3.8E-04	P4
Rfc1	2.7E-08	0.710	0.629	0.389	3.9E-04	P4
Psmc4	2.8E-08	0.543	0.742	0.47	4.1E-04	P4
Ndufa5	3.0E-08	0.505	0.952	0.859	4.3E-04	P4

Txnip	3.0E-08	-0.834	0.5	0.766	4.4E-04	P4
Prss23	3.1E-08	-0.855	0.645	0.88	4.4E-04	P4
Smc3	3.2E-08	0.546	0.742	0.463	4.6E-04	P4
Ift74	3.8E-08	0.540	0.532	0.256	5.5E-04	P4
Pi16	3.9E-08	-1.842	0.726	0.901	5.7E-04	P4
Txn1	4.1E-08	0.530	1	0.997	5.9E-04	P4
Thoc7	4.8E-08	0.562	0.839	0.66	6.9E-04	P4
Timp1	5.1E-08	1.052	0.677	0.41	7.4E-04	P4
C1ra	5.4E-08	-0.614	0.774	0.938	7.9E-04	P4
Dynlt1f	5.9E-08	0.585	0.758	0.472	8.6E-04	P4
Scaf11	6.1E-08	0.549	0.726	0.49	8.9E-04	P4
Hnrnpf	7.8E-08	0.626	0.968	0.803	1.1E-03	P4
Hprt	7.9E-08	0.548	0.694	0.403	1.2E-03	P4
Pdap1	9.4E-08	0.524	0.871	0.765	1.4E-03	P4
Paics	9.8E-08	0.512	0.726	0.452	1.4E-03	P4
Ifi205	1.0E-07	-0.794	0.887	0.967	1.5E-03	P4
Tpi1	1.0E-07	0.588	0.839	0.65	1.5E-03	P4
Gclm	1.0E-07	0.528	0.532	0.254	1.5E-03	P4
Tmem109	1.1E-07	0.581	0.645	0.373	1.6E-03	P4
Tgfbr2	1.3E-07	-0.735	0.726	0.879	1.9E-03	P4
Fndc1	1.3E-07	-0.756	0.565	0.836	1.9E-03	P4
Snu13	1.4E-07	0.592	0.79	0.527	2.0E-03	P4
Ly6a	1.4E-07	-0.810	0.919	0.975	2.0E-03	P4
Gstt1	1.6E-07	-0.645	0.452	0.773	2.3E-03	P4
Syncrip	1.7E-07	0.552	0.645	0.371	2.4E-03	P4
Sep 07	1.7E-07	0.528	0.919	0.853	2.4E-03	P4
Timm17a	1.7E-07	0.517	0.758	0.48	2.5E-03	P4
Iqgap1	1.8E-07	0.598	0.774	0.496	2.6E-03	P4
Tuba1a	1.9E-07	0.536	1	0.993	2.8E-03	P4
Uba2	2.0E-07	0.571	0.645	0.352	2.9E-03	P4
H2afy	2.2E-07	0.570	0.758	0.543	3.1E-03	P4
Aebp1	2.2E-07	-0.665	0.919	0.994	3.2E-03	P4
Sod3	2.3E-07	-0.610	0.565	0.846	3.3E-03	P4
Ywhaq	2.4E-07	0.562	0.903	0.778	3.5E-03	P4
Hspd1	2.7E-07	0.604	0.79	0.525	3.9E-03	P4
Smpd3	2.8E-07	-1.770	0.387	0.672	4.0E-03	P4
Map1lc3a	2.8E-07	-0.563	0.71	0.894	4.1E-03	P4
Ap2m1	3.0E-07	0.517	0.839	0.628	4.3E-03	P4
Atp5b	3.0E-07	0.513	1	0.968	4.4E-03	P4
Lrrn4cl	3.3E-07	-0.870	0.484	0.731	4.7E-03	P4
Gltp	3.5E-07	0.625	0.694	0.465	5.1E-03	P4
Mdh2	3.5E-07	0.511	0.935	0.73	5.1E-03	P4
Bzw1	3.7E-07	0.502	0.806	0.538	5.3E-03	P4
Vdac2	4.5E-07	0.565	0.823	0.642	6.6E-03	P4
Ackr3	4.6E-07	-0.946	0.565	0.788	6.7E-03	P4

Sdc1	4.7E-07	0.944	0.532	0.271	6.9E-03	P4
Pa2g4	5.1E-07	0.532	0.629	0.373	7.5E-03	P4
Ace	5.2E-07	-0.716	0.468	0.756	7.5E-03	P4
B230219D22Rik	5.8E-07	0.504	0.839	0.572	8.4E-03	P4
Pafah1b2	6.0E-07	0.506	0.726	0.469	8.7E-03	P4
Gpc3	6.0E-07	-0.793	0.516	0.791	8.7E-03	P4
Lrp1	6.1E-07	-0.535	0.79	0.969	8.9E-03	P4
Procr	6.1E-07	-0.914	0.419	0.703	8.9E-03	P4
Dusp1	6.9E-07	-0.888	0.839	0.922	1.0E-02	P4
Eif4h	7.4E-07	0.543	0.855	0.675	1.1E-02	P4
Cct7	7.8E-07	0.565	0.774	0.583	1.1E-02	P4
Igfbp4	7.8E-07	-0.823	0.871	0.935	1.1E-02	P4
Cd34	8.2E-07	-0.615	0.823	0.973	1.2E-02	P4
Ier2	8.8E-07	-0.842	0.887	0.944	1.3E-02	P4
Mrpl13	8.9E-07	0.522	0.758	0.477	1.3E-02	P4
Prnp	9.9E-07	-0.533	0.71	0.894	1.4E-02	P4
Jpt1	1.5E-06	0.791	0.935	0.851	2.1E-02	P4
Atp6v1e1	1.6E-06	-0.576	0.484	0.728	2.4E-02	P4
Ncl	1.7E-06	0.668	0.952	0.867	2.5E-02	P4
Cisd1	1.8E-06	0.523	0.629	0.374	2.6E-02	P4
Eif1ax	1.8E-06	0.525	0.742	0.53	2.6E-02	P4
Malat1	1.8E-06	-0.517	1	0.999	2.7E-02	P4
Mxra8	1.9E-06	-0.513	0.597	0.875	2.8E-02	P4
Pdpn	2.0E-06	-0.557	0.355	0.687	2.9E-02	P4
Nme1	2.0E-06	0.638	0.903	0.759	3.0E-02	P4
Clec2d	2.1E-06	-0.578	0.435	0.693	3.0E-02	P4
Mt1	2.6E-06	-0.865	0.968	0.978	3.7E-02	P4
Antxr1	2.9E-06	-0.566	0.581	0.806	4.2E-02	P4
Hmgn3	3.0E-06	0.571	0.855	0.776	4.4E-02	P4
Cpxm1	3.5E-06	-0.566	0.742	0.927	5.1E-02	P4
Metrnl	3.7E-06	-0.573	0.726	0.892	5.4E-02	P4
Ccl11	4.2E-06	-0.801	0.677	0.856	6.1E-02	P4
Ar	4.4E-06	-0.569	0.677	0.882	6.4E-02	P4
Emp1	5.2E-06	0.694	0.855	0.682	7.5E-02	P4
Eln	6.0E-06	-0.590	0.452	0.699	8.8E-02	P4
Igfbp6	6.1E-06	-0.695	0.855	0.993	8.8E-02	P4
Ube2k	6.3E-06	0.510	0.774	0.595	9.1E-02	P4
Col14a1	7.2E-06	-0.676	0.613	0.859	1.0E-01	P4
Axl	8.1E-06	-0.500	0.79	0.938	1.2E-01	P4
Cyc1	8.3E-06	0.543	0.71	0.53	1.2E-01	P4
Mmp23	9.6E-06	-0.515	0.661	0.865	1.4E-01	P4
Eif3a	1.2E-05	0.501	0.823	0.713	1.8E-01	P4
Tmem158	1.3E-05	-0.905	0.581	0.727	1.9E-01	P4
Ctsk	1.4E-05	-0.588	0.839	0.931	2.0E-01	P4
Sh3bgrl3	1.5E-05	0.693	0.919	0.907	2.1E-01	P4

Gsn	1.5E-05	-0.651	1	1	2.2E-01	P4
Ltbp1	1.6E-05	-0.508	0.323	0.579	2.4E-01	P4
Abca1	1.6E-05	-0.526	0.323	0.587	2.4E-01	P4
Xbp1	1.9E-05	-0.514	0.597	0.735	2.7E-01	P4
Fos	2.2E-05	-0.633	0.984	0.995	3.1E-01	P4
Glul	2.2E-05	-0.691	0.548	0.71	3.2E-01	P4
Mt2	2.9E-05	-0.907	0.726	0.875	4.2E-01	P4
Emilin2	3.0E-05	-0.510	0.661	0.873	4.4E-01	P4
Klf2	3.1E-05	-0.703	0.694	0.884	4.5E-01	P4
Cdc123	3.5E-05	0.509	0.548	0.356	5.1E-01	P4
Tmem159	3.7E-05	-0.506	0.339	0.563	5.4E-01	P4
Cdkn1c	5.0E-05	-0.859	0.468	0.676	7.2E-01	P4
Anxa3	5.5E-05	-0.807	0.661	0.842	8.0E-01	P4
Ubc	6.7E-05	-0.501	0.935	0.988	9.8E-01	P4
Mfap5	6.8E-05	-0.576	0.887	0.987	9.9E-01	P4
Zfp36	8.0E-05	-0.867	0.806	0.892	1.0E+00	P4
Sfrp4	1.0E-04	-0.798	0.468	0.715	1.0E+00	P4
Tsc22d3	1.1E-04	-0.514	0.435	0.682	1.0E+00	P4
Dbp	1.2E-04	-0.593	0.452	0.659	1.0E+00	P4
Plac8	1.8E-04	-0.839	0.887	0.939	1.0E+00	P4
Gpx3	2.0E-04	-0.802	0.806	0.916	1.0E+00	P4
Fus	3.6E-04	0.511	0.726	0.586	1.0E+00	P4
Zbtb16	3.9E-04	-0.524	0.355	0.545	1.0E+00	P4
Igfbp5	4.7E-04	-0.938	0.645	0.793	1.0E+00	P4
Nfkbiz	5.1E-04	-0.529	0.371	0.585	1.0E+00	P4
Lbp	6.5E-04	-0.542	0.371	0.55	1.0E+00	P4
Ifi207	7.2E-04	-0.555	0.548	0.653	1.0E+00	P4
Junb	7.5E-04	-0.561	0.935	0.978	1.0E+00	P4
Fabp4	7.6E-04	1.425	0.645	0.532	1.0E+00	P4
Egr1	8.6E-04	-0.534	0.935	0.98	1.0E+00	P4
Pla1a	9.3E-04	-0.616	0.645	0.804	1.0E+00	P4
Phlda3	1.3E-03	0.501	0.532	0.363	1.0E+00	P4
Atf3	2.0E-03	-0.710	0.629	0.728	1.0E+00	P4
Btg2	2.3E-03	-0.541	0.855	0.899	1.0E+00	P4
Crip2	2.5E-03	0.646	0.516	0.376	1.0E+00	P4
Lox	4.1E-03	-0.505	0.339	0.503	1.0E+00	P4
Il1r2	5.2E-03	-0.769	0.419	0.526	1.0E+00	P4
Ccn1	5.8E-03	-0.777	0.758	0.8	1.0E+00	P4

Extended data Table 4

Maker genes for human preadipocytes clusters from human deep neck adipose tissue

	p_val	avg_logFC	pct.1	pct.2	p_val_adj	cluster
ABCA10	2.37E-139	1.643247	0.95	0.691	4.43E-135	H-cluster1
COL15A1	3.27E-128	2.202305	0.75	0.101	6.12E-124	H-cluster1
GHR	2.94E-110	1.62349	0.842	0.404	5.51E-106	H-cluster1
FMO2	2.86E-106	1.934106	0.628	0.059	5.34E-102	H-cluster1
ABCA6	3.91E-84	1.064452	0.899	0.718	7.31E-80	H-cluster1
LIFR	1.70E-75	1.559298	0.645	0.212	3.18E-71	H-cluster1
ABCA9	3.86E-71	0.916248	0.94	0.817	7.23E-67	H-cluster1
COL4A2	1.97E-70	1.343027	0.575	0.129	3.69E-66	H-cluster1
PREX2	5.65E-68	1.333222	0.496	0.07	1.06E-63	H-cluster1
FGF10	9.68E-68	1.511671	0.504	0.076	1.81E-63	H-cluster1
BMPER	3.63E-67	1.465924	0.579	0.147	6.80E-63	H-cluster1
ABCA8	9.88E-66	0.992795	0.876	0.665	1.85E-61	H-cluster1
KAZN	3.59E-65	1.158915	0.871	0.71	6.71E-61	H-cluster1
TCF4	1.43E-64	0.985928	0.845	0.66	2.68E-60	H-cluster1
NAV2	7.55E-64	1.585897	0.547	0.136	1.41E-59	H-cluster1
MAML2	7.16E-61	1.094785	0.789	0.463	1.34E-56	H-cluster1
COL4A1	2.98E-60	1.257958	0.513	0.115	5.57E-56	H-cluster1
ARHGAP24	1.06E-58	1.042559	0.816	0.557	1.98E-54	H-cluster1
LHFPL6	9.55E-58	0.921128	0.846	0.591	1.79E-53	H-cluster1
SPECC1	1.30E-56	1.321465	0.424	0.059	2.44E-52	H-cluster1
LDB2	4.86E-54	1.078989	0.73	0.412	9.09E-50	H-cluster1
SLC9A9	2.06E-51	0.919904	0.776	0.49	3.85E-47	H-cluster1
ABCB11	4.46E-51	1.584358	0.328	0.014	8.34E-47	H-cluster1
PID1	1.61E-50	0.84469	0.836	0.629	3.01E-46	H-cluster1
RORA	1.96E-50	0.563972	0.964	0.917	3.67E-46	H-cluster1
KCND2	4.81E-48	0.997406	0.708	0.332	9.01E-44	H-cluster1
PPARG	1.91E-45	1.076946	0.514	0.172	3.58E-41	H-cluster1
CAMK2D	5.94E-45	0.877364	0.754	0.513	1.11E-40	H-cluster1
ACACB	6.60E-43	1.124224	0.629	0.337	1.23E-38	H-cluster1
TRHDE	7.24E-43	1.342242	0.352	0.056	1.35E-38	H-cluster1
BOC	9.23E-42	0.992619	0.559	0.237	1.73E-37	H-cluster1
PKD1L2	2.67E-40	1.128601	0.402	0.098	4.99E-36	H-cluster1
NRP1	2.77E-40	0.983764	0.655	0.382	5.19E-36	H-cluster1
PRKG1	5.73E-40	0.913944	0.721	0.424	1.07E-35	H-cluster1
TBC1D4	1.04E-39	0.959357	0.614	0.317	1.94E-35	H-cluster1
PLD1	1.00E-38	0.882197	0.659	0.374	1.88E-34	H-cluster1
RBPMS	1.06E-38	0.85745	0.741	0.529	1.99E-34	H-cluster1
IGF1	4.23E-38	0.927717	0.487	0.17	7.91E-34	H-cluster1
MME	9.59E-38	1.137304	0.398	0.108	1.79E-33	H-cluster1
ANGPT1	3.08E-37	1.070399	0.397	0.103	5.76E-33	H-cluster1
APOD	1.23E-35	0.604412	0.489	0.161	2.31E-31	H-cluster1
MAPK10	1.63E-35	0.937859	0.645	0.404	3.05E-31	H-cluster1
PRICKLE2	4.71E-35	0.881758	0.537	0.256	8.80E-31	H-cluster1

PLPP3	3.72E-34	1.108988	0.552	0.29	6.95E-30	H-cluster1
FAM13A	4.17E-34	0.800011	0.661	0.426	7.79E-30	H-cluster1
SLIT3	7.60E-34	0.694583	0.694	0.396	1.42E-29	H-cluster1
RUNX1T1	1.31E-33	0.70427	0.772	0.593	2.45E-29	H-cluster1
PRICKLE1	1.98E-33	0.793651	0.598	0.307	3.71E-29	H-cluster1
PQLC2L	2.03E-33	0.836948	0.329	0.07	3.79E-29	H-cluster1
IMMP2L	4.90E-33	0.717803	0.793	0.619	9.17E-29	H-cluster1
ACVR2A	7.41E-33	0.858494	0.568	0.287	1.39E-28	H-cluster1
DPP6	9.43E-33	1.128358	0.307	0.064	1.76E-28	H-cluster1
LAMA2	3.02E-32	0.614804	0.92	0.852	5.66E-28	H-cluster1
SUSD1	1.06E-31	0.820394	0.296	0.055	1.98E-27	H-cluster1
PTEN	1.47E-31	0.842656	0.678	0.499	2.75E-27	H-cluster1
DPYD	2.29E-31	0.593506	0.806	0.666	4.28E-27	H-cluster1
MYCBP2	2.59E-31	0.670875	0.761	0.622	4.84E-27	H-cluster1
ADH1B	8.74E-31	0.878248	0.598	0.328	1.63E-26	H-cluster1
RIPOR3	1.94E-29	0.781834	0.487	0.22	3.63E-25	H-cluster1
EPSTI1	4.98E-29	0.777846	0.279	0.053	9.32E-25	H-cluster1
HMGCLL1	1.14E-28	0.8719	0.269	0.048	2.13E-24	H-cluster1
ABCA5	1.51E-28	0.767265	0.441	0.184	2.82E-24	H-cluster1
GPC3	1.59E-28	0.840736	0.368	0.109	2.97E-24	H-cluster1
MOB3B	7.45E-28	0.859013	0.319	0.087	1.39E-23	H-cluster1
ITIH5	5.47E-27	0.808348	0.52	0.279	1.02E-22	H-cluster1
ENPEP	5.39E-25	0.731324	0.28	0.07	1.01E-20	H-cluster1
BCL6	2.25E-24	0.681831	0.434	0.193	4.20E-20	H-cluster1
CYP7B1	2.52E-24	0.9478	0.438	0.203	4.72E-20	H-cluster1
LAMC1	2.56E-24	0.623498	0.754	0.647	4.79E-20	H-cluster1
ANTXR2	4.71E-24	0.68607	0.657	0.477	8.81E-20	H-cluster1
MYOC	5.90E-24	0.55625	0.256	0.055	1.10E-19	H-cluster1
RHOBTB3	1.05E-23	0.709721	0.631	0.465	1.96E-19	H-cluster1
AMPH	2.14E-23	0.855766	0.381	0.168	4.00E-19	H-cluster1
LMO3	3.00E-23	0.75894	0.478	0.245	5.61E-19	H-cluster1
CXCL14	7.42E-23	0.511535	0.29	0.073	1.39E-18	H-cluster1
PCDH9	1.15E-22	0.880386	0.542	0.32	2.15E-18	H-cluster1
SRPX	2.08E-22	0.728082	0.295	0.089	3.88E-18	H-cluster1
PTPRS	4.54E-22	0.613789	0.704	0.565	8.49E-18	H-cluster1
TTC39C	1.07E-21	0.727581	0.399	0.189	2.00E-17	H-cluster1
SASH1	2.11E-21	0.589187	0.678	0.512	3.95E-17	H-cluster1
REV3L	2.71E-21	0.60029	0.72	0.596	5.06E-17	H-cluster1
EYA1	2.96E-21	0.698032	0.284	0.086	5.53E-17	H-cluster1
MITF	6.58E-21	0.685421	0.467	0.251	1.23E-16	H-cluster1
MAP3K5	6.64E-21	0.799572	0.397	0.184	1.24E-16	H-cluster1
MAN1A1	9.05E-21	0.6577	0.596	0.417	1.69E-16	H-cluster1
PDE1A	7.08E-20	0.705232	0.318	0.12	1.32E-15	H-cluster1
FAM129A	1.06E-19	0.671743	0.414	0.211	1.98E-15	H-cluster1
FGF7	2.60E-19	0.651889	0.547	0.353	4.86E-15	H-cluster1
TLN2	2.69E-19	0.598368	0.634	0.459	5.03E-15	H-cluster1
SVEP1	3.35E-19	0.556097	0.698	0.569	6.27E-15	H-cluster1

DHRS3	5.49E-19	0.520727	0.537	0.314	1.03E-14	H-cluster1
ADAMTS12	1.03E-18	0.6636	0.335	0.136	1.92E-14	H-cluster1
MYO1D	1.23E-18	0.842995	0.283	0.105	2.30E-14	H-cluster1
ECHDC2	3.38E-18	0.605507	0.58	0.431	6.33E-14	H-cluster1
CDH11	4.04E-18	0.494225	0.306	0.114	7.56E-14	H-cluster1
AGTR1	5.42E-18	0.726815	0.365	0.179	1.01E-13	H-cluster1
LMOD1	7.91E-17	0.581683	0.328	0.144	1.48E-12	H-cluster1
LAMA4	8.02E-17	0.490679	0.741	0.655	1.50E-12	H-cluster1
NFIB	1.06E-16	0.372277	0.851	0.803	1.97E-12	H-cluster1
NR3C1	1.46E-16	0.493006	0.688	0.576	2.73E-12	H-cluster1
CRYBG3	1.96E-16	0.667835	0.412	0.239	3.66E-12	H-cluster1
GLIS3	2.80E-16	0.514253	0.31	0.134	5.23E-12	H-cluster1
TMEM135	5.38E-16	0.512147	0.652	0.529	1.01E-11	H-cluster1
SBF2	5.71E-16	0.399445	0.796	0.736	1.07E-11	H-cluster1
FAM49A	6.24E-16	0.56281	0.303	0.133	1.17E-11	H-cluster1
RFTN1	1.85E-15	0.561877	0.588	0.437	3.46E-11	H-cluster1
DEPTOR	1.94E-15	0.688744	0.29	0.125	3.63E-11	H-cluster1
FAM214A	1.94E-15	0.595577	0.539	0.396	3.63E-11	H-cluster1
CLSTN2	2.54E-15	0.819472	0.261	0.103	4.76E-11	H-cluster1
LRFN5	4.43E-15	0.727842	0.414	0.24	8.29E-11	H-cluster1
ENOSF1	6.89E-15	0.548989	0.455	0.282	1.29E-10	H-cluster1
PNPLA7	8.29E-15	0.590133	0.284	0.126	1.55E-10	H-cluster1
DLC1	1.02E-14	0.337211	0.925	0.899	1.91E-10	H-cluster1
CHL1	1.30E-14	0.6718	0.54	0.398	2.43E-10	H-cluster1
MBNL1	1.65E-14	0.376817	0.839	0.789	3.09E-10	H-cluster1
CXCL12	2.49E-14	0.474314	0.33	0.165	4.67E-10	H-cluster1
COL5A3	3.86E-14	0.651123	0.261	0.112	7.21E-10	H-cluster1
COLEC12	4.18E-14	0.514093	0.56	0.395	7.82E-10	H-cluster1
BCL2	5.98E-14	0.528702	0.489	0.312	1.12E-09	H-cluster1
FOXP2	7.00E-14	0.450371	0.72	0.641	1.31E-09	H-cluster1
PNISR	9.14E-14	0.390234	0.717	0.635	1.71E-09	H-cluster1
RNF144A	2.08E-13	0.514931	0.259	0.109	3.88E-09	H-cluster1
SPATA6	2.45E-13	0.492212	0.602	0.487	4.58E-09	H-cluster1
C3	5.03E-13	0.318663	0.532	0.332	9.41E-09	H-cluster1
PARD3	5.19E-13	0.363604	0.777	0.735	9.71E-09	H-cluster1
PRKN	8.94E-13	0.485406	0.591	0.44	1.67E-08	H-cluster1
LYPLAL1	2.28E-12	0.530267	0.415	0.261	4.26E-08	H-cluster1
AUH	2.78E-12	0.522199	0.464	0.334	5.20E-08	H-cluster1
FOXN3	2.86E-12	0.327468	0.792	0.725	5.35E-08	H-cluster1
EDA	3.13E-12	0.663912	0.409	0.259	5.85E-08	H-cluster1
DMD	3.39E-12	0.688766	0.444	0.301	6.34E-08	H-cluster1
SNED1	6.45E-12	0.544235	0.499	0.362	1.21E-07	H-cluster1
PODN	6.61E-12	0.561282	0.468	0.342	1.24E-07	H-cluster1
BICC1	1.52E-11	0.304343	0.792	0.721	2.84E-07	H-cluster1
PTBP3	1.79E-11	0.557053	0.415	0.284	3.35E-07	H-cluster1
IGFBP7	1.84E-11	0.450324	0.379	0.225	3.45E-07	H-cluster1
IL16	2.01E-11	0.450791	0.319	0.172	3.77E-07	H-cluster1

WASF3	3.00E-11	0.431251	0.259	0.125	5.61E-07	H-cluster1
TCF7L1	3.01E-11	0.480421	0.585	0.491	5.64E-07	H-cluster1
EBF3	6.13E-11	0.391346	0.621	0.504	1.15E-06	H-cluster1
MAP4K3	6.19E-11	0.414527	0.524	0.398	1.16E-06	H-cluster1
RHOJ	6.83E-11	0.469802	0.293	0.154	1.28E-06	H-cluster1
TMEM132C	7.24E-11	0.43255	0.412	0.261	1.35E-06	H-cluster1
PIK3R1	7.36E-11	0.546315	0.516	0.398	1.38E-06	H-cluster1
FRMD6	7.71E-11	0.488825	0.565	0.459	1.44E-06	H-cluster1
COL6A3	8.06E-11	0.329383	0.657	0.534	1.51E-06	H-cluster1
NRIP1	8.79E-11	0.432894	0.516	0.399	1.64E-06	H-cluster1
ARHGAP44	8.98E-11	0.519556	0.256	0.125	1.68E-06	H-cluster1
SAMHD1	1.18E-10	0.530071	0.379	0.243	2.20E-06	H-cluster1
RNF24	1.77E-10	0.42901	0.257	0.125	3.32E-06	H-cluster1
COL5A2	2.00E-10	0.322515	0.659	0.552	3.73E-06	H-cluster1
PGM5	3.35E-10	0.537099	0.264	0.136	6.27E-06	H-cluster1
MACROD2	4.42E-10	0.655961	0.257	0.134	8.27E-06	H-cluster1
EGFR	4.61E-10	0.321606	0.754	0.713	8.61E-06	H-cluster1
DIAPH2	6.46E-10	0.284327	0.734	0.665	1.21E-05	H-cluster1
SPRED1	7.11E-10	0.488775	0.448	0.335	1.33E-05	H-cluster1
ZNF385D	8.92E-10	0.669853	0.487	0.376	1.67E-05	H-cluster1
HELZ	9.31E-10	0.439457	0.553	0.448	1.74E-05	H-cluster1
ANKRD28	1.80E-09	0.474493	0.447	0.328	3.37E-05	H-cluster1
SLC25A13	1.97E-09	0.479305	0.422	0.3	3.68E-05	H-cluster1
CCDC102B	2.09E-09	0.787183	0.263	0.14	3.91E-05	H-cluster1
LPAR1	2.63E-09	0.31427	0.68	0.579	4.93E-05	H-cluster1
CALD1	3.13E-09	0.310969	0.736	0.672	5.85E-05	H-cluster1
PLA2R1	4.03E-09	0.382864	0.289	0.164	7.53E-05	H-cluster1
ASPH	5.07E-09	0.356626	0.631	0.546	9.48E-05	H-cluster1
PCED1B	5.72E-09	0.531688	0.372	0.25	0.000107	H-cluster1
AC119674.2	5.88E-09	0.553879	0.421	0.301	0.00011	H-cluster1
DYNC2H1	6.86E-09	0.44018	0.56	0.482	0.000128	H-cluster1
SGCD	1.57E-08	0.403573	0.477	0.362	0.000294	H-cluster1
GPHN	1.62E-08	0.358204	0.625	0.548	0.000304	H-cluster1
MAML3	1.73E-08	0.512021	0.273	0.158	0.000323	H-cluster1
CDON	2.11E-08	0.397459	0.552	0.448	0.000395	H-cluster1
MICU3	2.29E-08	0.411881	0.421	0.31	0.000429	H-cluster1
SSBP2	2.88E-08	0.297282	0.737	0.677	0.000538	H-cluster1
SLC27A1	3.17E-08	0.420753	0.284	0.173	0.000592	H-cluster1
PLSCR4	6.29E-08	0.370975	0.542	0.465	0.001176	H-cluster1
MT-ND3	6.75E-08	0.295599	0.976	0.927	0.001262	H-cluster1
TRAK2	7.60E-08	0.391271	0.371	0.262	0.001422	H-cluster1
ZDHHC14	7.99E-08	0.482374	0.277	0.165	0.001494	H-cluster1
MIPOL1	8.18E-08	0.400878	0.397	0.279	0.00153	H-cluster1
FREM1	8.92E-08	0.399773	0.32	0.198	0.001668	H-cluster1
NR3C2	8.93E-08	0.398597	0.491	0.378	0.00167	H-cluster1
MAP3K20	9.72E-08	0.455601	0.46	0.363	0.001817	H-cluster1
ARHGAP10	1.15E-07	0.3421	0.672	0.638	0.002145	H-cluster1

GAB1	1.36E-07	0.445885	0.467	0.379	0.002553	H-cluster1
INSR	1.37E-07	0.34476	0.534	0.441	0.002564	H-cluster1
PHIP	1.39E-07	0.401372	0.52	0.424	0.002608	H-cluster1
CCND3	1.43E-07	0.529887	0.29	0.187	0.00268	H-cluster1
SETBP1	1.52E-07	0.378853	0.614	0.518	0.002845	H-cluster1
NCOA1	1.56E-07	0.290943	0.753	0.724	0.002921	H-cluster1
ANO6	1.91E-07	0.373484	0.526	0.446	0.003571	H-cluster1
FGD4	2.46E-07	0.440181	0.371	0.267	0.004599	H-cluster1
CFH	3.19E-07	0.305536	0.477	0.354	0.00596	H-cluster1
DAAM1	3.26E-07	0.483448	0.401	0.304	0.006101	H-cluster1
OGT	3.44E-07	0.337711	0.586	0.523	0.006427	H-cluster1
PITPNC1	3.85E-07	0.381557	0.468	0.362	0.007193	H-cluster1
GULP1	4.27E-07	0.362593	0.562	0.466	0.007989	H-cluster1
NDUFAF6	6.13E-07	0.434323	0.259	0.161	0.01147	H-cluster1
GPATCH8	6.47E-07	0.294678	0.622	0.548	0.0121	H-cluster1
SKAP2	6.66E-07	0.365256	0.534	0.454	0.012449	H-cluster1
HERC1	7.10E-07	0.350549	0.615	0.548	0.013277	H-cluster1
PDZRN3	7.72E-07	0.335472	0.522	0.417	0.014443	H-cluster1
CDK14	1.91E-06	0.320999	0.579	0.515	0.035759	H-cluster1
USP33	2.14E-06	0.354057	0.527	0.46	0.040078	H-cluster1
GSE1	2.17E-06	0.412469	0.379	0.278	0.040615	H-cluster1
PRRX1	2.76E-06	0.275564	0.701	0.674	0.051559	H-cluster1
MBTD1	3.26E-06	0.350943	0.411	0.318	0.060951	H-cluster1
HNMT	3.36E-06	0.308771	0.312	0.218	0.062864	H-cluster1
TNRC6A	3.52E-06	0.274061	0.71	0.693	0.065753	H-cluster1
RBPJ	3.85E-06	0.361482	0.615	0.562	0.071949	H-cluster1
ANKRD36	4.16E-06	0.315635	0.513	0.429	0.077869	H-cluster1
NTRK2	4.26E-06	0.394958	0.29	0.197	0.079659	H-cluster1
GAREM1	4.32E-06	0.408476	0.259	0.17	0.080744	H-cluster1
ARHGAP6	4.79E-06	0.375637	0.427	0.339	0.089539	H-cluster1
CC2D2A	5.04E-06	0.426797	0.259	0.173	0.094262	H-cluster1
EYA2	5.40E-06	0.326274	0.358	0.256	0.100916	H-cluster1
DLG2	5.61E-06	0.321807	0.68	0.632	0.104852	H-cluster1
PXK	6.89E-06	0.399685	0.326	0.24	0.128896	H-cluster1
CAPS2	7.42E-06	0.373738	0.322	0.229	0.138723	H-cluster1
ZFHX4	8.00E-06	0.384368	0.451	0.37	0.149686	H-cluster1
PTBP2	8.11E-06	0.331174	0.552	0.493	0.151752	H-cluster1
ITPR2	8.22E-06	0.37196	0.391	0.307	0.153744	H-cluster1
ETFDH	8.50E-06	0.356066	0.279	0.187	0.159048	H-cluster1
ST8SIA1	1.13E-05	0.46778	0.279	0.197	0.21077	H-cluster1
ACSS3	1.30E-05	0.320273	0.376	0.284	0.2427	H-cluster1
NPEPPS	1.34E-05	0.271873	0.589	0.523	0.250095	H-cluster1
RAD51B	1.52E-05	0.408043	0.507	0.418	0.284296	H-cluster1
TRPS1	1.53E-05	0.397984	0.372	0.293	0.286294	H-cluster1
SCP2	1.56E-05	0.392374	0.427	0.356	0.290874	H-cluster1
SNX29	1.58E-05	0.308358	0.568	0.524	0.29571	H-cluster1
SYNE3	2.17E-05	0.344847	0.362	0.281	0.404959	H-cluster1

ARID5B	2.21E-05	0.270804	0.408	0.317	0.413702	H-cluster1
SH3KBP1	2.24E-05	0.415515	0.306	0.225	0.418301	H-cluster1
NT5C2	2.89E-05	0.299992	0.511	0.443	0.54092	H-cluster1
PAN3	2.90E-05	0.2918	0.601	0.554	0.543032	H-cluster1
ZRANB2	3.25E-05	0.312951	0.507	0.449	0.606978	H-cluster1
ATP11C	3.48E-05	0.372798	0.28	0.197	0.650784	H-cluster1
ARHGAP26	3.65E-05	0.357985	0.408	0.326	0.683381	H-cluster1
TPCN1	4.12E-05	0.373598	0.369	0.293	0.770639	H-cluster1
ZNF292	4.16E-05	0.288618	0.536	0.482	0.778039	H-cluster1
PTPRK	4.16E-05	0.475497	0.29	0.214	0.778143	H-cluster1
MPZL1	4.41E-05	0.385585	0.428	0.365	0.824799	H-cluster1
JAK1	4.53E-05	0.337392	0.474	0.401	0.84694	H-cluster1
VWA8	4.82E-05	0.318098	0.384	0.307	0.902113	H-cluster1
ALDH1A1	5.19E-05	0.332404	0.276	0.197	0.970674	H-cluster1
HSDL2	5.71E-05	0.354672	0.266	0.189	1	H-cluster1
SGCE	6.33E-05	0.353655	0.362	0.285	1	H-cluster1
XPO1	6.75E-05	0.379757	0.421	0.365	1	H-cluster1
MAGI2	6.91E-05	0.265145	0.631	0.573	1	H-cluster1
GPX3	7.13E-05	0.395181	0.407	0.332	1	H-cluster1
DDI2	7.34E-05	0.299448	0.272	0.19	1	H-cluster1
KIAA1217	7.52E-05	0.314587	0.414	0.343	1	H-cluster1
ZNF680	8.27E-05	0.255239	0.28	0.2	1	H-cluster1
CLK4	8.30E-05	0.322144	0.3	0.222	1	H-cluster1
PLCL1	9.47E-05	0.320796	0.335	0.246	1	H-cluster1
WWP1	0.000106	0.328202	0.399	0.332	1	H-cluster1
HIBADH	0.000114	0.356485	0.402	0.331	1	H-cluster1
OSBPL1A	0.000148	0.340082	0.325	0.254	1	H-cluster1
MDM4	0.000158	0.298338	0.491	0.431	1	H-cluster1
KLHL24	0.000158	0.291178	0.392	0.328	1	H-cluster1
PDK4	0.00016	0.398799	0.399	0.337	1	H-cluster1
POU2F1	0.000173	0.279153	0.516	0.465	1	H-cluster1
TC2N	0.000199	0.331159	0.428	0.362	1	H-cluster1
TBL1XR1	0.000203	0.266267	0.609	0.571	1	H-cluster1
HSD17B4	0.000219	0.302506	0.272	0.198	1	H-cluster1
CFLAR	0.000251	0.274874	0.536	0.49	1	H-cluster1
MICU1	0.000255	0.262326	0.391	0.317	1	H-cluster1
EPS8	0.00026	0.337172	0.414	0.346	1	H-cluster1
PATJ	0.000279	0.303832	0.286	0.215	1	H-cluster1
ANKS1B	0.000307	0.286226	0.371	0.296	1	H-cluster1
ETFA	0.000359	0.291977	0.341	0.273	1	H-cluster1
MEIS2	0.000411	0.351261	0.522	0.477	1	H-cluster1
GABBR1	0.000426	0.32385	0.253	0.187	1	H-cluster1
MTURN	0.000428	0.253698	0.251	0.183	1	H-cluster1
UBE2H	0.000533	0.288188	0.532	0.479	1	H-cluster1
PDCD4	0.000673	0.304844	0.352	0.292	1	H-cluster1
SCAPER	0.000704	0.251652	0.463	0.404	1	H-cluster1
DIP2B	0.000706	0.298489	0.412	0.356	1	H-cluster1

ATG7	0.000788	0.290326	0.503	0.463	1	H-cluster1
MBOAT1	0.000797	0.256832	0.26	0.193	1	H-cluster1
ATF7IP	0.0008	0.293212	0.438	0.39	1	H-cluster1
RBM26	0.000819	0.261813	0.511	0.476	1	H-cluster1
ADGRL2	0.001242	0.274217	0.333	0.273	1	H-cluster1
ANAPC5	0.001287	0.272193	0.435	0.399	1	H-cluster1
FKBP5	0.001301	0.369254	0.289	0.229	1	H-cluster1
PUM3	0.001515	0.302899	0.315	0.257	1	H-cluster1
OSBPL8	0.001528	0.334583	0.412	0.378	1	H-cluster1
MEIS1	0.002294	0.279531	0.473	0.448	1	H-cluster1
DSE	0.002312	0.255959	0.466	0.42	1	H-cluster1
GPD2	0.002396	0.295935	0.323	0.267	1	H-cluster1
LRBA	0.002478	0.254946	0.565	0.538	1	H-cluster1
NSD3	0.002494	0.288781	0.468	0.438	1	H-cluster1
SLC25A27	0.0026	0.25962	0.287	0.232	1	H-cluster1
SSH2	0.002691	0.272413	0.608	0.598	1	H-cluster1
JARID2	0.002801	0.266436	0.323	0.27	1	H-cluster1
KIAA1328	0.002816	0.252294	0.46	0.412	1	H-cluster1
RFTN2	0.002899	0.281307	0.299	0.243	1	H-cluster1
NUBPL	0.003072	0.326181	0.264	0.211	1	H-cluster1
OXR1	0.003397	0.297535	0.428	0.392	1	H-cluster1
SUPT3H	0.003755	0.272824	0.322	0.265	1	H-cluster1
JAK2	0.004162	0.303624	0.276	0.229	1	H-cluster1
GARNL3	0.004502	0.264579	0.267	0.212	1	H-cluster1
INVS	0.005119	0.26761	0.458	0.423	1	H-cluster1
PHTF2	0.005359	0.272971	0.297	0.253	1	H-cluster1
DENND1B	0.00604	0.274079	0.266	0.217	1	H-cluster1
PCCA	0.006091	0.269192	0.509	0.493	1	H-cluster1
PER3	0.006481	0.288803	0.279	0.226	1	H-cluster1
TRANK1	0.006522	0.28644	0.279	0.236	1	H-cluster1
KIF16B	0.00732	0.265636	0.259	0.212	1	H-cluster1
ZNF248	0.007514	0.26757	0.269	0.22	1	H-cluster1
KCTD3	0.007938	0.270874	0.339	0.295	1	H-cluster1
CCDC91	0.008518	0.273704	0.341	0.301	1	H-cluster1
CNTLN	0.008672	0.261391	0.451	0.427	1	H-cluster1
PUM1	0.009284	0.273682	0.517	0.518	1	H-cluster1
MAGI1	0.009294	0.377799	0.267	0.223	1	H-cluster1
HP1BP3	0.009576	0.257632	0.489	0.477	1	H-cluster1
SEMA3C	2.01E-115	1.5404	0.963	0.415	3.76E-111	H-cluster2
FBN1	7.71E-79	0.972516	0.971	0.766	1.44E-74	H-cluster2
PLXDC2	3.86E-67	1.074875	0.947	0.752	7.21E-63	H-cluster2
CREB5	3.99E-67	1.064965	0.841	0.365	7.46E-63	H-cluster2
SDK1	9.34E-67	1.117885	0.91	0.623	1.75E-62	H-cluster2
DOCK4	2.28E-65	1.006396	0.92	0.758	4.26E-61	H-cluster2
KCNAB1	2.84E-63	1.374879	0.66	0.195	5.31E-59	H-cluster2
KCNB2	4.00E-61	1.391225	0.634	0.195	7.48E-57	H-cluster2
FRMD4A	1.37E-60	0.978033	0.841	0.488	2.56E-56	H-cluster2

PLCB1	1.29E-58	0.965614	0.851	0.445	2.41E-54	H-cluster2
SMURF2	1.15E-54	1.039717	0.83	0.472	2.16E-50	H-cluster2
STK32B	2.36E-53	1.144832	0.637	0.224	4.41E-49	H-cluster2
TBC1D12	1.99E-52	0.982028	0.692	0.305	3.71E-48	H-cluster2
DST	2.24E-52	0.724279	0.891	0.724	4.19E-48	H-cluster2
TRIO	5.09E-51	0.881554	0.881	0.654	9.52E-47	H-cluster2
STON2	3.41E-50	1.050869	0.607	0.212	6.37E-46	H-cluster2
ADAMTS6	1.54E-46	1.22838	0.485	0.13	2.89E-42	H-cluster2
HMCN1	3.12E-44	1.114794	0.626	0.269	5.84E-40	H-cluster2
SLC4A4	1.85E-39	0.911635	0.504	0.166	3.46E-35	H-cluster2
MFAP5	1.34E-37	0.518518	0.753	0.333	2.51E-33	H-cluster2
HUNK	2.73E-36	0.831972	0.451	0.138	5.10E-32	H-cluster2
KIRREL3	1.57E-35	1.150597	0.345	0.078	2.93E-31	H-cluster2
TMTC1	2.62E-35	0.909875	0.621	0.282	4.91E-31	H-cluster2
CD55	3.03E-35	0.610085	0.663	0.294	5.67E-31	H-cluster2
CAMK1D	3.54E-35	0.875224	0.698	0.377	6.62E-31	H-cluster2
NTM	9.80E-35	0.820942	0.666	0.332	1.83E-30	H-cluster2
NHSL1	2.05E-34	0.736638	0.655	0.323	3.84E-30	H-cluster2
TGFBR3	3.15E-34	0.859013	0.801	0.577	5.90E-30	H-cluster2
ARHGEF3	1.17E-33	0.838464	0.663	0.371	2.19E-29	H-cluster2
ZEB1	1.25E-33	0.588028	0.939	0.826	2.34E-29	H-cluster2
DPP4	6.57E-33	0.779406	0.329	0.075	1.23E-28	H-cluster2
HMCN2	1.89E-32	0.95172	0.469	0.174	3.54E-28	H-cluster2
PXDNL	3.49E-32	0.848979	0.531	0.224	6.53E-28	H-cluster2
FHOD3	4.52E-32	0.823978	0.557	0.234	8.45E-28	H-cluster2
PHLPP1	5.98E-32	0.82328	0.65	0.348	1.12E-27	H-cluster2
PRG4	1.33E-30	0.487643	0.414	0.121	2.49E-26	H-cluster2
AGAP1	1.95E-30	0.702316	0.772	0.535	3.64E-26	H-cluster2
DPYSL3	3.82E-30	0.967076	0.618	0.339	7.14E-26	H-cluster2
ITGA11	6.28E-30	0.727625	0.623	0.317	1.17E-25	H-cluster2
CNTN4	4.48E-29	0.766051	0.674	0.373	8.38E-25	H-cluster2
EHBP1	2.14E-28	0.627443	0.838	0.643	4.01E-24	H-cluster2
PDGFD	3.81E-28	0.769802	0.658	0.351	7.12E-24	H-cluster2
LGR4	8.60E-28	0.798057	0.469	0.185	1.61E-23	H-cluster2
KANK1	1.92E-27	1.025856	0.642	0.39	3.60E-23	H-cluster2
GUCY1A2	8.90E-27	0.744176	0.416	0.148	1.66E-22	H-cluster2
GAS7	1.27E-26	0.520368	0.796	0.606	2.38E-22	H-cluster2
AUTS2	2.09E-26	0.44259	0.968	0.898	3.91E-22	H-cluster2
LIMCH1	9.53E-26	0.708151	0.647	0.385	1.78E-21	H-cluster2
KIAA1671	9.88E-26	0.697676	0.472	0.198	1.85E-21	H-cluster2
MAST4	1.17E-25	0.777214	0.676	0.454	2.19E-21	H-cluster2
CARMIL1	1.53E-25	0.667447	0.708	0.473	2.86E-21	H-cluster2
ADGRD1	1.61E-25	0.664218	0.544	0.26	3.02E-21	H-cluster2
PSD3	2.30E-25	0.678525	0.653	0.402	4.31E-21	H-cluster2
RERG	2.31E-25	0.861592	0.289	0.077	4.33E-21	H-cluster2
UTRN	4.82E-25	0.506845	0.836	0.748	9.02E-21	H-cluster2
PCOLCE2	1.13E-24	0.71309	0.599	0.316	2.12E-20	H-cluster2

ADAMTSL1	1.38E-24	0.724945	0.706	0.466	2.57E-20	H-cluster2
ADAMTS5	1.52E-24	0.969251	0.541	0.29	2.84E-20	H-cluster2
VCAN	4.65E-24	1.046401	0.56	0.315	8.70E-20	H-cluster2
ANOS1	5.84E-24	0.834983	0.342	0.118	1.09E-19	H-cluster2
PCSK6	7.49E-24	0.790136	0.56	0.305	1.40E-19	H-cluster2
EXT1	1.71E-23	0.600077	0.854	0.705	3.21E-19	H-cluster2
APBB1IP	2.58E-23	0.701216	0.592	0.333	4.83E-19	H-cluster2
ADAMTS16	2.81E-23	0.834023	0.281	0.08	5.26E-19	H-cluster2
ASAP2	4.40E-23	0.629464	0.674	0.426	8.23E-19	H-cluster2
SH3PXD2B	1.23E-22	0.728819	0.613	0.382	2.31E-18	H-cluster2
PDE7A	1.27E-22	0.661056	0.637	0.391	2.37E-18	H-cluster2
PAK3	2.56E-22	0.817474	0.477	0.242	4.78E-18	H-cluster2
GRK5	3.23E-22	0.597279	0.759	0.569	6.05E-18	H-cluster2
ABLIM1	6.28E-22	0.578895	0.716	0.503	1.17E-17	H-cluster2
PAMR1	8.66E-22	0.714202	0.467	0.221	1.62E-17	H-cluster2
LMX1A	2.51E-21	0.626731	0.361	0.136	4.70E-17	H-cluster2
TRERF1	2.55E-21	0.626496	0.645	0.422	4.76E-17	H-cluster2
FGF14	9.05E-20	0.56672	0.626	0.372	1.69E-15	H-cluster2
SRPK2	1.73E-19	0.595158	0.698	0.484	3.24E-15	H-cluster2
ARHGAP29	1.75E-19	0.724235	0.501	0.27	3.27E-15	H-cluster2
FAR2	1.92E-19	0.676869	0.308	0.111	3.60E-15	H-cluster2
THSD4	4.82E-19	0.771116	0.329	0.13	9.01E-15	H-cluster2
PLXNA2	4.84E-19	0.58738	0.324	0.123	9.05E-15	H-cluster2
LTBP2	8.13E-19	0.623375	0.361	0.149	1.52E-14	H-cluster2
PRDM6	1.10E-18	0.610053	0.411	0.184	2.07E-14	H-cluster2
HDAC7	1.22E-18	0.590897	0.507	0.275	2.28E-14	H-cluster2
SULF1	1.28E-18	0.617586	0.623	0.399	2.40E-14	H-cluster2
MMP16	1.39E-18	0.704802	0.31	0.117	2.60E-14	H-cluster2
JAZF1	1.91E-18	0.56782	0.592	0.367	3.58E-14	H-cluster2
ADK	2.73E-18	0.551965	0.743	0.574	5.11E-14	H-cluster2
ZNF521	5.66E-18	0.482457	0.706	0.512	1.06E-13	H-cluster2
MYOF	1.17E-17	0.542056	0.613	0.389	2.19E-13	H-cluster2
PDE1C	2.24E-17	0.607796	0.422	0.203	4.20E-13	H-cluster2
GALNT17	3.20E-17	0.705251	0.324	0.13	5.98E-13	H-cluster2
NAV3	4.17E-17	0.490233	0.814	0.626	7.79E-13	H-cluster2
ZNF385B	4.42E-17	0.741705	0.432	0.22	8.26E-13	H-cluster2
TNXB	5.53E-17	0.28668	0.844	0.668	1.03E-12	H-cluster2
PCNX2	7.07E-17	0.549863	0.562	0.343	1.32E-12	H-cluster2
LTBP1	8.16E-17	0.539764	0.459	0.234	1.53E-12	H-cluster2
VPS13B	1.27E-16	0.440407	0.841	0.762	2.37E-12	H-cluster2
PARD3B	1.46E-16	0.455912	0.881	0.76	2.73E-12	H-cluster2
SORBS2	1.96E-16	0.559803	0.353	0.151	3.66E-12	H-cluster2
ITGBL1	5.14E-16	0.677259	0.305	0.124	9.61E-12	H-cluster2
ARHGAP20	8.62E-16	0.633489	0.501	0.304	1.61E-11	H-cluster2
STARD9	9.50E-16	0.544463	0.653	0.475	1.78E-11	H-cluster2
FNDC1	1.05E-15	0.63451	0.252	0.09	1.97E-11	H-cluster2
ITPR3	1.08E-15	0.445328	0.395	0.191	2.03E-11	H-cluster2

MYO9A	1.18E-15	0.443588	0.759	0.633	2.22E-11	H-cluster2
PTGIS	1.47E-15	0.560498	0.666	0.47	2.76E-11	H-cluster2
NCKAP5	3.08E-15	0.764039	0.286	0.116	5.77E-11	H-cluster2
TENM1	5.68E-15	0.530344	0.271	0.102	1.06E-10	H-cluster2
CACNB4	6.72E-15	0.590368	0.286	0.116	1.26E-10	H-cluster2
OSBPL10	8.45E-15	0.580875	0.52	0.309	1.58E-10	H-cluster2
HRH1	8.90E-15	0.451053	0.329	0.139	1.66E-10	H-cluster2
ADD3	1.05E-14	0.369579	0.841	0.739	1.96E-10	H-cluster2
RBMS1	1.05E-14	0.460556	0.798	0.701	1.97E-10	H-cluster2
SNX24	1.33E-14	0.466089	0.371	0.18	2.50E-10	H-cluster2
CDK6	4.07E-14	0.501261	0.401	0.206	7.62E-10	H-cluster2
EYA4	4.09E-14	0.513743	0.302	0.128	7.65E-10	H-cluster2
RAP1B	4.64E-14	0.460471	0.692	0.536	8.68E-10	H-cluster2
RECK	5.18E-14	0.518176	0.538	0.341	9.69E-10	H-cluster2
SH3D19	5.27E-14	0.428341	0.814	0.709	9.86E-10	H-cluster2
ITGB5	5.38E-14	0.500316	0.501	0.305	1.01E-09	H-cluster2
GLIPR2	6.36E-14	0.497282	0.313	0.143	1.19E-09	H-cluster2
DNM1	6.58E-14	0.470086	0.581	0.399	1.23E-09	H-cluster2
ANTXR1	7.02E-14	0.533015	0.618	0.45	1.31E-09	H-cluster2
LOXL1	8.74E-14	0.566656	0.294	0.128	1.64E-09	H-cluster2
SLC37A3	8.75E-14	0.460281	0.44	0.248	1.64E-09	H-cluster2
KIAA1456	1.42E-13	0.496071	0.302	0.135	2.65E-09	H-cluster2
SHISA6	2.19E-13	0.485189	0.252	0.097	4.09E-09	H-cluster2
EFNA5	2.59E-13	0.618839	0.57	0.382	4.85E-09	H-cluster2
DDX60L	4.48E-13	0.485262	0.406	0.221	8.38E-09	H-cluster2
MYO1E	4.85E-13	0.520276	0.44	0.25	9.08E-09	H-cluster2
DBN1	4.93E-13	0.327536	0.297	0.128	9.23E-09	H-cluster2
HERC4	5.25E-13	0.446664	0.729	0.612	9.82E-09	H-cluster2
ELMO1	5.37E-13	0.470948	0.706	0.577	1.01E-08	H-cluster2
PDGFC	5.64E-13	0.488025	0.26	0.106	1.06E-08	H-cluster2
EVI5	5.68E-13	0.438988	0.671	0.495	1.06E-08	H-cluster2
CDC42BPA	7.49E-13	0.463787	0.682	0.534	1.40E-08	H-cluster2
PALM	9.56E-13	0.441358	0.432	0.249	1.79E-08	H-cluster2
PI16	1.31E-12	0.499805	0.408	0.223	2.45E-08	H-cluster2
CD34	1.71E-12	0.407384	0.589	0.399	3.20E-08	H-cluster2
FGD5	1.85E-12	0.489721	0.427	0.248	3.46E-08	H-cluster2
NEDD9	1.90E-12	0.464301	0.308	0.143	3.55E-08	H-cluster2
SMIM14	1.93E-12	0.486173	0.554	0.375	3.61E-08	H-cluster2
AXL	2.17E-12	0.381445	0.645	0.46	4.06E-08	H-cluster2
PROCR	3.09E-12	0.456569	0.546	0.352	5.79E-08	H-cluster2
ATP2B1	3.23E-12	0.436235	0.462	0.279	6.04E-08	H-cluster2
ARHGAP28	3.72E-12	0.582969	0.31	0.151	6.96E-08	H-cluster2
ADAMTS2	3.85E-12	0.492612	0.432	0.253	7.20E-08	H-cluster2
TNS1	3.94E-12	0.415115	0.541	0.355	7.37E-08	H-cluster2
MID1	4.05E-12	0.564994	0.477	0.297	7.58E-08	H-cluster2
PXN	4.51E-12	0.424293	0.462	0.282	8.44E-08	H-cluster2
WDFY3	4.66E-12	0.379969	0.729	0.591	8.71E-08	H-cluster2

USP54	6.13E-12	0.361525	0.374	0.197	1.15E-07	H-cluster2
NIPAL2	1.14E-11	0.654719	0.342	0.183	2.13E-07	H-cluster2
GFPT2	1.22E-11	0.281287	0.592	0.411	2.29E-07	H-cluster2
TACC2	1.94E-11	0.366114	0.34	0.174	3.64E-07	H-cluster2
ST7	2.52E-11	0.474468	0.552	0.372	4.72E-07	H-cluster2
NLGN4Y	4.22E-11	0.404998	0.374	0.198	7.89E-07	H-cluster2
WLS	4.78E-11	0.462099	0.305	0.153	8.94E-07	H-cluster2
MTHFD1L	5.23E-11	0.435724	0.305	0.15	9.78E-07	H-cluster2
ROBO2	5.35E-11	0.763939	0.525	0.37	1.00E-06	H-cluster2
EPB41L3	5.51E-11	0.61256	0.618	0.476	1.03E-06	H-cluster2
KLF12	5.54E-11	0.376935	0.615	0.424	1.04E-06	H-cluster2
EBF2	5.59E-11	0.458716	0.812	0.731	1.05E-06	H-cluster2
AC005154.6	6.36E-11	0.50727	0.39	0.229	1.19E-06	H-cluster2
FER	6.71E-11	0.356954	0.751	0.658	1.26E-06	H-cluster2
ZMIZ1	8.17E-11	0.426017	0.483	0.311	1.53E-06	H-cluster2
SPSB4	8.24E-11	0.427776	0.255	0.115	1.54E-06	H-cluster2
PDLIM5	9.03E-11	0.448839	0.576	0.41	1.69E-06	H-cluster2
STXBP6	2.43E-10	0.443492	0.443	0.271	4.55E-06	H-cluster2
RASAL2	3.50E-10	0.36983	0.347	0.189	6.54E-06	H-cluster2
GLS	4.03E-10	0.519552	0.629	0.522	7.54E-06	H-cluster2
SH3RF3	4.13E-10	0.409205	0.472	0.302	7.72E-06	H-cluster2
COBLL1	4.28E-10	0.593639	0.324	0.177	8.00E-06	H-cluster2
L3MBTL4	6.00E-10	0.469422	0.308	0.162	1.12E-05	H-cluster2
SLC41A2	6.24E-10	0.297661	0.345	0.185	1.17E-05	H-cluster2
ZFHX3	8.83E-10	0.385893	0.592	0.44	1.65E-05	H-cluster2
TGFBR1	1.07E-09	0.414303	0.374	0.222	2.00E-05	H-cluster2
LVRN	1.23E-09	0.471562	0.302	0.16	2.29E-05	H-cluster2
CORO1C	1.58E-09	0.367433	0.334	0.186	2.96E-05	H-cluster2
TMEM131L	1.60E-09	0.466895	0.467	0.308	2.99E-05	H-cluster2
ST3GAL5	1.65E-09	0.502986	0.432	0.295	3.09E-05	H-cluster2
SRGAP2B	1.67E-09	0.32675	0.369	0.208	3.12E-05	H-cluster2
COL14A1	1.77E-09	0.525157	0.387	0.227	3.32E-05	H-cluster2
DISP1	1.87E-09	0.439954	0.321	0.177	3.49E-05	H-cluster2
BICD1	1.88E-09	0.377214	0.39	0.239	3.52E-05	H-cluster2
PLEKHG1	2.60E-09	0.346135	0.334	0.18	4.87E-05	H-cluster2
TENM3	2.61E-09	0.577184	0.35	0.209	4.88E-05	H-cluster2
TTC7A	2.65E-09	0.35582	0.573	0.41	4.95E-05	H-cluster2
STK39	2.99E-09	0.443747	0.448	0.309	5.59E-05	H-cluster2
RRAS2	3.20E-09	0.444496	0.385	0.235	5.99E-05	H-cluster2
ZNF423	3.50E-09	0.418024	0.424	0.271	6.55E-05	H-cluster2
GLI3	3.60E-09	0.360474	0.66	0.523	6.74E-05	H-cluster2
EEF2K	3.95E-09	0.435878	0.454	0.299	7.39E-05	H-cluster2
GALNT10	3.98E-09	0.27191	0.406	0.236	7.44E-05	H-cluster2
DENND2A	4.34E-09	0.354041	0.743	0.655	8.11E-05	H-cluster2
IQSEC1	4.39E-09	0.343104	0.292	0.154	8.20E-05	H-cluster2
GALNT12	4.52E-09	0.301849	0.252	0.124	8.45E-05	H-cluster2
ITSN2	4.94E-09	0.450583	0.586	0.43	9.25E-05	H-cluster2

FBXL2	4.95E-09	0.340061	0.305	0.166	9.26E-05	H-cluster2
MN1	5.10E-09	0.325898	0.289	0.154	9.54E-05	H-cluster2
TTLL11	5.16E-09	0.408025	0.305	0.168	9.64E-05	H-cluster2
GPSM2	5.33E-09	0.394129	0.34	0.198	9.97E-05	H-cluster2
KMT2C	5.47E-09	0.279904	0.769	0.674	0.000102	H-cluster2
FN1	5.71E-09	0.288775	0.501	0.32	0.000107	H-cluster2
ADSS	5.90E-09	0.412194	0.284	0.153	0.00011	H-cluster2
TRAK1	7.81E-09	0.348483	0.414	0.26	0.000146	H-cluster2
ACSL3	8.24E-09	0.428901	0.464	0.328	0.000154	H-cluster2
EMILIN2	8.87E-09	0.256563	0.337	0.184	0.000166	H-cluster2
PTK2	8.95E-09	0.298797	0.753	0.665	0.000167	H-cluster2
DLGAP4	9.43E-09	0.39335	0.424	0.273	0.000176	H-cluster2
SVIL	1.12E-08	0.400396	0.501	0.354	0.000209	H-cluster2
C11orf80	1.24E-08	0.421613	0.289	0.153	0.000232	H-cluster2
TPST1	1.28E-08	0.332299	0.653	0.507	0.000239	H-cluster2
PPP1R12B	1.76E-08	0.384824	0.557	0.415	0.000328	H-cluster2
APBA1	2.23E-08	0.386766	0.3	0.165	0.000417	H-cluster2
ROBO1	2.34E-08	0.369024	0.735	0.621	0.000438	H-cluster2
HEG1	2.46E-08	0.351635	0.464	0.307	0.000461	H-cluster2
SPTAN1	2.65E-08	0.322345	0.538	0.391	0.000495	H-cluster2
XAF1	2.76E-08	0.338141	0.268	0.144	0.000516	H-cluster2
SRGAP2	2.86E-08	0.422593	0.44	0.301	0.000535	H-cluster2
PHLDB2	3.01E-08	0.30006	0.597	0.44	0.000564	H-cluster2
CDC42BPB	3.17E-08	0.359735	0.464	0.318	0.000592	H-cluster2
EPB41L2	3.23E-08	0.281863	0.833	0.742	0.000603	H-cluster2
GALNT16	3.32E-08	0.34723	0.276	0.15	0.00062	H-cluster2
STK38	3.45E-08	0.362552	0.57	0.434	0.000646	H-cluster2
TTLL7	3.68E-08	0.430144	0.284	0.158	0.000689	H-cluster2
ANXA4	3.96E-08	0.378092	0.491	0.349	0.00074	H-cluster2
ELN	4.01E-08	0.431305	0.488	0.348	0.00075	H-cluster2
TTC3	4.58E-08	0.357965	0.546	0.423	0.000857	H-cluster2
MAP4	4.59E-08	0.314626	0.714	0.629	0.000858	H-cluster2
MAN2A1	5.28E-08	0.379375	0.464	0.324	0.000988	H-cluster2
STARD13	5.41E-08	0.380962	0.568	0.431	0.001012	H-cluster2
MCOLN3	5.73E-08	0.333935	0.302	0.171	0.001072	H-cluster2
STK3	6.41E-08	0.310572	0.732	0.67	0.001199	H-cluster2
APBB2	6.71E-08	0.275188	0.743	0.61	0.001255	H-cluster2
PDLIM7	7.00E-08	0.361778	0.297	0.172	0.001309	H-cluster2
DOCK1	7.72E-08	0.282391	0.714	0.593	0.001444	H-cluster2
ABCC1	8.25E-08	0.406581	0.499	0.362	0.001542	H-cluster2
MCC	8.32E-08	0.435918	0.385	0.248	0.001556	H-cluster2
PAM	8.51E-08	0.322382	0.692	0.582	0.001592	H-cluster2
PKD2	9.32E-08	0.361711	0.597	0.472	0.001743	H-cluster2
ADGRG2	9.49E-08	0.326013	0.255	0.134	0.001775	H-cluster2
SCARA5	1.40E-07	0.305912	0.565	0.403	0.00262	H-cluster2
NEO1	1.55E-07	0.392332	0.305	0.18	0.002906	H-cluster2
RADIL	1.63E-07	0.341651	0.321	0.192	0.003044	H-cluster2

PLCE1	1.68E-07	0.370748	0.257	0.14	0.003138	H-cluster2
LSP1	1.72E-07	0.296806	0.554	0.416	0.003226	H-cluster2
CMIP	1.95E-07	0.284421	0.406	0.259	0.003645	H-cluster2
CPXM2	1.98E-07	0.396221	0.334	0.212	0.003705	H-cluster2
MKL1	2.10E-07	0.278543	0.639	0.508	0.003933	H-cluster2
AFAP1	2.18E-07	0.391295	0.358	0.233	0.004082	H-cluster2
NOX4	2.61E-07	0.539027	0.626	0.56	0.004873	H-cluster2
DENND4C	2.71E-07	0.369964	0.634	0.527	0.005062	H-cluster2
AP3S1	2.74E-07	0.354876	0.411	0.273	0.005118	H-cluster2
FAM171A1	2.79E-07	0.381143	0.305	0.181	0.005209	H-cluster2
FLRT2	3.01E-07	0.368876	0.599	0.474	0.005622	H-cluster2
HSPA12A	3.45E-07	0.331851	0.294	0.174	0.006453	H-cluster2
TAOK1	3.81E-07	0.332093	0.589	0.466	0.00713	H-cluster2
FAM69A	4.00E-07	0.326492	0.398	0.259	0.007476	H-cluster2
SUN1	4.11E-07	0.308746	0.438	0.306	0.007691	H-cluster2
MARK3	4.13E-07	0.304209	0.679	0.584	0.007734	H-cluster2
CCDC186	4.54E-07	0.343707	0.308	0.189	0.008497	H-cluster2
SWAP70	5.00E-07	0.394302	0.401	0.27	0.009346	H-cluster2
KIF13B	6.03E-07	0.318544	0.308	0.183	0.011284	H-cluster2
EPB41L4A	6.51E-07	0.338066	0.374	0.25	0.012185	H-cluster2
LRRC17	6.60E-07	0.306042	0.252	0.14	0.012339	H-cluster2
ELOVL6	6.60E-07	0.250263	0.257	0.143	0.012351	H-cluster2
KPNA1	6.74E-07	0.386324	0.337	0.216	0.01261	H-cluster2
PDE4DIP	6.88E-07	0.408163	0.578	0.469	0.01287	H-cluster2
CPQ	6.97E-07	0.339901	0.708	0.64	0.013044	H-cluster2
UBE2E2	7.34E-07	0.307464	0.645	0.551	0.01373	H-cluster2
ATP11A	7.84E-07	0.285876	0.472	0.333	0.014657	H-cluster2
TPM1	8.81E-07	0.339557	0.401	0.271	0.01647	H-cluster2
USP9Y	9.70E-07	0.348126	0.432	0.301	0.018144	H-cluster2
BASP1	9.98E-07	0.308025	0.305	0.188	0.018664	H-cluster2
SIPA1L3	1.03E-06	0.367357	0.419	0.292	0.019183	H-cluster2
AP3B1	1.04E-06	0.306179	0.576	0.454	0.019465	H-cluster2
SGMS1	1.07E-06	0.326419	0.507	0.374	0.020088	H-cluster2
WWC2	1.08E-06	0.355282	0.499	0.372	0.020178	H-cluster2
TRAPPC10	1.36E-06	0.294848	0.31	0.194	0.025399	H-cluster2
COL12A1	1.58E-06	0.305734	0.443	0.308	0.029596	H-cluster2
TRIOBP	1.65E-06	0.251321	0.382	0.256	0.030786	H-cluster2
ADD1	1.68E-06	0.323754	0.536	0.428	0.03136	H-cluster2
DIAPH1	1.75E-06	0.363824	0.387	0.255	0.032652	H-cluster2
CEP152	1.88E-06	0.340978	0.281	0.169	0.035149	H-cluster2
RAB30	2.64E-06	0.355469	0.408	0.28	0.049387	H-cluster2
ADAMTSL4	2.64E-06	0.301426	0.297	0.184	0.049461	H-cluster2
FBXL20	2.66E-06	0.317992	0.589	0.489	0.049805	H-cluster2
USP32	2.73E-06	0.392793	0.493	0.377	0.050984	H-cluster2
DNMBP	2.75E-06	0.320672	0.456	0.335	0.051522	H-cluster2
PDE10A	2.78E-06	0.396059	0.358	0.238	0.051941	H-cluster2
CORO2B	2.98E-06	0.289808	0.366	0.249	0.055756	H-cluster2

TSC22D1	3.00E-06	0.42432	0.334	0.219	0.056136	H-cluster2
GLI2	3.43E-06	0.307642	0.31	0.199	0.064128	H-cluster2
SREBF2	3.46E-06	0.355568	0.321	0.211	0.064759	H-cluster2
RALGPS2	3.51E-06	0.353636	0.403	0.279	0.065589	H-cluster2
CCNY	3.59E-06	0.313298	0.562	0.461	0.067186	H-cluster2
PRKCE	3.74E-06	0.297464	0.525	0.414	0.06993	H-cluster2
ZNF704	3.92E-06	0.359134	0.515	0.39	0.07325	H-cluster2
SRGAP1	4.69E-06	0.274532	0.602	0.494	0.087696	H-cluster2
PPP2R2A	5.10E-06	0.328244	0.523	0.411	0.09533	H-cluster2
ASAP3	5.14E-06	0.292343	0.353	0.24	0.096122	H-cluster2
PRKD3	5.19E-06	0.269692	0.379	0.257	0.097019	H-cluster2
KIF2A	6.89E-06	0.283938	0.297	0.188	0.12883	H-cluster2
FAM49B	8.15E-06	0.259684	0.297	0.189	0.152496	H-cluster2
MEF2A	8.28E-06	0.272156	0.645	0.559	0.154953	H-cluster2
TAOK3	8.31E-06	0.326335	0.504	0.391	0.155481	H-cluster2
ADGRA3	8.40E-06	0.306735	0.363	0.25	0.157042	H-cluster2
C5orf42	1.02E-05	0.306159	0.525	0.404	0.19135	H-cluster2
JAG1	1.12E-05	0.37757	0.34	0.234	0.210296	H-cluster2
EVC	1.14E-05	0.28219	0.271	0.167	0.21404	H-cluster2
DOPEY1	1.17E-05	0.31391	0.387	0.272	0.219077	H-cluster2
MAPKAP1	1.27E-05	0.293289	0.414	0.3	0.23801	H-cluster2
DCBLD2	1.31E-05	0.32575	0.427	0.317	0.245219	H-cluster2
RUNX1	1.43E-05	0.265309	0.485	0.361	0.26694	H-cluster2
PBX3	1.45E-05	0.336235	0.528	0.425	0.271114	H-cluster2
NSF	1.53E-05	0.25846	0.395	0.276	0.286027	H-cluster2
CTIF	1.63E-05	0.318921	0.284	0.184	0.304241	H-cluster2
CCDC39	1.64E-05	0.367114	0.462	0.356	0.306358	H-cluster2
KANK2	1.66E-05	0.292691	0.361	0.246	0.310441	H-cluster2
GTDC1	1.67E-05	0.31041	0.472	0.352	0.311497	H-cluster2
OPTN	1.68E-05	0.310984	0.408	0.3	0.314073	H-cluster2
C9orf3	1.77E-05	0.309006	0.48	0.358	0.331235	H-cluster2
LARP1B	1.89E-05	0.27735	0.342	0.233	0.353548	H-cluster2
TBC1D22A	1.98E-05	0.290387	0.523	0.425	0.370631	H-cluster2
PRKD1	2.26E-05	0.28637	0.451	0.342	0.422959	H-cluster2
OGDH	2.27E-05	0.250672	0.406	0.289	0.424258	H-cluster2
ATP2B4	2.48E-05	0.262025	0.44	0.328	0.463171	H-cluster2
ADAM10	2.54E-05	0.423087	0.422	0.326	0.474336	H-cluster2
OPHN1	2.61E-05	0.303669	0.692	0.605	0.488531	H-cluster2
APP	3.03E-05	0.298138	0.538	0.419	0.565809	H-cluster2
PPFIA1	3.12E-05	0.312	0.414	0.312	0.583059	H-cluster2
NOVA1	3.17E-05	0.358368	0.828	0.794	0.593842	H-cluster2
BTBD9	3.18E-05	0.267259	0.536	0.424	0.594103	H-cluster2
FAM107B	3.25E-05	0.290102	0.326	0.218	0.607609	H-cluster2
PCNX4	3.66E-05	0.341843	0.43	0.322	0.684836	H-cluster2
SLC12A6	4.04E-05	0.361431	0.286	0.189	0.756122	H-cluster2
DLGAP2	4.05E-05	0.309214	0.257	0.161	0.757298	H-cluster2
ADCY9	4.07E-05	0.315924	0.39	0.284	0.760548	H-cluster2

MYO6	4.15E-05	0.28692	0.347	0.246	0.776763	H-cluster2
NKTR	4.25E-05	0.261133	0.602	0.511	0.79584	H-cluster2
CPEB2	4.48E-05	0.284087	0.286	0.19	0.837438	H-cluster2
AOX1	4.52E-05	0.332937	0.464	0.356	0.845112	H-cluster2
PSME4	4.72E-05	0.280117	0.462	0.354	0.882418	H-cluster2
C2CD2	4.85E-05	0.302278	0.321	0.22	0.907153	H-cluster2
SCRN1	4.87E-05	0.338449	0.286	0.19	0.910741	H-cluster2
PALLD	5.17E-05	0.283381	0.491	0.388	0.966762	H-cluster2
MAST2	5.21E-05	0.283855	0.345	0.242	0.97534	H-cluster2
ERBIN	5.55E-05	0.318788	0.589	0.496	1	H-cluster2
STIM1	5.88E-05	0.334479	0.48	0.397	1	H-cluster2
DOCK7	6.82E-05	0.294507	0.446	0.332	1	H-cluster2
SMG6	7.20E-05	0.271099	0.642	0.577	1	H-cluster2
GABRE	7.45E-05	0.264558	0.294	0.2	1	H-cluster2
KLHL29	7.90E-05	0.390194	0.382	0.283	1	H-cluster2
ZBTB1	8.16E-05	0.269015	0.387	0.284	1	H-cluster2
EXOC1	8.18E-05	0.268506	0.422	0.319	1	H-cluster2
FLNB	9.06E-05	0.291693	0.326	0.229	1	H-cluster2
FMNL2	9.16E-05	0.313426	0.573	0.49	1	H-cluster2
MBP	1.00E-04	0.352228	0.385	0.285	1	H-cluster2
ADAM33	0.000101	0.265619	0.363	0.266	1	H-cluster2
RABGEF1	0.000102	0.300045	0.332	0.236	1	H-cluster2
ATAD2B	0.000106	0.307071	0.517	0.427	1	H-cluster2
VTI1A	0.00013	0.271662	0.615	0.54	1	H-cluster2
CDK8	0.000147	0.285412	0.284	0.19	1	H-cluster2
SDC2	0.000151	0.346453	0.581	0.511	1	H-cluster2
PRSS23	0.000152	0.283946	0.406	0.304	1	H-cluster2
CUEDC1	0.000152	0.299807	0.324	0.228	1	H-cluster2
CEP85L	0.000165	0.292493	0.414	0.309	1	H-cluster2
KREMEN1	0.00019	0.316614	0.374	0.283	1	H-cluster2
HIVEP1	0.000229	0.27815	0.427	0.341	1	H-cluster2
TBCK	0.000275	0.250089	0.525	0.433	1	H-cluster2
IL6ST	0.000294	0.268422	0.592	0.528	1	H-cluster2
C11orf49	0.000321	0.324784	0.366	0.281	1	H-cluster2
ASXL2	0.000323	0.297847	0.294	0.206	1	H-cluster2
DLG1	0.000366	0.251121	0.509	0.425	1	H-cluster2
IL17RD	0.000367	0.296246	0.252	0.173	1	H-cluster2
RIMKLB	0.000368	0.267793	0.334	0.242	1	H-cluster2
GALNT15	0.000369	0.349196	0.289	0.204	1	H-cluster2
KIAA0825	0.000378	0.272969	0.342	0.248	1	H-cluster2
DYNC2LI1	0.000395	0.256098	0.286	0.197	1	H-cluster2
TAF1B	0.000441	0.254184	0.279	0.19	1	H-cluster2
SIPA1L1	0.000487	0.25621	0.263	0.179	1	H-cluster2
CCDC146	0.000489	0.279037	0.408	0.316	1	H-cluster2
CD44	0.000527	0.266821	0.568	0.494	1	H-cluster2
ESCO1	0.000538	0.255814	0.302	0.215	1	H-cluster2
GPC6	0.000587	0.561171	0.408	0.328	1	H-cluster2

CALU	0.000591	0.25158	0.342	0.253	1	H-cluster2
PRIM2	0.000732	0.380569	0.366	0.291	1	H-cluster2
FOXO1	0.000835	0.252314	0.692	0.62	1	H-cluster2
SETD7	0.000975	0.262052	0.464	0.376	1	H-cluster2
HEATR5B	0.001041	0.292275	0.363	0.279	1	H-cluster2
CYP4X1	0.001052	0.254583	0.35	0.27	1	H-cluster2
TTC37	0.001159	0.28513	0.411	0.333	1	H-cluster2
WASF1	0.001529	0.327162	0.318	0.245	1	H-cluster2
FAM126A	0.002117	0.294591	0.443	0.37	1	H-cluster2
BACH1	0.002148	0.289725	0.347	0.275	1	H-cluster2
SPIRE1	0.00267	0.250152	0.286	0.212	1	H-cluster2
PLPP1	0.002914	0.263963	0.517	0.443	1	H-cluster2
FAT4	0.003074	0.3059	0.496	0.428	1	H-cluster2
UBTD2	0.003286	0.282297	0.321	0.253	1	H-cluster2
KIFAP3	0.003459	0.255943	0.435	0.365	1	H-cluster2
PDE8A	0.004113	0.267581	0.52	0.47	1	H-cluster2
TPRG1	0.004253	0.264266	0.308	0.239	1	H-cluster2
AZIN1	0.005677	0.260838	0.286	0.226	1	H-cluster2
RNF13	0.005969	0.258253	0.43	0.362	1	H-cluster2
ODF2L	0.007536	0.265026	0.395	0.332	1	H-cluster2
LGALS1	3.43E-60	1.163273	0.481	0.085	6.41E-56	H-cluster3
CRIP1	5.23E-60	0.723349	0.314	0.018	9.79E-56	H-cluster3
MYADM	1.40E-57	1.181368	0.617	0.172	2.61E-53	H-cluster3
CST3	1.85E-57	1.824169	0.617	0.207	3.46E-53	H-cluster3
NR4A1	1.55E-56	1.557051	0.53	0.119	2.90E-52	H-cluster3
TIMP1	1.46E-55	1.019361	0.47	0.084	2.74E-51	H-cluster3
JUND	4.24E-55	1.184688	0.686	0.227	7.93E-51	H-cluster3
IGFBP6	1.38E-53	1.787527	0.659	0.262	2.59E-49	H-cluster3
UBB	1.33E-52	0.815894	0.402	0.06	2.48E-48	H-cluster3
S100A10	3.96E-52	1.563194	0.564	0.171	7.41E-48	H-cluster3
ACTG1	2.49E-51	1.202391	0.591	0.179	4.65E-47	H-cluster3
S100A6	2.64E-51	1.826697	0.568	0.181	4.94E-47	H-cluster3
S100A11	1.90E-50	1.068632	0.466	0.097	3.56E-46	H-cluster3
UBC	7.28E-49	1.184596	0.545	0.158	1.36E-44	H-cluster3
MFAP4	1.63E-48	1.382443	0.58	0.185	3.05E-44	H-cluster3
EIF1	1.73E-48	1.081175	0.572	0.17	3.23E-44	H-cluster3
CD63	2.43E-48	1.237705	0.496	0.13	4.55E-44	H-cluster3
CD248	2.45E-48	1.087868	0.5	0.12	4.58E-44	H-cluster3
TUBA1B	1.15E-46	0.727858	0.405	0.073	2.15E-42	H-cluster3
LTBP4	1.34E-46	1.296618	0.693	0.323	2.50E-42	H-cluster3
ACTB	7.06E-46	1.339177	0.678	0.314	1.32E-41	H-cluster3
MFAP51	9.50E-46	1.193826	0.765	0.375	1.78E-41	H-cluster3
H3F3B	1.19E-45	1.081168	0.561	0.172	2.22E-41	H-cluster3
CD551	1.60E-45	1.172085	0.746	0.312	2.99E-41	H-cluster3
FSTL1	2.58E-45	1.250014	0.826	0.568	4.82E-41	H-cluster3
HSPA1A	4.41E-45	0.490516	0.322	0.038	8.25E-41	H-cluster3
TPT1	9.12E-45	1.233508	0.652	0.269	1.71E-40	H-cluster3

CLU	1.42E-44	0.968393	0.405	0.075	2.65E-40	H-cluster3
CD81	2.02E-44	0.994327	0.511	0.14	3.77E-40	H-cluster3
NR4A3	1.03E-43	1.432439	0.261	0.021	1.93E-39	H-cluster3
RPS27	1.18E-43	0.822507	0.473	0.112	2.20E-39	H-cluster3
KLF2	4.94E-43	0.988503	0.489	0.13	9.24E-39	H-cluster3
IFITM3	5.02E-43	0.597664	0.352	0.054	9.39E-39	H-cluster3
LMNA	7.77E-43	1.178563	0.636	0.237	1.45E-38	H-cluster3
PTMA	1.44E-41	0.873864	0.496	0.134	2.70E-37	H-cluster3
TMSB4X	2.91E-41	1.293004	0.678	0.322	5.43E-37	H-cluster3
GPRC5A	3.00E-41	1.371618	0.398	0.081	5.61E-37	H-cluster3
ISLR	7.67E-41	0.866391	0.462	0.118	1.43E-36	H-cluster3
RPL36AL	1.20E-40	0.528734	0.314	0.044	2.25E-36	H-cluster3
ITM2A	1.48E-40	0.667953	0.39	0.075	2.77E-36	H-cluster3
CFD	3.54E-40	2.029331	0.723	0.479	6.62E-36	H-cluster3
JUN	4.17E-40	1.22077	0.515	0.153	7.81E-36	H-cluster3
HSPB1	5.11E-40	0.632891	0.337	0.053	9.56E-36	H-cluster3
EMP3	6.57E-40	0.812187	0.443	0.107	1.23E-35	H-cluster3
TMSB10	1.10E-39	1.088477	0.564	0.199	2.06E-35	H-cluster3
HSPA8	1.34E-39	0.56101	0.345	0.057	2.50E-35	H-cluster3
LGALS3BP	3.00E-39	0.752214	0.383	0.077	5.61E-35	H-cluster3
GAPDH	3.15E-39	0.658408	0.341	0.058	5.90E-35	H-cluster3
RPL18	6.34E-39	0.752468	0.451	0.11	1.19E-34	H-cluster3
RPL15	6.67E-39	0.867849	0.436	0.107	1.25E-34	H-cluster3
FTH1	1.52E-38	1.100228	0.481	0.144	2.85E-34	H-cluster3
VAT1	1.75E-38	0.495348	0.292	0.039	3.27E-34	H-cluster3
TUBA1A	1.89E-38	0.81815	0.481	0.133	3.53E-34	H-cluster3
GAS1	2.60E-38	0.885556	0.443	0.113	4.85E-34	H-cluster3
EEF1A1	4.19E-38	1.181902	0.621	0.273	7.85E-34	H-cluster3
B2M	4.99E-38	1.003456	0.716	0.388	9.34E-34	H-cluster3
CPE	8.08E-38	0.982175	0.602	0.233	1.51E-33	H-cluster3
OOEP	1.13E-37	0.820734	0.485	0.135	2.10E-33	H-cluster3
EEF2	1.36E-37	0.633853	0.402	0.089	2.55E-33	H-cluster3
CLIC1	1.82E-37	0.470371	0.295	0.041	3.40E-33	H-cluster3
RPS11	2.21E-37	0.891449	0.477	0.137	4.14E-33	H-cluster3
C17orf58	3.92E-37	0.847536	0.436	0.112	7.33E-33	H-cluster3
RPL8	4.39E-37	0.696219	0.451	0.118	8.21E-33	H-cluster3
CREB51	4.50E-37	0.856597	0.837	0.416	8.41E-33	H-cluster3
STEAP1B	7.44E-37	0.579946	0.402	0.089	1.39E-32	H-cluster3
ATP5E	9.53E-37	0.647684	0.333	0.06	1.78E-32	H-cluster3
LUM	1.07E-36	1.177953	0.53	0.185	1.99E-32	H-cluster3
TAGLN2	1.25E-36	0.721367	0.428	0.112	2.33E-32	H-cluster3
ALDOA	1.37E-36	0.438486	0.345	0.062	2.57E-32	H-cluster3
RPLP1	2.11E-36	0.994371	0.561	0.207	3.95E-32	H-cluster3
LDHA	3.64E-36	0.719672	0.405	0.093	6.80E-32	H-cluster3
RHOB	4.13E-36	0.641277	0.318	0.054	7.73E-32	H-cluster3
METRNL	4.50E-36	0.472958	0.322	0.055	8.41E-32	H-cluster3
RPS8	4.57E-36	0.821828	0.477	0.139	8.54E-32	H-cluster3

RPS4X	5.00E-36	0.745636	0.447	0.121	9.35E-32	H-cluster3
RPL23	9.28E-36	0.546001	0.371	0.077	1.74E-31	H-cluster3
KLF4	2.17E-35	0.990731	0.47	0.14	4.06E-31	H-cluster3
RPS14	2.23E-35	0.875946	0.481	0.149	4.18E-31	H-cluster3
RPL24	5.90E-35	0.609702	0.367	0.078	1.10E-30	H-cluster3
TUBB	6.88E-35	0.543024	0.364	0.077	1.29E-30	H-cluster3
RPL29	7.70E-35	0.755461	0.443	0.122	1.44E-30	H-cluster3
TPPP3	8.42E-35	0.605107	0.311	0.055	1.57E-30	H-cluster3
RPL38	8.43E-35	0.656109	0.371	0.082	1.58E-30	H-cluster3
PRDX1	8.54E-35	0.51615	0.333	0.063	1.60E-30	H-cluster3
IER2	8.88E-35	0.864549	0.409	0.103	1.66E-30	H-cluster3
RPL19	9.72E-35	0.892865	0.455	0.134	1.82E-30	H-cluster3
RPS15	1.12E-34	0.859279	0.458	0.135	2.09E-30	H-cluster3
CALR	2.21E-34	0.478023	0.326	0.061	4.14E-30	H-cluster3
RPL36	2.42E-34	0.685878	0.447	0.121	4.53E-30	H-cluster3
POLR2L	2.51E-34	0.482578	0.292	0.047	4.70E-30	H-cluster3
SRP14	2.57E-34	0.512815	0.322	0.06	4.81E-30	H-cluster3
NFKBIZ	2.77E-34	0.918116	0.496	0.159	5.17E-30	H-cluster3
FAU	3.28E-34	0.71909	0.394	0.095	6.14E-30	H-cluster3
MYL12B	4.24E-34	0.650699	0.402	0.101	7.94E-30	H-cluster3
TMBIM6	7.48E-34	0.722434	0.527	0.184	1.40E-29	H-cluster3
S100A4	8.86E-34	1.262609	0.527	0.214	1.66E-29	H-cluster3
MARCKS	9.79E-34	1.076423	0.451	0.14	1.83E-29	H-cluster3
TIMP2	1.01E-33	1.059095	0.652	0.345	1.89E-29	H-cluster3
DUSP1	1.02E-33	0.881428	0.405	0.106	1.90E-29	H-cluster3
HSPA5	1.11E-33	0.440639	0.307	0.053	2.08E-29	H-cluster3
SOD3	1.79E-33	0.498685	0.261	0.036	3.36E-29	H-cluster3
RPL7A	1.89E-33	0.724851	0.386	0.097	3.53E-29	H-cluster3
PLAC9	2.11E-33	1.001577	0.583	0.256	3.95E-29	H-cluster3
RPL13A	2.50E-33	0.815669	0.504	0.172	4.68E-29	H-cluster3
RPL41	2.56E-33	0.968744	0.542	0.207	4.79E-29	H-cluster3
CYR61	2.62E-33	1.067837	0.364	0.085	4.90E-29	H-cluster3
BAIAP2L1	3.24E-33	0.72867	0.417	0.111	6.05E-29	H-cluster3
FTL	3.74E-33	0.917839	0.417	0.114	6.99E-29	H-cluster3
RPS27A	3.95E-33	0.849801	0.496	0.163	7.40E-29	H-cluster3
TOMM7	5.45E-33	0.467378	0.326	0.063	1.02E-28	H-cluster3
RPL28	6.02E-33	0.779407	0.504	0.168	1.13E-28	H-cluster3
PPDPF	7.05E-33	0.416625	0.269	0.039	1.32E-28	H-cluster3
RPS13	1.00E-32	0.714876	0.383	0.094	1.87E-28	H-cluster3
UBA52	1.18E-32	0.633634	0.413	0.108	2.20E-28	H-cluster3
JUNB	1.21E-32	1.117212	0.47	0.149	2.25E-28	H-cluster3
CDV3	1.73E-32	0.623902	0.39	0.096	3.23E-28	H-cluster3
UAP1	2.28E-32	1.07934	0.636	0.282	4.26E-28	H-cluster3
OGN	3.17E-32	0.775008	0.33	0.071	5.92E-28	H-cluster3
TNXB1	3.87E-32	0.87664	0.841	0.687	7.24E-28	H-cluster3
RPL3	4.40E-32	0.748476	0.405	0.108	8.22E-28	H-cluster3
GFPT21	4.88E-32	1.099144	0.739	0.394	9.13E-28	H-cluster3

MMP2	5.23E-32	1.026101	0.553	0.231	9.78E-28	H-cluster3
PSAP	8.33E-32	0.908196	0.545	0.222	1.56E-27	H-cluster3
PPIC	8.41E-32	0.397708	0.299	0.054	1.57E-27	H-cluster3
SPARC	1.10E-31	1.097833	0.587	0.272	2.05E-27	H-cluster3
CFL1	2.40E-31	0.485206	0.333	0.071	4.49E-27	H-cluster3
RPS5	6.62E-31	0.546153	0.33	0.07	1.24E-26	H-cluster3
PPIA	8.22E-31	0.645456	0.473	0.156	1.54E-26	H-cluster3
RABAC1	9.33E-31	0.384868	0.269	0.044	1.75E-26	H-cluster3
LRRN4CL	9.43E-31	0.401775	0.265	0.042	1.76E-26	H-cluster3
VIM	1.53E-30	1.227181	0.659	0.379	2.86E-26	H-cluster3
RPL27A	1.63E-30	0.720237	0.432	0.131	3.05E-26	H-cluster3
FOSL2	2.17E-30	0.699579	0.345	0.081	4.06E-26	H-cluster3
OST4	2.19E-30	0.365731	0.254	0.038	4.10E-26	H-cluster3
CEBPD	2.56E-30	0.891619	0.356	0.088	4.80E-26	H-cluster3
RPS20	2.60E-30	0.555148	0.386	0.1	4.87E-26	H-cluster3
RPL10	3.34E-30	0.990698	0.58	0.269	6.26E-26	H-cluster3
RPS24	3.88E-30	0.698637	0.458	0.151	7.26E-26	H-cluster3
SH3BGRL3	5.40E-30	0.399476	0.28	0.049	1.01E-25	H-cluster3
RPLP2	5.73E-30	0.698025	0.466	0.154	1.07E-25	H-cluster3
XBP1	7.74E-30	0.509282	0.314	0.065	1.45E-25	H-cluster3
VSIR	9.00E-30	0.519679	0.36	0.088	1.68E-25	H-cluster3
IGFBP5	1.01E-29	1.323992	0.705	0.471	1.89E-25	H-cluster3
PGRMC1	1.09E-29	0.369441	0.269	0.046	2.03E-25	H-cluster3
RPS12	1.50E-29	0.811938	0.477	0.169	2.81E-25	H-cluster3
ZFP36	1.61E-29	1.181711	0.443	0.144	3.01E-25	H-cluster3
RPL10A	1.61E-29	0.597125	0.398	0.113	3.02E-25	H-cluster3
FHOD31	1.66E-29	0.764138	0.614	0.254	3.11E-25	H-cluster3
RPS7	1.66E-29	0.552702	0.428	0.124	3.11E-25	H-cluster3
RPL11	2.20E-29	0.89472	0.462	0.158	4.12E-25	H-cluster3
RPL13	2.44E-29	0.945036	0.606	0.297	4.56E-25	H-cluster3
CHCHD2	2.61E-29	0.462024	0.33	0.075	4.87E-25	H-cluster3
RPS9	2.83E-29	0.717305	0.47	0.159	5.29E-25	H-cluster3
PPIB	3.62E-29	0.479363	0.307	0.065	6.77E-25	H-cluster3
TIMP3	3.97E-29	1.017057	0.754	0.54	7.42E-25	H-cluster3
RPL23A	4.49E-29	0.746346	0.432	0.14	8.40E-25	H-cluster3
GNAS	6.41E-29	0.687628	0.633	0.304	1.20E-24	H-cluster3
RPS3A	6.44E-29	0.506573	0.345	0.083	1.20E-24	H-cluster3
ANXA5	6.61E-29	0.721046	0.511	0.188	1.24E-24	H-cluster3
RPS2	9.77E-29	0.753267	0.447	0.144	1.83E-24	H-cluster3
TUBB4B	9.83E-29	0.522363	0.28	0.055	1.84E-24	H-cluster3
SERPING1	1.10E-28	0.97379	0.576	0.291	2.06E-24	H-cluster3
APOO	1.16E-28	0.800067	0.504	0.191	2.17E-24	H-cluster3
TUBB6	1.62E-28	0.709624	0.564	0.231	3.03E-24	H-cluster3
TMEM109	1.96E-28	0.368813	0.265	0.048	3.67E-24	H-cluster3
CDKN1C	4.25E-28	0.472489	0.318	0.073	7.96E-24	H-cluster3
RPS19	4.80E-28	0.676534	0.485	0.169	8.97E-24	H-cluster3
RPL32	4.86E-28	0.739617	0.496	0.182	9.08E-24	H-cluster3

COX7C	5.23E-28	0.404111	0.265	0.048	9.78E-24	H-cluster3
HLA-A	5.60E-28	0.621204	0.511	0.189	1.05E-23	H-cluster3
MT2A	5.98E-28	0.603639	0.314	0.071	1.12E-23	H-cluster3
RPL34	6.09E-28	0.776413	0.466	0.169	1.14E-23	H-cluster3
GRN	7.74E-28	0.363632	0.307	0.065	1.45E-23	H-cluster3
RPS25	1.12E-27	0.659373	0.439	0.146	2.10E-23	H-cluster3
RPS18	1.21E-27	0.787983	0.481	0.18	2.26E-23	H-cluster3
RPL7	1.28E-27	0.567233	0.428	0.133	2.40E-23	H-cluster3
DBN11	1.29E-27	0.581089	0.402	0.12	2.40E-23	H-cluster3
MYL6	1.40E-27	0.674426	0.523	0.213	2.61E-23	H-cluster3
PRELP	2.54E-27	0.654747	0.398	0.119	4.75E-23	H-cluster3
GALNT101	3.27E-27	0.692333	0.545	0.22	6.11E-23	H-cluster3
SERF2	3.30E-27	0.739994	0.428	0.148	6.17E-23	H-cluster3
CTGF	4.07E-27	0.703769	0.311	0.072	7.61E-23	H-cluster3
SKIL	4.46E-27	0.782216	0.549	0.236	8.35E-23	H-cluster3
EID1	5.25E-27	0.624929	0.341	0.091	9.81E-23	H-cluster3
ANXA2	7.03E-27	0.776319	0.708	0.43	1.31E-22	H-cluster3
ADAL	7.41E-27	1.159438	0.322	0.082	1.39E-22	H-cluster3
MIDN	1.39E-26	0.539272	0.326	0.081	2.59E-22	H-cluster3
DNAJA1	1.48E-26	0.552975	0.333	0.084	2.77E-22	H-cluster3
COX4I1	1.53E-26	0.438284	0.265	0.051	2.86E-22	H-cluster3
RPS23	4.25E-26	0.72223	0.447	0.162	7.95E-22	H-cluster3
MGP	4.70E-26	1.277755	0.523	0.237	8.79E-22	H-cluster3
MELTF	4.71E-26	0.390094	0.254	0.048	8.81E-22	H-cluster3
RPL27	5.20E-26	0.471846	0.333	0.086	9.72E-22	H-cluster3
ACKR3	5.71E-26	0.893295	0.617	0.338	1.07E-21	H-cluster3
CAVIN1	7.74E-26	0.613739	0.477	0.178	1.45E-21	H-cluster3
GSTP1	8.06E-26	0.379927	0.273	0.056	1.51E-21	H-cluster3
RPSA	8.18E-26	0.499444	0.386	0.113	1.53E-21	H-cluster3
SFRP2	8.89E-26	0.908464	0.254	0.05	1.66E-21	H-cluster3
APLP2	9.98E-26	0.538256	0.466	0.164	1.87E-21	H-cluster3
IGF2	1.66E-25	0.7163	0.261	0.053	3.10E-21	H-cluster3
SELENOM	1.82E-25	0.411662	0.326	0.082	3.40E-21	H-cluster3
LAMB2	2.10E-25	0.574347	0.458	0.171	3.93E-21	H-cluster3
F10	2.18E-25	0.570288	0.341	0.097	4.08E-21	H-cluster3
RPL4	2.22E-25	0.510417	0.345	0.095	4.16E-21	H-cluster3
FLNA	2.30E-25	0.551417	0.39	0.12	4.31E-21	H-cluster3
OLFML2A	2.34E-25	0.678311	0.33	0.089	4.38E-21	H-cluster3
RPL35A	2.35E-25	0.61501	0.394	0.127	4.40E-21	H-cluster3
B4GALT1	2.56E-25	0.799567	0.545	0.226	4.78E-21	H-cluster3
PI161	2.62E-25	0.91774	0.523	0.214	4.90E-21	H-cluster3
PLAT	2.67E-25	0.718794	0.33	0.086	4.99E-21	H-cluster3
ECM1	3.30E-25	0.397154	0.292	0.068	6.17E-21	H-cluster3
ADI1	3.39E-25	0.38603	0.303	0.073	6.34E-21	H-cluster3
GNB2	4.09E-25	0.338664	0.307	0.073	7.66E-21	H-cluster3
OLFML3	5.52E-25	0.411718	0.295	0.069	1.03E-20	H-cluster3
ITM2B	6.71E-25	0.819377	0.621	0.365	1.25E-20	H-cluster3

RPS3	6.88E-25	0.603284	0.455	0.166	1.29E-20	H-cluster3
UGDH	7.22E-25	1.149986	0.428	0.148	1.35E-20	H-cluster3
ARL6IP5	8.05E-25	0.463421	0.367	0.109	1.51E-20	H-cluster3
PDIA3	1.09E-24	0.546087	0.424	0.144	2.04E-20	H-cluster3
C1R	1.14E-24	0.924208	0.534	0.264	2.13E-20	H-cluster3
RACK1	1.44E-24	0.522064	0.386	0.122	2.69E-20	H-cluster3
ATP1B3	1.48E-24	0.709502	0.341	0.096	2.77E-20	H-cluster3
LAPTM4A	1.63E-24	0.761607	0.527	0.247	3.05E-20	H-cluster3
CLIC4	2.07E-24	0.917615	0.527	0.219	3.87E-20	H-cluster3
FOS	2.25E-24	1.063019	0.545	0.255	4.21E-20	H-cluster3
FBN11	2.36E-24	0.597146	0.947	0.793	4.42E-20	H-cluster3
HSPB8	2.60E-24	0.538492	0.277	0.061	4.86E-20	H-cluster3
RPS17	2.72E-24	0.427336	0.284	0.064	5.09E-20	H-cluster3
RPL5	3.25E-24	0.584631	0.409	0.135	6.07E-20	H-cluster3
HSP90AB1	3.40E-24	0.44527	0.379	0.116	6.37E-20	H-cluster3
RPL14	3.54E-24	0.583249	0.39	0.126	6.62E-20	H-cluster3
PTMS	4.19E-24	0.43695	0.333	0.092	7.83E-20	H-cluster3
RPS16	5.23E-24	0.553244	0.371	0.114	9.78E-20	H-cluster3
H3F3A	9.34E-24	0.552299	0.436	0.162	1.75E-19	H-cluster3
RPS26	1.03E-23	0.335819	0.261	0.056	1.92E-19	H-cluster3
MXRA7	1.30E-23	0.445632	0.417	0.139	2.43E-19	H-cluster3
RNH1	1.34E-23	0.310627	0.318	0.084	2.51E-19	H-cluster3
HLA-C	1.34E-23	0.551518	0.367	0.113	2.51E-19	H-cluster3
PRG41	1.47E-23	1.213349	0.417	0.151	2.75E-19	H-cluster3
FN11	1.63E-23	0.951998	0.595	0.316	3.05E-19	H-cluster3
RPL21	2.55E-23	0.592493	0.348	0.104	4.76E-19	H-cluster3
DDAH2	2.77E-23	0.378716	0.326	0.091	5.19E-19	H-cluster3
FOSB	2.92E-23	0.954567	0.413	0.145	5.45E-19	H-cluster3
RPS15A	3.10E-23	0.531285	0.348	0.103	5.79E-19	H-cluster3
P4HB	3.32E-23	0.39653	0.333	0.092	6.20E-19	H-cluster3
RPL37	3.36E-23	0.647423	0.455	0.177	6.28E-19	H-cluster3
LAMP1	4.88E-23	0.394047	0.356	0.104	9.14E-19	H-cluster3
RPS6	5.19E-23	0.6645	0.458	0.183	9.71E-19	H-cluster3
PMEPA1	5.42E-23	0.526088	0.288	0.072	1.01E-18	H-cluster3
PCOLCE	8.30E-23	0.616482	0.428	0.158	1.55E-18	H-cluster3
SPSB41	1.41E-22	0.467151	0.352	0.105	2.64E-18	H-cluster3
HLA-B	1.86E-22	0.600939	0.572	0.258	3.48E-18	H-cluster3
PFN1	1.99E-22	0.410118	0.318	0.088	3.72E-18	H-cluster3
TMA7	2.31E-22	0.390681	0.28	0.068	4.31E-18	H-cluster3
RHOA	2.63E-22	0.596978	0.614	0.298	4.91E-18	H-cluster3
GNAI2	2.80E-22	0.342908	0.284	0.071	5.24E-18	H-cluster3
SCARA51	3.02E-22	0.638867	0.67	0.394	5.64E-18	H-cluster3
MEDAG	4.23E-22	0.777673	0.561	0.277	7.92E-18	H-cluster3
SDCBP	4.49E-22	0.548852	0.492	0.201	8.40E-18	H-cluster3
SOD1	4.94E-22	0.329585	0.299	0.08	9.24E-18	H-cluster3
ATP6AP2	6.55E-22	0.391319	0.277	0.07	1.22E-17	H-cluster3
RPLP0	8.02E-22	0.559697	0.413	0.153	1.50E-17	H-cluster3

PDGFRA	8.74E-22	0.480458	0.386	0.134	1.63E-17	H-cluster3
TSC22D3	9.12E-22	0.475832	0.311	0.089	1.71E-17	H-cluster3
DACT2	1.35E-21	0.485782	0.269	0.066	2.52E-17	H-cluster3
MYL12A	1.48E-21	0.406734	0.288	0.077	2.77E-17	H-cluster3
HSP90AA1	2.36E-21	0.598728	0.545	0.252	4.42E-17	H-cluster3
CD59	2.89E-21	0.548774	0.367	0.127	5.41E-17	H-cluster3
SELENOS	4.27E-21	0.443413	0.341	0.108	7.99E-17	H-cluster3
ARF1	4.42E-21	0.334712	0.318	0.092	8.26E-17	H-cluster3
DYNLL1	4.72E-21	0.357807	0.292	0.078	8.82E-17	H-cluster3
NT5E	7.04E-21	0.616367	0.409	0.154	1.32E-16	H-cluster3
COL1A1	8.40E-21	1.26442	0.564	0.316	1.57E-16	H-cluster3
LTBP11	9.50E-21	0.642991	0.538	0.239	1.78E-16	H-cluster3
RPL37A	9.56E-21	0.600505	0.428	0.171	1.79E-16	H-cluster3
NTM1	1.34E-20	0.655116	0.67	0.366	2.50E-16	H-cluster3
ARF4	1.36E-20	0.453649	0.405	0.146	2.55E-16	H-cluster3
PCBP1	1.59E-20	0.353661	0.277	0.073	2.98E-16	H-cluster3
HLA-E	1.63E-20	0.559178	0.405	0.151	3.06E-16	H-cluster3
GJA1	1.80E-20	0.447443	0.303	0.089	3.38E-16	H-cluster3
RPL35	2.16E-20	0.485348	0.402	0.147	4.04E-16	H-cluster3
C1S	2.22E-20	0.789821	0.67	0.434	4.15E-16	H-cluster3
SET	2.50E-20	0.313781	0.254	0.062	4.67E-16	H-cluster3
ALDH1A3	2.60E-20	0.699146	0.36	0.125	4.86E-16	H-cluster3
HSP90B1	3.54E-20	0.533282	0.405	0.158	6.61E-16	H-cluster3
DDX21	3.76E-20	0.57533	0.303	0.09	7.03E-16	H-cluster3
PCOLCE21	4.16E-20	0.610722	0.629	0.338	7.78E-16	H-cluster3
RPS29	4.22E-20	0.656407	0.496	0.226	7.89E-16	H-cluster3
GUCY1A21	4.36E-20	0.604836	0.439	0.171	8.16E-16	H-cluster3
ADAMTS21	4.52E-20	0.5149	0.542	0.245	8.45E-16	H-cluster3
PLEKHA7	5.38E-20	0.448621	0.36	0.121	1.01E-15	H-cluster3
CRY1	5.61E-20	0.593318	0.386	0.137	1.05E-15	H-cluster3
NFKBIA	5.90E-20	0.581958	0.33	0.106	1.10E-15	H-cluster3
HIC1	6.00E-20	0.337257	0.265	0.07	1.12E-15	H-cluster3
FBLN2	6.89E-20	0.850477	0.629	0.411	1.29E-15	H-cluster3
BSG	7.22E-20	0.297096	0.269	0.072	1.35E-15	H-cluster3
EEF1D	8.30E-20	0.423246	0.364	0.125	1.55E-15	H-cluster3
COL6A2	8.91E-20	0.692521	0.769	0.585	1.67E-15	H-cluster3
C14orf132	9.40E-20	0.275265	0.265	0.067	1.76E-15	H-cluster3
ZYX	9.66E-20	0.319872	0.28	0.076	1.81E-15	H-cluster3
SMURF21	1.09E-19	0.620082	0.803	0.516	2.03E-15	H-cluster3
NNMT	1.19E-19	0.592359	0.587	0.295	2.22E-15	H-cluster3
TPM4	1.27E-19	0.506927	0.379	0.134	2.37E-15	H-cluster3
ANKRD30BL	1.76E-19	0.918192	0.542	0.263	3.28E-15	H-cluster3
ADGRD11	1.84E-19	0.633803	0.583	0.281	3.43E-15	H-cluster3
RRBP1	2.07E-19	0.467182	0.591	0.296	3.88E-15	H-cluster3
MGAT1	2.73E-19	0.32372	0.284	0.081	5.10E-15	H-cluster3
RPS28	2.88E-19	0.336882	0.261	0.069	5.38E-15	H-cluster3

RPL6	2.97E-19	0.523863	0.394	0.152	5.56E-15	H-cluster3
NACA	3.06E-19	0.470067	0.337	0.116	5.73E-15	H-cluster3
PNRC1	3.35E-19	0.437675	0.326	0.103	6.27E-15	H-cluster3
CD164	4.47E-19	0.439861	0.383	0.143	8.36E-15	H-cluster3
PEBP1	5.33E-19	0.390162	0.288	0.084	9.97E-15	H-cluster3
RPL30	5.72E-19	0.663853	0.496	0.24	1.07E-14	H-cluster3
CHD1	6.44E-19	0.997965	0.523	0.268	1.20E-14	H-cluster3
ATP5B	6.68E-19	0.303878	0.269	0.075	1.25E-14	H-cluster3
GALNT151	7.29E-19	0.431602	0.443	0.175	1.36E-14	H-cluster3
EGR1	7.47E-19	0.872671	0.424	0.185	1.40E-14	H-cluster3
EIF5	8.42E-19	0.517089	0.417	0.165	1.58E-14	H-cluster3
GPNMB	8.53E-19	0.555708	0.432	0.186	1.60E-14	H-cluster3
DAZAP2	1.17E-18	0.298434	0.295	0.088	2.18E-14	H-cluster3
OAT	1.51E-18	0.255437	0.292	0.085	2.83E-14	H-cluster3
AHNAK	1.75E-18	0.676271	0.678	0.5	3.28E-14	H-cluster3
GPX4	2.01E-18	0.443104	0.405	0.159	3.76E-14	H-cluster3
CTSK	2.06E-18	0.414569	0.333	0.114	3.85E-14	H-cluster3
EMILIN21	2.34E-18	0.626705	0.42	0.18	4.38E-14	H-cluster3
CTSL	2.49E-18	0.466945	0.322	0.106	4.66E-14	H-cluster3
KLF9	2.92E-18	0.513275	0.451	0.188	5.47E-14	H-cluster3
ATP1A1	3.10E-18	0.487465	0.311	0.102	5.79E-14	H-cluster3
ZSWIM6	3.25E-18	0.978253	0.663	0.412	6.09E-14	H-cluster3
PSMA7	4.26E-18	0.273081	0.269	0.075	7.97E-14	H-cluster3
COL6A1	4.28E-18	0.777899	0.636	0.432	8.00E-14	H-cluster3
SURF4	4.65E-18	0.281485	0.299	0.093	8.70E-14	H-cluster3
CHRDL1	4.85E-18	0.550009	0.47	0.215	9.08E-14	H-cluster3
HEG11	7.08E-18	0.592584	0.568	0.298	1.32E-13	H-cluster3
SLC39A14	7.49E-18	0.760665	0.258	0.073	1.40E-13	H-cluster3
YWHAB	8.24E-18	0.372572	0.39	0.151	1.54E-13	H-cluster3
NECTIN2	9.37E-18	0.414152	0.303	0.097	1.75E-13	H-cluster3
OS9	1.08E-17	0.366035	0.318	0.104	2.02E-13	H-cluster3
SKI	1.18E-17	0.455599	0.545	0.262	2.20E-13	H-cluster3
HFM1	1.22E-17	0.674069	0.712	0.49	2.28E-13	H-cluster3
S100A13	1.23E-17	0.459682	0.402	0.163	2.29E-13	H-cluster3
SPSB1	1.26E-17	0.868694	0.527	0.269	2.36E-13	H-cluster3
CYP4B1	1.44E-17	0.416649	0.337	0.119	2.70E-13	H-cluster3
TES	1.91E-17	0.39091	0.28	0.086	3.58E-13	H-cluster3
PLTP	4.21E-17	0.420859	0.356	0.135	7.88E-13	H-cluster3
PAMR11	4.30E-17	0.43153	0.523	0.233	8.04E-13	H-cluster3
HRH11	5.13E-17	0.720999	0.371	0.148	9.59E-13	H-cluster3
KCNAB11	5.54E-17	0.502335	0.549	0.271	1.04E-12	H-cluster3
NPR1	5.81E-17	0.340264	0.258	0.075	1.09E-12	H-cluster3
CNTN41	6.41E-17	0.43013	0.716	0.394	1.20E-12	H-cluster3
DLGAP41	6.56E-17	0.422175	0.538	0.261	1.23E-12	H-cluster3
CD151	6.92E-17	0.315096	0.265	0.081	1.29E-12	H-cluster3
NAMPT	7.06E-17	1.018739	0.326	0.124	1.32E-12	H-cluster3
CALM1	7.84E-17	0.376644	0.383	0.15	1.47E-12	H-cluster3

VEGFA	9.77E-17	0.404651	0.371	0.145	1.83E-12	H-cluster3
ANXA1	9.82E-17	0.62708	0.561	0.326	1.84E-12	H-cluster3
ARHGDIA	1.01E-16	0.376259	0.337	0.121	1.89E-12	H-cluster3
AEBP1	1.02E-16	0.5177	0.402	0.171	1.91E-12	H-cluster3
NEDD91	1.05E-16	0.479161	0.371	0.144	1.96E-12	H-cluster3
YWHAQ	1.35E-16	0.432171	0.504	0.237	2.52E-12	H-cluster3
DDX5	1.51E-16	0.513714	0.731	0.48	2.82E-12	H-cluster3
ESYT2	1.74E-16	0.675429	0.727	0.473	3.26E-12	H-cluster3
ENO1	2.41E-16	0.352484	0.269	0.083	4.51E-12	H-cluster3
EIF1AX	2.69E-16	0.339629	0.322	0.116	5.03E-12	H-cluster3
PCYOX1	2.73E-16	0.315275	0.314	0.107	5.10E-12	H-cluster3
SLC4A41	2.98E-16	0.492345	0.466	0.211	5.58E-12	H-cluster3
EFHD1	3.68E-16	0.428214	0.417	0.175	6.89E-12	H-cluster3
ELOVL5	4.09E-16	0.908879	0.375	0.155	7.65E-12	H-cluster3
PDE1C1	4.25E-16	0.509185	0.47	0.214	7.94E-12	H-cluster3
DIAPH11	4.44E-16	0.436356	0.504	0.24	8.31E-12	H-cluster3
YBX1	5.03E-16	0.350626	0.307	0.108	9.42E-12	H-cluster3
COX6C	5.40E-16	0.301784	0.269	0.085	1.01E-11	H-cluster3
GLUL	5.43E-16	0.344069	0.405	0.166	1.02E-11	H-cluster3
PPP1R15A	5.72E-16	0.327964	0.273	0.087	1.07E-11	H-cluster3
GSN	8.12E-16	1.116198	0.758	0.701	1.52E-11	H-cluster3
SERPINF1	9.72E-16	0.585676	0.417	0.203	1.82E-11	H-cluster3
MYL9	1.03E-15	0.293978	0.371	0.143	1.92E-11	H-cluster3
SEMA3C1	1.11E-15	0.290093	0.837	0.503	2.08E-11	H-cluster3
TRIR	1.30E-15	0.305909	0.311	0.109	2.43E-11	H-cluster3
RPN1	1.35E-15	0.294886	0.277	0.089	2.53E-11	H-cluster3
TOB2	1.53E-15	0.422904	0.322	0.121	2.87E-11	H-cluster3
ATP13A3	1.59E-15	0.701166	0.527	0.273	2.98E-11	H-cluster3
HINT1	1.60E-15	0.257733	0.265	0.085	2.99E-11	H-cluster3
KLF13	1.82E-15	0.255484	0.314	0.109	3.40E-11	H-cluster3
PTHLH	2.16E-15	0.573246	0.277	0.093	4.04E-11	H-cluster3
REEP5	2.28E-15	0.363417	0.413	0.179	4.27E-11	H-cluster3
ADAMTSL41	2.44E-15	0.28739	0.405	0.17	4.56E-11	H-cluster3
HTRA3	2.65E-15	0.684743	0.534	0.309	4.97E-11	H-cluster3
PRNP	2.96E-15	0.294726	0.269	0.089	5.53E-11	H-cluster3
HTRA1	2.97E-15	0.403929	0.292	0.103	5.55E-11	H-cluster3
SSR3	3.70E-15	0.367912	0.314	0.116	6.93E-11	H-cluster3
ITGA111	4.19E-15	0.405421	0.636	0.346	7.83E-11	H-cluster3
ZNF90	5.49E-15	0.352014	0.311	0.115	1.03E-10	H-cluster3
ZFP36L2	6.20E-15	0.571624	0.568	0.336	1.16E-10	H-cluster3
CHST15	6.41E-15	0.404145	0.261	0.086	1.20E-10	H-cluster3
IL4R	7.08E-15	0.290732	0.428	0.184	1.33E-10	H-cluster3
SRSF3	7.75E-15	0.455681	0.436	0.205	1.45E-10	H-cluster3
ADGRG21	7.97E-15	0.449149	0.33	0.129	1.49E-10	H-cluster3
RTN4	9.20E-15	0.536517	0.758	0.543	1.72E-10	H-cluster3
PSMB1	1.23E-14	0.309062	0.303	0.11	2.31E-10	H-cluster3

ZBTB7A	1.40E-14	0.395405	0.39	0.171	2.62E-10	H-cluster3
PTTG1IP	1.58E-14	0.257158	0.295	0.105	2.96E-10	H-cluster3
PKM	1.75E-14	0.428526	0.261	0.088	3.28E-10	H-cluster3
LITAF	1.91E-14	0.474582	0.318	0.123	3.57E-10	H-cluster3
FGFR1	2.31E-14	0.59577	0.689	0.478	4.32E-10	H-cluster3
PALM1	2.33E-14	0.363468	0.508	0.25	4.36E-10	H-cluster3
PXDC1	2.43E-14	0.390067	0.269	0.092	4.55E-10	H-cluster3
NTN4	2.45E-14	0.57267	0.379	0.165	4.59E-10	H-cluster3
TXNRD1	2.74E-14	0.485243	0.564	0.328	5.13E-10	H-cluster3
PLEKHM2	3.45E-14	0.451329	0.443	0.211	6.44E-10	H-cluster3
EIF2S2	3.75E-14	0.277076	0.295	0.106	7.02E-10	H-cluster3
ABHD2	3.89E-14	0.36593	0.333	0.13	7.28E-10	H-cluster3
ETF1	4.19E-14	0.43622	0.307	0.116	7.84E-10	H-cluster3
STAT3	4.83E-14	0.638705	0.629	0.375	9.03E-10	H-cluster3
TMTC11	4.86E-14	0.501479	0.576	0.329	9.08E-10	H-cluster3
EGLN1	4.87E-14	0.438564	0.352	0.146	9.10E-10	H-cluster3
SCPEP1	4.91E-14	0.345552	0.288	0.104	9.19E-10	H-cluster3
CYB5R3	5.81E-14	0.321476	0.307	0.118	1.09E-09	H-cluster3
PRRX2	6.04E-14	0.311544	0.318	0.122	1.13E-09	H-cluster3
RHEB	7.77E-14	0.382669	0.341	0.137	1.45E-09	H-cluster3
CSF1	1.09E-13	0.409581	0.356	0.158	2.04E-09	H-cluster3
AXL1	1.15E-13	0.554584	0.693	0.468	2.15E-09	H-cluster3
CAPZB	1.17E-13	0.396648	0.549	0.29	2.19E-09	H-cluster3
YWHAG	1.20E-13	0.461633	0.375	0.163	2.25E-09	H-cluster3
CCNL1	1.22E-13	0.572354	0.561	0.328	2.28E-09	H-cluster3
SQSTM1	1.53E-13	0.615063	0.485	0.269	2.87E-09	H-cluster3
PLCB11	1.55E-13	0.428102	0.765	0.509	2.90E-09	H-cluster3
NCL	1.68E-13	0.344365	0.337	0.139	3.15E-09	H-cluster3
WNT2B	1.80E-13	0.321343	0.273	0.099	3.36E-09	H-cluster3
PFDN5	1.81E-13	0.357496	0.36	0.157	3.38E-09	H-cluster3
STK32B1	1.83E-13	0.347348	0.553	0.288	3.42E-09	H-cluster3
EIF4G1	1.83E-13	0.335406	0.292	0.11	3.43E-09	H-cluster3
SRPX2	2.61E-13	0.447547	0.534	0.291	4.89E-09	H-cluster3
SIK3	2.97E-13	0.542603	0.792	0.574	5.55E-09	H-cluster3
STK17A	3.06E-13	0.471875	0.326	0.137	5.72E-09	H-cluster3
GABARAPL1	3.34E-13	0.3987	0.33	0.136	6.24E-09	H-cluster3
TIPARP	3.56E-13	0.34351	0.303	0.119	6.66E-09	H-cluster3
METTL7A	3.64E-13	0.341053	0.348	0.146	6.81E-09	H-cluster3
DSTN	3.74E-13	0.396627	0.519	0.286	6.99E-09	H-cluster3
PCSK61	3.77E-13	0.447087	0.576	0.328	7.05E-09	H-cluster3
KCNB21	3.88E-13	0.300262	0.53	0.267	7.26E-09	H-cluster3
ABL1	3.90E-13	0.513934	0.727	0.514	7.30E-09	H-cluster3
SREBF1	4.63E-13	0.328419	0.337	0.141	8.66E-09	H-cluster3
ATP6V0E1	5.05E-13	0.302698	0.375	0.163	9.45E-09	H-cluster3
OGDH1	8.92E-13	0.428652	0.511	0.275	1.67E-08	H-cluster3
LTBP3	9.56E-13	0.361846	0.477	0.253	1.79E-08	H-cluster3

COL12A11	9.58E-13	0.572451	0.538	0.299	1.79E-08	H-cluster3
CCNI	1.01E-12	0.403795	0.504	0.274	1.89E-08	H-cluster3
FBLN1	1.12E-12	0.664867	0.712	0.565	2.09E-08	H-cluster3
LRP1	1.13E-12	0.542863	0.625	0.46	2.11E-08	H-cluster3
ZNF331	1.13E-12	0.453312	0.311	0.125	2.12E-08	H-cluster3
MYO1C	1.47E-12	0.261438	0.277	0.103	2.75E-08	H-cluster3
AMD1	1.75E-12	0.390024	0.261	0.097	3.28E-08	H-cluster3
FERMT2	1.84E-12	0.606053	0.606	0.379	3.45E-08	H-cluster3
PROCR1	2.05E-12	0.458912	0.595	0.361	3.83E-08	H-cluster3
CNN3	2.07E-12	0.310784	0.348	0.153	3.87E-08	H-cluster3
PNPLA8	2.45E-12	0.387294	0.326	0.141	4.59E-08	H-cluster3
LOXL11	2.56E-12	0.256306	0.333	0.136	4.79E-08	H-cluster3
PDE3A	2.58E-12	0.555819	0.678	0.476	4.83E-08	H-cluster3
NDRG1	2.81E-12	0.376045	0.485	0.267	5.25E-08	H-cluster3
ABLIM11	3.28E-12	0.360303	0.769	0.513	6.14E-08	H-cluster3
HDLBP	3.33E-12	0.348489	0.5	0.265	6.22E-08	H-cluster3
POLR2A	4.59E-12	0.328857	0.337	0.148	8.58E-08	H-cluster3
SGMS2	5.31E-12	0.490802	0.284	0.115	9.93E-08	H-cluster3
FHL1	5.34E-12	0.400772	0.432	0.219	9.99E-08	H-cluster3
SDK11	5.90E-12	0.262253	0.89	0.658	1.10E-07	H-cluster3
CCDC80	7.47E-12	0.802199	0.648	0.479	1.40E-07	H-cluster3
ELN1	7.95E-12	0.493083	0.572	0.342	1.49E-07	H-cluster3
HUNK1	8.18E-12	0.346852	0.394	0.185	1.53E-07	H-cluster3
NHSL11	8.68E-12	0.459576	0.61	0.369	1.62E-07	H-cluster3
ABCG1	8.72E-12	0.350362	0.265	0.103	1.63E-07	H-cluster3
PDLIM71	9.70E-12	0.305618	0.367	0.168	1.81E-07	H-cluster3
FCGRT	1.05E-11	0.280906	0.364	0.167	1.96E-07	H-cluster3
NFATC1	1.18E-11	0.408609	0.258	0.1	2.22E-07	H-cluster3
YWHAZ	1.23E-11	0.446339	0.561	0.335	2.31E-07	H-cluster3
CALU1	1.31E-11	0.313658	0.458	0.234	2.45E-07	H-cluster3
COL1A2	1.33E-11	0.769316	0.746	0.689	2.49E-07	H-cluster3
SOD2	1.48E-11	1.049462	0.455	0.25	2.77E-07	H-cluster3
SMC1A	1.66E-11	0.270112	0.265	0.103	3.11E-07	H-cluster3
YBX3	2.05E-11	0.417459	0.557	0.345	3.84E-07	H-cluster3
GAS71	2.43E-11	0.776459	0.799	0.625	4.55E-07	H-cluster3
EIF4G2	2.84E-11	0.2987	0.436	0.224	5.31E-07	H-cluster3
FAM107B1	3.09E-11	0.397112	0.413	0.208	5.77E-07	H-cluster3
HNRNPF	3.54E-11	0.33766	0.322	0.145	6.62E-07	H-cluster3
UGP2	4.12E-11	0.747835	0.5	0.287	7.70E-07	H-cluster3
DDX3X	4.20E-11	0.354576	0.447	0.24	7.85E-07	H-cluster3
ARMC9	4.64E-11	0.308123	0.36	0.171	8.69E-07	H-cluster3
BASP11	4.75E-11	0.336705	0.379	0.182	8.88E-07	H-cluster3
TSC22D2	5.16E-11	0.652778	0.473	0.261	9.65E-07	H-cluster3
KIAA14561	5.66E-11	0.411866	0.326	0.147	1.06E-06	H-cluster3
XYLT1	5.77E-11	0.717295	0.58	0.371	1.08E-06	H-cluster3
UBE2I	5.91E-11	0.343248	0.288	0.12	1.11E-06	H-cluster3
RPL31	6.71E-11	0.268025	0.28	0.114	1.25E-06	H-cluster3

SH3BP5	6.82E-11	0.440438	0.542	0.329	1.28E-06	H-cluster3
MORF4L2	7.75E-11	0.348467	0.439	0.232	1.45E-06	H-cluster3
MXRA8	8.16E-11	0.370514	0.477	0.276	1.53E-06	H-cluster3
HK1	8.32E-11	0.392836	0.273	0.113	1.56E-06	H-cluster3
JDP2	9.12E-11	0.391542	0.356	0.168	1.71E-06	H-cluster3
SKP1	9.53E-11	0.258899	0.383	0.188	1.78E-06	H-cluster3
KLF3	9.61E-11	0.440991	0.436	0.241	1.80E-06	H-cluster3
EIF3H	9.79E-11	0.316826	0.367	0.174	1.83E-06	H-cluster3
SLC2A3	1.08E-10	0.635862	0.28	0.125	2.01E-06	H-cluster3
TGFBR11	1.08E-10	0.325339	0.436	0.223	2.02E-06	H-cluster3
IL13RA1	1.10E-10	0.324039	0.379	0.185	2.06E-06	H-cluster3
RALY	1.12E-10	0.39399	0.432	0.231	2.10E-06	H-cluster3
SSC5D	1.23E-10	0.328727	0.292	0.128	2.30E-06	H-cluster3
SHISA61	1.29E-10	0.389048	0.265	0.11	2.41E-06	H-cluster3
UBA1	1.37E-10	0.30869	0.288	0.125	2.56E-06	H-cluster3
CPXM21	1.72E-10	0.351925	0.405	0.208	3.21E-06	H-cluster3
MORF4L1	1.88E-10	0.31696	0.473	0.266	3.51E-06	H-cluster3
MCL1	2.55E-10	0.362528	0.375	0.193	4.77E-06	H-cluster3
TMOD3	3.15E-10	0.333243	0.447	0.243	5.89E-06	H-cluster3
SMAD3	3.52E-10	0.407708	0.689	0.466	6.58E-06	H-cluster3
KPNA4	4.18E-10	0.31841	0.398	0.203	7.82E-06	H-cluster3
PSD31	4.48E-10	0.367207	0.674	0.423	8.39E-06	H-cluster3
ERCC1	5.27E-10	0.350671	0.322	0.151	9.86E-06	H-cluster3
SLC38A2	5.56E-10	0.387056	0.409	0.221	1.04E-05	H-cluster3
HECA	5.71E-10	0.344446	0.261	0.11	1.07E-05	H-cluster3
ZC3HAV1	6.17E-10	0.466953	0.424	0.234	1.15E-05	H-cluster3
IGFBP4	6.32E-10	0.27359	0.375	0.185	1.18E-05	H-cluster3
CAMK1D1	7.20E-10	0.311057	0.67	0.418	1.35E-05	H-cluster3
DCN	7.41E-10	0.820967	0.742	0.798	1.39E-05	H-cluster3
SRSF7	7.67E-10	0.305362	0.413	0.226	1.43E-05	H-cluster3
HERPUD1	7.68E-10	0.273872	0.299	0.131	1.44E-05	H-cluster3
SLC20A1	8.31E-10	0.341373	0.28	0.126	1.56E-05	H-cluster3
NDEL1	9.87E-10	0.369097	0.348	0.172	1.85E-05	H-cluster3
ACSL4	1.06E-09	1.040524	0.455	0.267	1.99E-05	H-cluster3
RAB7A	1.13E-09	0.467768	0.564	0.348	2.11E-05	H-cluster3
LRRC171	1.14E-09	0.287926	0.299	0.14	2.13E-05	H-cluster3
AGO2	1.15E-09	0.314281	0.462	0.258	2.14E-05	H-cluster3
ITGB1	1.19E-09	0.266245	0.504	0.3	2.23E-05	H-cluster3
FILIP1L	1.24E-09	0.438027	0.42	0.239	2.32E-05	H-cluster3
AHCYL1	1.30E-09	0.28557	0.383	0.196	2.44E-05	H-cluster3
REL	1.51E-09	0.542407	0.284	0.13	2.82E-05	H-cluster3
TNFRSF1A	1.75E-09	0.288581	0.307	0.144	3.28E-05	H-cluster3
SYNCRIP	2.07E-09	0.25975	0.326	0.158	3.88E-05	H-cluster3
COL5A1	2.24E-09	0.385119	0.557	0.355	4.19E-05	H-cluster3
SPTAN11	2.32E-09	0.355697	0.595	0.392	4.34E-05	H-cluster3
IQGAP1	2.53E-09	0.321167	0.67	0.463	4.73E-05	H-cluster3
KLF6	2.59E-09	0.39899	0.348	0.179	4.85E-05	H-cluster3

SLCO5A1	2.60E-09	0.286875	0.458	0.254	4.86E-05	H-cluster3
PTP4A2	3.76E-09	0.358186	0.432	0.247	7.04E-05	H-cluster3
RYBP	3.79E-09	0.485541	0.303	0.147	7.09E-05	H-cluster3
PABPC1	4.19E-09	0.379817	0.534	0.33	7.84E-05	H-cluster3
FAT1	4.31E-09	0.332023	0.292	0.138	8.07E-05	H-cluster3
IPO7	4.33E-09	0.350007	0.36	0.188	8.10E-05	H-cluster3
NFIX	4.64E-09	0.283739	0.564	0.365	8.68E-05	H-cluster3
SON	4.75E-09	0.2852	0.663	0.469	8.88E-05	H-cluster3
NUPR1	4.82E-09	0.280091	0.303	0.142	9.01E-05	H-cluster3
ATP2A2	4.90E-09	0.320269	0.455	0.255	9.17E-05	H-cluster3
RHOQ	5.21E-09	0.318233	0.443	0.248	9.74E-05	H-cluster3
ACTR3	5.84E-09	0.294862	0.424	0.234	0.000109	H-cluster3
CORO1C1	8.18E-09	0.276668	0.371	0.193	0.000153	H-cluster3
SF1	8.39E-09	0.285322	0.538	0.333	0.000157	H-cluster3
CYTH1	8.76E-09	0.34501	0.417	0.229	0.000164	H-cluster3
SRSF2	9.20E-09	0.376564	0.432	0.257	0.000172	H-cluster3
MSN	9.40E-09	0.3035	0.413	0.226	0.000176	H-cluster3
TMOD1	1.06E-08	0.296486	0.273	0.126	0.000198	H-cluster3
PXDNL1	1.07E-08	0.312692	0.47	0.271	0.0002	H-cluster3
TWIST2	1.12E-08	0.427448	0.519	0.325	0.000209	H-cluster3
MRC2	1.26E-08	0.364937	0.648	0.46	0.000235	H-cluster3
EIF3J	1.36E-08	0.26587	0.307	0.148	0.000255	H-cluster3
MYO1E1	1.63E-08	0.262943	0.462	0.265	0.000305	H-cluster3
GPX31	1.72E-08	0.253519	0.538	0.33	0.000321	H-cluster3
PHF20	1.92E-08	0.433143	0.451	0.273	0.00036	H-cluster3
INTS6	1.98E-08	0.434483	0.462	0.281	0.00037	H-cluster3
AGAP11	1.98E-08	0.336759	0.773	0.56	0.00037	H-cluster3
PXN1	2.04E-08	0.322828	0.492	0.294	0.000381	H-cluster3
MARCH3	2.10E-08	0.404796	0.549	0.34	0.000393	H-cluster3
TNFAIP2	2.38E-08	0.550321	0.447	0.26	0.000444	H-cluster3
SULF11	2.39E-08	0.341965	0.633	0.42	0.000448	H-cluster3
ITGAV	2.68E-08	0.347706	0.5	0.294	0.000501	H-cluster3
SPPL2A	2.84E-08	0.337888	0.47	0.281	0.00053	H-cluster3
HNRNPH3	2.84E-08	0.260558	0.504	0.304	0.000532	H-cluster3
UBE2B	2.88E-08	0.318974	0.489	0.301	0.000539	H-cluster3
MT-ND2	2.90E-08	0.261888	0.898	0.812	0.000542	H-cluster3
ADAMTS61	3.12E-08	0.381599	0.367	0.197	0.000583	H-cluster3
PCF11	3.22E-08	0.304266	0.417	0.243	0.000603	H-cluster3
VCL	3.42E-08	0.472899	0.53	0.337	0.000639	H-cluster3
ABCC11	3.42E-08	0.353847	0.561	0.362	0.00064	H-cluster3
SLC4A7	3.49E-08	0.847553	0.527	0.359	0.000653	H-cluster3
RAC1	5.10E-08	0.304149	0.367	0.205	0.000955	H-cluster3
PPP2CB	5.36E-08	0.285285	0.409	0.229	0.001002	H-cluster3
RIPK1	6.86E-08	0.298957	0.333	0.178	0.001283	H-cluster3
SUB1	6.95E-08	0.272948	0.33	0.174	0.001299	H-cluster3
TXNIP	7.42E-08	0.680222	0.587	0.461	0.001388	H-cluster3
ERRFI1	7.47E-08	0.30831	0.386	0.214	0.001398	H-cluster3

EMP1	8.11E-08	0.497355	0.485	0.314	0.001517	H-cluster3
GRB10	8.79E-08	0.399091	0.405	0.24	0.001644	H-cluster3
PMP22	9.21E-08	0.299075	0.53	0.362	0.001722	H-cluster3
A4GALT	9.35E-08	0.255182	0.277	0.133	0.001748	H-cluster3
HSPD1	1.00E-07	0.375249	0.261	0.127	0.001873	H-cluster3
RFX2	1.00E-07	0.486977	0.458	0.294	0.001874	H-cluster3
CLIP1	1.01E-07	0.574769	0.648	0.461	0.001886	H-cluster3
DRAM1	1.17E-07	0.342802	0.394	0.216	0.002184	H-cluster3
PCSK5	1.27E-07	0.405317	0.553	0.377	0.002377	H-cluster3
IL6ST1	1.32E-07	0.314273	0.667	0.516	0.002461	H-cluster3
L3MBTL41	1.33E-07	0.304863	0.326	0.173	0.002482	H-cluster3
TRIO1	1.36E-07	0.268733	0.837	0.689	0.002545	H-cluster3
GNB1	1.50E-07	0.454823	0.621	0.432	0.002808	H-cluster3
RUNX11	1.63E-07	0.369712	0.542	0.361	0.003051	H-cluster3
CMIP1	1.71E-07	0.338196	0.439	0.267	0.003202	H-cluster3
FLNB1	2.54E-07	0.356995	0.39	0.224	0.004754	H-cluster3
PTGIS1	2.69E-07	0.343366	0.667	0.49	0.005038	H-cluster3
GATAD2A	2.82E-07	0.326555	0.364	0.204	0.005278	H-cluster3
TJP1	3.34E-07	0.260188	0.371	0.206	0.006255	H-cluster3
MYO9B	3.78E-07	0.316268	0.466	0.289	0.007072	H-cluster3
MTHFD1L1	4.81E-07	0.349425	0.307	0.166	0.009005	H-cluster3
PHLDB1	4.89E-07	0.375655	0.572	0.395	0.009144	H-cluster3
ZFP36L1	4.89E-07	0.305408	0.511	0.331	0.009155	H-cluster3
GALNT121	5.19E-07	0.275844	0.265	0.134	0.009714	H-cluster3
ANXA11	5.63E-07	0.261624	0.364	0.214	0.010527	H-cluster3
CORO2B1	6.39E-07	0.383552	0.413	0.25	0.011946	H-cluster3
GPCPD1	7.15E-07	0.377557	0.436	0.279	0.013368	H-cluster3
PRKCA	7.25E-07	0.323297	0.511	0.337	0.013553	H-cluster3
WISP2	7.26E-07	0.523856	0.383	0.248	0.013573	H-cluster3
PRKAR2A	7.30E-07	0.333099	0.409	0.245	0.013654	H-cluster3
GNAI3	8.02E-07	0.359155	0.402	0.246	0.014994	H-cluster3
CDK17	9.01E-07	0.34701	0.439	0.277	0.016849	H-cluster3
RRAS21	9.93E-07	0.293118	0.409	0.245	0.018564	H-cluster3
WTAP	1.09E-06	0.343268	0.451	0.285	0.020342	H-cluster3
MT-ATP8	1.16E-06	0.288543	0.47	0.299	0.02177	H-cluster3
EFEMP1	1.30E-06	0.318845	0.545	0.391	0.024371	H-cluster3
AGAP3	1.44E-06	0.257647	0.261	0.137	0.026869	H-cluster3
PFKFB3	1.59E-06	0.324096	0.311	0.171	0.029656	H-cluster3
CD341	1.86E-06	0.280575	0.576	0.422	0.034871	H-cluster3
MAPK6	1.99E-06	0.278316	0.39	0.235	0.03713	H-cluster3
STX12	2.10E-06	0.26479	0.485	0.321	0.039294	H-cluster3
CYBRD1	2.12E-06	0.273867	0.572	0.412	0.039586	H-cluster3
SOAT1	2.25E-06	0.255359	0.295	0.161	0.042168	H-cluster3
USP12	2.67E-06	0.502335	0.462	0.31	0.050015	H-cluster3
PHLPP11	3.20E-06	0.260825	0.576	0.398	0.059812	H-cluster3
SORBS21	3.29E-06	0.369542	0.311	0.183	0.061606	H-cluster3
KIAA0232	4.27E-06	0.364594	0.432	0.279	0.079779	H-cluster3

BAZ1A	5.26E-06	0.352849	0.375	0.229	0.098464	H-cluster3
EPB41L31	6.10E-06	0.337358	0.644	0.485	0.114071	H-cluster3
ABR	8.07E-06	0.438484	0.462	0.316	0.150981	H-cluster3
DENND5A	8.18E-06	0.450594	0.515	0.366	0.152999	H-cluster3
GRK51	9.19E-06	0.346393	0.731	0.596	0.171922	H-cluster3
DISC1	9.31E-06	0.267486	0.288	0.165	0.174045	H-cluster3
GLUD1	1.04E-05	0.303968	0.39	0.25	0.193994	H-cluster3
BACH11	1.05E-05	0.320046	0.413	0.267	0.196248	H-cluster3
NFKB1	1.16E-05	1.251842	0.47	0.327	0.216965	H-cluster3
HIVEP2	1.25E-05	0.414261	0.58	0.424	0.233747	H-cluster3
RBMS11	1.38E-05	0.283672	0.837	0.702	0.258583	H-cluster3
ABL2	1.44E-05	0.592927	0.481	0.342	0.269727	H-cluster3
PRSS231	1.96E-05	0.272797	0.455	0.303	0.367297	H-cluster3
PAPSS2	2.42E-05	0.292289	0.345	0.216	0.451891	H-cluster3
PSEN1	3.31E-05	0.263178	0.458	0.313	0.618515	H-cluster3
IFRD1	3.42E-05	0.344456	0.326	0.205	0.639863	H-cluster3
CBLB	3.68E-05	0.350032	0.886	0.795	0.687398	H-cluster3
RAB8B	4.77E-05	0.257563	0.424	0.275	0.893053	H-cluster3
MARK1	6.40E-05	0.291781	0.394	0.255	1	H-cluster3
LHFPL2	7.16E-05	0.261731	0.322	0.198	1	H-cluster3
ATP2B11	8.04E-05	0.328305	0.447	0.302	1	H-cluster3
MAP3K8	9.21E-05	0.307738	0.371	0.252	1	H-cluster3
ADAM17	0.000102	0.415413	0.443	0.309	1	H-cluster3
ARL13B	0.000117	0.292208	0.261	0.158	1	H-cluster3
AKAP12	0.000127	0.308341	0.572	0.42	1	H-cluster3
ELL2	0.000146	0.564879	0.261	0.165	1	H-cluster3
LATS2	0.00015	0.273707	0.398	0.27	1	H-cluster3
BICD11	0.000168	0.293508	0.386	0.255	1	H-cluster3
C31	0.000174	0.417606	0.519	0.416	1	H-cluster3
CSNK1A1	0.000182	0.274392	0.64	0.489	1	H-cluster3
ULK1	0.000197	0.257526	0.292	0.185	1	H-cluster3
TRAF3	0.000216	0.272967	0.333	0.221	1	H-cluster3
PAPD7	0.000352	0.250503	0.269	0.165	1	H-cluster3
EML4	0.000474	0.266721	0.402	0.276	1	H-cluster3
SLC16A7	0.000476	0.315714	0.515	0.363	1	H-cluster3
KCTD20	0.000487	0.289125	0.288	0.185	1	H-cluster3
HNRNPU	0.000567	0.275929	0.617	0.496	1	H-cluster3
MCU	0.001028	0.252677	0.42	0.303	1	H-cluster3
GBE1	0.001484	0.289874	0.53	0.402	1	H-cluster3
LRRFIP1	0.002414	0.278551	0.273	0.187	1	H-cluster3
WWTR1	0.00248	0.261976	0.477	0.361	1	H-cluster3
EIF2AK3	0.002989	0.254336	0.269	0.186	1	H-cluster3
PHLDB21	0.003282	0.302629	0.576	0.461	1	H-cluster3
SAMD4A	0.007556	0.438093	0.636	0.536	1	H-cluster3

Extended Data Table 5

Pearson coefficient correlation of male subjects between RSPO2 (ng/ml) in plasma and following parameters

Serum RSPO2 level (ng/ml) correlation with	Age (years)	Body weight (m)	Height (m)	BMI (kg/m^2)	Visceral fat area (cm^2)	SC fat area (cm^2)	Glucose infusion rate clamp (mmol/kg/min)	% Body fat	Waist (cm)	Fasting plasma glucose (mmol/l)	HbA1c (%)	Fasting plasma insulin (pmol/l)	Total cholesterol (mmol/l)	HDL-Cholesterol (mmol/l)	LDL-Cholesterol (mmol/l)	Triglycerides (mmol/l)	Free fatty acids (mmol/l)	Leptin (ng/ml)
Spearman r																		
r	0.158	-0.309	0.082	-0.351	0.513	-0.325	-0.437	-0.342	-0.255	0.396	0.354	0.444	-0.015	-0.554	0.199	-0.322	0.411	0.032
95% confidence interval	-0.306 to 0.561	-0.661 to 0.155	-0.375 to 0.506	-0.686 to 0.109	0.091 to 0.779	-0.671 to 0.138	-0.7372 to 0.006917	-0.682 to 0.118	-0.627 to 0.212	-0.057 to 0.713	-0.105 to 0.689	0.002 to 0.741	-0.455 to 0.431	-0.801 to -0.148	-0.268 to 0.59	-0.670 to 0.141	-0.039 to 0.722	-0.416 to 0.469
P value																		
P (two-tailed)	0.495	0.172	0.725	0.119	0.017	0.151	0.048	0.129	0.266	0.076	0.116	0.044	0.949	0.009	0.388	0.155	0.064	0.889
P value summary	ns	ns	ns	ns	*	ns	*	ns	ns	ns	ns	*	ns	**	ns	ns	ns	ns
q value (FDR=0.05)	0.631	0.293	0.835	0.266	0.155	0.290	0.188	0.270	0.389	0.226	0.266	0.188	0.996	0.108	0.513	0.290	0.226	0.981
Number of XY Pairs	21	21	21	21	21	21	21	21	21	21	21	21	21	21	21	21	21	21

Continued

Serum RSPO2 level (ng/ml) correlation with	Adiponektin (µg/ml)	CrP (mg/dl)	IL-6 (pg/ml)	ASAT (µkat/l)	ALAT (µkat/l)	gGT (µkat/l)	MCP-1 (pg/ml)	Chemerin (ng/ml)	Progranulin (ng/ml)	Fetuin-A (µg/ml)	RBP4 (µg/ml)	Vaspin (ng/ml)	% Macs in omental fat	Mean adi size (pl)	Max adi size (pl)	HOMA-IR
Spearman r																
r	-0.251	0.399	0.273	0.252	0.211	0.554	0.088	0.127	0.467	0.460	0.594	0.027	0.363	0.312	0.361	0.478
95% confidence interval	-0.624 to 0.216	-0.053 to 0.715	-0.193 to 0.638	-0.215 to 0.625	-0.255 to 0.598	0.148 to 0.800	-0.369 to 0.511	-0.334 to 0.540	0.030 to 0.754	0.021 to 0.750	0.205 to 0.821	-0.421 to 0.464	-0.096 to 0.694	-0.151 to 0.663	-0.099 to 0.693	0.045 to 0.760
P value																
P (two-tailed)	0.2725	0.0733	0.2315	0.2715	0.358	0.0091	0.7035	0.5825	0.033	0.036	0.0046	0.9065	0.1062	0.1679	0.1083	0.0284
P value summary	ns	ns	ns	ns	ns	**	ns	ns	*	*	**	ns	ns	ns	ns	*
q value (FDR=0.05)	0.389	0.226	0.376	0.389	0.492	0.108	0.835	0.717	0.184	0.184	0.108	0.981	0.266	0.293	0.266	0.184
Number of XY Pairs	21	21	21	21	21	21	21	21	21	21	21	21	21	21	21	21

Extended Data Table 6

Pearson coefficient correlation of female subjects between RSPO2 (ng/ml) in plasma and following parameters

Serum RSPO2 level (ng/ml) correlation with	Age (years)	Body weight (m)	Height (m)	BMI (kg/m^2)	Visceral fat area (cm^2)	SC fat area (cm^2)	Glucose infusion rate clamp (mmol/kg/min)	% Body fat	Waist (cm)	Fasting plasma glucose (mmol/l)	HbA1c (%)	Fasting plasma insulin (pmol/l)	Total cholesterol (mmol/l)	HDL-Cholesterol (mmol/l)	LDL-Cholesterol (mmol/l)	Triglycerides (mmol/l)	Free fatty acids (mmol/l)
Spearman r																	
r	0.026	0.116	-0.035	0.157	-0.022	0.018	0.030	0.232	0.080	-0.128	0.094	0.057	0.405	0.069	0.183	0.120	0.006
95% confidence interval	-0.300 to 0.348	-0.217 to 0.424	-0.355 to 0.293	-0.177 to 0.458	-0.344 to 0.304	-0.308 to 0.340	-0.297 to 0.351	-0.100 to 0.517	-0.251 to 0.394	-0.434 to 0.205	-0.237 to 0.406	-0.272 to 0.374	0.093 to 0.644	-0.261 to 0.385	-0.150 to 0.479	-0.212 to 0.428	-0.319 to 0.330
P value																	
P (two-tailed)	0.873	0.484	0.834	0.341	0.894	0.912	0.854	0.156	0.628	0.438	0.568	0.730	0.011	0.674	0.265	0.467	0.969
P value summary	ns	ns	ns	ns	ns	ns	ns	ns	ns	ns	ns	ns	*	ns	ns	ns	ns
q value (FDR=0.05)	1	1	1	1	1	1	1	1	1	1	1	1	0.3784	1	1	1	1
Number of XY Pairs	39	39	39	39	39	39	39	39	39	39	39	39	39	39	39	39	39

Continued

Serum RSPO2 level (ng/ml) correlation with	Leptin (ng/ml)	Adiponektin (µg/ml)	CrP (mg/dl)	IL-6 (pg/ml)	ASAT (µkat/l)	ALAT (µkat/l)	gGT (µkat/l)	MCP-1 (pg/ml)	Chemerin (ng/ml)	Progranulin (ng/ml)	Fetuin-A (µg/ml)	RBP4 (µg/ml)	Vaspin (ng/ml)	% Macs in omental fat	Mean adi size (pl)	Max adi size (pl)	HOMA-IR
Spearman r																	
r	-0.225	-0.039	-0.051	0.036	0.048	-0.023	-0.003	0.237	0.222	-0.161	0.013	-0.062	0.047	0.056	0.119	-0.033	0.065
95% confidence interval	-0.512 to 0.107	-0.359 to 0.289	-0.369 to 0.278	-0.292 to 0.356	-0.280 to 0.367	-0.344 to 0.304	-0.327 to 0.321	-0.095 to 0.521	-0.110 to 0.509	-0.461 to 0.172	-0.313 to 0.336	-0.378 to 0.268	-0.282 to 0.365	-0.273 to 0.374	-0.214 to 0.427	-0.354 to 0.294	-0.265 to 0.382
P value																	
P (two-tailed)	0.169	0.813	0.756	0.828	0.771	0.891	0.984	0.147	0.175	0.327	0.939	0.709	0.778	0.735	0.471	0.841	0.693
P value summary	ns	ns	ns	ns	ns	ns	ns	ns	ns	ns	ns	ns	ns	ns	ns	ns	ns
q value (FDR=0.05)	1	1	1	1	1	1	1	1	1	1	1	1	1	1	1	1	1
Number of XY Pairs	39	39	39	39	39	39	39	39	39	39	39	39	39	39	39	39	39

Extened Data Table 7:maker genes for each cluster idenfied in 10x snRNA seq of ingWAT CAG-GFP and CAG-RSPO2 AAV infection HFD mice						
gene	p_val	avg_log	pct.1	pct.2	p_val_adj	cluster
Sorbs1	0	5.365	1.000	0.197	0	Adipocytes
Acsl1	0	5.302	0.995	0.114	0	Adipocytes
Npr3	0	4.909	0.995	0.084	0	Adipocytes
Ghr	0	4.879	1.000	0.6	0	Adipocytes
Car3	0	4.739	0.973	0.056	0	Adipocytes
Tenm4	0	4.718	1.000	0.329	0	Adipocytes
Prkar2b	0	4.711	0.999	0.174	0	Adipocytes
Cidec	0	4.212	0.994	0.064	0	Adipocytes
Lpgat1	0	4.129	0.995	0.251	0	Adipocytes
Tshr	0	4.123	0.981	0.085	0	Adipocytes
Mageb18	0	4.060	0.721	0.084	0	Adipocytes
Gm42906	0	4.007	0.921	0.08	0	Adipocytes
Tmem45b	0	3.943	0.941	0.1	0	Adipocytes
Mpp7	0	3.839	0.988	0.203	0	Adipocytes
Sntb1	0	3.815	0.995	0.109	0	Adipocytes
Hacd2	0	3.774	0.992	0.187	0	Adipocytes
Mmd	0	3.774	0.985	0.093	0	Adipocytes
Ankef1	0	3.716	0.991	0.059	0	Adipocytes
Fabp4	0	3.713	0.997	0.396	0	Adipocytes
Nnat	0	3.667	0.839	0.121	0	Adipocytes
Cav1	0	3.579	0.985	0.094	0	Adipocytes
Chpt1	0	3.516	0.978	0.124	0	Adipocytes
Fry	0	3.515	1.000	0.427	0	Adipocytes
Pnpla3	0	3.481	0.952	0.057	0	Adipocytes
Plin4	0	3.440	0.985	0.063	0	Adipocytes
Prr16	0	3.382	0.978	0.308	0	Adipocytes
Nrg4	0	3.365	0.974	0.084	0	Adipocytes
Slc1a5	0	3.337	0.997	0.411	0	Adipocytes
Adipor2	0	3.306	0.984	0.293	0	Adipocytes
Sik2	0	3.303	0.998	0.572	0	Adipocytes
Pcx	0	3.223	0.962	0.05	0	Adipocytes
Smoc1	0	3.222	0.952	0.076	0	Adipocytes
Pparg	0	3.208	0.997	0.533	0	Adipocytes
Tmem135	0	3.142	0.993	0.546	0	Adipocytes
Sgcd	0	3.108	0.998	0.552	0	Adipocytes
Bckdhb	0	3.075	0.999	0.475	0	Adipocytes
Tspan12	0	3.066	0.968	0.128	0	Adipocytes
Dgat2	0	3.056	0.933	0.041	0	Adipocytes
Plin1	0	3.052	0.977	0.063	0	Adipocytes
Pde3b	0	3.033	0.998	0.514	0	Adipocytes
Fam126b	0	3.011	0.979	0.249	0	Adipocytes
Lipe	0	2.947	0.982	0.087	0	Adipocytes
Eepd1	0	2.938	0.929	0.29	0	Adipocytes
Fgf14	0	2.925	0.534	0.077	0	Adipocytes
Me1	0	2.924	0.893	0.154	0	Adipocytes
Atp1a2	0	2.897	0.963	0.066	0	Adipocytes
Myl1	0	2.882	0.960	0.088	0	Adipocytes
Art3	0	2.878	0.935	0.081	0	Adipocytes
Prkd1	0	2.865	0.999	0.52	0	Adipocytes
Acox1	0	2.865	0.979	0.291	0	Adipocytes
Mycbp2	0	2.842	0.999	0.831	0	Adipocytes
Fam13a	0	2.751	0.876	0.298	0	Adipocytes
Acvr1c	0	2.748	0.921	0.056	0	Adipocytes
Ryr2	0	2.725	0.877	0.091	0	Adipocytes
Ralgapa2	0	2.719	0.996	0.558	0	Adipocytes
Slc24a3	0	2.705	0.991	0.536	0	Adipocytes
Gnai1	0	2.703	0.923	0.087	0	Adipocytes
Pdzrn3	0	2.682	0.983	0.366	0	Adipocytes

Mgll	0	2.636	0.974	0.371	0	Adipocytes
Grb14	0	2.617	0.884	0.229	0	Adipocytes
Fmr1	0	2.615	0.867	0.282	0	Adipocytes
Slc1a3	0	2.600	0.886	0.172	0	Adipocytes
Palmd	0	2.592	0.943	0.057	0	Adipocytes
Pde4d	0	2.585	0.995	0.591	0	Adipocytes
Plcb1	0	2.583	0.998	0.52	0	Adipocytes
Rgs7	0	2.582	0.847	0.302	0	Adipocytes
Slc7a10	0	2.560	0.919	0.051	0	Adipocytes
Rgcc	0	2.534	0.947	0.058	0	Adipocytes
Apol6	0	2.519	0.934	0.047	0	Adipocytes
Dram1	0	2.512	0.942	0.153	0	Adipocytes
Adam12	0	2.508	0.955	0.467	0	Adipocytes
Lep	0	2.477	0.897	0.036	0	Adipocytes
Trhde	0	2.469	0.806	0.124	0	Adipocytes
Sox6	0	2.455	0.928	0.11	0	Adipocytes
Fgf10	0	2.451	0.894	0.287	0	Adipocytes
Enpp2	0	2.442	0.916	0.256	0	Adipocytes
Lhfpl2	0	2.421	0.910	0.394	0	Adipocytes
Cat	0	2.398	0.926	0.169	0	Adipocytes
Pfkfb3	0	2.387	0.932	0.355	0	Adipocytes
Bcar3	0	2.370	0.951	0.346	0	Adipocytes
Rasgrf2	0	2.349	0.897	0.115	0	Adipocytes
Kcnc2	0	2.322	0.669	0.245	0	Adipocytes
Ptchd4	0	2.312	0.708	0.094	0	Adipocytes
C9orf72	0	2.301	0.923	0.209	0	Adipocytes
Trabd2b	0	2.299	0.827	0.28	0	Adipocytes
Nrip1	0	2.282	0.997	0.661	0	Adipocytes
Tln2	0	2.243	0.944	0.391	0	Adipocytes
Slc36a2	0	2.236	0.851	0.041	0	Adipocytes
4931428L18Ril	0	2.228	0.842	0.06	0	Adipocytes
Acss2	0	2.216	0.761	0.095	0	Adipocytes
Nedd4l	0	2.216	0.979	0.649	0	Adipocytes
Slc27a1	0	2.206	0.937	0.364	0	Adipocytes
Epn2	0	2.200	0.976	0.487	0	Adipocytes
Snhg11	0	2.191	0.881	0.224	0	Adipocytes
Gm20532	0	2.190	0.792	0.041	0	Adipocytes
Magi1	0	2.168	0.959	0.489	0	Adipocytes
Optn	0	2.165	0.907	0.186	0	Adipocytes
Cacna1e	0	2.155	0.722	0.118	0	Adipocytes
Cobll1	0	2.149	0.930	0.364	0	Adipocytes
Adcy5	0	2.145	0.852	0.167	0	Adipocytes
Lgals12	0	2.143	0.891	0.047	0	Adipocytes
Ptprd	0	2.130	0.991	0.533	0	Adipocytes
Pck1	0	2.127	0.709	0.076	0	Adipocytes
Med12l	0	2.124	0.871	0.075	0	Adipocytes
Cd36	0	2.110	0.999	0.524	0	Adipocytes
Tsc22d3	0	2.096	0.952	0.46	0	Adipocytes
Cavin2	0	2.061	0.881	0.098	0	Adipocytes
Ces1d	0	2.054	0.810	0.029	0	Adipocytes
Zfp423	0	2.047	0.945	0.431	0	Adipocytes
Peg3	0	2.031	0.753	0.183	0	Adipocytes
G0s2	0	2.016	0.841	0.022	0	Adipocytes
Hipk2	0	2.016	0.985	0.566	0	Adipocytes
Tns1	0	2.011	0.994	0.839	0	Adipocytes
Ywhab	0	2.008	0.915	0.277	0	Adipocytes
Lpin1	0	2.007	0.911	0.308	0	Adipocytes
Pfkfb1	0	2.006	0.874	0.067	0	Adipocytes
2810004N23Ri	0	1.996	0.858	0.226	0	Adipocytes
Txlng	0	1.994	0.978	0.589	0	Adipocytes
Acss3	0	1.984	0.981	0.548	0	Adipocytes
Ptger3	0	1.982	0.760	0.087	0	Adipocytes

Pde7a	0	1.972	0.955	0.67	0	Adipocytes
Dst	0	1.963	0.997	0.608	0	Adipocytes
Mical3	0	1.950	0.963	0.397	0	Adipocytes
Cdo1	0	1.948	0.755	0.039	0	Adipocytes
Tmem120b	0	1.944	0.861	0.191	0	Adipocytes
Ctdspl	0	1.917	0.946	0.463	0	Adipocytes
Pacrg	0	1.893	0.881	0.363	0	Adipocytes
Hmgcs1	0	1.891	0.810	0.123	0	Adipocytes
Por	0	1.887	0.901	0.318	0	Adipocytes
Cnnm2	0	1.879	0.917	0.554	0	Adipocytes
Abcd2	0	1.879	0.915	0.45	0	Adipocytes
Efna5	0	1.877	0.990	0.592	0	Adipocytes
Sybu	0	1.867	0.799	0.044	0	Adipocytes
Lama4	0	1.865	0.993	0.599	0	Adipocytes
Cpeb4	0	1.856	0.969	0.534	0	Adipocytes
Negr1	0	1.851	0.982	0.724	0	Adipocytes
Trp53inp2	0	1.839	0.817	0.04	0	Adipocytes
Bnip3	0	1.836	0.799	0.079	0	Adipocytes
Ctnna3	0	1.835	0.610	0.206	0	Adipocytes
Erbb4	0	1.832	0.528	0.085	0	Adipocytes
Apex2	0	1.829	0.912	0.517	0	Adipocytes
Mlxipl	0	1.823	0.801	0.053	0	Adipocytes
Esrrg	0	1.812	0.678	0.14	0	Adipocytes
Acyp2	0	1.803	0.973	0.623	0	Adipocytes
Ptprm	0	1.789	0.997	0.751	0	Adipocytes
Vwc2l	0	1.772	0.545	0.033	0	Adipocytes
Sorbs2	0	1.768	0.559	0.088	0	Adipocytes
Ccdc3	0	1.766	0.796	0.27	0	Adipocytes
Aox1	0	1.760	0.923	0.403	0	Adipocytes
Anxa2	0	1.757	0.941	0.471	0	Adipocytes
Tmem120a	0	1.756	0.802	0.081	0	Adipocytes
Thrb	0	1.754	0.992	0.779	0	Adipocytes
Scd1	0	1.753	0.678	0.169	0	Adipocytes
Col15a1	0	1.752	0.823	0.331	0	Adipocytes
Glb1l2	0	1.751	0.796	0.037	0	Adipocytes
Sort1	0	1.744	0.883	0.287	0	Adipocytes
Slc22a3	0	1.744	0.811	0.026	0	Adipocytes
Heph	0	1.743	0.766	0.042	0	Adipocytes
Sipa1l1	0	1.730	0.953	0.517	0	Adipocytes
Sh3pxd2a	0	1.723	0.885	0.492	0	Adipocytes
Clybl	0	1.717	0.931	0.575	0	Adipocytes
Cd1d1	0	1.715	0.773	0.034	0	Adipocytes
Large1	0	1.715	0.992	0.829	0	Adipocytes
Rnf24	0	1.692	0.828	0.244	0	Adipocytes
Foxo1	0	1.685	0.972	0.618	0	Adipocytes
Gpat3	0	1.680	0.765	0.05	0	Adipocytes
Ifrd1	0	1.680	0.907	0.482	0	Adipocytes
Lpl	0	1.679	0.983	0.514	0	Adipocytes
Dgat1	0	1.670	0.820	0.079	0	Adipocytes
Lgr4	0	1.664	0.903	0.374	0	Adipocytes
Plaat3	0	1.653	0.775	0.119	0	Adipocytes
Pdzd2	0	1.641	0.967	0.467	0	Adipocytes
Trarg1	0	1.632	0.804	0.067	0	Adipocytes
Adrb3	0	1.631	0.656	0.05	0	Adipocytes
Dusp10	0	1.625	0.765	0.274	0	Adipocytes
Pkp2	0	1.616	0.778	0.226	0	Adipocytes
Ptprk	0	1.599	0.797	0.367	0	Adipocytes
Shroom3	0	1.599	0.737	0.235	0	Adipocytes
Fggy	0	1.588	0.894	0.456	0	Adipocytes
Pmepa1	0	1.587	0.911	0.348	0	Adipocytes
Car5b	0	1.585	0.758	0.155	0	Adipocytes
Fam214a	0	1.583	0.953	0.686	0	Adipocytes

Adra1a	0	1.575	0.754	0.231	0	Adipocytes
Larp1b	0	1.568	0.853	0.37	0	Adipocytes
Tead1	0	1.556	0.972	0.625	0	Adipocytes
Ephx2	0	1.555	0.759	0.051	0	Adipocytes
Ccdc80	0	1.554	0.960	0.454	0	Adipocytes
Arap2	0	1.550	0.804	0.174	0	Adipocytes
Slc22a23	0	1.546	0.782	0.317	0	Adipocytes
Aplp2	0	1.541	0.962	0.444	0	Adipocytes
Rgs7bp	0	1.540	0.747	0.35	0	Adipocytes
Ntm	0	1.539	0.634	0.022	0	Adipocytes
Prelp	0	1.529	0.813	0.118	0	Adipocytes
Msrb3	0	1.524	0.942	0.434	0	Adipocytes
Irak2	0	1.523	0.895	0.363	0	Adipocytes
Arhgap29	0	1.523	0.942	0.496	0	Adipocytes
Snta1	0	1.511	0.842	0.432	0	Adipocytes
Slc5a3	0	1.511	0.815	0.291	0	Adipocytes
Synpo2	0	1.505	0.760	0.188	0	Adipocytes
Acacb	0	1.498	0.720	0.131	0	Adipocytes
Ptprg	0	1.497	0.989	0.637	0	Adipocytes
Macrod2	0	1.494	0.888	0.465	0	Adipocytes
Nap1l1	0	1.472	0.845	0.289	0	Adipocytes
Dpyd	0	1.463	0.907	0.558	0	Adipocytes
Vcl	0	1.451	0.930	0.52	0	Adipocytes
Fhit	0	1.449	0.978	0.797	0	Adipocytes
Prdm16	0	1.446	0.793	0.279	0	Adipocytes
Lctl	0	1.438	0.640	0.031	0	Adipocytes
Gbe1	0	1.436	0.893	0.522	0	Adipocytes
Ppip5k1	0	1.416	0.872	0.474	0	Adipocytes
Wwtr1	0	1.414	0.917	0.475	0	Adipocytes
Fign	0	1.400	0.839	0.39	0	Adipocytes
Ptpn14	0	1.398	0.788	0.249	0	Adipocytes
Mme	0	1.397	0.832	0.309	0	Adipocytes
Cgnl1	0	1.394	0.824	0.349	0	Adipocytes
Retn	0	1.388	0.692	0.011	0	Adipocytes
Ahcyl2	0	1.388	0.976	0.582	0	Adipocytes
Col4a1	0	1.376	0.947	0.462	0	Adipocytes
Adamts9	0	1.366	0.698	0.163	0	Adipocytes
Col4a2	0	1.362	0.918	0.395	0	Adipocytes
Svep1	0	1.353	0.918	0.586	0	Adipocytes
Ntrk2	0	1.340	0.825	0.362	0	Adipocytes
Gprin3	0	1.335	0.577	0.054	0	Adipocytes
Rad18	0	1.335	0.805	0.424	0	Adipocytes
Slc16a10	0	1.334	0.936	0.541	0	Adipocytes
Itga7	0	1.323	0.710	0.019	0	Adipocytes
Xkr4	0	1.305	0.776	0.428	0	Adipocytes
Dmd	0	1.303	0.851	0.501	0	Adipocytes
Fgf13	0	1.299	0.784	0.373	0	Adipocytes
Hk2	0	1.291	0.733	0.114	0	Adipocytes
Lrrc4c	0	1.290	0.510	0.097	0	Adipocytes
Dlg2	0	1.285	0.750	0.418	0	Adipocytes
Plxna2	0	1.275	0.816	0.493	0	Adipocytes
Acer2	0	1.268	0.683	0.123	0	Adipocytes
Aoc3	0	1.267	0.716	0.032	0	Adipocytes
Bcap31	0	1.262	0.796	0.228	0	Adipocytes
Slc16a12	0	1.262	0.641	0.06	0	Adipocytes
Itga6	0	1.257	0.678	0.208	0	Adipocytes
Ephb2	0	1.248	0.733	0.273	0	Adipocytes
Cachd1	0	1.247	0.881	0.518	0	Adipocytes
Tspan18	0	1.240	0.731	0.27	0	Adipocytes
Cecr2	0	1.240	0.656	0.107	0	Adipocytes
Aldh1a7	0	1.238	0.569	0.013	0	Adipocytes
Ell2	0	1.227	0.890	0.533	0	Adipocytes

Zbtb7c	0	1.223	0.831	0.446	0	Adipocytes
Fzd4	0	1.221	0.753	0.149	0	Adipocytes
Ccdc85a	0	1.218	0.561	0.197	0	Adipocytes
Jazf1	0	1.212	0.818	0.468	0	Adipocytes
Aldh6a1	0	1.211	0.692	0.1	0	Adipocytes
Clmn	0	1.207	0.709	0.248	0	Adipocytes
Ppp1r9a	0	1.197	0.895	0.659	0	Adipocytes
Angptl4	0	1.197	0.768	0.185	0	Adipocytes
Jph1	0	1.190	0.689	0.269	0	Adipocytes
Fads3	0	1.182	0.687	0.033	0	Adipocytes
Adipoq	0	1.181	0.640	0.004	0	Adipocytes
Bche	0	1.180	0.627	0.02	0	Adipocytes
Mcam	0	1.177	0.660	0.021	0	Adipocytes
Fam241a	0	1.171	0.715	0.187	0	Adipocytes
Mest	0	1.171	0.484	0.001	0	Adipocytes
Slc2a13	0	1.158	0.800	0.431	0	Adipocytes
Gpd1	0	1.157	0.635	0.017	0	Adipocytes
Fgf1	0	1.153	0.635	0.206	0	Adipocytes
March3	0	1.153	0.968	0.82	0	Adipocytes
Prxl2a	0	1.152	0.687	0.033	0	Adipocytes
Tcaf1	0	1.152	0.779	0.392	0	Adipocytes
Grid1	0	1.150	0.489	0.043	0	Adipocytes
A530016L24Ri	0	1.148	0.624	0.017	0	Adipocytes
Mgst1	0	1.145	0.752	0.171	0	Adipocytes
Dbi	0	1.139	0.637	0.13	0	Adipocytes
Vegfa	0	1.139	0.755	0.206	0	Adipocytes
Poln	0	1.135	0.649	0.241	0	Adipocytes
Asph	0	1.130	0.957	0.733	0	Adipocytes
Nr3c2	0	1.129	0.887	0.66	0	Adipocytes
Irs1	0	1.109	0.857	0.404	0	Adipocytes
Bcl2	0	1.108	0.890	0.586	0	Adipocytes
Kcns3	0	1.104	0.643	0.05	0	Adipocytes
B3galt2	0	1.103	0.645	0.102	0	Adipocytes
Tmem182	0	1.102	0.632	0.013	0	Adipocytes
Mmp28	0	1.095	0.596	0.064	0	Adipocytes
Adhfe1	0	1.095	0.678	0.339	0	Adipocytes
Gm42892	0	1.085	0.621	0.053	0	Adipocytes
Noct	0	1.084	0.806	0.391	0	Adipocytes
Sycp3	0	1.083	0.589	0.086	0	Adipocytes
Pag1	0	1.077	0.758	0.393	0	Adipocytes
Mrap	0	1.071	0.640	0.015	0	Adipocytes
Gpc4	0	1.059	0.670	0.283	0	Adipocytes
Itpr1	0	1.045	0.920	0.564	0	Adipocytes
Cd59a	0	1.043	0.617	0.029	0	Adipocytes
Unc79	0	1.039	0.552	0.054	0	Adipocytes
Igf1	0	1.030	0.905	0.562	0	Adipocytes
Chd7	0	1.029	0.796	0.333	0	Adipocytes
Xkr6	0	1.028	0.644	0.316	0	Adipocytes
Mpc1	0	1.022	0.780	0.332	0	Adipocytes
Ighm	0	1.015	0.607	0.18	0	Adipocytes
Pik3cb	0	0.994	0.778	0.294	0	Adipocytes
A830018L16Ri	0	0.993	0.553	0.041	0	Adipocytes
9330159F19Ri	0	0.985	0.623	0.092	0	Adipocytes
Myo1d	0	0.983	0.699	0.348	0	Adipocytes
Syne1	0	0.981	0.926	0.669	0	Adipocytes
Fhod3	0	0.974	0.594	0.105	0	Adipocytes
Klb	0	0.952	0.559	0.02	0	Adipocytes
Palld	0	0.943	0.829	0.524	0	Adipocytes
Reep5	0	0.929	0.717	0.259	0	Adipocytes
Gk	0	0.928	0.596	0.269	0	Adipocytes
Serpine1	0	0.928	0.467	0.096	0	Adipocytes
Pde7b	0	0.926	0.957	0.762	0	Adipocytes

Cavin1	0	0.922	0.685	0.166	0	Adipocytes
Stox2	0	0.922	0.769	0.409	0	Adipocytes
Hebp1	0	0.917	0.623	0.163	0	Adipocytes
Tes	0	0.905	0.677	0.263	0	Adipocytes
Ctcfl	0	0.901	0.597	0.053	0	Adipocytes
Tbc1d4	0	0.894	0.941	0.736	0	Adipocytes
Scn8a	0	0.890	0.540	0.21	0	Adipocytes
Mylk	0	0.888	0.513	0.084	0	Adipocytes
Exd1	0	0.884	0.587	0.19	0	Adipocytes
Slc5a7	0	0.873	0.401	0.001	0	Adipocytes
Rasl12	0	0.866	0.563	0.129	0	Adipocytes
Slc12a2	0	0.863	0.605	0.162	0	Adipocytes
Flacc1	0	0.862	0.497	0.035	0	Adipocytes
Tkt	0	0.861	0.644	0.221	0	Adipocytes
Pex5l	0	0.858	0.394	0.066	0	Adipocytes
Trim67	0	0.855	0.473	0.022	0	Adipocytes
Scp2	0	0.845	0.606	0.145	0	Adipocytes
Pabpc1l	0	0.842	0.505	0.011	0	Adipocytes
Aqp7	0	0.839	0.558	0.019	0	Adipocytes
Thrsp	0	0.828	0.411	0.004	0	Adipocytes
Mc2r	0	0.819	0.501	0.04	0	Adipocytes
Gpr156	0	0.801	0.428	0.024	0	Adipocytes
Adora1	0	0.793	0.511	0.035	0	Adipocytes
Dysf	0	0.786	0.529	0.142	0	Adipocytes
Laptm4b	0	0.786	0.548	0.109	0	Adipocytes
Cav2	0	0.785	0.551	0.063	0	Adipocytes
Tmeff1	0	0.766	0.600	0.185	0	Adipocytes
Mdh1	0	0.765	0.595	0.151	0	Adipocytes
Itga1	0	0.759	0.533	0.133	0	Adipocytes
Kcnb1	0	0.750	0.452	0.05	0	Adipocytes
Itgad	0	0.748	0.333	0.008	0	Adipocytes
Tcim	0	0.741	0.476	0.032	0	Adipocytes
Ranbp17	0	0.739	0.626	0.249	0	Adipocytes
Cyp2e1	0	0.737	0.307	0.013	0	Adipocytes
Mgst3	0	0.711	0.510	0.086	0	Adipocytes
Taldo1	0	0.695	0.579	0.168	0	Adipocytes
Grin2b	0	0.682	0.451	0.024	0	Adipocytes
Ifi27	0	0.676	0.560	0.199	0	Adipocytes
Wdr93	0	0.672	0.456	0.014	0	Adipocytes
Pclo	0	0.671	0.472	0.043	0	Adipocytes
Cux2	0	0.664	0.454	0.044	0	Adipocytes
Klhdc7a	0	0.659	0.440	0.003	0	Adipocytes
5033425B01Ri	0	0.653	0.446	0.016	0	Adipocytes
Zdhhc2	0	0.652	0.499	0.193	0	Adipocytes
Rasd1	0	0.638	0.436	0.004	0	Adipocytes
Fam83a	0	0.636	0.437	0.026	0	Adipocytes
Btg1	0	0.634	0.619	0.218	0	Adipocytes
Kcnj15	0	0.611	0.307	0.002	0	Adipocytes
Marc1	0	0.593	0.398	0.015	0	Adipocytes
Syt12	0	0.585	0.384	0.012	0	Adipocytes
Mettl24	0	0.578	0.436	0.113	0	Adipocytes
Tph2	0	0.576	0.358	0.023	0	Adipocytes
Crtac1	0	0.567	0.436	0.003	0	Adipocytes
Ces1f	0	0.563	0.361	0.042	0	Adipocytes
Gpc5	0	0.558	0.307	0.01	0	Adipocytes
Efcab8	0	0.558	0.389	0.04	0	Adipocytes
Bicdl1	0	0.503	0.415	0.03	0	Adipocytes
Dpf3	5.96E-308	0.786	0.643	0.344	1.19E-304	Adipocytes
Sesn2	6.23E-301	0.785	0.500	0.193	1.25E-297	Adipocytes
Nabp1	1.07E-299	0.839	0.685	0.299	2.15E-296	Adipocytes
Mid1	3.64E-297	1.254	0.959	0.662	7.28E-294	Adipocytes
Itih5	2.44E-287	0.883	0.895	0.49	4.88E-284	Adipocytes

Auts2	4.28E-286	0.926	0.907	0.627	8.57E-283	Adipocytes
Aldh1a1	1.29E-281	0.974	0.955	0.697	2.59E-278	Adipocytes
Arhgap26	7.99E-280	0.827	0.619	0.299	1.60E-276	Adipocytes
Ldha	6.18E-278	0.535	0.508	0.174	1.24E-274	Adipocytes
4931406P16Ri	3.52E-277	1.309	0.813	0.576	7.05E-274	Adipocytes
Nkd1	3.61E-272	0.568	0.468	0.154	7.22E-269	Adipocytes
Col5a3	7.61E-267	1.173	0.814	0.524	1.52E-263	Adipocytes
Gcnt2	4.27E-264	0.807	0.544	0.25	8.53E-261	Adipocytes
Atp5f1	1.19E-263	0.557	0.588	0.261	2.39E-260	Adipocytes
Met	2.52E-257	0.812	0.527	0.228	5.04E-254	Adipocytes
Hivep3	1.13E-252	0.901	0.874	0.656	2.26E-249	Adipocytes
Pgd	7.84E-250	0.550	0.566	0.26	1.57E-246	Adipocytes
Rin2	1.18E-243	0.930	0.892	0.71	2.36E-240	Adipocytes
Nlrc5	1.50E-243	0.714	0.673	0.398	2.99E-240	Adipocytes
Matn2	3.42E-243	0.954	0.499	0.193	6.83E-240	Adipocytes
Adcy6	1.14E-237	0.578	0.540	0.255	2.28E-234	Adipocytes
Oxct1	3.20E-233	0.859	0.802	0.473	6.39E-230	Adipocytes
Dok6	3.18E-232	1.133	0.601	0.395	6.36E-229	Adipocytes
Angpt1	3.69E-232	0.932	0.776	0.487	7.39E-229	Adipocytes
Adamts5	2.17E-231	0.808	0.948	0.645	4.34E-228	Adipocytes
Clstn2	1.64E-230	0.775	0.478	0.204	3.29E-227	Adipocytes
Fam184a	2.86E-229	0.648	0.466	0.183	5.72E-226	Adipocytes
Atp6v0a1	1.65E-228	0.571	0.932	0.615	3.31E-225	Adipocytes
Anxa5	6.25E-219	0.571	0.628	0.312	1.25E-215	Adipocytes
Grhl1	1.95E-217	0.547	0.371	0.068	3.90E-214	Adipocytes
Rbm47	3.06E-217	0.564	0.927	0.54	6.11E-214	Adipocytes
Ccdc152	3.88E-217	0.721	0.461	0.214	7.75E-214	Adipocytes
Eda	1.66E-215	0.567	0.891	0.67	3.32E-212	Adipocytes
Clec3b	5.52E-215	-1.933	0.150	0.554	1.10E-211	Adipocytes
Pde1a	2.19E-213	0.798	0.756	0.453	4.39E-210	Adipocytes
Dcn	5.41E-210	-2.500	0.350	0.659	1.08E-206	Adipocytes
Erc2	2.07E-204	0.570	0.409	0.147	4.14E-201	Adipocytes
Ttyh2	6.66E-203	0.618	0.652	0.418	1.33E-199	Adipocytes
Arid5b	1.08E-202	0.698	0.887	0.67	2.15E-199	Adipocytes
Dnajc6	3.30E-198	0.656	0.265	0.026	6.61E-195	Adipocytes
Net1	4.84E-197	0.521	0.391	0.158	9.69E-194	Adipocytes
Col1a2	1.51E-193	-2.317	0.361	0.668	3.03E-190	Adipocytes
Tank	1.16E-190	0.603	0.866	0.611	2.32E-187	Adipocytes
P2ry14	1.55E-175	0.561	0.520	0.299	3.10E-172	Adipocytes
Sema5a	3.14E-174	0.711	0.589	0.333	6.27E-171	Adipocytes
Kcnt2	9.15E-160	-1.273	0.263	0.644	1.83E-156	Adipocytes
Fbln2	2.76E-158	-1.349	0.187	0.493	5.52E-155	Adipocytes
Nkain2	2.59E-155	1.252	0.519	0.284	5.17E-152	Adipocytes
Clic4	1.47E-153	0.633	0.767	0.528	2.95E-150	Adipocytes
Kitl	3.03E-150	-0.907	0.436	0.759	6.05E-147	Adipocytes
Nos1ap	4.57E-149	0.768	0.646	0.471	9.14E-146	Adipocytes
Col3a1	1.79E-145	-2.394	0.518	0.768	3.57E-142	Adipocytes
Col14a1	6.74E-145	-2.134	0.412	0.707	1.35E-141	Adipocytes
Gpt2	9.75E-145	0.760	0.540	0.339	1.95E-141	Adipocytes
Pdk4	2.35E-144	0.662	0.418	0.255	4.70E-141	Adipocytes
Col1a1	8.52E-142	-1.792	0.239	0.537	1.70E-138	Adipocytes
Btc	2.79E-141	0.564	0.297	0.057	5.59E-138	Adipocytes
Nox4	5.20E-136	-2.357	0.465	0.732	1.04E-132	Adipocytes
mt-Nd6	3.06E-133	-1.074	0.472	0.726	6.12E-130	Adipocytes
Nr1h4	8.15E-133	-0.930	0.188	0.518	1.63E-129	Adipocytes
Cenpu	1.33E-130	0.509	0.401	0.259	2.66E-127	Adipocytes
Hp	7.94E-128	0.529	0.290	0.058	1.59E-124	Adipocytes
Gask1b	2.39E-127	0.650	0.665	0.485	4.78E-124	Adipocytes
St6galnac5	4.17E-127	1.167	0.446	0.271	8.35E-124	Adipocytes
Calcrl	2.39E-123	-1.238	0.389	0.686	4.78E-120	Adipocytes
Sugct	3.56E-122	0.720	0.627	0.528	7.13E-119	Adipocytes
Pcdh9	2.89E-120	-1.957	0.335	0.634	5.78E-117	Adipocytes

Sctr	7.67E-120	0.580	0.251	0.022	1.53E-116	Adipocytes
Zfp385b	1.58E-118	0.716	0.491	0.311	3.16E-115	Adipocytes
Pitpnc1	2.70E-117	-1.650	0.610	0.739	5.41E-114	Adipocytes
Mfap5	3.13E-112	-1.739	0.309	0.555	6.25E-109	Adipocytes
Prkg1	2.76E-108	-1.865	0.495	0.753	5.53E-105	Adipocytes
Sntg2	1.13E-105	0.544	0.461	0.305	2.26E-102	Adipocytes
Lvrn	3.74E-105	-1.356	0.297	0.582	7.47E-102	Adipocytes
Adamtsl1	6.69E-104	-2.010	0.542	0.767	1.34E-100	Adipocytes
Gfpt2	2.38E-102	-1.848	0.371	0.594	4.76E-99	Adipocytes
Gas6	7.79E-102	-0.738	0.179	0.493	1.56E-98	Adipocytes
Sfrp4	2.71E-101	-0.894	0.026	0.324	5.43E-98	Adipocytes
Ptch2	7.88E-100	0.578	0.408	0.222	1.58E-96	Adipocytes
Rab7b	1.14E-99	-0.830	0.241	0.566	2.27E-96	Adipocytes
Insyn2b	1.46E-99	-0.717	0.229	0.455	2.91E-96	Adipocytes
Galnt17	1.94E-99	-1.142	0.332	0.601	3.88E-96	Adipocytes
Pi16	4.66E-99	-2.190	0.290	0.551	9.33E-96	Adipocytes
Kcnq5	1.63E-98	-0.927	0.212	0.52	3.26E-95	Adipocytes
Gabra3	5.51E-95	-1.394	0.313	0.571	1.10E-91	Adipocytes
Rorb	8.35E-95	-1.294	0.272	0.549	1.67E-91	Adipocytes
Sema6a	2.00E-92	-0.532	0.291	0.602	4.01E-89	Adipocytes
Postn	4.48E-92	-1.180	0.235	0.451	8.96E-89	Adipocytes
Timp3	6.76E-92	-0.934	0.252	0.473	1.35E-88	Adipocytes
Myo1e	2.58E-91	-1.543	0.491	0.701	5.17E-88	Adipocytes
Gm17268	1.65E-90	0.758	0.447	0.332	3.30E-87	Adipocytes
Apcdd1	2.09E-90	0.526	0.390	0.202	4.18E-87	Adipocytes
Cdh9	5.23E-90	-0.731	0.235	0.499	1.05E-86	Adipocytes
Rnf180	2.95E-88	-0.509	0.296	0.593	5.90E-85	Adipocytes
Gabre	5.77E-87	-0.526	0.157	0.381	1.15E-83	Adipocytes
Itgbl1	6.89E-87	-1.857	0.441	0.688	1.38E-83	Adipocytes
Ano3	3.07E-84	-1.332	0.241	0.494	6.13E-81	Adipocytes
Chn2	9.61E-83	-1.153	0.295	0.574	1.92E-79	Adipocytes
Sema3d	6.20E-82	-0.882	0.171	0.379	1.24E-78	Adipocytes
Plagl1	2.77E-81	0.522	0.321	0.168	5.55E-78	Adipocytes
Aebp1	1.21E-79	-1.198	0.041	0.344	2.41E-76	Adipocytes
Mgmt	2.20E-79	0.520	0.541	0.456	4.41E-76	Adipocytes
C6	5.55E-78	0.530	0.430	0.307	1.11E-74	Adipocytes
Lcp1	5.50E-77	-1.355	0.326	0.486	1.10E-73	Adipocytes
Pla2g7	1.00E-76	-0.719	0.152	0.378	2.01E-73	Adipocytes
C5ar1	1.42E-76	-0.535	0.127	0.365	2.83E-73	Adipocytes
Ror1	4.97E-75	-1.129	0.383	0.643	9.93E-72	Adipocytes
Sema3e	8.56E-75	-1.666	0.331	0.566	1.71E-71	Adipocytes
Ctss	2.89E-73	-1.096	0.160	0.378	5.79E-70	Adipocytes
Osbpl3	4.58E-73	-0.681	0.293	0.586	9.15E-70	Adipocytes
Gria3	8.13E-72	-1.008	0.313	0.597	1.63E-68	Adipocytes
Msr1	1.16E-71	-0.611	0.222	0.42	2.32E-68	Adipocytes
Plac8	2.81E-71	-0.552	0.046	0.396	5.62E-68	Adipocytes
Basp1	4.13E-71	-0.769	0.236	0.526	8.26E-68	Adipocytes
Eln	7.88E-71	-1.657	0.320	0.532	1.58E-67	Adipocytes
Cybb	8.32E-71	-0.974	0.180	0.376	1.66E-67	Adipocytes
Myo1f	1.72E-68	-1.535	0.421	0.53	3.44E-65	Adipocytes
Thsd7a	2.64E-67	-1.432	0.515	0.718	5.28E-64	Adipocytes
St8sia4	4.12E-66	-1.161	0.271	0.428	8.24E-63	Adipocytes
Xylt1	5.56E-66	-1.077	0.481	0.641	1.11E-62	Adipocytes
Bmp6	2.24E-65	-0.820	0.235	0.483	4.48E-62	Adipocytes
Clec10a	5.43E-65	-1.230	0.306	0.468	1.09E-61	Adipocytes
Mmp3	2.27E-64	-0.783	0.141	0.316	4.53E-61	Adipocytes
Igfbp6	2.96E-64	-1.212	0.062	0.313	5.91E-61	Adipocytes
Itga4	3.51E-64	-1.254	0.274	0.437	7.03E-61	Adipocytes
Fyb	1.46E-63	-1.583	0.430	0.542	2.93E-60	Adipocytes
Skap1	9.14E-63	-0.522	0.084	0.276	1.83E-59	Adipocytes
Cd84	1.24E-62	-1.373	0.356	0.485	2.47E-59	Adipocytes
Ly6a	2.35E-62	-1.195	0.063	0.375	4.71E-59	Adipocytes

Adamtsl3	8.17E-62	-0.847	0.232	0.471	1.63E-58	Adipocytes
Ms4a7	1.04E-59	-0.533	0.248	0.433	2.09E-56	Adipocytes
Vcan	4.01E-59	-1.435	0.349	0.587	8.03E-56	Adipocytes
Lyve1	6.78E-58	-0.617	0.047	0.297	1.36E-54	Adipocytes
Spi1	4.87E-57	-0.783	0.256	0.409	9.75E-54	Adipocytes
Col4a5	1.38E-56	-0.615	0.279	0.51	2.77E-53	Adipocytes
Plek	1.89E-56	-0.636	0.169	0.474	3.78E-53	Adipocytes
Nckap1l	2.34E-56	-0.914	0.320	0.447	4.68E-53	Adipocytes
Diaph3	3.87E-56	-0.765	0.285	0.551	7.74E-53	Adipocytes
Lyz2	9.77E-56	-1.250	0.138	0.362	1.95E-52	Adipocytes
Sntg1	2.45E-55	-2.031	0.367	0.599	4.90E-52	Adipocytes
Eya4	9.34E-55	-0.799	0.364	0.607	1.87E-51	Adipocytes
Emb	1.85E-54	-0.657	0.206	0.466	3.71E-51	Adipocytes
Gpnmb	2.17E-54	-1.121	0.194	0.427	4.34E-51	Adipocytes
B4galt5	2.40E-53	-0.750	0.353	0.577	4.80E-50	Adipocytes
Prss23	1.04E-52	-0.753	0.045	0.268	2.09E-49	Adipocytes
Lgmn	2.72E-52	-1.211	0.294	0.465	5.45E-49	Adipocytes
Smpd3	3.26E-52	-1.533	0.342	0.579	6.52E-49	Adipocytes
Adgre1	3.80E-52	-1.458	0.419	0.51	7.60E-49	Adipocytes
Sgip1	5.33E-52	-0.596	0.278	0.524	1.07E-48	Adipocytes
Nav2	3.62E-50	-0.539	0.209	0.464	7.23E-47	Adipocytes
Ngf	1.61E-49	-0.528	0.159	0.332	3.21E-46	Adipocytes
Shtn1	1.98E-48	-1.223	0.403	0.484	3.96E-45	Adipocytes
Blnk	9.08E-47	-1.179	0.390	0.493	1.82E-43	Adipocytes
Epha3	1.04E-46	-1.104	0.261	0.455	2.08E-43	Adipocytes
Ccl6	3.73E-46	-0.998	0.182	0.358	7.47E-43	Adipocytes
Gria4	1.36E-45	-0.991	0.196	0.407	2.71E-42	Adipocytes
Pcdh19	1.88E-45	-1.049	0.391	0.56	3.76E-42	Adipocytes
Pde10a	6.03E-45	-0.798	0.171	0.432	1.21E-41	Adipocytes
Dclk1	7.13E-45	-0.890	0.437	0.628	1.43E-41	Adipocytes
Igfbp5	1.30E-43	-1.729	0.247	0.424	2.59E-40	Adipocytes
C1qtnf7	2.14E-43	-1.045	0.293	0.496	4.28E-40	Adipocytes
Serpinf1	3.47E-43	-1.092	0.166	0.332	6.93E-40	Adipocytes
Anpep	4.01E-43	-0.666	0.143	0.46	8.02E-40	Adipocytes
Csf2ra	3.29E-41	-0.918	0.346	0.42	6.57E-38	Adipocytes
Gas2l3	7.60E-41	-0.591	0.278	0.419	1.52E-37	Adipocytes
Il7r	9.45E-41	-0.514	0.152	0.307	1.89E-37	Adipocytes
Ednrb	9.51E-41	-0.824	0.252	0.425	1.90E-37	Adipocytes
Cd200r1	5.92E-40	-1.087	0.331	0.452	1.18E-36	Adipocytes
Pik3r5	3.04E-39	-0.786	0.269	0.42	6.09E-36	Adipocytes
Gpc3	3.99E-39	-0.853	0.267	0.448	7.99E-36	Adipocytes
Ripor2	4.39E-39	-0.664	0.238	0.424	8.77E-36	Adipocytes
Col8a1	9.64E-39	-0.764	0.219	0.386	1.93E-35	Adipocytes
Slc25a21	1.57E-38	-0.521	0.134	0.295	3.14E-35	Adipocytes
Fgf7	6.30E-38	-0.648	0.251	0.392	1.26E-34	Adipocytes
Ppp2r2b	1.14E-37	-1.157	0.253	0.459	2.28E-34	Adipocytes
Tnfrsf11a	1.51E-37	-0.851	0.348	0.469	3.02E-34	Adipocytes
Rgs6	1.91E-37	-1.212	0.333	0.548	3.83E-34	Adipocytes
Ccr2	3.35E-37	-1.170	0.091	0.287	6.70E-34	Adipocytes
Ms4a6c	1.84E-36	-0.775	0.271	0.411	3.68E-33	Adipocytes
Wdfy4	4.07E-35	-0.922	0.330	0.429	8.14E-32	Adipocytes
Ntn4	6.04E-35	-0.934	0.236	0.426	1.21E-31	Adipocytes
Zfyve28	1.22E-34	0.583	0.499	0.515	2.43E-31	Adipocytes
Map1b	1.52E-34	-0.673	0.189	0.346	3.05E-31	Adipocytes
Mpeg1	2.11E-34	-0.507	0.020	0.259	4.23E-31	Adipocytes
Car8	4.89E-34	-0.801	0.210	0.376	9.77E-31	Adipocytes
Plxnc1	1.12E-33	-0.517	0.234	0.371	2.24E-30	Adipocytes
Lilr4b	3.04E-33	-0.504	0.197	0.353	6.09E-30	Adipocytes
C7	3.11E-33	-2.115	0.288	0.442	6.22E-30	Adipocytes
Tec	7.91E-33	-0.798	0.343	0.454	1.58E-29	Adipocytes
Cd248	2.08E-32	-0.860	0.062	0.271	4.16E-29	Adipocytes
Apod	2.23E-32	-0.665	0.124	0.291	4.46E-29	Adipocytes

Gucy1a2	2.55E-32	-0.564	0.225	0.42	5.10E-29	Adipocytes
Smoc2	2.77E-32	-0.706	0.279	0.444	5.53E-29	Adipocytes
Il1r2	7.90E-32	-0.525	0.126	0.283	1.58E-28	Adipocytes
Abca8a	1.98E-31	-1.349	0.493	0.604	3.96E-28	Adipocytes
Sirpa	1.71E-30	-0.979	0.362	0.512	3.43E-27	Adipocytes
St18	2.00E-30	-0.913	0.326	0.457	4.01E-27	Adipocytes
Kcnk13	2.24E-30	-0.930	0.366	0.488	4.47E-27	Adipocytes
Aspn	2.54E-30	-1.127	0.284	0.424	5.09E-27	Adipocytes
Ubash3b	4.33E-30	-1.070	0.357	0.484	8.65E-27	Adipocytes
Fat3	5.95E-30	-0.718	0.254	0.383	1.19E-26	Adipocytes
Robo1	2.39E-29	-1.272	0.421	0.587	4.78E-26	Adipocytes
Errfi1	3.11E-29	-1.297	0.360	0.557	6.22E-26	Adipocytes
Srgap3	5.58E-29	-0.507	0.190	0.445	1.12E-25	Adipocytes
Fnip2	5.64E-29	-0.743	0.636	0.763	1.13E-25	Adipocytes
Cfh	2.11E-27	-1.602	0.425	0.572	4.23E-24	Adipocytes
Plxna4	2.99E-27	-0.801	0.355	0.498	5.98E-24	Adipocytes
Specc1	5.19E-27	-0.826	0.432	0.534	1.04E-23	Adipocytes
C3	1.81E-26	-1.201	0.129	0.317	3.62E-23	Adipocytes
Epsti1	2.04E-26	-1.261	0.421	0.492	4.08E-23	Adipocytes
Atp6v0d2	2.26E-26	-1.002	0.320	0.44	4.52E-23	Adipocytes
Sult1e1	8.41E-26	-0.823	0.185	0.315	1.68E-22	Adipocytes
Meox2	1.07E-25	-0.833	0.298	0.463	2.14E-22	Adipocytes
Ptpn22	1.62E-25	-0.876	0.271	0.382	3.24E-22	Adipocytes
Plcxd3	1.97E-25	-1.149	0.215	0.387	3.94E-22	Adipocytes
Dkk2	4.30E-25	-0.778	0.212	0.382	8.60E-22	Adipocytes
Cdk6	6.98E-25	-0.624	0.355	0.521	1.40E-21	Adipocytes
Kctd12	7.01E-25	-0.624	0.086	0.304	1.40E-21	Adipocytes
Arhgap25	5.24E-24	-0.876	0.311	0.408	1.05E-20	Adipocytes
Csmd1	1.01E-23	-1.119	0.179	0.313	2.03E-20	Adipocytes
Aoah	1.16E-22	-0.724	0.362	0.484	2.31E-19	Adipocytes
Cotl1	1.69E-22	-0.620	0.163	0.345	3.38E-19	Adipocytes
Cst3	5.55E-22	-0.787	0.037	0.358	1.11E-18	Adipocytes
C1qb	5.66E-22	-0.901	0.165	0.32	1.13E-18	Adipocytes
Nlgn1	6.25E-22	-0.673	0.159	0.296	1.25E-18	Adipocytes
Hspa5	6.64E-22	-0.731	0.195	0.431	1.33E-18	Adipocytes
Cd44	9.26E-22	-1.466	0.407	0.508	1.85E-18	Adipocytes
Cd300a	1.07E-21	-0.759	0.292	0.376	2.15E-18	Adipocytes
Ddx60	1.51E-21	-0.610	0.518	0.68	3.01E-18	Adipocytes
Fnbp1l	1.68E-21	-0.561	0.195	0.437	3.35E-18	Adipocytes
Gpm6b	5.49E-21	-0.608	0.230	0.374	1.10E-17	Adipocytes
Nipal2	5.79E-21	-0.672	0.167	0.317	1.16E-17	Adipocytes
Bgn	8.27E-21	-0.864	0.078	0.253	1.65E-17	Adipocytes
Ccr5	2.13E-20	-0.681	0.258	0.361	4.27E-17	Adipocytes
Ehd4	4.42E-20	-1.009	0.345	0.466	8.84E-17	Adipocytes
Fmo2	8.70E-20	-0.862	0.215	0.32	1.74E-16	Adipocytes
Adamts16	6.04E-19	-0.952	0.221	0.385	1.21E-15	Adipocytes
Mafb	1.34E-18	-0.817	0.228	0.361	2.67E-15	Adipocytes
Ldb2	4.34E-18	-0.713	0.333	0.489	8.67E-15	Adipocytes
Hexa	4.36E-18	-0.571	0.208	0.429	8.71E-15	Adipocytes
Cfp	9.23E-17	-0.603	0.139	0.287	1.85E-13	Adipocytes
Pcolce	3.16E-16	-0.686	0.135	0.331	6.32E-13	Adipocytes
Itgb2	3.67E-16	-0.522	0.088	0.272	7.34E-13	Adipocytes
Pola1	5.70E-16	-0.504	0.535	0.666	1.14E-12	Adipocytes
Stab1	2.07E-15	-0.879	0.416	0.487	4.15E-12	Adipocytes
Abcg3	3.74E-15	-0.600	0.211	0.334	7.47E-12	Adipocytes
Csgalnact1	7.78E-15	-0.631	0.430	0.577	1.56E-11	Adipocytes
Ciita	1.21E-14	-0.648	0.177	0.319	2.42E-11	Adipocytes
Bank1	2.90E-14	-0.776	0.303	0.399	5.80E-11	Adipocytes
Col12a1	6.78E-14	-0.803	0.317	0.393	1.36E-10	Adipocytes
Cd180	1.50E-13	-0.661	0.224	0.337	3.01E-10	Adipocytes
Cd300lb	2.94E-13	-0.572	0.290	0.367	5.88E-10	Adipocytes
Grn	2.99E-13	-0.793	0.196	0.396	5.98E-10	Adipocytes

Aldh1a3	5.50E-13	-0.665	0.226	0.337	1.10E-09	Adipocytes
Ftl1	1.39E-12	-0.651	0.146	0.333	2.78E-09	Adipocytes
Selenop	1.83E-12	-1.127	0.399	0.497	3.65E-09	Adipocytes
Cacna1d	2.81E-12	-0.803	0.417	0.486	5.62E-09	Adipocytes
Abhd12	2.99E-12	-0.520	0.748	0.537	5.98E-09	Adipocytes
Prkcb	9.45E-12	-0.892	0.419	0.473	1.89E-08	Adipocytes
Foxp2	9.59E-12	-0.658	0.198	0.303	1.92E-08	Adipocytes
Ms4a6b	1.33E-11	-0.517	0.305	0.395	2.65E-08	Adipocytes
Ccbe1	5.64E-10	-0.662	0.228	0.336	1.13E-06	Adipocytes
P3h2	1.11E-09	-0.643	0.348	0.449	2.22E-06	Adipocytes
Hmcn2	1.73E-09	-0.979	0.300	0.423	3.46E-06	Adipocytes
Ctsb	1.07E-08	-1.191	0.443	0.462	2.14E-05	Adipocytes
Ctsl	1.42E-08	-0.662	0.107	0.348	2.84E-05	Adipocytes
Pde11a	3.69E-08	-0.755	0.305	0.434	7.39E-05	Adipocytes
C1qa	4.37E-08	-0.985	0.210	0.308	8.73E-05	Adipocytes
Mgl2	8.05E-08	-0.660	0.118	0.251	0.000160919	Adipocytes
Apoe	1.66E-07	-1.848	0.518	0.506	0.000332292	Adipocytes
Psap	1.85E-07	-1.587	0.545	0.52	0.000370039	Adipocytes
Tmem106a	1.94E-07	-0.517	0.274	0.422	0.000387528	Adipocytes
Cadm1	1.97E-07	-0.945	0.352	0.41	0.000393837	Adipocytes
Entpd1	2.03E-07	-0.885	0.407	0.504	0.000406611	Adipocytes
Atp8b4	6.26E-07	-0.974	0.349	0.39	0.001251561	Adipocytes
Colec12	7.52E-07	-0.603	0.533	0.67	0.001503999	Adipocytes
Soat1	7.57E-07	-0.617	0.117	0.371	0.001514223	Adipocytes
Retnla	1.71E-06	-1.363	0.201	0.302	0.003422922	Adipocytes
Mamdc2	1.80E-06	-0.857	0.316	0.412	0.00360298	Adipocytes
Col6a1	1.98E-06	-0.537	0.278	0.298	0.003951408	Adipocytes
Cdh13	2.18E-06	-0.790	0.386	0.512	0.004361848	Adipocytes
Adamts12	3.07E-06	-0.632	0.799	0.726	0.006149339	Adipocytes
Abtb2	9.76E-06	-0.511	0.255	0.416	0.01951619	Adipocytes
Atp1a1	3.38E-05	-0.535	0.077	0.328	0.067672227	Adipocytes
Thsd4	4.57E-05	-0.641	0.085	0.311	0.091326282	Adipocytes
Pcolce2	0.0002026	-0.774	0.471	0.479	0.405136152	Adipocytes
P2rx7	0.0003321	-0.582	0.286	0.437	0.664288795	Adipocytes
Rasa1	0.0009316	-0.704	0.795	0.699	1	Adipocytes
Tox	0.0013526	-0.523	0.233	0.344	1	Adipocytes
Gsn	0.0013537	-1.687	0.833	0.684	1	Adipocytes
Evl	0.0014528	-0.864	0.344	0.389	1	Adipocytes
Trim25	0.0019791	-0.626	0.408	0.496	1	Adipocytes
Lgals3	0.0021768	-1.041	0.349	0.31	1	Adipocytes
Abca8b	0.0023905	-0.541	0.473	0.45	1	Adipocytes
Cd9	0.0024486	-0.603	0.117	0.338	1	Adipocytes
Adgre4	0.0031076	-0.631	0.230	0.301	1	Adipocytes
Tmtc2	0.0037264	-0.641	0.469	0.583	1	Adipocytes
Cd226	0.0040168	-0.913	0.165	0.264	1	Adipocytes
Cd163	0.0062566	-1.153	0.395	0.447	1	Adipocytes
Sdc2	0.0069273	-0.718	0.539	0.588	1	Adipocytes
Nrp2	0.008412	-0.715	0.407	0.439	1	Adipocytes
Dact2	0.0085063	-0.531	0.148	0.268	1	Adipocytes
Igfbp7	0.0092416	-0.508	0.399	0.365	1	Adipocytes
Pi16.1	0	3.228	0.936	0.358	0	Preadipocytes_1
Sema3e.1	0	3.124	0.886	0.402	0	Preadipocytes_1
Sntg1.1	0	2.846	0.801	0.482	0	Preadipocytes_1
Igfbp5.1	0	2.821	0.851	0.23	0	Preadipocytes_1
Gfpt2.1	0	2.791	0.933	0.426	0	Preadipocytes_1
Rgs6.1	0	2.431	0.741	0.437	0	Preadipocytes_1
Ano3.1	0	2.416	0.832	0.322	0	Preadipocytes_1
Col14a1.1	0	2.394	0.941	0.566	0	Preadipocytes_1
Rorb.1	0	2.389	0.779	0.413	0	Preadipocytes_1
Pcdh9.1	0	2.367	0.811	0.514	0	Preadipocytes_1
Adamtsl1.1	0	2.298	0.969	0.651	0	Preadipocytes_1
Mfap5.1	0	2.241	0.898	0.382	0	Preadipocytes_1

Nox4.1	0	2.195	0.956	0.6	0	Preadipocytes_1
Robo1.1	0	2.108	0.749	0.496	0	Preadipocytes_1
Adamts16.1	0	2.088	0.620	0.267	0	Preadipocytes_1
Smpd3.1	0	2.074	0.735	0.478	0	Preadipocytes_1
Lvrn.1	0	1.984	0.793	0.452	0	Preadipocytes_1
Vcan.1	0	1.899	0.815	0.458	0	Preadipocytes_1
Adamts5.1	0	1.857	0.964	0.577	0	Preadipocytes_1
Bmp6.1	0	1.833	0.690	0.36	0	Preadipocytes_1
Nr1h4.1	0	1.823	0.719	0.383	0	Preadipocytes_1
Pcolce2.1	0	1.798	0.803	0.355	0	Preadipocytes_1
Car8.1	0	1.761	0.655	0.24	0	Preadipocytes_1
Ugdh	0	1.749	0.732	0.359	0	Preadipocytes_1
Ntn4.1	0	1.718	0.677	0.297	0	Preadipocytes_1
Vgll3	0	1.713	0.876	0.492	0	Preadipocytes_1
Prkg1.1	0	1.711	0.894	0.654	0	Preadipocytes_1
Eya4.1	0	1.700	0.753	0.509	0	Preadipocytes_1
Errfi1.1	0	1.624	0.778	0.438	0	Preadipocytes_1
Nlgn1.1	0	1.587	0.502	0.194	0	Preadipocytes_1
Col12a1.1	0	1.571	0.683	0.269	0	Preadipocytes_1
Aldh1a3.1	0	1.567	0.615	0.212	0	Preadipocytes_1
Thsd7a.1	0	1.525	0.925	0.604	0	Preadipocytes_1
Clec3b.1	0	1.523	0.875	0.361	0	Preadipocytes_1
C3.1	0	1.509	0.628	0.166	0	Preadipocytes_1
Dcn.1	0	1.478	0.961	0.49	0	Preadipocytes_1
Sema3d.1	0	1.442	0.678	0.229	0	Preadipocytes_1
Aspn.1	0	1.435	0.711	0.29	0	Preadipocytes_1
Igfbp6.1	0	1.427	0.664	0.135	0	Preadipocytes_1
Cdh9.1	0	1.412	0.701	0.376	0	Preadipocytes_1
Pcdh19.1	0	1.357	0.754	0.456	0	Preadipocytes_1
Ly6a.1	0	1.336	0.674	0.207	0	Preadipocytes_1
C1qtnf7.1	0	1.328	0.659	0.399	0	Preadipocytes_1
Sdc2.1	0	1.326	0.725	0.528	0	Preadipocytes_1
Cd248.1	0	1.277	0.592	0.112	0	Preadipocytes_1
Aebp1.1	0	1.276	0.656	0.172	0	Preadipocytes_1
Efna5.1	0	1.264	0.836	0.57	0	Preadipocytes_1
Gabra3.1	0	1.236	0.760	0.453	0	Preadipocytes_1
Prss23.1	0	1.235	0.572	0.113	0	Preadipocytes_1
Pde10a.1	0	1.214	0.639	0.307	0	Preadipocytes_1
Pcsk5	0	1.179	0.784	0.566	0	Preadipocytes_1
Postn.1	0	1.149	0.733	0.306	0	Preadipocytes_1
Fbln2.1	0	1.086	0.711	0.356	0	Preadipocytes_1
Abca8a.1	0	1.044	0.814	0.504	0	Preadipocytes_1
Il1r2.1	0	1.033	0.555	0.152	0	Preadipocytes_1
Dclk1.1	0	1.011	0.780	0.536	0	Preadipocytes_1
Timp3.1	0	0.971	0.684	0.354	0	Preadipocytes_1
Anxa1	0	0.897	0.800	0.505	0	Preadipocytes_1
Col1a2.1	0	0.857	0.936	0.512	0	Preadipocytes_1
Mid1.1	0	0.797	0.946	0.607	0	Preadipocytes_1
Lama4.1	0	0.788	0.920	0.546	0	Preadipocytes_1
Tead1.1	0	0.783	0.861	0.597	0	Preadipocytes_1
Itih5.1	0	0.772	0.774	0.453	0	Preadipocytes_1
Col3a1.1	0	0.764	0.977	0.644	0	Preadipocytes_1
Gabre.1	0	0.762	0.582	0.265	0	Preadipocytes_1
Sparc	0	0.751	0.801	0.478	0	Preadipocytes_1
Sult1e1.1	0	0.684	0.564	0.198	0	Preadipocytes_1
Dst.1	0	0.622	0.938	0.551	0	Preadipocytes_1
Spock3	0	0.621	0.474	0.082	0	Preadipocytes_1
Sfrp4.1	0	0.620	0.588	0.171	0	Preadipocytes_1
Tbc1d8	0	-0.501	0.073	0.416	0	Preadipocytes_1
Dpf3.1	0	-0.514	0.120	0.482	0	Preadipocytes_1
Scd1.1	0	-0.525	0.032	0.311	0	Preadipocytes_1
Lair1	0	-0.531	0.001	0.462	0	Preadipocytes_1

Efr3b	0	-0.550	0.030	0.452	0	Preadipocytes_1
Myo10	0	-0.598	0.109	0.52	0	Preadipocytes_1
Rasgrp3	0	-0.602	0.013	0.484	0	Preadipocytes_1
Hck	0	-0.606	0.001	0.443	0	Preadipocytes_1
Naa50	0	-0.612	0.108	0.494	0	Preadipocytes_1
Hgf	0	-0.626	0.138	0.63	0	Preadipocytes_1
Slc22a23.1	0	-0.629	0.017	0.513	0	Preadipocytes_1
Mical2	0	-0.631	0.170	0.558	0	Preadipocytes_1
Smc4	0	-0.633	0.136	0.534	0	Preadipocytes_1
P2ry12	0	-0.654	0.027	0.515	0	Preadipocytes_1
Gm5150	0	-0.677	0.001	0.425	0	Preadipocytes_1
Klf8	0	-0.687	0.015	0.516	0	Preadipocytes_1
Ms4a4a	0	-0.689	0.040	0.523	0	Preadipocytes_1
Ptchd1	0	-0.707	0.008	0.454	0	Preadipocytes_1
Peg3.1	0	-0.711	0.122	0.307	0	Preadipocytes_1
Gcnt1	0	-0.735	0.001	0.484	0	Preadipocytes_1
Nlrc5.1	0	-0.736	0.061	0.574	0	Preadipocytes_1
Rel	0	-0.738	0.033	0.428	0	Preadipocytes_1
Zranb3	0	-0.740	0.089	0.417	0	Preadipocytes_1
Il7r.1	0	-0.756	0.001	0.396	0	Preadipocytes_1
Lilr4b.1	0	-0.758	0.001	0.459	0	Preadipocytes_1
C5ar1.1	0	-0.771	0.000	0.462	0	Preadipocytes_1
Slc5a3.1	0	-0.773	0.022	0.486	0	Preadipocytes_1
Card11	0	-0.781	0.009	0.463	0	Preadipocytes_1
Nrg2	0	-0.795	0.086	0.55	0	Preadipocytes_1
Cgnl1.1	0	-0.806	0.090	0.531	0	Preadipocytes_1
Rin2.1	0	-0.817	0.516	0.816	0	Preadipocytes_1
Tcirg1	0	-0.828	0.081	0.459	0	Preadipocytes_1
Soat1.1	0	-0.829	0.080	0.437	0	Preadipocytes_1
Prune2	0	-0.831	0.054	0.602	0	Preadipocytes_1
Mctp2	0	-0.841	0.020	0.513	0	Preadipocytes_1
Fnbp1l.1	0	-0.851	0.060	0.537	0	Preadipocytes_1
Ms4a7.1	0	-0.859	0.002	0.565	0	Preadipocytes_1
Zbtb7c.1	0	-0.882	0.162	0.622	0	Preadipocytes_1
Slc7a8	0	-0.889	0.040	0.552	0	Preadipocytes_1
Grk3	0	-0.890	0.037	0.481	0	Preadipocytes_1
Ms4a6b.1	0	-0.893	0.007	0.527	0	Preadipocytes_1
Sort1.1	0	-0.894	0.001	0.501	0	Preadipocytes_1
Sh3pxd2a.1	0	-0.908	0.187	0.676	0	Preadipocytes_1
Plxnc1.1	0	-0.908	0.007	0.486	0	Preadipocytes_1
Hexa.1	0	-0.909	0.143	0.499	0	Preadipocytes_1
P2rx7.1	0	-0.922	0.071	0.549	0	Preadipocytes_1
Cd300lb.1	0	-0.924	0.004	0.491	0	Preadipocytes_1
Msr1.1	0	-0.925	0.002	0.544	0	Preadipocytes_1
Pag1.1	0	-0.937	0.068	0.581	0	Preadipocytes_1
Calm2	0	-0.942	0.174	0.557	0	Preadipocytes_1
Nos1ap.1	0	-0.946	0.139	0.628	0	Preadipocytes_1
Pla2g7.1	0	-0.954	0.000	0.482	0	Preadipocytes_1
Chd7.1	0	-0.958	0.028	0.53	0	Preadipocytes_1
Tmem106a.1	0	-0.960	0.065	0.531	0	Preadipocytes_1
Ripor2.1	0	-0.973	0.066	0.527	0	Preadipocytes_1
Mpp1	0	-0.989	0.066	0.571	0	Preadipocytes_1
Ccr5.1	0	-0.990	0.009	0.477	0	Preadipocytes_1
Gas2l3.1	0	-0.992	0.002	0.553	0	Preadipocytes_1
Grn.1	0	-0.993	0.102	0.473	0	Preadipocytes_1
Pacrg.1	0	-1.006	0.147	0.536	0	Preadipocytes_1
Lpin1.1	0	-1.037	0.053	0.511	0	Preadipocytes_1
Hpgds	0	-1.061	0.002	0.612	0	Preadipocytes_1
Trim25.1	0	-1.071	0.127	0.621	0	Preadipocytes_1
Nceh1	0	-1.078	0.080	0.478	0	Preadipocytes_1
Atp6v1a	0	-1.092	0.130	0.636	0	Preadipocytes_1
Insyn2b.1	0	-1.112	0.050	0.569	0	Preadipocytes_1

Tank.1	0	-1.113	0.300	0.774	0	Preadipocytes_1
Rnf149	0	-1.115	0.045	0.566	0	Preadipocytes_1
Palld.1	0	-1.122	0.286	0.669	0	Preadipocytes_1
Gramd1b	0	-1.122	0.044	0.596	0	Preadipocytes_1
Cdk6.1	0	-1.125	0.252	0.594	0	Preadipocytes_1
Fggy.1	0	-1.139	0.100	0.669	0	Preadipocytes_1
Cd300a.1	0	-1.152	0.002	0.503	0	Preadipocytes_1
Spi1.1	0	-1.153	0.001	0.536	0	Preadipocytes_1
Ank	0	-1.164	0.138	0.643	0	Preadipocytes_1
Mafb.1	0	-1.173	0.003	0.474	0	Preadipocytes_1
Fat3.1	0	-1.174	0.010	0.502	0	Preadipocytes_1
Ms4a6c.1	0	-1.181	0.004	0.54	0	Preadipocytes_1
Noct.1	0	-1.182	0.095	0.577	0	Preadipocytes_1
Itm2b	0	-1.184	0.296	0.66	0	Preadipocytes_1
Atp6v1b2	0	-1.187	0.234	0.674	0	Preadipocytes_1
Tspan12.1	0	-1.191	0.088	0.292	0	Preadipocytes_1
Irak2.1	0	-1.224	0.083	0.563	0	Preadipocytes_1
Cybb.1	0	-1.238	0.001	0.485	0	Preadipocytes_1
Ednrb.1	0	-1.246	0.063	0.532	0	Preadipocytes_1
Pik3r5.1	0	-1.252	0.053	0.533	0	Preadipocytes_1
P3h2.1	0	-1.262	0.064	0.577	0	Preadipocytes_1
Ptpn22.1	0	-1.288	0.005	0.505	0	Preadipocytes_1
Aoah.1	0	-1.298	0.067	0.621	0	Preadipocytes_1
Tec.1	0	-1.302	0.021	0.598	0	Preadipocytes_1
Entpd1.1	0	-1.311	0.132	0.628	0	Preadipocytes_1
Enpp2.1	0	-1.322	0.204	0.393	0	Preadipocytes_1
Bank1.1	0	-1.322	0.004	0.532	0	Preadipocytes_1
Plxna4.1	0	-1.366	0.073	0.634	0	Preadipocytes_1
Ccl6.1	0	-1.374	0.002	0.462	0	Preadipocytes_1
Aplp2.1	0	-1.378	0.187	0.632	0	Preadipocytes_1
Nrp2.1	0	-1.386	0.018	0.594	0	Preadipocytes_1
Mamdc2.1	0	-1.398	0.077	0.523	0	Preadipocytes_1
Evl.1	0	-1.405	0.009	0.525	0	Preadipocytes_1
Wdfy4.1	0	-1.414	0.036	0.561	0	Preadipocytes_1
Tnfrsf11a.1	0	-1.416	0.045	0.609	0	Preadipocytes_1
Ctss.1	0	-1.435	0.000	0.483	0	Preadipocytes_1
Nckap1l.1	0	-1.440	0.005	0.593	0	Preadipocytes_1
Arhgap25.1	0	-1.472	0.004	0.545	0	Preadipocytes_1
Slc27a1.1	0	-1.486	0.001	0.603	0	Preadipocytes_1
Sipa1l1.1	0	-1.488	0.161	0.729	0	Preadipocytes_1
Prkch	0	-1.493	0.009	0.613	0	Preadipocytes_1
Plekhg5	0	-1.505	0.052	0.636	0	Preadipocytes_1
Tns3	0	-1.510	0.047	0.66	0	Preadipocytes_1
Kcnk13.1	0	-1.511	0.069	0.626	0	Preadipocytes_1
Pfkfb3.1	0	-1.513	0.013	0.587	0	Preadipocytes_1
Ehd4.1	0	-1.531	0.035	0.608	0	Preadipocytes_1
Pik3ap1	0	-1.531	0.004	0.671	0	Preadipocytes_1
Arid5b.1	0	-1.543	0.370	0.822	0	Preadipocytes_1
Cacna1d.1	0	-1.548	0.044	0.642	0	Preadipocytes_1
Atp8b4.1	0	-1.567	0.004	0.53	0	Preadipocytes_1
Csf2ra.1	0	-1.568	0.002	0.566	0	Preadipocytes_1
St18.1	0	-1.584	0.012	0.603	0	Preadipocytes_1
Rasa1.1	0	-1.610	0.369	0.841	0	Preadipocytes_1
Stab1.1	0	-1.613	0.010	0.656	0	Preadipocytes_1
Fgf13.1	0	-1.613	0.078	0.557	0	Preadipocytes_1
St8sia4.1	0	-1.624	0.036	0.549	0	Preadipocytes_1
Cd200r1.1	0	-1.626	0.027	0.592	0	Preadipocytes_1
Prkcb.1	0	-1.648	0.012	0.639	0	Preadipocytes_1
Slc6a6	0	-1.667	0.174	0.701	0	Preadipocytes_1
Hipk2.1	0	-1.669	0.237	0.765	0	Preadipocytes_1
Lyz2.1	0	-1.670	0.002	0.46	0	Preadipocytes_1
Atp6v0d2.1	0	-1.677	0.014	0.581	0	Preadipocytes_1

Osbpl8	0	-1.704	0.442	0.86	0	Preadipocytes_1
Lgmn.1	0	-1.705	0.014	0.606	0	Preadipocytes_1
Selenop.1	0	-1.713	0.132	0.618	0	Preadipocytes_1
Cadm1.1	0	-1.730	0.016	0.55	0	Preadipocytes_1
Specc1.1	0	-1.734	0.085	0.687	0	Preadipocytes_1
Ubash3b.1	0	-1.758	0.089	0.611	0	Preadipocytes_1
Cpeb4.1	0	-1.777	0.181	0.745	0	Preadipocytes_1
Clec10a.1	0	-1.782	0.004	0.616	0	Preadipocytes_1
Itga4.1	0	-1.792	0.042	0.559	0	Preadipocytes_1
Tsc22d3.1	0	-1.798	0.167	0.659	0	Preadipocytes_1
Cd44.1	0	-1.799	0.204	0.606	0	Preadipocytes_1
Blnk.1	0	-1.844	0.034	0.649	0	Preadipocytes_1
Adap2	0	-1.895	0.066	0.762	0	Preadipocytes_1
Shtn1.1	0	-1.901	0.005	0.651	0	Preadipocytes_1
Mertk	0	-1.905	0.126	0.745	0	Preadipocytes_1
Igf1.1	0	-1.908	0.107	0.795	0	Preadipocytes_1
Slc16a10.1	0	-1.958	0.144	0.762	0	Preadipocytes_1
Cd163.1	0	-1.973	0.074	0.579	0	Preadipocytes_1
Plin2	0	-2.025	0.015	0.672	0	Preadipocytes_1
Abhd12.1	0	-2.028	0.104	0.739	0	Preadipocytes_1
Pltp	0	-2.032	0.007	0.608	0	Preadipocytes_1
Atp6v0a1.1	0	-2.036	0.232	0.816	0	Preadipocytes_1
Ctsb.1	0	-2.060	0.096	0.598	0	Preadipocytes_1
Lcp1.1	0	-2.065	0.002	0.641	0	Preadipocytes_1
Epsti1.1	0	-2.076	0.014	0.661	0	Preadipocytes_1
Cd84.1	0	-2.087	0.008	0.644	0	Preadipocytes_1
Pitpnc1.1	0	-2.106	0.408	0.842	0	Preadipocytes_1
Cfh.1	0	-2.119	0.174	0.697	0	Preadipocytes_1
Ahcyl2.1	0	-2.150	0.213	0.791	0	Preadipocytes_1
Ctsc	0	-2.186	0.053	0.652	0	Preadipocytes_1
Adgre1.1	0	-2.249	0.038	0.673	0	Preadipocytes_1
Bcar3.1	0	-2.288	0.011	0.58	0	Preadipocytes_1
Rbm47.1	0	-2.462	0.061	0.79	0	Preadipocytes_1
Myo1f.1	0	-2.491	0.005	0.71	0	Preadipocytes_1
Fyb.1	0	-2.494	0.060	0.705	0	Preadipocytes_1
Pde4d.1	0	-2.524	0.177	0.819	0	Preadipocytes_1
Apobec1	0	-2.548	0.069	0.718	0	Preadipocytes_1
Plcb1.1	0	-2.579	0.310	0.684	0	Preadipocytes_1
Psap.1	0	-2.708	0.164	0.66	0	Preadipocytes_1
Lpl.1	0	-2.720	0.277	0.687	0	Preadipocytes_1
Mtss1	0	-2.817	0.085	0.812	0	Preadipocytes_1
Fry.1	0	-2.837	0.131	0.64	0	Preadipocytes_1
Pparg.1	0	-2.843	0.318	0.696	0	Preadipocytes_1
Hdac9	0	-2.873	0.065	0.726	0	Preadipocytes_1
Pde3b.1	0	-2.955	0.092	0.759	0	Preadipocytes_1
Apoe.1	0	-3.214	0.064	0.676	0	Preadipocytes_1
Alcam	0	-3.566	0.046	0.71	0	Preadipocytes_1
Cd36.1	0	-3.750	0.056	0.786	0	Preadipocytes_1
F13a1	0	-3.782	0.117	0.731	0	Preadipocytes_1
Sbsn	5.47E-307	1.044	0.471	0.069	1.09E-303	Preadipocytes_1
Eln.1	6.39E-306	0.795	0.702	0.43	1.28E-302	Preadipocytes_1
Col8a1.1	1.09E-305	0.789	0.566	0.289	2.18E-302	Preadipocytes_1
Nabp1.1	1.70E-299	-0.885	0.003	0.48	3.39E-296	Preadipocytes_1
Sirpa.1	2.57E-299	-1.125	0.274	0.576	5.14E-296	Preadipocytes_1
Cd180.1	1.21E-295	-1.018	0.002	0.444	2.42E-292	Preadipocytes_1
Aldh1a1.1	7.81E-295	0.522	0.893	0.668	1.56E-291	Preadipocytes_1
Cobll1.1	9.17E-295	-1.231	0.167	0.539	1.83E-291	Preadipocytes_1
Slc37a2	2.42E-292	-0.650	0.001	0.432	4.84E-289	Preadipocytes_1
Nav2.1	3.97E-292	-0.813	0.213	0.515	7.94E-289	Preadipocytes_1
Grm7	2.72E-291	0.617	0.436	0.136	5.45E-288	Preadipocytes_1
Gas6.1	5.63E-290	-1.032	0.202	0.548	1.13E-286	Preadipocytes_1
Gnai1.1	4.00E-289	-1.019	0.008	0.264	8.00E-286	Preadipocytes_1

Foxp2.1	1.04E-288	0.750	0.529	0.199	2.08E-285	Preadipocytes_1
Ppp2r2b.1	4.52E-287	1.884	0.618	0.362	9.04E-284	Preadipocytes_1
Cotl1.1	8.48E-287	-0.818	0.053	0.424	1.70E-283	Preadipocytes_1
Aff2	1.37E-285	0.669	0.657	0.35	2.74E-282	Preadipocytes_1
C3ar1	3.21E-285	-0.651	0.000	0.411	6.42E-282	Preadipocytes_1
Ngf.1	7.78E-284	0.957	0.544	0.22	1.56E-280	Preadipocytes_1
Lgals3.1	2.72E-280	-1.834	0.002	0.434	5.44E-277	Preadipocytes_1
Adipor2.1	8.98E-279	-1.790	0.093	0.491	1.80E-275	Preadipocytes_1
Sorbs1.1	1.88E-278	-3.102	0.154	0.355	3.76E-275	Preadipocytes_1
Tenm4.1	3.94E-278	-3.514	0.076	0.543	7.88E-275	Preadipocytes_1
Galnt17.1	2.29E-276	0.727	0.722	0.508	4.59E-273	Preadipocytes_1
mt-Nd6.1	8.45E-276	-0.913	0.499	0.768	1.69E-272	Preadipocytes_1
Tshr.1	1.32E-275	-1.990	0.001	0.275	2.65E-272	Preadipocytes_1
Ptpre	1.17E-272	-1.125	0.005	0.512	2.35E-269	Preadipocytes_1
Cd86	4.86E-272	-0.739	0.019	0.458	9.72E-269	Preadipocytes_1
Ptprg.1	1.72E-269	0.574	0.889	0.604	3.43E-266	Preadipocytes_1
Sox6.1	4.40E-268	-0.900	0.001	0.295	8.79E-265	Preadipocytes_1
St6galnac3	2.97E-266	-0.661	0.039	0.392	5.93E-263	Preadipocytes_1
Gbe1.1	2.15E-264	-0.642	0.337	0.658	4.29E-261	Preadipocytes_1
Col4a5.1	7.81E-260	0.889	0.634	0.423	1.56E-256	Preadipocytes_1
Larp1b.1	1.79E-257	-0.734	0.200	0.519	3.59E-254	Preadipocytes_1
B4galt5.1	9.94E-257	-0.732	0.341	0.628	1.99E-253	Preadipocytes_1
Nedd4l.1	9.66E-256	-1.228	0.498	0.765	1.93E-252	Preadipocytes_1
Dock5	2.03E-254	-0.611	0.172	0.459	4.05E-251	Preadipocytes_1
Itga6.1	3.11E-254	-0.504	0.000	0.37	6.21E-251	Preadipocytes_1
Slc25a13	4.71E-252	-0.637	0.134	0.415	9.43E-249	Preadipocytes_1
Abca8b.1	8.48E-250	0.957	0.640	0.382	1.70E-246	Preadipocytes_1
Nrg4.1	9.59E-249	-1.420	0.001	0.272	1.92E-245	Preadipocytes_1
Dtna	5.18E-248	1.127	0.533	0.285	1.04E-244	Preadipocytes_1
Tmem165	8.72E-247	-0.510	0.319	0.651	1.74E-243	Preadipocytes_1
Atp1a1.1	1.50E-245	-0.611	0.090	0.374	2.99E-242	Preadipocytes_1
Lyve1.1	4.61E-245	-0.796	0.001	0.365	9.22E-242	Preadipocytes_1
Pdzrn3.1	1.86E-244	-1.213	0.306	0.497	3.73E-241	Preadipocytes_1
Myl1.1	1.00E-243	-1.116	0.001	0.275	2.01E-240	Preadipocytes_1
Lipa	1.08E-243	-1.126	0.037	0.409	2.17E-240	Preadipocytes_1
Pdzd2.1	7.28E-243	0.893	0.720	0.459	1.46E-239	Preadipocytes_1
Pik3cb.1	4.44E-242	-0.928	0.002	0.49	8.88E-239	Preadipocytes_1
Lhfpl2.1	4.34E-240	-0.843	0.277	0.53	8.68E-237	Preadipocytes_1
Oxct1.1	5.18E-235	-0.688	0.275	0.606	1.04E-231	Preadipocytes_1
Cachd1.1	1.25E-227	0.648	0.745	0.496	2.50E-224	Preadipocytes_1
Adgre4.1	1.59E-225	-0.908	0.001	0.402	3.19E-222	Preadipocytes_1
Celf4	4.93E-225	-0.707	0.018	0.451	9.86E-222	Preadipocytes_1
Akr1a1	5.11E-223	-0.648	0.166	0.466	1.02E-219	Preadipocytes_1
Calcrl.1	6.00E-223	1.302	0.725	0.619	1.20E-219	Preadipocytes_1
Ctsd	4.41E-222	-1.263	0.044	0.404	8.83E-219	Preadipocytes_1
Abcg3.1	2.17E-221	-0.896	0.001	0.439	4.34E-218	Preadipocytes_1
Cd9.1	6.28E-219	-0.510	0.138	0.375	1.26E-215	Preadipocytes_1
Grb14.1	6.74E-219	-1.128	0.120	0.386	1.35E-215	Preadipocytes_1
Dlg2.1	1.75E-218	0.760	0.642	0.392	3.50E-215	Preadipocytes_1
Tmem45b.1	2.82E-218	-1.850	0.002	0.285	5.64E-215	Preadipocytes_1
Itpr1.1	6.13E-216	-0.919	0.425	0.679	1.23E-212	Preadipocytes_1
Clec4a2	9.30E-216	-0.615	0.000	0.377	1.86E-212	Preadipocytes_1
Adra1a.1	1.16E-215	-0.533	0.108	0.37	2.32E-212	Preadipocytes_1
Eepd1.1	1.31E-215	-1.667	0.037	0.499	2.62E-212	Preadipocytes_1
Npr3.1	1.85E-214	-2.641	0.018	0.269	3.71E-211	Preadipocytes_1
Skap1.1	2.11E-211	-0.664	0.029	0.336	4.22E-208	Preadipocytes_1
Pde1a.1	1.60E-209	0.597	0.674	0.423	3.21E-206	Preadipocytes_1
Ctsz	4.42E-207	-0.607	0.091	0.375	8.85E-204	Preadipocytes_1
Rgs1	2.26E-205	-0.575	0.001	0.327	4.51E-202	Preadipocytes_1
Cnnm2.1	1.02E-204	-0.737	0.437	0.662	2.05E-201	Preadipocytes_1
Ncf1	1.11E-204	-0.742	0.001	0.405	2.23E-201	Preadipocytes_1
Dact2.1	1.05E-202	1.328	0.481	0.166	2.10E-199	Preadipocytes_1

Arhgap29.1	5.39E-201	0.566	0.746	0.48	1.08E-197	Preadipocytes_1
Dmd.1	1.97E-199	0.502	0.711	0.483	3.94E-196	Preadipocytes_1
Pde7b.1	1.44E-196	-0.899	0.680	0.828	2.88E-193	Preadipocytes_1
Cav1.1	3.97E-196	-1.666	0.022	0.278	7.95E-193	Preadipocytes_1
Col4a1.1	3.77E-195	-1.275	0.399	0.571	7.54E-192	Preadipocytes_1
Retnla.1	4.38E-194	-1.927	0.002	0.398	8.75E-191	Preadipocytes_1
Adamtsl3.1	1.05E-192	1.099	0.586	0.385	2.09E-189	Preadipocytes_1
C1qa.1	2.02E-192	-1.346	0.002	0.406	4.03E-189	Preadipocytes_1
Enah	5.96E-192	0.781	0.510	0.239	1.19E-188	Preadipocytes_1
Clec12a	8.65E-192	-0.696	0.001	0.383	1.73E-188	Preadipocytes_1
Nipal2.1	1.14E-191	1.181	0.520	0.214	2.29E-188	Preadipocytes_1
Pou2f2	2.76E-191	-0.669	0.031	0.411	5.51E-188	Preadipocytes_1
Stap1	1.27E-190	-0.670	0.000	0.383	2.53E-187	Preadipocytes_1
Fgf10.1	3.44E-190	-1.014	0.275	0.399	6.89E-187	Preadipocytes_1
Map1b.1	3.20E-189	0.614	0.541	0.244	6.40E-186	Preadipocytes_1
Mmp12	3.99E-189	-0.903	0.000	0.293	7.98E-186	Preadipocytes_1
Cidec.1	3.60E-188	-2.058	0.002	0.251	7.21E-185	Preadipocytes_1
Mycbp2.1	3.05E-186	-1.369	0.754	0.89	6.10E-183	Preadipocytes_1
Anxa2.1	7.16E-185	-1.036	0.354	0.599	1.43E-181	Preadipocytes_1
C1qb.1	2.32E-184	-1.190	0.036	0.401	4.65E-181	Preadipocytes_1
Por.1	5.99E-184	-0.983	0.158	0.482	1.20E-180	Preadipocytes_1
Slc11a1	4.11E-183	-0.628	0.001	0.379	8.21E-180	Preadipocytes_1
Tnip3	8.91E-183	-0.819	0.001	0.349	1.78E-179	Preadipocytes_1
Smoc1.1	9.45E-183	-1.323	0.001	0.259	1.89E-179	Preadipocytes_1
Tmem135.1	1.68E-180	-1.476	0.407	0.677	3.36E-177	Preadipocytes_1
Kctd12.1	2.75E-179	-0.781	0.004	0.38	5.51E-176	Preadipocytes_1
Gabra2	2.30E-178	0.847	0.398	0.086	4.61E-175	Preadipocytes_1
Ipcef1	8.13E-178	-0.568	0.001	0.347	1.63E-174	Preadipocytes_1
Coro2a	2.12E-175	-0.735	0.016	0.423	4.23E-172	Preadipocytes_1
Sik2.1	3.75E-175	-1.559	0.423	0.704	7.50E-172	Preadipocytes_1
Prr16.1	4.20E-175	-1.559	0.309	0.426	8.39E-172	Preadipocytes_1
Neto1	1.73E-174	0.612	0.414	0.133	3.46E-171	Preadipocytes_1
Lamp1	3.34E-173	-0.501	0.174	0.436	6.67E-170	Preadipocytes_1
Nxn	3.42E-172	-0.505	0.297	0.514	6.83E-169	Preadipocytes_1
Fam78b	2.58E-168	-0.760	0.382	0.587	5.16E-165	Preadipocytes_1
Ftl1.1	3.19E-168	-0.855	0.092	0.392	6.37E-165	Preadipocytes_1
Irf8	1.04E-167	-0.563	0.001	0.378	2.08E-164	Preadipocytes_1
Col15a1.1	2.40E-167	-1.127	0.273	0.44	4.80E-164	Preadipocytes_1
Cfp.1	2.64E-167	-0.773	0.000	0.369	5.29E-164	Preadipocytes_1
Tll1	2.68E-164	0.616	0.416	0.13	5.36E-161	Preadipocytes_1
Nnat.1	6.93E-164	-1.644	0.056	0.272	1.39E-160	Preadipocytes_1
Igsf6	1.78E-163	-0.537	0.001	0.402	3.56E-160	Preadipocytes_1
C9orf72.1	3.22E-161	-0.986	0.050	0.396	6.45E-158	Preadipocytes_1
Dok2	4.83E-161	-0.717	0.000	0.387	9.66E-158	Preadipocytes_1
Anxa5.1	8.29E-160	-0.505	0.149	0.429	1.66E-156	Preadipocytes_1
Clec4b1	2.78E-158	-0.632	0.001	0.361	5.56E-155	Preadipocytes_1
Abcg1	2.66E-157	-0.760	0.002	0.431	5.31E-154	Preadipocytes_1
Ccl9	7.73E-157	-0.632	0.000	0.368	1.55E-153	Preadipocytes_1
Fnip2.1	4.49E-155	-0.932	0.640	0.788	8.97E-152	Preadipocytes_1
Gpc3.1	8.63E-155	0.814	0.573	0.369	1.73E-151	Preadipocytes_1
Fcer1g	1.59E-154	-0.628	0.000	0.357	3.18E-151	Preadipocytes_1
Ly6e	2.68E-153	-0.630	0.153	0.331	5.35E-150	Preadipocytes_1
Slc25a21.1	4.96E-153	0.702	0.464	0.202	9.92E-150	Preadipocytes_1
Malt1	1.64E-152	-0.688	0.308	0.547	3.29E-149	Preadipocytes_1
Ccr2.1	1.97E-152	-1.430	0.001	0.362	3.94E-149	Preadipocytes_1
Cst3.1	2.73E-152	-0.835	0.121	0.391	5.46E-149	Preadipocytes_1
Gask1b.1	3.51E-152	-0.780	0.356	0.566	7.01E-149	Preadipocytes_1
Ntrk3	5.29E-152	0.689	0.502	0.262	1.06E-148	Preadipocytes_1
Slc2a13.1	7.71E-152	0.663	0.637	0.417	1.54E-148	Preadipocytes_1
Rgs18	4.35E-151	-0.517	0.001	0.37	8.71E-148	Preadipocytes_1
Ctsl.1	1.44E-150	-0.669	0.178	0.37	2.87E-147	Preadipocytes_1
Nrcam	4.73E-150	0.726	0.510	0.308	9.46E-147	Preadipocytes_1

Thsd4.1	6.52E-149	-0.589	0.151	0.332	1.30E-145	Preadipocytes_1
2810004N23Ri	2.37E-148	-0.758	0.098	0.386	4.74E-145	Preadipocytes_1
Trf	2.47E-148	-0.702	0.034	0.363	4.95E-145	Preadipocytes_1
Bcl2.1	5.57E-147	-0.854	0.484	0.679	1.11E-143	Preadipocytes_1
Adam8	1.86E-146	-0.621	0.000	0.324	3.72E-143	Preadipocytes_1
Negr1.1	9.79E-145	0.658	0.822	0.733	1.96E-141	Preadipocytes_1
Cbfa2t3	1.26E-144	-0.748	0.022	0.402	2.52E-141	Preadipocytes_1
Nap1l1.1	2.20E-143	-0.608	0.162	0.436	4.39E-140	Preadipocytes_1
Fam13a.1	2.57E-143	-1.304	0.223	0.428	5.14E-140	Preadipocytes_1
Tbc1d4.1	3.22E-143	-0.699	0.625	0.815	6.43E-140	Preadipocytes_1
Ciita.1	5.40E-142	-0.981	0.025	0.406	1.08E-138	Preadipocytes_1
Serpinf1.1	6.85E-142	0.703	0.546	0.222	1.37E-138	Preadipocytes_1
Ralgapa2.1	5.39E-137	-1.091	0.444	0.679	1.08E-133	Preadipocytes_1
Npas3	8.05E-136	0.679	0.373	0.103	1.61E-132	Preadipocytes_1
Slc1a3.1	2.03E-133	-1.130	0.086	0.33	4.07E-130	Preadipocytes_1
Tmem120b.1	4.41E-132	-0.674	0.075	0.354	8.82E-129	Preadipocytes_1
Arap2.1	2.20E-131	-0.720	0.001	0.35	4.40E-128	Preadipocytes_1
Samsn1	1.35E-130	-0.677	0.001	0.339	2.70E-127	Preadipocytes_1
Lat2	4.36E-128	-0.512	0.000	0.346	8.71E-125	Preadipocytes_1
Trhde.1	9.41E-127	-0.946	0.019	0.284	1.88E-123	Preadipocytes_1
Ucp2	2.68E-126	-0.575	0.000	0.34	5.36E-123	Preadipocytes_1
Gpnmb.1	4.08E-125	-1.605	0.256	0.451	8.16E-122	Preadipocytes_1
Gusb	1.38E-124	-0.638	0.038	0.347	2.75E-121	Preadipocytes_1
Pcdh11x	3.38E-124	0.757	0.393	0.122	6.75E-121	Preadipocytes_1
Plac8.1	7.34E-123	0.709	0.481	0.302	1.47E-119	Preadipocytes_1
Hmgcs1.1	2.99E-120	-0.649	0.060	0.268	5.99E-117	Preadipocytes_1
Ctsa	7.66E-118	-0.603	0.035	0.344	1.53E-114	Preadipocytes_1
Ywhab.1	1.33E-117	-0.741	0.188	0.423	2.67E-114	Preadipocytes_1
Emb.1	1.94E-117	-0.780	0.318	0.477	3.88E-114	Preadipocytes_1
Me1.1	1.20E-116	-0.832	0.211	0.263	2.40E-113	Preadipocytes_1
Runx2	2.53E-115	-0.666	0.489	0.623	5.06E-112	Preadipocytes_1
Lmna	2.87E-114	-0.596	0.166	0.405	5.75E-111	Preadipocytes_1
Itgb2.1	9.16E-114	-0.670	0.000	0.343	1.83E-110	Preadipocytes_1
Kcnq5.1	1.93E-113	1.053	0.555	0.452	3.85E-110	Preadipocytes_1
Fgr	8.44E-110	-0.618	0.001	0.319	1.69E-106	Preadipocytes_1
Mpeg1.1	1.54E-109	-0.622	0.000	0.315	3.07E-106	Preadipocytes_1
Chst11	2.09E-109	-0.744	0.449	0.603	4.19E-106	Preadipocytes_1
Rap1gap2	1.65E-108	-0.948	0.019	0.43	3.31E-105	Preadipocytes_1
Acox1.1	2.20E-108	-1.104	0.198	0.447	4.41E-105	Preadipocytes_1
Cd226.1	5.33E-107	-1.179	0.003	0.346	1.07E-103	Preadipocytes_1
Optn.1	9.82E-104	-0.558	0.167	0.32	1.96E-100	Preadipocytes_1
Plcxd3.1	3.85E-103	0.695	0.507	0.311	7.70E-100	Preadipocytes_1
Sntb1.1	4.10E-103	-1.869	0.003	0.305	8.20E-100	Preadipocytes_1
Ctnna3.1	6.21E-102	-0.758	0.160	0.295	1.24E-98	Preadipocytes_1
Vim	6.76E-100	-0.595	0.269	0.47	1.35E-96	Preadipocytes_1
H2-DMb1	6.72E-98	-0.504	0.000	0.317	1.34E-94	Preadipocytes_1
Epha3.1	9.46E-97	0.581	0.561	0.381	1.89E-93	Preadipocytes_1
Fam129b	1.28E-95	-0.841	0.245	0.447	2.56E-92	Preadipocytes_1
Sertad2	3.21E-94	-0.564	0.422	0.564	6.42E-91	Preadipocytes_1
Il33	2.65E-93	0.685	0.411	0.17	5.30E-90	Preadipocytes_1
Ppfia2	3.75E-93	0.771	0.422	0.185	7.50E-90	Preadipocytes_1
Clybl.1	3.07E-92	-0.539	0.499	0.667	6.14E-89	Preadipocytes_1
Ghr.1	5.46E-92	-2.124	0.877	0.565	1.09E-88	Preadipocytes_1
Batf3	1.31E-91	-0.565	0.001	0.328	2.61E-88	Preadipocytes_1
Cd83	2.03E-88	-0.526	0.003	0.338	4.05E-85	Preadipocytes_1
Fcrls	7.40E-88	-0.601	0.000	0.314	1.48E-84	Preadipocytes_1
Chpt1.1	9.97E-88	-1.493	0.120	0.276	1.99E-84	Preadipocytes_1
Btg1.1	1.08E-87	-0.564	0.031	0.36	2.17E-84	Preadipocytes_1
Tes.1	2.75E-87	-0.651	0.096	0.399	5.49E-84	Preadipocytes_1
Cadps2	1.61E-86	0.724	0.475	0.286	3.22E-83	Preadipocytes_1
Mgl2.1	2.79E-84	-0.834	0.004	0.322	5.58E-81	Preadipocytes_1
Tmtc2.1	2.11E-83	0.887	0.637	0.542	4.22E-80	Preadipocytes_1

Cat.1	3.20E-83	-0.889	0.086	0.335	6.40E-80	Preadipocytes_1
Cacna1e.1	1.87E-82	-0.772	0.043	0.253	3.73E-79	Preadipocytes_1
Hacd2.1	4.45E-79	-1.697	0.134	0.349	8.91E-76	Preadipocytes_1
C1qc	1.34E-77	-0.905	0.001	0.337	2.69E-74	Preadipocytes_1
Dkk2.1	3.60E-74	0.551	0.469	0.319	7.21E-71	Preadipocytes_1
Lpgat1.1	5.14E-74	-1.974	0.216	0.395	1.03E-70	Preadipocytes_1
Ddx60.1	1.67E-72	-0.609	0.563	0.696	3.34E-69	Preadipocytes_1
Basp1.1	4.65E-70	0.724	0.553	0.465	9.31E-67	Preadipocytes_1
Fabp4.1	8.07E-68	-2.128	0.395	0.503	1.61E-64	Preadipocytes_1
F3	1.46E-67	-0.768	0.167	0.262	2.91E-64	Preadipocytes_1
Col4a2.1	6.45E-66	-1.105	0.391	0.489	1.29E-62	Preadipocytes_1
Wnt5b	7.42E-65	0.961	0.408	0.194	1.48E-61	Preadipocytes_1
Vcl.1	2.31E-63	-0.679	0.467	0.612	4.61E-60	Preadipocytes_1
Il1rl1	1.02E-61	-0.584	0.015	0.336	2.05E-58	Preadipocytes_1
Ell2.1	2.39E-61	-0.670	0.490	0.612	4.79E-58	Preadipocytes_1
Apod.1	3.30E-61	0.511	0.439	0.205	6.59E-58	Preadipocytes_1
Mme.1	2.86E-60	-0.678	0.364	0.381	5.73E-57	Preadipocytes_1
Kcnt2.1	4.93E-56	0.540	0.612	0.589	9.86E-53	Preadipocytes_1
Fam126b.1	1.32E-52	-1.125	0.202	0.395	2.64E-49	Preadipocytes_1
Pdpn	7.01E-52	0.709	0.437	0.265	1.40E-48	Preadipocytes_1
Me3	6.23E-48	0.649	0.392	0.19	1.25E-44	Preadipocytes_1
Ltc4s	1.77E-47	-0.509	0.037	0.346	3.54E-44	Preadipocytes_1
Crispld1	1.81E-46	0.762	0.371	0.133	3.62E-43	Preadipocytes_1
Sema3a	5.60E-44	0.509	0.461	0.32	1.12E-40	Preadipocytes_1
Cass4	3.94E-43	-0.518	0.001	0.322	7.88E-40	Preadipocytes_1
Chn2.1	2.83E-42	-0.840	0.483	0.559	5.65E-39	Preadipocytes_1
Adcy5.1	2.95E-42	-0.554	0.269	0.248	5.89E-39	Preadipocytes_1
Dram1.1	6.46E-42	-0.786	0.164	0.288	1.29E-38	Preadipocytes_1
Ccser1	1.66E-40	0.795	0.542	0.491	3.31E-37	Preadipocytes_1
Ryr2.1	2.12E-40	-1.017	0.001	0.264	4.25E-37	Preadipocytes_1
Plbd1	5.53E-40	-0.516	0.000	0.31	1.11E-36	Preadipocytes_1
Adam12.1	2.90E-39	-1.873	0.525	0.531	5.79E-36	Preadipocytes_1
Myo1d.1	2.92E-39	-0.617	0.314	0.423	5.84E-36	Preadipocytes_1
Ighm.1	3.23E-38	-0.649	0.000	0.323	6.45E-35	Preadipocytes_1
Fam241a.1	8.56E-38	-0.567	0.001	0.351	1.71E-34	Preadipocytes_1
Abcd2.1	1.18E-37	-0.545	0.425	0.542	2.35E-34	Preadipocytes_1
Dnm3	1.95E-36	0.555	0.632	0.636	3.89E-33	Preadipocytes_1
Rgs7.1	4.93E-36	-0.639	0.487	0.328	9.86E-33	Preadipocytes_1
Prkar2b.1	1.10E-35	-2.601	0.007	0.383	2.19E-32	Preadipocytes_1
Gpr1	2.86E-35	0.971	0.362	0.097	5.73E-32	Preadipocytes_1
Edn1	2.20E-33	0.893	0.389	0.206	4.41E-30	Preadipocytes_1
Mpp7.1	7.90E-33	-1.608	0.207	0.34	1.58E-29	Preadipocytes_1
Slc1a5.1	1.45E-32	-0.741	0.598	0.444	2.91E-29	Preadipocytes_1
Gria4.1	1.64E-32	-0.584	0.451	0.353	3.28E-29	Preadipocytes_1
Npas2	2.33E-32	0.563	0.433	0.333	4.66E-29	Preadipocytes_1
Trabd2b.1	5.63E-30	-0.939	0.336	0.356	1.13E-26	Preadipocytes_1
Inpp4b	8.13E-30	-0.779	0.444	0.533	1.63E-26	Preadipocytes_1
Fmr1.1	1.66E-28	-0.882	0.246	0.399	3.32E-25	Preadipocytes_1
Ccbe1.1	3.45E-28	-0.789	0.435	0.279	6.90E-25	Preadipocytes_1
Tox.1	1.26E-27	-0.555	0.312	0.336	2.52E-24	Preadipocytes_1
Ndnf	5.84E-27	-0.505	0.001	0.276	1.17E-23	Preadipocytes_1
Slc24a3.1	8.16E-26	-1.158	0.555	0.609	1.63E-22	Preadipocytes_1
Sgcd.1	4.83E-23	-1.056	0.741	0.559	9.66E-20	Preadipocytes_1
Skint3	4.71E-22	-0.546	0.000	0.258	9.43E-19	Preadipocytes_1
Ccn2	2.18E-20	0.586	0.354	0.125	4.35E-17	Preadipocytes_1
Akr1c18	5.96E-20	0.518	0.270	0.089	1.19E-16	Preadipocytes_1
Nrip1.1	6.00E-20	-0.519	0.821	0.659	1.20E-16	Preadipocytes_1
Prkd1.1	1.53E-19	-0.848	0.710	0.532	3.05E-16	Preadipocytes_1
Cd200	2.04E-19	0.513	0.294	0.312	4.08E-16	Preadipocytes_1
Cobl	5.72E-17	0.554	0.347	0.144	1.14E-13	Preadipocytes_1
Ldb2.1	9.23E-17	-0.546	0.524	0.448	1.85E-13	Preadipocytes_1
Cytip	1.72E-16	-0.642	0.001	0.265	3.43E-13	Preadipocytes_1

Lsamp	2.50E-13	-0.570	0.309	0.284	5.01E-10	Preadipocytes_1
Serpine2	2.96E-12	0.519	0.361	0.179	5.91E-09	Preadipocytes_1
Tmeff1.1	1.18E-11	0.542	0.353	0.194	2.36E-08	Preadipocytes_1
Kcnc2.1	4.71E-11	-0.667	0.392	0.264	9.42E-08	Preadipocytes_1
Rgs20	3.98E-10	0.522	0.314	0.323	7.96E-07	Preadipocytes_1
Has2	3.64E-09	0.713	0.339	0.156	7.29E-06	Preadipocytes_1
Fmo2.1	4.29E-08	-1.079	0.343	0.293	8.57E-05	Preadipocytes_1
Lipe.1	4.07E-06	-1.192	0.001	0.277	0.008131997	Preadipocytes_1
Sgip1.1	0.0001046	-0.881	0.469	0.501	0.20916604	Preadipocytes_1
Tln2.1	0.0001981	-0.542	0.530	0.436	0.396254521	Preadipocytes_1
Bckdhb.1	0.0003779	-0.849	0.579	0.528	0.755817721	Preadipocytes_1
Mmd.1	0.0010236	-1.784	0.001	0.285	1	Preadipocytes_1
Pde11a.1	0.0085761	-1.060	0.472	0.396	1	Preadipocytes_1
Gsn.1	0	3.009	0.970	0.65	0	Preadipocytes_2
Fmo2.2	0	2.728	0.665	0.236	0	Preadipocytes_2
Pde11a.2	0	2.618	0.693	0.363	0	Preadipocytes_2
Itgbl1.1	0	2.566	0.930	0.603	0	Preadipocytes_2
Col3a1.2	0	2.490	0.991	0.686	0	Preadipocytes_2
Col1a1.1	0	2.489	0.870	0.425	0	Preadipocytes_2
Hmcn2.1	0	2.454	0.692	0.352	0	Preadipocytes_2
Ldb2.2	0	2.353	0.819	0.4	0	Preadipocytes_2
Bmper	0	2.349	0.869	0.547	0	Preadipocytes_2
Adamts12.1	0	2.303	0.964	0.69	0	Preadipocytes_2
Sgip1.2	0	2.293	0.738	0.444	0	Preadipocytes_2
Col1a2.2	0	2.277	0.968	0.562	0	Preadipocytes_2
Meox2.1	0	2.256	0.706	0.389	0	Preadipocytes_2
Bgn.1	0	2.208	0.786	0.121	0	Preadipocytes_2
Ccbe1.2	0	2.205	0.661	0.255	0	Preadipocytes_2
Igfbp7.1	0	2.153	0.825	0.279	0	Preadipocytes_2
Col6a3	0	2.095	0.890	0.469	0	Preadipocytes_2
Gria3.1	0	2.075	0.835	0.506	0	Preadipocytes_2
Col4a1.2	0	2.061	0.907	0.448	0	Preadipocytes_2
Prickle1	0	2.019	0.783	0.503	0	Preadipocytes_2
Ror1.1	0	2.010	0.877	0.557	0	Preadipocytes_2
B3galt1	0	2.008	0.888	0.533	0	Preadipocytes_2
Smoc2.1	0	1.975	0.763	0.356	0	Preadipocytes_2
Mmp3.1	0	1.919	0.687	0.216	0	Preadipocytes_2
Sparc.1	0	1.854	0.867	0.507	0	Preadipocytes_2
Col4a2.2	0	1.788	0.846	0.386	0	Preadipocytes_2
Col6a2	0	1.731	0.819	0.324	0	Preadipocytes_2
Dcn.2	0	1.730	0.961	0.552	0	Preadipocytes_2
Abca8a.2	0	1.716	0.855	0.537	0	Preadipocytes_2
Svep1.1	0	1.705	0.928	0.569	0	Preadipocytes_2
Epha3.2	0	1.669	0.683	0.381	0	Preadipocytes_2
Auts2.1	0	1.646	0.910	0.614	0	Preadipocytes_2
Csgalnact1.1	0	1.611	0.766	0.517	0	Preadipocytes_2
Lum	0	1.598	0.674	0.084	0	Preadipocytes_2
Galnt17.2	0	1.576	0.805	0.52	0	Preadipocytes_2
Adam23	0	1.562	0.749	0.459	0	Preadipocytes_2
Boc	0	1.550	0.726	0.427	0	Preadipocytes_2
Plpp1	0	1.549	0.746	0.45	0	Preadipocytes_2
Gpm6b.1	0	1.543	0.674	0.292	0	Preadipocytes_2
Gucy1a2.1	0	1.524	0.659	0.344	0	Preadipocytes_2
Col6a1.1	0	1.486	0.746	0.207	0	Preadipocytes_2
Cacna1c	0	1.429	0.671	0.422	0	Preadipocytes_2
Gpr39	0	1.405	0.561	0.25	0	Preadipocytes_2
Serpinf1.2	0	1.395	0.679	0.239	0	Preadipocytes_2
Mgp	0	1.393	0.498	0.068	0	Preadipocytes_2
Thsd7a.2	0	1.389	0.894	0.653	0	Preadipocytes_2
Aldh1a1.2	0	1.380	0.924	0.692	0	Preadipocytes_2
Col5a3.1	0	1.343	0.807	0.513	0	Preadipocytes_2
Gdf10	0	1.336	0.503	0.052	0	Preadipocytes_2

Dclk1.2	0	1.333	0.832	0.558	0	Preadipocytes_2
Fbln2.2	0	1.331	0.804	0.385	0	Preadipocytes_2
Prickle2	0	1.307	0.724	0.41	0	Preadipocytes_2
Sparcl1	0	1.229	0.511	0.058	0	Preadipocytes_2
Gas1	0	1.217	0.562	0.127	0	Preadipocytes_2
Pcolce.1	0	1.215	0.647	0.238	0	Preadipocytes_2
Gask1b.2	0	1.197	0.746	0.461	0	Preadipocytes_2
Kalrn	0	1.083	0.520	0.112	0	Preadipocytes_2
Col15a1.2	0	1.068	0.690	0.336	0	Preadipocytes_2
Prdm6	0	1.021	0.498	0.144	0	Preadipocytes_2
Mdga2	0	1.017	0.455	0.144	0	Preadipocytes_2
Fhl2	0	0.973	0.556	0.224	0	Preadipocytes_2
Steap4	0	0.960	0.553	0.112	0	Preadipocytes_2
Mid1.2	0	0.957	0.959	0.649	0	Preadipocytes_2
Nox4.2	0	0.922	0.908	0.657	0	Preadipocytes_2
Ntf3	0	0.843	0.506	0.132	0	Preadipocytes_2
Cdh11	0	0.796	0.501	0.124	0	Preadipocytes_2
Lox	0	0.787	0.558	0.183	0	Preadipocytes_2
Abcc9	0	0.732	0.477	0.143	0	Preadipocytes_2
Egflam	0	0.663	0.431	0.09	0	Preadipocytes_2
L3mbtl4	0	0.645	0.414	0.055	0	Preadipocytes_2
Hpgds.1	0	-0.851	0.123	0.507	0	Preadipocytes_2
Pde7a.1	0	-1.247	0.325	0.781	0	Preadipocytes_2
Slc27a1.2	0	-1.270	0.072	0.51	0	Preadipocytes_2
Pfkfb3.2	0	-1.289	0.068	0.5	0	Preadipocytes_2
St8sia4.2	0	-1.344	0.054	0.478	0	Preadipocytes_2
Slc6a6.1	0	-1.448	0.177	0.63	0	Preadipocytes_2
Adgre1.2	0	-1.836	0.215	0.555	0	Preadipocytes_2
Apobec1.1	0	-1.891	0.227	0.601	0	Preadipocytes_2
Rbm47.2	0	-1.979	0.310	0.644	0	Preadipocytes_2
Hdac9.1	0	-2.248	0.225	0.607	0	Preadipocytes_2
Fbln1	3.16E-306	1.115	0.527	0.12	6.32E-303	Preadipocytes_2
Bcar3.2	6.90E-306	-1.940	0.100	0.487	1.38E-302	Preadipocytes_2
Eda.1	1.08E-305	1.507	0.845	0.669	2.15E-302	Preadipocytes_2
Csf2ra.2	4.34E-305	-1.383	0.043	0.483	8.67E-302	Preadipocytes_2
Adam12.2	2.18E-300	1.191	0.773	0.481	4.37E-297	Preadipocytes_2
Nrp2.2	7.49E-300	-0.898	0.155	0.491	1.50E-296	Preadipocytes_2
Cd84.2	1.92E-296	-1.791	0.186	0.525	3.84E-293	Preadipocytes_2
Pltp.1	1.39E-295	-1.651	0.115	0.507	2.79E-292	Preadipocytes_2
Fmo1	1.29E-293	1.354	0.649	0.323	2.57E-290	Preadipocytes_2
Clec3b.2	4.27E-291	1.213	0.797	0.444	8.55E-288	Preadipocytes_2
Plin2.1	7.71E-290	-1.438	0.181	0.552	1.54E-286	Preadipocytes_2
Alcam.1	1.54E-289	-3.020	0.265	0.579	3.08E-286	Preadipocytes_2
Ccn5	3.44E-289	0.975	0.513	0.164	6.87E-286	Preadipocytes_2
Ism1	1.57E-287	1.366	0.566	0.257	3.15E-284	Preadipocytes_2
Angpt1.1	1.38E-286	1.428	0.752	0.479	2.76E-283	Preadipocytes_2
Cd200r1.2	1.77E-283	-1.382	0.154	0.492	3.55E-280	Preadipocytes_2
Shtn1.2	2.25E-283	-1.518	0.181	0.531	4.50E-280	Preadipocytes_2
Wdfy4.2	2.26E-280	-1.185	0.077	0.483	4.53E-277	Preadipocytes_2
Mtss1.1	4.45E-279	-1.897	0.410	0.651	8.90E-276	Preadipocytes_2
Fgf7.1	2.23E-278	1.153	0.645	0.32	4.45E-275	Preadipocytes_2
Sgcd.2	6.46E-276	0.563	0.883	0.555	1.29E-272	Preadipocytes_2
Blnk.2	1.00E-273	-1.507	0.220	0.531	2.01E-270	Preadipocytes_2
Ctsb.2	5.33E-271	-1.847	0.080	0.534	1.07E-267	Preadipocytes_2
C7.1	8.17E-271	2.827	0.671	0.373	1.63E-267	Preadipocytes_2
Timp3.2	1.47E-269	1.154	0.728	0.389	2.94E-266	Preadipocytes_2
Abhd12.2	1.49E-264	-1.561	0.250	0.626	2.98E-261	Preadipocytes_2
Ptpre.1	6.18E-262	-0.834	0.099	0.426	1.24E-258	Preadipocytes_2
Eln.2	5.50E-261	1.967	0.745	0.458	1.10E-257	Preadipocytes_2
Gas2l3.2	2.57E-259	-0.796	0.107	0.459	5.15E-256	Preadipocytes_2
Psap.2	1.05E-258	-2.326	0.230	0.582	2.10E-255	Preadipocytes_2
Postn.2	2.95E-255	1.053	0.702	0.368	5.90E-252	Preadipocytes_2

Apoe.2	5.06E-251	-2.359	0.260	0.557	1.01E-247	Preadipocytes_2
Noct.2	6.70E-248	-0.812	0.174	0.497	1.34E-244	Preadipocytes_2
Prex2	5.65E-247	1.327	0.706	0.456	1.13E-243	Preadipocytes_2
Prkd1.2	2.70E-246	0.508	0.855	0.527	5.40E-243	Preadipocytes_2
Cacna1d.2	1.04E-245	-1.174	0.269	0.518	2.08E-242	Preadipocytes_2
Cd36.2	3.38E-245	-2.480	0.357	0.63	6.76E-242	Preadipocytes_2
Pik3cb.2	1.11E-244	-0.771	0.031	0.42	2.22E-241	Preadipocytes_2
Ltbp4	1.10E-242	1.337	0.504	0.108	2.20E-239	Preadipocytes_2
Pde3b.2	1.10E-242	-2.062	0.327	0.625	2.21E-239	Preadipocytes_2
Ctsc.1	1.73E-242	-1.569	0.282	0.528	3.47E-239	Preadipocytes_2
Prkg1.2	2.26E-241	1.192	0.838	0.697	4.52E-238	Preadipocytes_2
Cybb.2	5.48E-241	-1.093	0.010	0.419	1.10E-237	Preadipocytes_2
Fry.2	5.22E-239	-2.145	0.229	0.554	1.04E-235	Preadipocytes_2
Fyb.2	1.02E-237	-1.978	0.334	0.566	2.03E-234	Preadipocytes_2
Slc16a10.2	1.25E-237	-1.474	0.294	0.65	2.50E-234	Preadipocytes_2
Ehd4.2	1.75E-233	-1.166	0.196	0.5	3.50E-230	Preadipocytes_2
C5ar1.2	2.65E-233	-0.646	0.023	0.396	5.29E-230	Preadipocytes_2
Pdzrn3.2	3.50E-232	0.584	0.725	0.389	7.00E-229	Preadipocytes_2
Tenm4.2	2.87E-231	-3.200	0.083	0.48	5.74E-228	Preadipocytes_2
Nrip1.2	5.55E-231	0.619	0.919	0.662	1.11E-227	Preadipocytes_2
Aebp1.2	9.43E-231	0.836	0.637	0.24	1.89E-227	Preadipocytes_2
F3.1	4.59E-230	1.612	0.523	0.179	9.17E-227	Preadipocytes_2
Sirpa.2	1.02E-229	-1.152	0.184	0.554	2.04E-226	Preadipocytes_2
Ghr.2	3.39E-228	-1.037	0.931	0.596	6.78E-225	Preadipocytes_2
Pcdh19.2	4.09E-227	0.915	0.754	0.496	8.17E-224	Preadipocytes_2
Evl.2	1.28E-225	-1.126	0.134	0.432	2.55E-222	Preadipocytes_2
Nrep	1.02E-223	1.500	0.595	0.301	2.03E-220	Preadipocytes_2
Prkch.1	2.41E-223	-1.027	0.228	0.49	4.82E-220	Preadipocytes_2
Nckap1l.2	3.90E-223	-1.165	0.172	0.482	7.79E-220	Preadipocytes_2
Pik3ap1.1	2.45E-220	-1.226	0.283	0.527	4.89E-217	Preadipocytes_2
Sned1	1.11E-219	1.399	0.628	0.394	2.23E-216	Preadipocytes_2
Pik3r5.2	3.69E-219	-1.001	0.152	0.45	7.38E-216	Preadipocytes_2
Cd300a.2	2.36E-217	-0.988	0.032	0.431	4.73E-214	Preadipocytes_2
Cdh13.1	1.76E-216	1.783	0.670	0.461	3.51E-213	Preadipocytes_2
Aplp2.2	3.66E-214	-1.146	0.187	0.574	7.33E-211	Preadipocytes_2
Chd7.2	4.64E-214	-0.734	0.088	0.452	9.28E-211	Preadipocytes_2
Rnf149.1	8.56E-214	-0.845	0.131	0.48	1.71E-210	Preadipocytes_2
Lcp1.2	9.31E-214	-1.725	0.222	0.513	1.86E-210	Preadipocytes_2
Cygb	3.07E-213	1.046	0.472	0.092	6.15E-210	Preadipocytes_2
Clec10a.2	7.40E-213	-1.431	0.217	0.493	1.48E-209	Preadipocytes_2
Gabra3.2	8.17E-212	1.272	0.726	0.501	1.63E-208	Preadipocytes_2
Pcsk5.1	1.59E-211	1.257	0.778	0.596	3.18E-208	Preadipocytes_2
Mme.2	3.53E-210	0.826	0.618	0.328	7.06E-207	Preadipocytes_2
Myo1f.2	6.24E-210	-2.000	0.348	0.549	1.25E-206	Preadipocytes_2
Mpp1.1	1.12E-208	-0.721	0.120	0.493	2.25E-205	Preadipocytes_2
Insyn2b.2	5.61E-208	-0.928	0.196	0.471	1.12E-204	Preadipocytes_2
Atp8b4.2	2.04E-206	-1.320	0.131	0.435	4.09E-203	Preadipocytes_2
Shisa6	8.67E-206	0.555	0.449	0.173	1.73E-202	Preadipocytes_2
Ctss.2	7.82E-205	-1.272	0.016	0.416	1.56E-201	Preadipocytes_2
Eya2	2.23E-203	1.470	0.617	0.388	4.46E-200	Preadipocytes_2
Spi1.2	8.65E-203	-0.960	0.093	0.448	1.73E-199	Preadipocytes_2
Vegfc	3.82E-202	1.335	0.574	0.323	7.64E-199	Preadipocytes_2
Ccl6.2	3.89E-201	-1.195	0.019	0.398	7.79E-198	Preadipocytes_2
Hipk2.2	1.23E-200	-1.316	0.357	0.672	2.46E-197	Preadipocytes_2
Lgmn.2	1.69E-197	-1.434	0.185	0.494	3.38E-194	Preadipocytes_2
Colec12.1	3.03E-196	1.013	0.760	0.631	6.07E-193	Preadipocytes_2
Abcd2.2	3.81E-196	-0.869	0.204	0.57	7.62E-193	Preadipocytes_2
Cnnm2.2	8.85E-196	-0.746	0.338	0.652	1.77E-192	Preadipocytes_2
Ccr5.2	6.85E-195	-0.849	0.050	0.407	1.37E-191	Preadipocytes_2
Il7r.2	1.48E-194	-0.647	0.016	0.34	2.97E-191	Preadipocytes_2
Cpeb4.2	1.50E-191	-1.275	0.310	0.645	3.00E-188	Preadipocytes_2
Ptprg.2	3.74E-190	0.691	0.895	0.64	7.49E-187	Preadipocytes_2

Gramd1b.1	3.93E-190	-0.703	0.228	0.487	7.86E-187	Preadipocytes_2
Atp6v0d2.2	7.95E-190	-1.431	0.184	0.473	1.59E-186	Preadipocytes_2
Fgf13.2	5.49E-189	-1.218	0.225	0.465	1.10E-185	Preadipocytes_2
Adamtsl1.2	6.83E-189	0.571	0.886	0.709	1.37E-185	Preadipocytes_2
Mgll.1	1.25E-188	-1.087	0.243	0.488	2.50E-185	Preadipocytes_2
Ptpn22.2	3.50E-188	-1.087	0.108	0.419	7.01E-185	Preadipocytes_2
Irak2.2	1.11E-187	-0.867	0.169	0.482	2.22E-184	Preadipocytes_2
Lyz2.2	2.09E-186	-1.445	0.028	0.394	4.18E-183	Preadipocytes_2
Nabp1.2	3.08E-186	-0.753	0.069	0.404	6.16E-183	Preadipocytes_2
Adh1	1.62E-185	0.953	0.515	0.223	3.23E-182	Preadipocytes_2
Itga4.2	3.67E-183	-1.455	0.191	0.461	7.34E-180	Preadipocytes_2
Slc6a7	4.28E-183	0.696	0.429	0.131	8.56E-180	Preadipocytes_2
Msr1.2	2.82E-182	-0.729	0.169	0.439	5.63E-179	Preadipocytes_2
Lsamp.1	6.98E-182	1.647	0.497	0.25	1.40E-178	Preadipocytes_2
Pacrg.2	2.39E-181	-0.828	0.140	0.486	4.78E-178	Preadipocytes_2
Lrp12	1.95E-177	-0.552	0.275	0.575	3.91E-174	Preadipocytes_2
Gpc3.2	6.29E-175	1.100	0.617	0.387	1.26E-171	Preadipocytes_2
Lpin1.2	7.82E-175	-0.772	0.106	0.44	1.56E-171	Preadipocytes_2
Kcnt2.2	8.35E-175	1.561	0.691	0.576	1.67E-171	Preadipocytes_2
St18.2	4.51E-174	-1.328	0.243	0.479	9.02E-171	Preadipocytes_2
Ccdc80.1	1.45E-173	0.820	0.779	0.468	2.91E-170	Preadipocytes_2
Notch3	2.69E-173	0.707	0.425	0.139	5.37E-170	Preadipocytes_2
Slc5a3.2	1.20E-171	-0.639	0.080	0.413	2.40E-168	Preadipocytes_2
Lilr4b.2	1.18E-169	-0.646	0.049	0.389	2.36E-166	Preadipocytes_2
Ubash3b.2	7.62E-169	-1.265	0.300	0.501	1.52E-165	Preadipocytes_2
Ahcyl2.2	1.05E-168	-1.525	0.433	0.671	2.11E-165	Preadipocytes_2
Enpp2.2	8.65E-168	0.746	0.591	0.291	1.73E-164	Preadipocytes_2
Grk3.1	2.38E-167	-0.749	0.117	0.406	4.76E-164	Preadipocytes_2
Tec.2	2.72E-166	-1.036	0.258	0.475	5.44E-163	Preadipocytes_2
Plcb1.2	2.18E-165	-2.016	0.349	0.627	4.37E-162	Preadipocytes_2
Serpinh1	6.85E-163	1.030	0.532	0.207	1.37E-159	Preadipocytes_2
Foxp2.2	1.15E-160	0.898	0.519	0.244	2.30E-157	Preadipocytes_2
Tmem106a.2	1.93E-160	-0.695	0.134	0.456	3.87E-157	Preadipocytes_2
Osbpl8.1	3.03E-159	-1.253	0.599	0.773	6.07E-156	Preadipocytes_2
Atp6v1b2.1	1.11E-157	-0.925	0.300	0.602	2.22E-154	Preadipocytes_2
Adamts3	1.26E-157	0.582	0.401	0.137	2.52E-154	Preadipocytes_2
Chrdl1	2.08E-155	1.380	0.589	0.366	4.17E-152	Preadipocytes_2
Specc1.2	9.02E-155	-1.115	0.385	0.548	1.80E-151	Preadipocytes_2
Map1b.2	4.07E-153	1.055	0.565	0.279	8.13E-150	Preadipocytes_2
Pla2g7.2	5.25E-153	-0.820	0.107	0.397	1.05E-149	Preadipocytes_2
Itih5.2	1.14E-152	0.635	0.779	0.495	2.28E-149	Preadipocytes_2
Ptprk.1	2.40E-152	0.581	0.630	0.38	4.80E-149	Preadipocytes_2
Msrb3.1	4.26E-151	0.576	0.705	0.458	8.53E-148	Preadipocytes_2
Ptprm.1	7.67E-151	0.610	0.894	0.761	1.53E-147	Preadipocytes_2
C3ar1.1	5.79E-150	-0.568	0.006	0.356	1.16E-146	Preadipocytes_2
Lpl.2	6.62E-150	-1.411	0.358	0.617	1.32E-146	Preadipocytes_2
Mafb.2	4.52E-149	-0.938	0.081	0.396	9.05E-146	Preadipocytes_2
Tnfrsf11a.2	1.37E-147	-1.087	0.268	0.49	2.75E-144	Preadipocytes_2
Zranb3.1	4.27E-147	-0.580	0.120	0.367	8.53E-144	Preadipocytes_2
Lyve1.2	1.12E-146	-0.558	0.050	0.308	2.24E-143	Preadipocytes_2
Lgals3.2	3.45E-146	-1.630	0.027	0.372	6.90E-143	Preadipocytes_2
Arhgap25.2	5.15E-143	-1.227	0.154	0.444	1.03E-139	Preadipocytes_2
Tcirg1.1	1.87E-141	-0.632	0.114	0.403	3.74E-138	Preadipocytes_2
Ptprd.1	2.48E-140	0.546	0.805	0.549	4.97E-137	Preadipocytes_2
Fign.1	7.91E-140	0.765	0.637	0.41	1.58E-136	Preadipocytes_2
Ralgapa2.2	4.65E-139	-1.212	0.374	0.662	9.30E-136	Preadipocytes_2
Myo1d.2	4.86E-139	0.759	0.559	0.36	9.73E-136	Preadipocytes_2
Gria4.2	1.23E-138	2.462	0.539	0.348	2.46E-135	Preadipocytes_2
Rap1gap2.1	5.43E-137	-0.623	0.142	0.352	1.09E-133	Preadipocytes_2
Gabre.2	1.69E-135	0.521	0.550	0.313	3.39E-132	Preadipocytes_2
Calm2.1	2.66E-135	-0.697	0.182	0.505	5.32E-132	Preadipocytes_2
Nceh1.1	1.92E-134	-0.943	0.104	0.421	3.83E-131	Preadipocytes_2

Stab1.2	3.64E-134	-1.125	0.365	0.5	7.27E-131	Preadipocytes_2
Zfp385b.1	5.77E-133	0.690	0.508	0.3	1.15E-129	Preadipocytes_2
Atp6v1a.1	4.28E-132	-0.677	0.246	0.546	8.56E-129	Preadipocytes_2
Adap2.1	1.93E-131	-1.003	0.452	0.594	3.85E-128	Preadipocytes_2
Pde1a.2	4.04E-131	0.706	0.679	0.455	8.08E-128	Preadipocytes_2
Tns3.1	7.03E-131	-0.855	0.346	0.52	1.41E-127	Preadipocytes_2
Grn.2	1.14E-130	-0.811	0.120	0.42	2.29E-127	Preadipocytes_2
Rel.1	2.30E-130	-0.596	0.043	0.374	4.59E-127	Preadipocytes_2
Sipa1l1.2	3.01E-129	-0.961	0.358	0.615	6.02E-126	Preadipocytes_2
Cdk6.2	2.60E-128	-0.735	0.338	0.532	5.19E-125	Preadipocytes_2
Retnla.2	1.83E-127	-1.588	0.039	0.338	3.67E-124	Preadipocytes_2
Cd300lb.2	5.83E-127	-0.783	0.100	0.408	1.17E-123	Preadipocytes_2
Sema3a.1	3.68E-125	0.842	0.540	0.323	7.36E-122	Preadipocytes_2
Sdc2.2	6.86E-125	0.888	0.700	0.559	1.37E-121	Preadipocytes_2
Sfrp4.2	1.23E-124	1.181	0.523	0.239	2.46E-121	Preadipocytes_2
Slc37a2.1	1.30E-124	-0.559	0.060	0.364	2.61E-121	Preadipocytes_2
Stox2.1	2.36E-124	0.698	0.609	0.424	4.72E-121	Preadipocytes_2
Tbc1d4.2	4.90E-124	-0.848	0.588	0.797	9.79E-121	Preadipocytes_2
Myo1b	2.20E-123	0.603	0.459	0.253	4.39E-120	Preadipocytes_2
Aspn.2	3.22E-123	0.680	0.636	0.361	6.44E-120	Preadipocytes_2
Atp6v0a1.2	5.13E-123	-1.061	0.450	0.696	1.03E-119	Preadipocytes_2
Kctd12.2	1.29E-122	-0.643	0.044	0.322	2.58E-119	Preadipocytes_2
Gcnt1.1	3.58E-122	-0.514	0.151	0.391	7.16E-119	Preadipocytes_2
Mmp12.1	4.66E-121	-0.812	0.003	0.254	9.32E-118	Preadipocytes_2
Sfrp2	6.06E-121	1.153	0.408	0.1	1.21E-117	Preadipocytes_2
Hexa.2	2.28E-120	-0.687	0.162	0.448	4.55E-117	Preadipocytes_2
Slit2	4.42E-120	0.870	0.535	0.306	8.83E-117	Preadipocytes_2
Wwtr1.1	4.58E-120	0.538	0.724	0.493	9.17E-117	Preadipocytes_2
Cd44.2	5.89E-120	-1.378	0.305	0.532	1.18E-116	Preadipocytes_2
Me1.2	2.29E-119	-1.001	0.128	0.272	4.58E-116	Preadipocytes_2
Cdh19	6.49E-119	0.554	0.418	0.176	1.30E-115	Preadipocytes_2
Eepd1.2	5.16E-117	-1.422	0.121	0.421	1.03E-113	Preadipocytes_2
Kcnk13.2	1.50E-116	-1.126	0.354	0.496	3.00E-113	Preadipocytes_2
Epsti1.2	5.95E-116	-1.557	0.385	0.502	1.19E-112	Preadipocytes_2
Diaph3.1	7.71E-116	-0.827	0.342	0.551	1.54E-112	Preadipocytes_2
Tsc22d3.2	1.78E-115	-1.088	0.310	0.565	3.56E-112	Preadipocytes_2
Irs1.1	2.49E-115	0.869	0.649	0.425	4.97E-112	Preadipocytes_2
Nalcn	3.49E-115	1.266	0.435	0.156	6.97E-112	Preadipocytes_2
Cfp.2	8.07E-115	-0.671	0.016	0.318	1.61E-111	Preadipocytes_2
Ms4a7.2	5.77E-114	-0.657	0.259	0.44	1.15E-110	Preadipocytes_2
Cd180.2	8.71E-114	-0.874	0.053	0.376	1.74E-110	Preadipocytes_2
Cotl1.2	1.37E-112	-0.743	0.030	0.38	2.74E-109	Preadipocytes_2
Nxn.1	5.38E-112	0.739	0.566	0.432	1.08E-108	Preadipocytes_2
Nhs	1.37E-111	0.875	0.470	0.227	2.74E-108	Preadipocytes_2
Sort1.2	1.58E-110	-0.731	0.133	0.409	3.15E-107	Preadipocytes_2
Xkr4.1	2.20E-110	0.882	0.634	0.44	4.40E-107	Preadipocytes_2
Apod.2	3.01E-110	0.993	0.497	0.224	6.02E-107	Preadipocytes_2
Clec4a2.1	9.17E-110	-0.541	0.004	0.326	1.83E-106	Preadipocytes_2
Piezo2	2.46E-107	0.701	0.374	0.148	4.92E-104	Preadipocytes_2
Soat1.2	2.89E-107	-0.633	0.122	0.381	5.78E-104	Preadipocytes_2
Slc11a1.1	9.11E-106	-0.542	0.011	0.327	1.82E-102	Preadipocytes_2
Cadm1.2	7.45E-104	-1.414	0.260	0.431	1.49E-100	Preadipocytes_2
C1qb.2	1.18E-103	-1.004	0.042	0.352	2.37E-100	Preadipocytes_2
Adam8.1	1.35E-103	-0.547	0.005	0.28	2.69E-100	Preadipocytes_2
Ctsl.2	1.22E-102	-0.677	0.101	0.36	2.43E-99	Preadipocytes_2
Lipa.1	1.55E-102	-0.993	0.053	0.356	3.10E-99	Preadipocytes_2
Slc7a8.1	2.94E-102	-0.516	0.277	0.438	5.88E-99	Preadipocytes_2
Akr1a1.1	1.56E-101	-0.556	0.142	0.431	3.12E-98	Preadipocytes_2
Chrm3	5.02E-101	1.100	0.412	0.174	1.00E-97	Preadipocytes_2
Tnip3.1	6.67E-101	-0.691	0.017	0.3	1.33E-97	Preadipocytes_2
Fnbp1l.2	2.83E-100	-0.620	0.210	0.444	5.66E-97	Preadipocytes_2
Abcg1.1	7.84E-99	-0.620	0.116	0.352	1.57E-95	Preadipocytes_2

Sema5a.1	1.94E-98	0.865	0.523	0.334	3.88E-95	Preadipocytes_2
Fcer1g.1	6.15E-98	-0.549	0.011	0.308	1.23E-94	Preadipocytes_2
Prkcb.2	1.97E-97	-1.108	0.369	0.485	3.93E-94	Preadipocytes_2
Clec12a.1	1.13E-96	-0.615	0.006	0.331	2.26E-93	Preadipocytes_2
Ccl9.1	2.74E-96	-0.554	0.011	0.317	5.47E-93	Preadipocytes_2
Itm2b.1	2.62E-95	-0.652	0.349	0.601	5.24E-92	Preadipocytes_2
Ms4a6c.2	1.31E-94	-0.900	0.255	0.42	2.62E-91	Preadipocytes_2
Gpx3	1.71E-94	0.865	0.375	0.069	3.42E-91	Preadipocytes_2
Cd226.2	1.40E-93	-1.016	0.022	0.297	2.80E-90	Preadipocytes_2
Snhg11.1	8.28E-92	-0.715	0.234	0.322	1.66E-88	Preadipocytes_2
Adipor2.2	3.20E-91	-1.383	0.138	0.429	6.40E-88	Preadipocytes_2
Negr1.2	8.66E-91	-1.283	0.667	0.775	1.73E-87	Preadipocytes_2
Cd9.2	1.01E-90	0.773	0.476	0.277	2.03E-87	Preadipocytes_2
Pag1.2	2.65E-90	-0.560	0.276	0.472	5.29E-87	Preadipocytes_2
Dock5.1	1.05E-89	-0.520	0.183	0.419	2.09E-86	Preadipocytes_2
Dchs2	1.14E-89	0.711	0.388	0.16	2.28E-86	Preadipocytes_2
Prune2.1	1.37E-89	-0.535	0.339	0.473	2.73E-86	Preadipocytes_2
Chn2.2	4.43E-89	-1.149	0.399	0.566	8.85E-86	Preadipocytes_2
Grem2	5.27E-89	0.731	0.366	0.124	1.05E-85	Preadipocytes_2
Abtb2.1	2.36E-88	0.809	0.521	0.37	4.72E-85	Preadipocytes_2
Slc25a13.1	6.19E-88	-0.571	0.109	0.382	1.24E-84	Preadipocytes_2
Calcrl.2	7.17E-88	-0.713	0.496	0.678	1.43E-84	Preadipocytes_2
Kcnab1	8.91E-88	0.757	0.529	0.45	1.78E-84	Preadipocytes_2
Hmcn1	6.04E-87	0.583	0.367	0.136	1.21E-83	Preadipocytes_2
Ctsz.1	1.11E-84	-0.547	0.052	0.345	2.22E-81	Preadipocytes_2
Cyp7b1	3.27E-83	0.518	0.416	0.23	6.55E-80	Preadipocytes_2
Ftl1.2	9.70E-83	-0.768	0.051	0.36	1.94E-79	Preadipocytes_2
Ctsd.1	3.74E-82	-1.119	0.050	0.355	7.48E-79	Preadipocytes_2
Selenop.2	3.88E-82	-0.892	0.324	0.516	7.75E-79	Preadipocytes_2
Gm5150.1	5.87E-82	-0.548	0.128	0.344	1.17E-78	Preadipocytes_2
Ptchd1.1	1.19E-81	-0.531	0.155	0.366	2.37E-78	Preadipocytes_2
Ccr2.2	6.76E-81	-1.237	0.017	0.311	1.35E-77	Preadipocytes_2
Aoah.2	7.12E-81	-0.895	0.396	0.483	1.42E-77	Preadipocytes_2
Samsn1.1	2.02E-80	-0.569	0.012	0.292	4.04E-77	Preadipocytes_2
Cxcl14	2.12E-80	0.698	0.315	0.012	4.23E-77	Preadipocytes_2
Tnik	3.32E-80	0.628	0.527	0.378	6.63E-77	Preadipocytes_2
Abca8b.2	3.46E-80	0.932	0.582	0.427	6.93E-77	Preadipocytes_2
Clec4b1.1	1.70E-79	-0.553	0.006	0.312	3.40E-76	Preadipocytes_2
Nap1l1.2	2.40E-79	-0.562	0.140	0.404	4.79E-76	Preadipocytes_2
Malt1.1	5.96E-77	-0.679	0.300	0.517	1.19E-73	Preadipocytes_2
Atp1a1.2	6.25E-77	-0.546	0.055	0.344	1.25E-73	Preadipocytes_2
Ccn2.1	2.63E-76	0.655	0.405	0.145	5.26E-73	Preadipocytes_2
Inmt	7.29E-76	1.170	0.391	0.098	1.46E-72	Preadipocytes_2
B4galt5.2	9.37E-76	-0.537	0.399	0.578	1.87E-72	Preadipocytes_2
Csmd1.1	9.46E-76	2.038	0.461	0.263	1.89E-72	Preadipocytes_2
Plxna4.2	2.32E-75	-0.732	0.404	0.495	4.64E-72	Preadipocytes_2
Ctsa.1	2.15E-74	-0.501	0.061	0.298	4.30E-71	Preadipocytes_2
Adcy5.2	2.69E-74	-0.508	0.202	0.264	5.38E-71	Preadipocytes_2
Col4a5.2	3.34E-74	0.717	0.586	0.46	6.69E-71	Preadipocytes_2
Ms4a6b.2	1.37E-73	-0.670	0.264	0.407	2.74E-70	Preadipocytes_2
Myo1e.1	1.74E-73	-1.243	0.562	0.697	3.47E-70	Preadipocytes_2
Emb.2	2.07E-73	-0.673	0.277	0.464	4.14E-70	Preadipocytes_2
Gusb.1	3.06E-73	-0.550	0.047	0.304	6.12E-70	Preadipocytes_2
Astn2	9.52E-73	0.717	0.373	0.2	1.90E-69	Preadipocytes_2
Pparg.2	1.44E-72	-1.077	0.430	0.624	2.89E-69	Preadipocytes_2
Stap1.1	1.79E-72	-0.549	0.045	0.324	3.58E-69	Preadipocytes_2
C9orf72.2	2.14E-71	-0.866	0.067	0.347	4.28E-68	Preadipocytes_2
Nav2.2	9.97E-71	-0.616	0.302	0.457	1.99E-67	Preadipocytes_2
Anxa2.2	1.15E-70	-0.763	0.353	0.567	2.30E-67	Preadipocytes_2
Abcg3.2	4.57E-70	-0.727	0.190	0.344	9.14E-67	Preadipocytes_2
Plxnc1.2	6.94E-70	-0.708	0.189	0.386	1.39E-66	Preadipocytes_2
Sorbs1.2	1.77E-69	-2.549	0.201	0.319	3.53E-66	Preadipocytes_2

Mertk.1	2.50E-68	-0.975	0.506	0.588	5.00E-65	Preadipocytes_2
Dnm3.1	9.24E-68	0.623	0.689	0.624	1.85E-64	Preadipocytes_2
Pde7b.2	2.14E-67	-0.576	0.674	0.809	4.29E-64	Preadipocytes_2
C1qa.2	2.94E-66	-1.109	0.109	0.332	5.87E-63	Preadipocytes_2
Ncf1.1	5.18E-66	-0.600	0.087	0.335	1.04E-62	Preadipocytes_2
Tank.2	9.30E-66	-0.694	0.537	0.665	1.86E-62	Preadipocytes_2
Entpd1.2	9.77E-66	-0.794	0.349	0.52	1.95E-62	Preadipocytes_2
Mpeg1.2	1.60E-64	-0.550	0.001	0.273	3.21E-61	Preadipocytes_2
Gpnmb.2	9.48E-64	-0.775	0.296	0.418	1.90E-60	Preadipocytes_2
Rasa1.2	7.97E-63	-0.923	0.629	0.728	1.59E-59	Preadipocytes_2
Arid5b.2	1.11E-62	-0.780	0.618	0.714	2.21E-59	Preadipocytes_2
C1qtnf7.2	1.69E-62	0.824	0.581	0.448	3.38E-59	Preadipocytes_2
Aff2.1	6.47E-62	0.915	0.567	0.408	1.29E-58	Preadipocytes_2
Ly6a.2	4.53E-61	0.643	0.531	0.297	9.06E-58	Preadipocytes_2
Chsy3	1.68E-60	0.775	0.556	0.479	3.35E-57	Preadipocytes_2
Dok2.1	1.86E-60	-0.595	0.073	0.321	3.72E-57	Preadipocytes_2
Optn.2	2.54E-58	-0.664	0.118	0.309	5.09E-55	Preadipocytes_2
Bank1.2	3.90E-58	-0.953	0.299	0.404	7.79E-55	Preadipocytes_2
Errfi1.2	5.30E-58	0.759	0.610	0.516	1.06E-54	Preadipocytes_2
Itgb2.2	3.43E-57	-0.586	0.005	0.297	6.86E-54	Preadipocytes_2
Lrrtm3	6.25E-57	0.740	0.383	0.172	1.25E-53	Preadipocytes_2
Lhfpl2.2	4.66E-56	-0.624	0.332	0.486	9.31E-53	Preadipocytes_2
Dact2.2	7.38E-56	-0.548	0.185	0.266	1.48E-52	Preadipocytes_2
Trf.1	3.19E-55	-0.657	0.020	0.323	6.38E-52	Preadipocytes_2
Fgr.1	6.07E-53	-0.525	0.023	0.272	1.21E-49	Preadipocytes_2
F13a1.1	2.36E-52	-2.257	0.544	0.566	4.73E-49	Preadipocytes_2
Hpgd	6.72E-51	0.559	0.624	0.545	1.34E-47	Preadipocytes_2
Vit	1.05E-50	0.913	0.417	0.196	2.11E-47	Preadipocytes_2
Vcl.2	6.96E-50	-0.507	0.418	0.602	1.39E-46	Preadipocytes_2
Fat3.2	1.86E-49	-0.910	0.262	0.387	3.73E-46	Preadipocytes_2
Fcrls.1	1.87E-49	-0.512	0.006	0.271	3.73E-46	Preadipocytes_2
Lpgat1.2	2.58E-47	-1.920	0.144	0.386	5.16E-44	Preadipocytes_2
Tspan11	4.65E-47	0.817	0.376	0.155	9.30E-44	Preadipocytes_2
Vcam1	1.89E-46	0.530	0.482	0.416	3.77E-43	Preadipocytes_2
Fabp4.2	1.25E-45	-0.558	0.335	0.5	2.50E-42	Preadipocytes_2
Dram1.2	1.70E-42	-0.819	0.113	0.282	3.40E-39	Preadipocytes_2
Greb1l	1.97E-42	0.730	0.413	0.313	3.94E-39	Preadipocytes_2
Ciita.2	2.35E-42	-0.787	0.182	0.324	4.70E-39	Preadipocytes_2
Dmd.2	7.59E-42	0.502	0.641	0.526	1.52E-38	Preadipocytes_2
Tes.2	2.08E-41	-0.517	0.140	0.35	4.15E-38	Preadipocytes_2
Hacd2.2	2.08E-40	-1.605	0.106	0.326	4.16E-37	Preadipocytes_2
Col8a1.2	2.97E-40	0.960	0.485	0.341	5.94E-37	Preadipocytes_2
Ywhab.2	1.29E-38	-0.575	0.172	0.395	2.57E-35	Preadipocytes_2
Igfbp6.2	2.09E-38	0.608	0.476	0.242	4.18E-35	Preadipocytes_2
Inpp4b.1	4.52E-38	-0.972	0.405	0.529	9.04E-35	Preadipocytes_2
Trim25.2	4.78E-38	-0.651	0.387	0.504	9.57E-35	Preadipocytes_2
Ell2.2	1.45E-37	-0.521	0.459	0.602	2.91E-34	Preadipocytes_2
Cbfa2t3.1	2.76E-37	-0.514	0.181	0.32	5.52E-34	Preadipocytes_2
Slc25a21.2	2.93E-37	0.620	0.413	0.247	5.86E-34	Preadipocytes_2
Arap2.2	3.15E-37	-0.554	0.134	0.278	6.30E-34	Preadipocytes_2
Mgl2.2	1.87E-34	-0.686	0.022	0.276	3.74E-31	Preadipocytes_2
Card11.1	1.72E-32	-0.642	0.259	0.354	3.45E-29	Preadipocytes_2
Fnip2.2	1.37E-30	-0.596	0.656	0.765	2.74E-27	Preadipocytes_2
Igfbp5.2	2.62E-30	-0.769	0.384	0.404	5.23E-27	Preadipocytes_2
Chpt1.2	3.16E-29	-1.339	0.113	0.257	6.31E-26	Preadipocytes_2
C1qc.1	9.69E-29	-0.752	0.077	0.277	1.94E-25	Preadipocytes_2
Pitpnc1.2	7.07E-28	-0.985	0.718	0.723	1.41E-24	Preadipocytes_2
C3.2	7.73E-28	0.535	0.460	0.261	1.55E-24	Preadipocytes_2
Ighm.2	1.77E-27	-0.559	0.014	0.278	3.55E-24	Preadipocytes_2
Dusp10.1	1.06E-26	-0.564	0.177	0.369	2.13E-23	Preadipocytes_2
Plcxd3.2	2.49E-26	1.543	0.455	0.347	4.97E-23	Preadipocytes_2
Efna5.2	3.12E-26	-1.210	0.602	0.651	6.23E-23	Preadipocytes_2

Ddx60.2	8.81E-26	-0.516	0.578	0.675	1.76E-22	Preadipocytes_2
Myoc	2.67E-25	0.520	0.330	0.166	5.35E-22	Preadipocytes_2
Jazf1.1	1.50E-24	0.518	0.554	0.505	3.00E-21	Preadipocytes_2
Adgre4.2	1.78E-22	-0.715	0.172	0.315	3.56E-19	Preadipocytes_2
Fam126b.2	7.75E-22	-0.969	0.199	0.37	1.55E-18	Preadipocytes_2
Ano3.2	9.18E-22	-0.699	0.419	0.471	1.84E-18	Preadipocytes_2
Coro2a.1	7.90E-18	-0.574	0.228	0.327	1.58E-14	Preadipocytes_2
Sik2.2	1.46E-16	-1.099	0.559	0.64	2.92E-13	Preadipocytes_2
Gas6.2	4.24E-16	0.561	0.502	0.443	8.48E-13	Preadipocytes_2
2810004N23Ri	7.99E-16	-0.623	0.162	0.335	1.60E-12	Preadipocytes_2
Nrg2.1	8.68E-16	-0.518	0.417	0.424	1.74E-12	Preadipocytes_2
Hmgcs2	1.45E-15	0.513	0.314	0.134	2.89E-12	Preadipocytes_2
Dkk2.2	3.56E-15	1.164	0.429	0.347	7.11E-12	Preadipocytes_2
P3h2.2	2.37E-14	-0.765	0.441	0.435	4.74E-11	Preadipocytes_2
Cd163.2	4.25E-14	-1.274	0.442	0.44	8.50E-11	Preadipocytes_2
Mecom	1.44E-13	1.064	0.457	0.427	2.89E-10	Preadipocytes_2
Slc1a5.2	2.35E-13	-0.734	0.591	0.465	4.70E-10	Preadipocytes_2
Cat.2	1.80E-12	-0.680	0.103	0.298	3.60E-09	Preadipocytes_2
Acsl1.1	3.02E-12	-2.815	0.059	0.26	6.04E-09	Preadipocytes_2
Car8.2	2.32E-11	-0.609	0.363	0.353	4.64E-08	Preadipocytes_2
Ednrb.2	3.93E-11	-0.761	0.398	0.403	7.87E-08	Preadipocytes_2
Cilp	7.86E-11	0.913	0.321	0.139	1.57E-07	Preadipocytes_2
Acox1.2	9.27E-11	-0.833	0.283	0.397	1.85E-07	Preadipocytes_2
Mpp7.2	3.29E-10	-1.387	0.213	0.321	6.59E-07	Preadipocytes_2
Aldh1a3.2	4.23E-10	-0.533	0.346	0.318	8.46E-07	Preadipocytes_2
Tns1.1	1.37E-09	-0.512	0.838	0.863	2.73E-06	Preadipocytes_2
Sntg1.2	1.25E-08	-0.511	0.594	0.565	2.50E-05	Preadipocytes_2
Mctp2.1	3.19E-08	-0.513	0.372	0.379	6.37E-05	Preadipocytes_2
Thsd4.2	2.98E-07	-0.633	0.270	0.285	0.000596857	Preadipocytes_2
Bckdhb.2	1.35E-06	-0.845	0.476	0.555	0.00269319	Preadipocytes_2
Plekhg5.1	1.46E-06	-0.736	0.472	0.475	0.002924376	Preadipocytes_2
Fmr1.2	1.55E-06	-0.689	0.254	0.377	0.003100398	Preadipocytes_2
Rorb.2	9.84E-05	-0.705	0.483	0.52	0.196756885	Preadipocytes_2
Sema3e.2	0.000177	-1.412	0.531	0.536	0.354035107	Preadipocytes_2
Tmem135.2	0.0002423	-0.867	0.563	0.611	0.484653087	Preadipocytes_2
Mamdc2.2	0.0004946	-0.686	0.418	0.397	0.989283882	Preadipocytes_2
Kcnq1	0.0028368	-0.511	0.545	0.556	1	Preadipocytes_2
Prkar2b.2	0.0087947	-2.212	0.199	0.295	1	Preadipocytes_2
Diaph3.2	4.47E-67	3.134	0.973	0.513	8.94E-64	Preadipocytes_3
Cenpp	4.37E-48	2.583	0.894	0.509	8.74E-45	Preadipocytes_3
Ezh2	1.76E-39	1.823	0.858	0.467	3.53E-36	Preadipocytes_3
Atad2	7.95E-39	1.763	0.841	0.479	1.59E-35	Preadipocytes_3
Vcan.2	4.66E-38	1.538	0.947	0.553	9.32E-35	Preadipocytes_3
Postn.3	3.22E-37	2.059	0.832	0.42	6.44E-34	Preadipocytes_3
Kif15	1.94E-36	1.548	0.717	0.298	3.88E-33	Preadipocytes_3
Neil3	1.20E-34	1.704	0.673	0.27	2.39E-31	Preadipocytes_3
Knl1	1.32E-34	1.615	0.690	0.279	2.63E-31	Preadipocytes_3
Kif4	1.34E-33	1.488	0.611	0.228	2.67E-30	Preadipocytes_3
Pola1.1	5.89E-33	1.711	0.867	0.648	1.18E-29	Preadipocytes_3
Top2a	1.74E-32	1.823	0.664	0.27	3.47E-29	Preadipocytes_3
Col1a1.2	9.18E-31	1.456	0.903	0.496	1.84E-27	Preadipocytes_3
Ncapg2	8.34E-30	1.641	0.690	0.331	1.67E-26	Preadipocytes_3
Mfap5.2	5.27E-29	1.200	0.938	0.52	1.05E-25	Preadipocytes_3
Col3a1.3	8.60E-29	1.234	1.000	0.734	1.72E-25	Preadipocytes_3
Anln	1.22E-28	1.882	0.673	0.321	2.44E-25	Preadipocytes_3
Zwilch	1.39E-28	1.304	0.673	0.329	2.77E-25	Preadipocytes_3
Col1a2.3	1.90E-27	1.171	0.991	0.626	3.81E-24	Preadipocytes_3
Racgap1	1.91E-27	1.164	0.549	0.152	3.83E-24	Preadipocytes_3
Kif11	3.89E-26	1.465	0.637	0.287	7.78E-23	Preadipocytes_3
Tpx2	1.17E-25	1.352	0.593	0.266	2.33E-22	Preadipocytes_3
Arnt2	2.26E-25	0.772	0.460	0.13	4.53E-22	Preadipocytes_3
Dclk1.3	2.65E-24	1.372	0.867	0.601	5.29E-21	Preadipocytes_3

Mki67	6.14E-24	1.296	0.611	0.28	1.23E-20	Preadipocytes_3
Smc2	2.35E-22	0.887	0.540	0.207	4.70E-19	Preadipocytes_3
Efcab11	5.61E-22	1.364	0.628	0.352	1.12E-18	Preadipocytes_3
Cenph	8.70E-22	1.076	0.522	0.191	1.74E-18	Preadipocytes_3
Mms22l	1.45E-21	1.571	0.708	0.469	2.90E-18	Preadipocytes_3
Adamtsl1.3	4.70E-21	1.453	0.912	0.737	9.39E-18	Preadipocytes_3
Cdh2	6.08E-21	1.231	0.540	0.235	1.22E-17	Preadipocytes_3
Ugdh.1	7.83E-21	1.318	0.788	0.459	1.57E-17	Preadipocytes_3
Kif20b	8.27E-21	1.109	0.540	0.255	1.65E-17	Preadipocytes_3
Sparc.2	1.15E-20	1.325	0.841	0.564	2.30E-17	Preadipocytes_3
Bub1	1.55E-20	0.884	0.425	0.12	3.10E-17	Preadipocytes_3
Lmnb1	4.37E-20	1.184	0.637	0.353	8.74E-17	Preadipocytes_3
Cit	6.95E-20	1.200	0.575	0.293	1.39E-16	Preadipocytes_3
Prc1	8.36E-20	1.268	0.522	0.241	1.67E-16	Preadipocytes_3
Csgalnact1.2	8.80E-20	1.180	0.779	0.556	1.76E-16	Preadipocytes_3
Smc4.1	9.63E-20	1.156	0.673	0.422	1.93E-16	Preadipocytes_3
Col5a3.2	1.35E-19	1.522	0.796	0.559	2.69E-16	Preadipocytes_3
Col8a1.3	1.89E-19	1.596	0.637	0.363	3.78E-16	Preadipocytes_3
Rbm47.3	2.03E-19	-2.008	0.204	0.592	4.06E-16	Preadipocytes_3
Pcdh19.3	3.15E-19	1.036	0.796	0.536	6.30E-16	Preadipocytes_3
Shcbp1	3.37E-19	0.879	0.504	0.237	6.74E-16	Preadipocytes_3
Wdfy4.3	3.43E-19	-1.171	0.027	0.419	6.86E-16	Preadipocytes_3
Kntc1	3.76E-19	1.087	0.496	0.205	7.51E-16	Preadipocytes_3
Depdc1a	4.57E-19	0.907	0.407	0.13	9.15E-16	Preadipocytes_3
Cd84.3	4.64E-19	-1.753	0.071	0.472	9.28E-16	Preadipocytes_3
Bub1b	6.99E-19	0.716	0.407	0.137	1.40E-15	Preadipocytes_3
Pltp.2	1.55E-18	-1.733	0.009	0.446	3.09E-15	Preadipocytes_3
Insyn2b.3	2.13E-18	-0.905	0.053	0.429	4.27E-15	Preadipocytes_3
Slfn9	3.44E-18	0.675	0.398	0.151	6.89E-15	Preadipocytes_3
Mastl	3.82E-18	1.160	0.558	0.312	7.65E-15	Preadipocytes_3
Hdac9.2	4.91E-18	-2.238	0.124	0.547	9.83E-15	Preadipocytes_3
Brip1	1.15E-17	1.485	0.611	0.395	2.31E-14	Preadipocytes_3
Ms4a6c.3	1.51E-17	-0.973	0.009	0.396	3.01E-14	Preadipocytes_3
Iqgap3	2.51E-17	1.166	0.469	0.19	5.02E-14	Preadipocytes_3
Rad18.1	2.51E-17	1.040	0.673	0.471	5.02E-14	Preadipocytes_3
Fbln2.3	3.00E-17	0.818	0.788	0.451	6.00E-14	Preadipocytes_3
Ranbp17.1	3.39E-17	1.090	0.593	0.295	6.79E-14	Preadipocytes_3
Galnt17.3	6.94E-17	1.018	0.788	0.565	1.39E-13	Preadipocytes_3
Enah.1	1.07E-16	0.769	0.602	0.312	2.14E-13	Preadipocytes_3
Rad54b	1.53E-16	0.867	0.478	0.215	3.06E-13	Preadipocytes_3
Uhrf1	3.26E-16	0.556	0.372	0.128	6.53E-13	Preadipocytes_3
Itga4.3	3.43E-16	-1.487	0.044	0.419	6.86E-13	Preadipocytes_3
Mtss1.2	3.79E-16	-2.156	0.265	0.614	7.59E-13	Preadipocytes_3
Ctsc.2	5.14E-16	-1.779	0.106	0.49	1.03E-12	Preadipocytes_3
Lgmn.3	5.77E-16	-1.415	0.044	0.446	1.15E-12	Preadipocytes_3
Cd300a.3	6.53E-16	-0.937	0.000	0.368	1.31E-12	Preadipocytes_3
Robo1.2	7.66E-16	0.768	0.796	0.564	1.53E-12	Preadipocytes_3
Myo1f.3	1.39E-15	-2.057	0.159	0.519	2.78E-12	Preadipocytes_3
Csf2ra.3	2.03E-15	-1.241	0.053	0.414	4.06E-12	Preadipocytes_3
Cenpe	2.99E-15	1.177	0.504	0.246	5.98E-12	Preadipocytes_3
Tead1.2	3.00E-15	0.737	0.885	0.668	6.00E-12	Preadipocytes_3
Cacna1c.1	3.18E-15	1.602	0.637	0.462	6.37E-12	Preadipocytes_3
Bmp6.2	3.73E-15	1.039	0.673	0.449	7.47E-12	Preadipocytes_3
Ect2	4.76E-15	1.397	0.549	0.331	9.52E-12	Preadipocytes_3
Col14a1.2	5.30E-15	0.728	0.885	0.668	1.06E-11	Preadipocytes_3
Blnk.3	6.07E-15	-1.525	0.133	0.483	1.21E-11	Preadipocytes_3
Adgre1.3	7.24E-15	-1.862	0.142	0.501	1.45E-11	Preadipocytes_3
Fyb.3	1.06E-14	-2.040	0.177	0.531	2.13E-11	Preadipocytes_3
Mcm6	1.27E-14	0.568	0.407	0.217	2.53E-11	Preadipocytes_3
Arhgap25.3	1.48E-14	-1.166	0.044	0.399	2.96E-11	Preadipocytes_3
Mertk.2	1.79E-14	-1.430	0.221	0.577	3.58E-11	Preadipocytes_3
Ctss.3	1.93E-14	-1.191	0.009	0.353	3.85E-11	Preadipocytes_3

Myo1b.1	2.01E-14	0.807	0.513	0.285	4.01E-11	Preadipocytes_3
Tmtc2.2	2.67E-14	1.716	0.717	0.567	5.34E-11	Preadipocytes_3
Ncapg	2.85E-14	0.796	0.407	0.174	5.71E-11	Preadipocytes_3
Melk	3.55E-14	0.878	0.460	0.219	7.10E-11	Preadipocytes_3
Tnfrsf11a.3	3.72E-14	-1.099	0.115	0.456	7.44E-11	Preadipocytes_3
Aoah.3	4.14E-14	-0.978	0.124	0.471	8.28E-11	Preadipocytes_3
Lcp1.3	4.22E-14	-1.680	0.124	0.468	8.44E-11	Preadipocytes_3
Brca1	5.03E-14	1.118	0.558	0.358	1.01E-10	Preadipocytes_3
Pik3ap1.2	5.76E-14	-1.186	0.150	0.49	1.15E-10	Preadipocytes_3
Sdc2.3	9.27E-14	0.866	0.779	0.581	1.85E-10	Preadipocytes_3
Kif14	9.80E-14	0.655	0.354	0.125	1.96E-10	Preadipocytes_3
Ccr5.3	1.26E-13	-0.804	0.018	0.35	2.52E-10	Preadipocytes_3
Adap2.2	1.32E-13	-1.306	0.230	0.573	2.65E-10	Preadipocytes_3
Bgn.2	1.33E-13	0.855	0.540	0.228	2.65E-10	Preadipocytes_3
Ccl6.3	2.23E-13	-1.120	0.018	0.338	4.47E-10	Preadipocytes_3
Il7r.3	2.39E-13	-0.581	0.018	0.289	4.79E-10	Preadipocytes_3
Bard1	4.50E-13	1.116	0.496	0.319	9.01E-10	Preadipocytes_3
St8sia4.3	4.59E-13	-1.306	0.088	0.411	9.17E-10	Preadipocytes_3
Hells	5.35E-13	1.285	0.566	0.398	1.07E-09	Preadipocytes_3
Col12a1.2	5.35E-13	0.816	0.628	0.381	1.07E-09	Preadipocytes_3
Spi1.3	7.11E-13	-0.864	0.088	0.392	1.42E-09	Preadipocytes_3
Shtn1.3	7.63E-13	-1.518	0.159	0.476	1.53E-09	Preadipocytes_3
Serpinh1.1	1.08E-12	0.782	0.558	0.259	2.16E-09	Preadipocytes_3
Pik3r5.3	1.14E-12	-0.942	0.062	0.403	2.28E-09	Preadipocytes_3
Tec.3	1.34E-12	-1.013	0.115	0.442	2.68E-09	Preadipocytes_3
Ncapd2	1.65E-12	1.039	0.540	0.382	3.31E-09	Preadipocytes_3
Incenp	1.67E-12	0.794	0.460	0.252	3.34E-09	Preadipocytes_3
Lyz2.3	1.91E-12	-1.417	0.000	0.336	3.82E-09	Preadipocytes_3
Alcam.2	2.00E-12	-2.731	0.221	0.53	3.99E-09	Preadipocytes_3
Cdca2	2.09E-12	1.090	0.496	0.295	4.18E-09	Preadipocytes_3
Itm2b.2	2.22E-12	-1.213	0.177	0.563	4.44E-09	Preadipocytes_3
Igf1.2	2.57E-12	-1.433	0.204	0.609	5.14E-09	Preadipocytes_3
F13a1.2	3.23E-12	-2.910	0.265	0.565	6.46E-09	Preadipocytes_3
Clspn	3.77E-12	0.898	0.451	0.232	7.53E-09	Preadipocytes_3
Gfpt2.2	4.08E-12	0.539	0.832	0.563	8.16E-09	Preadipocytes_3
Ckap2	5.43E-12	1.097	0.478	0.277	1.09E-08	Preadipocytes_3
Apobec1.2	6.13E-12	-2.053	0.195	0.542	1.23E-08	Preadipocytes_3
Nr3c2.1	6.49E-12	-0.926	0.327	0.692	1.30E-08	Preadipocytes_3
Clec10a.3	6.60E-12	-1.447	0.124	0.45	1.32E-08	Preadipocytes_3
Atp8b4.3	7.04E-12	-1.243	0.053	0.387	1.41E-08	Preadipocytes_3
Plin2.2	7.77E-12	-1.486	0.106	0.494	1.55E-08	Preadipocytes_3
Col6a3.1	9.12E-12	1.137	0.743	0.537	1.82E-08	Preadipocytes_3
Pde4d.2	1.29E-11	-1.661	0.301	0.645	2.59E-08	Preadipocytes_3
Apoe.3	1.36E-11	-2.658	0.195	0.51	2.72E-08	Preadipocytes_3
Slc6a6.2	1.52E-11	-1.292	0.177	0.559	3.04E-08	Preadipocytes_3
Col6a2.1	1.91E-11	0.821	0.664	0.404	3.82E-08	Preadipocytes_3
Msr1.3	2.47E-11	-0.723	0.106	0.397	4.93E-08	Preadipocytes_3
Gas6.3	3.48E-11	-0.873	0.106	0.456	6.95E-08	Preadipocytes_3
Psap.3	4.22E-11	-2.247	0.168	0.526	8.44E-08	Preadipocytes_3
Col6a1.2	4.24E-11	0.631	0.584	0.293	8.48E-08	Preadipocytes_3
Sgo2a	4.25E-11	0.792	0.398	0.19	8.50E-08	Preadipocytes_3
Epsti1.3	5.35E-11	-1.591	0.195	0.485	1.07E-07	Preadipocytes_3
Cd248.2	6.42E-11	0.765	0.549	0.242	1.28E-07	Preadipocytes_3
Gna14	8.89E-11	0.726	0.416	0.224	1.78E-07	Preadipocytes_3
Trim59	9.32E-11	0.710	0.336	0.153	1.86E-07	Preadipocytes_3
Tns3.2	9.79E-11	-0.936	0.168	0.494	1.96E-07	Preadipocytes_3
Mis18bp1	1.31E-10	1.035	0.478	0.306	2.63E-07	Preadipocytes_3
Pitpnc1.3	1.33E-10	-1.728	0.513	0.724	2.66E-07	Preadipocytes_3
Cd36.3	1.45E-10	-2.270	0.345	0.587	2.89E-07	Preadipocytes_3
Hpgds.2	1.63E-10	-0.818	0.142	0.446	3.26E-07	Preadipocytes_3
Abhd12.3	1.63E-10	-1.588	0.204	0.567	3.26E-07	Preadipocytes_3
Basp1.2	1.98E-10	0.732	0.655	0.488	3.97E-07	Preadipocytes_3

Cacna1d.3	2.61E-10	-1.033	0.159	0.479	5.22E-07	Preadipocytes_3
Ntn4.2	2.74E-10	0.664	0.628	0.4	5.47E-07	Preadipocytes_3
Cd200r1.3	3.15E-10	-1.285	0.133	0.439	6.30E-07	Preadipocytes_3
Galnt18	3.29E-10	0.920	0.522	0.323	6.59E-07	Preadipocytes_3
Wdhd1	3.67E-10	0.818	0.469	0.348	7.33E-07	Preadipocytes_3
Pole	3.99E-10	0.977	0.478	0.319	7.98E-07	Preadipocytes_3
Kcnk13.3	4.03E-10	-1.236	0.177	0.475	8.07E-07	Preadipocytes_3
Ms4a7.3	4.29E-10	-0.650	0.133	0.412	8.59E-07	Preadipocytes_3
Ptpn22.3	5.60E-10	-0.970	0.088	0.37	1.12E-06	Preadipocytes_3
C1qb.3	6.14E-10	-0.989	0.018	0.303	1.23E-06	Preadipocytes_3
Cep55	6.70E-10	0.613	0.283	0.096	1.34E-06	Preadipocytes_3
Rin2.2	6.83E-10	-0.792	0.451	0.736	1.37E-06	Preadipocytes_3
Mamdc2.3	7.02E-10	-1.114	0.088	0.403	1.40E-06	Preadipocytes_3
Igfbp5.3	7.09E-10	0.633	0.681	0.399	1.42E-06	Preadipocytes_3
Ctsb.3	7.81E-10	-1.753	0.142	0.462	1.56E-06	Preadipocytes_3
Kif18a	9.10E-10	0.614	0.398	0.263	1.82E-06	Preadipocytes_3
Nckap1l.3	9.97E-10	-1.106	0.142	0.433	1.99E-06	Preadipocytes_3
C4b	1.21E-09	-1.172	0.159	0.533	2.43E-06	Preadipocytes_3
Nrp2.3	1.33E-09	-0.908	0.115	0.438	2.67E-06	Preadipocytes_3
Prss23.2	1.39E-09	0.706	0.531	0.237	2.77E-06	Preadipocytes_3
Abtb2.2	1.50E-09	0.645	0.558	0.394	3.00E-06	Preadipocytes_3
Gen1	2.48E-09	0.813	0.398	0.243	4.96E-06	Preadipocytes_3
Prkcb.3	2.62E-09	-1.286	0.186	0.468	5.25E-06	Preadipocytes_3
Tacc3	2.67E-09	0.697	0.336	0.199	5.34E-06	Preadipocytes_3
Npas2.1	3.89E-09	0.708	0.513	0.359	7.77E-06	Preadipocytes_3
St18.3	3.90E-09	-1.215	0.159	0.442	7.79E-06	Preadipocytes_3
Retnla.3	3.95E-09	-1.663	0.000	0.291	7.90E-06	Preadipocytes_3
Ckap2l	4.16E-09	0.772	0.416	0.234	8.33E-06	Preadipocytes_3
Pde7b.3	4.27E-09	-1.004	0.513	0.789	8.53E-06	Preadipocytes_3
Atp6v0d2.3	5.24E-09	-1.315	0.168	0.427	1.05E-05	Preadipocytes_3
C1qa.3	7.03E-09	-1.111	0.000	0.297	1.41E-05	Preadipocytes_3
Thrb.1	1.13E-08	-1.030	0.584	0.808	2.26E-05	Preadipocytes_3
Piezo2.1	1.44E-08	0.641	0.345	0.184	2.87E-05	Preadipocytes_3
Dok2.2	1.62E-08	-0.592	0.000	0.283	3.24E-05	Preadipocytes_3
Polq	1.79E-08	0.792	0.425	0.279	3.57E-05	Preadipocytes_3
Lgals3.3	1.83E-08	-1.480	0.044	0.317	3.66E-05	Preadipocytes_3
Fcer1g.2	1.96E-08	-0.504	0.000	0.261	3.92E-05	Preadipocytes_3
Cfp.3	1.99E-08	-0.617	0.000	0.27	3.98E-05	Preadipocytes_3
Timp3.3	2.11E-08	0.599	0.664	0.443	4.21E-05	Preadipocytes_3
Slc27a1.3	2.16E-08	-1.067	0.133	0.44	4.33E-05	Preadipocytes_3
Tnfrsf11b	2.32E-08	0.533	0.301	0.131	4.65E-05	Preadipocytes_3
Adgre4.3	2.72E-08	-0.713	0.044	0.294	5.44E-05	Preadipocytes_3
Aplp2.3	3.76E-08	-1.071	0.168	0.513	7.51E-05	Preadipocytes_3
Ccl9.2	4.28E-08	-0.523	0.000	0.269	8.56E-05	Preadipocytes_3
Stap1.2	5.77E-08	-0.516	0.027	0.28	0.000115491	Preadipocytes_3
Cadm1.3	6.10E-08	-1.366	0.159	0.405	0.000121925	Preadipocytes_3
Lig1	6.13E-08	0.594	0.301	0.162	0.0001225	Preadipocytes_3
Nusap1	6.40E-08	0.892	0.442	0.302	0.000128055	Preadipocytes_3
Chd7.3	6.41E-08	-0.648	0.115	0.394	0.000128233	Preadipocytes_3
Ncf1.2	6.84E-08	-0.578	0.035	0.296	0.000136721	Preadipocytes_3
Cd300lb.3	8.07E-08	-0.726	0.088	0.359	0.000161318	Preadipocytes_3
Ccr2.3	8.18E-08	-1.200	0.018	0.264	0.000163528	Preadipocytes_3
Clybl.2	1.04E-07	-0.530	0.283	0.623	0.000208721	Preadipocytes_3
Stab1.3	1.17E-07	-1.145	0.239	0.48	0.000234695	Preadipocytes_3
Kif23	1.20E-07	0.688	0.381	0.242	0.000239355	Preadipocytes_3
Celf4.1	1.37E-07	-0.540	0.071	0.334	0.000274176	Preadipocytes_3
Grk3.2	1.42E-07	-0.587	0.115	0.361	0.000283584	Preadipocytes_3
Sirpa.3	1.45E-07	-1.079	0.168	0.496	0.000289477	Preadipocytes_3
Atp6v0a1.3	1.45E-07	-1.198	0.407	0.657	0.000290605	Preadipocytes_3
P3h2.3	1.48E-07	-0.882	0.186	0.438	0.000295641	Preadipocytes_3
Tnip3.2	1.68E-07	-0.650	0.018	0.255	0.000336036	Preadipocytes_3
Auts2.2	1.68E-07	0.553	0.770	0.662	0.000336097	Preadipocytes_3

Specc1.3	1.75E-07	-1.019	0.283	0.523	0.000349515	Preadipocytes_3
Chsy3.1	1.91E-07	0.814	0.575	0.491	0.000382742	Preadipocytes_3
Efna5.3	2.39E-07	0.621	0.770	0.642	0.00047855	Preadipocytes_3
Stil	2.44E-07	0.741	0.451	0.313	0.000487917	Preadipocytes_3
Cfh.2	2.46E-07	-1.816	0.248	0.555	0.000491021	Preadipocytes_3
Cybb.3	2.50E-07	-0.977	0.106	0.353	0.000499368	Preadipocytes_3
Ncam1	2.65E-07	1.105	0.310	0.146	0.000530979	Preadipocytes_3
Prkch.2	2.83E-07	-1.024	0.168	0.449	0.000565061	Preadipocytes_3
Cip2a	3.11E-07	0.556	0.319	0.182	0.000621365	Preadipocytes_3
Cenpf	3.14E-07	1.221	0.425	0.277	0.000627569	Preadipocytes_3
Pik3cb.3	3.25E-07	-0.714	0.053	0.358	0.000649234	Preadipocytes_3
Nrg2.2	3.31E-07	-0.586	0.168	0.425	0.000661701	Preadipocytes_3
Dtl	3.62E-07	1.101	0.434	0.282	0.000723178	Preadipocytes_3
Bank1.3	4.18E-07	-0.995	0.115	0.389	0.000835154	Preadipocytes_3
Igfbp7.2	4.25E-07	0.801	0.558	0.368	0.000850167	Preadipocytes_3
Plxnc1.3	4.33E-07	-0.691	0.097	0.356	0.000865854	Preadipocytes_3
Ptpre.2	4.71E-07	-0.857	0.097	0.374	0.000941291	Preadipocytes_3
Dbf4	6.06E-07	0.669	0.372	0.279	0.001211811	Preadipocytes_3
Adam12.3	7.64E-07	0.897	0.637	0.528	0.001528577	Preadipocytes_3
Pla2g7.3	8.02E-07	-0.721	0.106	0.351	0.001603124	Preadipocytes_3
Tsc22d3.3	8.16E-07	-1.363	0.221	0.526	0.001631614	Preadipocytes_3
Kctd12.3	9.52E-07	-0.620	0.018	0.278	0.001903673	Preadipocytes_3
Ngf.2	9.91E-07	0.574	0.478	0.308	0.001981688	Preadipocytes_3
Ror1.2	1.13E-06	0.709	0.690	0.609	0.00225467	Preadipocytes_3
Cpeb4.3	1.31E-06	-1.261	0.327	0.592	0.002610558	Preadipocytes_3
Plekhg5.2	1.74E-06	-1.109	0.204	0.477	0.003480252	Preadipocytes_3
Tenm3	1.85E-06	1.139	0.319	0.141	0.003705582	Preadipocytes_3
Fry.3	2.16E-06	-1.747	0.283	0.502	0.004320505	Preadipocytes_3
Plxna4.3	2.25E-06	-0.900	0.248	0.482	0.004503742	Preadipocytes_3
C5ar1.3	2.29E-06	-0.577	0.106	0.336	0.004578666	Preadipocytes_3
Selenop.3	2.36E-06	-1.293	0.204	0.486	0.004726658	Preadipocytes_3
Aspm	2.58E-06	0.909	0.363	0.229	0.005151497	Preadipocytes_3
Jazf1.2	2.71E-06	0.696	0.602	0.512	0.005419043	Preadipocytes_3
Ttk	2.73E-06	0.754	0.389	0.224	0.005461084	Preadipocytes_3
Ehd4.3	2.87E-06	-1.163	0.177	0.452	0.005740851	Preadipocytes_3
Osbpl8.2	3.32E-06	-1.111	0.646	0.745	0.006632032	Preadipocytes_3
Cotl1.3	3.38E-06	-0.611	0.053	0.324	0.006762362	Preadipocytes_3
Abcg3.3	3.40E-06	-0.709	0.080	0.321	0.006809873	Preadipocytes_3
Rad51	3.67E-06	0.928	0.434	0.332	0.007347631	Preadipocytes_3
Lpl.3	3.75E-06	-1.799	0.336	0.576	0.007509195	Preadipocytes_3
Ciita.3	3.91E-06	-0.743	0.062	0.303	0.007823818	Preadipocytes_3
Grn.3	4.01E-06	-0.804	0.080	0.373	0.008015802	Preadipocytes_3
Cd163.3	4.80E-06	-1.428	0.204	0.442	0.009591021	Preadipocytes_3
Hmmr	5.49E-06	0.740	0.310	0.153	0.010983973	Preadipocytes_3
Ednrb.3	5.54E-06	-0.833	0.159	0.404	0.011075428	Preadipocytes_3
Gcnt1.2	6.11E-06	-0.536	0.115	0.353	0.012227865	Preadipocytes_3
Rgs7.2	6.93E-06	-0.676	0.195	0.373	0.013869302	Preadipocytes_3
Lilr4b.3	7.16E-06	-0.575	0.106	0.335	0.014325411	Preadipocytes_3
Slc7a8.2	7.40E-06	-0.666	0.159	0.413	0.014805711	Preadipocytes_3
Ahcyl2.3	7.71E-06	-1.350	0.487	0.633	0.015416266	Preadipocytes_3
Ndc80	7.74E-06	0.598	0.336	0.192	0.015482331	Preadipocytes_3
Slc16a10.3	8.19E-06	-1.152	0.363	0.594	0.016373796	Preadipocytes_3
Rasa1.3	9.56E-06	-1.034	0.549	0.713	0.019129849	Preadipocytes_3
Cst3.2	1.00E-05	-0.781	0.044	0.319	0.019996486	Preadipocytes_3
Ptchd1.2	1.02E-05	-0.528	0.106	0.333	0.020320247	Preadipocytes_3
Gpnmb.3	1.41E-05	-1.355	0.177	0.399	0.028115226	Preadipocytes_3
Mpp7.3	1.50E-05	-0.813	0.434	0.302	0.03005272	Preadipocytes_3
Pak3	1.52E-05	0.739	0.372	0.23	0.030402206	Preadipocytes_3
Atp6v1b2.2	1.57E-05	-0.967	0.310	0.554	0.031412288	Preadipocytes_3
Cd226.3	1.67E-05	-0.888	0.044	0.253	0.033456577	Preadipocytes_3
Gramd1b.2	1.86E-05	-0.699	0.195	0.446	0.037270181	Preadipocytes_3
Fam214a.1	1.90E-05	-0.739	0.478	0.722	0.038044467	Preadipocytes_3

Fat3.3	1.93E-05	-0.860	0.159	0.368	0.038597645	Preadipocytes_3
Ftl1.3	2.30E-05	-0.744	0.053	0.311	0.045979974	Preadipocytes_3
Nrep.1	2.53E-05	0.563	0.460	0.348	0.050650296	Preadipocytes_3
Mafb.3	3.31E-05	-0.887	0.106	0.346	0.066103419	Preadipocytes_3
Mpp1.2	3.31E-05	-0.722	0.115	0.434	0.066170238	Preadipocytes_3
Ccnf	3.48E-05	0.663	0.301	0.22	0.069619681	Preadipocytes_3
Cd44.3	3.58E-05	0.528	0.664	0.494	0.071506292	Preadipocytes_3
Ctsz.2	3.59E-05	-0.507	0.035	0.299	0.071890955	Preadipocytes_3
P2rx7.2	4.03E-05	-0.692	0.115	0.42	0.080680359	Preadipocytes_3
Il34	4.22E-05	0.543	0.345	0.223	0.084386179	Preadipocytes_3
Ms4a6b.3	4.67E-05	-0.630	0.168	0.385	0.09336696	Preadipocytes_3
Entpd1.3	4.78E-05	-0.975	0.230	0.494	0.095626083	Preadipocytes_3
Card11.2	4.98E-05	-0.596	0.142	0.34	0.099547901	Preadipocytes_3
Adamts19	5.65E-05	0.759	0.283	0.126	0.112917988	Preadipocytes_3
Depdc1b	5.73E-05	0.512	0.257	0.153	0.114675586	Preadipocytes_3
Ank.1	5.89E-05	-0.623	0.230	0.506	0.117777854	Preadipocytes_3
Sema5a.2	7.02E-05	0.704	0.460	0.365	0.140420579	Preadipocytes_3
Cadps2.1	7.30E-05	0.507	0.442	0.337	0.146059757	Preadipocytes_3
Plpp1.1	8.15E-05	0.588	0.531	0.499	0.163081488	Preadipocytes_3
Adam23.1	8.33E-05	0.507	0.566	0.507	0.166602519	Preadipocytes_3
Mycbp2.2	8.39E-05	-1.073	0.788	0.853	0.167882565	Preadipocytes_3
Ddx60.3	9.38E-05	-0.612	0.398	0.661	0.187651188	Preadipocytes_3
Nabp1.3	0.0001004	-0.674	0.115	0.351	0.200786976	Preadipocytes_3
Hacd2.3	0.0001201	-0.798	0.354	0.289	0.240255054	Preadipocytes_3
Tox.2	0.0001233	-0.528	0.142	0.331	0.246503121	Preadipocytes_3
Itgb2.3	0.0001315	-0.524	0.035	0.25	0.263039322	Preadipocytes_3
Arid5b.3	0.0001327	-0.695	0.513	0.699	0.265307568	Preadipocytes_3
Pde3b.3	0.000138	-1.706	0.425	0.577	0.276045943	Preadipocytes_3
Cbfa2t3.2	0.0001561	-0.561	0.080	0.299	0.312248496	Preadipocytes_3
Sipa1l1.3	0.0001743	-0.836	0.363	0.574	0.348544246	Preadipocytes_3
Ctsl.3	0.0001879	-0.653	0.062	0.319	0.375700439	Preadipocytes_3
Pdpn.1	0.0001913	0.515	0.425	0.311	0.382686245	Preadipocytes_3
Trf.2	0.0002173	-0.595	0.018	0.275	0.434662735	Preadipocytes_3
Sort1.3	0.0002175	-0.650	0.150	0.365	0.434938533	Preadipocytes_3
Dlgap5	0.0002181	0.578	0.310	0.169	0.436147839	Preadipocytes_3
Kif22	0.0002313	0.629	0.274	0.175	0.462687735	Preadipocytes_3
Pfkfb3.3	0.0002353	-1.054	0.204	0.431	0.47057305	Preadipocytes_3
Ntrk2.1	0.0002602	-0.614	0.248	0.423	0.520349341	Preadipocytes_3
Rap1gap2.2	0.000279	-0.641	0.088	0.319	0.557913088	Preadipocytes_3
Fhit.1	0.0002878	-0.579	0.619	0.821	0.575574947	Preadipocytes_3
Tank.3	0.0002965	-0.650	0.469	0.645	0.592953971	Preadipocytes_3
Fgf13.3	0.000377	-0.810	0.248	0.427	0.753945181	Preadipocytes_3
Tenm4.3	0.0003884	-2.887	0.204	0.416	0.776787876	Preadipocytes_3
Ccdc18	0.0003901	0.777	0.310	0.225	0.780228889	Preadipocytes_3
Rnf149.2	0.0004217	-0.822	0.150	0.425	0.843331837	Preadipocytes_3
Pbk	0.0004634	0.614	0.274	0.129	0.926824549	Preadipocytes_3
Parpbp	0.0005019	0.573	0.257	0.152	1	Preadipocytes_3
Cd180.3	0.0005243	-0.745	0.115	0.324	1	Preadipocytes_3
Fmo2.3	0.0005299	-0.800	0.168	0.308	1	Preadipocytes_3
Ctsd.2	0.0005586	-1.024	0.080	0.307	1	Preadipocytes_3
Ppfia2.1	0.0005597	0.519	0.354	0.25	1	Preadipocytes_3
Atp6v1a.2	0.0006357	-0.717	0.257	0.499	1	Preadipocytes_3
Mctp2.2	0.0009675	-0.604	0.186	0.379	1	Preadipocytes_3
Hexa.3	0.0009998	-0.697	0.133	0.403	1	Preadipocytes_3
Pou2f2.1	0.0010068	-0.511	0.097	0.308	1	Preadipocytes_3
Pag1.3	0.0010388	-0.575	0.204	0.442	1	Preadipocytes_3
Txlng.1	0.0012806	-0.691	0.451	0.641	1	Preadipocytes_3
Bcar3.3	0.0016644	-1.698	0.239	0.425	1	Preadipocytes_3
Fgf10.2	0.0017668	-0.676	0.204	0.366	1	Preadipocytes_3
Ubash3b.3	0.0018617	-1.000	0.327	0.469	1	Preadipocytes_3
Cd86.1	0.0019097	-0.532	0.168	0.338	1	Preadipocytes_3
Trim25.3	0.0019343	-0.696	0.283	0.486	1	Preadipocytes_3

Tmem106a.3	0.002099	-0.775	0.142	0.405	1	Preadipocytes_3
Soat1.3	0.002182	-0.676	0.053	0.341	1	Preadipocytes_3
Fam126b.3	0.0023063	-0.647	0.389	0.342	1	Preadipocytes_3
Spag5	0.0026679	0.586	0.283	0.2	1	Preadipocytes_3
Esco2	0.0027772	0.518	0.257	0.141	1	Preadipocytes_3
Eepd1.3	0.0028733	-1.006	0.177	0.373	1	Preadipocytes_3
Clec12a.2	0.0035393	-0.528	0.097	0.279	1	Preadipocytes_3
Fnip2.3	0.0036568	-0.649	0.584	0.748	1	Preadipocytes_3
Inpp4b.2	0.0044225	-0.638	0.301	0.51	1	Preadipocytes_3
Ghr.3	0.0052352	-1.915	0.894	0.649	1	Preadipocytes_3
Ctsa.2	0.00693	-0.508	0.009	0.261	1	Preadipocytes_3
Lyve1.3	0.0087254	-0.607	0.133	0.266	1	Preadipocytes_3
Evl.3	0.0099765	-0.835	0.239	0.384	1	Preadipocytes_3
F13a1.3	0	3.649	0.935	0.437	0	Macrophages_1
Apoe.4	0	2.701	0.930	0.365	0	Macrophages_1
Cd163.4	0	2.577	0.753	0.335	0	Macrophages_1
Cfh.3	0	2.542	0.908	0.433	0	Macrophages_1
Ctsc.3	0	2.495	0.947	0.332	0	Macrophages_1
Retnla.4	0	2.327	0.746	0.135	0	Macrophages_1
Hdac9.3	0	1.996	0.935	0.412	0	Macrophages_1
Selenop.4	0	1.966	0.890	0.348	0	Macrophages_1
Plekhg5.3	0	1.964	0.850	0.348	0	Macrophages_1
Clec10a.4	0	1.928	0.900	0.295	0	Macrophages_1
Ednrb.4	0	1.899	0.777	0.277	0	Macrophages_1
C1qa.4	0	1.893	0.689	0.163	0	Macrophages_1
Fyb.4	0	1.797	0.955	0.384	0	Macrophages_1
Ccl6.4	0	1.781	0.795	0.181	0	Macrophages_1
Epsti1.4	0	1.777	0.910	0.339	0	Macrophages_1
Stab1.4	0	1.776	0.904	0.334	0	Macrophages_1
Adgre1.4	0	1.772	0.956	0.344	0	Macrophages_1
Pltp.3	0	1.771	0.845	0.307	0	Macrophages_1
P3h2.4	0	1.770	0.787	0.317	0	Macrophages_1
Bank1.4	0	1.736	0.756	0.263	0	Macrophages_1
Itga4.4	0	1.716	0.839	0.274	0	Macrophages_1
Mertk.3	0	1.715	0.938	0.452	0	Macrophages_1
Mtss1.3	0	1.680	0.963	0.493	0	Macrophages_1
Ccr2.4	0	1.676	0.658	0.129	0	Macrophages_1
Aoah.4	0	1.646	0.880	0.33	0	Macrophages_1
Lyz2.4	0	1.646	0.748	0.194	0	Macrophages_1
C1qb.4	0	1.611	0.706	0.164	0	Macrophages_1
Ms4a6c.4	0	1.581	0.839	0.242	0	Macrophages_1
Adgre4.4	0	1.564	0.660	0.168	0	Macrophages_1
Lyve1.4	0	1.497	0.767	0.096	0	Macrophages_1
Ddx60.4	0	1.423	0.886	0.583	0	Macrophages_1
Wdfy4.4	0	1.418	0.837	0.274	0	Macrophages_1
Shtn1.4	0	1.384	0.915	0.325	0	Macrophages_1
St8sia4.4	0	1.365	0.867	0.253	0	Macrophages_1
Prkcb.4	0	1.326	0.857	0.335	0	Macrophages_1
Ehd4.4	0	1.322	0.858	0.313	0	Macrophages_1
Cybb.4	0	1.305	0.790	0.204	0	Macrophages_1
Nrg2.3	0	1.292	0.782	0.301	0	Macrophages_1
Mctp2.3	0	1.280	0.739	0.256	0	Macrophages_1
Adap2.3	0	1.278	0.922	0.452	0	Macrophages_1
Ms4a6b.4	0	1.270	0.789	0.247	0	Macrophages_1
Kcnk13.4	0	1.250	0.799	0.363	0	Macrophages_1
Cfp.4	0	1.224	0.668	0.133	0	Macrophages_1
Csf2ra.4	0	1.221	0.833	0.268	0	Macrophages_1
Mafb.4	0	1.193	0.698	0.225	0	Macrophages_1
Ms4a4a.1	0	1.159	0.801	0.251	0	Macrophages_1
Kctd12.4	0	1.156	0.697	0.134	0	Macrophages_1
Spi1.4	0	1.156	0.815	0.246	0	Macrophages_1
Myo1f.4	0	1.141	0.934	0.375	0	Macrophages_1

Entpd1.4	0	1.116	0.849	0.371	0	Macrophages_1
Blnk.4	0	1.107	0.895	0.34	0	Macrophages_1
Lgmn.4	0	1.104	0.851	0.306	0	Macrophages_1
Abcg3.4	0	1.100	0.684	0.196	0	Macrophages_1
Ly6e.1	0	1.099	0.657	0.156	0	Macrophages_1
Itm2b.3	0	1.094	0.806	0.477	0	Macrophages_1
Gas6.4	0	1.070	0.822	0.329	0	Macrophages_1
Pla2g7.4	0	1.055	0.788	0.202	0	Macrophages_1
Tec.4	0	1.052	0.811	0.314	0	Macrophages_1
Nckap1l.4	0	1.043	0.823	0.299	0	Macrophages_1
Nrp2.4	0	1.031	0.839	0.299	0	Macrophages_1
Ncf1.3	0	1.028	0.669	0.167	0	Macrophages_1
Ms4a7.4	0	1.020	0.850	0.262	0	Macrophages_1
Pik3ap1.3	0	1.018	0.871	0.358	0	Macrophages_1
Cd300a.4	0	1.011	0.736	0.24	0	Macrophages_1
Pou2f2.2	0	0.998	0.644	0.193	0	Macrophages_1
Apobec1.3	0	0.994	0.924	0.41	0	Macrophages_1
Msr1.4	0	0.978	0.842	0.244	0	Macrophages_1
Cd86.2	0	0.957	0.665	0.227	0	Macrophages_1
Cd209f	0	0.956	0.616	0.082	0	Macrophages_1
Cd200r1.4	0	0.946	0.858	0.294	0	Macrophages_1
Abhd12.4	0	0.933	0.932	0.44	0	Macrophages_1
Plxna4.4	0	0.916	0.827	0.363	0	Macrophages_1
Lcp1.4	0	0.899	0.885	0.324	0	Macrophages_1
Ccr5.4	0	0.889	0.760	0.209	0	Macrophages_1
Pitpnc1.4	0	0.870	0.965	0.641	0	Macrophages_1
Plxnc1.4	0	0.867	0.766	0.215	0	Macrophages_1
Slc6a6.3	0	0.857	0.883	0.445	0	Macrophages_1
Mpp1.3	0	0.851	0.747	0.326	0	Macrophages_1
Grk3.3	0	0.846	0.699	0.244	0	Macrophages_1
Ubash3b.4	0	0.826	0.805	0.354	0	Macrophages_1
Cd84.4	0	0.787	0.900	0.324	0	Macrophages_1
Ptpn22.4	0	0.781	0.724	0.248	0	Macrophages_1
Cd209b	0	0.781	0.637	0.177	0	Macrophages_1
Cd38	0	0.779	0.731	0.251	0	Macrophages_1
C5ar1.4	0	0.777	0.757	0.192	0	Macrophages_1
Gcnt1.3	0	0.767	0.712	0.23	0	Macrophages_1
Hck.1	0	0.766	0.693	0.196	0	Macrophages_1
Insyn2b.4	0	0.760	0.833	0.289	0	Macrophages_1
Cacna1d.4	0	0.749	0.870	0.344	0	Macrophages_1
Tmem106a.4	0	0.747	0.748	0.287	0	Macrophages_1
Ripor2.2	0	0.744	0.734	0.288	0	Macrophages_1
Vsig4	0	0.732	0.671	0.205	0	Macrophages_1
Rnf149.3	0	0.724	0.754	0.311	0	Macrophages_1
Rnf180.1	0	0.722	0.805	0.471	0	Macrophages_1
Tnfrsf11a.4	0	0.721	0.795	0.339	0	Macrophages_1
Prune2.2	0	0.663	0.817	0.328	0	Macrophages_1
Pik3r5.4	0	0.661	0.747	0.284	0	Macrophages_1
Trim25.4	0	0.660	0.774	0.388	0	Macrophages_1
Ak8	0	0.638	0.613	0.152	0	Macrophages_1
Rbm47.4	0	0.636	0.939	0.471	0	Macrophages_1
Ipcef1.1	0	0.636	0.620	0.128	0	Macrophages_1
Ifi213	0	0.614	0.678	0.153	0	Macrophages_1
C6.1	0	0.589	0.744	0.181	0	Macrophages_1
Gm5150.2	0	0.535	0.666	0.188	0	Macrophages_1
Arhgap26.1	0	-0.521	0.072	0.43	0	Macrophages_1
Xkr6.1	0	-0.540	0.089	0.449	0	Macrophages_1
Clmn.1	0	-0.579	0.025	0.402	0	Macrophages_1
Ephb2.1	0	-0.586	0.027	0.435	0	Macrophages_1
Jph1.1	0	-0.594	0.008	0.429	0	Macrophages_1
Anpep.1	0	-0.649	0.282	0.466	0	Macrophages_1
Dusp10.2	0	-0.739	0.137	0.404	0	Macrophages_1

Nkain2.1	0	-0.773	0.001	0.419	0	Macrophages_1
Mme.3	0	-0.892	0.071	0.479	0	Macrophages_1
Fgf7.2	0	-0.918	0.108	0.463	0	Macrophages_1
Fmo1.1	0	-0.930	0.066	0.482	0	Macrophages_1
Trhde.2	0	-0.932	0.007	0.279	0	Macrophages_1
Sned1.1	0	-0.952	0.267	0.488	0	Macrophages_1
Apex2.1	0	-0.972	0.394	0.626	0	Macrophages_1
Foxp2.3	0	-0.987	0.003	0.386	0	Macrophages_1
Stox2.2	0	-1.034	0.171	0.55	0	Macrophages_1
Trabd2b.2	0	-1.048	0.074	0.443	0	Macrophages_1
Txlng.2	0	-1.068	0.454	0.701	0	Macrophages_1
Pde10a.2	0	-1.087	0.187	0.47	0	Macrophages_1
Asph.1	0	-1.093	0.573	0.825	0	Macrophages_1
Syne1.1	0	-1.097	0.521	0.763	0	Macrophages_1
Kcnc2.2	0	-1.099	0.012	0.395	0	Macrophages_1
Jazf1.3	0	-1.106	0.310	0.581	0	Macrophages_1
Prickle2.1	0	-1.111	0.241	0.537	0	Macrophages_1
Rgs7.3	0	-1.122	0.028	0.487	0	Macrophages_1
Ifrd1.1	0	-1.136	0.224	0.642	0	Macrophages_1
Boc.1	0	-1.158	0.227	0.56	0	Macrophages_1
Fign.2	0	-1.177	0.098	0.565	0	Macrophages_1
Ntrk2.2	0	-1.204	0.092	0.532	0	Macrophages_1
Ptprk.2	0	-1.210	0.020	0.557	0	Macrophages_1
Tcaf1.1	0	-1.245	0.046	0.575	0	Macrophages_1
Fam13a.2	0	-1.267	0.100	0.463	0	Macrophages_1
Ctdspl.1	0	-1.268	0.215	0.629	0	Macrophages_1
Gask1b.3	0	-1.289	0.303	0.577	0	Macrophages_1
Abca8b.3	0	-1.325	0.211	0.534	0	Macrophages_1
Anxa1.1	0	-1.346	0.325	0.674	0	Macrophages_1
Ldb2.3	0	-1.363	0.263	0.538	0	Macrophages_1
Dlg2.2	0	-1.376	0.151	0.565	0	Macrophages_1
Tln2.2	0	-1.381	0.087	0.588	0	Macrophages_1
Aebp1.3	0	-1.386	0.004	0.407	0	Macrophages_1
Csgalnact1.3	0	-1.406	0.421	0.604	0	Macrophages_1
Aox1.1	0	-1.410	0.074	0.603	0	Macrophages_1
Pmepa1.1	0	-1.419	0.018	0.556	0	Macrophages_1
Slc2a13.2	0	-1.425	0.100	0.605	0	Macrophages_1
Lgr4.1	0	-1.433	0.022	0.582	0	Macrophages_1
Aff2.2	0	-1.434	0.137	0.534	0	Macrophages_1
Col6a2.2	0	-1.451	0.056	0.524	0	Macrophages_1
Mical3.1	0	-1.451	0.067	0.605	0	Macrophages_1
Zfp423.1	0	-1.464	0.107	0.628	0	Macrophages_1
Ugdh.2	0	-1.479	0.194	0.552	0	Macrophages_1
Irs1.2	0	-1.482	0.109	0.581	0	Macrophages_1
Xkr4.2	0	-1.511	0.188	0.568	0	Macrophages_1
Pde1a.3	0	-1.531	0.091	0.627	0	Macrophages_1
Aspn.3	0	-1.532	0.023	0.535	0	Macrophages_1
Msrb3.2	0	-1.555	0.067	0.645	0	Macrophages_1
Prickle1.1	0	-1.597	0.374	0.608	0	Macrophages_1
Wwtr1.2	0	-1.654	0.134	0.665	0	Macrophages_1
Macrod2.1	0	-1.698	0.176	0.635	0	Macrophages_1
Foxo1.1	0	-1.702	0.393	0.754	0	Macrophages_1
Pcolce2.2	0	-1.708	0.203	0.571	0	Macrophages_1
Col4a2.3	0	-1.709	0.199	0.551	0	Macrophages_1
Postn.4	0	-1.715	0.143	0.518	0	Macrophages_1
Cachd1.2	0	-1.721	0.145	0.706	0	Macrophages_1
Bckdhb.3	0	-1.746	0.286	0.628	0	Macrophages_1
Ptprm.2	0	-1.768	0.687	0.815	0	Macrophages_1
Dmd.3	0	-1.769	0.168	0.672	0	Macrophages_1
Ror1.3	0	-1.790	0.494	0.649	0	Macrophages_1
Magi1.1	0	-1.791	0.259	0.647	0	Macrophages_1
Col6a3.2	0	-1.796	0.261	0.631	0	Macrophages_1

Pcdh19.4	0	-1.801	0.290	0.622	0	Macrophages_1
Acyp2.1	0	-1.808	0.346	0.776	0	Macrophages_1
Col5a3.3	0	-1.825	0.280	0.656	0	Macrophages_1
Angpt1.2	0	-1.841	0.199	0.633	0	Macrophages_1
Itih5.3	0	-1.858	0.093	0.693	0	Macrophages_1
Arhgap29.2	0	-1.887	0.150	0.689	0	Macrophages_1
Vcan.3	0	-1.888	0.431	0.599	0	Macrophages_1
Bmper.1	0	-1.898	0.435	0.656	0	Macrophages_1
Gabra3.3	0	-1.901	0.352	0.6	0	Macrophages_1
Errfi1.3	0	-1.912	0.391	0.579	0	Macrophages_1
Smpd3.2	0	-1.913	0.399	0.599	0	Macrophages_1
Dclk1.4	0	-1.939	0.425	0.663	0	Macrophages_1
Epn2.1	0	-1.946	0.140	0.687	0	Macrophages_1
Pdzd2.2	0	-1.946	0.234	0.631	0	Macrophages_1
Eda.2	0	-1.990	0.593	0.733	0	Macrophages_1
Large1.1	0	-2.033	0.636	0.922	0	Macrophages_1
Slc24a3.2	0	-2.105	0.369	0.671	0	Macrophages_1
Col4a1.3	0	-2.134	0.244	0.618	0	Macrophages_1
Prr16.2	0	-2.170	0.010	0.523	0	Macrophages_1
Clec3b.3	0	-2.198	0.246	0.589	0	Macrophages_1
Nrip1.3	0	-2.200	0.414	0.802	0	Macrophages_1
Vgll3.1	0	-2.209	0.251	0.714	0	Macrophages_1
B3galt1.1	0	-2.240	0.324	0.682	0	Macrophages_1
Tead1.3	0	-2.253	0.373	0.769	0	Macrophages_1
Pcsk5.2	0	-2.259	0.417	0.697	0	Macrophages_1
Adam12.4	0	-2.275	0.225	0.632	0	Macrophages_1
Mfap5.3	0	-2.279	0.233	0.622	0	Macrophages_1
Ccdc80.2	0	-2.316	0.058	0.674	0	Macrophages_1
Acss3.1	0	-2.338	0.171	0.749	0	Macrophages_1
Abca8a.3	0	-2.401	0.301	0.687	0	Macrophages_1
Slc1a5.3	0	-2.412	0.166	0.594	0	Macrophages_1
Sparc.3	0	-2.414	0.241	0.676	0	Macrophages_1
Ptprd.2	0	-2.455	0.174	0.732	0	Macrophages_1
Gfpt2.3	0	-2.524	0.315	0.65	0	Macrophages_1
Col1a1.3	0	-2.544	0.229	0.59	0	Macrophages_1
Auts2.3	0	-2.546	0.392	0.753	0	Macrophages_1
Ptprg.3	0	-2.600	0.295	0.812	0	Macrophages_1
Svep1.2	0	-2.652	0.284	0.744	0	Macrophages_1
Prkd1.3	0	-2.731	0.120	0.737	0	Macrophages_1
C7.2	0	-2.744	0.085	0.536	0	Macrophages_1
Efna5.4	0	-2.809	0.372	0.734	0	Macrophages_1
Prkg1.3	0	-2.822	0.636	0.749	0	Macrophages_1
Lama4.2	0	-2.834	0.162	0.813	0	Macrophages_1
Dst.2	0	-2.864	0.163	0.824	0	Macrophages_1
Itgbl1.2	0	-2.918	0.519	0.703	0	Macrophages_1
Thsd7a.3	0	-2.919	0.520	0.75	0	Macrophages_1
Col14a1.3	0	-2.923	0.519	0.72	0	Macrophages_1
Aldh1a1.3	0	-2.951	0.445	0.826	0	Macrophages_1
Adamts12.2	0	-2.952	0.518	0.809	0	Macrophages_1
Adamts5.2	0	-3.131	0.266	0.824	0	Macrophages_1
Adamtsl1.4	0	-3.153	0.638	0.772	0	Macrophages_1
Nox4.3	0	-3.345	0.503	0.764	0	Macrophages_1
Dcn.3	0	-3.353	0.313	0.723	0	Macrophages_1
Col1a2.4	0	-3.358	0.308	0.737	0	Macrophages_1
Mid1.3	0	-3.375	0.291	0.838	0	Macrophages_1
Sgcd.3	0	-3.408	0.168	0.758	0	Macrophages_1
Gsn.2	0	-3.928	0.504	0.77	0	Macrophages_1
Col3a1.4	0	-4.124	0.548	0.799	0	Macrophages_1
Ghr.4	0	-5.254	0.137	0.824	0	Macrophages_1
Pcolce.2	2.43E-305	-0.848	0.119	0.369	4.86E-302	Macrophages_1
Mgl2.3	5.01E-304	1.378	0.612	0.107	1.00E-300	Macrophages_1
Ctsb.4	7.88E-304	0.521	0.793	0.347	1.58E-300	Macrophages_1

Pcdh9.2	8.37E-304	-2.710	0.472	0.637	1.67E-300	Macrophages_1
Cd226.4	1.30E-303	1.264	0.616	0.128	2.60E-300	Macrophages_1
Galnt17.4	1.88E-303	-1.661	0.423	0.615	3.75E-300	Macrophages_1
Cacna1c.2	1.60E-302	-0.848	0.333	0.507	3.20E-299	Macrophages_1
Aqp9	5.43E-300	0.643	0.591	0.111	1.09E-296	Macrophages_1
Prkar2b.3	4.07E-299	-2.352	0.255	0.288	8.14E-296	Macrophages_1
Pi16.2	5.49E-297	-2.794	0.299	0.591	1.10E-293	Macrophages_1
Pdzrn3.3	1.08E-296	-1.962	0.154	0.543	2.16E-293	Macrophages_1
Dnm3.2	3.16E-293	-1.281	0.560	0.66	6.32E-290	Macrophages_1
Mgll.2	5.51E-293	-1.860	0.124	0.557	1.10E-289	Macrophages_1
Atp6v0d2.4	6.67E-289	-0.645	0.775	0.307	1.33E-285	Macrophages_1
Ctss.4	2.58E-286	0.715	0.707	0.23	5.16E-283	Macrophages_1
Chrdl1.1	1.44E-285	-1.012	0.169	0.481	2.88E-282	Macrophages_1
Sema3e.3	3.56E-285	-2.340	0.359	0.595	7.11E-282	Macrophages_1
Rnf24.1	6.83E-284	-0.749	0.120	0.385	1.37E-280	Macrophages_1
Tns3.3	1.03E-283	0.729	0.794	0.39	2.05E-280	Macrophages_1
Ahcyl2.4	1.62E-282	0.917	0.898	0.543	3.25E-279	Macrophages_1
Slc7a8.3	5.58E-282	0.535	0.727	0.305	1.12E-278	Macrophages_1
Dok2.3	1.43E-280	0.811	0.631	0.162	2.87E-277	Macrophages_1
Skap1.2	1.46E-280	-0.597	0.505	0.166	2.92E-277	Macrophages_1
Fgr.2	9.17E-279	0.921	0.572	0.117	1.83E-275	Macrophages_1
Fbln2.4	3.99E-278	-1.768	0.225	0.531	7.98E-275	Macrophages_1
Ccdc3.1	1.86E-276	-0.877	0.028	0.441	3.72E-273	Macrophages_1
Col12a1.3	3.63E-272	-1.403	0.131	0.468	7.26E-269	Macrophages_1
Col15a1.3	3.90E-270	-1.209	0.153	0.475	7.79E-267	Macrophages_1
P2ry12.1	3.03E-269	1.001	0.667	0.284	6.06E-266	Macrophages_1
Tsc22d3.4	1.22E-268	0.520	0.804	0.429	2.44E-265	Macrophages_1
Ptpre.3	2.40E-268	0.971	0.681	0.268	4.80E-265	Macrophages_1
Mpp7.4	9.92E-268	-2.026	0.133	0.36	1.98E-264	Macrophages_1
Ucp2.1	1.48E-266	0.780	0.601	0.127	2.96E-263	Macrophages_1
Eln.3	2.25E-264	-2.213	0.308	0.572	4.50E-261	Macrophages_1
Fam126b.4	9.34E-263	-1.320	0.223	0.382	1.87E-259	Macrophages_1
Ptpn14.1	1.49E-261	-0.564	0.010	0.422	2.99E-258	Macrophages_1
Negr1.3	7.94E-260	-1.354	0.657	0.791	1.59E-256	Macrophages_1
Ctnna3.2	1.28E-259	-0.804	0.027	0.336	2.56E-256	Macrophages_1
Fcrls.2	2.01E-259	1.002	0.589	0.106	4.01E-256	Macrophages_1
Emb.3	6.49E-259	0.861	0.676	0.351	1.30E-255	Macrophages_1
Myo1e.2	2.05E-258	-1.616	0.568	0.71	4.11E-255	Macrophages_1
Robo1.3	4.92E-252	-2.061	0.449	0.605	9.84E-249	Macrophages_1
Cacna1e.2	1.26E-247	-0.711	0.033	0.25	2.51E-244	Macrophages_1
Slit2.1	2.84E-247	-1.022	0.111	0.422	5.69E-244	Macrophages_1
Sdc2.4	1.16E-245	-1.836	0.473	0.619	2.33E-242	Macrophages_1
P2rx7.3	7.42E-244	0.723	0.709	0.32	1.48E-240	Macrophages_1
Igfbp5.4	1.66E-240	-2.273	0.126	0.494	3.31E-237	Macrophages_1
Igf1.3	3.09E-237	0.692	0.902	0.506	6.19E-234	Macrophages_1
Fcna	6.92E-237	1.007	0.545	0.067	1.38E-233	Macrophages_1
St6galnac5.1	1.68E-236	-0.623	0.119	0.352	3.36E-233	Macrophages_1
Ntrk3.1	2.42E-236	-0.743	0.032	0.427	4.84E-233	Macrophages_1
Plxna2.1	3.57E-236	-1.047	0.386	0.584	7.13E-233	Macrophages_1
Snhg11.2	5.34E-235	-0.902	0.008	0.409	1.07E-231	Macrophages_1
Folr2	7.55E-235	0.837	0.561	0.082	1.51E-231	Macrophages_1
Prex2.1	1.03E-234	-1.352	0.313	0.559	2.07E-231	Macrophages_1
Dtna.1	2.63E-233	-0.688	0.226	0.396	5.26E-230	Macrophages_1
C9orf72.3	2.18E-232	-0.783	0.207	0.332	4.36E-229	Macrophages_1
Sirpa.4	2.11E-231	0.706	0.737	0.411	4.23E-228	Macrophages_1
Plpp1.2	1.90E-230	-0.766	0.416	0.527	3.79E-227	Macrophages_1
Ciita.4	2.97E-229	1.351	0.611	0.196	5.95E-226	Macrophages_1
Igfbp7.3	6.13E-228	-1.380	0.087	0.464	1.23E-224	Macrophages_1
Ano3.3	1.65E-226	-1.724	0.278	0.524	3.29E-223	Macrophages_1
Rad18.2	1.88E-226	-0.695	0.384	0.502	3.76E-223	Macrophages_1
Clec4a2.2	2.90E-226	0.619	0.616	0.158	5.81E-223	Macrophages_1
Nnat.2	5.55E-223	-1.629	0.010	0.28	1.11E-219	Macrophages_1

Adamts16.2	5.81E-223	-1.246	0.191	0.422	1.16E-219	Macrophages_1
Ttyh2.1	3.15E-222	-0.599	0.330	0.488	6.30E-219	Macrophages_1
C3ar1.2	6.64E-222	0.536	0.632	0.186	1.33E-218	Macrophages_1
Myo1d.3	2.91E-220	-0.664	0.221	0.451	5.82E-217	Macrophages_1
Rasa1.4	3.55E-217	0.552	0.933	0.637	7.10E-214	Macrophages_1
Fgf10.3	6.24E-217	-1.363	0.079	0.461	1.25E-213	Macrophages_1
Por.2	2.70E-215	-0.985	0.263	0.436	5.40E-212	Macrophages_1
Timp3.4	4.04E-214	-1.458	0.250	0.51	8.07E-211	Macrophages_1
Sult1e1.2	4.33E-214	-1.067	0.055	0.381	8.66E-211	Macrophages_1
Cbfa2t3.3	3.53E-213	1.014	0.603	0.194	7.07E-210	Macrophages_1
Sntb1.2	4.61E-212	-1.806	0.076	0.271	9.22E-209	Macrophages_1
Gm4951	1.03E-211	0.675	0.804	0.464	2.06E-208	Macrophages_1
Eya4.2	2.69E-211	-1.532	0.504	0.601	5.39E-208	Macrophages_1
Gria3.2	2.14E-210	-1.450	0.515	0.576	4.29E-207	Macrophages_1
Tshr.2	2.15E-210	-1.955	0.009	0.264	4.31E-207	Macrophages_1
Sema3d.2	7.04E-210	-1.168	0.116	0.432	1.41E-206	Macrophages_1
Mmp3.2	8.94E-210	-1.012	0.068	0.37	1.79E-206	Macrophages_1
Samsn1.2	1.23E-206	0.535	0.580	0.134	2.47E-203	Macrophages_1
Cotl1.4	3.72E-204	0.686	0.635	0.217	7.45E-201	Macrophages_1
Lvrn.2	1.42E-203	-1.834	0.443	0.58	2.85E-200	Macrophages_1
Gpc4.1	2.82E-203	-0.784	0.070	0.42	5.65E-200	Macrophages_1
Nrg4.2	2.74E-202	-1.392	0.010	0.26	5.48E-199	Macrophages_1
Ell2.3	5.38E-202	-0.870	0.470	0.615	1.08E-198	Macrophages_1
Col4a5.3	7.08E-200	-1.036	0.365	0.52	1.42E-196	Macrophages_1
Fcer1g.3	4.31E-199	0.723	0.581	0.15	8.62E-196	Macrophages_1
Grn.4	6.61E-199	0.689	0.659	0.274	1.32E-195	Macrophages_1
Col8a1.4	1.87E-198	-1.165	0.172	0.43	3.74E-195	Macrophages_1
Grb14.2	4.51E-198	-1.111	0.092	0.387	9.02E-195	Macrophages_1
Epha3.3	1.02E-197	-1.541	0.251	0.491	2.04E-194	Macrophages_1
Aldh1a3.3	3.57E-196	-0.920	0.076	0.406	7.14E-193	Macrophages_1
Rel.2	4.79E-196	0.550	0.605	0.223	9.58E-193	Macrophages_1
Col6a1.3	8.83E-196	-1.093	0.002	0.395	1.77E-192	Macrophages_1
Fmr1.3	3.37E-195	-1.121	0.284	0.381	6.73E-192	Macrophages_1
Clic4.1	2.60E-194	-0.747	0.450	0.595	5.21E-191	Macrophages_1
Cdh9.2	4.23E-194	-1.180	0.322	0.513	8.46E-191	Macrophages_1
Pkp2.1	6.72E-194	-0.611	0.009	0.393	1.34E-190	Macrophages_1
Ngf.3	1.00E-193	-0.702	0.064	0.392	2.00E-190	Macrophages_1
Sox6.2	1.95E-192	-0.879	0.025	0.278	3.90E-189	Macrophages_1
Npr3.2	4.76E-191	-2.609	0.010	0.264	9.51E-188	Macrophages_1
Hmcn2.2	1.67E-188	-1.443	0.246	0.462	3.34E-185	Macrophages_1
H2-DMb1.1	2.85E-188	0.730	0.561	0.118	5.69E-185	Macrophages_1
Acox1.3	2.45E-184	-1.181	0.281	0.412	4.91E-181	Macrophages_1
Cst3.3	7.07E-183	0.850	0.597	0.223	1.41E-179	Macrophages_1
C1qtnf7.3	1.16E-182	-1.546	0.339	0.515	2.33E-179	Macrophages_1
Esrrg.1	4.44E-178	-0.550	0.008	0.276	8.88E-175	Macrophages_1
Nrcam.1	7.76E-177	-0.715	0.201	0.418	1.55E-173	Macrophages_1
Galnt18.1	3.14E-176	-0.634	0.093	0.403	6.27E-173	Macrophages_1
Tmem45b.2	5.72E-176	-1.822	0.010	0.273	1.14E-172	Macrophages_1
Rasgrp3.1	4.60E-174	0.609	0.614	0.267	9.21E-171	Macrophages_1
Dram1.3	3.34E-173	-0.978	0.097	0.307	6.69E-170	Macrophages_1
Prdm16.1	5.96E-173	-0.740	0.077	0.435	1.19E-169	Macrophages_1
Atp8b4.4	8.24E-169	0.905	0.660	0.292	1.65E-165	Macrophages_1
Serpinf1.3	2.23E-167	-1.300	0.073	0.391	4.47E-164	Macrophages_1
Il1r2.2	1.32E-166	-0.654	0.051	0.334	2.63E-163	Macrophages_1
C1qc.2	1.80E-165	1.398	0.566	0.136	3.60E-162	Macrophages_1
Igfbp6.3	9.45E-165	-1.384	0.002	0.375	1.89E-161	Macrophages_1
Car8.3	1.23E-162	-1.073	0.145	0.425	2.46E-159	Macrophages_1
Tmem135.3	1.65E-161	-1.490	0.521	0.63	3.31E-158	Macrophages_1
Mamdc2.4	4.51E-158	1.473	0.637	0.32	9.02E-155	Macrophages_1
Gabre.3	5.58E-157	-0.723	0.186	0.409	1.12E-153	Macrophages_1
C3.3	1.86E-155	-1.400	0.087	0.363	3.72E-152	Macrophages_1
Bcl2.2	4.25E-155	-0.685	0.554	0.649	8.49E-152	Macrophages_1

Cd300lb.4	5.25E-152	0.534	0.651	0.258	1.05E-148	Macrophages_1
Vegfc.1	5.48E-152	-0.702	0.210	0.416	1.10E-148	Macrophages_1
Serpinh1.2	1.82E-151	-0.857	0.000	0.349	3.63E-148	Macrophages_1
Sntg1.3	8.30E-151	-2.348	0.512	0.589	1.66E-147	Macrophages_1
Optn.3	4.86E-150	-0.814	0.096	0.339	9.73E-147	Macrophages_1
Myl1.2	1.02E-149	-1.089	0.037	0.254	2.04E-146	Macrophages_1
Batf3.1	2.34E-149	0.840	0.547	0.134	4.68E-146	Macrophages_1
Ppip5k1.1	3.67E-149	-0.609	0.462	0.546	7.34E-146	Macrophages_1
Tnik.1	1.93E-148	-0.721	0.293	0.439	3.87E-145	Macrophages_1
Adh1.1	4.97E-147	-0.535	0.036	0.35	9.94E-144	Macrophages_1
Hacd2.4	3.15E-146	-1.733	0.223	0.312	6.29E-143	Macrophages_1
Csmd1.2	2.47E-145	-1.403	0.080	0.368	4.94E-142	Macrophages_1
Gpc3.3	7.68E-145	-1.217	0.291	0.47	1.54E-141	Macrophages_1
Pdpn.2	1.46E-144	-0.644	0.088	0.387	2.93E-141	Macrophages_1
Adam23.2	9.68E-144	-1.116	0.413	0.539	1.94E-140	Macrophages_1
2810004N23Ri	1.12E-143	-0.577	0.249	0.326	2.25E-140	Macrophages_1
Cat.3	1.68E-143	-0.792	0.237	0.276	3.35E-140	Macrophages_1
Trf.3	6.62E-143	0.933	0.570	0.172	1.32E-139	Macrophages_1
Slc1a3.2	6.74E-143	-1.097	0.065	0.33	1.35E-139	Macrophages_1
Ly6a.3	4.14E-142	-1.296	0.165	0.393	8.28E-139	Macrophages_1
Tmem51	2.33E-141	0.595	0.517	0.108	4.66E-138	Macrophages_1
Skint3.1	5.38E-140	1.031	0.511	0.078	1.08E-136	Macrophages_1
Slc11a1.2	1.80E-138	0.671	0.579	0.173	3.60E-135	Macrophages_1
Shroom3.1	2.65E-138	-0.722	0.098	0.367	5.31E-135	Macrophages_1
Lpgat1.3	3.15E-138	-2.050	0.269	0.371	6.30E-135	Macrophages_1
Klf8.1	5.13E-138	0.560	0.640	0.29	1.03E-134	Macrophages_1
Ccbe1.3	5.32E-137	-0.957	0.142	0.382	1.06E-133	Macrophages_1
Cobll1.2	1.20E-136	-1.011	0.381	0.455	2.39E-133	Macrophages_1
Enpp2.3	2.43E-136	-1.611	0.083	0.427	4.85E-133	Macrophages_1
Ank.2	1.03E-134	0.608	0.698	0.439	2.06E-131	Macrophages_1
Chpt1.3	7.56E-133	-1.507	0.097	0.279	1.51E-129	Macrophages_1
Tbc1d4.3	3.31E-130	0.803	0.920	0.709	6.63E-127	Macrophages_1
Ccl9.3	6.63E-126	0.592	0.563	0.167	1.33E-122	Macrophages_1
Chn2.3	1.76E-125	1.161	0.693	0.486	3.53E-122	Macrophages_1
Pde7b.4	7.66E-123	0.898	0.911	0.745	1.53E-119	Macrophages_1
Vcl.3	9.81E-123	-0.647	0.487	0.601	1.96E-119	Macrophages_1
Ntn4.3	1.15E-122	-1.254	0.255	0.451	2.31E-119	Macrophages_1
Bmp6.3	1.84E-122	-1.230	0.358	0.482	3.67E-119	Macrophages_1
Smoc2.2	3.30E-122	-1.134	0.300	0.464	6.60E-119	Macrophages_1
Cd44.4	3.60E-122	0.658	0.683	0.432	7.20E-119	Macrophages_1
Pcdh11x.1	5.32E-122	-0.589	0.001	0.262	1.06E-118	Macrophages_1
Fam214a.2	4.76E-121	-0.707	0.684	0.732	9.51E-118	Macrophages_1
Fat3.4	7.59E-121	-0.704	0.609	0.285	1.52E-117	Macrophages_1
Cadm1.4	3.62E-120	-0.565	0.639	0.323	7.23E-117	Macrophages_1
Pde11a.3	7.03E-120	-1.365	0.288	0.461	1.41E-116	Macrophages_1
Ltc4s.1	1.25E-119	0.763	0.552	0.163	2.50E-116	Macrophages_1
Sfrp4.3	1.71E-117	-1.009	0.113	0.344	3.43E-114	Macrophages_1
Tmem120b.2	3.03E-117	-0.606	0.224	0.295	6.07E-114	Macrophages_1
Prss23.3	5.67E-117	-0.922	0.001	0.319	1.13E-113	Macrophages_1
Gpr39.1	2.27E-115	-0.538	0.145	0.354	4.54E-112	Macrophages_1
Ranbp17.2	1.44E-114	-0.652	0.028	0.388	2.89E-111	Macrophages_1
Enah.2	1.97E-114	-0.694	0.109	0.383	3.95E-111	Macrophages_1
Basp1.3	1.29E-113	-0.818	0.423	0.511	2.59E-110	Macrophages_1
Rap1gap2.3	5.29E-113	0.927	0.569	0.232	1.06E-109	Macrophages_1
Dok6.1	7.86E-112	-0.509	0.329	0.452	1.57E-108	Macrophages_1
Tspan18.1	1.50E-111	-0.798	0.074	0.415	3.01E-108	Macrophages_1
Gucy1a2.2	4.88E-110	-0.924	0.285	0.433	9.76E-107	Macrophages_1
Fmo2.4	6.23E-110	-1.344	0.123	0.368	1.25E-106	Macrophages_1
Fzd4.1	4.14E-106	-0.561	0.029	0.293	8.28E-103	Macrophages_1
Ccl8	1.53E-104	0.666	0.374	0.051	3.07E-101	Macrophages_1
Nhs.1	4.07E-104	-0.659	0.047	0.341	8.14E-101	Macrophages_1
March3.1	1.01E-102	-0.683	0.867	0.83	2.03E-99	Macrophages_1

Cd248.3	1.12E-102	-0.985	0.002	0.326	2.24E-99	Macrophages_1
Cd209g	2.25E-102	0.884	0.498	0.112	4.50E-99	Macrophages_1
Sik2.3	3.07E-101	-1.485	0.594	0.637	6.15E-98	Macrophages_1
Fth1	5.09E-101	0.582	0.520	0.237	1.02E-97	Macrophages_1
Rgs6.2	1.88E-99	-1.795	0.494	0.53	3.75E-96	Macrophages_1
Peg3.2	4.73E-98	-0.839	0.007	0.339	9.46E-95	Macrophages_1
Meox2.2	1.08E-97	-1.318	0.337	0.477	2.16E-94	Macrophages_1
Il1rl1.1	8.75E-97	0.587	0.532	0.152	1.75E-93	Macrophages_1
Lhfpl2.3	1.73E-96	-1.506	0.329	0.504	3.47E-93	Macrophages_1
Bcl11a	5.03E-95	0.726	0.450	0.091	1.01E-91	Macrophages_1
Clec4b1.2	8.49E-95	0.627	0.546	0.166	1.70E-91	Macrophages_1
Map1b.3	1.67E-93	-0.974	0.150	0.385	3.34E-90	Macrophages_1
Mycbp2.3	2.51E-93	-1.202	0.842	0.857	5.02E-90	Macrophages_1
Sema5a.3	3.60E-93	-0.828	0.209	0.418	7.19E-90	Macrophages_1
Irf8.1	1.05E-91	0.530	0.562	0.177	2.10E-88	Macrophages_1
Anxa2.3	1.60E-91	-0.795	0.482	0.548	3.20E-88	Macrophages_1
Nrep.2	3.67E-91	-0.801	0.183	0.405	7.35E-88	Macrophages_1
Nedd4l.2	5.15E-91	-0.962	0.663	0.701	1.03E-87	Macrophages_1
Rgs18.1	8.78E-90	0.575	0.550	0.173	1.76E-86	Macrophages_1
Sgip1.3	9.49E-90	-1.059	0.475	0.498	1.90E-86	Macrophages_1
Rorb.3	6.23E-89	-1.701	0.475	0.527	1.25E-85	Macrophages_1
Wnt5b.1	4.08E-88	-0.565	0.063	0.317	8.15E-85	Macrophages_1
Gpm6b.2	3.30E-87	-0.992	0.210	0.404	6.61E-84	Macrophages_1
Slc25a21.3	3.03E-84	-0.747	0.102	0.332	6.07E-81	Macrophages_1
Ndnf.1	1.36E-83	0.718	0.463	0.111	2.73E-80	Macrophages_1
Cd209d	3.79E-83	0.953	0.492	0.055	7.59E-80	Macrophages_1
Gas1.1	1.53E-81	-0.733	0.000	0.266	3.06E-78	Macrophages_1
Vit.1	2.24E-81	-0.609	0.049	0.295	4.47E-78	Macrophages_1
Tenm4.4	8.42E-80	-2.557	0.457	0.4	1.68E-76	Macrophages_1
Rgs7bp.1	1.26E-79	-0.512	0.349	0.418	2.53E-76	Macrophages_1
Atp1a1.3	1.95E-79	0.541	0.536	0.215	3.89E-76	Macrophages_1
Cdh13.2	7.35E-79	-1.505	0.454	0.51	1.47E-75	Macrophages_1
Ism1.1	8.81E-78	-0.705	0.188	0.348	1.76E-74	Macrophages_1
Adcy5.3	2.78E-77	-0.812	0.010	0.336	5.55E-74	Macrophages_1
Zfp385b.2	8.89E-73	-0.535	0.192	0.382	1.78E-69	Macrophages_1
Cadps2.2	4.27E-72	-0.695	0.230	0.375	8.53E-69	Macrophages_1
Vegfa.1	5.18E-71	-0.777	0.009	0.366	1.04E-67	Macrophages_1
Nipal2.2	4.40E-69	-0.884	0.115	0.36	8.81E-66	Macrophages_1
Nav2.3	7.86E-67	-0.534	0.634	0.363	1.57E-63	Macrophages_1
Plcxd3.3	3.08E-65	-1.450	0.235	0.408	6.16E-62	Macrophages_1
Ralgapa2.3	4.23E-65	-1.134	0.624	0.611	8.47E-62	Macrophages_1
Cbr2	7.22E-65	0.742	0.478	0.075	1.44E-61	Macrophages_1
Nr3c2.2	1.66E-64	-0.792	0.741	0.671	3.32E-61	Macrophages_1
Fhit.2	2.06E-63	-0.647	0.855	0.808	4.11E-60	Macrophages_1
Eepd1.4	4.86E-63	-0.948	0.436	0.35	9.72E-60	Macrophages_1
Tmeff1.2	5.45E-63	-0.581	0.008	0.315	1.09E-59	Macrophages_1
Gria4.3	1.28E-62	-1.382	0.307	0.404	2.56E-59	Macrophages_1
Pparg.3	1.04E-60	-1.613	0.583	0.596	2.09E-57	Macrophages_1
Fry.4	1.98E-59	-0.559	0.714	0.428	3.96E-56	Macrophages_1
Dpyd.1	4.35E-58	-0.823	0.621	0.596	8.70E-55	Macrophages_1
Mmp12.2	2.13E-57	-0.757	0.377	0.157	4.26E-54	Macrophages_1
Thsd4.3	3.11E-56	1.092	0.469	0.22	6.22E-53	Macrophages_1
Sema3a.2	4.00E-56	-0.770	0.271	0.388	7.99E-53	Macrophages_1
Bcar3.4	6.55E-56	-1.360	0.507	0.395	1.31E-52	Macrophages_1
Ccn2.2	7.91E-56	-0.653	0.000	0.251	1.58E-52	Macrophages_1
Adra1a.2	2.79E-55	-0.509	0.230	0.321	5.57E-52	Macrophages_1
Celf4.2	1.50E-54	0.732	0.542	0.261	3.00E-51	Macrophages_1
Synpo2.1	1.33E-53	-0.698	0.074	0.324	2.66E-50	Macrophages_1
Chrm3.1	7.79E-53	-0.566	0.043	0.27	1.56E-49	Macrophages_1
Chst11.1	2.38E-52	-0.895	0.590	0.551	4.76E-49	Macrophages_1
Abtb2.3	1.33E-51	-0.608	0.354	0.409	2.66E-48	Macrophages_1
Bgn.3	1.84E-51	-1.084	0.055	0.289	3.67E-48	Macrophages_1

Cd9.3	1.17E-48	-0.535	0.248	0.331	2.33E-45	Macrophages_1
Arap2.3	1.34E-47	-0.566	0.245	0.257	2.68E-44	Macrophages_1
Ppp2r2b.2	4.15E-47	-1.495	0.376	0.452	8.29E-44	Macrophages_1
Thrb.2	4.05E-46	-0.578	0.829	0.798	8.11E-43	Macrophages_1
Pilra	1.64E-45	0.526	0.450	0.082	3.28E-42	Macrophages_1
Nr1h4.2	3.88E-44	-1.196	0.481	0.474	7.76E-41	Macrophages_1
Cd36.4	4.57E-43	-1.085	0.852	0.495	9.14E-40	Macrophages_1
Ywhab.3	5.09E-43	-0.596	0.372	0.354	1.02E-39	Macrophages_1
Apod.3	1.80E-42	-0.788	0.127	0.317	3.61E-39	Macrophages_1
Nlgn1.2	5.43E-40	-0.864	0.180	0.312	1.09E-36	Macrophages_1
Fabp4.3	9.11E-40	-2.064	0.438	0.485	1.82E-36	Macrophages_1
Zranb3.2	1.40E-39	-0.666	0.447	0.286	2.80E-36	Macrophages_1
Me1.3	1.14E-38	-1.324	0.011	0.329	2.28E-35	Macrophages_1
Ccser1.1	2.30E-38	-0.794	0.490	0.51	4.59E-35	Macrophages_1
Gnai1.2	9.41E-36	-1.006	0.008	0.256	1.88E-32	Macrophages_1
Coro2a.2	1.41E-33	0.510	0.524	0.239	2.83E-30	Macrophages_1
Kcnq1.1	6.07E-31	0.609	0.648	0.523	1.21E-27	Macrophages_1
Npas2.2	6.99E-31	-0.605	0.307	0.378	1.40E-27	Macrophages_1
Prelp.1	3.89E-30	-0.637	0.000	0.276	7.77E-27	Macrophages_1
Cav1.2	8.13E-28	-1.658	0.009	0.275	1.63E-24	Macrophages_1
Ppfia2.2	1.20E-27	-0.614	0.117	0.295	2.40E-24	Macrophages_1
Sorbs1.3	1.41E-26	-3.233	0.105	0.365	2.83E-23	Macrophages_1
Cpeb4.4	2.42E-26	-0.516	0.820	0.512	4.84E-23	Macrophages_1
Dkk2.3	3.61E-26	-1.004	0.307	0.378	7.22E-23	Macrophages_1
Mpeg1.3	3.88E-26	0.538	0.455	0.152	7.76E-23	Macrophages_1
Adamtsl3.2	1.44E-25	-1.087	0.411	0.45	2.87E-22	Macrophages_1
Clybl.3	2.70E-25	-0.505	0.652	0.61	5.41E-22	Macrophages_1
Lsamp.2	1.29E-24	-0.695	0.210	0.318	2.59E-21	Macrophages_1
F3.2	7.26E-23	-0.864	0.070	0.292	1.45E-19	Macrophages_1
Serpine2.1	1.74E-22	-0.636	0.051	0.289	3.48E-19	Macrophages_1
Tspan12.2	4.80E-22	-1.335	0.009	0.312	9.60E-19	Macrophages_1
Lpl.4	1.06E-20	-1.114	0.598	0.566	2.11E-17	Macrophages_1
Eya2.1	4.14E-17	-0.850	0.404	0.433	8.28E-14	Macrophages_1
Ctsl.4	5.88E-17	-0.722	0.454	0.271	1.18E-13	Macrophages_1
Pacrg.3	3.78E-15	-0.928	0.508	0.402	7.55E-12	Macrophages_1
Cenpp.1	3.19E-14	-0.620	0.601	0.483	6.38E-11	Macrophages_1
BC035044	1.16E-13	0.545	0.432	0.097	2.32E-10	Macrophages_1
Adamts9.1	1.71E-12	-0.511	0.069	0.286	3.42E-09	Macrophages_1
Plbd1.1	2.54E-12	0.633	0.447	0.15	5.07E-09	Macrophages_1
Adipor2.3	3.89E-11	-1.078	0.470	0.351	7.78E-08	Macrophages_1
Cytip.1	2.04E-09	0.614	0.428	0.113	4.08E-06	Macrophages_1
Edn1.1	3.91E-09	-0.520	0.153	0.291	7.82E-06	Macrophages_1
Igf2bp2	1.08E-08	-0.505	0.249	0.279	2.16E-05	Macrophages_1
Calcrl.3	9.65E-08	-0.795	0.734	0.619	0.000193	Macrophages_1
Cavin2.1	6.49E-06	-0.757	0.006	0.263	0.01297493	Macrophages_1
Mecom.1	9.52E-06	-0.651	0.458	0.424	0.019046823	Macrophages_1
Ikzf2	5.84E-05	-0.564	0.695	0.533	0.116712952	Macrophages_1
Kcnq5.2	9.14E-05	-1.223	0.561	0.454	0.182809632	Macrophages_1
Dact2.3	0.0001399	-0.680	0.187	0.275	0.279734401	Macrophages_1
Fgf13.4	0.0004466	-1.245	0.545	0.385	0.893177058	Macrophages_1
Tox.3	0.000837	-0.847	0.335	0.328	1	Macrophages_1
Pde7a.2	0.0030498	-0.557	0.805	0.672	1	Macrophages_1
Pf4	0.0033176	0.534	0.381	0.062	1	Macrophages_1
Art3.1	0.0065373	-1.145	0.009	0.251	1	Macrophages_1
Osbpl3.1	0.008816	-0.704	0.732	0.487	1	Macrophages_1
Lgals3.4	0	3.177	0.927	0.21	0	Macrophages_2
Gpnmb.4	0	3.110	0.841	0.321	0	Macrophages_2
Psap.4	0	2.985	0.981	0.445	0	Macrophages_2
Atp6v0d2.5	0	2.909	0.905	0.342	0	Macrophages_2
Cadm1.5	0	2.860	0.868	0.323	0	Macrophages_2
St18.4	0	2.716	0.905	0.36	0	Macrophages_2
Myo1e.3	0	2.523	0.947	0.627	0	Macrophages_2

Fat3.5	0	2.444	0.768	0.297	0	Macrophages_2
Ctsd.3	0	2.382	0.836	0.213	0	Macrophages_2
Ctsb.5	0	2.317	0.959	0.373	0	Macrophages_2
Mmp12.3	0	2.271	0.641	0.139	0	Macrophages_2
Lipa.2	0	2.226	0.904	0.203	0	Macrophages_2
Alcam.3	0	2.181	0.989	0.448	0	Macrophages_2
Arhgap25.4	0	2.161	0.878	0.313	0	Macrophages_2
Plin2.3	0	2.116	0.953	0.411	0	Macrophages_2
Evl.4	0	2.093	0.910	0.292	0	Macrophages_2
Ctsl.5	0	2.081	0.730	0.246	0	Macrophages_2
Apobec1.4	0	2.031	0.982	0.463	0	Macrophages_2
Lpl.5	0	2.005	0.940	0.511	0	Macrophages_2
Nceh1.2	0	1.985	0.902	0.276	0	Macrophages_2
Cd36.5	0	1.978	0.977	0.518	0	Macrophages_2
Cd84.5	0	1.917	0.953	0.385	0	Macrophages_2
Osbpl8.3	0	1.899	0.980	0.704	0	Macrophages_2
Cd44.5	0	1.860	0.944	0.417	0	Macrophages_2
Atp6v0a1.4	0	1.853	0.963	0.602	0	Macrophages_2
Fnip2.4	0	1.839	0.894	0.722	0	Macrophages_2
Myo1f.5	0	1.828	0.978	0.436	0	Macrophages_2
Specc1.4	0	1.824	0.934	0.45	0	Macrophages_2
Zranb3.3	0	1.809	0.736	0.256	0	Macrophages_2
Lcp1.5	0	1.770	0.963	0.379	0	Macrophages_2
Gas2l3.3	0	1.724	0.877	0.319	0	Macrophages_2
Ctss.5	0	1.680	0.872	0.26	0	Macrophages_2
Pitpnc1.5	0	1.659	0.972	0.68	0	Macrophages_2
Rbm47.5	0	1.639	0.968	0.524	0	Macrophages_2
Atp6v1b2.3	0	1.622	0.912	0.49	0	Macrophages_2
Fam129b.1	0	1.611	0.780	0.324	0	Macrophages_2
Cacna1d.5	0	1.603	0.854	0.412	0	Macrophages_2
Cd180.4	0	1.596	0.839	0.233	0	Macrophages_2
Abhd12.5	0	1.594	0.949	0.498	0	Macrophages_2
Rasa1.5	0	1.574	0.960	0.668	0	Macrophages_2
Blnk.5	0	1.565	0.934	0.401	0	Macrophages_2
Efr3b.1	0	1.562	0.735	0.267	0	Macrophages_2
Rgs1.1	0	1.559	0.690	0.159	0	Macrophages_2
Prkch.3	0	1.558	0.888	0.371	0	Macrophages_2
Ubash3b.5	0	1.544	0.856	0.4	0	Macrophages_2
Tnfrsf11a.5	0	1.535	0.887	0.379	0	Macrophages_2
Tnip3.3	0	1.534	0.621	0.19	0	Macrophages_2
Lgmn.5	0	1.512	0.921	0.361	0	Macrophages_2
Slc37a2.2	0	1.480	0.801	0.229	0	Macrophages_2
Ptchd1.3	0	1.468	0.754	0.259	0	Macrophages_2
Sirpa.5	0	1.468	0.905	0.422	0	Macrophages_2
Adam8.2	0	1.461	0.739	0.148	0	Macrophages_2
Cd200r1.5	0	1.459	0.872	0.361	0	Macrophages_2
Nav2.4	0	1.443	0.789	0.37	0	Macrophages_2
Card11.3	0	1.433	0.707	0.275	0	Macrophages_2
Bcar3.5	0	1.420	0.895	0.342	0	Macrophages_2
Tcirg1.2	0	1.387	0.810	0.276	0	Macrophages_2
Tns3.4	0	1.386	0.891	0.422	0	Macrophages_2
Atp6v1a.3	0	1.385	0.871	0.432	0	Macrophages_2
Ftl1.4	0	1.374	0.714	0.239	0	Macrophages_2
Il7r.4	0	1.354	0.696	0.216	0	Macrophages_2
Cdk6.3	0	1.341	0.806	0.447	0	Macrophages_2
Fnbp1l.3	0	1.333	0.805	0.337	0	Macrophages_2
Gusb.2	0	1.319	0.757	0.176	0	Macrophages_2
Mfsd12	0	1.307	0.756	0.135	0	Macrophages_2
Trem2	0	1.293	0.694	0.131	0	Macrophages_2
Soat1.4	0	1.282	0.781	0.262	0	Macrophages_2
Pik3r5.5	0	1.270	0.818	0.329	0	Macrophages_2
Shtn1.5	0	1.260	0.932	0.394	0	Macrophages_2

Slc6a6.4	0	1.239	0.932	0.491	0	Macrophages_2
Hexa.4	0	1.238	0.839	0.325	0	Macrophages_2
mt-Nd6.2	0	1.236	0.925	0.654	0	Macrophages_2
Cd300lb.5	0	1.226	0.797	0.281	0	Macrophages_2
Atp8b4.5	0	1.226	0.873	0.301	0	Macrophages_2
Ctsz.3	0	1.217	0.708	0.226	0	Macrophages_2
Grn.5	0	1.216	0.764	0.303	0	Macrophages_2
Mki67.1	0	1.215	0.624	0.224	0	Macrophages_2
Igf2bp2.1	0	1.212	0.689	0.199	0	Macrophages_2
Lrp12.1	0	1.187	0.789	0.48	0	Macrophages_2
Hpgds.3	0	1.171	0.809	0.381	0	Macrophages_2
Csf2ra.5	0	1.169	0.865	0.332	0	Macrophages_2
Plxna4.5	0	1.156	0.791	0.426	0	Macrophages_2
Slc16a10.4	0	1.155	0.921	0.535	0	Macrophages_2
Lmna.1	0	1.154	0.691	0.278	0	Macrophages_2
Lilr4b.4	0	1.150	0.741	0.263	0	Macrophages_2
Trim25.5	0	1.148	0.794	0.431	0	Macrophages_2
Wfs1	0	1.147	0.566	0.121	0	Macrophages_2
Noct.3	0	1.127	0.812	0.38	0	Macrophages_2
Hmga2	0	1.120	0.587	0.174	0	Macrophages_2
Nckap1l.5	0	1.120	0.888	0.352	0	Macrophages_2
Top2a.1	0	1.120	0.631	0.212	0	Macrophages_2
Myo10.1	0	1.119	0.764	0.345	0	Macrophages_2
Gm38248	0	1.093	0.617	0.185	0	Macrophages_2
B4galt5.3	0	1.081	0.837	0.499	0	Macrophages_2
Adgre1.5	0	1.079	0.915	0.427	0	Macrophages_2
Cpeb4.5	0	1.070	0.907	0.535	0	Macrophages_2
Insyn2b.5	0	1.069	0.804	0.361	0	Macrophages_2
Pik3ap1.4	0	1.068	0.873	0.42	0	Macrophages_2
Itgb2.4	0	1.065	0.712	0.168	0	Macrophages_2
Stap1.3	0	1.064	0.632	0.217	0	Macrophages_2
Pld3	0	1.060	0.608	0.142	0	Macrophages_2
Calm2.2	0	1.059	0.817	0.389	0	Macrophages_2
Fyb.5	0	1.053	0.942	0.456	0	Macrophages_2
Kcnk13.5	0	1.052	0.849	0.408	0	Macrophages_2
Rnf128	0	1.050	0.698	0.16	0	Macrophages_2
Nrp2.5	0	1.024	0.811	0.37	0	Macrophages_2
Clec12a.3	0	1.022	0.717	0.201	0	Macrophages_2
Rnf149.4	0	1.020	0.804	0.357	0	Macrophages_2
Tec.5	0	1.015	0.820	0.374	0	Macrophages_2
Slc25a13.2	0	1.012	0.719	0.271	0	Macrophages_2
Cd300a.5	0	1.011	0.788	0.292	0	Macrophages_2
Ctsa.3	0	1.010	0.677	0.186	0	Macrophages_2
Mtss1.4	0	0.997	0.956	0.552	0	Macrophages_2
Lat2.1	0	0.990	0.678	0.177	0	Macrophages_2
Ptpn22.5	0	0.983	0.765	0.299	0	Macrophages_2
Ehd4.5	0	0.979	0.853	0.381	0	Macrophages_2
Akr1a1.2	0	0.956	0.744	0.321	0	Macrophages_2
Dot1l	0	0.953	0.750	0.396	0	Macrophages_2
Cd68	0	0.922	0.600	0.129	0	Macrophages_2
Neil3.1	0	0.914	0.576	0.221	0	Macrophages_2
Tmem106a.5	0	0.909	0.801	0.334	0	Macrophages_2
Actn1	0	0.907	0.676	0.257	0	Macrophages_2
Kcnn4	0	0.903	0.579	0.113	0	Macrophages_2
Ankdd1a	0	0.900	0.659	0.181	0	Macrophages_2
Fam20c	0	0.876	0.687	0.254	0	Macrophages_2
C3ar1.3	0	0.863	0.697	0.229	0	Macrophages_2
Capg	0	0.851	0.624	0.167	0	Macrophages_2
St8sia4.5	0	0.848	0.794	0.341	0	Macrophages_2
Naa50.1	0	0.817	0.738	0.327	0	Macrophages_2
Gpr137b	0	0.809	0.661	0.25	0	Macrophages_2
Efhd2	0	0.779	0.658	0.199	0	Macrophages_2

Spi1.5	0	0.777	0.774	0.323	0	Macrophages_2
Plxdc1	0	0.755	0.656	0.213	0	Macrophages_2
Wdfy4.5	0	0.747	0.812	0.348	0	Macrophages_2
Cdk15	0	0.738	0.642	0.237	0	Macrophages_2
Cpe	0	0.734	0.632	0.217	0	Macrophages_2
Aph1c	0	0.717	0.634	0.227	0	Macrophages_2
C5ar1.5	0	0.703	0.696	0.272	0	Macrophages_2
Kif23.1	0	0.693	0.568	0.187	0	Macrophages_2
Brca1.1	0	0.681	0.644	0.311	0	Macrophages_2
Myh15	0	0.664	0.575	0.183	0	Macrophages_2
Gm15155	0	0.657	0.592	0.247	0	Macrophages_2
Plau	0	0.648	0.560	0.114	0	Macrophages_2
Lair1.1	0	0.626	0.673	0.276	0	Macrophages_2
Tm4sf19	0	0.521	0.513	0.123	0	Macrophages_2
Tns1.2	0	-1.592	0.679	0.89	0	Macrophages_2
Itih5.4	0	-1.679	0.242	0.593	0	Macrophages_2
Arhgap29.3	0	-1.682	0.274	0.601	0	Macrophages_2
Tead1.4	0	-1.997	0.405	0.715	0	Macrophages_2
Eda.3	0	-2.226	0.453	0.74	0	Macrophages_2
Ptprm.3	0	-2.233	0.523	0.828	0	Macrophages_2
Sparc.4	0	-2.268	0.217	0.627	0	Macrophages_2
Ptprg.4	0	-2.302	0.416	0.728	0	Macrophages_2
Svep1.3	0	-2.374	0.308	0.683	0	Macrophages_2
Lama4.3	0	-2.551	0.417	0.689	0	Macrophages_2
Adamts12.3	0	-2.577	0.472	0.781	0	Macrophages_2
Aldh1a1.4	0	-2.649	0.476	0.774	0	Macrophages_2
Adamts5.3	0	-2.883	0.426	0.728	0	Macrophages_2
Adamtsl1.5	0	-2.904	0.458	0.787	0	Macrophages_2
Mid1.4	0	-3.016	0.385	0.755	0	Macrophages_2
Dcn.4	0	-3.104	0.306	0.674	0	Macrophages_2
Gsn.3	0	-3.628	0.276	0.777	0	Macrophages_2
Col3a1.5	0	-3.805	0.472	0.782	0	Macrophages_2
Nox4.4	8.63E-308	-3.085	0.481	0.736	1.73E-304	Macrophages_2
Vim.1	1.21E-307	1.081	0.731	0.36	2.42E-304	Macrophages_2
Lonrf3	1.43E-306	0.810	0.660	0.353	2.86E-303	Macrophages_2
Mpeg1.4	3.79E-305	0.796	0.619	0.161	7.59E-302	Macrophages_2
Vgll3.2	1.50E-304	-2.008	0.350	0.64	2.99E-301	Macrophages_2
P2rx7.4	2.23E-304	0.718	0.747	0.361	4.47E-301	Macrophages_2
Thsd7a.4	2.59E-303	-2.576	0.451	0.734	5.17E-300	Macrophages_2
Col12a1.4	1.47E-301	-1.283	0.049	0.441	2.93E-298	Macrophages_2
Ptprd.3	5.19E-301	-2.166	0.354	0.633	1.04E-297	Macrophages_2
Mfap5.4	3.05E-300	-2.136	0.217	0.577	6.10E-297	Macrophages_2
Slc39a11	7.53E-300	1.218	0.817	0.554	1.51E-296	Macrophages_2
Fgf13.5	1.17E-295	1.780	0.726	0.374	2.34E-292	Macrophages_2
Col1a2.5	3.57E-295	-3.134	0.406	0.668	7.14E-292	Macrophages_2
Eif4a1	1.53E-294	0.818	0.659	0.273	3.06E-291	Macrophages_2
Atp1a1.4	2.63E-294	0.724	0.660	0.233	5.26E-291	Macrophages_2
Dst.3	5.93E-293	-2.543	0.406	0.701	1.19E-289	Macrophages_2
F7	1.29E-289	0.958	0.543	0.149	2.59E-286	Macrophages_2
Ghr.5	2.88E-289	-4.405	0.446	0.686	5.75E-286	Macrophages_2
Gramd1b.3	6.86E-289	0.800	0.758	0.39	1.37E-285	Macrophages_2
Adap2.4	9.46E-289	0.702	0.888	0.516	1.89E-285	Macrophages_2
Ywhah	3.01E-288	0.647	0.611	0.206	6.02E-285	Macrophages_2
Col6a3.3	1.03E-287	-1.611	0.218	0.593	2.06E-284	Macrophages_2
Pik3cb.4	7.80E-287	0.714	0.717	0.293	1.56E-283	Macrophages_2
Abcg1.2	9.53E-287	0.929	0.668	0.252	1.91E-283	Macrophages_2
Pltp.4	4.06E-283	0.839	0.829	0.376	8.11E-280	Macrophages_2
Col14a1.4	5.78E-281	-2.722	0.448	0.708	1.16E-277	Macrophages_2
Cenpe.1	4.06E-279	0.698	0.551	0.196	8.12E-276	Macrophages_2
Cd200r4	6.37E-279	0.608	0.572	0.194	1.27E-275	Macrophages_2
Cybb.5	2.40E-276	0.783	0.720	0.288	4.79E-273	Macrophages_2
Col6a2.3	5.09E-275	-1.323	0.052	0.467	1.02E-271	Macrophages_2

Mical2.1	1.05E-274	0.913	0.720	0.405	2.10E-271	Macrophages_2
Grk3.4	1.34E-274	0.632	0.682	0.303	2.67E-271	Macrophages_2
Pi16.3	2.65E-274	-2.644	0.244	0.565	5.29E-271	Macrophages_2
Pacrg.4	1.60E-273	0.691	0.704	0.381	3.20E-270	Macrophages_2
Sema3d.3	2.78E-273	-1.076	0.027	0.409	5.56E-270	Macrophages_2
Nrip1.4	2.73E-269	-2.021	0.452	0.748	5.47E-266	Macrophages_2
Tpm1	3.05E-268	0.753	0.640	0.268	6.10E-265	Macrophages_2
Col5a3.4	5.98E-266	-1.629	0.298	0.607	1.20E-262	Macrophages_2
Postn.5	3.97E-264	-1.561	0.100	0.479	7.94E-261	Macrophages_2
Msr1.5	5.67E-264	0.659	0.708	0.341	1.13E-260	Macrophages_2
Aplp2.4	7.95E-263	0.801	0.831	0.454	1.59E-259	Macrophages_2
Clec3b.4	1.61E-259	-2.070	0.191	0.556	3.22E-256	Macrophages_2
Ggta1	3.77E-259	0.891	0.598	0.22	7.55E-256	Macrophages_2
Cotl1.5	4.84E-259	0.754	0.683	0.259	9.67E-256	Macrophages_2
Knl1.1	1.10E-258	1.041	0.588	0.229	2.21E-255	Macrophages_2
Entpd1.5	7.18E-258	0.861	0.794	0.439	1.44E-254	Macrophages_2
Bmper.2	2.63E-257	-1.668	0.376	0.639	5.26E-254	Macrophages_2
Cd9.4	2.18E-248	0.802	0.644	0.252	4.36E-245	Macrophages_2
Acss3.2	3.83E-248	-2.087	0.416	0.636	7.66E-245	Macrophages_2
Ulbp1	1.23E-247	0.698	0.592	0.237	2.46E-244	Macrophages_2
Ap1s3	2.45E-244	0.585	0.538	0.182	4.90E-241	Macrophages_2
Clec4a2.3	9.66E-240	0.664	0.616	0.214	1.93E-236	Macrophages_2
Ell2.4	1.04E-238	0.941	0.820	0.537	2.09E-235	Macrophages_2
Ctsk	2.10E-238	0.662	0.485	0.12	4.20E-235	Macrophages_2
Incenp.1	1.09E-237	0.551	0.540	0.204	2.19E-234	Macrophages_2
Anxa2.4	2.10E-237	0.721	0.811	0.483	4.20E-234	Macrophages_2
Fbln2.5	3.24E-237	-1.635	0.132	0.51	6.48E-234	Macrophages_2
Asph.2	7.77E-237	0.993	0.931	0.733	1.55E-233	Macrophages_2
Nr3c2.3	8.10E-234	-1.445	0.504	0.721	1.62E-230	Macrophages_2
Pcolce2.3	2.88E-233	-1.587	0.199	0.527	5.75E-230	Macrophages_2
Aox1.2	1.95E-232	-1.261	0.223	0.512	3.90E-229	Macrophages_2
Mafb.5	2.41E-231	0.760	0.702	0.283	4.82E-228	Macrophages_2
Sgcd.4	4.78E-231	-3.026	0.443	0.638	9.56E-228	Macrophages_2
Sema3e.4	2.11E-229	-2.174	0.321	0.573	4.22E-226	Macrophages_2
Rab7b.1	2.27E-228	0.874	0.764	0.483	4.54E-225	Macrophages_2
Itgbl1.3	6.36E-228	-2.626	0.467	0.689	1.27E-224	Macrophages_2
Cyba	9.20E-227	0.722	0.555	0.173	1.84E-223	Macrophages_2
Clic1	1.57E-224	0.642	0.599	0.233	3.15E-221	Macrophages_2
Kif15.1	8.58E-224	0.933	0.578	0.254	1.72E-220	Macrophages_2
Tenm4.5	3.82E-223	-0.740	0.783	0.351	7.65E-220	Macrophages_2
Vdac2	8.69E-223	0.584	0.594	0.242	1.74E-219	Macrophages_2
Bcl2l1	1.20E-219	0.918	0.772	0.553	2.41E-216	Macrophages_2
Igfbp5.5	5.71E-219	-2.119	0.110	0.451	1.14E-215	Macrophages_2
Pcsk5.3	1.18E-218	-2.076	0.431	0.66	2.36E-215	Macrophages_2
Sec61g	1.03E-216	0.642	0.521	0.155	2.07E-213	Macrophages_2
Xylt1.1	1.30E-216	0.999	0.839	0.583	2.61E-213	Macrophages_2
Efna5.5	3.38E-216	-2.501	0.457	0.675	6.76E-213	Macrophages_2
Rel.3	2.80E-215	0.604	0.621	0.267	5.60E-212	Macrophages_2
Ank.3	6.14E-215	0.770	0.782	0.456	1.23E-211	Macrophages_2
Sdc2.5	1.70E-214	-1.665	0.370	0.619	3.40E-211	Macrophages_2
Diaph3.3	2.20E-214	1.334	0.712	0.483	4.40E-211	Macrophages_2
Prkd1.4	4.87E-213	-2.454	0.416	0.61	9.73E-210	Macrophages_2
Ccr5.5	8.27E-213	0.856	0.645	0.297	1.65E-209	Macrophages_2
Wwtr1.3	1.82E-211	-1.467	0.333	0.565	3.64E-208	Macrophages_2
Sntg1.4	1.56E-210	-2.259	0.352	0.607	3.11E-207	Macrophages_2
Shisa9	4.08E-210	0.557	0.600	0.362	8.16E-207	Macrophages_2
Auts2.4	5.25E-205	-2.223	0.470	0.696	1.05E-201	Macrophages_2
Ccl9.4	1.03E-204	0.765	0.596	0.21	2.06E-201	Macrophages_2
Tmem165.1	2.36E-204	0.752	0.790	0.52	4.73E-201	Macrophages_2
Pdzd2.3	1.68E-203	-1.732	0.296	0.572	3.35E-200	Macrophages_2
Pkm	9.36E-201	0.640	0.573	0.228	1.87E-197	Macrophages_2
Abca8a.4	1.42E-200	-2.101	0.413	0.62	2.83E-197	Macrophages_2

Sms	3.65E-200	0.575	0.557	0.224	7.30E-197	Macrophages_2
Slc11a1.3	6.39E-199	0.610	0.595	0.22	1.28E-195	Macrophages_2
Osbpl3.2	1.93E-198	0.613	0.752	0.513	3.87E-195	Macrophages_2
Foxo1.2	1.04E-196	-1.603	0.457	0.699	2.07E-193	Macrophages_2
Slc7a8.4	1.60E-196	0.618	0.682	0.364	3.19E-193	Macrophages_2
Prkcb.5	1.03E-195	0.779	0.752	0.417	2.05E-192	Macrophages_2
Ccdc80.3	2.55E-195	-2.114	0.287	0.559	5.10E-192	Macrophages_2
Gfpt2.4	8.89E-195	-2.272	0.363	0.6	1.78E-191	Macrophages_2
Thrb.3	2.14E-194	-1.376	0.684	0.827	4.28E-191	Macrophages_2
Epn2.2	1.11E-193	-1.740	0.274	0.597	2.21E-190	Macrophages_2
Rnh1	1.18E-193	0.577	0.587	0.253	2.36E-190	Macrophages_2
Cit.1	5.20E-192	0.639	0.548	0.251	1.04E-188	Macrophages_2
Vcan.4	6.19E-191	-1.741	0.318	0.597	1.24E-187	Macrophages_2
Kif11.1	1.35E-190	0.767	0.542	0.247	2.69E-187	Macrophages_2
Rasgef1b	1.54E-190	0.637	0.634	0.391	3.08E-187	Macrophages_2
Smc2.1	2.87E-189	0.575	0.473	0.164	5.75E-186	Macrophages_2
Hspa5.1	1.08E-187	0.839	0.666	0.355	2.15E-184	Macrophages_2
Clec4b1.3	3.24E-187	0.704	0.577	0.207	6.47E-184	Macrophages_2
Rps6ka2	4.14E-186	0.575	0.559	0.292	8.27E-183	Macrophages_2
Dclk1.5	4.17E-186	-1.705	0.417	0.635	8.34E-183	Macrophages_2
Ccbe1.4	6.37E-186	-0.856	0.121	0.356	1.27E-182	Macrophages_2
F3.3	9.08E-185	-0.657	0.089	0.261	1.82E-181	Macrophages_2
Anxa5.2	2.97E-184	0.656	0.610	0.307	5.93E-181	Macrophages_2
Tpx2.1	4.96E-184	0.645	0.529	0.223	9.93E-181	Macrophages_2
Calr	7.39E-183	0.786	0.581	0.258	1.48E-179	Macrophages_2
Smc4.2	2.14E-182	0.813	0.659	0.384	4.29E-179	Macrophages_2
Atf3	2.39E-182	0.960	0.508	0.111	4.77E-179	Macrophages_2
Nek6	4.63E-182	0.663	0.693	0.492	9.26E-179	Macrophages_2
Lyz2.5	1.76E-180	0.752	0.681	0.274	3.53E-177	Macrophages_2
Col1a1.4	3.54E-179	-2.378	0.263	0.539	7.07E-176	Macrophages_2
Adam23.3	4.81E-179	-1.063	0.327	0.538	9.62E-176	Macrophages_2
B3galt1.2	1.33E-178	-2.015	0.427	0.62	2.66E-175	Macrophages_2
Itgax	2.78E-177	0.862	0.499	0.115	5.56E-174	Macrophages_2
H3f3b	6.97E-176	0.605	0.561	0.233	1.39E-172	Macrophages_2
Cd93	3.33E-175	0.576	0.513	0.159	6.66E-172	Macrophages_2
Rorb.4	3.02E-174	-1.555	0.310	0.549	6.03E-171	Macrophages_2
Prdx1	1.89E-169	0.867	0.547	0.229	3.77E-166	Macrophages_2
Galnt17.5	2.57E-169	-1.496	0.409	0.594	5.14E-166	Macrophages_2
Gabra3.4	3.23E-169	-1.682	0.376	0.566	6.45E-166	Macrophages_2
Anln.1	1.81E-168	0.649	0.539	0.287	3.63E-165	Macrophages_2
Pkib	1.20E-166	0.517	0.558	0.322	2.39E-163	Macrophages_2
Cachd1.3	7.16E-166	-1.511	0.451	0.584	1.43E-162	Macrophages_2
C4b.1	1.41E-165	-1.379	0.287	0.572	2.83E-162	Macrophages_2
Prkg1.4	1.85E-165	-2.364	0.572	0.746	3.70E-162	Macrophages_2
Gm5150.3	7.10E-165	0.860	0.567	0.264	1.42E-161	Macrophages_2
Basp1.4	1.52E-164	0.592	0.723	0.449	3.04E-161	Macrophages_2
Aff2.3	2.89E-164	-1.307	0.188	0.477	5.79E-161	Macrophages_2
Mms22l.1	4.44E-163	0.617	0.651	0.44	8.88E-160	Macrophages_2
Chn2.4	1.26E-162	0.602	0.747	0.502	2.52E-159	Macrophages_2
Nkain2.2	1.02E-161	-0.661	0.092	0.352	2.05E-158	Macrophages_2
Fblim1	1.29E-161	0.645	0.602	0.344	2.57E-158	Macrophages_2
Celf4.3	1.40E-161	0.582	0.589	0.288	2.81E-158	Macrophages_2
Fign.3	2.42E-160	-1.042	0.242	0.483	4.85E-157	Macrophages_2
Kitl.1	8.62E-160	0.812	0.848	0.695	1.72E-156	Macrophages_2
Mtmr10	2.19E-156	0.729	0.717	0.49	4.39E-153	Macrophages_2
Eln.4	2.22E-156	-2.057	0.315	0.538	4.44E-153	Macrophages_2
Pla2g7.5	8.05E-156	0.677	0.627	0.301	1.61E-152	Macrophages_2
Brip1.1	1.15E-154	0.784	0.588	0.364	2.30E-151	Macrophages_2
Pcdh19.5	1.81E-152	-1.601	0.386	0.565	3.62E-149	Macrophages_2
Ly6a.4	8.12E-152	-1.252	0.010	0.391	1.62E-148	Macrophages_2
Gria3.3	3.79E-151	-1.309	0.397	0.588	7.58E-148	Macrophages_2
Dpyd.2	4.85E-151	-1.302	0.442	0.63	9.70E-148	Macrophages_2

Map1b.4	6.57E-151	-0.879	0.100	0.365	1.31E-147	Macrophages_2
Sertad2.1	3.50E-148	0.826	0.690	0.496	6.99E-145	Macrophages_2
Sfrp4.4	3.86E-148	-0.923	0.002	0.335	7.72E-145	Macrophages_2
Timp3.5	6.90E-147	-1.350	0.201	0.487	1.38E-143	Macrophages_2
Kif4.1	1.05E-145	0.539	0.470	0.189	2.10E-142	Macrophages_2
Col4a2.4	1.12E-145	-1.541	0.228	0.502	2.24E-142	Macrophages_2
Slc1a5.4	1.78E-145	-2.066	0.274	0.523	3.55E-142	Macrophages_2
Actg1	4.39E-145	0.569	0.517	0.201	8.78E-142	Macrophages_2
Angpt1.3	2.95E-144	-1.615	0.379	0.549	5.91E-141	Macrophages_2
Prc1.1	1.12E-143	0.641	0.493	0.2	2.24E-140	Macrophages_2
Ccser1.2	1.44E-143	-0.905	0.345	0.533	2.88E-140	Macrophages_2
Pde11a.4	6.24E-143	-1.219	0.267	0.443	1.25E-139	Macrophages_2
Pcdh9.3	1.37E-141	-2.493	0.478	0.616	2.73E-138	Macrophages_2
Bmp6.4	4.40E-141	-1.119	0.274	0.481	8.80E-138	Macrophages_2
Oxct1.2	9.90E-141	0.518	0.749	0.475	1.98E-137	Macrophages_2
Cd83.1	1.98E-140	0.656	0.510	0.2	3.96E-137	Macrophages_2
Robo1.4	2.19E-140	-1.836	0.406	0.593	4.38E-137	Macrophages_2
Nusap1.1	2.53E-140	0.519	0.512	0.268	5.05E-137	Macrophages_2
Efcab11.1	1.01E-139	0.798	0.547	0.321	2.03E-136	Macrophages_2
Prickle1.2	3.52E-139	-1.483	0.366	0.581	7.04E-136	Macrophages_2
Mmp19	4.90E-139	0.816	0.441	0.144	9.79E-136	Macrophages_2
Dhrs9	6.41E-139	0.600	0.516	0.251	1.28E-135	Macrophages_2
Snta1.1	4.32E-137	-0.807	0.254	0.524	8.63E-134	Macrophages_2
Ror1.4	9.03E-137	-1.605	0.485	0.632	1.81E-133	Macrophages_2
Ano3.4	9.11E-137	-1.583	0.293	0.491	1.82E-133	Macrophages_2
Adam12.5	2.30E-136	-1.964	0.357	0.559	4.61E-133	Macrophages_2
Coro2a.3	3.24E-136	0.585	0.567	0.267	6.48E-133	Macrophages_2
Xkr4.3	3.36E-136	-1.310	0.317	0.499	6.72E-133	Macrophages_2
Bcl2l11	6.29E-136	0.546	0.541	0.308	1.26E-132	Macrophages_2
Errfi1.4	7.40E-136	-1.667	0.323	0.568	1.48E-132	Macrophages_2
Igsf6.1	3.60E-135	0.592	0.561	0.245	7.20E-132	Macrophages_2
Mis18bp1.1	1.49E-134	0.759	0.530	0.269	2.97E-131	Macrophages_2
Malt1.2	2.93E-134	0.807	0.679	0.447	5.86E-131	Macrophages_2
Rgs6.3	3.49E-133	-1.644	0.379	0.545	6.98E-130	Macrophages_2
Clic4.2	3.73E-133	0.637	0.759	0.524	7.47E-130	Macrophages_2
Itpr1.2	1.23E-132	-0.816	0.382	0.648	2.46E-129	Macrophages_2
Lamp1.1	9.27E-132	0.547	0.588	0.325	1.85E-128	Macrophages_2
Igfbp6.4	1.59E-131	-1.269	0.008	0.328	3.19E-128	Macrophages_2
Serpinf1.4	1.45E-130	-1.199	0.020	0.361	2.91E-127	Macrophages_2
Arid5b.4	2.78E-129	0.764	0.856	0.671	5.56E-126	Macrophages_2
Mmp3.3	5.15E-128	-0.918	0.052	0.335	1.03E-124	Macrophages_2
Stox2.3	1.98E-127	-0.956	0.235	0.493	3.96E-124	Macrophages_2
Slfn2	1.26E-126	0.507	0.481	0.144	2.52E-123	Macrophages_2
Kif20b.1	3.20E-126	0.506	0.462	0.221	6.40E-123	Macrophages_2
Tcaf1.2	1.92E-124	-1.107	0.264	0.472	3.83E-121	Macrophages_2
Ect2.1	2.84E-124	0.626	0.520	0.301	5.68E-121	Macrophages_2
Sel1l3	2.97E-124	0.543	0.549	0.36	5.94E-121	Macrophages_2
Ercc6	3.07E-124	0.538	0.650	0.456	6.14E-121	Macrophages_2
C3.4	8.40E-124	-1.286	0.013	0.342	1.68E-120	Macrophages_2
Dok2.4	1.03E-122	0.508	0.558	0.232	2.06E-119	Macrophages_2
Magi1.2	7.93E-117	-1.651	0.382	0.578	1.59E-113	Macrophages_2
Chrdl1.2	1.37E-116	-0.915	0.227	0.433	2.75E-113	Macrophages_2
Jph1.2	4.02E-116	-0.523	0.154	0.352	8.03E-113	Macrophages_2
Pola1.2	1.83E-115	0.878	0.785	0.626	3.65E-112	Macrophages_2
Atad2.1	1.37E-114	0.741	0.646	0.454	2.74E-111	Macrophages_2
Dlg2.3	1.50E-114	-1.214	0.330	0.483	2.99E-111	Macrophages_2
Gpc3.4	1.09E-113	-1.119	0.242	0.457	2.19E-110	Macrophages_2
Ism1.2	1.92E-112	-0.637	0.073	0.348	3.84E-109	Macrophages_2
Plxnc1.5	2.63E-112	0.515	0.600	0.311	5.25E-109	Macrophages_2
Plk2	5.74E-109	0.652	0.465	0.168	1.15E-105	Macrophages_2
Sema5a.4	7.35E-108	-0.719	0.183	0.397	1.47E-104	Macrophages_2
Syne1.2	1.69E-107	0.547	0.856	0.675	3.38E-104	Macrophages_2

Cdh9.3	1.74E-105	-1.010	0.321	0.49	3.47E-102	Macrophages_2
Pcolce.3	2.60E-105	-0.773	0.026	0.354	5.20E-102	Macrophages_2
Aebp1.4	4.72E-105	-1.272	0.033	0.352	9.45E-102	Macrophages_2
Col4a1.4	6.49E-105	-1.889	0.385	0.548	1.30E-101	Macrophages_2
Dmd.4	3.92E-104	-1.548	0.461	0.56	7.84E-101	Macrophages_2
Aspn.4	7.49E-104	-1.370	0.258	0.432	1.50E-100	Macrophages_2
Lgr4.2	8.73E-104	-1.289	0.255	0.473	1.75E-100	Macrophages_2
Fos	1.72E-103	0.619	0.425	0.094	3.44E-100	Macrophages_2
Cenpf.1	8.10E-102	0.633	0.489	0.241	1.62E-98	Macrophages_2
Tank.4	1.55E-101	0.553	0.822	0.613	3.10E-98	Macrophages_2
Ksr2	2.97E-101	0.719	0.492	0.236	5.93E-98	Macrophages_2
Prss23.4	6.66E-100	-0.836	0.004	0.28	1.33E-96	Macrophages_2
Pmepa1.2	1.62E-99	-1.263	0.246	0.45	3.24E-96	Macrophages_2
Abca8b.4	3.82E-99	-1.196	0.294	0.48	7.65E-96	Macrophages_2
Kcnt2.3	5.29E-99	-1.490	0.505	0.611	1.06E-95	Macrophages_2
Racgap1.1	1.14E-98	0.509	0.381	0.117	2.27E-95	Macrophages_2
Slc2a13.3	1.21E-98	-1.248	0.389	0.493	2.42E-95	Macrophages_2
Cd63	7.29E-98	0.650	0.430	0.133	1.46E-94	Macrophages_2
Gabre.4	7.43E-98	-0.627	0.189	0.381	1.49E-94	Macrophages_2
Col6a1.4	9.73E-98	-0.988	0.032	0.341	1.95E-94	Macrophages_2
Epha3.4	6.72E-97	-1.390	0.301	0.453	1.34E-93	Macrophages_2
Nlgn1.3	1.03E-96	-0.774	0.089	0.311	2.05E-93	Macrophages_2
Ppp1r9a.1	1.52E-96	-0.941	0.594	0.705	3.04E-93	Macrophages_2
Nr1h4.3	2.97E-96	-1.086	0.339	0.499	5.94E-93	Macrophages_2
Chst11.2	6.30E-94	1.247	0.663	0.543	1.26E-90	Macrophages_2
Large1.2	8.29E-94	-0.958	0.828	0.854	1.66E-90	Macrophages_2
Car8.4	1.54E-93	-0.966	0.201	0.381	3.08E-90	Macrophages_2
Foxp2.4	4.16E-93	-0.900	0.098	0.323	8.33E-90	Macrophages_2
Irs1.3	6.02E-93	-1.294	0.343	0.483	1.20E-89	Macrophages_2
Ntn4.4	4.35E-92	-1.152	0.217	0.433	8.70E-89	Macrophages_2
Prex2.2	5.67E-89	-1.187	0.375	0.518	1.13E-85	Macrophages_2
Ikzf2.1	7.51E-89	-0.870	0.455	0.594	1.50E-85	Macrophages_2
Smoc2.3	6.89E-88	-1.012	0.245	0.453	1.38E-84	Macrophages_2
Cd248.4	1.85E-85	-0.893	0.009	0.285	3.70E-82	Macrophages_2
Anxa1.2	7.13E-84	0.700	0.746	0.559	1.43E-80	Macrophages_2
Meox2.3	9.48E-82	-1.201	0.316	0.463	1.90E-78	Macrophages_2
Plac8.2	2.57E-81	-0.554	0.125	0.39	5.14E-78	Macrophages_2
Plaur	5.39E-81	0.602	0.415	0.135	1.08E-77	Macrophages_2
Tln2.3	6.12E-81	-1.208	0.307	0.489	1.22E-77	Macrophages_2
Gpc4.2	2.32E-80	-0.689	0.155	0.363	4.65E-77	Macrophages_2
Il1r2.3	6.66E-80	-0.585	0.067	0.297	1.33E-76	Macrophages_2
Pde1a.4	7.46E-80	-1.296	0.385	0.51	1.49E-76	Macrophages_2
Csgalnact1.4	2.54E-79	-1.137	0.469	0.574	5.07E-76	Macrophages_2
Fgf7.3	8.88E-79	-0.804	0.229	0.398	1.78E-75	Macrophages_2
Vit.2	9.85E-79	-0.545	0.007	0.272	1.97E-75	Macrophages_2
Sema3a.3	9.98E-79	-0.693	0.185	0.389	2.00E-75	Macrophages_2
Vcl.4	6.37E-78	0.530	0.748	0.542	1.27E-74	Macrophages_2
Slit2.2	4.94E-76	-0.889	0.181	0.372	9.89E-73	Macrophages_2
Col8a1.5	3.28E-75	-1.006	0.235	0.387	6.56E-72	Macrophages_2
Serpinh1.3	4.80E-75	-0.774	0.017	0.303	9.59E-72	Macrophages_2
Serpine2.2	1.27E-74	-0.566	0.034	0.263	2.54E-71	Macrophages_2
Vegfc.2	1.75E-72	-0.662	0.254	0.383	3.49E-69	Macrophages_2
Chsy3.2	2.47E-72	-0.588	0.357	0.515	4.95E-69	Macrophages_2
Negr1.4	7.96E-71	-1.159	0.696	0.768	1.59E-67	Macrophages_2
Fmo1.2	8.43E-69	-0.821	0.242	0.4	1.69E-65	Macrophages_2
Ptprk.3	9.77E-68	-1.098	0.269	0.448	1.95E-64	Macrophages_2
Fabp4.4	1.76E-67	-1.519	0.340	0.496	3.51E-64	Macrophages_2
Hmcn2.3	1.97E-67	-1.289	0.269	0.432	3.94E-64	Macrophages_2
Pde7a.3	1.63E-65	-1.059	0.666	0.713	3.25E-62	Macrophages_2
Lvrn.3	2.20E-62	-1.614	0.485	0.556	4.40E-59	Macrophages_2
Sema6a.1	4.48E-61	-0.773	0.481	0.576	8.96E-58	Macrophages_2
Mycbp2.4	8.13E-61	-1.194	0.869	0.85	1.63E-57	Macrophages_2

Colec12.2	4.53E-60	-0.790	0.575	0.666	9.06E-57	Macrophages_2
Eya2.2	6.02E-59	-0.758	0.326	0.443	1.20E-55	Macrophages_2
Acyp2.2	8.40E-59	-1.335	0.632	0.674	1.68E-55	Macrophages_2
Zfp423.2	1.13E-58	-1.262	0.471	0.501	2.26E-55	Macrophages_2
Bgn.4	4.94E-58	-0.988	0.002	0.27	9.88E-55	Macrophages_2
Mical3.2	2.42E-57	-1.167	0.351	0.49	4.84E-54	Macrophages_2
Fam78b.1	8.11E-57	0.583	0.625	0.515	1.62E-53	Macrophages_2
Col15a1.4	6.00E-55	-1.056	0.274	0.415	1.20E-51	Macrophages_2
Boc.2	1.45E-54	-1.011	0.407	0.488	2.89E-51	Macrophages_2
Igfbp7.4	5.61E-53	-1.224	0.228	0.394	1.12E-49	Macrophages_2
Nipal2.3	6.20E-53	-0.807	0.141	0.325	1.24E-49	Macrophages_2
Gria4.4	3.01E-52	-1.239	0.283	0.396	6.02E-49	Macrophages_2
Tmem267	1.27E-50	0.538	0.388	0.213	2.54E-47	Macrophages_2
Fhit.3	5.38E-50	-0.964	0.774	0.828	1.08E-46	Macrophages_2
Mgll.3	9.06E-50	-1.392	0.382	0.459	1.81E-46	Macrophages_2
Gucy1a2.3	3.55E-49	-0.806	0.281	0.415	7.10E-46	Macrophages_2
Sult1e1.3	2.28E-48	-0.959	0.153	0.324	4.55E-45	Macrophages_2
Apod.4	2.58E-48	-0.715	0.092	0.3	5.17E-45	Macrophages_2
Prickle2.2	5.49E-48	-0.994	0.363	0.479	1.10E-44	Macrophages_2
Aldh1a3.4	5.81E-48	-0.825	0.195	0.345	1.16E-44	Macrophages_2
Prdm16.2	1.18E-47	-0.636	0.251	0.361	2.37E-44	Macrophages_2
Mageb18.1	2.67E-47	-1.664	0.308	0.141	5.33E-44	Macrophages_2
C1qtnf7.4	1.02E-46	-1.355	0.385	0.485	2.05E-43	Macrophages_2
Ppfia2.3	3.12E-46	-0.549	0.131	0.271	6.24E-43	Macrophages_2
Msrb3.3	3.16E-46	-1.405	0.418	0.513	6.31E-43	Macrophages_2
Tshr.3	4.51E-46	-1.703	0.355	0.173	9.02E-43	Macrophages_2
C7.3	7.61E-46	-2.458	0.377	0.43	1.52E-42	Macrophages_2
Pdzrn3.4	1.18E-45	-1.730	0.352	0.461	2.35E-42	Macrophages_2
Fmo2.5	3.72E-45	-1.197	0.157	0.332	7.44E-42	Macrophages_2
Kcnq1.2	9.28E-45	0.583	0.643	0.539	1.86E-41	Macrophages_2
Cdh13.3	1.65E-44	-1.311	0.415	0.51	3.31E-41	Macrophages_2
Nrep.3	2.31E-44	-0.718	0.254	0.366	4.61E-41	Macrophages_2
Sox6.3	9.91E-43	-0.735	0.372	0.187	1.98E-39	Macrophages_2
Smoc1.2	1.43E-42	-1.129	0.327	0.164	2.87E-39	Macrophages_2
Pck1.1	1.37E-41	-0.557	0.274	0.137	2.74E-38	Macrophages_2
Plcxd3.4	3.34E-40	-1.332	0.284	0.379	6.69E-37	Macrophages_2
Adamts6	9.10E-40	-0.620	0.487	0.582	1.82E-36	Macrophages_2
Hpgd.1	9.54E-40	-0.720	0.489	0.57	1.91E-36	Macrophages_2
Slc1a3.3	2.08E-39	-0.956	0.198	0.274	4.17E-36	Macrophages_2
Tnik.2	2.68E-38	-0.605	0.316	0.417	5.35E-35	Macrophages_2
Ppp2r2b.3	5.71E-38	-1.379	0.324	0.451	1.14E-34	Macrophages_2
Trabd2b.3	3.78E-37	-0.911	0.268	0.364	7.55E-34	Macrophages_2
Plpp1.3	3.96E-37	-0.663	0.406	0.515	7.91E-34	Macrophages_2
Kcnq5.3	1.04E-36	-1.050	0.412	0.492	2.07E-33	Macrophages_2
Cenpp.2	1.32E-36	0.644	0.598	0.498	2.65E-33	Macrophages_2
Enah.3	1.85E-34	-0.618	0.220	0.33	3.71E-31	Macrophages_2
Tmtc2.3	1.29E-33	-0.669	0.478	0.584	2.59E-30	Macrophages_2
Npr3.3	2.87E-33	-2.337	0.333	0.177	5.73E-30	Macrophages_2
Mme.4	2.93E-33	-0.762	0.294	0.39	5.87E-30	Macrophages_2
Txlng.3	3.56E-33	-0.920	0.616	0.643	7.12E-30	Macrophages_2
Dact2.4	1.44E-32	-0.623	0.144	0.272	2.88E-29	Macrophages_2
Pde10a.3	5.13E-32	-0.955	0.330	0.411	1.03E-28	Macrophages_2
Cfh.4	1.46E-31	-0.829	0.722	0.524	2.92E-28	Macrophages_2
Pde4d.3	2.18E-31	-0.691	0.836	0.609	4.35E-28	Macrophages_2
Adamtsl3.3	3.95E-30	-1.014	0.382	0.451	7.90E-27	Macrophages_2
Rnf24.2	5.06E-30	-0.646	0.223	0.335	1.01E-26	Macrophages_2
Cp	1.63E-28	-0.664	0.516	0.553	3.26E-25	Macrophages_2
Pnpla3.1	2.28E-28	-1.299	0.276	0.153	4.56E-25	Macrophages_2
Ezh2.1	4.62E-28	0.521	0.537	0.458	9.25E-25	Macrophages_2
Cadps2.3	9.77E-28	-0.601	0.222	0.358	1.95E-24	Macrophages_2
Ugdh.3	1.49E-27	-1.075	0.409	0.471	2.98E-24	Macrophages_2
Prkar2b.4	5.52E-27	-2.190	0.234	0.287	1.10E-23	Macrophages_2

Dnm3.3	4.73E-26	-0.581	0.586	0.643	9.46E-23	Macrophages_2
Snhg11.3	1.51E-25	-0.775	0.212	0.324	3.01E-22	Macrophages_2
Plekhg5.4	9.63E-25	-0.660	0.606	0.452	1.93E-21	Macrophages_2
Lsamp.3	1.37E-23	-0.594	0.175	0.311	2.73E-20	Macrophages_2
Pde7b.5	1.46E-23	-0.820	0.755	0.793	2.92E-20	Macrophages_2
Slc25a21.4	4.44E-23	-0.654	0.193	0.288	8.89E-20	Macrophages_2
Ntrk2.3	4.89E-23	-1.029	0.418	0.422	9.79E-20	Macrophages_2
Rgs7bp.2	5.13E-23	-0.610	0.288	0.42	1.03E-19	Macrophages_2
Lyve1.5	7.27E-23	-0.604	0.245	0.269	1.45E-19	Macrophages_2
Sgip1.4	9.43E-23	-0.875	0.442	0.501	1.89E-19	Macrophages_2
Prr16.3	1.07E-22	-1.921	0.376	0.396	2.15E-19	Macrophages_2
Ccdc3.2	1.21E-22	-0.762	0.253	0.352	2.41E-19	Macrophages_2
Ldb2.4	1.60E-22	-1.176	0.441	0.474	3.19E-19	Macrophages_2
Smpd3.3	3.74E-22	-0.687	0.679	0.526	7.49E-19	Macrophages_2
Nhs.2	1.01E-21	-0.590	0.156	0.286	2.02E-18	Macrophages_2
Sned1.2	1.01E-21	-0.868	0.351	0.447	2.03E-18	Macrophages_2
Gpm6b.3	1.33E-21	-0.881	0.265	0.371	2.66E-18	Macrophages_2
Mctp2.4	1.65E-21	-0.569	0.515	0.354	3.29E-18	Macrophages_2
Adamts16.3	2.16E-20	-1.124	0.296	0.376	4.32E-17	Macrophages_2
Optn.4	4.53E-20	-0.573	0.353	0.265	9.06E-17	Macrophages_2
Dkk2.4	8.06E-20	-0.886	0.294	0.372	1.61E-16	Macrophages_2
Gas6.5	5.16E-18	-0.664	0.429	0.457	1.03E-14	Macrophages_2
Fam13a.3	9.15E-18	-1.085	0.473	0.354	1.83E-14	Macrophages_2
Tmem135.4	9.45E-18	-0.787	0.774	0.573	1.89E-14	Macrophages_2
Sorbs1.4	1.08E-17	-2.795	0.407	0.281	2.15E-14	Macrophages_2
St6galnac5.2	1.38E-17	-0.515	0.342	0.284	2.77E-14	Macrophages_2
Rgs7.4	7.76E-17	-0.959	0.358	0.374	1.55E-13	Macrophages_2
Ddx60.5	2.15E-16	-0.782	0.643	0.662	4.30E-13	Macrophages_2
Slc24a3.3	3.94E-15	-1.425	0.576	0.598	7.88E-12	Macrophages_2
Col4a5.4	2.60E-14	-0.795	0.475	0.482	5.21E-11	Macrophages_2
F13a1.4	3.97E-14	-1.180	0.714	0.536	7.94E-11	Macrophages_2
Skap1.3	8.25E-14	-0.501	0.203	0.26	1.65E-10	Macrophages_2
Cd163.5	1.23E-13	-0.904	0.569	0.418	2.45E-10	Macrophages_2
Ranbp17.3	3.10E-13	-0.539	0.270	0.302	6.19E-10	Macrophages_2
Tmem45b.3	7.34E-13	-1.597	0.305	0.19	1.47E-09	Macrophages_2
Fam126b.5	1.37E-12	-0.997	0.399	0.332	2.75E-09	Macrophages_2
Fgf10.4	1.66E-11	-1.212	0.329	0.371	3.32E-08	Macrophages_2
Tsc22d3.5	2.09E-11	-0.931	0.539	0.52	4.17E-08	Macrophages_2
Nrg4.3	6.63E-11	-1.198	0.295	0.18	1.33E-07	Macrophages_2
Bckdhb.4	8.12E-11	-1.314	0.537	0.543	1.62E-07	Macrophages_2
Ly6e.2	1.05E-10	-0.561	0.171	0.301	2.10E-07	Macrophages_2
Adipor2.4	8.96E-10	-1.059	0.472	0.366	1.79E-06	Macrophages_2
Dcstamp	1.16E-09	0.730	0.312	0.082	2.32E-06	Macrophages_2
Inpp4b.3	1.18E-09	-0.505	0.566	0.499	2.35E-06	Macrophages_2
Tbc1d4.4	2.74E-09	-0.647	0.776	0.76	5.49E-06	Macrophages_2
Gm42906.1	4.60E-09	-1.650	0.284	0.17	9.20E-06	Macrophages_2
Peg3.3	5.57E-09	-0.731	0.278	0.252	1.11E-05	Macrophages_2
Nnat.3	6.04E-09	-1.429	0.312	0.195	1.21E-05	Macrophages_2
Lpgat1.4	1.91E-08	-1.549	0.407	0.335	3.82E-05	Macrophages_2
Cidec.2	5.48E-08	-1.810	0.272	0.167	0.000109541	Macrophages_2
Galnt18.2	8.57E-08	-0.554	0.294	0.33	0.000171305	Macrophages_2
Fry.5	1.14E-07	-1.112	0.661	0.473	0.000228323	Macrophages_2
Enpp2.4	1.58E-07	-1.439	0.296	0.348	0.000315649	Macrophages_2
Hacd2.5	2.13E-07	-1.448	0.302	0.288	0.000426275	Macrophages_2
Dtna.2	1.71E-06	-0.616	0.302	0.362	0.003415136	Macrophages_2
Cat.4	1.71E-06	-0.639	0.291	0.262	0.00342172	Macrophages_2
Kcnc2.3	4.59E-06	-0.969	0.216	0.313	0.009176101	Macrophages_2
Ngf.4	1.02E-05	-0.617	0.303	0.311	0.02039126	Macrophages_2
Grb14.3	4.64E-05	-0.949	0.333	0.309	0.092711847	Macrophages_2
Dram1.4	0.0001405	-0.681	0.289	0.248	0.281098377	Macrophages_2
Eya4.3	0.0002139	-0.779	0.594	0.573	0.427747296	Macrophages_2
Csmd1.3	0.0002412	-1.210	0.263	0.302	0.482421243	Macrophages_2

Ctnna3.3	0.0005217	-0.678	0.283	0.254	1	Macrophages_2
Cobll1.3	0.0008165	-0.993	0.436	0.437	1	Macrophages_2
Shroom3.2	0.0011462	-0.636	0.238	0.31	1	Macrophages_2
Sik2.4	0.0027559	-1.041	0.764	0.603	1	Macrophages_2
Gm4951.1	0.0041581	-0.545	0.553	0.549	1	Macrophages_2
Mecom.2	0.005002	-0.517	0.431	0.433	1	Macrophages_2
Tox.4	0.005563	-0.730	0.295	0.336	1	Macrophages_2
Macrod2.2	0.0061275	-1.114	0.551	0.514	1	Macrophages_2
Myl1.3	0.0065343	-0.951	0.289	0.184	1	Macrophages_2
Itk	0	2.974	0.905	0.093	0	NK cells
Clnk	0	2.338	0.825	0.043	0	NK cells
Il23r	0	1.989	0.788	0.056	0	NK cells
Cd6	0	1.507	0.758	0.005	0	NK cells
Olfr1259	0	1.306	0.593	0.006	0	NK cells
Blk	0	1.261	0.805	0.095	0	NK cells
Ly6c2	0	1.233	0.744	0.027	0	NK cells
Klrc2	0	1.175	0.721	0.021	0	NK cells
Cdh10	0	0.937	0.747	0.05	0	NK cells
Klrc1	0	0.897	0.546	0	0	NK cells
Mmp25	0	0.813	0.808	0.017	0	NK cells
Pcsk1	0	0.797	0.518	0.001	0	NK cells
Prf1	0	0.773	0.638	0.007	0	NK cells
Ablim3	0	0.662	0.788	0.091	0	NK cells
Olfr60	0	0.582	0.755	0.049	0	NK cells
Fstl4	0	0.578	0.730	0.009	0	NK cells
Npnt	0	0.570	0.554	0.004	0	NK cells
Fasl	0	0.538	0.604	0.001	0	NK cells
Ikzf3	7.17E-305	2.864	0.816	0.084	1.43E-301	NK cells
Sidt1	1.70E-296	1.540	0.763	0.099	3.41E-293	NK cells
Skap1.4	6.95E-287	4.013	0.930	0.234	1.39E-283	NK cells
Klrb1f	6.30E-278	0.814	0.599	0.033	1.26E-274	NK cells
Stat4	1.18E-245	2.904	0.908	0.206	2.35E-242	NK cells
Klra8	9.19E-242	1.598	0.479	0.039	1.84E-238	NK cells
St6galnac3.1	1.61E-238	3.593	0.914	0.279	3.22E-235	NK cells
Gimap3	3.43E-232	1.849	0.635	0.033	6.86E-229	NK cells
Bcl11b	2.14E-227	1.957	0.643	0.035	4.29E-224	NK cells
Rnf43	1.23E-219	1.271	0.688	0.056	2.47E-216	NK cells
Gimap8	3.65E-217	2.178	0.755	0.116	7.29E-214	NK cells
Btla	2.52E-214	1.166	0.719	0.106	5.03E-211	NK cells
Ccl4	1.61E-200	1.231	0.755	0.117	3.23E-197	NK cells
Klri2	1.43E-193	0.681	0.641	0.016	2.86E-190	NK cells
Samsn1.3	1.91E-192	2.260	0.900	0.229	3.82E-189	NK cells
Txk	4.77E-192	1.431	0.741	0.112	9.55E-189	NK cells
Arap2.4	2.21E-183	1.964	0.880	0.238	4.43E-180	NK cells
Camk4	5.77E-172	2.782	0.813	0.245	1.15E-168	NK cells
Mreg	6.00E-168	0.518	0.482	0.047	1.20E-164	NK cells
Il18r1	2.17E-167	2.677	0.850	0.233	4.35E-164	NK cells
Gata3	3.66E-167	2.211	0.613	0.055	7.32E-164	NK cells
Tespa1	3.12E-161	1.867	0.696	0.071	6.25E-158	NK cells
Grap2	3.87E-156	2.765	0.791	0.143	7.73E-153	NK cells
Tox.5	1.34E-151	3.543	0.836	0.317	2.69E-148	NK cells
Klrk1	1.94E-144	1.763	0.727	0.088	3.88E-141	NK cells
Ets1	2.78E-136	2.484	0.836	0.32	5.57E-133	NK cells
Cd163l1	9.50E-135	2.284	0.808	0.36	1.90E-131	NK cells
Kit	6.27E-134	0.679	0.582	0.048	1.25E-130	NK cells
Gpr183	9.06E-133	0.937	0.799	0.224	1.81E-129	NK cells
Inpp4b.4	2.21E-126	3.830	0.900	0.499	4.43E-123	NK cells
Unc5cl	2.71E-125	0.545	0.507	0.013	5.42E-122	NK cells
Tnik.3	6.91E-125	2.102	0.855	0.391	1.38E-121	NK cells
Nr4a3	8.42E-124	0.625	0.602	0.059	1.68E-120	NK cells
Gramd1b.4	2.77E-121	2.046	0.928	0.432	5.54E-118	NK cells
Strip2	1.02E-116	1.026	0.808	0.175	2.05E-113	NK cells

Mageb18.2	8.34E-107	-1.565	0.682	0.152	1.67E-103	NK cells
Gimap7	2.39E-106	0.545	0.253	0.039	4.78E-103	NK cells
Il7r.5	3.19E-104	2.188	0.774	0.274	6.38E-101	NK cells
Tnfrsf9	2.12E-103	0.623	0.568	0.09	4.25E-100	NK cells
Gimap4	1.31E-98	1.841	0.582	0.074	2.62E-95	NK cells
Ripor2.3	5.22E-96	2.629	0.802	0.39	1.04E-92	NK cells
Epsti1.5	7.04E-92	2.572	0.903	0.472	1.41E-88	NK cells
H2-Q7	1.00E-87	1.489	0.699	0.141	2.01E-84	NK cells
Itga4.5	7.72E-86	2.053	0.847	0.405	1.54E-82	NK cells
Trhde.3	2.82E-85	-0.687	0.719	0.198	5.64E-82	NK cells
Ncr1	3.40E-84	1.471	0.526	0.09	6.80E-81	NK cells
Ccr2.5	1.15E-83	1.305	0.805	0.248	2.29E-80	NK cells
Fgf14.1	1.20E-83	-0.724	0.526	0.126	2.40E-80	NK cells
Runx2.1	7.25E-83	1.709	0.886	0.578	1.45E-79	NK cells
Tmem45b.4	2.42E-82	-1.437	0.733	0.194	4.83E-79	NK cells
Atp8b4.6	8.61E-82	1.456	0.889	0.372	1.72E-78	NK cells
Bcl2.3	1.47E-81	2.172	0.930	0.617	2.95E-78	NK cells
Cd247	5.33E-80	2.422	0.694	0.199	1.07E-76	NK cells
Pag1.4	1.43E-79	1.497	0.844	0.429	2.85E-76	NK cells
Sox6.4	4.48E-78	-0.617	0.685	0.202	8.95E-75	NK cells
Csmd1.4	7.55E-78	-0.681	0.752	0.284	1.51E-74	NK cells
Chst11.3	8.72E-77	1.282	0.872	0.553	1.74E-73	NK cells
Lmnb1.1	6.24E-75	0.578	0.822	0.343	1.25E-71	NK cells
Fmo2.6	5.13E-73	-0.794	0.777	0.294	1.03E-69	NK cells
Cotl1.6	9.10E-71	0.978	0.825	0.309	1.82E-67	NK cells
Plin1.1	1.32E-70	-0.934	0.638	0.168	2.64E-67	NK cells
Emb.4	4.38E-70	1.496	0.825	0.423	8.77E-67	NK cells
Fgf13.6	1.07E-69	0.880	0.819	0.415	2.13E-66	NK cells
Lck	3.09E-69	1.392	0.557	0.063	6.19E-66	NK cells
Prkcq	1.10E-68	1.943	0.669	0.232	2.19E-65	NK cells
Jazf1.4	2.40E-68	1.129	0.864	0.504	4.80E-65	NK cells
Atp1a2.1	2.88E-68	-0.879	0.641	0.169	5.76E-65	NK cells
Chd7.4	1.35E-66	0.918	0.858	0.38	2.70E-63	NK cells
Rgs7.5	6.10E-66	-0.920	0.033	0.38	1.22E-62	NK cells
Trac	6.52E-65	0.746	0.279	0.017	1.30E-61	NK cells
Gria4.5	3.33E-64	-0.685	0.791	0.369	6.67E-61	NK cells
Cd84.6	3.31E-63	0.907	0.916	0.457	6.63E-60	NK cells
Osbpl3.3	6.39E-63	1.802	0.788	0.542	1.28E-59	NK cells
Nnat.4	6.17E-62	-1.238	0.660	0.201	1.23E-58	NK cells
Gnai1.3	9.37E-62	-0.792	0.652	0.182	1.87E-58	NK cells
Cd4	3.63E-61	0.654	0.273	0.071	7.26E-58	NK cells
Rab27b	5.50E-61	0.609	0.568	0.133	1.10E-57	NK cells
Sult1e1.4	1.54E-60	-0.638	0.786	0.286	3.08E-57	NK cells
Plek.1	8.47E-59	0.924	0.850	0.424	1.69E-55	NK cells
Cntn1	1.39E-58	1.381	0.549	0.124	2.78E-55	NK cells
Ezh2.2	1.74E-58	0.827	0.847	0.46	3.49E-55	NK cells
Lpin1.3	1.75E-58	-0.619	0.209	0.39	3.50E-55	NK cells
Dnah8	8.18E-58	0.798	0.630	0.181	1.64E-54	NK cells
Il2rb	1.49E-57	1.726	0.465	0.002	2.99E-54	NK cells
Pitpnc1.6	5.38E-57	0.951	0.936	0.717	1.08E-53	NK cells
Fcer1g.4	6.08E-57	0.621	0.769	0.246	1.22E-53	NK cells
Nr1h4.4	9.15E-57	-0.991	0.081	0.486	1.83E-53	NK cells
Insyn2b.6	1.56E-55	0.745	0.877	0.414	3.11E-52	NK cells
Aebp1.5	4.53E-55	-0.947	0.234	0.307	9.06E-52	NK cells
Aoah.5	5.16E-54	0.544	0.855	0.459	1.03E-50	NK cells
Mmp12.4	1.79E-53	-0.514	0.618	0.202	3.57E-50	NK cells
Ighm.3	4.92E-53	0.595	0.738	0.221	9.84E-50	NK cells
Cd3g	2.74E-52	0.661	0.320	0	5.47E-49	NK cells
Mcm6.1	6.99E-52	0.663	0.655	0.207	1.40E-48	NK cells
Cd226.5	7.29E-52	1.962	0.702	0.24	1.46E-48	NK cells
Slc22a23.2	1.40E-51	0.509	0.794	0.365	2.80E-48	NK cells
Macrod2.3	1.53E-50	-1.245	0.234	0.527	3.07E-47	NK cells

Cd5	2.71E-50	0.984	0.499	0.063	5.43E-47	NK cells
Mpped2	1.58E-49	0.604	0.529	0.117	3.16E-46	NK cells
Mctp2.5	1.83E-49	0.986	0.738	0.368	3.66E-46	NK cells
Slc1a3.4	2.17E-49	-0.777	0.702	0.252	4.34E-46	NK cells
Kcnc2.4	6.17E-49	-0.532	0.735	0.288	1.23E-45	NK cells
Plcxd3.5	5.97E-48	-1.012	0.786	0.354	1.19E-44	NK cells
Ly75	1.90E-46	0.754	0.251	0.058	3.80E-43	NK cells
Ryr2.2	7.14E-46	-0.681	0.565	0.182	1.43E-42	NK cells
Ptpn22.6	2.89E-45	1.783	0.730	0.358	5.77E-42	NK cells
Dock5.2	2.90E-45	1.249	0.733	0.371	5.80E-42	NK cells
Dach2	3.81E-45	2.302	0.267	0.1	7.61E-42	NK cells
Apoe.5	3.62E-44	-0.831	0.919	0.497	7.23E-41	NK cells
Klra4	4.96E-44	0.958	0.326	0.029	9.92E-41	NK cells
Nckap1l.6	4.01E-43	0.612	0.850	0.42	8.03E-40	NK cells
Tbc1d8.1	5.32E-43	0.507	0.808	0.309	1.06E-39	NK cells
Tmem123	1.99E-42	1.274	0.699	0.281	3.97E-39	NK cells
Ly6a.5	8.80E-42	-0.555	0.256	0.337	1.76E-38	NK cells
Lcp1.6	9.67E-42	0.951	0.816	0.456	1.93E-38	NK cells
Bcl2l1.1	9.88E-42	0.887	0.866	0.578	1.98E-38	NK cells
Pmepa1.3	3.89E-41	-0.990	0.304	0.423	7.79E-38	NK cells
Myo3b	1.42E-40	0.714	0.270	0.142	2.83E-37	NK cells
Nav2.5	1.54E-40	2.041	0.708	0.425	3.08E-37	NK cells
Gpnmb.5	2.00E-40	-1.013	0.802	0.387	4.00E-37	NK cells
Anxa1.3	5.75E-40	-0.755	0.320	0.593	1.15E-36	NK cells
Prkcb.6	6.28E-40	0.878	0.822	0.457	1.26E-36	NK cells
Nlrc5.2	2.77E-39	1.361	0.699	0.426	5.55E-36	NK cells
Ptprcap	7.58E-39	1.122	0.504	0.005	1.52E-35	NK cells
Specc1.5	2.01E-38	0.879	0.886	0.512	4.03E-35	NK cells
Slc1a5.5	2.94E-38	-1.655	0.373	0.489	5.87E-35	NK cells
Fam13a.4	3.56E-38	-0.888	0.788	0.361	7.12E-35	NK cells
Ccdc80.4	7.47E-38	-1.803	0.357	0.523	1.49E-34	NK cells
Igfbp6.5	2.46E-37	-0.865	0.273	0.281	4.92E-34	NK cells
Igfbp7.5	2.85E-37	-0.564	0.315	0.371	5.70E-34	NK cells
Kcnq1.3	3.05E-37	-0.691	0.376	0.559	6.11E-34	NK cells
Tmtc2.4	4.39E-37	0.615	0.295	0.575	8.79E-34	NK cells
Optn.5	9.18E-37	-0.648	0.721	0.266	1.84E-33	NK cells
Smoc2.4	1.05E-36	-0.508	0.783	0.413	2.09E-33	NK cells
Irs1.4	9.25E-36	-1.036	0.150	0.47	1.85E-32	NK cells
Kcnq5.4	1.23E-35	2.454	0.682	0.475	2.46E-32	NK cells
Calm2.3	6.88E-35	0.567	0.858	0.441	1.38E-31	NK cells
Gucy1a2.4	3.46E-34	-0.583	0.783	0.386	6.92E-31	NK cells
Epha3.5	4.10E-34	-0.706	0.788	0.421	8.20E-31	NK cells
Car8.5	4.24E-34	-0.603	0.730	0.345	8.48E-31	NK cells
Ppp1r16b	7.90E-34	1.487	0.574	0.125	1.58E-30	NK cells
Meox2.4	6.80E-33	-0.712	0.791	0.433	1.36E-29	NK cells
F13a1.5	1.46E-32	-0.819	0.914	0.553	2.91E-29	NK cells
Srgap3.1	1.46E-32	0.531	0.738	0.404	2.93E-29	NK cells
Ank.4	1.59E-32	0.648	0.861	0.495	3.18E-29	NK cells
Slc9a7	1.72E-32	0.871	0.749	0.372	3.44E-29	NK cells
Malt1.3	2.75E-32	1.372	0.744	0.474	5.50E-29	NK cells
Rgs6.4	1.03E-31	-1.287	0.201	0.529	2.07E-28	NK cells
Dst.4	2.68E-31	-1.506	0.574	0.66	5.35E-28	NK cells
Rasa1.6	6.85E-31	0.639	0.947	0.705	1.37E-27	NK cells
Msrb3.4	7.47E-31	-0.791	0.365	0.503	1.49E-27	NK cells
Zc3h12c	5.04E-30	0.698	0.811	0.472	1.01E-26	NK cells
Traf1	3.35E-29	1.080	0.565	0.119	6.71E-26	NK cells
Cd44.6	9.12E-29	0.580	0.811	0.487	1.82E-25	NK cells
Trabd2b.4	1.15E-28	-0.814	0.081	0.357	2.30E-25	NK cells
Adamtsl3.4	1.17E-28	-0.646	0.791	0.432	2.34E-25	NK cells
Dusp5	1.29E-28	0.582	0.253	0.1	2.58E-25	NK cells
Cadm1.6	1.86E-28	-0.977	0.819	0.392	3.72E-25	NK cells
Fyb.6	3.64E-28	0.604	0.822	0.52	7.28E-25	NK cells

Tns1.3	4.79E-28	-1.243	0.919	0.858	9.57E-25	NK cells
Cyfip2	1.10E-27	1.377	0.568	0.145	2.20E-24	NK cells
Hmcn2.4	1.66E-27	-0.954	0.769	0.398	3.31E-24	NK cells
Pacrg.5	4.83E-27	-0.733	0.777	0.42	9.66E-24	NK cells
Tspan12.3	7.78E-27	-1.018	0.560	0.227	1.56E-23	NK cells
Scml4	1.24E-26	1.577	0.607	0.304	2.47E-23	NK cells
Gbe1.2	1.81E-26	0.523	0.866	0.562	3.61E-23	NK cells
Ly6e.3	3.28E-26	1.326	0.602	0.274	6.56E-23	NK cells
St8sia4.6	5.55E-26	1.087	0.724	0.4	1.11E-22	NK cells
Itgal	8.72E-26	0.929	0.546	0.059	1.74E-22	NK cells
Sgip1.5	1.73E-25	-0.559	0.749	0.486	3.46E-22	NK cells
Me1.4	2.28E-25	-1.029	0.560	0.241	4.56E-22	NK cells
Bmp6.5	5.61E-25	-0.811	0.791	0.442	1.12E-21	NK cells
Itpr1.3	1.02E-24	0.656	0.855	0.603	2.04E-21	NK cells
Fmo1.3	3.70E-24	-0.567	0.769	0.367	7.41E-21	NK cells
Gfod1	3.78E-24	0.901	0.649	0.374	7.56E-21	NK cells
Tshr.4	5.67E-24	-1.645	0.493	0.192	1.13E-20	NK cells
Cavin2.2	7.58E-24	-0.598	0.474	0.191	1.52E-20	NK cells
Themis	9.89E-24	2.176	0.329	0.135	1.98E-20	NK cells
Chrdl1.3	1.12E-23	-0.700	0.772	0.393	2.23E-20	NK cells
Nrg4.4	1.27E-23	-1.112	0.487	0.19	2.54E-20	NK cells
Rab27a	2.21E-23	1.497	0.540	0.071	4.41E-20	NK cells
Mbnl3	2.37E-23	0.747	0.538	0.134	4.75E-20	NK cells
Spata13	3.64E-23	1.380	0.540	0.136	7.28E-20	NK cells
Icos	5.78E-23	0.866	0.290	0.245	1.16E-19	NK cells
Por.3	9.14E-23	-0.630	0.231	0.397	1.83E-19	NK cells
Cd300a.6	9.65E-23	-0.545	0.825	0.353	1.93E-19	NK cells
Pik3r5.6	4.07E-22	0.998	0.696	0.393	8.14E-19	NK cells
Dtna.3	5.02E-22	-0.553	0.064	0.361	1.00E-18	NK cells
Cobll1.4	5.07E-22	1.252	0.669	0.431	1.01E-18	NK cells
Prkch.4	1.11E-21	1.443	0.680	0.441	2.22E-18	NK cells
Col4a2.5	1.27E-21	-1.224	0.407	0.463	2.53E-18	NK cells
Pola1.3	1.74E-21	0.563	0.875	0.644	3.48E-18	NK cells
Sema3d.4	2.49E-21	-0.741	0.774	0.342	4.97E-18	NK cells
Ppp1r9a.2	3.25E-21	-1.062	0.811	0.686	6.50E-18	NK cells
Tbx21	3.51E-21	1.139	0.312	0.085	7.03E-18	NK cells
Col8a1.6	3.85E-21	-0.894	0.727	0.356	7.70E-18	NK cells
Acox1.4	3.91E-21	-0.653	0.786	0.368	7.83E-18	NK cells
Nebl	4.25E-21	2.028	0.515	0.135	8.50E-18	NK cells
Tspan18.2	4.96E-21	-0.527	0.716	0.319	9.92E-18	NK cells
Lipa.3	8.22E-21	-0.614	0.730	0.296	1.64E-17	NK cells
Id2	1.03E-20	1.067	0.549	0.127	2.06E-17	NK cells
Col15a1.5	1.13E-20	-0.831	0.769	0.384	2.25E-17	NK cells
Ctla2a	1.20E-20	0.936	0.290	0.017	2.39E-17	NK cells
Snhg11.4	1.34E-20	-0.718	0.045	0.315	2.67E-17	NK cells
Cdh13.4	2.69E-20	-0.876	0.799	0.488	5.38E-17	NK cells
Asph.3	6.50E-20	-1.080	0.855	0.759	1.30E-16	NK cells
Mpp7.5	2.10E-19	-1.352	0.217	0.305	4.19E-16	NK cells
Atp6v0d2.6	2.16E-19	-1.018	0.799	0.415	4.33E-16	NK cells
Klra9	4.62E-19	0.584	0.345	0.007	9.25E-16	NK cells
Mical3.3	4.70E-19	-0.757	0.382	0.472	9.39E-16	NK cells
Prkar2b.5	5.66E-19	-2.027	0.680	0.269	1.13E-15	NK cells
Coro2a.4	6.31E-19	1.287	0.607	0.303	1.26E-15	NK cells
Ggta1.1	6.90E-19	0.622	0.337	0.274	1.38E-15	NK cells
C7.4	7.83E-19	-1.612	0.788	0.413	1.57E-15	NK cells
Gpm6b.4	8.00E-19	-0.623	0.685	0.347	1.60E-15	NK cells
Mme.5	8.72E-19	-0.636	0.139	0.382	1.74E-15	NK cells
Plxnc1.6	9.08E-19	0.964	0.354	0.354	1.82E-15	NK cells
Ccnd2	1.49E-18	1.274	0.591	0.333	2.98E-15	NK cells
Lef1	1.97E-18	1.175	0.343	0.062	3.94E-15	NK cells
Prex2.3	2.14E-18	-0.663	0.819	0.489	4.29E-15	NK cells
Stap1.4	3.28E-18	0.736	0.610	0.269	6.56E-15	NK cells

Timp3.6	3.86E-18	-1.033	0.265	0.45	7.73E-15	NK cells
Prr16.4	7.03E-18	-1.617	0.772	0.384	1.41E-14	NK cells
Cacna1d.6	7.37E-18	-0.510	0.841	0.468	1.47E-14	NK cells
Pi16.4	1.65E-17	-1.673	0.504	0.518	3.30E-14	NK cells
Sytl3	2.35E-17	1.071	0.334	0.051	4.71E-14	NK cells
Plxna4.6	5.60E-17	-0.550	0.827	0.471	1.12E-13	NK cells
Ms4a4b	6.18E-17	2.295	0.524	0.137	1.24E-13	NK cells
Rel.4	6.86E-17	0.801	0.318	0.319	1.37E-13	NK cells
Klri1	8.67E-17	0.611	0.493	0.101	1.73E-13	NK cells
Klrd1	9.64E-17	0.790	0.284	0.05	1.93E-13	NK cells
Ccl5	1.02E-16	2.212	0.345	0.019	2.03E-13	NK cells
Angpt1.4	1.10E-16	-1.052	0.571	0.523	2.20E-13	NK cells
Ptprk.4	1.30E-16	-0.718	0.786	0.412	2.60E-13	NK cells
Anxa2.5	2.13E-16	-0.619	0.407	0.535	4.26E-13	NK cells
Cfh.5	2.29E-16	-0.838	0.875	0.545	4.59E-13	NK cells
Sorbs1.5	3.02E-16	-2.042	0.295	0.3	6.03E-13	NK cells
Cat.5	3.81E-16	-0.600	0.150	0.269	7.61E-13	NK cells
Nrcam.2	1.05E-15	-0.546	0.125	0.37	2.10E-12	NK cells
Cd244a	1.70E-15	0.691	0.304	0.146	3.39E-12	NK cells
Aldh1a3.5	1.72E-15	-0.606	0.655	0.314	3.44E-12	NK cells
Enpp2.5	2.67E-15	-1.286	0.092	0.347	5.34E-12	NK cells
Selenop.5	3.05E-15	-0.629	0.855	0.475	6.10E-12	NK cells
Sema4d	3.25E-15	1.153	0.560	0.26	6.50E-12	NK cells
Cd52	5.38E-15	1.321	0.507	0.13	1.08E-11	NK cells
Lgmn.6	6.71E-15	-0.729	0.825	0.433	1.34E-11	NK cells
Aspn.5	7.59E-15	-1.009	0.805	0.396	1.52E-11	NK cells
Pde7a.4	1.04E-14	0.707	0.875	0.702	2.07E-11	NK cells
Abca8b.5	1.81E-14	-0.806	0.802	0.444	3.62E-11	NK cells
Lpgat1.5	2.68E-14	-1.565	0.212	0.349	5.37E-11	NK cells
Pecam1	3.50E-14	0.670	0.304	0.203	7.01E-11	NK cells
Kcnk13.6	3.86E-14	-0.633	0.819	0.464	7.72E-11	NK cells
Gabre.5	4.34E-14	-0.553	0.189	0.357	8.67E-11	NK cells
Pdzrn3.5	6.08E-14	-1.270	0.799	0.436	1.22E-10	NK cells
Ctsl.6	1.35E-13	-0.677	0.627	0.309	2.70E-10	NK cells
Samd3	2.45E-13	2.038	0.429	0.016	4.89E-10	NK cells
C3.5	2.49E-13	-1.046	0.072	0.299	4.99E-10	NK cells
Serpinf1.5	2.54E-13	-0.999	0.109	0.316	5.09E-10	NK cells
Foxo1.3	5.05E-13	-0.650	0.613	0.664	1.01E-09	NK cells
Sfrp4.5	5.44E-13	-0.725	0.136	0.29	1.09E-09	NK cells
Hacd2.6	1.08E-12	-1.204	0.206	0.292	2.17E-09	NK cells
Bcar3.6	2.03E-12	-0.895	0.830	0.413	4.07E-09	NK cells
Cgnl1.2	2.18E-12	-0.516	0.780	0.4	4.36E-09	NK cells
Trbc1	2.43E-12	1.804	0.432	0.001	4.86E-09	NK cells
Itm2b.4	2.81E-12	0.525	0.760	0.555	5.62E-09	NK cells
Cxcr6	3.18E-12	1.360	0.493	0.124	6.36E-09	NK cells
Ucp2.2	3.22E-12	0.605	0.565	0.238	6.44E-09	NK cells
Il18rap	3.33E-12	1.377	0.504	0.09	6.65E-09	NK cells
Gimap1	4.48E-12	0.789	0.446	0.003	8.96E-09	NK cells
Prickle2.3	5.67E-12	-0.656	0.772	0.454	1.13E-08	NK cells
Lama4.4	6.39E-12	-2.011	0.791	0.645	1.28E-08	NK cells
Hsd11b1	9.98E-12	1.706	0.543	0.242	2.00E-08	NK cells
C4b.2	1.55E-11	-0.726	0.833	0.522	3.09E-08	NK cells
Ikzf2.2	3.89E-11	2.900	0.604	0.573	7.78E-08	NK cells
Galnt6	4.61E-11	0.774	0.337	0.17	9.21E-08	NK cells
Sorl1	5.85E-11	1.241	0.526	0.182	1.17E-07	NK cells
Nkg7	9.55E-11	1.282	0.334	0.001	1.91E-07	NK cells
Map1b.5	1.17E-10	-0.718	0.131	0.331	2.34E-07	NK cells
Gpc3.5	1.75E-10	-0.854	0.769	0.416	3.50E-07	NK cells
Cytip.2	1.95E-10	1.225	0.540	0.184	3.90E-07	NK cells
C1qtnf7.5	2.43E-10	-1.175	0.404	0.472	4.86E-07	NK cells
Irf8.2	2.80E-10	0.642	0.362	0.272	5.60E-07	NK cells
Ngf.5	4.64E-10	-0.568	0.167	0.313	9.29E-07	NK cells

Relb	5.01E-10	0.910	0.549	0.32	1.00E-06	NK cells
Xkr4.4	5.22E-10	-0.845	0.802	0.464	1.04E-06	NK cells
Tenm4.6	5.69E-10	-2.726	0.805	0.404	1.14E-06	NK cells
Col4a5.5	9.73E-10	-0.768	0.368	0.484	1.95E-06	NK cells
Fgf7.4	1.27E-09	-0.502	0.348	0.374	2.54E-06	NK cells
Ctsb.6	1.34E-09	-0.895	0.844	0.45	2.68E-06	NK cells
Ptprm.4	1.42E-09	-1.579	0.844	0.781	2.83E-06	NK cells
Gsn.4	1.50E-09	-2.329	0.805	0.7	3.00E-06	NK cells
Smc4.3	1.72E-09	0.752	0.630	0.419	3.44E-06	NK cells
Thrb.4	1.94E-09	-1.153	0.869	0.804	3.88E-06	NK cells
Fabp4.5	2.07E-09	-1.630	0.733	0.466	4.14E-06	NK cells
Adamts5.4	2.24E-09	-2.229	0.830	0.68	4.49E-06	NK cells
Pltp.5	2.43E-09	-0.724	0.844	0.432	4.85E-06	NK cells
Stox2.4	2.51E-09	-0.669	0.794	0.446	5.02E-06	NK cells
Eln.5	2.90E-09	-1.418	0.816	0.497	5.79E-06	NK cells
Ldb2.5	3.49E-09	-0.775	0.493	0.468	6.98E-06	NK cells
Vegfa.2	6.51E-09	-0.559	0.036	0.282	1.30E-05	NK cells
Pde4b	8.50E-09	0.971	0.529	0.228	1.70E-05	NK cells
Ubash3b.6	9.06E-09	0.883	0.655	0.463	1.81E-05	NK cells
Adamts16.4	1.02E-08	-0.976	0.214	0.368	2.05E-05	NK cells
Zbtb7c.2	1.70E-08	-0.527	0.825	0.487	3.39E-05	NK cells
Rin2.3	1.80E-08	-0.801	0.864	0.73	3.59E-05	NK cells
Pmp22	2.18E-08	-0.502	0.075	0.354	4.35E-05	NK cells
Errfi1.5	2.42E-08	-1.006	0.819	0.524	4.83E-05	NK cells
Ghr.6	3.80E-08	-4.120	0.836	0.646	7.60E-05	NK cells
Col4a1.5	3.84E-08	-1.289	0.827	0.516	7.69E-05	NK cells
Ppfia2.4	3.87E-08	-0.501	0.042	0.256	7.74E-05	NK cells
Eepd1.5	5.98E-08	-0.660	0.429	0.37	0.000119519	NK cells
Slc2a13.4	7.14E-08	-0.885	0.797	0.469	0.000142777	NK cells
Gapdh	8.38E-08	0.501	0.267	0.138	0.000167545	NK cells
Mid1.5	8.49E-08	-1.943	0.861	0.696	0.000169878	NK cells
Ms4a6b.5	1.20E-07	0.512	0.643	0.377	0.000240276	NK cells
Cdh9.4	1.38E-07	-0.718	0.794	0.457	0.000276829	NK cells
Eya4.4	1.47E-07	-1.138	0.643	0.574	0.000293196	NK cells
Lvrn.4	1.60E-07	-1.239	0.797	0.539	0.000320778	NK cells
Serpinh1.4	1.61E-07	-0.590	0.460	0.256	0.000322597	NK cells
Shroom3.3	1.69E-07	-0.585	0.086	0.305	0.000337216	NK cells
Cd3d	2.09E-07	0.694	0.351	0.001	0.00041772	NK cells
Ipcef1.2	2.11E-07	1.118	0.384	0.248	0.000422728	NK cells
Adap2.5	2.78E-07	-0.506	0.866	0.563	0.000555788	NK cells
Pparg.4	2.83E-07	-0.977	0.852	0.586	0.00056521	NK cells
Ccser1.3	3.18E-07	-0.534	0.766	0.498	0.00063583	NK cells
Arid5b.5	3.80E-07	0.651	0.827	0.695	0.000759188	NK cells
Fggy.2	4.61E-07	-0.585	0.813	0.504	0.000921353	NK cells
Fbln2.6	4.68E-07	-1.204	0.772	0.446	0.000935017	NK cells
Ubash3a	4.78E-07	0.939	0.337	0.042	0.000956034	NK cells
Apod.5	7.61E-07	-0.574	0.106	0.274	0.001522937	NK cells
St18.5	8.27E-07	-0.955	0.786	0.431	0.001653522	NK cells
Bmper.3	9.23E-07	-0.955	0.833	0.594	0.001846536	NK cells
Ppp2r2b.4	1.31E-06	-1.200	0.738	0.425	0.002621924	NK cells
Col6a2.4	1.35E-06	-0.987	0.760	0.397	0.002691761	NK cells
Dlg2.4	1.39E-06	-0.593	0.769	0.453	0.002780284	NK cells
Bgn.5	1.49E-06	-0.718	0.368	0.227	0.00298298	NK cells
Gm37004	1.75E-06	1.505	0.379	0.008	0.003495933	NK cells
Blnk.6	2.14E-06	-0.550	0.819	0.471	0.004278753	NK cells
Tns3.5	2.60E-06	-0.639	0.841	0.483	0.005191575	NK cells
Dmd.5	3.27E-06	-0.897	0.819	0.538	0.006545295	NK cells
Card11.4	3.67E-06	1.069	0.543	0.333	0.007331097	NK cells
Lgals3.5	3.84E-06	-0.726	0.448	0.312	0.007684587	NK cells
Sntg1.5	3.91E-06	-1.755	0.788	0.564	0.007824102	NK cells
Mertk.4	4.00E-06	-0.648	0.850	0.567	0.008001281	NK cells
Pip5k1b	4.11E-06	1.386	0.329	0.16	0.008223488	NK cells

Ntn4.5	4.50E-06	-0.894	0.688	0.394	0.00900254	NK cells
Clec3b.5	4.51E-06	-1.459	0.604	0.5	0.009027409	NK cells
Slc27a1.4	4.57E-06	-0.951	0.808	0.428	0.009144692	NK cells
Cav1.3	6.27E-06	-1.344	0.309	0.205	0.012545373	NK cells
Nlgn1.4	6.72E-06	-0.712	0.187	0.281	0.013441238	NK cells
H2afz	7.27E-06	0.684	0.323	0.175	0.014548537	NK cells
Plpp1.4	7.98E-06	-0.660	0.577	0.497	0.015959811	NK cells
Tox2	1.24E-05	0.612	0.362	0.195	0.024785325	NK cells
Aldh1a1.5	1.57E-05	-1.611	0.855	0.727	0.031428702	NK cells
Igfbp5.6	1.62E-05	-1.384	0.735	0.392	0.032439128	NK cells
Rab7b.2	2.91E-05	-0.568	0.816	0.517	0.058202007	NK cells
Ptprg.5	3.43E-05	-1.489	0.847	0.678	0.068516355	NK cells
Robo1.5	3.47E-05	-1.183	0.811	0.559	0.069347619	NK cells
S100a4	3.49E-05	0.745	0.323	0.119	0.069856054	NK cells
Lpl.6	3.69E-05	-1.227	0.858	0.567	0.073794619	NK cells
Pcolce2.4	4.06E-05	-1.053	0.780	0.471	0.081206775	NK cells
Rpl10	5.69E-05	0.765	0.309	0.177	0.113763074	NK cells
Postn.6	6.92E-05	-1.213	0.741	0.415	0.138482422	NK cells
Thsd4.4	7.45E-05	-0.597	0.262	0.283	0.148948178	NK cells
Gask1b.4	0.0001121	-0.851	0.799	0.5	0.224190109	NK cells
Btg1.2	0.0001483	0.804	0.499	0.263	0.296690714	NK cells
Adcy5.4	0.0001554	-0.632	0.089	0.258	0.310824772	NK cells
Tead1.5	0.0001705	-1.585	0.819	0.666	0.341087433	NK cells
Rps27a	0.0001781	0.961	0.329	0.204	0.356269739	NK cells
Pcdh19.6	0.0001855	-1.152	0.808	0.531	0.371023171	NK cells
Gprin3.1	0.000198	0.809	0.471	0.112	0.395905666	NK cells
Pcdh9.4	0.0002497	-1.740	0.819	0.59	0.499406717	NK cells
Pde1a.5	0.0002568	-1.080	0.591	0.489	0.513659182	NK cells
Prkd1.5	0.0002587	-2.088	0.802	0.576	0.517445668	NK cells
Slc24a3.4	0.0002857	-0.803	0.646	0.593	0.571406732	NK cells
P2ry10	0.0003415	0.607	0.351	0.017	0.683064399	NK cells
Sema3a.4	0.0003924	-0.502	0.496	0.355	0.784814217	NK cells
Ugdh.4	0.0004383	-0.848	0.758	0.454	0.876582091	NK cells
Pde7b.6	0.0004429	-0.527	0.872	0.785	0.885717082	NK cells
Fnip2.5	0.0004558	-0.842	0.872	0.744	0.911577491	NK cells
Cdk6.4	0.0004676	1.114	0.577	0.498	0.935105621	NK cells
Lgr4.3	0.0005302	-1.152	0.490	0.44	1	NK cells
Col12a1.5	0.0005966	-1.122	0.696	0.375	1	NK cells
Rasgrf2.1	0.000633	-0.524	0.471	0.208	1	NK cells
Rap1gap2.4	0.0006381	0.622	0.409	0.315	1	NK cells
Dram1.5	0.0006759	-0.798	0.047	0.259	1	NK cells
Rps2	0.0007649	0.826	0.320	0.176	1	NK cells
Sgcd.5	0.0007649	-2.271	0.780	0.605	1	NK cells
Col1a1.5	0.0009943	-1.633	0.788	0.491	1	NK cells
Svep1.4	0.0010495	-1.715	0.816	0.623	1	NK cells
Atp6v0a1.5	0.0010555	-0.879	0.891	0.649	1	NK cells
Tmem135.5	0.0010975	-0.656	0.852	0.596	1	NK cells
Negr1.5	0.0011209	-1.486	0.889	0.754	1	NK cells
Galnt17.6	0.0011242	-1.153	0.802	0.561	1	NK cells
Tes.3	0.001168	0.832	0.513	0.31	1	NK cells
Tank.5	0.0012081	0.570	0.733	0.641	1	NK cells
Cd200r1.6	0.0012218	0.574	0.621	0.431	1	NK cells
Hmgb2	0.0013104	0.633	0.348	0.158	1	NK cells
Il2ra	0.0013786	1.994	0.454	0.06	1	NK cells
Fgf10.5	0.0014621	-0.907	0.515	0.361	1	NK cells
Kcnt2.4	0.0014643	-1.291	0.794	0.59	1	NK cells
Acss3.3	0.0016663	-1.672	0.799	0.598	1	NK cells
Slit2.3	0.0017831	-0.767	0.334	0.344	1	NK cells
Tln2.4	0.0018531	-0.982	0.755	0.454	1	NK cells
Adamts12.4	0.0021671	-1.612	0.861	0.732	1	NK cells
Tagln2	0.00226	0.575	0.345	0.214	1	NK cells
Vcl.5	0.0023009	-0.770	0.721	0.568	1	NK cells

Slc25a21.5	0.0030238	-0.542	0.192	0.276	1	NK cells
Lhfpl2.4	0.003693	-1.167	0.772	0.452	1	NK cells
Tcaf1.3	0.0038616	-0.907	0.758	0.434	1	NK cells
Vgll3.3	0.0041872	-1.615	0.813	0.592	1	NK cells
Pcsk5.4	0.0044113	-1.663	0.799	0.622	1	NK cells
Mgll.4	0.0048639	-1.304	0.741	0.44	1	NK cells
Ctdspl.2	0.0049427	-0.680	0.791	0.518	1	NK cells
B3galt1.3	0.0057992	-1.420	0.727	0.588	1	NK cells
Rpl15	0.0058571	0.779	0.329	0.166	1	NK cells
Eya2.3	0.0060941	0.583	0.462	0.425	1	NK cells
Tpt1	0.0064196	1.662	0.359	0.239	1	NK cells
Hsp90ab1	0.0066087	1.045	0.443	0.288	1	NK cells
Fign.4	0.0074273	-0.798	0.724	0.441	1	NK cells
Mtss1.5	0.0085144	-0.725	0.894	0.604	1	NK cells
Aox1.3	0.0091955	-1.009	0.772	0.462	1	NK cells
Prkg1.5	0.0092361	-1.350	0.883	0.716	1	NK cells
Ror1.5	0.0096717	-0.998	0.808	0.605	1	NK cells
Lgals1	0.0099104	0.817	0.343	0.23	1	NK cells
Thsd7a.5	0.0099192	-1.927	0.833	0.689	1	NK cells

Extened Data Table 8: maker genes for preadipocyte clusters idenfied in 10x snRNA seq of ingWAT CAG-GFP and CAG-RSPO2						
gene	p_val	avg_log2FC	pct.1	pct.2	p_val_adj	cluster
Sema3e	0	2.485	0.823	0.203	0	PA-1
Pi16	0	2.436	0.941	0.408	0	PA-1
Igfbp5	0	2.400	0.829	0.312	0	PA-1
Nav3	0	2.238	0.968	0.641	0	PA-1
Dpp4	0	2.133	0.977	0.507	0	PA-1
Limch1	0	2.098	0.883	0.287	0	PA-1
Anxa3	0	1.932	0.971	0.586	0	PA-1
Creb5	0	1.892	0.994	0.755	0	PA-1
Sema3c	0	1.865	0.987	0.692	0	PA-1
Cd55	0	1.796	0.869	0.403	0	PA-1
Cmah	0	1.771	0.806	0.305	0	PA-1
Efna5	0	1.747	0.786	0.32	0	PA-1
Ston2	0	1.741	0.806	0.295	0	PA-1
Efemp1	0	1.707	0.912	0.495	0	PA-1
Efhd1	0	1.657	0.653	0.155	0	PA-1
Ano3	0	1.590	0.688	0.195	0	PA-1
Tek	0	1.586	0.837	0.402	0	PA-1
Gfpt2	0	1.576	0.927	0.547	0	PA-1
Fbn1	0	1.449	0.998	0.943	0	PA-1
Pla1a	0	1.409	0.777	0.348	0	PA-1
Slco3a1	0	1.167	0.922	0.667	0	PA-1
Axl	0	1.162	0.882	0.596	0	PA-1
Fndc1	0	1.128	0.985	0.819	0	PA-1
Sdk1	0	0.945	0.991	0.804	0	PA-1
Nfib	0	-1.110	0.9	0.982	0	PA-1
Sparcl1	0	-1.272	0.011	0.355	0	PA-1
Myo5a	0	-1.274	0.085	0.493	0	PA-1
Nrp1	0	-1.608	0.293	0.754	0	PA-1
Col15a1	0	-1.646	0.017	0.446	0	PA-1
Igfbp7	0	-1.812	0.234	0.717	0	PA-1
Pde4d	0	-1.839	0.046	0.443	0	PA-1
Vav3	0	-1.885	0.041	0.571	0	PA-1
Bgn	0	-1.945	0.135	0.634	0	PA-1
Ldb2	0	-1.982	0.133	0.649	0	PA-1
Sgip1	0	-2.063	0.034	0.499	0	PA-1
Col4a2	0	-2.111	0.161	0.713	0	PA-1
Bmper	0	-2.116	0.184	0.676	0	PA-1
Hmcn2	0	-2.118	0.133	0.578	0	PA-1
Adam12	0	-2.314	0.061	0.456	0	PA-1
Col4a1	0	-2.369	0.238	0.803	0	PA-1
Fmo2	0	-2.514	0.055	0.502	0	PA-1
Smurf2	3.02E-305	1.176	0.936	0.77	6.02E-301	PA-1
Adamts5	3.42E-304	1.215	0.928	0.726	6.82E-300	PA-1
Eps8	1.97E-301	-1.414	0.228	0.659	3.94E-297	PA-1
F3	2.65E-298	-1.732	0.028	0.376	5.28E-294	PA-1
Adamts16	1.24E-294	1.900	0.486	0.055	2.47E-290	PA-1
Lurap1l	1.15E-291	1.427	0.712	0.279	2.29E-287	PA-1
Hdac7	1.18E-291	1.109	0.884	0.649	2.36E-287	PA-1
Rorb	1.28E-291	1.655	0.657	0.206	2.55E-287	PA-1
Ddr2	6.66E-291	0.875	0.98	0.894	1.33E-286	PA-1
Col6a3	1.97E-289	-1.524	0.41	0.764	3.92E-285	PA-1
Dact2	2.50E-287	1.722	0.477	0.057	5.00E-283	PA-1
Kank1	1.05E-286	1.475	0.6	0.164	2.09E-282	PA-1
Auts2	3.12E-284	-1.380	0.56	0.871	6.23E-280	PA-1
Car8	3.46E-283	1.523	0.553	0.118	6.90E-279	PA-1
Aldh1a3	6.37E-281	1.529	0.529	0.108	1.27E-276	PA-1
Ank2	1.53E-279	-1.329	0.554	0.867	3.05E-275	PA-1
Fn1	8.54E-278	1.046	0.973	0.757	1.70E-273	PA-1

Gan	3.87E-277	1.714	0.633	0.233	7.72E-273	PA-1
Adgrd1	6.32E-276	0.910	0.966	0.815	1.26E-271	PA-1
Galnt16	1.63E-272	1.218	0.797	0.439	3.26E-268	PA-1
Gas6	5.29E-269	-1.253	0.026	0.348	1.06E-264	PA-1
Dock10	1.61E-268	-1.265	0.234	0.646	3.21E-264	PA-1
Ccbe1	2.21E-266	-1.860	0.033	0.357	4.40E-262	PA-1
Pparg	4.53E-264	-1.513	0.027	0.347	9.03E-260	PA-1
Opcml	6.64E-264	0.977	0.984	0.774	1.32E-259	PA-1
Zfp462	6.33E-261	1.260	0.788	0.43	1.26E-256	PA-1
Gria3	6.50E-261	-1.386	0.205	0.622	1.30E-256	PA-1
Mfap5	6.78E-261	1.189	0.898	0.708	1.35E-256	PA-1
Deptor	3.70E-260	-1.449	0.156	0.541	7.38E-256	PA-1
Eya2	3.55E-259	-1.272	0.112	0.496	7.08E-255	PA-1
Pde11a	4.65E-259	-2.170	0.045	0.374	9.28E-255	PA-1
Agmo	1.73E-258	-1.209	0.592	0.896	3.46E-254	PA-1
Pde8a	1.81E-258	1.119	0.845	0.563	3.62E-254	PA-1
Slco2b1	3.45E-256	-1.238	0.085	0.445	6.89E-252	PA-1
Sulf1	1.31E-255	1.002	0.933	0.741	2.62E-251	PA-1
Lum	1.55E-252	-1.311	0.11	0.47	3.10E-248	PA-1
Ackr3	1.39E-250	1.252	0.779	0.439	2.78E-246	PA-1
Nova1	1.98E-250	0.944	0.975	0.855	3.95E-246	PA-1
Gask1b	1.22E-249	-1.314	0.129	0.503	2.43E-245	PA-1
Pcsk6	2.01E-242	1.260	0.745	0.388	4.01E-238	PA-1
Ebf2	1.52E-237	0.897	0.992	0.948	3.03E-233	PA-1
Stxbp6	8.15E-236	1.100	0.843	0.486	1.63E-231	PA-1
Gas7	7.27E-235	1.008	0.939	0.749	1.45E-230	PA-1
Ildr2	6.19E-233	1.325	0.632	0.259	1.23E-228	PA-1
Ebf1	8.51E-233	0.745	0.999	0.995	1.70E-228	PA-1
Adgrg2	2.01E-230	1.221	0.618	0.223	4.01E-226	PA-1
Lpl	6.74E-229	-1.349	0.034	0.33	1.35E-224	PA-1
Plpp1	7.65E-229	-1.272	0.155	0.516	1.53E-224	PA-1
Steap4	9.24E-229	-0.981	0.01	0.273	1.84E-224	PA-1
Svep1	9.72E-229	-1.369	0.488	0.787	1.94E-224	PA-1
Gdf10	1.30E-228	-1.289	0.013	0.28	2.59E-224	PA-1
Itgbl1	6.30E-227	-1.382	0.598	0.854	1.26E-222	PA-1
Fam49a	2.35E-226	-1.190	0.139	0.495	4.69E-222	PA-1
Negr1	1.09E-222	1.592	0.691	0.364	2.17E-218	PA-1
Sbsn	6.06E-222	1.530	0.41	0.059	1.21E-217	PA-1
Samhd1	6.10E-222	1.067	0.818	0.546	1.22E-217	PA-1
Ror1	4.06E-221	-1.209	0.439	0.775	8.10E-217	PA-1
Plcl2	5.85E-221	-1.146	0.074	0.393	1.17E-216	PA-1
Igf1	5.33E-219	-1.269	0.097	0.428	1.06E-214	PA-1
Slc4a4	3.46E-217	0.875	0.939	0.735	6.91E-213	PA-1
Rgs6	1.65E-214	1.769	0.546	0.179	3.29E-210	PA-1
Fyn	9.63E-214	0.784	0.928	0.783	1.92E-209	PA-1
Fgf2	1.83E-212	1.106	0.673	0.314	3.64E-208	PA-1
Frmd6	6.36E-212	-1.194	0.621	0.844	1.27E-207	PA-1
Calcrl	4.13E-211	1.401	0.638	0.274	8.24E-207	PA-1
Nrep	2.72E-209	-1.256	0.127	0.457	5.43E-205	PA-1
Dock4	1.38E-208	1.009	0.916	0.766	2.76E-204	PA-1
Nhsl1	7.45E-208	1.014	0.843	0.589	1.49E-203	PA-1
Ptprj	1.10E-207	0.904	0.861	0.565	2.19E-203	PA-1
Palld	4.12E-204	-1.107	0.045	0.328	8.23E-200	PA-1
Gpc6	6.81E-202	0.766	0.992	0.958	1.36E-197	PA-1
Nid1	1.36E-199	0.790	0.946	0.841	2.71E-195	PA-1
Prkd1	2.24E-198	-1.118	0.406	0.744	4.46E-194	PA-1
Emilin2	2.90E-198	1.033	0.701	0.381	5.79E-194	PA-1
Uap1	2.99E-198	1.135	0.761	0.483	5.97E-194	PA-1
Anxa1	8.89E-198	0.969	0.765	0.453	1.77E-193	PA-1
Dhrs3	8.93E-198	-0.973	0.078	0.375	1.78E-193	PA-1
Gli2	1.37E-197	-0.903	0.053	0.338	2.74E-193	PA-1
Meox2	1.99E-197	-1.718	0.154	0.479	3.98E-193	PA-1

Acvr2a	1.64E-196	-1.226	0.345	0.67	3.28E-192	PA-1
Myo1d	4.39E-195	-0.946	0.014	0.253	8.76E-191	PA-1
Frmd4b	4.53E-195	-1.201	0.473	0.77	9.04E-191	PA-1
Smoc2	1.91E-194	-1.387	0.206	0.529	3.81E-190	PA-1
Sgcd	1.93E-194	-1.291	0.418	0.749	3.85E-190	PA-1
Kif2a	7.62E-191	0.997	0.762	0.487	1.52E-186	PA-1
Reps2	4.18E-188	-1.030	0.187	0.522	8.35E-184	PA-1
Ntn4	7.57E-188	1.169	0.627	0.29	1.51E-183	PA-1
Cacna1c	1.98E-186	-1.223	0.118	0.427	3.95E-182	PA-1
Pcolce2	9.02E-186	1.015	0.781	0.504	1.80E-181	PA-1
Nr1h4	9.31E-186	1.301	0.536	0.205	1.86E-181	PA-1
Cped1	5.63E-185	-0.769	0.89	0.97	1.12E-180	PA-1
Cd9	1.41E-184	-1.019	0.106	0.404	2.81E-180	PA-1
Lifr	6.86E-183	-0.880	0.031	0.276	1.37E-178	PA-1
Ugdh	1.80E-182	1.098	0.696	0.38	3.59E-178	PA-1
Col14a1	2.31E-182	0.930	0.899	0.666	4.61E-178	PA-1
Col5a3	1.86E-181	-1.297	0.371	0.662	3.71E-177	PA-1
Mme	2.14E-181	-1.030	0.042	0.298	4.26E-177	PA-1
Prkca	9.54E-180	0.742	0.956	0.848	1.90E-175	PA-1
Clip1	1.95E-178	0.870	0.818	0.594	3.88E-174	PA-1
Bmp6	5.06E-176	1.323	0.471	0.141	1.01E-171	PA-1
Smpd3	2.30E-175	1.275	0.688	0.362	4.59E-171	PA-1
Mgll	5.61E-175	0.826	0.551	0.209	1.12E-170	PA-1
Tgfbr2	1.76E-174	0.844	0.85	0.662	3.52E-170	PA-1
Prickle1	1.14E-173	-1.429	0.277	0.59	2.27E-169	PA-1
Marcks	2.15E-172	1.128	0.71	0.434	4.29E-168	PA-1
Utrn	5.56E-172	0.606	0.977	0.931	1.11E-167	PA-1
Ltbp1	2.52E-170	0.742	0.922	0.759	5.04E-166	PA-1
Angpt1	2.57E-170	-1.188	0.287	0.614	5.13E-166	PA-1
Pdgfc	3.24E-170	1.127	0.609	0.278	6.46E-166	PA-1
Adamts12	1.00E-168	-1.141	0.792	0.914	2.00E-164	PA-1
Mmp3	9.65E-168	-1.337	0.093	0.373	1.93E-163	PA-1
Pdzd2	2.16E-166	1.016	0.691	0.406	4.31E-162	PA-1
Plxdc2	3.11E-166	0.632	0.989	0.912	6.20E-162	PA-1
Arhgap24	3.62E-165	-0.924	0.868	0.954	7.22E-161	PA-1
Scara5	4.65E-165	0.741	0.92	0.723	9.28E-161	PA-1
Sytl2	5.91E-165	1.017	0.401	0.097	1.18E-160	PA-1
Podn	8.23E-164	-0.880	0.122	0.409	1.64E-159	PA-1
Enpp2	1.29E-162	-1.347	0.085	0.349	2.57E-158	PA-1
Robo1	1.77E-162	1.216	0.685	0.387	3.54E-158	PA-1
Rasa3	5.80E-162	-0.892	0.103	0.385	1.16E-157	PA-1
mt-Co2	6.53E-162	-0.830	0.923	0.983	1.30E-157	PA-1
B3galt1	1.41E-161	-1.041	0.469	0.762	2.82E-157	PA-1
Flnb	1.66E-159	0.845	0.751	0.477	3.31E-155	PA-1
Tiam2	5.54E-159	-0.852	0.034	0.259	1.11E-154	PA-1
Ltbp4	6.47E-159	-1.204	0.169	0.451	1.29E-154	PA-1
Col3a1	1.10E-157	-1.119	0.973	0.985	2.20E-153	PA-1
Col6a2	5.36E-157	-1.003	0.48	0.72	1.07E-152	PA-1
Slc43a3	1.30E-156	0.774	0.913	0.786	2.60E-152	PA-1
Sept11	1.45E-155	-0.968	0.234	0.534	2.89E-151	PA-1
Lims2	3.78E-153	0.855	0.724	0.466	7.54E-149	PA-1
Vgll3	1.04E-152	0.924	0.766	0.495	2.07E-148	PA-1
Arhgef28	3.47E-152	0.914	0.736	0.473	6.92E-148	PA-1
mt-Atp6	5.42E-152	-0.804	0.933	0.988	1.08E-147	PA-1
mt-Co3	9.14E-152	-0.788	0.933	0.99	1.82E-147	PA-1
Myo1e	1.27E-151	0.880	0.665	0.365	2.54E-147	PA-1
Gsn	5.09E-151	-1.382	0.882	0.953	1.02E-146	PA-1
mt-Nd1	3.27E-150	-0.774	0.876	0.973	6.52E-146	PA-1
Gucy1a2	1.37E-149	-1.083	0.16	0.449	2.73E-145	PA-1
Klf12	9.19E-149	0.947	0.759	0.505	1.83E-144	PA-1
Pdgfd	3.88E-148	0.881	0.879	0.694	7.74E-144	PA-1
Adamtsl1	1.05E-147	0.808	0.952	0.823	2.10E-143	PA-1

Sestd1	2.02E-147	0.867	0.745	0.521	4.04E-143	PA-1
Heg1	3.67E-147	0.663	0.978	0.926	7.33E-143	PA-1
Tspan5	5.03E-147	0.650	0.957	0.89	1.00E-142	PA-1
Trim30d	1.24E-144	1.033	0.489	0.201	2.48E-140	PA-1
Stxbp5	2.49E-144	0.882	0.778	0.57	4.97E-140	PA-1
Fstl1	7.58E-144	0.565	0.981	0.936	1.51E-139	PA-1
Cacna2d1	5.83E-142	-0.881	0.591	0.813	1.16E-137	PA-1
Spsb1	1.09E-140	0.910	0.62	0.339	2.17E-136	PA-1
Gpr1	2.33E-140	0.953	0.296	0.044	4.65E-136	PA-1
Rras2	3.50E-140	0.992	0.559	0.264	6.98E-136	PA-1
Pdzrn3	6.47E-140	-1.080	0.183	0.461	1.29E-135	PA-1
Setbp1	2.30E-139	-0.805	0.775	0.899	4.60E-135	PA-1
Dtna	1.11E-138	1.028	0.308	0.053	2.21E-134	PA-1
mt-Co1	3.15E-138	-0.733	0.944	0.989	6.28E-134	PA-1
mt-Nd4	4.96E-137	-0.744	0.87	0.969	9.90E-133	PA-1
Nfia	7.39E-137	-0.650	0.973	0.99	1.47E-132	PA-1
Csgalnact1	1.98E-135	-1.035	0.301	0.589	3.96E-131	PA-1
Kalrn	3.03E-134	-0.977	0.044	0.251	6.04E-130	PA-1
Boc	4.12E-134	-1.002	0.26	0.536	8.23E-130	PA-1
Rora	5.32E-134	-0.676	0.972	0.986	1.06E-129	PA-1
Psd3	7.41E-134	0.646	0.954	0.836	1.48E-129	PA-1
Inmt	1.43E-133	-1.118	0.077	0.308	2.86E-129	PA-1
Adra1b	6.42E-133	0.918	0.344	0.083	1.28E-128	PA-1
mt-Nd5	3.23E-131	-0.751	0.707	0.898	6.45E-127	PA-1
mt-Cytb	1.33E-129	-0.746	0.891	0.977	2.65E-125	PA-1
Adam33	2.52E-129	0.860	0.721	0.491	5.03E-125	PA-1
mt-Nd2	7.37E-128	-0.705	0.861	0.967	1.47E-123	PA-1
Il18	1.80E-127	0.853	0.418	0.149	3.60E-123	PA-1
mt-Nd4l	1.70E-126	-0.764	0.675	0.875	3.39E-122	PA-1
Antxr1	3.06E-126	0.602	0.921	0.802	6.11E-122	PA-1
Basp1	3.32E-125	0.892	0.483	0.209	6.62E-121	PA-1
Plpp3	3.77E-124	0.806	0.916	0.829	7.52E-120	PA-1
Cp	6.80E-123	-0.946	0.109	0.344	1.36E-118	PA-1
Lama2	1.12E-122	-0.694	0.974	0.976	2.23E-118	PA-1
Tox	1.54E-122	-0.887	0.054	0.258	3.08E-118	PA-1
Chst1	1.75E-122	0.713	0.269	0.043	3.49E-118	PA-1
Thbd	2.04E-122	0.897	0.382	0.126	4.08E-118	PA-1
Tmem100	2.20E-122	0.885	0.46	0.191	4.40E-118	PA-1
Apbb2	2.45E-122	0.601	0.917	0.801	4.89E-118	PA-1
Vcan	1.96E-121	0.768	0.788	0.552	3.91E-117	PA-1
Ppp2r2b	5.40E-121	0.979	0.545	0.257	1.08E-116	PA-1
Plce1	1.17E-120	0.613	0.943	0.842	2.33E-116	PA-1
Adam23	1.35E-120	-1.032	0.214	0.474	2.70E-116	PA-1
Procr	1.51E-120	0.905	0.449	0.188	3.01E-116	PA-1
Tgfbi	8.25E-119	-0.793	0.056	0.254	1.65E-114	PA-1
Vegfc	1.83E-118	-0.985	0.118	0.353	3.66E-114	PA-1
Fmo1	1.98E-117	-1.012	0.169	0.414	3.95E-113	PA-1
Rhobtb1	4.48E-117	-0.720	0.084	0.303	8.94E-113	PA-1
Dcbld2	4.43E-116	0.696	0.792	0.592	8.84E-112	PA-1
Ablim1	2.06E-114	0.554	0.943	0.834	4.11E-110	PA-1
Cyp1b1	2.09E-114	-0.634	0.059	0.259	4.18E-110	PA-1
Prss23	4.77E-114	0.944	0.539	0.298	9.52E-110	PA-1
Smurf1	7.45E-114	0.793	0.675	0.451	1.49E-109	PA-1
Maml3	1.42E-113	-0.784	0.502	0.742	2.83E-109	PA-1
Pde7a	2.74E-113	0.735	0.603	0.334	5.47E-109	PA-1
Itpr1	2.79E-112	-0.828	0.217	0.473	5.56E-108	PA-1
Prr16	2.87E-112	-0.971	0.121	0.354	5.73E-108	PA-1
Eda	4.81E-111	-0.931	0.456	0.711	9.60E-107	PA-1
Fign	8.93E-110	-0.806	0.126	0.361	1.78E-105	PA-1
Fnbp1	1.19E-109	-0.740	0.293	0.556	2.38E-105	PA-1
Rhoq	1.85E-109	0.742	0.692	0.49	3.70E-105	PA-1
Timp2	2.41E-109	0.970	0.754	0.569	4.81E-105	PA-1

Atp10d	1.52E-108	-0.763	0.108	0.324	3.04E-104	PA-1
Dact1	1.90E-108	0.819	0.461	0.214	3.79E-104	PA-1
Cd47	1.16E-107	0.633	0.786	0.662	2.31E-103	PA-1
Nxn	2.41E-107	-0.833	0.067	0.265	4.82E-103	PA-1
Wnt2	1.22E-106	0.810	0.342	0.108	2.43E-102	PA-1
Klf4	1.13E-105	0.809	0.6	0.371	2.25E-101	PA-1
Trps1	1.35E-105	-0.939	0.127	0.348	2.70E-101	PA-1
Iqsec1	8.02E-105	0.762	0.552	0.315	1.60E-100	PA-1
Eya4	1.12E-104	0.964	0.524	0.27	2.23E-100	PA-1
mt-Rnr2	2.91E-104	-0.654	0.869	0.972	5.81E-100	PA-1
Klf2	4.68E-104	0.789	0.414	0.17	9.33E-100	PA-1
Ext1	8.19E-104	0.618	0.976	0.936	1.64E-99	PA-1
Sntg1	8.70E-104	1.577	0.55	0.324	1.74E-99	PA-1
Sema3d	8.80E-104	0.769	0.483	0.214	1.76E-99	PA-1
Nrip1	1.10E-103	-0.751	0.688	0.828	2.20E-99	PA-1
Hivep2	1.40E-102	0.551	0.95	0.892	2.79E-98	PA-1
Plod1	1.79E-102	-0.564	0.064	0.252	3.56E-98	PA-1
Cd34	1.85E-101	0.616	0.792	0.626	3.68E-97	PA-1
Dpt	4.54E-101	0.892	0.766	0.583	9.05E-97	PA-1
Metrnl	5.42E-101	0.747	0.359	0.134	1.08E-96	PA-1
Mycbp2	5.61E-101	-0.602	0.736	0.872	1.12E-96	PA-1
Rab31	7.97E-101	-0.659	0.125	0.344	1.59E-96	PA-1
Rin3	8.67E-101	0.727	0.365	0.131	1.73E-96	PA-1
Nptn	1.24E-100	0.642	0.815	0.686	2.47E-96	PA-1
Cygb	4.66E-100	-0.799	0.151	0.366	9.29E-96	PA-1
Slc24a3	5.57E-100	-1.122	0.186	0.413	1.11E-95	PA-1
Aff3	1.72E-99	-0.841	0.338	0.571	3.43E-95	PA-1
Wnt5b	1.20E-98	0.887	0.301	0.088	2.39E-94	PA-1
Pcolce	2.94E-98	-0.727	0.269	0.512	5.87E-94	PA-1
Raph1	3.36E-98	-0.713	0.403	0.631	6.71E-94	PA-1
Abca5	4.06E-98	0.745	0.448	0.211	8.10E-94	PA-1
Fgfr1	2.84E-97	0.594	0.829	0.7	5.66E-93	PA-1
Pamr1	3.93E-97	0.726	0.639	0.411	7.85E-93	PA-1
Tbc1d12	4.56E-97	0.719	0.667	0.464	9.11E-93	PA-1
Ifi207	1.10E-96	-0.723	0.527	0.727	2.20E-92	PA-1
Nalcn	1.61E-96	-0.890	0.097	0.295	3.21E-92	PA-1
Ghr	2.29E-96	-0.851	0.835	0.916	4.57E-92	PA-1
Glce	2.53E-96	0.805	0.535	0.306	5.05E-92	PA-1
Arhgap42	3.57E-95	0.616	0.892	0.794	7.13E-91	PA-1
Dbn1	9.70E-95	0.718	0.492	0.264	1.94E-90	PA-1
Lrrfip1	3.32E-94	-0.614	0.121	0.332	6.64E-90	PA-1
Zeb1	3.77E-94	0.513	0.97	0.935	7.53E-90	PA-1
Man1c1	2.87E-93	-0.672	0.269	0.505	5.74E-89	PA-1
Prkg2	4.91E-93	0.767	0.324	0.108	9.80E-89	PA-1
Arhgap6	8.98E-93	-0.579	0.904	0.958	1.79E-88	PA-1
Map4k4	1.10E-92	-0.578	0.665	0.829	2.20E-88	PA-1
Pcdh7	2.92E-92	-0.705	0.787	0.894	5.83E-88	PA-1
Pitpnc1	1.68E-90	-0.744	0.309	0.543	3.35E-86	PA-1
Map3k5	2.92E-90	-0.690	0.369	0.592	5.83E-86	PA-1
Prex2	6.07E-90	-0.776	0.362	0.597	1.21E-85	PA-1
Fam78b	4.15E-89	-0.612	0.089	0.277	8.28E-85	PA-1
Bach2	4.15E-89	0.709	0.646	0.438	8.29E-85	PA-1
Flrt2	8.07E-89	0.693	0.878	0.775	1.61E-84	PA-1
Disp1	1.57E-88	0.688	0.591	0.369	3.14E-84	PA-1
Clip4	1.99E-88	0.676	0.395	0.172	3.98E-84	PA-1
Sh3kbp1	3.54E-88	-0.673	0.223	0.443	7.07E-84	PA-1
Abca8a	4.16E-88	-0.757	0.606	0.791	8.31E-84	PA-1
Gria4	4.23E-88	-1.990	0.078	0.251	8.44E-84	PA-1
Gxylt2	4.38E-88	-0.713	0.231	0.461	8.75E-84	PA-1
Pdgfrb	6.34E-88	-0.693	0.199	0.411	1.27E-83	PA-1
Qpct	1.79E-87	0.661	0.307	0.105	3.57E-83	PA-1
Rreb1	4.50E-87	-0.687	0.327	0.565	8.97E-83	PA-1

Igfbp6	4.91E-87	0.913	0.667	0.457	9.80E-83	PA-1
Wdr66	6.28E-87	-0.635	0.088	0.272	1.25E-82	PA-1
Kcnk5	3.44E-86	0.646	0.252	0.064	6.86E-82	PA-1
Cd248	6.40E-86	0.844	0.56	0.354	1.28E-81	PA-1
Col6a1	8.96E-86	-0.734	0.422	0.63	1.79E-81	PA-1
Fhl1	9.17E-86	0.765	0.48	0.265	1.83E-81	PA-1
Entpd2	1.14E-85	-0.701	0.219	0.427	2.28E-81	PA-1
Prickle2	8.49E-85	-0.779	0.261	0.482	1.70E-80	PA-1
Jazf1	2.34E-84	-0.764	0.157	0.361	4.67E-80	PA-1
Daglb	2.54E-84	0.845	0.433	0.221	5.06E-80	PA-1
Pid1	3.04E-84	-0.931	0.714	0.827	6.07E-80	PA-1
Pten	9.42E-84	-0.769	0.556	0.717	1.88E-79	PA-1
Col1a1	1.10E-83	-1.079	0.729	0.847	2.20E-79	PA-1
Tep1	3.19E-83	-0.534	0.101	0.284	6.37E-79	PA-1
Abi3bp	4.94E-83	-0.775	0.807	0.875	9.86E-79	PA-1
Rad51b	5.80E-83	-0.726	0.531	0.733	1.16E-78	PA-1
Ppp3ca	2.29E-82	-0.562	0.744	0.876	4.57E-78	PA-1
Cfh	2.61E-82	-0.903	0.164	0.363	5.21E-78	PA-1
Rhoa	1.14E-81	0.541	0.718	0.506	2.28E-77	PA-1
Mtus1	2.05E-81	-0.625	0.22	0.438	4.09E-77	PA-1
Gnb4	2.19E-81	-0.524	0.084	0.259	4.38E-77	PA-1
Mbnl1	7.76E-81	-0.607	0.801	0.877	1.55E-76	PA-1
Myh9	2.02E-80	-0.624	0.167	0.367	4.04E-76	PA-1
Sh3rf3	2.04E-80	-0.661	0.208	0.426	4.07E-76	PA-1
Edn1	6.99E-80	0.715	0.282	0.092	1.40E-75	PA-1
Cacna1a	7.35E-80	-0.657	0.402	0.621	1.47E-75	PA-1
Il1rl2	3.06E-79	0.650	0.751	0.581	6.12E-75	PA-1
Jpt1	4.27E-79	0.625	0.412	0.201	8.53E-75	PA-1
Cyth3	4.51E-79	-0.769	0.509	0.695	9.00E-75	PA-1
Il11ra1	9.33E-79	-0.605	0.13	0.314	1.86E-74	PA-1
Dock11	1.07E-78	0.600	0.741	0.576	2.14E-74	PA-1
Fbxl7	1.15E-78	0.652	0.863	0.755	2.30E-74	PA-1
Myof	2.08E-78	0.548	0.803	0.66	4.15E-74	PA-1
Zyx	6.70E-78	0.620	0.359	0.158	1.34E-73	PA-1
Islr2	3.98E-77	0.584	0.283	0.097	7.95E-73	PA-1
Colec12	7.89E-77	-0.739	0.348	0.562	1.57E-72	PA-1
Ptgis	1.28E-76	0.591	0.386	0.177	2.56E-72	PA-1
Phlpp1	1.90E-76	0.608	0.769	0.617	3.79E-72	PA-1
Slc41a2	2.51E-76	-0.556	0.222	0.44	5.01E-72	PA-1
Tcf4	6.18E-76	-0.640	0.869	0.912	1.23E-71	PA-1
Fez1	2.64E-75	0.570	0.287	0.101	5.27E-71	PA-1
Lrp6	3.29E-75	-0.581	0.641	0.783	6.56E-71	PA-1
Plcl1	5.83E-74	-0.663	0.525	0.72	1.16E-69	PA-1
Pcdh9	8.08E-74	0.940	0.614	0.417	1.61E-69	PA-1
Chst11	1.09E-73	-0.594	0.095	0.265	2.17E-69	PA-1
C7	2.26E-73	-1.803	0.394	0.553	4.52E-69	PA-1
Notch2	4.12E-73	0.593	0.628	0.453	8.22E-69	PA-1
Slc7a6	4.34E-73	0.644	0.515	0.315	8.66E-69	PA-1
Gnpnat1	5.05E-73	0.554	0.285	0.102	1.01E-68	PA-1
Bcl2	1.18E-72	-0.708	0.22	0.418	2.36E-68	PA-1
Fam20a	1.59E-72	0.620	0.608	0.411	3.16E-68	PA-1
Atp2b1	1.84E-72	0.506	0.779	0.617	3.66E-68	PA-1
Mtcl1	2.56E-72	0.586	0.267	0.091	5.10E-68	PA-1
Emp1	2.91E-72	-0.651	0.249	0.447	5.81E-68	PA-1
Vit	4.96E-72	-0.682	0.126	0.306	9.89E-68	PA-1
Asap2	1.45E-71	0.560	0.688	0.514	2.90E-67	PA-1
Sh3pxd2a	1.75E-71	-0.518	0.126	0.308	3.48E-67	PA-1
Egfr	1.43E-70	-0.561	0.966	0.972	2.85E-66	PA-1
Apbb1ip	3.48E-70	-0.620	0.634	0.78	6.95E-66	PA-1
Gm44080	5.53E-70	0.619	0.295	0.114	1.10E-65	PA-1
Aig1	1.16E-69	0.652	0.601	0.41	2.32E-65	PA-1
mt-Nd6	1.49E-69	-0.598	0.349	0.563	2.97E-65	PA-1

Dock9	3.43E-69	-0.706	0.395	0.594	6.85E-65	PA-1
Sema5a	3.85E-69	-0.738	0.134	0.309	7.68E-65	PA-1
Cers6	3.80E-68	0.592	0.615	0.438	7.59E-64	PA-1
Atp6v0a1	8.49E-68	-0.566	0.227	0.419	1.69E-63	PA-1
St5	1.59E-67	-0.607	0.382	0.582	3.17E-63	PA-1
Cask	1.82E-67	0.553	0.802	0.692	3.63E-63	PA-1
Cadm3	3.39E-67	0.664	0.419	0.231	6.77E-63	PA-1
Ism1	1.09E-66	-0.950	0.099	0.257	2.18E-62	PA-1
Adh1	1.40E-66	-0.660	0.106	0.266	2.80E-62	PA-1
Lmx1a	2.31E-66	0.647	0.376	0.184	4.61E-62	PA-1
Galnt17	4.25E-66	-0.667	0.473	0.663	8.48E-62	PA-1
Gpm6b	6.56E-66	-0.778	0.204	0.392	1.31E-61	PA-1
C3	6.80E-66	0.802	0.637	0.445	1.36E-61	PA-1
Arhgap32	6.81E-66	-0.544	0.419	0.619	1.36E-61	PA-1
Adamtsl4	7.14E-66	0.585	0.369	0.185	1.43E-61	PA-1
Thrb	8.17E-66	-0.625	0.49	0.672	1.63E-61	PA-1
Irak3	2.01E-65	0.571	0.629	0.454	4.01E-61	PA-1
Scara3	4.35E-65	0.595	0.478	0.293	8.69E-61	PA-1
Cpne8	1.89E-64	0.549	0.74	0.568	3.77E-60	PA-1
1700019D03Ri	2.30E-64	0.594	0.341	0.16	4.59E-60	PA-1
Slc9a9	2.56E-64	-0.632	0.455	0.651	5.11E-60	PA-1
Lvrn	8.35E-64	0.699	0.635	0.45	1.67E-59	PA-1
Il6ra	1.27E-63	0.601	0.572	0.39	2.54E-59	PA-1
Lrrn4cl	3.60E-63	0.540	0.287	0.117	7.20E-59	PA-1
Srpx	6.91E-63	-0.537	0.106	0.261	1.38E-58	PA-1
Atxn1	1.33E-62	-0.605	0.525	0.689	2.66E-58	PA-1
Mylip	2.88E-62	0.565	0.29	0.12	5.75E-58	PA-1
Tbx15	3.28E-62	-0.553	0.64	0.785	6.55E-58	PA-1
Zdhhc14	5.65E-62	-0.604	0.238	0.423	1.13E-57	PA-1
Ugp2	1.12E-61	0.610	0.684	0.54	2.24E-57	PA-1
Arhgap18	1.24E-61	-0.579	0.312	0.512	2.47E-57	PA-1
Pik3r1	2.20E-61	-0.543	0.643	0.782	4.39E-57	PA-1
mt-Nd3	2.20E-61	-0.538	0.42	0.62	4.39E-57	PA-1
Igfbp4	5.21E-61	0.627	0.516	0.334	1.04E-56	PA-1
Sned1	1.02E-60	-0.853	0.254	0.433	2.04E-56	PA-1
St3gal4	6.16E-60	-0.534	0.235	0.421	1.23E-55	PA-1
Megf9	6.80E-60	0.558	0.57	0.391	1.36E-55	PA-1
Jag1	7.21E-60	0.669	0.467	0.294	1.44E-55	PA-1
Slc16a9	2.43E-59	0.688	0.402	0.228	4.86E-55	PA-1
Rpl13	3.56E-59	1.278	0.269	0.11	7.11E-55	PA-1
Ly6c1	3.89E-59	0.597	0.5	0.314	7.76E-55	PA-1
Itga11	7.78E-59	0.574	0.571	0.389	1.55E-54	PA-1
Snrk	7.82E-59	-0.581	0.203	0.377	1.56E-54	PA-1
Dennd4a	3.12E-58	-0.738	0.39	0.564	6.22E-54	PA-1
Phldb2	5.43E-58	-0.619	0.378	0.56	1.08E-53	PA-1
Abca6	7.27E-58	0.550	0.635	0.473	1.45E-53	PA-1
Adam22	7.60E-58	0.629	0.398	0.216	1.52E-53	PA-1
Fam13c	7.89E-58	0.567	0.49	0.31	1.57E-53	PA-1
Adcy2	1.15E-57	0.629	0.488	0.304	2.30E-53	PA-1
Dab2	1.54E-57	-0.608	0.224	0.401	3.07E-53	PA-1
Lrrc8b	1.65E-57	0.533	0.271	0.111	3.29E-53	PA-1
Micu1	2.87E-57	0.545	0.622	0.462	5.72E-53	PA-1
Arl4a	4.11E-57	0.571	0.483	0.312	8.21E-53	PA-1
Elmo1	5.14E-57	-0.563	0.632	0.76	1.03E-52	PA-1
Gm33869	6.70E-57	0.533	0.515	0.334	1.34E-52	PA-1
Snx21	8.33E-57	0.569	0.346	0.177	1.66E-52	PA-1
Ly6a	1.06E-56	0.627	0.656	0.493	2.11E-52	PA-1
Msi2	1.77E-56	0.541	0.635	0.482	3.54E-52	PA-1
Col1a2	3.40E-56	-0.676	0.934	0.956	6.79E-52	PA-1
Cdh13	1.60E-55	-0.818	0.385	0.549	3.19E-51	PA-1
Adgrg6	5.69E-55	0.622	0.26	0.108	1.14E-50	PA-1
Plat	1.73E-54	0.597	0.469	0.307	3.46E-50	PA-1

Rapgef1	7.32E-54	0.504	0.636	0.499	1.46E-49	PA-1
Rps15	2.24E-53	0.907	0.347	0.186	4.48E-49	PA-1
Col12a1	3.67E-53	0.669	0.402	0.231	7.33E-49	PA-1
Kctd9	7.65E-53	0.537	0.491	0.322	1.53E-48	PA-1
Cdh9	1.57E-52	0.641	0.437	0.266	3.14E-48	PA-1
Gas1	1.93E-52	-0.794	0.231	0.386	3.85E-48	PA-1
Bmp1	2.50E-52	0.510	0.66	0.539	4.99E-48	PA-1
Nckap5	4.09E-51	-0.586	0.509	0.674	8.16E-47	PA-1
Rps8	6.78E-51	1.076	0.272	0.124	1.35E-46	PA-1
Nipal2	1.18E-50	0.583	0.423	0.251	2.35E-46	PA-1
Eef1a1	1.72E-50	1.110	0.273	0.125	3.43E-46	PA-1
Plac8	1.24E-49	0.603	0.277	0.128	2.48E-45	PA-1
Chrdl1	3.94E-49	-0.897	0.154	0.3	7.87E-45	PA-1
Tlr4	6.90E-49	0.564	0.567	0.42	1.38E-44	PA-1
Epb41l3	2.83E-48	0.534	0.473	0.31	5.65E-44	PA-1
Itga2	5.12E-48	0.550	0.265	0.123	1.02E-43	PA-1
Dlg2	8.35E-48	0.664	0.368	0.208	1.67E-43	PA-1
Irs1	8.80E-48	-0.578	0.382	0.549	1.76E-43	PA-1
Ogn	9.28E-48	0.583	0.665	0.551	1.85E-43	PA-1
Nfkbiz	1.12E-47	0.561	0.266	0.123	2.24E-43	PA-1
Bicd1	1.47E-47	0.550	0.466	0.302	2.94E-43	PA-1
Magi1	2.30E-46	-0.678	0.347	0.515	4.58E-42	PA-1
Man2a1	1.44E-45	-0.536	0.785	0.853	2.88E-41	PA-1
Glp2r	1.66E-44	0.616	0.295	0.151	3.32E-40	PA-1
Rab38	2.32E-44	0.502	0.273	0.131	4.63E-40	PA-1
S100a11	1.16E-43	0.609	0.302	0.16	2.31E-39	PA-1
St6gal1	1.55E-43	0.530	0.409	0.258	3.10E-39	PA-1
Kitl	1.78E-43	-0.540	0.26	0.418	3.56E-39	PA-1
Klf3	8.35E-43	0.514	0.555	0.426	1.67E-38	PA-1
Klhl29	1.30E-42	0.501	0.649	0.515	2.60E-38	PA-1
Aldh1a1	1.75E-42	-0.623	0.742	0.815	3.50E-38	PA-1
Arid5b	7.21E-42	-0.510	0.217	0.366	1.44E-37	PA-1
Xkr4	1.83E-41	-0.673	0.239	0.389	3.65E-37	PA-1
Ccdc80	8.81E-41	-0.545	0.617	0.743	1.76E-36	PA-1
Ptprk	3.91E-40	-0.562	0.191	0.334	7.81E-36	PA-1
Adam19	4.34E-40	-0.524	0.436	0.575	8.67E-36	PA-1
Fgf7	1.58E-39	-0.524	0.249	0.402	3.16E-35	PA-1
Vegfd	1.29E-38	0.536	0.428	0.292	2.58E-34	PA-1
Hsp90ab1	2.27E-38	0.644	0.268	0.14	4.52E-34	PA-1
Ppp1r9a	3.50E-38	0.538	0.411	0.267	6.98E-34	PA-1
Kcnt2	2.17E-37	-0.736	0.444	0.56	4.33E-33	PA-1
Parp8	1.12E-36	0.538	0.657	0.565	2.24E-32	PA-1
Il1r2	4.68E-36	0.635	0.26	0.139	9.33E-32	PA-1
Frmd4a	1.27E-35	-0.511	0.265	0.404	2.53E-31	PA-1
Plcb1	2.02E-33	-0.585	0.191	0.317	4.03E-29	PA-1
Rps21	6.80E-33	0.698	0.393	0.27	1.36E-28	PA-1
Selenop	1.31E-32	-0.522	0.151	0.265	2.60E-28	PA-1
Fth1	3.18E-31	0.687	0.25	0.136	6.34E-27	PA-1
Rplp1	4.09E-29	0.854	0.266	0.157	8.17E-25	PA-1
Tmtc2	7.14E-29	0.698	0.356	0.241	1.42E-24	PA-1
Fbln1	1.33E-27	-0.518	0.237	0.357	2.65E-23	PA-1
Rnf220	2.62E-27	0.645	0.304	0.2	5.22E-23	PA-1
Epha3	2.40E-20	-0.590	0.314	0.41	4.80E-16	PA-1
Sult1e1	1.13E-13	-0.528	0.197	0.271	2.25E-09	PA-1
Adam12.1	0	2.292	0.572	0.124	0	PA-2
Col4a1.1	0	1.810	0.858	0.359	0	PA-2
Bmper.1	8.05E-295	1.876	0.755	0.281	1.61E-290	PA-2
Col4a2.1	7.93E-286	1.625	0.756	0.282	1.58E-281	PA-2
Creb5.1	7.49E-260	-1.572	0.768	0.933	1.49E-255	PA-2
Col6a3.1	6.41E-257	1.523	0.842	0.474	1.28E-252	PA-2
Pparg.1	6.73E-257	1.390	0.448	0.076	1.34E-252	PA-2
Col5a3.1	8.98E-248	1.537	0.792	0.405	1.79E-243	PA-2

Pde11a.1	2.19E-244	2.160	0.471	0.098	4.37E-240	PA-2
Pi16.1	2.17E-242	-1.940	0.4	0.815	4.33E-238	PA-2
Sema3c.1	1.23E-240	-1.544	0.674	0.921	2.46E-236	PA-2
Anxa3.1	3.10E-240	-1.534	0.604	0.873	6.18E-236	PA-2
Col15a1.1	1.41E-238	1.401	0.492	0.108	2.81E-234	PA-2
Sema3e.1	1.16E-237	-2.267	0.194	0.676	2.31E-233	PA-2
Limch1.1	3.08E-230	-1.782	0.297	0.737	6.14E-226	PA-2
Gria3.1	4.21E-227	1.429	0.718	0.279	8.40E-223	PA-2
Dpp4.1	9.36E-227	-1.605	0.561	0.849	1.87E-222	PA-2
Enpp2.1	1.84E-221	1.687	0.474	0.114	3.68E-217	PA-2
Nav3.1	2.30E-204	-1.847	0.724	0.866	4.60E-200	PA-2
Hmcn2.1	4.01E-194	1.493	0.623	0.228	8.00E-190	PA-2
Gfpt2.1	5.14E-189	-1.383	0.537	0.838	1.03E-184	PA-2
Igfbp5.1	9.18E-189	-2.118	0.305	0.706	1.83E-184	PA-2
Auts2.1	3.62E-188	1.229	0.902	0.626	7.23E-184	PA-2
Ldb2.1	4.11E-179	1.197	0.651	0.257	8.20E-175	PA-2
Fam49a.1	3.74E-177	1.182	0.566	0.205	7.47E-173	PA-2
Opcml.1	2.16E-175	-1.077	0.717	0.949	4.30E-171	PA-2
Smoc2.1	1.47E-173	1.354	0.615	0.26	2.93E-169	PA-2
Cd55.1	7.82E-169	-1.458	0.441	0.746	1.56E-164	PA-2
Sparcl1.1	1.41E-168	0.990	0.383	0.086	2.81E-164	PA-2
Rora.1	5.32E-164	0.830	0.996	0.973	1.06E-159	PA-2
Mme.1	7.97E-163	1.100	0.369	0.084	1.59E-158	PA-2
Ston2.1	1.77E-162	-1.436	0.311	0.678	3.53E-158	PA-2
Efemp1.1	2.86E-162	-1.332	0.533	0.801	5.70E-158	PA-2
Cmah.1	1.17E-161	-1.506	0.317	0.682	2.33E-157	PA-2
Slco3a1.1	5.41E-160	-1.054	0.62	0.873	1.08E-155	PA-2
Nrep.1	5.74E-159	1.209	0.521	0.189	1.14E-154	PA-2
Frmd4b.1	2.40E-155	1.072	0.832	0.527	4.79E-151	PA-2
Csgalnact1.1	2.74E-154	1.226	0.692	0.342	5.47E-150	PA-2
Lpl.1	1.21E-153	1.247	0.378	0.092	2.42E-149	PA-2
Fbn1.1	6.49E-151	-1.065	0.954	0.981	1.29E-146	PA-2
Gask1b.1	7.03E-151	1.116	0.546	0.208	1.40E-146	PA-2
Efna5.1	1.55E-148	-1.641	0.343	0.668	3.10E-144	PA-2
Scara5.1	4.81E-148	-0.937	0.649	0.893	9.60E-144	PA-2
Bgn.1	1.08E-147	1.042	0.621	0.259	2.15E-143	PA-2
Col6a2.1	5.55E-146	1.049	0.786	0.519	1.11E-141	PA-2
Fgf10	5.28E-145	1.177	0.341	0.079	1.05E-140	PA-2
Nfia.1	2.86E-143	0.752	0.994	0.976	5.70E-139	PA-2
Efhd1.1	4.60E-139	-1.349	0.169	0.529	9.19E-135	PA-2
Cacna2d1.1	6.46E-137	0.989	0.861	0.631	1.29E-132	PA-2
Adam23.1	1.82E-136	1.263	0.564	0.252	3.63E-132	PA-2
Sgip1.1	1.25E-135	1.297	0.459	0.157	2.50E-131	PA-2
Vav3.1	9.08E-135	0.885	0.517	0.184	1.81E-130	PA-2
Negr1.1	3.16E-134	-1.611	0.302	0.629	6.32E-130	PA-2
Kank1.1	2.28E-133	-1.314	0.143	0.501	4.54E-129	PA-2
Col27a1	2.48E-133	0.845	0.316	0.073	4.95E-129	PA-2
Eps8.1	1.32E-130	1.041	0.657	0.332	2.63E-126	PA-2
Fn1.1	6.16E-130	-0.874	0.723	0.93	1.23E-125	PA-2
Car8.1	2.19E-129	-1.384	0.105	0.452	4.37E-125	PA-2
Cped1.1	1.71E-128	0.719	0.976	0.907	3.41E-124	PA-2
Lama2.1	6.82E-128	0.759	0.993	0.97	1.36E-123	PA-2
Adamts12.1	8.04E-128	1.094	0.932	0.817	1.61E-123	PA-2
Igfbp7.1	1.20E-127	0.820	0.699	0.356	2.39E-123	PA-2
Ildr2.1	1.99E-127	-1.260	0.211	0.556	3.97E-123	PA-2
Lurap1l.1	2.52E-126	-1.248	0.274	0.609	5.03E-122	PA-2
Dact2.1	1.90E-124	-1.510	0.05	0.378	3.79E-120	PA-2
Ano3.1	2.37E-124	-1.274	0.208	0.566	4.74E-120	PA-2
Col3a1.1	4.15E-124	0.942	0.992	0.974	8.29E-120	PA-2
Slc24a3.1	4.20E-124	1.389	0.506	0.215	8.37E-120	PA-2
Gucy1a2.1	4.37E-123	1.100	0.515	0.212	8.72E-119	PA-2
Gan.1	7.01E-123	-1.550	0.223	0.539	1.40E-118	PA-2

Fndc1.1	1.13E-121	-0.803	0.831	0.942	2.25E-117	PA-2
Nfib.1	1.38E-121	0.607	0.983	0.919	2.76E-117	PA-2
Svep1.1	3.63E-121	0.995	0.812	0.553	7.25E-117	PA-2
Eya2.1	6.72E-121	0.975	0.505	0.202	1.34E-116	PA-2
Adamts16.1	2.10E-120	-1.583	0.054	0.382	4.18E-116	PA-2
Tek.1	1.00E-118	-1.171	0.463	0.715	2.00E-114	PA-2
Aldh1a3.1	1.36E-118	-1.319	0.106	0.428	2.72E-114	PA-2
Ank2.1	1.32E-117	0.904	0.873	0.628	2.64E-113	PA-2
Samhd1.1	1.78E-117	-0.997	0.516	0.761	3.55E-113	PA-2
Deptor.1	5.52E-115	1.004	0.546	0.247	1.10E-110	PA-2
Ntn4.1	4.32E-114	-1.185	0.241	0.559	8.63E-110	PA-2
Zfp462.1	5.89E-114	-1.081	0.416	0.705	1.18E-109	PA-2
Ror1.1	3.49E-113	0.904	0.794	0.515	6.97E-109	PA-2
Adgrg2.1	1.68E-112	-1.138	0.201	0.528	3.35E-108	PA-2
Pcolce2.1	1.64E-111	-1.019	0.462	0.726	3.27E-107	PA-2
Sema5a.1	5.28E-111	0.983	0.41	0.148	1.05E-106	PA-2
Agmo.1	5.49E-111	0.827	0.894	0.666	1.09E-106	PA-2
Palld.1	1.41E-110	0.894	0.352	0.107	2.81E-106	PA-2
Dock10.1	1.45E-106	0.866	0.64	0.334	2.89E-102	PA-2
Smpd3.1	3.47E-106	-1.195	0.304	0.625	6.92E-102	PA-2
Plcl2.1	1.24E-105	0.811	0.41	0.147	2.48E-101	PA-2
Sept11.1	1.29E-103	0.898	0.573	0.295	2.57E-99	PA-2
Pde8a.1	8.16E-103	-0.913	0.562	0.777	1.63E-98	PA-2
Ggt5	2.81E-102	0.682	0.251	0.058	5.62E-98	PA-2
Lum.1	4.13E-102	0.762	0.474	0.196	8.24E-98	PA-2
Adgrd1.1	4.21E-101	-0.700	0.817	0.929	8.41E-97	PA-2
Acvr2a.1	4.23E-101	0.925	0.689	0.418	8.43E-97	PA-2
Pdgfc.1	4.63E-101	-1.201	0.24	0.54	9.24E-97	PA-2
Plpp1.1	6.69E-101	0.887	0.523	0.24	1.33E-96	PA-2
Ptprj.1	1.10E-98	-0.927	0.566	0.789	2.20E-94	PA-2
Setbp1.1	1.81E-98	0.739	0.923	0.798	3.61E-94	PA-2
Smurf2.1	3.06E-98	-0.873	0.8	0.887	6.10E-94	PA-2
Fap	4.11E-98	0.847	0.876	0.731	8.20E-94	PA-2
Rgs6.1	1.26E-97	-1.555	0.165	0.462	2.51E-93	PA-2
Dock4.1	2.22E-97	-0.884	0.756	0.883	4.42E-93	PA-2
Sbsn.1	3.00E-97	-1.325	0.052	0.327	6.00E-93	PA-2
Axl.1	4.08E-97	-0.820	0.621	0.806	8.14E-93	PA-2
Arhgap24.1	4.16E-97	0.721	0.978	0.882	8.31E-93	PA-2
Sema3d.1	8.53E-96	-1.049	0.133	0.44	1.70E-91	PA-2
Eda.1	3.97E-95	1.024	0.756	0.505	7.93E-91	PA-2
Gli2.1	6.28E-95	0.617	0.354	0.117	1.25E-90	PA-2
Ddr2.1	8.46E-95	-0.631	0.908	0.955	1.69E-90	PA-2
Uap1.1	3.59E-93	-0.995	0.463	0.7	7.16E-89	PA-2
Hdac7.1	2.33E-92	-0.821	0.695	0.814	4.64E-88	PA-2
Sdk1.1	7.89E-92	-0.663	0.853	0.932	1.58E-87	PA-2
Rorb.1	9.42E-92	-1.218	0.244	0.537	1.88E-87	PA-2
Gas7.1	7.34E-91	-0.730	0.738	0.896	1.47E-86	PA-2
Sulf1.1	9.98E-91	-0.751	0.74	0.887	1.99E-86	PA-2
Nrp1.1	1.91E-90	0.757	0.705	0.418	3.82E-86	PA-2
Pcdh7.1	7.13E-89	0.756	0.93	0.803	1.42E-84	PA-2
Pdgfd.1	2.14E-88	-0.886	0.665	0.842	4.27E-84	PA-2
Nr1h4.1	3.16E-87	-1.138	0.187	0.461	6.31E-83	PA-2
Nhsl1.1	3.94E-87	-0.837	0.589	0.781	7.85E-83	PA-2
Arhgap6.1	3.11E-85	0.652	0.973	0.913	6.21E-81	PA-2
Pcolce.1	9.14E-84	0.754	0.572	0.311	1.82E-79	PA-2
Tox.1	6.36E-83	0.793	0.293	0.093	1.27E-78	PA-2
Bmp6.1	1.84E-82	-1.276	0.129	0.395	3.66E-78	PA-2
Galnt16.1	2.68E-82	-0.855	0.477	0.7	5.35E-78	PA-2
Ackr3.1	3.90E-82	-0.910	0.464	0.69	7.78E-78	PA-2
Fyn.1	4.64E-82	-0.602	0.786	0.892	9.27E-78	PA-2
Myo5a.1	9.47E-82	0.571	0.452	0.194	1.89E-77	PA-2
Col5a2	1.04E-81	0.609	0.968	0.933	2.07E-77	PA-2

Arhgef28.1	4.05E-81	-0.830	0.436	0.683	8.08E-77	PA-2
Ugdh.1	8.00E-81	-0.975	0.374	0.621	1.60E-76	PA-2
Fgf2.1	2.07E-80	-0.869	0.321	0.585	4.14E-76	PA-2
Tcf4.1	2.70E-80	0.716	0.937	0.872	5.38E-76	PA-2
Mrc2	2.88E-80	0.700	0.692	0.461	5.76E-76	PA-2
Klf12.1	2.43E-79	-0.873	0.482	0.704	4.85E-75	PA-2
Adam33.1	1.58E-78	-0.831	0.446	0.677	3.15E-74	PA-2
Ablim1.1	7.67E-78	-0.619	0.807	0.924	1.53E-73	PA-2
Kif2a.1	1.43E-77	-0.802	0.49	0.695	2.85E-73	PA-2
Flnb.1	2.33E-77	-0.727	0.458	0.691	4.65E-73	PA-2
Dclk1	2.66E-77	0.761	0.822	0.636	5.31E-73	PA-2
Abca8a.1	1.38E-76	0.763	0.834	0.639	2.75E-72	PA-2
Slc43a3.1	4.27E-76	-0.692	0.786	0.882	8.53E-72	PA-2
Cyp1b1.1	5.11E-76	0.603	0.29	0.099	1.02E-71	PA-2
Pla1a.1	6.59E-76	-0.865	0.441	0.648	1.32E-71	PA-2
Mfap5.1	9.37E-76	-0.820	0.735	0.845	1.87E-71	PA-2
Slc4a4.1	1.44E-75	-0.646	0.749	0.886	2.88E-71	PA-2
Itgbl1.1	7.89E-75	0.765	0.853	0.66	1.57E-70	PA-2
Kalrn.1	1.93E-74	0.733	0.27	0.089	3.84E-70	PA-2
Dhrs3.1	1.29E-73	0.668	0.364	0.153	2.57E-69	PA-2
Emilin2.1	1.53E-73	-0.797	0.393	0.62	3.05E-69	PA-2
Egfr.1	2.65E-73	0.605	0.986	0.964	5.28E-69	PA-2
Frmd6.1	1.31E-71	0.650	0.847	0.674	2.62E-67	PA-2
Timp2.1	1.53E-71	-0.955	0.53	0.72	3.05E-67	PA-2
Anxa1.1	1.91E-71	-0.727	0.464	0.686	3.80E-67	PA-2
Adam22.1	3.08E-70	-0.884	0.13	0.378	6.14E-66	PA-2
Rhoj	5.46E-70	0.737	0.917	0.842	1.09E-65	PA-2
Mgll.1	1.13E-69	-0.788	0.216	0.466	2.26E-65	PA-2
Tspan5.1	1.37E-69	-0.564	0.892	0.94	2.72E-65	PA-2
Stxbp6.1	1.40E-69	-0.776	0.531	0.744	2.80E-65	PA-2
Reps2.1	1.63E-69	0.685	0.511	0.271	3.26E-65	PA-2
Marcks.1	2.06E-69	-0.894	0.428	0.646	4.12E-65	PA-2
Jazf1.1	2.76E-69	0.797	0.408	0.193	5.51E-65	PA-2
Plce1.1	8.52E-69	-0.576	0.822	0.924	1.70E-64	PA-2
Prickle2.1	1.01E-68	0.826	0.527	0.302	2.03E-64	PA-2
Gpc6.1	2.66E-68	-0.566	0.973	0.98	5.32E-64	PA-2
Prkd1.1	2.94E-68	0.657	0.735	0.489	5.87E-64	PA-2
Pten.1	3.41E-68	0.804	0.749	0.586	6.80E-64	PA-2
mt-Co3.1	2.43E-67	0.600	0.987	0.947	4.85E-63	PA-2
Col6a1.1	6.79E-67	0.707	0.674	0.46	1.35E-62	PA-2
Rasa3.1	7.78E-67	0.696	0.38	0.173	1.55E-62	PA-2
mt-Nd5.1	1.46E-66	0.599	0.909	0.75	2.91E-62	PA-2
Sestd1.1	2.84E-66	-0.728	0.52	0.691	5.67E-62	PA-2
Prkca.1	3.10E-66	-0.555	0.861	0.926	6.19E-62	PA-2
Tgfbr2.1	5.72E-65	-0.639	0.668	0.803	1.14E-60	PA-2
Thbd.1	6.51E-65	-0.824	0.104	0.327	1.30E-60	PA-2
Sntg1.1	7.90E-64	-1.695	0.29	0.505	1.58E-59	PA-2
Clip1.1	8.32E-64	-0.656	0.61	0.759	1.66E-59	PA-2
Maml3.1	1.84E-63	0.632	0.759	0.555	3.67E-59	PA-2
Lifr.1	2.04E-63	0.554	0.264	0.094	4.07E-59	PA-2
Tmem100.1	3.34E-63	-0.806	0.171	0.401	6.66E-59	PA-2
Pcsk6.1	3.45E-63	-0.835	0.45	0.641	6.89E-59	PA-2
Dact1.1	4.42E-63	-0.791	0.183	0.41	8.82E-59	PA-2
Mmp3.1	6.73E-63	0.663	0.367	0.162	1.34E-58	PA-2
Lims2.1	6.97E-63	-0.680	0.464	0.662	1.39E-58	PA-2
Pdzrn3.1	1.07E-62	0.907	0.46	0.25	2.13E-58	PA-2
Sytl2.1	1.32E-62	-0.793	0.105	0.325	2.64E-58	PA-2
Slco2b1.1	1.47E-62	0.616	0.398	0.185	2.94E-58	PA-2
Adamtsl1.1	2.38E-62	-0.700	0.833	0.918	4.75E-58	PA-2
Plcl1.1	4.58E-62	0.703	0.76	0.561	9.14E-58	PA-2
Calcrl.1	1.68E-61	-1.043	0.322	0.537	3.35E-57	PA-2
Myo1e.1	1.95E-61	-0.755	0.37	0.591	3.89E-57	PA-2

mt-Nd1.1	9.96E-61	0.546	0.97	0.901	1.99E-56	PA-2
Trim30d.1	2.02E-60	-0.855	0.201	0.42	4.03E-56	PA-2
Errfi1	3.03E-60	-0.712	0.528	0.726	6.06E-56	PA-2
Igfbp6.1	9.95E-60	-0.891	0.409	0.63	1.99E-55	PA-2
Plod1.1	1.31E-59	0.511	0.271	0.104	2.60E-55	PA-2
Klf4.1	7.16E-59	-0.747	0.343	0.553	1.43E-54	PA-2
Ssc5d	7.32E-59	0.500	0.301	0.122	1.46E-54	PA-2
Apbb2.1	8.25E-59	-0.533	0.794	0.89	1.65E-54	PA-2
Rras2.1	6.20E-58	-0.857	0.27	0.486	1.24E-53	PA-2
4930402H24Ri	2.08E-57	0.548	0.794	0.622	4.16E-53	PA-2
Col14a1.1	2.33E-57	-0.619	0.673	0.841	4.64E-53	PA-2
Mycbp2.1	5.66E-57	0.524	0.877	0.767	1.13E-52	PA-2
Rab31.1	1.37E-56	0.560	0.364	0.172	2.73E-52	PA-2
Map4k4.1	1.64E-56	0.511	0.849	0.699	3.28E-52	PA-2
Pdzd2.1	1.69E-56	-0.756	0.427	0.617	3.37E-52	PA-2
mt-Cytb.1	3.22E-56	0.537	0.974	0.913	6.43E-52	PA-2
Pdgfrb.1	4.50E-56	0.598	0.442	0.242	8.98E-52	PA-2
Gsn.1	9.09E-56	0.593	0.967	0.895	1.82E-51	PA-2
St5.1	1.03E-55	0.659	0.62	0.419	2.05E-51	PA-2
Ebf2.1	1.10E-55	-0.560	0.967	0.976	2.20E-51	PA-2
Adamtsl3	1.12E-55	-0.887	0.185	0.406	2.24E-51	PA-2
Adamts5.1	1.76E-55	-0.689	0.809	0.857	3.52E-51	PA-2
Bach2.1	7.67E-55	-0.688	0.403	0.606	1.53E-50	PA-2
mt-Co2.1	1.16E-54	0.507	0.981	0.938	2.32E-50	PA-2
Antxr1.1	1.80E-54	-0.511	0.809	0.89	3.60E-50	PA-2
Clip4.1	2.82E-54	-0.651	0.14	0.35	5.63E-50	PA-2
Plekha5	4.75E-54	0.537	0.838	0.698	9.47E-50	PA-2
Snrk.1	6.28E-54	0.591	0.428	0.231	1.25E-49	PA-2
Eya4.1	2.53E-53	-0.849	0.249	0.469	5.06E-49	PA-2
Adh1.1	5.95E-53	0.706	0.301	0.134	1.19E-48	PA-2
Ptprd	1.72E-52	0.704	0.692	0.515	3.44E-48	PA-2
Metrnl.1	5.81E-52	-0.663	0.117	0.31	1.16E-47	PA-2
Prkg2.1	1.62E-51	-0.731	0.088	0.278	3.23E-47	PA-2
Apbb1ip.1	4.90E-51	0.632	0.797	0.664	9.79E-47	PA-2
Adam19.1	7.23E-51	0.684	0.632	0.454	1.44E-46	PA-2
Stxbp5.1	3.16E-50	-0.664	0.584	0.724	6.30E-46	PA-2
Fgfr1.1	4.09E-50	-0.527	0.689	0.801	8.17E-46	PA-2
Flrt2.1	1.51E-49	-0.631	0.761	0.857	3.01E-45	PA-2
Ccdc80.1	5.61E-49	0.611	0.79	0.634	1.12E-44	PA-2
Spsb1.1	6.69E-49	-0.655	0.354	0.548	1.33E-44	PA-2
Twist2	6.76E-49	0.569	0.385	0.203	1.35E-44	PA-2
Phldb2.1	1.54E-48	0.662	0.599	0.412	3.08E-44	PA-2
Prex2.1	2.27E-48	0.621	0.608	0.415	4.53E-44	PA-2
Adra1b.1	3.62E-48	-0.702	0.094	0.278	7.23E-44	PA-2
Fhl1.1	4.93E-48	-0.713	0.239	0.436	9.84E-44	PA-2
St6gal1.1	9.06E-48	-0.679	0.192	0.391	1.81E-43	PA-2
Rin3.1	1.11E-47	-0.621	0.121	0.311	2.22E-43	PA-2
Plcxd3	2.35E-47	-0.954	0.164	0.37	4.70E-43	PA-2
Cadm3.1	3.57E-47	-0.681	0.193	0.385	7.12E-43	PA-2
Il1r1	3.59E-47	0.662	0.731	0.58	7.16E-43	PA-2
Prss23.1	3.64E-47	-0.746	0.297	0.481	7.26E-43	PA-2
Atxn1.1	4.33E-47	0.564	0.73	0.553	8.65E-43	PA-2
Sh3pxd2a.1	4.44E-47	0.514	0.333	0.163	8.86E-43	PA-2
Fam78b.1	4.55E-47	0.525	0.29	0.131	9.07E-43	PA-2
Cyth3.1	5.97E-47	0.684	0.712	0.549	1.19E-42	PA-2
Slc8a1	9.05E-47	-0.701	0.539	0.708	1.81E-42	PA-2
Frmd4a.1	3.16E-46	0.594	0.474	0.279	6.30E-42	PA-2
Enox1	3.43E-46	0.534	0.726	0.55	6.84E-42	PA-2
Wnt2.1	3.71E-46	-0.656	0.104	0.287	7.41E-42	PA-2
Hspg2	5.03E-46	0.567	0.669	0.52	1.00E-41	PA-2
Map3k5.1	6.35E-46	0.575	0.597	0.421	1.27E-41	PA-2
Il18.1	3.90E-45	-0.641	0.164	0.349	7.78E-41	PA-2

Iqsec1.1	4.23E-45	-0.615	0.311	0.496	8.45E-41	PA-2
Serping1	7.18E-45	-0.676	0.711	0.802	1.43E-40	PA-2
Psd3.1	8.21E-45	-0.545	0.879	0.914	1.64E-40	PA-2
Scara3.1	2.25E-44	-0.620	0.261	0.442	4.48E-40	PA-2
Wnt5b.1	2.73E-44	-0.743	0.083	0.251	5.45E-40	PA-2
Raph1.1	3.84E-44	0.535	0.638	0.455	7.66E-40	PA-2
Atp2b1.1	5.08E-44	-0.505	0.607	0.743	1.01E-39	PA-2
Abca5.1	6.19E-44	-0.629	0.206	0.392	1.24E-39	PA-2
Colec12.1	1.47E-43	0.613	0.581	0.394	2.94E-39	PA-2
Arhgef3	2.20E-43	-0.559	0.51	0.684	4.38E-39	PA-2
Igfbp4.1	1.30E-42	-0.648	0.295	0.483	2.60E-38	PA-2
Pcdh9.1	1.91E-42	-0.879	0.389	0.574	3.81E-38	PA-2
Rnf144a	2.60E-42	0.593	0.256	0.115	5.19E-38	PA-2
Klf2.1	3.56E-42	-0.621	0.173	0.354	7.11E-38	PA-2
Tshz3	4.56E-42	0.558	0.623	0.45	9.11E-38	PA-2
Robo1.1	6.08E-42	-0.881	0.439	0.599	1.21E-37	PA-2
Angpt1.1	7.53E-42	0.595	0.575	0.376	1.50E-37	PA-2
Basp1.1	1.63E-41	-0.643	0.229	0.412	3.25E-37	PA-2
Pamr1.1	2.01E-41	-0.576	0.405	0.586	4.02E-37	PA-2
Adamtsl4.1	2.32E-41	-0.564	0.157	0.332	4.63E-37	PA-2
Ly6c1.1	2.35E-41	-0.665	0.28	0.465	4.70E-37	PA-2
Clec3b	3.85E-41	-0.613	0.74	0.866	7.69E-37	PA-2
Glce.1	4.13E-41	-0.667	0.305	0.48	8.24E-37	PA-2
Sh3rf3.1	4.19E-41	0.510	0.441	0.256	8.36E-37	PA-2
Cpne8.1	1.06E-40	-0.528	0.537	0.707	2.11E-36	PA-2
Procr.1	2.85E-40	-0.659	0.208	0.381	5.69E-36	PA-2
Mef2c	4.85E-40	-0.570	0.397	0.565	9.69E-36	PA-2
Jag1.1	5.31E-40	-0.707	0.263	0.434	1.06E-35	PA-2
Lvrn.1	8.31E-40	-0.654	0.419	0.599	1.66E-35	PA-2
Nipal2.1	8.31E-40	-0.652	0.211	0.392	1.66E-35	PA-2
Atp6v0a1.1	1.36E-39	0.506	0.439	0.268	2.71E-35	PA-2
Cdk8	1.74E-39	0.548	0.71	0.554	3.47E-35	PA-2
Epha3.1	1.77E-39	-0.829	0.208	0.393	3.54E-35	PA-2
Arl4a.1	3.60E-39	-0.607	0.28	0.451	7.19E-35	PA-2
Disp1.1	5.12E-39	-0.578	0.37	0.537	1.02E-34	PA-2
Hspa12b	9.29E-39	-0.539	0.099	0.261	1.86E-34	PA-2
Pde7a.1	3.97E-38	-0.529	0.349	0.534	7.93E-34	PA-2
Gpm6b.1	4.32E-38	0.752	0.408	0.245	8.63E-34	PA-2
Cp.1	7.88E-38	0.548	0.324	0.172	1.57E-33	PA-2
Zyx.1	1.55E-37	-0.526	0.147	0.313	3.10E-33	PA-2
Cd248.1	3.29E-37	-0.670	0.347	0.512	6.58E-33	PA-2
Rhoq.1	4.05E-37	-0.531	0.505	0.639	8.09E-33	PA-2
Smurf1.1	4.62E-37	-0.570	0.471	0.615	9.23E-33	PA-2
Lox	4.82E-37	0.620	0.288	0.15	9.62E-33	PA-2
C3.1	7.15E-37	-0.755	0.426	0.596	1.43E-32	PA-2
Emp1.1	2.08E-36	0.548	0.456	0.294	4.15E-32	PA-2
Cacna1c.1	2.15E-36	0.561	0.37	0.209	4.29E-32	PA-2
Clmp	2.65E-36	0.504	0.662	0.49	5.28E-32	PA-2
Ly6a.1	6.83E-36	-0.579	0.459	0.626	1.36E-31	PA-2
Gda	1.06E-35	-0.529	0.329	0.5	2.11E-31	PA-2
Tbc1d12.1	1.96E-35	-0.554	0.477	0.614	3.91E-31	PA-2
Dlg2.1	5.20E-35	-0.668	0.172	0.339	1.04E-30	PA-2
Ppp1r9a.1	2.09E-34	-0.609	0.221	0.389	4.17E-30	PA-2
Col8a1	3.40E-34	0.923	0.305	0.165	6.78E-30	PA-2
Bicd1.1	3.68E-34	-0.579	0.267	0.436	7.35E-30	PA-2
Mylip.1	1.70E-33	-0.525	0.106	0.252	3.40E-29	PA-2
Fbln2	2.27E-33	0.542	0.764	0.643	4.53E-29	PA-2
Igf1.1	2.76E-33	0.533	0.35	0.199	5.52E-29	PA-2
Xkr4.1	3.33E-33	0.668	0.421	0.266	6.65E-29	PA-2
Pi15	5.65E-33	-0.527	0.121	0.274	1.13E-28	PA-2
Dbn1.1	2.67E-32	-0.506	0.275	0.434	5.34E-28	PA-2
Prr16.1	6.08E-32	0.505	0.331	0.183	1.21E-27	PA-2

Nedd4l	7.28E-32	0.551	0.542	0.389	1.45E-27	PA-2
Plpp3.1	3.61E-31	-0.514	0.845	0.89	7.20E-27	PA-2
Slc16a9.1	4.04E-31	-0.599	0.213	0.364	8.07E-27	PA-2
Gabra3	7.43E-31	-0.657	0.383	0.541	1.48E-26	PA-2
Fam110b	1.07E-30	0.528	0.348	0.209	2.13E-26	PA-2
Daglb.1	5.02E-30	-0.644	0.231	0.379	1.00E-25	PA-2
Adamtsl5	9.99E-30	-0.509	0.267	0.424	1.99E-25	PA-2
Irs1.1	1.58E-29	0.505	0.569	0.417	3.16E-25	PA-2
Nckap5.1	1.29E-28	0.521	0.679	0.547	2.58E-24	PA-2
Rps15.1	3.17E-28	-0.776	0.171	0.312	6.33E-24	PA-2
Hpgd	3.82E-28	0.693	0.423	0.283	7.62E-24	PA-2
Mpp6	5.74E-26	-0.501	0.255	0.399	1.15E-21	PA-2
Sfrp2	1.08E-25	-0.559	0.157	0.293	2.16E-21	PA-2
Sned1.1	1.65E-25	0.677	0.431	0.298	3.29E-21	PA-2
Col1a1.1	3.06E-25	0.538	0.835	0.76	6.11E-21	PA-2
Chsy3	2.79E-23	0.501	0.344	0.219	5.58E-19	PA-2
Dkk2	3.60E-22	1.057	0.256	0.153	7.19E-18	PA-2
Dpt.1	1.95E-21	-0.541	0.625	0.71	3.89E-17	PA-2
Rps21.1	2.32E-21	-0.637	0.247	0.37	4.63E-17	PA-2
Postn	5.09E-20	0.676	0.507	0.389	1.01E-15	PA-2
Aebp1	2.27E-19	-0.568	0.471	0.564	4.52E-15	PA-2
Esr1	3.08E-19	0.544	0.712	0.648	6.15E-15	PA-2
Rnf220.1	1.74E-17	-0.632	0.184	0.283	3.48E-13	PA-2
Thsd7a	3.49E-17	0.536	0.852	0.806	6.97E-13	PA-2
Gas1.1	4.01E-17	0.561	0.375	0.272	8.00E-13	PA-2
C7.1	2.32E-11	-0.734	0.39	0.478	4.64E-07	PA-2
Tmtc2.1	4.28E-10	-0.643	0.252	0.325	8.54E-06	PA-2
Eln	4.75E-06	0.619	0.588	0.661	0.094892669	PA-2
Fmo2.1	0	3.021	0.876	0.145	0	PA-3
C7.2	0	2.934	0.912	0.394	0	PA-3
Ccbe1.1	0	2.409	0.673	0.091	0	PA-3
Meox2.1	0	2.398	0.837	0.207	0	PA-3
F3.1	0	2.393	0.703	0.093	0	PA-3
Gdf10.1	0	2.099	0.621	0.05	0	PA-3
Vit.1	5.69E-274	1.581	0.648	0.135	1.14E-269	PA-3
Nav3.2	3.55E-257	-3.001	0.438	0.892	7.09E-253	PA-3
Prdm6	2.14E-255	1.526	0.494	0.075	4.28E-251	PA-3
Gpnmb	1.40E-244	1.195	0.438	0.057	2.79E-240	PA-3
Epha3.2	3.32E-236	1.786	0.822	0.286	6.63E-232	PA-3
Prickle1.1	3.46E-236	2.001	0.836	0.342	6.90E-232	PA-3
Dpp4.2	6.24E-236	-2.518	0.366	0.847	1.25E-231	PA-3
Mgp	1.06E-235	1.795	0.424	0.055	2.12E-231	PA-3
Chrdl1.1	7.77E-234	1.943	0.626	0.154	1.55E-229	PA-3
Tspan11	4.70E-229	1.229	0.388	0.046	9.38E-225	PA-3
Gria4.1	2.98E-226	3.034	0.502	0.097	5.94E-222	PA-3
Creb5.2	1.74E-222	-2.113	0.7	0.925	3.47E-218	PA-3
Abi3bp.1	1.66E-220	1.451	0.974	0.814	3.31E-216	PA-3
Anxa3.2	7.70E-218	-2.155	0.49	0.861	1.54E-213	PA-3
Inmt.1	4.05E-213	1.746	0.54	0.118	8.08E-209	PA-3
Kcnt2.1	1.27E-212	1.668	0.879	0.435	2.54E-208	PA-3
Pde4d.1	1.96E-210	1.896	0.597	0.151	3.92E-206	PA-3
Gas6.1	3.68E-209	1.443	0.515	0.105	7.35E-205	PA-3
Gpr39	8.05E-208	1.762	0.371	0.048	1.61E-203	PA-3
Nalcn.1	4.47E-207	1.499	0.55	0.124	8.93E-203	PA-3
Tiam2.1	4.45E-204	1.207	0.451	0.078	8.88E-200	PA-3
Sgip1.2	2.44E-199	1.309	0.636	0.163	4.88E-195	PA-3
Igfbp7.2	2.33E-195	1.585	0.82	0.374	4.66E-191	PA-3
Plxdc2.1	1.82E-193	-1.349	0.803	0.979	3.63E-189	PA-3
Nova1.1	3.33E-189	-1.525	0.738	0.953	6.64E-185	PA-3
Limch1.2	8.48E-187	-2.324	0.169	0.709	1.69E-182	PA-3
Fbn1.2	9.22E-185	-1.679	0.943	0.98	1.84E-180	PA-3
Ltbp4.1	1.40E-181	1.613	0.654	0.23	2.80E-177	PA-3

Bgn.2	1.28E-180	1.542	0.731	0.281	2.55E-176	PA-3
C2	2.07E-180	1.081	0.351	0.051	4.12E-176	PA-3
Pid1.1	3.77E-178	1.527	0.964	0.731	7.53E-174	PA-3
Nfib.2	2.31E-177	1.101	0.991	0.925	4.61E-173	PA-3
Sema3c.2	6.21E-172	-1.884	0.714	0.89	1.24E-167	PA-3
Csmd1	5.32E-168	2.321	0.507	0.129	1.06E-163	PA-3
Adamts5.2	8.14E-168	-1.657	0.575	0.885	1.62E-163	PA-3
Cmah.2	9.24E-168	-2.240	0.148	0.668	1.84E-163	PA-3
Ebf1.1	2.29E-167	-1.088	0.99	0.998	4.56E-163	PA-3
Pla1a.2	2.47E-167	-1.836	0.141	0.669	4.94E-163	PA-3
Sgcd.1	4.85E-167	1.612	0.877	0.506	9.67E-163	PA-3
Cd55.2	1.47E-166	-2.047	0.285	0.736	2.93E-162	PA-3
Itgbl1.2	4.11E-164	1.384	0.944	0.667	8.20E-160	PA-3
Gsn.2	1.20E-162	1.661	0.991	0.899	2.39E-158	PA-3
Pi16.2	3.08E-162	-2.545	0.387	0.773	6.15E-158	PA-3
Efemp1.2	5.52E-161	-1.865	0.393	0.793	1.10E-156	PA-3
Tek.2	1.78E-158	-1.808	0.238	0.721	3.55E-154	PA-3
Grem2	8.10E-157	1.058	0.282	0.035	1.62E-152	PA-3
Sema3e.2	1.07E-152	-2.475	0.132	0.634	2.14E-148	PA-3
Ism1.1	1.81E-152	1.627	0.469	0.119	3.61E-148	PA-3
Stxbp6.2	1.14E-149	-1.708	0.299	0.755	2.27E-145	PA-3
Sdk1.2	2.04E-148	-1.212	0.721	0.943	4.08E-144	PA-3
Nrp1.2	2.48E-145	1.289	0.825	0.431	4.95E-141	PA-3
Spock2	6.08E-143	0.876	0.289	0.043	1.21E-138	PA-3
Efna5.2	7.24E-143	-2.148	0.184	0.657	1.44E-138	PA-3
Ston2.2	2.02E-141	-1.835	0.192	0.657	4.04E-137	PA-3
Psd3.2	3.53E-140	-1.246	0.709	0.935	7.05E-136	PA-3
Vav3.2	8.69E-139	1.033	0.622	0.204	1.73E-134	PA-3
Ephb2	3.43E-133	0.979	0.317	0.057	6.85E-129	PA-3
Ptprj.2	1.54E-132	-1.513	0.414	0.788	3.08E-128	PA-3
Hmgcs2	2.33E-132	0.825	0.275	0.041	4.65E-128	PA-3
Cdh13.1	3.42E-132	1.343	0.776	0.405	6.83E-128	PA-3
Frmd6.2	3.63E-132	1.205	0.915	0.682	7.25E-128	PA-3
Tgfbi.1	6.94E-131	1.106	0.402	0.098	1.38E-126	PA-3
Slc6a7	1.29E-130	0.898	0.265	0.038	2.57E-126	PA-3
Plcxd3.1	1.37E-130	1.916	0.648	0.279	2.73E-126	PA-3
Igf1.2	2.53E-130	1.225	0.553	0.185	5.06E-126	PA-3
Smurf2.2	1.04E-128	-1.284	0.679	0.896	2.07E-124	PA-3
Lum.2	1.19E-127	1.245	0.567	0.212	2.38E-123	PA-3
Igfbp5.2	2.50E-126	-2.342	0.255	0.671	4.98E-122	PA-3
Cfh.1	6.59E-126	1.269	0.566	0.199	1.32E-121	PA-3
Rorb.2	2.85E-125	-2.057	0.072	0.531	5.69E-121	PA-3
Podn.1	1.49E-124	1.000	0.552	0.193	2.97E-120	PA-3
Ank2.2	3.93E-124	1.048	0.923	0.646	7.85E-120	PA-3
Steap4.1	6.82E-122	0.989	0.361	0.082	1.36E-117	PA-3
Cd9.1	2.21E-121	1.082	0.529	0.184	4.41E-117	PA-3
Angpt1.2	2.86E-121	1.232	0.751	0.372	5.71E-117	PA-3
Ak5	8.00E-120	0.933	0.25	0.038	1.60E-115	PA-3
Boc.1	9.65E-118	1.223	0.685	0.327	1.93E-113	PA-3
B3galt1.1	9.63E-116	1.180	0.868	0.548	1.92E-111	PA-3
Efhd1.2	3.16E-115	-1.627	0.07	0.505	6.31E-111	PA-3
Calcrl.2	9.08E-115	-1.762	0.104	0.546	1.81E-110	PA-3
Mast4	2.27E-114	-1.128	0.806	0.961	4.54E-110	PA-3
Vgll3.1	3.70E-114	-1.377	0.303	0.707	7.39E-110	PA-3
Cacna1c.2	6.64E-114	1.055	0.555	0.199	1.33E-109	PA-3
Ppl	1.40E-113	0.951	0.377	0.096	2.80E-109	PA-3
Hmcn2.2	1.15E-110	1.276	0.629	0.268	2.29E-106	PA-3
Cygb.1	1.37E-110	1.078	0.522	0.197	2.74E-106	PA-3
Pcsk6.2	2.46E-109	-1.499	0.251	0.65	4.90E-105	PA-3
Ano3.2	1.06E-108	-1.604	0.107	0.543	2.11E-104	PA-3
Ghr.1	4.18E-107	1.118	0.964	0.854	8.35E-103	PA-3
Slco2b1.2	1.44E-106	1.009	0.524	0.189	2.87E-102	PA-3

Ext1.1	2.77E-106	-1.066	0.894	0.969	5.52E-102	PA-3
Prkd1.2	4.08E-106	1.091	0.816	0.503	8.15E-102	PA-3
Ldb2.2	5.76E-105	0.936	0.674	0.295	1.15E-100	PA-3
Wdr66.1	2.16E-104	0.907	0.419	0.126	4.32E-100	PA-3
Hdac7.2	4.05E-102	-1.080	0.551	0.822	8.08E-98	PA-3
Utrn.1	7.51E-102	-0.822	0.907	0.965	1.50E-97	PA-3
Galnt16.2	2.98E-100	-1.265	0.346	0.695	5.94E-96	PA-3
Eya2.2	3.19E-98	0.914	0.572	0.224	6.37E-94	PA-3
Axl.2	6.42E-98	-1.083	0.552	0.796	1.28E-93	PA-3
Cst3	2.37E-96	1.647	0.425	0.149	4.74E-92	PA-3
Vegfc.1	1.56E-94	1.173	0.474	0.176	3.11E-90	PA-3
Gas7.2	1.64E-94	-1.200	0.711	0.884	3.27E-90	PA-3
Entpd2.1	3.59E-94	1.000	0.575	0.264	7.17E-90	PA-3
Nid1.1	5.80E-93	-0.882	0.796	0.919	1.16E-88	PA-3
Fndc1.2	1.46E-92	-0.955	0.827	0.931	2.91E-88	PA-3
Gfpt2.2	4.35E-90	-1.148	0.563	0.803	8.68E-86	PA-3
Robo1.2	8.21E-90	-1.345	0.23	0.612	1.64E-85	PA-3
Col4a1.2	1.49E-88	0.806	0.769	0.424	2.98E-84	PA-3
Agmo.2	1.52E-88	0.922	0.923	0.686	3.03E-84	PA-3
Aspn	8.33E-88	-1.178	0.243	0.624	1.66E-83	PA-3
Col4a2.2	1.03E-85	0.831	0.688	0.341	2.05E-81	PA-3
Lurap1l.2	1.59E-85	-1.336	0.221	0.582	3.17E-81	PA-3
Vcan.1	3.83E-85	-1.216	0.432	0.73	7.65E-81	PA-3
Il11ra1.1	1.26E-83	0.861	0.445	0.17	2.51E-79	PA-3
Ebf2.2	1.35E-83	-0.863	0.94	0.979	2.70E-79	PA-3
Arhgap24.2	5.80E-83	0.838	0.976	0.892	1.16E-78	PA-3
Col1a1.2	9.10E-83	1.248	0.918	0.756	1.82E-78	PA-3
Myo1e.2	3.40E-82	-1.172	0.245	0.586	6.78E-78	PA-3
Kank1.2	3.94E-82	-1.377	0.118	0.467	7.87E-78	PA-3
Ppp2r2b.1	4.76E-82	-1.660	0.114	0.473	9.50E-78	PA-3
Deptor.2	7.29E-82	0.999	0.583	0.273	1.46E-77	PA-3
Fbxl7.1	3.13E-80	-1.083	0.617	0.848	6.24E-76	PA-3
Svep1.2	6.85E-80	1.012	0.826	0.578	1.37E-75	PA-3
Pdgfc.2	3.14E-78	-1.398	0.177	0.518	6.26E-74	PA-3
Gan.2	3.58E-78	-1.585	0.185	0.511	7.14E-74	PA-3
Bmp6.2	8.83E-78	-1.618	0.045	0.379	1.76E-73	PA-3
Adamts16.2	2.29E-77	-1.751	0.025	0.352	4.57E-73	PA-3
Pde5a	8.71E-77	0.696	0.277	0.072	1.74E-72	PA-3
Mfap5.2	1.66E-76	-1.115	0.685	0.84	3.31E-72	PA-3
Col15a1.2	4.64E-76	0.627	0.439	0.156	9.26E-72	PA-3
Rras2.2	4.70E-76	-1.265	0.141	0.482	9.38E-72	PA-3
Aldh1a3.2	6.77E-76	-1.315	0.066	0.4	1.35E-71	PA-3
Ror1.2	1.50E-75	0.885	0.817	0.541	3.00E-71	PA-3
Ackr3.2	1.63E-75	-1.157	0.384	0.678	3.24E-71	PA-3
Pde8a.2	3.63E-75	-1.010	0.524	0.76	7.25E-71	PA-3
Nrip1.1	3.70E-75	0.917	0.861	0.728	7.39E-71	PA-3
Srgap1	1.36E-74	-0.879	0.639	0.86	2.71E-70	PA-3
Myo5a.2	1.70E-74	0.702	0.506	0.214	3.39E-70	PA-3
Tmtc1	1.74E-74	1.029	0.639	0.353	3.47E-70	PA-3
Dennd4a.1	3.60E-74	1.067	0.695	0.427	7.19E-70	PA-3
Dact2.2	5.59E-74	-1.485	0.03	0.347	1.12E-69	PA-3
Myo1d.1	3.28E-73	0.660	0.3	0.084	6.55E-69	PA-3
Adamtsl3.1	1.05E-72	0.945	0.622	0.32	2.09E-68	PA-3
Bcl2.1	1.91E-72	0.957	0.54	0.266	3.82E-68	PA-3
Cxcl12	1.12E-71	0.680	0.277	0.078	2.23E-67	PA-3
Sulf1.2	1.39E-71	-0.891	0.735	0.873	2.78E-67	PA-3
Zfp462.2	2.03E-71	-1.122	0.399	0.677	4.04E-67	PA-3
Ctsk	5.44E-71	0.836	0.333	0.114	1.09E-66	PA-3
Tanc2	7.92E-71	-1.032	0.311	0.625	1.58E-66	PA-3
Gxylt2.1	2.06E-70	0.896	0.571	0.289	4.11E-66	PA-3
Mgll.2	6.56E-69	-1.039	0.133	0.452	1.31E-64	PA-3
Pde3a	8.02E-69	-1.067	0.418	0.707	1.60E-64	PA-3

Gli2.2	2.46E-68	0.797	0.377	0.139	4.91E-64	PA-3
Nhsl1.2	4.48E-68	-0.959	0.542	0.768	8.94E-64	PA-3
Cgnl1	1.99E-67	0.623	0.276	0.08	3.97E-63	PA-3
Nr2f2	3.41E-67	0.718	0.265	0.075	6.80E-63	PA-3
Car8.2	6.45E-67	-1.241	0.101	0.416	1.29E-62	PA-3
Mmp3.2	1.84E-66	1.332	0.422	0.176	3.67E-62	PA-3
Zeb1.1	4.74E-66	-0.680	0.909	0.962	9.46E-62	PA-3
Dpyd	1.30E-65	0.868	0.573	0.294	2.59E-61	PA-3
Reps2.2	2.60E-65	0.754	0.57	0.288	5.19E-61	PA-3
Nhs	3.09E-65	0.894	0.352	0.125	6.17E-61	PA-3
Aff2	4.19E-65	0.983	0.618	0.34	8.36E-61	PA-3
Ddr2.2	1.27E-64	-0.658	0.903	0.951	2.53E-60	PA-3
Stxbp5.2	1.28E-64	-0.939	0.481	0.724	2.55E-60	PA-3
Thrb.1	1.51E-64	0.854	0.77	0.534	3.01E-60	PA-3
Dock10.2	3.35E-64	0.757	0.653	0.365	6.69E-60	PA-3
Basp1.2	3.50E-64	-1.055	0.107	0.41	6.98E-60	PA-3
Man2a1.1	6.28E-64	0.793	0.913	0.798	1.25E-59	PA-3
Fmo1.1	1.11E-63	1.162	0.49	0.237	2.22E-59	PA-3
Srpx.1	2.62E-63	0.744	0.366	0.14	5.23E-59	PA-3
Sparcl1.2	4.06E-63	0.714	0.348	0.122	8.10E-59	PA-3
Slc4a4.2	5.55E-63	-0.734	0.704	0.878	1.11E-58	PA-3
Pdzd2.2	1.51E-62	-1.014	0.323	0.612	3.02E-58	PA-3
Col1a2.1	1.55E-62	0.883	0.98	0.938	3.10E-58	PA-3
Dock4.2	3.87E-62	-0.891	0.738	0.872	7.72E-58	PA-3
Maf	4.10E-62	-0.852	0.326	0.629	8.18E-58	PA-3
Ifi207.1	5.37E-62	0.749	0.794	0.582	1.07E-57	PA-3
Flnb.2	5.40E-62	-0.943	0.418	0.672	1.08E-57	PA-3
Dock11.1	1.53E-61	-0.831	0.438	0.708	3.05E-57	PA-3
Prr16.2	2.48E-61	1.030	0.428	0.185	4.96E-57	PA-3
Itpr1.1	4.70E-61	0.776	0.553	0.288	9.39E-57	PA-3
Gpc6.2	7.20E-61	-0.672	0.959	0.981	1.44E-56	PA-3
Fgf2.2	1.09E-60	-0.981	0.275	0.564	2.18E-56	PA-3
Sfrp2.1	1.75E-59	1.462	0.463	0.235	3.49E-55	PA-3
Tgfbr2.2	9.64E-59	-0.790	0.609	0.797	1.92E-54	PA-3
Col12a1.1	1.26E-58	-1.410	0.085	0.368	2.52E-54	PA-3
Adgrg2.2	1.94E-57	-1.052	0.208	0.493	3.88E-53	PA-3
Sytl2.2	3.25E-57	-0.965	0.041	0.311	6.48E-53	PA-3
Fyn.2	3.21E-56	-0.661	0.771	0.883	6.41E-52	PA-3
Eps8.2	4.21E-56	0.661	0.647	0.368	8.40E-52	PA-3
Gabra3.1	5.12E-56	1.016	0.71	0.478	1.02E-51	PA-3
Kif2a.2	6.19E-56	-0.882	0.447	0.68	1.24E-51	PA-3
Clip1.2	1.68E-55	-0.787	0.544	0.753	3.35E-51	PA-3
Armh4	2.10E-55	0.631	0.303	0.108	4.18E-51	PA-3
Pde7a.2	2.32E-55	-0.815	0.238	0.53	4.63E-51	PA-3
Rgs6.2	2.75E-55	-1.570	0.153	0.432	5.50E-51	PA-3
Dcbld2.1	6.21E-55	-0.763	0.507	0.74	1.24E-50	PA-3
Dcn	6.90E-55	0.683	0.99	0.954	1.38E-50	PA-3
Asap2.1	7.78E-55	-0.810	0.392	0.65	1.55E-50	PA-3
Fign.1	8.54E-55	0.811	0.429	0.191	1.70E-50	PA-3
Sbsn.2	1.23E-54	-1.241	0.042	0.3	2.46E-50	PA-3
Slco3a1.2	1.27E-54	-0.805	0.721	0.832	2.54E-50	PA-3
Smurf1.2	1.41E-54	-0.864	0.351	0.617	2.81E-50	PA-3
Pde10a	2.21E-54	-0.887	0.178	0.468	4.42E-50	PA-3
Naalad2	5.09E-54	0.646	0.33	0.126	1.02E-49	PA-3
Gas1.2	5.23E-54	0.857	0.496	0.265	1.04E-49	PA-3
Trim30d.2	5.44E-54	-0.989	0.133	0.406	1.09E-49	PA-3
Emilin2.2	2.74E-53	-0.883	0.35	0.602	5.46E-49	PA-3
Ltbp1.1	2.06E-52	-0.666	0.729	0.874	4.12E-48	PA-3
Rhobtb1.1	2.12E-52	0.679	0.356	0.146	4.22E-48	PA-3
Abtb2	2.51E-52	0.783	0.463	0.227	5.00E-48	PA-3
Cers6.1	1.52E-51	-0.817	0.313	0.576	3.03E-47	PA-3
Aff3.1	1.71E-51	0.791	0.641	0.403	3.41E-47	PA-3

Dpep1	2.90E-51	0.750	0.663	0.443	5.79E-47	PA-3
Postn.1	1.28E-50	-1.308	0.18	0.448	2.55E-46	PA-3
Fam13c.1	1.57E-50	-0.818	0.179	0.451	3.13E-46	PA-3
Dock9.1	3.52E-50	0.823	0.675	0.447	7.02E-46	PA-3
Ugdh.2	4.36E-50	-0.963	0.353	0.598	8.71E-46	PA-3
Adgrd1.2	6.55E-50	-0.602	0.837	0.914	1.31E-45	PA-3
Spsb1.2	1.13E-49	-0.903	0.291	0.537	2.26E-45	PA-3
Acvr2a.2	1.42E-49	0.841	0.672	0.449	2.83E-45	PA-3
Aldh1a1.1	3.45E-49	0.759	0.878	0.756	6.89E-45	PA-3
Col3a1.2	4.54E-49	0.803	0.993	0.976	9.06E-45	PA-3
Gm33869.1	4.72E-49	-0.824	0.213	0.474	9.41E-45	PA-3
Ccn5	6.61E-49	0.638	0.265	0.091	1.32E-44	PA-3
Auts2.2	9.93E-49	0.615	0.866	0.66	1.98E-44	PA-3
Fnbp1.1	3.12E-48	0.658	0.609	0.37	6.23E-44	PA-3
Itga11.1	5.56E-48	-0.824	0.264	0.531	1.11E-43	PA-3
Samhd1.2	7.09E-48	-0.772	0.531	0.733	1.41E-43	PA-3
Hivep2.1	1.13E-47	-0.600	0.858	0.936	2.25E-43	PA-3
Col14a1.2	1.70E-47	-0.801	0.652	0.826	3.39E-43	PA-3
Prkca.2	5.22E-47	-0.602	0.833	0.923	1.04E-42	PA-3
Rin3.2	5.54E-47	-0.777	0.058	0.3	1.11E-42	PA-3
Marcks.2	9.93E-46	-0.963	0.408	0.626	1.98E-41	PA-3
Plpp1.2	1.93E-45	0.728	0.505	0.272	3.84E-41	PA-3
Atp10d.1	1.96E-45	0.647	0.372	0.17	3.91E-41	PA-3
Daglb.2	4.22E-45	-0.934	0.134	0.377	8.42E-41	PA-3
Fn1.2	7.17E-45	-0.704	0.812	0.895	1.43E-40	PA-3
Srgap2	7.63E-45	-0.558	0.743	0.883	1.52E-40	PA-3
Ebf3	8.09E-45	-0.765	0.3	0.561	1.62E-40	PA-3
Anxa1.2	1.24E-44	-0.742	0.439	0.667	2.48E-40	PA-3
Uap1.2	1.31E-44	-0.913	0.484	0.672	2.62E-40	PA-3
Il18.2	2.13E-44	-0.777	0.096	0.339	4.26E-40	PA-3
Fap.1	3.03E-44	-0.713	0.592	0.787	6.04E-40	PA-3
Cp.2	4.12E-44	0.585	0.387	0.178	8.22E-40	PA-3
Phlda1	4.43E-44	0.609	0.291	0.116	8.85E-40	PA-3
Fam13a	4.59E-44	0.537	0.262	0.095	9.17E-40	PA-3
Rad51b.1	1.07E-43	0.619	0.803	0.585	2.14E-39	PA-3
Nptn.1	1.26E-43	-0.670	0.642	0.78	2.51E-39	PA-3
Col4a4	1.34E-43	0.753	0.321	0.136	2.68E-39	PA-3
Adra1b.2	1.47E-43	-0.827	0.044	0.266	2.94E-39	PA-3
Selenop.1	2.24E-43	0.837	0.364	0.173	4.48E-39	PA-3
Fbln1.1	5.52E-43	0.780	0.476	0.258	1.10E-38	PA-3
Msi2.1	6.11E-43	-0.728	0.361	0.604	1.22E-38	PA-3
Fzd4	6.59E-43	0.574	0.301	0.127	1.32E-38	PA-3
Cask.1	1.91E-42	-0.652	0.601	0.78	3.82E-38	PA-3
Cmip	2.05E-42	-0.695	0.555	0.732	4.09E-38	PA-3
Notch2.1	2.27E-42	-0.706	0.357	0.586	4.53E-38	PA-3
Ssh2	2.64E-42	-0.585	0.764	0.887	5.27E-38	PA-3
Setbp1.2	6.20E-42	0.560	0.907	0.814	1.24E-37	PA-3
Ildr2.2	8.11E-42	-0.974	0.295	0.508	1.62E-37	PA-3
Gask1b.2	1.15E-41	0.598	0.479	0.253	2.30E-37	PA-3
Trio	4.95E-41	-0.584	0.649	0.802	9.87E-37	PA-3
Ecm1	7.55E-41	0.773	0.741	0.561	1.51E-36	PA-3
Atp2b1.2	2.06E-40	-0.621	0.534	0.739	4.11E-36	PA-3
Maml3.2	3.19E-40	0.639	0.771	0.575	6.36E-36	PA-3
Serpinf1	3.86E-40	0.636	0.748	0.558	7.70E-36	PA-3
Itih5	4.57E-40	-0.633	0.447	0.675	9.12E-36	PA-3
Carmil1	1.90E-39	0.557	0.577	0.358	3.79E-35	PA-3
Tbx15.1	5.29E-39	0.620	0.817	0.682	1.06E-34	PA-3
Cacnb4	1.17E-38	-0.769	0.136	0.369	2.33E-34	PA-3
Dhrs3.2	1.33E-38	0.561	0.364	0.175	2.66E-34	PA-3
Sestd1.2	3.06E-38	-0.699	0.49	0.678	6.10E-34	PA-3
Galnt17.1	3.32E-38	0.668	0.728	0.525	6.63E-34	PA-3
Mcc	3.80E-38	-0.681	0.617	0.785	7.59E-34	PA-3

Arhgap42.1	7.82E-38	-0.606	0.764	0.865	1.56E-33	PA-3
Itsn1	1.55E-37	-0.604	0.537	0.729	3.09E-33	PA-3
Plcb4	1.81E-37	0.530	0.886	0.782	3.62E-33	PA-3
Rhoq.2	2.05E-37	-0.685	0.439	0.635	4.10E-33	PA-3
Fgf7.1	5.12E-37	0.785	0.49	0.285	1.02E-32	PA-3
Megf9.1	5.78E-37	-0.691	0.297	0.526	1.15E-32	PA-3
Il31ra	2.59E-36	0.735	0.442	0.252	5.16E-32	PA-3
Pdzrn3.2	2.76E-36	0.614	0.474	0.27	5.50E-32	PA-3
Heg1.1	2.98E-36	-0.532	0.92	0.962	5.95E-32	PA-3
Lars2	3.13E-36	0.601	0.777	0.612	6.25E-32	PA-3
mt-Nd4l.1	3.66E-36	0.557	0.884	0.738	7.30E-32	PA-3
Negr1.2	4.60E-36	-1.158	0.405	0.58	9.18E-32	PA-3
Cmss1	8.69E-36	0.554	0.919	0.769	1.74E-31	PA-3
Enox1.1	9.11E-36	-0.705	0.398	0.615	1.82E-31	PA-3
Mtus1.1	1.21E-35	0.608	0.483	0.284	2.41E-31	PA-3
Cpeb1	2.12E-35	0.618	0.632	0.437	4.24E-31	PA-3
Il6ra.1	3.04E-35	-0.688	0.312	0.525	6.07E-31	PA-3
Trps1.1	1.11E-34	0.650	0.379	0.193	2.22E-30	PA-3
Parp8.1	1.58E-34	-0.723	0.454	0.644	3.16E-30	PA-3
Plpp3.2	1.68E-34	-0.658	0.807	0.891	3.35E-30	PA-3
Phlpp1.1	1.99E-34	-0.630	0.562	0.728	3.97E-30	PA-3
C4b	1.03E-33	0.848	0.48	0.295	2.06E-29	PA-3
Pik3r1.1	1.57E-33	0.560	0.816	0.683	3.14E-29	PA-3
Glp2r.1	1.80E-33	-0.796	0.068	0.26	3.59E-29	PA-3
Khdrbs3	2.34E-33	0.508	0.281	0.125	4.67E-29	PA-3
Iqsec1.2	2.55E-33	-0.678	0.281	0.481	5.09E-29	PA-3
Lims2.2	3.24E-33	-0.633	0.461	0.642	6.47E-29	PA-3
Myof.1	1.28E-32	-0.547	0.624	0.762	2.56E-28	PA-3
Olfr1033	3.96E-32	0.899	0.495	0.317	7.91E-28	PA-3
Gm48542	9.69E-32	-0.630	0.154	0.36	1.93E-27	PA-3
Disp1.2	1.13E-31	-0.630	0.315	0.528	2.25E-27	PA-3
Nr1h4.2	2.72E-31	-0.900	0.235	0.426	5.44E-27	PA-3
Dbn1.2	3.23E-31	-0.632	0.228	0.424	6.44E-27	PA-3
Arhgef28.2	4.00E-31	-0.701	0.493	0.649	7.98E-27	PA-3
Cd47.1	5.80E-31	-0.525	0.624	0.752	1.16E-26	PA-3
Sh3rf3.2	5.94E-31	0.594	0.46	0.273	1.19E-26	PA-3
Adamts12.2	6.57E-31	0.508	0.937	0.828	1.31E-26	PA-3
Glce.2	7.56E-31	-0.688	0.265	0.468	1.51E-26	PA-3
Abca8a.2	1.07E-30	0.568	0.822	0.661	2.14E-26	PA-3
Procr.2	1.30E-30	-0.709	0.173	0.368	2.60E-26	PA-3
Pdgfra	1.59E-30	0.516	0.785	0.667	3.18E-26	PA-3
Ptgis.1	1.60E-30	-0.570	0.129	0.326	3.19E-26	PA-3
Ptprk.1	2.48E-30	0.725	0.397	0.228	4.95E-26	PA-3
Adamtsl1.2	2.60E-30	-0.622	0.836	0.909	5.20E-26	PA-3
Lama4	2.73E-30	-0.521	0.745	0.839	5.46E-26	PA-3
Kdm7a	3.28E-30	-0.568	0.417	0.62	6.56E-26	PA-3
Tbc1d12.2	4.80E-30	-0.619	0.423	0.608	9.59E-26	PA-3
Cdh9.1	8.96E-30	-0.758	0.194	0.393	1.79E-25	PA-3
Lsp1	9.88E-30	-0.560	0.368	0.569	1.97E-25	PA-3
Cacna1a.1	1.67E-29	0.567	0.639	0.469	3.33E-25	PA-3
Epb41l3.1	2.79E-29	-0.663	0.236	0.431	5.57E-25	PA-3
Atp5o	3.02E-29	-0.624	0.199	0.404	6.04E-25	PA-3
Ecm2	5.23E-29	0.528	0.373	0.208	1.04E-24	PA-3
Sned1.2	8.50E-29	0.644	0.48	0.305	1.70E-24	PA-3
Arhgap29	8.63E-29	-0.574	0.424	0.612	1.72E-24	PA-3
Ntn4.2	1.08E-28	-0.736	0.323	0.514	2.16E-24	PA-3
Lamb1	1.14E-28	-0.626	0.205	0.405	2.27E-24	PA-3
Clcn5	2.06E-28	-0.635	0.312	0.502	4.11E-24	PA-3
Tlr4.1	2.92E-28	-0.625	0.342	0.531	5.83E-24	PA-3
Klf12.2	3.47E-28	-0.685	0.505	0.678	6.92E-24	PA-3
Clasp1	7.56E-28	-0.523	0.48	0.651	1.51E-23	PA-3
Ngf	8.30E-28	-0.703	0.103	0.279	1.66E-23	PA-3

Tmtc2.2	2.36E-27	-1.020	0.153	0.332	4.70E-23	PA-3
Pxdn	2.38E-27	-0.599	0.265	0.459	4.75E-23	PA-3
Gpc3	2.43E-27	0.565	0.529	0.339	4.85E-23	PA-3
Hexb	6.09E-27	0.531	0.592	0.424	1.22E-22	PA-3
Nckap5.2	6.81E-27	0.533	0.716	0.556	1.36E-22	PA-3
Ogn.1	7.02E-27	-0.641	0.47	0.64	1.40E-22	PA-3
Aig1.1	8.24E-27	-0.618	0.353	0.548	1.65E-22	PA-3
Osr1	9.27E-27	0.515	0.364	0.213	1.85E-22	PA-3
Dpt.2	1.62E-26	-0.746	0.56	0.71	3.24E-22	PA-3
Fam20a.1	2.62E-26	-0.569	0.372	0.55	5.22E-22	PA-3
Sfrp4	2.82E-26	0.984	0.485	0.347	5.63E-22	PA-3
Ror2	2.99E-26	-0.603	0.178	0.365	5.97E-22	PA-3
Runx1t1	7.80E-26	0.512	0.703	0.53	1.56E-21	PA-3
Agpat4	8.09E-26	-0.578	0.23	0.413	1.62E-21	PA-3
Klf2.2	9.65E-26	-0.599	0.158	0.337	1.93E-21	PA-3
Micu1.1	1.05E-25	-0.526	0.399	0.579	2.10E-21	PA-3
Lamb2	1.28E-25	0.584	0.515	0.367	2.56E-21	PA-3
Serpinh1	1.74E-25	0.532	0.582	0.425	3.47E-21	PA-3
Wnt2.2	5.10E-25	-0.624	0.104	0.268	1.02E-20	PA-3
Immp2l	8.61E-25	-0.683	0.405	0.571	1.72E-20	PA-3
Prss23.2	1.27E-24	-0.722	0.302	0.461	2.53E-20	PA-3
Jpt1.1	3.74E-24	-0.502	0.173	0.348	7.46E-20	PA-3
Zdhhc14.1	5.55E-24	0.502	0.455	0.292	1.11E-19	PA-3
Kctd9.1	7.17E-24	-0.508	0.259	0.446	1.43E-19	PA-3
Abca5.2	7.43E-24	-0.531	0.198	0.374	1.48E-19	PA-3
Meis1	1.14E-23	-0.526	0.352	0.536	2.28E-19	PA-3
Snta1	1.17E-22	0.509	0.341	0.198	2.34E-18	PA-3
Pdgfd.2	2.46E-22	-0.530	0.71	0.817	4.92E-18	PA-3
Il1rl2.1	3.39E-22	-0.525	0.552	0.701	6.76E-18	PA-3
Pdlim1	3.90E-22	-0.525	0.272	0.443	7.79E-18	PA-3
Pex13	1.00E-21	-0.506	0.17	0.335	2.00E-17	PA-3
Lmx1a.1	1.85E-20	-0.544	0.16	0.318	3.70E-16	PA-3
Klhl29.1	9.99E-20	-0.579	0.484	0.611	1.99E-15	PA-3
Ugp2.1	1.13E-19	-0.544	0.526	0.64	2.26E-15	PA-3
Greb1l	1.50E-19	0.503	0.267	0.144	2.99E-15	PA-3
Tmem100.2	8.48E-19	-0.521	0.218	0.37	1.69E-14	PA-3
Eln.1	1.26E-18	0.508	0.749	0.63	2.51E-14	PA-3
Sult1e1.1	1.88E-18	0.756	0.341	0.21	3.75E-14	PA-3
Ccser1	4.33E-18	-0.669	0.173	0.319	8.64E-14	PA-3
Eya4.2	5.61E-18	-0.619	0.29	0.44	1.12E-13	PA-3
Apod	1.20E-17	0.557	0.45	0.324	2.40E-13	PA-3
Smpd3.2	2.81E-17	-0.788	0.45	0.571	5.61E-13	PA-3
Adcy2.1	5.61E-17	-0.516	0.284	0.432	1.12E-12	PA-3
Jag1.2	3.54E-16	-0.535	0.281	0.414	7.06E-12	PA-3
Vegfd.1	3.84E-16	-0.542	0.259	0.389	7.67E-12	PA-3
Hpgd.1	1.77E-15	-0.518	0.197	0.33	3.53E-11	PA-3
Fhl1.2	2.98E-15	-0.504	0.284	0.409	5.95E-11	PA-3
Pde7b	6.90E-15	-0.564	0.226	0.356	1.38E-10	PA-3
Errfi1.1	8.03E-15	0.604	0.754	0.673	1.60E-10	PA-3
Kitl.1	4.08E-14	0.509	0.427	0.309	8.13E-10	PA-3
Mbd1	4.21E-13	0.551	0.286	0.187	8.41E-09	PA-3
Cd248.2	5.51E-13	-0.546	0.387	0.489	1.10E-08	PA-3
Sntg1.2	1.32E-12	-0.882	0.366	0.472	2.63E-08	PA-3
Dmd	5.47E-11	0.672	0.499	0.413	1.09E-06	PA-3
Rnf220.2	2.39E-10	-0.632	0.183	0.273	4.78E-06	PA-3
Top2a	0	2.227	0.637	0.005	0	PA-4
Neil3	0	1.985	0.646	0.007	0	PA-4
Knl1	0	1.903	0.619	0.006	0	PA-4
Kif15	0	1.791	0.637	0.008	0	PA-4
Mki67	0	1.715	0.566	0.004	0	PA-4
Kif11	0	1.668	0.566	0.009	0	PA-4
Kif4	0	1.623	0.566	0.003	0	PA-4

Tpx2	0	1.620	0.584	0.006	0	PA-4
Efcab11	0	1.554	0.566	0.014	0	PA-4
Prc1	0	1.471	0.487	0.005	0	PA-4
Zwilch	0	1.438	0.611	0.018	0	PA-4
Racgap1	0	1.418	0.504	0.004	0	PA-4
Cit	0	1.378	0.549	0.009	0	PA-4
Cenpe	0	1.348	0.442	0.004	0	PA-4
Mis18bp1	0	1.304	0.46	0.006	0	PA-4
Brca1	0	1.298	0.513	0.008	0	PA-4
Iqgap3	0	1.296	0.425	0.003	0	PA-4
Kntc1	0	1.241	0.487	0.005	0	PA-4
Cenph	0	1.169	0.487	0.005	0	PA-4
Cenpi	0	1.037	0.442	0.006	0	PA-4
Kif23	0	1.034	0.372	0.003	0	PA-4
Depdc1a	0	1.029	0.389	0.002	0	PA-4
Rad54b	0	0.977	0.389	0.004	0	PA-4
Melk	0	0.974	0.407	0.002	0	PA-4
Bub1	0	0.968	0.389	0	0	PA-4
Shcbp1	0	0.935	0.407	0.003	0	PA-4
Hmmr	0	0.912	0.31	0.001	0	PA-4
Ttk	0	0.897	0.363	0.003	0	PA-4
Ncapg	0	0.872	0.354	0.002	0	PA-4
Ckap2l	0	0.857	0.372	0.004	0	PA-4
Bub1b	3.41E-306	0.845	0.354	0.004	6.81E-302	PA-4
Cenpf	4.53E-305	1.388	0.381	0.005	9.05E-301	PA-4
Pbk	6.74E-296	0.653	0.265	0.001	1.34E-291	PA-4
Sgo1	3.12E-293	0.726	0.292	0.002	6.23E-289	PA-4
Kif14	2.53E-290	0.782	0.31	0.003	5.06E-286	PA-4
Ckap2	1.07E-284	1.221	0.425	0.008	2.14E-280	PA-4
Sgo2a	5.38E-283	0.928	0.354	0.004	1.07E-278	PA-4
Kifc1	9.94E-279	0.698	0.31	0.003	1.98E-274	PA-4
Dlgap5	9.06E-278	0.717	0.274	0.002	1.81E-273	PA-4
Birc5	2.92E-276	0.542	0.257	0.001	5.82E-272	PA-4
Dtl	1.04E-272	1.238	0.398	0.007	2.08E-268	PA-4
Aspm	3.29E-269	0.993	0.319	0.003	6.57E-265	PA-4
Nuf2	1.61E-268	0.570	0.257	0.001	3.21E-264	PA-4
Cep55	2.33E-266	0.720	0.265	0.002	4.66E-262	PA-4
Ndc80	1.35E-258	0.727	0.301	0.003	2.70E-254	PA-4
Spag5	8.74E-255	0.714	0.257	0.002	1.75E-250	PA-4
Trim59	1.99E-252	0.763	0.292	0.003	3.98E-248	PA-4
Ect2	7.32E-246	1.613	0.531	0.018	1.46E-241	PA-4
Tk1	1.79E-242	0.543	0.257	0.002	3.57E-238	PA-4
Anln	5.92E-242	2.086	0.628	0.028	1.18E-237	PA-4
Kif20b	1.34E-232	1.221	0.469	0.014	2.67E-228	PA-4
Uhrf1	2.24E-231	0.611	0.265	0.003	4.46E-227	PA-4
Cenpk	1.03E-227	1.124	0.522	0.019	2.05E-223	PA-4
Mastl	3.23E-226	1.257	0.504	0.018	6.44E-222	PA-4
Gen1	7.41E-221	0.865	0.336	0.006	1.48E-216	PA-4
Ccdc18	8.56E-218	0.922	0.292	0.004	1.71E-213	PA-4
Kif22	1.23E-212	0.804	0.265	0.003	2.45E-208	PA-4
Fanci	3.40E-209	0.761	0.301	0.005	6.78E-205	PA-4
Prr11	2.10E-206	0.648	0.257	0.003	4.19E-202	PA-4
Cdca2	1.21E-204	1.170	0.469	0.017	2.42E-200	PA-4
Bard1	2.19E-203	1.238	0.416	0.013	4.37E-199	PA-4
Fanca	1.48E-201	0.859	0.372	0.01	2.96E-197	PA-4
Polq	2.22E-201	0.895	0.389	0.011	4.44E-197	PA-4
Stil	3.05E-198	0.907	0.398	0.012	6.08E-194	PA-4
Rmi2	2.85E-194	0.897	0.31	0.006	5.68E-190	PA-4
Cip2a	5.26E-191	0.729	0.283	0.005	1.05E-186	PA-4
Clspn	1.68E-183	0.956	0.372	0.011	3.34E-179	PA-4
Fignl1	1.04E-175	0.603	0.274	0.005	2.08E-171	PA-4
Ncapg2	3.25E-168	1.759	0.619	0.043	6.48E-164	PA-4

Incenp	9.13E-163	1.002	0.416	0.018	1.82E-158	PA-4
Pole	1.76E-160	1.030	0.434	0.02	3.51E-156	PA-4
Slfn9	2.50E-160	0.797	0.319	0.009	5.00E-156	PA-4
Brip1	2.46E-152	1.709	0.575	0.04	4.92E-148	PA-4
Rad51	1.71E-150	1.000	0.398	0.017	3.41E-146	PA-4
Ccnf	4.88E-149	0.835	0.283	0.008	9.74E-145	PA-4
Diaph3	1.24E-148	3.489	0.965	0.159	2.48E-144	PA-4
Dbf4	1.25E-145	0.799	0.319	0.011	2.49E-141	PA-4
Espl1	9.09E-133	0.685	0.274	0.009	1.81E-128	PA-4
Smc2	4.50E-130	1.129	0.513	0.037	8.99E-126	PA-4
Mms22l	2.00E-129	1.868	0.664	0.067	3.99E-125	PA-4
Nusap1	2.30E-114	1.076	0.381	0.022	4.60E-110	PA-4
Tacc3	1.50E-112	0.746	0.283	0.012	2.99E-108	PA-4
Ncapd2	3.12E-107	1.191	0.504	0.044	6.22E-103	PA-4
Blm	3.38E-103	1.001	0.469	0.038	6.74E-99	PA-4
Kif24	1.87E-101	1.259	0.549	0.055	3.73E-97	PA-4
Wdhd1	2.35E-100	1.050	0.425	0.032	4.70E-96	PA-4
Zgrf1	4.21E-95	1.230	0.522	0.053	8.39E-91	PA-4
Atad2	6.74E-90	1.911	0.796	0.151	1.34E-85	PA-4
Nav2	4.44E-86	1.364	0.363	0.028	8.85E-82	PA-4
Cenpp	2.66E-85	2.590	0.858	0.204	5.31E-81	PA-4
Smc4	1.23E-84	1.657	0.664	0.104	2.46E-80	PA-4
Zfp367	2.02E-84	0.811	0.319	0.022	4.04E-80	PA-4
Brca2	6.55E-83	0.838	0.381	0.032	1.31E-78	PA-4
Plk4	1.64E-79	0.570	0.301	0.02	3.27E-75	PA-4
Ezh2	7.53E-73	1.928	0.814	0.191	1.50E-68	PA-4
Fancd2	1.02E-70	0.683	0.327	0.027	2.04E-66	PA-4
BC055324	5.91E-69	0.604	0.274	0.019	1.18E-64	PA-4
Mybl1	2.02E-67	0.831	0.389	0.041	4.03E-63	PA-4
Atad5	1.90E-66	1.126	0.478	0.062	3.79E-62	PA-4
Lig1	1.14E-64	0.699	0.301	0.026	2.28E-60	PA-4
Rrm1	1.66E-63	1.092	0.522	0.078	3.31E-59	PA-4
Tyms	7.31E-63	0.878	0.381	0.042	1.46E-58	PA-4
Rad18	3.70E-61	1.303	0.637	0.121	7.39E-57	PA-4
E2f7	5.98E-60	1.052	0.575	0.096	1.19E-55	PA-4
Hells	1.08E-59	1.226	0.531	0.084	2.15E-55	PA-4
Ndc1	8.07E-55	0.690	0.354	0.041	1.61E-50	PA-4
Prim2	3.89E-52	1.555	0.779	0.227	7.76E-48	PA-4
Zranb3	4.80E-52	0.739	0.336	0.039	9.59E-48	PA-4
Nsd2	3.46E-50	1.705	0.85	0.315	6.91E-46	PA-4
Cep128	2.52E-49	1.654	0.823	0.28	5.03E-45	PA-4
Pola1	4.49E-49	1.933	0.841	0.326	8.96E-45	PA-4
Cdc7	5.50E-49	0.913	0.469	0.078	1.10E-44	PA-4
Prim1	4.00E-47	0.701	0.274	0.029	7.98E-43	PA-4
Kif18a	1.37E-45	0.660	0.292	0.034	2.74E-41	PA-4
Rbl1	1.58E-45	1.031	0.54	0.109	3.15E-41	PA-4
Fbxo5	2.76E-45	0.614	0.257	0.026	5.50E-41	PA-4
Tmpo	3.45E-44	1.455	0.77	0.255	6.88E-40	PA-4
Cep57l1	2.01E-42	0.869	0.478	0.091	4.02E-38	PA-4
Bora	1.50E-41	0.748	0.274	0.033	3.00E-37	PA-4
Ncam1	2.76E-41	1.134	0.265	0.031	5.52E-37	PA-4
Rangap1	7.08E-41	0.646	0.372	0.058	1.41E-36	PA-4
Lmnb1	4.12E-39	1.184	0.611	0.165	8.22E-35	PA-4
Mthfd1l	2.96E-38	0.940	0.434	0.083	5.92E-34	PA-4
Haus6	8.28E-38	0.774	0.451	0.089	1.65E-33	PA-4
Ncapd3	1.85E-37	0.916	0.487	0.107	3.68E-33	PA-4
Pbx3	9.67E-36	1.326	0.885	0.396	1.93E-31	PA-4
Topbp1	1.20E-35	1.050	0.584	0.159	2.40E-31	PA-4
Hjurp	2.26E-35	1.366	0.796	0.36	4.50E-31	PA-4
Nemp1	3.13E-35	0.512	0.327	0.051	6.24E-31	PA-4
Dnmt1	3.72E-34	0.658	0.398	0.077	7.43E-30	PA-4
Ska2	1.67E-33	0.717	0.442	0.094	3.34E-29	PA-4

Cep192	5.71E-33	1.021	0.681	0.219	1.14E-28	PA-4
Cchcr1	4.52E-32	0.709	0.381	0.075	9.03E-28	PA-4
Pkp4	1.80E-31	0.646	0.381	0.074	3.59E-27	PA-4
Mcm6	4.37E-31	0.587	0.265	0.04	8.73E-27	PA-4
Hat1	1.45E-30	0.901	0.531	0.139	2.90E-26	PA-4
Cep152	1.87E-30	0.738	0.451	0.104	3.74E-26	PA-4
Skp2	7.42E-30	0.615	0.354	0.069	1.48E-25	PA-4
Nup155	1.80E-29	0.719	0.54	0.144	3.60E-25	PA-4
Polh	3.73E-29	0.695	0.381	0.081	7.44E-25	PA-4
Tenm3	7.21E-29	1.131	0.301	0.054	1.44E-24	PA-4
Sass6	2.02E-28	0.508	0.283	0.048	4.02E-24	PA-4
Bcat1	3.33E-28	0.716	0.389	0.085	6.64E-24	PA-4
Arnt2	3.44E-28	0.648	0.292	0.051	6.86E-24	PA-4
Wdr76	4.29E-28	0.666	0.319	0.061	8.56E-24	PA-4
BC052040	7.62E-28	0.899	0.628	0.194	1.52E-23	PA-4
Cdh2	2.58E-27	1.078	0.381	0.087	5.14E-23	PA-4
Ranbp17	2.75E-27	1.109	0.549	0.171	5.50E-23	PA-4
Rad51b.2	1.11E-26	1.210	0.938	0.607	2.21E-22	PA-4
Rfwd3	1.69E-26	0.715	0.496	0.135	3.37E-22	PA-4
Lin54	5.57E-26	0.695	0.451	0.117	1.11E-21	PA-4
Pde3b	4.48E-25	0.563	0.363	0.081	8.93E-21	PA-4
Rttn	6.58E-25	0.735	0.54	0.164	1.31E-20	PA-4
Sdk1.3	1.10E-24	0.948	1	0.913	2.20E-20	PA-4
Map4	1.34E-24	0.974	0.929	0.662	2.68E-20	PA-4
Mtbp	1.58E-24	0.574	0.345	0.076	3.15E-20	PA-4
Platr25	3.40E-24	0.869	0.522	0.168	6.79E-20	PA-4
Cd44	3.93E-24	1.589	0.619	0.24	7.85E-20	PA-4
Cbx5	1.18E-23	1.059	0.726	0.322	2.36E-19	PA-4
Cdk2	1.49E-23	0.518	0.283	0.056	2.98E-19	PA-4
Lhfpl2	2.18E-23	0.828	0.602	0.217	4.36E-19	PA-4
Plcb1.1	2.72E-23	1.086	0.637	0.235	5.42E-19	PA-4
Gjc1	9.72E-23	0.776	0.469	0.141	1.94E-18	PA-4
Lbr	1.68E-22	0.521	0.407	0.106	3.34E-18	PA-4
Cntln	3.92E-22	0.984	0.876	0.469	7.83E-18	PA-4
Rora.2	7.40E-22	-1.061	0.965	0.978	1.48E-17	PA-4
Dcn.1	8.34E-22	-1.396	0.92	0.96	1.67E-17	PA-4
Lin9	2.07E-21	0.503	0.265	0.053	4.13E-17	PA-4
Rpa1	2.14E-21	0.676	0.425	0.12	4.27E-17	PA-4
Celf2	2.46E-21	-0.671	1	0.997	4.91E-17	PA-4
Myh9.1	2.91E-21	0.818	0.637	0.242	5.81E-17	PA-4
Vrk1	4.33E-21	0.631	0.372	0.099	8.63E-17	PA-4
Zdhhc15	4.64E-21	0.546	0.292	0.064	9.26E-17	PA-4
Suz12	9.75E-21	0.807	0.699	0.293	1.95E-16	PA-4
Rbbp8	2.20E-20	0.786	0.513	0.181	4.39E-16	PA-4
Serping1.1	2.66E-20	-1.556	0.566	0.786	5.30E-16	PA-4
Postn.2	1.16E-19	1.050	0.805	0.407	2.31E-15	PA-4
Dot1l	2.00E-19	0.554	0.442	0.136	3.99E-15	PA-4
Ubap2	3.27E-19	0.767	0.681	0.291	6.52E-15	PA-4
G2e3	3.47E-19	0.906	0.54	0.211	6.93E-15	PA-4
Tmtc2.3	4.16E-19	1.481	0.655	0.303	8.30E-15	PA-4
Dek	4.37E-19	0.689	0.513	0.185	8.72E-15	PA-4
Mcph1	4.39E-19	0.751	0.584	0.221	8.76E-15	PA-4
Sae1	6.56E-19	0.856	0.628	0.267	1.31E-14	PA-4
Cdk5rap2	6.97E-19	0.846	0.611	0.257	1.39E-14	PA-4
Cep85	8.55E-19	0.576	0.416	0.125	1.71E-14	PA-4
Taco1	1.38E-18	1.130	0.796	0.478	2.76E-14	PA-4
Ccdc82	1.94E-18	0.731	0.637	0.267	3.87E-14	PA-4
Pold3	2.64E-18	0.636	0.451	0.146	5.27E-14	PA-4
Xpo1	2.93E-18	0.835	0.681	0.307	5.86E-14	PA-4
Lcorl	4.05E-18	0.966	0.726	0.384	8.09E-14	PA-4
E2f3	5.31E-18	0.732	0.496	0.175	1.06E-13	PA-4
Trim37	1.17E-17	0.832	0.451	0.156	2.34E-13	PA-4

Tfdp1	1.30E-17	0.561	0.407	0.128	2.60E-13	PA-4
Mdc1	2.62E-17	0.507	0.292	0.075	5.24E-13	PA-4
Naaladl2	2.74E-17	-0.914	0.965	0.967	5.47E-13	PA-4
Fam111a	5.46E-17	0.532	0.319	0.09	1.09E-12	PA-4
Ptprs	9.02E-17	0.645	0.593	0.242	1.80E-12	PA-4
Itgb3bp	1.09E-16	0.588	0.496	0.177	2.17E-12	PA-4
Cep112	1.27E-16	0.831	0.885	0.647	2.53E-12	PA-4
Sh3pxd2a.2	1.31E-16	0.666	0.513	0.194	2.61E-12	PA-4
Usp37	1.34E-16	0.752	0.637	0.289	2.68E-12	PA-4
Rad21	1.40E-16	0.704	0.558	0.232	2.79E-12	PA-4
Gsn.3	1.88E-16	-1.645	0.77	0.913	3.75E-12	PA-4
Xdh	2.50E-16	-1.045	0.549	0.788	4.99E-12	PA-4
Tgfbr3	2.80E-16	-0.864	0.965	0.974	5.59E-12	PA-4
Mast2	4.20E-16	0.741	0.85	0.527	8.38E-12	PA-4
Nup160	8.34E-16	0.605	0.522	0.207	1.66E-11	PA-4
1700025G04Ri	1.21E-15	0.947	0.673	0.34	2.42E-11	PA-4
Cacna1c.3	1.47E-15	1.190	0.54	0.238	2.94E-11	PA-4
Tipin	1.87E-15	0.624	0.381	0.127	3.74E-11	PA-4
Ckap5	1.92E-15	0.933	0.69	0.404	3.82E-11	PA-4
Nup107	2.27E-15	0.565	0.354	0.111	4.53E-11	PA-4
Lama2.2	2.99E-15	-0.810	0.965	0.975	5.96E-11	PA-4
4930503L19Rik	3.60E-15	0.577	0.46	0.168	7.19E-11	PA-4
Slx4ip	5.32E-15	0.604	0.478	0.179	1.06E-10	PA-4
Smc6	5.33E-15	0.612	0.841	0.466	1.06E-10	PA-4
Hpf1	7.72E-15	0.592	0.416	0.147	1.54E-10	PA-4
Piezo1	8.28E-15	0.560	0.301	0.09	1.65E-10	PA-4
Psd3.3	1.00E-14	0.735	0.991	0.905	2.00E-10	PA-4
Zbtb16	1.07E-14	-0.779	0.876	0.953	2.14E-10	PA-4
Col8a1.1	1.27E-14	0.837	0.478	0.19	2.54E-10	PA-4
Adam12.2	1.53E-14	1.194	0.504	0.216	3.05E-10	PA-4
Hipk2	1.80E-14	0.733	0.558	0.244	3.60E-10	PA-4
Ccdc138	2.73E-14	0.567	0.425	0.152	5.45E-10	PA-4
Trabd2b	3.51E-14	0.684	0.292	0.086	7.01E-10	PA-4
Mitf	5.71E-14	-1.115	0.593	0.771	1.14E-09	PA-4
Ccdc34	5.83E-14	0.649	0.389	0.136	1.16E-09	PA-4
Casp8ap2	7.63E-14	0.539	0.531	0.215	1.52E-09	PA-4
Nucks1	2.08E-13	0.563	0.575	0.254	4.16E-09	PA-4
Mfhas1	3.96E-13	0.649	0.726	0.394	7.90E-09	PA-4
Arhgap24.3	4.50E-13	-1.076	0.823	0.904	8.98E-09	PA-4
Rif1	4.85E-13	0.719	0.664	0.341	9.68E-09	PA-4
Eln.2	5.39E-13	-1.603	0.381	0.65	1.08E-08	PA-4
Gmds	5.55E-13	0.662	0.947	0.707	1.11E-08	PA-4
Sept10	5.81E-13	0.706	0.416	0.169	1.16E-08	PA-4
Slbp	6.63E-13	0.629	0.363	0.13	1.32E-08	PA-4
Smchd1	8.49E-13	0.692	0.796	0.457	1.70E-08	PA-4
Ints7	1.06E-12	0.673	0.407	0.162	2.12E-08	PA-4
Galnt18	1.65E-12	0.580	0.46	0.186	3.29E-08	PA-4
Rcc1	1.80E-12	0.506	0.425	0.163	3.60E-08	PA-4
Cobll1	2.09E-12	0.640	0.301	0.098	4.18E-08	PA-4
Cbfb	3.33E-12	0.692	0.69	0.388	6.64E-08	PA-4
Sfmbt1	5.55E-12	0.659	0.619	0.306	1.11E-07	PA-4
Spidr	6.00E-12	0.647	0.77	0.485	1.20E-07	PA-4
Sept11.2	6.10E-12	0.658	0.673	0.35	1.22E-07	PA-4
Smc5	6.89E-12	0.675	0.77	0.435	1.38E-07	PA-4
Ror2.1	7.76E-12	0.595	0.664	0.335	1.55E-07	PA-4
Ccdc88a	8.41E-12	0.569	0.903	0.632	1.68E-07	PA-4
Rras2.3	1.27E-11	0.906	0.681	0.435	2.53E-07	PA-4
Ctdspl2	1.45E-11	0.598	0.699	0.392	2.89E-07	PA-4
Srbd1	1.53E-11	0.576	0.496	0.22	3.05E-07	PA-4
Mill2	1.55E-11	-0.955	0.407	0.654	3.09E-07	PA-4
Abca8a.3	1.98E-11	-1.332	0.469	0.685	3.95E-07	PA-4
Man1a	2.05E-11	-0.665	0.973	0.965	4.09E-07	PA-4

Exoc4	2.70E-11	0.500	0.982	0.903	5.39E-07	PA-4
Slfn5	2.86E-11	-1.029	0.221	0.513	5.70E-07	PA-4
Frmd4a.2	2.91E-11	0.716	0.602	0.317	5.80E-07	PA-4
Ext1.2	2.91E-11	0.554	1	0.959	5.81E-07	PA-4
Egfr.2	3.01E-11	-0.708	0.938	0.969	6.00E-07	PA-4
Terf1	3.20E-11	0.602	0.522	0.246	6.39E-07	PA-4
Scara5.2	3.23E-11	-0.859	0.699	0.842	6.45E-07	PA-4
Ccdc141	3.63E-11	0.611	0.416	0.178	7.25E-07	PA-4
Lvrn.2	3.70E-11	-1.344	0.283	0.565	7.38E-07	PA-4
Baz1b	4.09E-11	0.617	0.779	0.5	8.17E-07	PA-4
Plpp3.3	5.57E-11	-0.958	0.805	0.882	1.11E-06	PA-4
Tnxb	5.89E-11	-0.644	0.965	0.948	1.18E-06	PA-4
Cdk14	7.52E-11	0.522	0.973	0.876	1.50E-06	PA-4
Jazf1.2	9.33E-11	0.630	0.504	0.235	1.86E-06	PA-4
Slf1	9.40E-11	0.618	0.558	0.277	1.88E-06	PA-4
Col5a3.2	9.69E-11	0.974	0.752	0.484	1.93E-06	PA-4
mt-Rnr1	1.01E-10	1.115	0.938	0.908	2.02E-06	PA-4
Csgalnact1.2	1.05E-10	0.633	0.726	0.412	2.09E-06	PA-4
Chsy3.1	1.06E-10	0.587	0.522	0.241	2.12E-06	PA-4
Smarcc1	1.09E-10	0.575	0.673	0.373	2.18E-06	PA-4
Ablim1.2	1.15E-10	-0.791	0.814	0.9	2.29E-06	PA-4
Mtf2	1.33E-10	0.518	0.566	0.269	2.66E-06	PA-4
Psmd14	1.68E-10	0.557	0.77	0.443	3.36E-06	PA-4
Aebp1.1	1.87E-10	-1.313	0.31	0.548	3.74E-06	PA-4
Galnt15	2.37E-10	-1.039	0.204	0.481	4.73E-06	PA-4
Supt3	2.43E-10	0.606	0.646	0.359	4.85E-06	PA-4
Timp2.2	2.76E-10	-1.225	0.513	0.681	5.50E-06	PA-4
Rnase4	2.85E-10	-1.146	0.177	0.44	5.68E-06	PA-4
Runx2	3.20E-10	0.686	0.345	0.136	6.39E-06	PA-4
Pid1.2	4.24E-10	-1.066	0.566	0.763	8.46E-06	PA-4
Taf1d	5.55E-10	0.522	0.496	0.24	1.11E-05	PA-4
Mast4.1	5.83E-10	0.527	1	0.941	1.16E-05	PA-4
Vcan.2	6.07E-10	0.586	0.929	0.688	1.21E-05	PA-4
Abca6.1	6.09E-10	-1.105	0.372	0.573	1.21E-05	PA-4
AW554918	6.71E-10	0.590	0.85	0.581	1.34E-05	PA-4
Srsf10	9.27E-10	0.544	0.566	0.296	1.85E-05	PA-4
Vrk2	9.83E-10	0.528	0.743	0.415	1.96E-05	PA-4
Slc10a7	1.00E-09	0.530	0.788	0.489	2.00E-05	PA-4
Clec2d	1.12E-09	-0.827	0.628	0.729	2.24E-05	PA-4
Abca1	1.21E-09	-0.780	0.637	0.756	2.41E-05	PA-4
Cenpc1	1.35E-09	0.542	0.584	0.301	2.70E-05	PA-4
Rps6ka5	1.40E-09	0.536	0.513	0.254	2.79E-05	PA-4
Ccnd3	1.80E-09	-0.965	0.319	0.547	3.60E-05	PA-4
Kdm7a.1	2.09E-09	-0.860	0.372	0.599	4.16E-05	PA-4
Iqgap2	2.13E-09	0.715	0.575	0.35	4.26E-05	PA-4
Asxl1	2.37E-09	0.518	0.726	0.441	4.73E-05	PA-4
Chn2	2.55E-09	0.685	0.363	0.159	5.09E-05	PA-4
Memo1	2.71E-09	0.652	0.717	0.447	5.41E-05	PA-4
Dpep1.1	3.05E-09	-1.026	0.248	0.475	6.08E-05	PA-4
C3.2	3.08E-09	-1.441	0.345	0.563	6.15E-05	PA-4
Flnb.3	3.58E-09	0.600	0.841	0.637	7.14E-05	PA-4
Dapk1	4.84E-09	-0.681	0.885	0.914	9.66E-05	PA-4
Lrp1	7.53E-09	-0.718	0.69	0.775	0.000150377	PA-4
Pds5a	7.65E-09	0.572	0.85	0.589	0.000152727	PA-4
Htr2a	7.70E-09	0.524	0.274	0.102	0.000153716	PA-4
Itga9	8.49E-09	-0.848	0.301	0.528	0.000169363	PA-4
Tcerg1	1.03E-08	0.534	0.681	0.401	0.000205871	PA-4
Clic4	1.04E-08	0.541	0.619	0.364	0.000208534	PA-4
Ifi207.2	1.06E-08	-0.851	0.442	0.611	0.000212237	PA-4
Vcl	1.56E-08	0.600	0.522	0.292	0.000311709	PA-4
Prkg1	1.63E-08	-0.941	0.735	0.801	0.000325923	PA-4
Errfi1.2	1.66E-08	-1.025	0.531	0.686	0.000330784	PA-4

Clec3b.1	1.66E-08	-0.936	0.743	0.84	0.000331419	PA-4
Actn4	1.75E-08	0.507	0.522	0.277	0.000349362	PA-4
Jag1.3	1.95E-08	0.699	0.628	0.393	0.000389606	PA-4
Igfbp4.2	1.97E-08	-1.005	0.212	0.446	0.000393024	PA-4
Elovl6	2.09E-08	0.512	0.345	0.154	0.000417757	PA-4
Syne2	2.22E-08	0.526	0.805	0.559	0.000442349	PA-4
Arl15	2.33E-08	-0.631	0.912	0.938	0.000465338	PA-4
C4b.1	3.39E-08	-1.266	0.097	0.322	0.000675667	PA-4
Pcolce2.2	3.39E-08	-0.831	0.531	0.671	0.000676935	PA-4
mt-Cytb.2	4.02E-08	0.834	0.956	0.925	0.000803123	PA-4
Dclk1.1	4.41E-08	0.608	0.858	0.673	0.00087965	PA-4
Procr.3	4.63E-08	-0.975	0.124	0.347	0.000924391	PA-4
Pds5b	4.68E-08	0.534	0.796	0.588	0.000934979	PA-4
Pcsk5	5.26E-08	-1.025	0.54	0.68	0.001050543	PA-4
Nr3c2	5.47E-08	-1.063	0.195	0.419	0.001091596	PA-4
Adgrg2.3	8.03E-08	0.630	0.681	0.453	0.001602234	PA-4
Il1rl2.2	1.13E-07	-0.863	0.566	0.684	0.002258077	PA-4
C1s1	1.33E-07	-0.758	0.566	0.671	0.00265164	PA-4
Cped1.2	1.40E-07	-0.587	0.929	0.922	0.002788039	PA-4
Maml3.3	1.59E-07	-0.982	0.469	0.602	0.003170946	PA-4
Serpinf1.1	1.80E-07	-0.900	0.416	0.584	0.003595837	PA-4
Sclt1	1.81E-07	0.579	0.628	0.366	0.003605725	PA-4
Ecm1.1	1.84E-07	-0.830	0.372	0.587	0.003678111	PA-4
Opcml.2	2.20E-07	-0.677	0.903	0.899	0.004398971	PA-4
mt-Rnr2.1	2.22E-07	0.845	0.938	0.91	0.004431342	PA-4
Gulp1	2.37E-07	-0.654	0.832	0.863	0.00472517	PA-4
Cblb	2.44E-07	-0.558	0.903	0.896	0.004874534	PA-4
Svep1.3	2.47E-07	-1.063	0.46	0.612	0.004925402	PA-4
Pdgfc.3	2.52E-07	0.779	0.673	0.471	0.00502737	PA-4
mt-Atp6.1	2.74E-07	0.861	0.956	0.955	0.005475917	PA-4
Thsd7a.1	2.87E-07	-0.851	0.761	0.817	0.005718792	PA-4
mt-Co2.2	3.76E-07	0.834	0.938	0.948	0.007499351	PA-4
Ace	4.02E-07	-0.790	0.133	0.353	0.008015742	PA-4
Kpna3	4.96E-07	0.528	0.575	0.339	0.009904314	PA-4
Lamc1	5.99E-07	-0.544	0.876	0.92	0.011964596	PA-4
Ltbp1.2	6.98E-07	-0.584	0.814	0.857	0.01393083	PA-4
mt-Atp8	8.15E-07	0.513	0.619	0.415	0.016264665	PA-4
Pi16.3	1.05E-06	-1.249	0.708	0.725	0.020998744	PA-4
Rsrp1	1.17E-06	-0.582	0.708	0.744	0.023267159	PA-4
Nova1.2	1.20E-06	-0.657	0.956	0.926	0.024041618	PA-4
Ogn.2	1.22E-06	-0.922	0.558	0.62	0.024281025	PA-4
Nckap5.3	1.23E-06	-0.999	0.451	0.578	0.024593578	PA-4
Thrb.2	1.27E-06	-0.894	0.434	0.566	0.025355604	PA-4
Dlgap4	1.49E-06	-0.620	0.398	0.566	0.029783609	PA-4
mt-Co3.2	1.63E-06	0.705	0.973	0.956	0.032479201	PA-4
Stxbp6.3	2.08E-06	0.536	0.867	0.695	0.041581626	PA-4
Mmp2	2.09E-06	-0.758	0.416	0.56	0.041752635	PA-4
Klf9	2.24E-06	-0.720	0.407	0.553	0.044643874	PA-4
Acss3	2.36E-06	-0.656	0.46	0.623	0.047139451	PA-4
Kdm5b	2.40E-06	-0.685	0.221	0.401	0.047820268	PA-4
Fndc1.3	2.41E-06	-0.556	0.885	0.918	0.04807402	PA-4
Adarb1	2.49E-06	-0.674	0.239	0.449	0.049743018	PA-4
C7.3	3.74E-06	-2.262	0.31	0.462	0.074603709	PA-4
Pik3r1.2	3.98E-06	-0.692	0.637	0.7	0.079473767	PA-4
Tmem178b	4.08E-06	-0.922	0.195	0.38	0.081515644	PA-4
Sh3d19	4.16E-06	-0.519	0.832	0.862	0.082958247	PA-4
Crebrf	4.17E-06	-0.668	0.372	0.534	0.083296083	PA-4
Rb1cc1	4.73E-06	-0.546	0.584	0.685	0.094436685	PA-4
Igfbp6.2	5.07E-06	-1.030	0.434	0.584	0.101242718	PA-4
Palm	5.44E-06	-0.598	0.425	0.563	0.108589928	PA-4
Nox4	5.57E-06	-0.874	0.876	0.857	0.111249231	PA-4
Hmcn2.3	5.68E-06	-1.176	0.124	0.317	0.113346474	PA-4

Itgbl1.3	5.85E-06	-1.117	0.619	0.703	0.116830676	PA-4
C1ra	5.90E-06	-0.762	0.204	0.376	0.117853772	PA-4
mt-Nd1.2	6.78E-06	0.740	0.903	0.916	0.135396874	PA-4
Samhd1.3	7.63E-06	-0.698	0.628	0.709	0.152386558	PA-4
Pced1b	8.58E-06	-0.722	0.177	0.357	0.171321575	PA-4
mt-Co1.1	9.26E-06	0.623	0.965	0.962	0.184831244	PA-4
Slc8a1.1	9.34E-06	0.604	0.814	0.669	0.186328151	PA-4
mt-Nd6.1	9.57E-06	0.558	0.619	0.432	0.190941525	PA-4
Ugdh.3	1.05E-05	0.577	0.77	0.564	0.209838472	PA-4
Itga11.2	1.82E-05	-0.744	0.345	0.5	0.36298212	PA-4
mt-Nd4.1	1.86E-05	0.606	0.92	0.91	0.370741955	PA-4
Sfrp2.2	1.90E-05	-0.880	0.088	0.267	0.379477318	PA-4
Itm2b	2.18E-05	-1.049	0.195	0.346	0.435060624	PA-4
Scn7a	2.57E-05	-0.591	0.602	0.695	0.513254155	PA-4
Apod.1	2.66E-05	-0.802	0.177	0.343	0.53042724	PA-4
Pdgfra.1	3.43E-05	-0.628	0.619	0.683	0.685410607	PA-4
Tek.3	3.82E-05	-0.756	0.637	0.661	0.76162131	PA-4
Ly6a.2	3.87E-05	-0.947	0.54	0.591	0.773164743	PA-4
Txnip	4.32E-05	-0.748	0.23	0.38	0.861898347	PA-4
Dpp4.3	4.93E-05	-0.747	0.858	0.785	0.983856315	PA-4
Abi3bp.2	5.03E-05	-0.657	0.805	0.835	1	PA-4
Fam129a	5.25E-05	-0.570	0.628	0.69	1	PA-4
Ahnak	6.30E-05	-0.513	0.823	0.841	1	PA-4
Foxn3	7.80E-05	-0.511	0.69	0.78	1	PA-4
Islr	9.30E-05	-0.616	0.177	0.336	1	PA-4
Aff1	9.34E-05	-0.506	0.628	0.698	1	PA-4
Ube2h	0.0001128	-0.510	0.593	0.651	1	PA-4
Lmbrd1	0.000114	-0.592	0.257	0.397	1	PA-4
Mxd4	0.0001348	-0.534	0.221	0.371	1	PA-4
Adamts6	0.000136	0.866	0.389	0.252	1	PA-4
Zfp36l1	0.0001602	-0.584	0.451	0.529	1	PA-4
Rarres2	0.0001797	-0.881	0.195	0.321	1	PA-4
Ahnak2	0.0001946	-0.639	0.257	0.381	1	PA-4
Papss2	0.0001958	-0.728	0.372	0.492	1	PA-4
Ppp2r2b.2	0.0001976	-0.777	0.274	0.431	1	PA-4
Fbxl20	0.0001986	-0.546	0.381	0.506	1	PA-4
Ezh1	0.0002079	-0.507	0.212	0.362	1	PA-4
Maf.1	0.0002114	-0.510	0.487	0.592	1	PA-4
Dock9.2	0.0002354	-0.716	0.372	0.477	1	PA-4
Klf4.2	0.0002424	-0.653	0.398	0.509	1	PA-4
Susd6	0.0002462	-0.585	0.531	0.588	1	PA-4
Nrip1.2	0.000288	-0.537	0.743	0.745	1	PA-4
Fgf7.2	0.000289	-0.727	0.177	0.313	1	PA-4
Fgd4	0.0003303	-0.590	0.381	0.487	1	PA-4
Rps21.2	0.0003734	-1.005	0.221	0.345	1	PA-4
Ntrk2	0.0003979	-0.798	0.195	0.315	1	PA-4
Olfr1033.1	0.0004255	-0.597	0.195	0.342	1	PA-4
Lbp	0.0004366	-0.553	0.195	0.333	1	PA-4
Fbln1.2	0.0005371	-0.909	0.168	0.288	1	PA-4
Kmt5b	0.0005461	-0.510	0.407	0.504	1	PA-4
Rcan2	0.0006165	-0.586	0.584	0.651	1	PA-4
Anpep	0.0006787	-0.504	0.186	0.316	1	PA-4
Prkn	0.0007074	-0.584	0.664	0.663	1	PA-4
Ltbp4.2	0.0007515	-0.799	0.168	0.285	1	PA-4
Gm48582	0.0007532	-0.503	0.248	0.375	1	PA-4
Nr1h4.3	0.0009279	-0.688	0.292	0.404	1	PA-4
Prex2.2	0.0009379	-0.647	0.363	0.459	1	PA-4
Zdhhc17	0.0009658	-0.539	0.248	0.357	1	PA-4
Mta3	0.0010027	-0.510	0.381	0.476	1	PA-4
Gas1.3	0.0011128	-0.776	0.186	0.296	1	PA-4
Mecp2	0.0012691	-0.510	0.381	0.47	1	PA-4
Sfxn1	0.0014151	-0.550	0.204	0.308	1	PA-4

Pla1a.3	0.0014229	-0.595	0.611	0.603	1	PA-4
Lum.3	0.0014426	-0.732	0.15	0.258	1	PA-4
Acadsb	0.0015125	-0.505	0.389	0.475	1	PA-4
Dpt.3	0.0015325	-0.586	0.664	0.692	1	PA-4
Adamts16.3	0.0016425	-0.974	0.204	0.313	1	PA-4
Serpinb6a	0.0017101	-0.561	0.389	0.462	1	PA-4
Tom1	0.0017837	-0.517	0.257	0.362	1	PA-4
Pdcd4	0.0019937	-0.539	0.31	0.402	1	PA-4
Plcxd3.2	0.0019976	-1.278	0.221	0.327	1	PA-4
Angpt1.3	0.0021104	-0.660	0.301	0.421	1	PA-4
Gstm1	0.0021265	-0.559	0.15	0.256	1	PA-4
Epha3.3	0.0021642	-0.798	0.23	0.355	1	PA-4
Dnm3	0.0022166	-0.603	0.389	0.47	1	PA-4
Fat4	0.0022207	-0.565	0.159	0.271	1	PA-4
Abca8b	0.0028581	-0.708	0.381	0.474	1	PA-4
Lrmda	0.0028741	-0.558	0.743	0.737	1	PA-4
Deptor.3	0.0032665	-0.546	0.195	0.314	1	PA-4
Gfpt2.3	0.0034245	-0.520	0.779	0.773	1	PA-4
Runx1t1.1	0.0035222	-0.523	0.478	0.553	1	PA-4
Smpd3.3	0.0035975	-0.587	0.451	0.557	1	PA-4
Sntg1.3	0.0037272	-0.521	0.345	0.46	1	PA-4
Klhl13	0.0037474	-0.578	0.407	0.472	1	PA-4
Upp2	0.0038151	-0.603	0.283	0.372	1	PA-4
Sfrp4.1	0.003902	-0.798	0.283	0.366	1	PA-4
Ly6c1.2	0.0040522	-0.613	0.345	0.426	1	PA-4
Zfp652	0.0040823	-0.520	0.31	0.394	1	PA-4
Dab2.1	0.0044494	-0.562	0.204	0.297	1	PA-4
Adamtsl1.3	0.004631	0.556	0.903	0.899	1	PA-4
Dusp1	0.0052317	-0.522	0.186	0.285	1	PA-4
Cdh9.2	0.0075231	-0.539	0.265	0.37	1	PA-4
Fbln5	0.0076819	-0.507	0.257	0.34	1	PA-4
Fmo1.2	0.0081376	-0.616	0.177	0.27	1	PA-4
Pde1a	0.0096452	-0.590	0.319	0.401	1	PA-4
F13a1	0	3.433	0.724	0.036	0	PA-5
Mctp1	0	3.112	0.747	0.034	0	PA-5
Mrc1	0	2.762	0.652	0.015	0	PA-5
Mtss1	0	2.421	0.724	0.028	0	PA-5
Dock2	0	2.407	0.747	0.016	0	PA-5
Arhgap15	0	2.383	0.724	0.059	0	PA-5
Fli1	0	2.238	0.744	0.05	0	PA-5
Runx1	0	2.149	0.669	0.036	0	PA-5
March1	0	1.921	0.56	0.015	0	PA-5
Hdac9	0	1.907	0.539	0.017	0	PA-5
Ctsc	0	1.765	0.505	0.014	0	PA-5
Inpp5d	0	1.703	0.567	0.011	0	PA-5
Ptprc	0	1.596	0.481	0.007	0	PA-5
Fyb	0	1.573	0.522	0.01	0	PA-5
Myo1f	0	1.499	0.502	0.009	0	PA-5
Tbxas1	0	1.488	0.481	0.008	0	PA-5
Epsti1	0	1.482	0.437	0.013	0	PA-5
Adgre1	0	1.459	0.447	0.003	0	PA-5
Stab1	0	1.421	0.451	0.009	0	PA-5
Nrros	0	1.395	0.481	0.008	0	PA-5
Dock8	0	1.389	0.454	0.011	0	PA-5
Rbm47	0	1.378	0.471	0.012	0	PA-5
Shtn1	0	1.219	0.423	0.004	0	PA-5
Pstpip2	0	1.187	0.403	0.011	0	PA-5
Kcnk13	0	1.181	0.389	0.01	0	PA-5
Blnk	0	1.148	0.355	0.005	0	PA-5
Clec10a	0	1.146	0.365	0.003	0	PA-5
Gm21188	0	1.139	0.362	0.002	0	PA-5
Ikzf1	0	1.138	0.355	0.007	0	PA-5

Tm6sf1	0	1.119	0.396	0.002	0	PA-5
Lcp1	0	1.031	0.307	0.003	0	PA-5
Itga4	0	1.030	0.341	0.006	0	PA-5
Gng2	0	1.018	0.362	0.008	0	PA-5
Ly86	0	1.018	0.328	0.004	0	PA-5
Tnfrsf11a	0	1.008	0.345	0.005	0	PA-5
Cd84	0	0.964	0.317	0.003	0	PA-5
Nckap1l	0	0.927	0.294	0.003	0	PA-5
Cyth4	0	0.897	0.304	0.003	0	PA-5
Prkch	9.15E-306	1.479	0.403	0.014	1.83E-301	PA-5
Bank1	8.14E-301	1.207	0.338	0.008	1.62E-296	PA-5
Pik3ap1	3.95E-299	0.906	0.307	0.005	7.88E-295	PA-5
Apoe	1.54E-296	2.146	0.481	0.025	3.08E-292	PA-5
Ednrb	4.99E-294	1.364	0.355	0.01	9.95E-290	PA-5
St8sia4	2.21E-288	0.965	0.266	0.003	4.41E-284	PA-5
Arhgap45	1.60E-287	1.053	0.358	0.011	3.20E-283	PA-5
Tpd52	8.67E-272	0.897	0.294	0.006	1.73E-267	PA-5
Cd163	1.07E-268	1.761	0.413	0.019	2.13E-264	PA-5
Ms4a6c	1.41E-267	0.844	0.28	0.005	2.81E-263	PA-5
Arhgap26.1	1.98E-267	1.365	0.498	0.032	3.96E-263	PA-5
Mertk	4.92E-259	1.634	0.56	0.046	9.83E-255	PA-5
Stard8	7.75E-257	1.337	0.485	0.032	1.55E-252	PA-5
Rnf150	7.24E-255	2.072	0.614	0.059	1.44E-250	PA-5
Tec	8.58E-255	0.733	0.28	0.006	1.71E-250	PA-5
Prkcb	3.58E-247	1.082	0.406	0.021	7.14E-243	PA-5
Lyn	3.99E-247	1.896	0.666	0.076	7.96E-243	PA-5
Cd36	9.07E-244	2.608	0.457	0.03	1.81E-239	PA-5
Rcsd1	1.27E-243	0.869	0.297	0.008	2.54E-239	PA-5
B4galt6	8.83E-242	0.698	0.263	0.005	1.76E-237	PA-5
Otulinl	1.55E-240	0.728	0.28	0.007	3.09E-236	PA-5
Aoah	1.30E-239	1.109	0.321	0.011	2.59E-235	PA-5
Specc1	1.76E-236	1.351	0.42	0.025	3.51E-232	PA-5
Plcg2	1.18E-232	0.803	0.256	0.005	2.35E-228	PA-5
Alcam	3.76E-232	1.769	0.362	0.017	7.50E-228	PA-5
Slc43a2	1.23E-226	1.095	0.403	0.024	2.46E-222	PA-5
P3h2	2.34E-209	1.259	0.321	0.015	4.67E-205	PA-5
Nrp2	4.25E-204	1.253	0.41	0.029	8.49E-200	PA-5
Adap2	6.23E-201	1.428	0.478	0.042	1.24E-196	PA-5
Pik3r5	2.55E-195	0.674	0.253	0.008	5.09E-191	PA-5
Apobec1	2.62E-193	1.432	0.491	0.049	5.23E-189	PA-5
Ehd4	4.67E-192	1.207	0.416	0.034	9.32E-188	PA-5
Fgd6	2.74E-183	0.662	0.253	0.009	5.47E-179	PA-5
Cacna1d	1.38E-179	0.961	0.307	0.017	2.76E-175	PA-5
Plekhg5	4.11E-179	1.507	0.468	0.049	8.21E-175	PA-5
Plxna4	1.66E-178	1.507	0.372	0.028	3.32E-174	PA-5
Pltp	2.98E-170	1.060	0.273	0.013	5.95E-166	PA-5
Nedd9	9.48E-151	1.343	0.584	0.096	1.89E-146	PA-5
Epb41	3.96E-147	1.010	0.399	0.041	7.90E-143	PA-5
Unc93b1	1.12E-146	1.016	0.382	0.039	2.24E-142	PA-5
Ubash3b	6.40E-141	0.978	0.345	0.031	1.28E-136	PA-5
Tns3	7.13E-140	0.928	0.386	0.04	1.42E-135	PA-5
Fry	3.90E-136	1.499	0.355	0.035	7.79E-132	PA-5
Vav3.3	1.08E-134	1.742	0.819	0.229	2.16E-130	PA-5
Pde3b.1	1.52E-129	1.167	0.471	0.068	3.04E-125	PA-5
Lifr.2	4.09E-114	1.322	0.553	0.11	8.17E-110	PA-5
Trps1.2	1.76E-111	1.655	0.71	0.193	3.52E-107	PA-5
Prkcd	6.24E-110	0.772	0.331	0.038	1.25E-105	PA-5
Pepd	2.53E-103	1.097	0.44	0.077	5.04E-99	PA-5
Cdk6	3.08E-100	0.821	0.294	0.032	6.14E-96	PA-5
Plcl2.2	2.49E-99	1.468	0.659	0.182	4.97E-95	PA-5
Mamdc2	1.97E-96	1.130	0.307	0.038	3.94E-92	PA-5
Serinc3	4.22E-94	1.326	0.553	0.14	8.42E-90	PA-5

Myo5a.3	1.72E-93	1.556	0.7	0.229	3.44E-89	PA-5
Stk17b	6.37E-93	0.746	0.311	0.04	1.27E-88	PA-5
Nos1ap	1.19E-90	1.148	0.311	0.041	2.38E-86	PA-5
Dclre1c	4.10E-90	1.185	0.468	0.098	8.19E-86	PA-5
Snx8	5.88E-90	1.043	0.44	0.086	1.17E-85	PA-5
Tnfaip8	7.75E-90	1.096	0.444	0.086	1.55E-85	PA-5
Ripor2	6.59E-89	0.791	0.283	0.034	1.31E-84	PA-5
Sept9	8.75E-88	0.787	0.338	0.05	1.75E-83	PA-5
Plin2	1.22E-84	0.776	0.341	0.053	2.43E-80	PA-5
Pecam1	4.79E-84	2.002	0.273	0.035	9.56E-80	PA-5
Pip4k2a	7.72E-83	1.441	0.724	0.267	1.54E-78	PA-5
Rreb1.1	8.69E-82	1.626	0.826	0.404	1.74E-77	PA-5
Pkp4.1	1.58E-81	1.303	0.369	0.065	3.16E-77	PA-5
Dab2.2	4.43E-79	1.635	0.71	0.276	8.84E-75	PA-5
Gramd1b	1.76E-78	0.607	0.253	0.031	3.52E-74	PA-5
Fam49b	7.86E-78	1.202	0.676	0.226	1.57E-73	PA-5
Wwp1	9.89E-77	1.606	0.809	0.398	1.97E-72	PA-5
Pparg.2	2.16E-76	1.654	0.536	0.139	4.31E-72	PA-5
Pitpnc1.1	3.11E-76	1.409	0.823	0.384	6.21E-72	PA-5
Slc9a9.1	3.63E-75	1.624	0.877	0.518	7.25E-71	PA-5
Entpd1	2.70E-74	1.113	0.471	0.115	5.39E-70	PA-5
Arhgef6	1.61E-73	0.859	0.427	0.095	3.21E-69	PA-5
Elk3	5.61E-71	1.172	0.474	0.121	1.12E-66	PA-5
Mob3b	1.34E-70	0.890	0.413	0.09	2.68E-66	PA-5
Snx5	1.58E-70	1.017	0.522	0.149	3.16E-66	PA-5
Itprid2	1.48E-67	0.764	0.358	0.072	2.96E-63	PA-5
P2rx7	1.55E-65	0.628	0.331	0.062	3.10E-61	PA-5
Fbn1.3	3.05E-65	-1.512	0.867	0.981	6.09E-61	PA-5
Abhd12	4.42E-65	0.979	0.433	0.11	8.83E-61	PA-5
Rbpj	4.63E-65	2.333	0.874	0.658	9.24E-61	PA-5
Ap1b1	2.54E-62	0.760	0.341	0.07	5.08E-58	PA-5
Sh3kbp1.1	5.01E-62	1.085	0.706	0.294	1.00E-57	PA-5
Dgkd	1.45E-61	0.787	0.42	0.104	2.89E-57	PA-5
Sema3c.3	8.59E-60	-1.676	0.631	0.879	1.71E-55	PA-5
Frmd4b.2	3.23E-59	1.407	0.877	0.58	6.45E-55	PA-5
Epb41l1	3.65E-59	0.825	0.416	0.108	7.28E-55	PA-5
Gab3	3.91E-59	0.928	0.505	0.147	7.80E-55	PA-5
Nid1.2	9.47E-59	-1.228	0.727	0.912	1.89E-54	PA-5
Pde4d.2	4.45E-58	0.890	0.584	0.189	8.88E-54	PA-5
Mfap5.3	1.38E-57	-1.620	0.556	0.834	2.75E-53	PA-5
Atp10d.2	4.55E-56	0.949	0.546	0.179	9.08E-52	PA-5
Zdhhc14.2	3.85E-54	1.189	0.666	0.296	7.68E-50	PA-5
Zswim6	5.97E-54	1.196	0.843	0.504	1.19E-49	PA-5
Slc16a7	6.80E-54	0.804	0.427	0.116	1.36E-49	PA-5
Adgrd1.3	1.09E-53	-1.156	0.703	0.914	2.17E-49	PA-5
Arhgap39	2.43E-53	0.824	0.433	0.122	4.86E-49	PA-5
Dhrs3.3	1.36E-52	0.838	0.543	0.182	2.71E-48	PA-5
Dgkz	1.67E-52	0.647	0.307	0.067	3.33E-48	PA-5
Fstl1.1	4.11E-52	-0.997	0.867	0.967	8.20E-48	PA-5
Atp8a1	6.09E-52	0.891	0.416	0.117	1.22E-47	PA-5
Epas1	1.22E-51	0.754	0.297	0.062	2.44E-47	PA-5
N4bp2l1	1.27E-51	0.577	0.283	0.057	2.53E-47	PA-5
Sipa1l1	1.81E-51	0.844	0.464	0.14	3.60E-47	PA-5
Gas6.2	3.33E-51	0.736	0.478	0.141	6.64E-47	PA-5
Sdk1.4	3.99E-51	-1.156	0.727	0.924	7.96E-47	PA-5
Ddr2.3	4.82E-51	-1.007	0.778	0.953	9.63E-47	PA-5
Fndc1.4	5.37E-51	-1.224	0.713	0.927	1.07E-46	PA-5
Exoc6	1.12E-50	0.967	0.321	0.074	2.23E-46	PA-5
Rab31.2	1.19E-50	0.919	0.549	0.198	2.38E-46	PA-5
Slco2b1.3	1.64E-50	0.946	0.587	0.214	3.27E-46	PA-5
Cfh.2	2.17E-50	1.314	0.573	0.229	4.32E-46	PA-5
Myo1d.2	1.35E-49	1.108	0.372	0.098	2.69E-45	PA-5

Ebf1.2	2.06E-49	-0.908	0.99	0.998	4.11E-45	PA-5
Elmo1.1	3.12E-49	1.069	0.911	0.673	6.23E-45	PA-5
Arid5b.1	4.25E-49	1.041	0.631	0.26	8.47E-45	PA-5
Arhgap18.1	9.09E-49	1.098	0.754	0.375	1.82E-44	PA-5
Tnxb.1	1.30E-48	-0.964	0.829	0.954	2.59E-44	PA-5
Dpp4.4	1.92E-48	-1.543	0.502	0.8	3.83E-44	PA-5
Grb2	4.32E-48	0.912	0.618	0.26	8.63E-44	PA-5
Myh9.2	7.42E-48	0.925	0.577	0.233	1.48E-43	PA-5
Celf2.1	1.22E-47	-0.697	0.976	0.998	2.43E-43	PA-5
Map3k1	4.12E-47	0.878	0.399	0.117	8.22E-43	PA-5
Sgms1	4.37E-47	1.032	0.898	0.616	8.72E-43	PA-5
Mbnl1.1	5.19E-47	0.893	0.966	0.825	1.04E-42	PA-5
Gfpt2.4	1.55E-46	-1.451	0.461	0.788	3.10E-42	PA-5
Ldb2.3	2.99E-46	1.401	0.703	0.325	5.97E-42	PA-5
Rbms3	5.64E-46	-0.825	0.904	0.991	1.13E-41	PA-5
Opcml.3	6.82E-46	-1.284	0.717	0.907	1.36E-41	PA-5
Creb5.3	7.41E-46	-1.359	0.775	0.903	1.48E-41	PA-5
Casp8	1.70E-45	0.639	0.331	0.084	3.39E-41	PA-5
Cobll1.1	1.73E-45	0.783	0.348	0.089	3.46E-41	PA-5
Trim25	3.20E-45	0.725	0.362	0.1	6.39E-41	PA-5
Pi16.4	4.43E-45	-1.837	0.389	0.741	8.83E-41	PA-5
Pnpla7	4.74E-45	0.680	0.334	0.087	9.46E-41	PA-5
5031439G07Ri	5.81E-45	0.609	0.328	0.084	1.16E-40	PA-5
Axl.3	1.11E-44	-1.183	0.505	0.778	2.22E-40	PA-5
Gpc6.3	1.46E-44	-1.008	0.874	0.984	2.92E-40	PA-5
Igf1.3	1.68E-44	0.738	0.573	0.215	3.36E-40	PA-5
Tbc1d1	1.34E-43	0.727	0.297	0.072	2.67E-39	PA-5
Ppp3ca.1	1.67E-43	0.924	0.952	0.79	3.33E-39	PA-5
Nrp1.3	2.61E-43	0.902	0.823	0.464	5.20E-39	PA-5
Itpkb	3.35E-43	0.944	0.505	0.188	6.68E-39	PA-5
Nav3.3	1.56E-42	-1.624	0.676	0.843	3.11E-38	PA-5
Fnbp1l	1.87E-42	0.655	0.259	0.058	3.73E-38	PA-5
Fn1.3	4.53E-42	-1.240	0.693	0.894	9.05E-38	PA-5
Anxa3.3	5.00E-42	-1.337	0.642	0.823	9.97E-38	PA-5
Etv1	7.52E-42	0.665	0.266	0.06	1.50E-37	PA-5
Cmklr1	8.62E-42	0.606	0.276	0.065	1.72E-37	PA-5
Clec3b.2	1.15E-41	-1.321	0.601	0.85	2.30E-37	PA-5
Tgfbi.2	1.30E-41	0.638	0.406	0.123	2.60E-37	PA-5
Kalrn.2	1.93E-41	1.304	0.375	0.116	3.85E-37	PA-5
Ebf2.3	3.02E-41	-0.964	0.867	0.979	6.02E-37	PA-5
Tbc1d14	4.69E-41	0.729	0.369	0.111	9.35E-37	PA-5
Nova1.3	8.89E-41	-1.080	0.785	0.933	1.77E-36	PA-5
Eps8.3	6.86E-40	0.828	0.758	0.386	1.37E-35	PA-5
Slc25a37	8.42E-40	0.597	0.317	0.085	1.68E-35	PA-5
Lpl.2	1.63E-39	1.005	0.42	0.142	3.26E-35	PA-5
Stk10	1.79E-39	0.644	0.266	0.064	3.57E-35	PA-5
Efemp1.3	2.63E-39	-1.353	0.478	0.756	5.24E-35	PA-5
Mecom	3.77E-39	2.442	0.334	0.1	7.52E-35	PA-5
Tmcc1	5.66E-39	0.889	0.928	0.771	1.13E-34	PA-5
Ppm1h	1.68E-38	0.890	0.679	0.345	3.36E-34	PA-5
Dcn.2	1.81E-38	-1.067	0.823	0.965	3.62E-34	PA-5
Tsc22d1	8.31E-38	0.982	0.666	0.339	1.66E-33	PA-5
Palld.2	2.12E-37	0.768	0.433	0.147	4.23E-33	PA-5
Fchsd2	2.29E-37	1.013	0.689	0.383	4.57E-33	PA-5
Apobec3	2.89E-37	0.607	0.311	0.087	5.77E-33	PA-5
Rhobtb1.2	3.38E-37	0.746	0.447	0.16	6.75E-33	PA-5
Zeb2	4.85E-37	0.864	0.942	0.951	9.68E-33	PA-5
Gna12	1.18E-36	0.806	0.502	0.206	2.36E-32	PA-5
Prcp	1.22E-36	0.668	0.321	0.096	2.43E-32	PA-5
Syk	1.71E-36	0.875	0.584	0.27	3.40E-32	PA-5
Ank	2.24E-36	0.685	0.41	0.141	4.48E-32	PA-5
Slc43a3.2	3.31E-36	-0.980	0.679	0.87	6.61E-32	PA-5

Dpt.4	5.76E-36	-1.351	0.416	0.705	1.15E-31	PA-5
Fnbp1.2	7.63E-36	0.982	0.686	0.386	1.52E-31	PA-5
Tcf4.2	1.67E-35	0.782	0.976	0.882	3.33E-31	PA-5
Serping1.2	1.77E-35	-1.157	0.543	0.794	3.52E-31	PA-5
Col14a1.3	1.92E-35	-1.189	0.608	0.814	3.83E-31	PA-5
Tanc2.1	4.57E-35	1.117	0.812	0.575	9.12E-31	PA-5
Adamts5.3	1.35E-34	-1.127	0.679	0.854	2.69E-30	PA-5
Slco3a1.3	1.64E-34	-1.020	0.642	0.827	3.28E-30	PA-5
Cerk	1.76E-34	0.739	0.451	0.178	3.51E-30	PA-5
Cd55.3	2.72E-34	-1.332	0.406	0.693	5.43E-30	PA-5
Fggy	3.63E-34	0.557	0.273	0.072	7.24E-30	PA-5
Ppp1r13b	4.87E-34	0.648	0.259	0.068	9.72E-30	PA-5
Tgfbr3.1	5.98E-34	-0.769	0.867	0.979	1.19E-29	PA-5
Pid1.3	7.49E-34	0.952	0.898	0.753	1.49E-29	PA-5
Ackr3.3	1.52E-33	-1.278	0.358	0.654	3.03E-29	PA-5
Antxr1.2	1.91E-33	-0.849	0.703	0.881	3.81E-29	PA-5
Slc16a10	2.28E-33	0.688	0.403	0.142	4.56E-29	PA-5
Tep1.1	2.53E-33	0.730	0.427	0.163	5.06E-29	PA-5
Picalm	2.54E-33	0.817	0.86	0.615	5.08E-29	PA-5
Chst11.1	2.64E-33	0.742	0.413	0.152	5.26E-29	PA-5
Skap2	4.05E-33	0.830	0.802	0.511	8.08E-29	PA-5
Smap2	4.55E-33	0.614	0.338	0.11	9.09E-29	PA-5
Cd34.1	8.80E-33	-0.966	0.502	0.735	1.76E-28	PA-5
Scara5.3	9.26E-33	-0.932	0.635	0.85	1.85E-28	PA-5
Adam12.3	1.05E-32	0.780	0.512	0.207	2.09E-28	PA-5
Osbpl8	1.34E-32	0.757	0.778	0.453	2.68E-28	PA-5
Plxdc2.2	1.62E-32	-0.817	0.887	0.961	3.23E-28	PA-5
Ahcyl2	2.24E-32	0.698	0.519	0.22	4.47E-28	PA-5
Pard3b	3.36E-32	-0.651	0.949	0.984	6.71E-28	PA-5
Anxa1.3	4.68E-32	-1.060	0.338	0.653	9.35E-28	PA-5
Ltbp1.3	5.09E-32	-0.897	0.676	0.864	1.02E-27	PA-5
Dock10.3	5.16E-32	0.736	0.72	0.385	1.03E-27	PA-5
Iqgap2.1	6.54E-32	0.823	0.642	0.34	1.31E-27	PA-5
Heg1.2	1.00E-31	-0.820	0.884	0.961	2.00E-27	PA-5
Plcb1.2	1.08E-31	1.168	0.512	0.229	2.15E-27	PA-5
Dapp1	1.12E-31	0.556	0.29	0.085	2.23E-27	PA-5
Pcolce2.3	1.67E-31	-1.137	0.379	0.683	3.33E-27	PA-5
Ap1s2	1.80E-31	0.594	0.338	0.112	3.59E-27	PA-5
Selenop.2	2.41E-31	0.868	0.451	0.185	4.81E-27	PA-5
Rassf3	3.49E-31	0.681	0.365	0.127	6.96E-27	PA-5
Adamtsl1.4	3.85E-31	-0.973	0.706	0.909	7.69E-27	PA-5
Hmox2	5.71E-31	0.569	0.314	0.101	1.14E-26	PA-5
Tpk1	1.14E-30	0.673	0.413	0.157	2.27E-26	PA-5
Zfhx4	5.12E-30	-0.707	0.788	0.954	1.02E-25	PA-5
2610507B11Ri	5.19E-30	0.567	0.461	0.191	1.04E-25	PA-5
Arhgap31	5.30E-30	0.910	0.648	0.343	1.06E-25	PA-5
Vcan.3	5.34E-30	-1.090	0.427	0.705	1.07E-25	PA-5
Igfbp6.3	9.74E-30	-1.448	0.276	0.596	1.94E-25	PA-5
Sulf1.3	1.48E-29	-0.859	0.703	0.863	2.95E-25	PA-5
Ankrd44	1.95E-29	0.764	0.73	0.446	3.89E-25	PA-5
Gm12353	2.06E-29	0.812	0.608	0.311	4.12E-25	PA-5
Rasa3.2	2.08E-29	0.544	0.498	0.204	4.15E-25	PA-5
Atp6v0a1.2	2.64E-29	0.733	0.577	0.292	5.27E-25	PA-5
Gpcpd1	3.26E-29	0.726	0.515	0.244	6.51E-25	PA-5
Tet3	3.45E-29	0.751	0.638	0.352	6.89E-25	PA-5
Alpk1	5.22E-29	0.595	0.386	0.141	1.04E-24	PA-5
Gda.1	6.33E-29	0.813	0.734	0.45	1.26E-24	PA-5
Lrrfip1.1	1.05E-28	0.652	0.457	0.195	2.09E-24	PA-5
Pkn1	1.32E-28	0.543	0.369	0.137	2.64E-24	PA-5
Tbc1d22a	1.36E-28	0.687	0.529	0.246	2.71E-24	PA-5
Igfbp5.3	1.59E-28	-1.739	0.345	0.632	3.18E-24	PA-5
Arhgap42.2	1.84E-28	-0.845	0.666	0.862	3.68E-24	PA-5

Sema6a	2.11E-28	1.160	0.451	0.2	4.22E-24	PA-5
Sgpl1	2.26E-28	0.574	0.389	0.15	4.51E-24	PA-5
Sema3e.3	2.80E-28	-1.473	0.273	0.586	5.58E-24	PA-5
Sgk3	3.17E-28	0.636	0.451	0.189	6.33E-24	PA-5
Hdac7.3	4.09E-28	-0.876	0.621	0.796	8.16E-24	PA-5
Rasa1	4.41E-28	0.774	0.676	0.396	8.81E-24	PA-5
Nxn.1	2.42E-27	1.325	0.355	0.138	4.83E-23	PA-5
Emilin2.3	3.32E-27	-1.022	0.297	0.584	6.63E-23	PA-5
Tspan5.2	4.35E-27	-0.735	0.819	0.935	8.67E-23	PA-5
C3.3	4.96E-27	-1.531	0.283	0.572	9.91E-23	PA-5
Limch1.3	7.70E-27	-1.175	0.403	0.653	1.54E-22	PA-5
Ston2.3	8.98E-27	-1.137	0.331	0.611	1.79E-22	PA-5
Piezo1.1	9.56E-27	0.513	0.27	0.086	1.91E-22	PA-5
Galnt16.3	1.44E-26	-1.035	0.416	0.663	2.88E-22	PA-5
Ets1	1.48E-26	0.786	0.29	0.099	2.95E-22	PA-5
Ptbp3	1.80E-26	0.679	0.751	0.449	3.60E-22	PA-5
Pla1a.4	1.85E-26	-1.082	0.375	0.614	3.69E-22	PA-5
Rnf130	1.85E-26	0.595	0.553	0.273	3.70E-22	PA-5
Mdfic	2.52E-26	0.760	0.679	0.409	5.04E-22	PA-5
Snx2	2.59E-26	0.686	0.587	0.308	5.17E-22	PA-5
Tshz2	3.78E-26	-0.635	0.952	0.979	7.54E-22	PA-5
Errfi1.3	4.19E-26	-1.154	0.461	0.694	8.36E-22	PA-5
Abr	6.53E-26	0.516	0.307	0.106	1.30E-21	PA-5
Prkca.3	7.01E-26	-0.721	0.792	0.918	1.40E-21	PA-5
Col4a2.3	1.02E-25	0.557	0.693	0.37	2.03E-21	PA-5
Cdk17	1.05E-25	0.805	0.502	0.242	2.10E-21	PA-5
Man1c1.1	1.12E-25	0.672	0.621	0.352	2.24E-21	PA-5
Timp2.3	1.20E-25	-1.145	0.444	0.69	2.39E-21	PA-5
Mgat5	1.56E-25	0.597	0.502	0.231	3.11E-21	PA-5
Antxr2	3.01E-25	-0.831	0.775	0.892	6.01E-21	PA-5
Cltc	3.17E-25	0.701	0.614	0.35	6.33E-21	PA-5
Fbxw7	3.23E-25	0.670	0.611	0.333	6.44E-21	PA-5
Ap2a2	3.85E-25	0.746	0.751	0.532	7.68E-21	PA-5
Acer3	4.50E-25	0.784	0.594	0.313	8.99E-21	PA-5
Etv6	4.68E-25	0.671	0.833	0.626	9.33E-21	PA-5
Aftph	7.23E-25	0.828	0.638	0.383	1.44E-20	PA-5
Tpcn1	1.60E-24	0.534	0.324	0.122	3.20E-20	PA-5
Sept11.3	2.71E-24	0.710	0.621	0.343	5.41E-20	PA-5
Dgkh	3.21E-24	0.673	0.454	0.204	6.40E-20	PA-5
Col1a2.2	3.67E-24	-0.694	0.826	0.949	7.32E-20	PA-5
Snx30	5.30E-24	0.654	0.379	0.159	1.06E-19	PA-5
Tmeff2	6.12E-24	-0.635	0.826	0.974	1.22E-19	PA-5
Lamc1.1	8.53E-24	-0.638	0.833	0.924	1.70E-19	PA-5
Magi1.1	9.12E-24	1.340	0.659	0.403	1.82E-19	PA-5
Sema6d	9.89E-24	0.530	0.28	0.097	1.97E-19	PA-5
Plpp1.3	1.42E-23	0.698	0.553	0.289	2.83E-19	PA-5
Cdon	1.42E-23	-0.851	0.532	0.738	2.84E-19	PA-5
Tmem164	3.20E-23	0.678	0.751	0.511	6.39E-19	PA-5
Col4a1.3	3.26E-23	0.604	0.758	0.453	6.51E-19	PA-5
Plce1.2	4.06E-23	-0.672	0.778	0.908	8.11E-19	PA-5
Tsc22d3	4.47E-23	0.506	0.461	0.212	8.92E-19	PA-5
Atxn1.2	5.55E-23	0.691	0.792	0.582	1.11E-18	PA-5
Hpcal1	7.00E-23	0.675	0.546	0.298	1.40E-18	PA-5
Zbtb46	8.93E-23	0.568	0.283	0.1	1.78E-18	PA-5
Etl4	1.01E-22	1.867	0.287	0.111	2.02E-18	PA-5
Foxn3.1	1.08E-22	0.618	0.918	0.771	2.16E-18	PA-5
Ophn1	1.09E-22	0.685	0.799	0.644	2.18E-18	PA-5
Adgrg2.4	1.59E-22	-1.022	0.188	0.47	3.18E-18	PA-5
Mef2c.1	1.65E-22	0.751	0.758	0.518	3.29E-18	PA-5
Rnpep	2.33E-22	0.523	0.345	0.141	4.66E-18	PA-5
Efhd1.3	2.58E-22	-1.136	0.201	0.463	5.14E-18	PA-5
Itpr1.2	2.73E-22	0.914	0.553	0.31	5.46E-18	PA-5

Zfp710	2.89E-22	0.571	0.512	0.258	5.77E-18	PA-5
Gm37033	5.51E-22	0.510	0.294	0.109	1.10E-17	PA-5
Loxl1	6.76E-22	-0.855	0.382	0.599	1.35E-17	PA-5
Medag	8.51E-22	-0.786	0.57	0.748	1.70E-17	PA-5
Ecm1.2	1.04E-21	-0.993	0.341	0.595	2.07E-17	PA-5
Slc4a4.3	1.24E-21	-0.733	0.686	0.864	2.47E-17	PA-5
Zfp521	1.51E-21	-0.557	0.857	0.938	3.02E-17	PA-5
Ildr2.3	1.85E-21	-1.103	0.239	0.492	3.69E-17	PA-5
Gnaq	2.17E-21	0.575	0.935	0.852	4.33E-17	PA-5
Sh3d19.1	2.75E-21	-0.637	0.73	0.868	5.48E-17	PA-5
Pde8a.3	4.13E-21	-0.829	0.584	0.737	8.25E-17	PA-5
Fyn.3	4.22E-21	-0.576	0.758	0.874	8.42E-17	PA-5
Hip1	5.16E-21	0.724	0.676	0.433	1.03E-16	PA-5
Rab8b	5.87E-21	0.682	0.706	0.478	1.17E-16	PA-5
Arhgap20	6.41E-21	-0.863	0.338	0.579	1.28E-16	PA-5
Cp.3	7.43E-21	1.053	0.41	0.195	1.48E-16	PA-5
Uap1.3	8.55E-21	-0.994	0.474	0.657	1.71E-16	PA-5
Ugdh.4	1.07E-20	-0.985	0.334	0.579	2.14E-16	PA-5
Raph1.2	1.18E-20	0.649	0.71	0.485	2.36E-16	PA-5
Tank	1.26E-20	0.571	0.519	0.27	2.52E-16	PA-5
Dlc1	1.32E-20	-0.534	0.952	0.994	2.64E-16	PA-5
Zeb1.2	1.40E-20	-0.596	0.922	0.957	2.79E-16	PA-5
Rassf2	1.49E-20	0.536	0.474	0.235	2.97E-16	PA-5
Cd248.3	1.79E-20	-1.065	0.242	0.488	3.58E-16	PA-5
mt-Co1.2	1.83E-20	0.730	0.993	0.961	3.65E-16	PA-5
Plpp3.4	2.33E-20	-0.848	0.819	0.883	4.65E-16	PA-5
mt-Nd6.2	3.97E-20	0.660	0.686	0.424	7.93E-16	PA-5
Actr3	4.44E-20	0.608	0.584	0.338	8.87E-16	PA-5
Arrb1	5.65E-20	0.562	0.355	0.155	1.13E-15	PA-5
Prss23.3	6.98E-20	-0.985	0.201	0.453	1.39E-15	PA-5
Man1a.1	7.01E-20	-0.585	0.918	0.968	1.40E-15	PA-5
Tek.4	7.21E-20	-0.850	0.471	0.67	1.44E-15	PA-5
Ano3.3	8.38E-20	-1.110	0.246	0.5	1.67E-15	PA-5
Osbpl9	9.52E-20	0.727	0.84	0.702	1.90E-15	PA-5
Lvrn.3	9.72E-20	-1.002	0.314	0.572	1.94E-15	PA-5
Sh3pxd2a.3	1.06E-19	0.523	0.41	0.19	2.12E-15	PA-5
mt-Rnr2.2	1.20E-19	0.677	0.986	0.907	2.39E-15	PA-5
Tspan14	1.26E-19	0.547	0.406	0.191	2.51E-15	PA-5
Lurap1l.3	1.58E-19	-0.958	0.3	0.548	3.15E-15	PA-5
Ehbp1	1.68E-19	-0.585	0.816	0.932	3.36E-15	PA-5
Snrk.2	1.78E-19	0.828	0.478	0.264	3.55E-15	PA-5
Pcsk6.3	2.31E-19	-0.936	0.379	0.61	4.62E-15	PA-5
Zfp462.3	3.26E-19	-0.900	0.461	0.651	6.50E-15	PA-5
Tbc1d4	3.30E-19	0.609	0.642	0.392	6.58E-15	PA-5
Cd44.1	7.20E-19	0.700	0.454	0.237	1.44E-14	PA-5
Htra3	7.28E-19	-0.729	0.539	0.724	1.45E-14	PA-5
Fgf2.3	9.76E-19	-0.817	0.297	0.538	1.95E-14	PA-5
Lama4.1	1.14E-18	-0.551	0.73	0.832	2.29E-14	PA-5
Synj1	1.51E-18	0.600	0.584	0.373	3.01E-14	PA-5
Mical2	1.76E-18	0.543	0.266	0.104	3.51E-14	PA-5
Ly6a.3	1.83E-18	-1.063	0.392	0.599	3.65E-14	PA-5
Rnase4.1	1.95E-18	-0.919	0.205	0.447	3.88E-14	PA-5
Bmp1.1	2.03E-18	-0.709	0.43	0.619	4.04E-14	PA-5
Il1rl2.3	2.07E-18	-0.818	0.491	0.691	4.13E-14	PA-5
Sgip1.3	2.48E-18	0.503	0.444	0.212	4.96E-14	PA-5
Nr1h4.4	2.49E-18	-1.042	0.174	0.413	4.97E-14	PA-5
Kif2a.3	2.62E-18	-0.781	0.474	0.659	5.22E-14	PA-5
Adamts16.4	3.40E-18	-1.222	0.082	0.322	6.79E-14	PA-5
Slc6a6	3.60E-18	0.507	0.338	0.152	7.18E-14	PA-5
Car8.3	4.41E-18	-0.973	0.147	0.388	8.80E-14	PA-5
Cdc42se2	4.46E-18	0.520	0.577	0.33	8.91E-14	PA-5
Dnmt3a	4.86E-18	0.623	0.618	0.395	9.70E-14	PA-5

Flrt2.2	5.75E-18	-0.799	0.713	0.842	1.15E-13	PA-5
Naaladl2.1	7.37E-18	-0.570	0.867	0.972	1.47E-13	PA-5
Jarid2	7.57E-18	0.643	0.713	0.501	1.51E-13	PA-5
mt-Nd5.2	9.54E-18	0.653	0.915	0.778	1.90E-13	PA-5
Klhl29.2	1.00E-17	-0.839	0.386	0.605	2.01E-13	PA-5
Cbl	1.50E-17	0.566	0.601	0.373	3.00E-13	PA-5
Slc24a3.2	2.50E-17	0.564	0.491	0.268	4.99E-13	PA-5
Aldh1a3.3	2.87E-17	-1.027	0.147	0.368	5.74E-13	PA-5
Mapk14	2.88E-17	0.605	0.659	0.468	5.76E-13	PA-5
Kitl.2	4.20E-17	0.653	0.536	0.314	8.38E-13	PA-5
Klf4.3	4.24E-17	-0.817	0.29	0.518	8.46E-13	PA-5
Gfod1	4.58E-17	0.764	0.266	0.109	9.15E-13	PA-5
Inpp4a	4.87E-17	0.524	0.505	0.279	9.72E-13	PA-5
Nox4.1	4.91E-17	-0.783	0.71	0.865	9.80E-13	PA-5
Plekhm3	4.94E-17	0.548	0.635	0.427	9.85E-13	PA-5
Clip1.3	4.98E-17	-0.653	0.584	0.734	9.94E-13	PA-5
Pitpna	5.10E-17	0.598	0.655	0.452	1.02E-12	PA-5
Mast4.2	5.19E-17	-0.672	0.942	0.942	1.04E-12	PA-5
Rnf169	5.25E-17	0.635	0.686	0.479	1.05E-12	PA-5
Aebp1.2	5.32E-17	-1.018	0.352	0.553	1.06E-12	PA-5
Pde7b.1	5.33E-17	0.672	0.556	0.33	1.06E-12	PA-5
Marcks.3	6.27E-17	-0.872	0.423	0.607	1.25E-12	PA-5
Sox5	6.29E-17	-0.520	0.881	0.954	1.26E-12	PA-5
Mitf.1	6.61E-17	0.531	0.85	0.764	1.32E-12	PA-5
Fndc3a	7.34E-17	0.582	0.659	0.464	1.46E-12	PA-5
mt-Nd4l.2	7.82E-17	0.645	0.908	0.749	1.56E-12	PA-5
Dcbld2.2	1.16E-16	-0.684	0.57	0.717	2.31E-12	PA-5
Reps2.3	1.34E-16	0.585	0.546	0.313	2.67E-12	PA-5
Rarres2.1	1.57E-16	-0.937	0.106	0.329	3.13E-12	PA-5
Sbsn.3	1.69E-16	-1.089	0.061	0.277	3.38E-12	PA-5
mt-Co2.3	1.81E-16	0.657	0.993	0.945	3.61E-12	PA-5
Dact2.3	2.36E-16	-1.089	0.096	0.317	4.71E-12	PA-5
BC005537	3.04E-16	0.529	0.447	0.248	6.07E-12	PA-5
Sh3bgrl	3.05E-16	0.584	0.546	0.312	6.10E-12	PA-5
Aopep	4.11E-16	0.504	0.734	0.51	8.20E-12	PA-5
Aff3.2	4.68E-16	0.751	0.614	0.424	9.34E-12	PA-5
Maf.2	4.75E-16	0.655	0.761	0.582	9.47E-12	PA-5
Prrx1	5.58E-16	-0.528	0.788	0.915	1.11E-11	PA-5
Pamr1.2	6.30E-16	-0.740	0.358	0.556	1.26E-11	PA-5
Enox2	7.73E-16	0.505	0.693	0.452	1.54E-11	PA-5
Ugp2.2	7.94E-16	-0.849	0.481	0.632	1.59E-11	PA-5
Magi2	1.05E-15	-0.673	0.795	0.879	2.10E-11	PA-5
Col5a1	1.11E-15	-0.573	0.604	0.746	2.22E-11	PA-5
Spsb1.3	1.16E-15	-0.819	0.297	0.516	2.31E-11	PA-5
Scara3.2	1.16E-15	-0.719	0.191	0.413	2.31E-11	PA-5
Adam33.2	1.21E-15	-0.728	0.444	0.636	2.42E-11	PA-5
Bicc1	1.25E-15	-0.507	0.833	0.954	2.49E-11	PA-5
Fnip2	1.38E-15	0.585	0.584	0.37	2.76E-11	PA-5
Gtdc1	1.66E-15	0.516	0.526	0.297	3.32E-11	PA-5
Smpd3.4	1.97E-15	-0.987	0.362	0.565	3.93E-11	PA-5
Phactr2	2.26E-15	0.624	0.594	0.391	4.50E-11	PA-5
Mef2a	2.97E-15	0.503	0.87	0.732	5.93E-11	PA-5
Rorb.3	3.03E-15	-1.028	0.273	0.483	6.06E-11	PA-5
Pdgfra.2	3.91E-15	-0.660	0.488	0.691	7.81E-11	PA-5
Tmem100.3	4.35E-15	-0.787	0.15	0.361	8.69E-11	PA-5
Lims2.3	5.16E-15	-0.672	0.454	0.627	1.03E-10	PA-5
Cyth1	5.64E-15	0.569	0.502	0.306	1.13E-10	PA-5
Adamts2	6.99E-15	-0.605	0.59	0.721	1.40E-10	PA-5
Dip2b	9.64E-15	0.640	0.655	0.482	1.92E-10	PA-5
Inpp4b	1.05E-14	0.643	0.297	0.134	2.10E-10	PA-5
Dpysl3	1.08E-14	-0.678	0.563	0.714	2.15E-10	PA-5
Abca9	1.10E-14	0.537	0.662	0.448	2.19E-10	PA-5

Srgap1.1	1.16E-14	-0.607	0.683	0.839	2.32E-10	PA-5
mt-Atp6.2	1.23E-14	0.587	0.997	0.953	2.46E-10	PA-5
Fgd4.1	1.29E-14	0.623	0.666	0.477	2.57E-10	PA-5
Smurf2.3	1.31E-14	-0.687	0.812	0.871	2.62E-10	PA-5
mt-Co3.3	2.02E-14	0.580	0.997	0.954	4.03E-10	PA-5
mt-Rnr1.1	2.22E-14	0.510	0.969	0.906	4.44E-10	PA-5
Ppp2r2b.3	3.41E-14	-1.065	0.235	0.437	6.80E-10	PA-5
Sparc	5.25E-14	-0.662	0.71	0.822	1.05E-09	PA-5
Camk1d	8.65E-14	-0.677	0.85	0.87	1.73E-09	PA-5
Slc7a6.1	9.02E-14	-0.697	0.253	0.442	1.80E-09	PA-5
Reck	9.45E-14	-0.681	0.324	0.508	1.89E-09	PA-5
Ogn.3	1.12E-13	-0.772	0.481	0.625	2.23E-09	PA-5
Gsn.4	1.17E-13	-0.746	0.853	0.914	2.34E-09	PA-5
mt-Nd4.2	1.21E-13	0.607	0.976	0.907	2.41E-09	PA-5
Serpinf1.2	1.30E-13	-0.760	0.413	0.59	2.60E-09	PA-5
Ddx60	1.39E-13	0.581	0.437	0.255	2.77E-09	PA-5
Thbd.2	2.23E-13	-0.742	0.096	0.287	4.45E-09	PA-5
Hecw2	2.70E-13	-0.600	0.611	0.754	5.40E-09	PA-5
Rasal2	2.92E-13	0.562	0.659	0.455	5.82E-09	PA-5
Cmah.3	3.00E-13	-0.882	0.468	0.609	5.99E-09	PA-5
Nhsl1.3	3.41E-13	-0.640	0.601	0.746	6.80E-09	PA-5
Cadm3.2	4.60E-13	-0.709	0.16	0.352	9.18E-09	PA-5
Itih5.1	4.94E-13	-0.650	0.549	0.651	9.87E-09	PA-5
Gan.3	6.36E-13	-0.993	0.307	0.478	1.27E-08	PA-5
Calu	6.98E-13	-0.589	0.352	0.525	1.39E-08	PA-5
Ntn4.3	7.99E-13	-0.746	0.307	0.499	1.59E-08	PA-5
Cd47.2	8.01E-13	-0.517	0.686	0.738	1.60E-08	PA-5
Apba1	8.12E-13	0.505	0.724	0.541	1.62E-08	PA-5
Prex2.3	9.66E-13	0.665	0.655	0.448	1.93E-08	PA-5
Tut7	1.17E-12	0.516	0.618	0.44	2.33E-08	PA-5
Aspn.1	1.51E-12	-0.751	0.423	0.584	3.02E-08	PA-5
Vgll3.2	1.63E-12	-0.736	0.522	0.663	3.26E-08	PA-5
Rps15.2	1.87E-12	-0.875	0.099	0.29	3.73E-08	PA-5
Fxyd1	2.16E-12	-0.563	0.082	0.258	4.30E-08	PA-5
Ly6c1.3	2.16E-12	-0.519	0.239	0.433	4.31E-08	PA-5
Rgs6.3	2.20E-12	-1.034	0.215	0.406	4.40E-08	PA-5
Kank1.3	2.53E-12	-0.813	0.253	0.432	5.06E-08	PA-5
Igfbp4.3	2.66E-12	-0.766	0.27	0.45	5.30E-08	PA-5
Procr.4	2.73E-12	-0.725	0.164	0.352	5.45E-08	PA-5
Atp5o.1	2.79E-12	0.522	0.553	0.37	5.57E-08	PA-5
Gstm1.1	3.70E-12	-0.607	0.085	0.263	7.38E-08	PA-5
Tbc1d12.3	3.96E-12	-0.570	0.43	0.592	7.91E-08	PA-5
Tmcc3	4.24E-12	0.546	0.666	0.478	8.45E-08	PA-5
Islr.1	5.77E-12	-0.646	0.157	0.342	1.15E-07	PA-5
Arl4a.2	6.39E-12	-0.658	0.249	0.422	1.28E-07	PA-5
Psap	6.40E-12	0.655	0.334	0.179	1.28E-07	PA-5
Klf2.3	8.31E-12	-0.669	0.14	0.323	1.66E-07	PA-5
Itga11.3	8.91E-12	-0.647	0.328	0.506	1.78E-07	PA-5
Xiap	9.22E-12	0.501	0.686	0.518	1.84E-07	PA-5
Gm33869.2	9.98E-12	-0.603	0.266	0.45	1.99E-07	PA-5
Klf12.3	1.12E-11	-0.612	0.522	0.662	2.23E-07	PA-5
Pdzd2.3	1.20E-11	-0.669	0.43	0.583	2.41E-07	PA-5
Slc1a5	1.25E-11	-0.585	0.457	0.598	2.49E-07	PA-5
Dock9.3	1.31E-11	0.708	0.642	0.467	2.61E-07	PA-5
Radil	1.65E-11	-0.581	0.256	0.44	3.30E-07	PA-5
Dbn1.3	2.28E-11	-0.641	0.242	0.407	4.56E-07	PA-5
Epb41l3.2	2.30E-11	-0.686	0.242	0.415	4.60E-07	PA-5
Wnt2.3	2.38E-11	-0.647	0.085	0.255	4.74E-07	PA-5
Arhgef28.3	2.48E-11	-0.569	0.488	0.636	4.94E-07	PA-5
Sestd1.3	4.12E-11	-0.585	0.522	0.661	8.22E-07	PA-5
Vegfc.2	4.89E-11	0.613	0.372	0.206	9.77E-07	PA-5
Csf1	5.03E-11	-0.551	0.27	0.442	1.00E-06	PA-5

Dact1.2	6.00E-11	-0.706	0.212	0.368	1.20E-06	PA-5
Robo1.3	6.59E-11	-0.840	0.427	0.571	1.32E-06	PA-5
Rhoa.1	6.82E-11	-0.503	0.481	0.639	1.36E-06	PA-5
Ptprj.3	9.02E-11	0.706	0.867	0.735	1.80E-06	PA-5
S100a10	1.20E-10	-0.771	0.123	0.294	2.39E-06	PA-5
Sytl2.3	1.20E-10	-0.637	0.119	0.285	2.39E-06	PA-5
Rps21.3	1.29E-10	-0.879	0.177	0.351	2.58E-06	PA-5
Abca6.2	1.45E-10	-0.585	0.423	0.576	2.90E-06	PA-5
Ccdc80.2	1.47E-10	-0.699	0.549	0.674	2.94E-06	PA-5
Ahnak.1	1.59E-10	-0.587	0.799	0.843	3.18E-06	PA-5
Cep112.1	2.01E-10	-0.649	0.577	0.655	4.01E-06	PA-5
S100a6	2.02E-10	-1.289	0.242	0.396	4.04E-06	PA-5
Col1a1.3	2.07E-10	-0.558	0.7	0.78	4.12E-06	PA-5
Fbln2.1	2.09E-10	-0.659	0.556	0.675	4.18E-06	PA-5
Il18.3	3.05E-10	-0.623	0.157	0.316	6.09E-06	PA-5
Plat.1	3.15E-10	-0.630	0.253	0.411	6.29E-06	PA-5
Cmss1.1	3.22E-10	-0.784	0.72	0.791	6.42E-06	PA-5
mt-Nd2.1	3.85E-10	0.529	0.962	0.902	7.69E-06	PA-5
Lsp1.1	3.96E-10	-0.525	0.396	0.551	7.90E-06	PA-5
Negr1.3	5.37E-10	-0.947	0.457	0.563	1.07E-05	PA-5
Ar	6.22E-10	-0.534	0.604	0.711	1.24E-05	PA-5
Dusp1.1	6.63E-10	-0.589	0.133	0.29	1.32E-05	PA-5
Irak3.1	6.71E-10	-0.525	0.43	0.564	1.34E-05	PA-5
Lbp.1	6.99E-10	-0.540	0.174	0.338	1.40E-05	PA-5
Thbs3	7.92E-10	-0.535	0.235	0.4	1.58E-05	PA-5
C1ra.1	7.95E-10	-0.529	0.212	0.381	1.59E-05	PA-5
Serpinh1.1	8.33E-10	-0.608	0.294	0.452	1.66E-05	PA-5
Abca5.3	8.53E-10	-0.617	0.201	0.359	1.70E-05	PA-5
S100a11.1	8.82E-10	-0.810	0.102	0.251	1.76E-05	PA-5
Grb10	9.94E-10	-0.553	0.638	0.725	1.98E-05	PA-5
Ccnd3.1	1.03E-09	0.519	0.666	0.537	2.06E-05	PA-5
Cdh9.3	1.28E-09	-0.691	0.215	0.375	2.56E-05	PA-5
Ptgis.2	1.45E-09	-0.550	0.154	0.308	2.89E-05	PA-5
Fam20a.2	1.77E-09	-0.529	0.382	0.535	3.53E-05	PA-5
Rcan2.1	1.79E-09	-0.557	0.519	0.656	3.57E-05	PA-5
Tanc1	1.86E-09	0.593	0.526	0.367	3.72E-05	PA-5
Jpt1.2	2.02E-09	-0.529	0.174	0.334	4.04E-05	PA-5
Fryl	2.32E-09	0.551	0.659	0.487	4.62E-05	PA-5
Pcsk5.1	2.43E-09	-0.663	0.587	0.681	4.85E-05	PA-5
Lmx1a.2	3.33E-09	-0.592	0.15	0.305	6.66E-05	PA-5
Eya4.3	3.50E-09	-0.728	0.276	0.428	6.98E-05	PA-5
Gabra3.2	4.14E-09	-0.743	0.369	0.513	8.27E-05	PA-5
Rhoq.3	4.62E-09	-0.529	0.519	0.614	9.22E-05	PA-5
Pgm3	6.05E-09	-0.516	0.195	0.348	0.00012081	PA-5
Ahnak2.1	6.84E-09	-0.546	0.239	0.385	0.00013644	PA-5
Adamtsl3.2	7.56E-09	-0.766	0.212	0.365	0.000150961	PA-5
Acss3.1	8.14E-09	-0.550	0.519	0.625	0.000162562	PA-5
Iqsec1.3	8.34E-09	-0.536	0.314	0.462	0.000166464	PA-5
1700019D03Ri	8.74E-09	-0.539	0.133	0.274	0.000174375	PA-5
Basp1.3	1.02E-08	-0.603	0.232	0.379	0.000204277	PA-5
CT010467.1	1.20E-08	-0.579	0.785	0.821	0.000240144	PA-5
Pde10a.1	1.55E-08	-0.615	0.3	0.438	0.000308499	PA-5
Stox2	1.58E-08	0.955	0.433	0.293	0.000314703	PA-5
Hexb.1	1.74E-08	-0.584	0.314	0.451	0.000347694	PA-5
Bicd1.2	2.24E-08	-0.602	0.27	0.406	0.000447113	PA-5
Gas7.3	2.66E-08	-0.506	0.826	0.864	0.000531644	PA-5
Clip4.2	2.86E-08	-0.503	0.167	0.311	0.000571846	PA-5
Sgce	3.53E-08	-0.521	0.403	0.526	0.000705224	PA-5
Nipal2.2	3.58E-08	-0.666	0.225	0.359	0.000713835	PA-5
Fhl1.3	4.71E-08	-0.510	0.253	0.4	0.000940246	PA-5
Crim1	4.86E-08	0.564	0.509	0.361	0.000969138	PA-5
Abca8b.1	5.45E-08	-0.583	0.341	0.479	0.001087102	PA-5

Nr3c2.1	5.74E-08	0.562	0.563	0.408	0.001144857	PA-5
Smurf1.3	7.74E-08	-0.542	0.509	0.587	0.001544249	PA-5
Megf9.2	1.04E-07	-0.522	0.382	0.503	0.002073699	PA-5
Cdh13.2	1.54E-07	0.788	0.58	0.446	0.003078013	PA-5
Sdc2	1.61E-07	-0.584	0.529	0.631	0.003218678	PA-5
Hspa5	3.03E-07	-0.576	0.212	0.342	0.006045377	PA-5
Jun	3.30E-07	-0.523	0.181	0.306	0.006578525	PA-5
Hsp90b1	3.37E-07	-0.632	0.242	0.362	0.006731566	PA-5
Lars2.1	3.59E-07	-0.617	0.549	0.636	0.007160345	PA-5
C1qtnf7	5.02E-07	-0.601	0.396	0.509	0.010011753	PA-5
Slc16a9.2	5.98E-07	-0.550	0.218	0.337	0.011928184	PA-5
Ptprk.2	8.53E-07	0.526	0.372	0.243	0.01701791	PA-5
Plekhg1	1.65E-06	0.613	0.526	0.401	0.033012301	PA-5
Adcy2.2	2.26E-06	-0.544	0.3	0.419	0.045145703	PA-5
Sorbs1	3.79E-06	0.687	0.259	0.169	0.075632703	PA-5
Syne1	4.05E-06	0.508	0.543	0.423	0.080852092	PA-5
Ptprm	4.09E-06	0.838	0.829	0.758	0.08168195	PA-5
Tmem178b.1	4.10E-06	-0.602	0.276	0.382	0.081837297	PA-5
Col12a1.2	5.01E-06	-0.677	0.225	0.337	0.09999392	PA-5
Galnt18.1	6.69E-06	1.023	0.276	0.186	0.133503228	PA-5
Sntg1.4	6.77E-06	-1.033	0.365	0.463	0.135064739	PA-5
Pcdh9.2	3.24E-05	-0.709	0.464	0.537	0.646685633	PA-5
Gpc3.1	5.39E-05	-0.636	0.27	0.367	1	PA-5
Calcrl.3	9.99E-05	-0.525	0.416	0.494	1	PA-5
Vim	0.0001333	-0.593	0.201	0.292	1	PA-5

Extended Data Table 9 Oligonucleotides	
siRNA	sense
Ctrl-1	UGGUUUACAUGUCGACUAA
Ctrl-2	UGGUUUACAUGUUGUGUGA
Ctrl-3	UGGUUUACAUGUUUUCUGA
Ctrl-4	UGGUUUACAUGUUUUCCUA
si-Cgref1-1	CAG AGA AGG UGC UGA GGA ATT
si-Cgref1-2	AGG AAC AGG UGG AGA UCA ATT
si-Cgref1-3	CAA UGA AGG AGA AGC CAA ATT
si-Colec11-1	UCA ACU GAC AAC UGA GCU ATT
si-Colec11-2	CAA CUG ACA ACU GAG CUA ATT
si-Colec11-3	GGA CAA CCA GGU CAC UCA ATT
si-Cpb1-1	GGG CUU AUG AUC AGG GAA UTT
si-Cpb1-2	AGG AGA ACU UUG AUG GCA ATT
si-Cpb1-3	GGU GAA AGG UGC AGC AAA GTT
si-Edn3-1	CUG GGA AAC AAG AGG ACA ATT
si-Edn3-2	GCA GAA GAG AUG CGG GAG ATT
si-Edn3-3	CCA GAG AUG UCA CCA GUU ATT
si-Kazald1-1	UCA CAU UCC AAA UAU UGU GAT T
si-Kazald1-2	AGU AAU CGC CCA ACU CUU CAT T
si-Kazald1-3	UUA AAC AAU CCA UGU AAG GAT T
si-Lgi1-1	GAG CAA AGC AAC ACA GCU ATT
si-Lgi1-2	GGA AUA UAA GAA ACG GAA ATT
si-Lgi1-3	CAA CAC AGC UAU UCA CUA ATT
si-Lgr4-1	GCA GUC ACC UCC GAC AGUU
si-Lgr4-2	GCA CGU UGC UUG GCU CAUC
si-Lgr4-3	GCC AUU AUC UAC AUU AAAC
si-Lgr4-4	GAA ACA CGG AUC AGU CUCA
si-Lgr5-1	GCA UUU GCC UGA ACU AAGA
si-Lgr5-2	GUA GCU GGC UGA UCC GAAU
si-Lgr5-3	GUA CAU CUC UUC CAU AAAG
si-Lgr5-4	GGA AUC GGC UGC CAA AUCG
si-Lgr6-1	GGA CUU UCA UCC AGA GGA AUU
si-Lgr6-2	ACA ACA ACA UCA AGG CUA UUU
si-Lgr6-3	GAA AUU GGC CUC CGA CAU AUU
si-Ndp-1	GGG AGA UAC AGA CAG CAA ATT
si-Ndp-2	CAG CAC UGU CCU CAA GCA ATT
si-Ndp-3	GCU GCA UGA GAC ACC AUU ATT
si-Nog-1	CCG AGG GCA UGG UGU GUA ATT
si-Nog-2	CCA UCA UUU CCG AGU GUA ATT
si-Nog-3	UCU UUG ACC CUA AGG AGA ATT
si-Rspo2-1	AGA CGC AAU AAG CGA GCUA
si-Rspo2-2	GUU GGU CAU UGG AGC GAAU
si-Rspo2-3	GGG CUU GAU UCU AAC CGUA
si-Rspo2-4	GGA CAA UGG UUG CAG CCGA
si-S100a9-1	UGG CAA CCU UUA UGA AGA ATT
si-S100a9-2	GUG GAA GCA CAG UUG GCA ATT
si-S100a9-3	UGG ACA CAA ACC AGG ACA ATT
si-Serpinb6c-1	GGA CAG GAU GAA AGG AGA ATT
si-Serpinb6c-2	GGG UGA AGA CAG AUC CAA ATT
si-Serpinb6c-3	AGG CAC UGU CUU UGG GUA ATT

Primers	
Adam12-for	ATG GCA CCC ATC ATG AGC
Adam12-rev	GGG GGC TGT CTG AAT GGT
Anxa3-for	GCA GTA CCA AGC AGC GTA TG
Anxa3-rev	GCC AGA GAG ATC ACC CTT CA
Axl-for	TAT ACC CTC GGC TGG GGA TG
Axl-rev	AGG TGG GGG TTC ACT CAC T
Bgn-for	TGC CAT GTG TCC TTT CGG TT
Bgn-rev	CAG GTC TAG CAG TGT GGT GTC
C7-for	ATG TCA GCC TTG GGA GAA AC
C7-rev	CTA CTC TCA TCT CGG GCA CA
CD142-for	GGG GAG CCT CTG TAT GAG AA
CD142-rev	ATT GTT GGC TGT CCG AGG T
CD55-for	ACT GTT GAT TGG GAC GAT GAG
CD55-rev	TGG TGG CTC TGG ACA ATG TA
Cebpa-for	GGT GCG CAA GAG CCG AGA TAA A
Cebpa-rev	CCC CGC AGC GTG TCC AGT T
Cgref1-for	CCA CTA TTG CTG CTC CCT TT
Cgref1-rev	TTG TAC CTC AGG GTC CAA CC
Col4a1-for	CTC TCC AGG GAT CAC AGG TT
Col4a1-rev	AGC CCT TTC TGA CCT TGA AA
Col4a2-for	AGC TCC TGA CCC TGA GGT TA
Col4a2-rev	TCC ATG ATC CCA GTC TTT GA
Colec11-for	GCT CTG TGC AGA TTC TTG TCC
Colec11-rev	GTC CTG GGG CTC CTT TGT
Cpb1-for	TGG TTT CCA CGC AAG AGA GT
Cpb1-rev	TCT CTT GTT TAT AGG TAC GGA CAG C
Col18a1-For	GCT CAC CAG TTT GGT CTT GC
Col18a1-Rev	CCA CCT CCT CAG CAA CAT TC
Dpp4-for	CGG TAT CAT TTA GTA AAG AGG CAA A
Dpp4-rev	GTA GAG TGT AGA GGG GCA GAC C
Edn3-for	GAG ATG CGG GAG ACT GGA
Edn3-rev	GGG AGA GCA GGT GAA GTC C
Efhd1-for	TCT GGA GAA GAT GTT CAA AAC G
Efhd1-rev	TTT CAG CTC CAT CAG GTC AA
Fmo2-for	TTC AGT GAC TTC CCG ATG C
Fmo2-rev	CAC ACT AAT GAC AGT GGT CTG AAA
Fth1-for	TGG AGT TGT ATG CCT CCT ACG
Fth1-rev	TGG AGA AAG TAT TTG GCA AAG TT
Gna14a-for	TCC GAG ACA GAG GGT GAT CT
Gna14a-rev	CCT GGT CCA GAA GCA CTT G
Icam1-for	TGG TAG ACA GCA TTT ACC CTC A
Icam1-rev	GGC CAC CAT CCT GTT CTG
Igfbp3-For	AAG CAC AGA CAC CCA GAA CT
Igfbp3-Rev	GCG GCA CTG CTT CTT CTT AT
Kazald1-for	GCT AAC CTC ACT GTG GTG CAT
Kazald1-rev	AAT ATT GTG AGG CTG CGA CA
Lgi1-for	ATG GGA ACA ACC AGA GCA AC
Lgi1-rev	TTT GCC TCT TCC CTT TAA GTC A
Lgr4-for	CAG TTA CCA GAA GAT GCA TTT AAG AA
Lgr4-rev	CAA GGC TTT TGG GTG GAT AA
Lgr5-for	GTG TCT TTT TGC CTT TTG ACG

Lgr5-rev	TGT CAG TCC AAG AAA CTG GTG T
Lgr6-for	GAG CTC GGG CTC TCA GTG
Lgr6-rev	GAG GTT GTT CAT ACT GAG GTC TAG G
Lpl-for	GCA CTC ACC TTT AAT CCC AGC ACT
Lpl-rev	TCC CTC CCT CTT CTT CTT CTC CTT
Meox2-for	AAT CTA GAC CTC ACT GAA AGA CAG G
Meox2-rev	CTT GCT GTC CCC CTT TGA
Mgp-for	GGC AAC CCT GTG CTA CGA AT
Mgp-rev	CCT GGA CTC TCT TTT GGG CTT TA
Ndp-for	GCT TTC TGA CTG GCA CAA CC
Ndp-rev	TTT CTT GCC AGT CCG TCT CT
Nog-for	AGA CAA ACC GGT GCC AAC
Nog-rev	TGG GCC ACT TCT CTC TGC
Pi16-for	GTG CGG CCA CTA CAC TCA G
Pcsk6-For	GAA CCT CCT GCA TCA CCA AC
Pcsk6-Rev	GGA TGA AGA GCT TCC GTT CG
Pi16-rev	CAC CAG CAA ATG GAT GTT AGC
Pparg-for	GCA TGG TGC CTT CGC TGA
Pparg-rev	TGG CAT CTC TGT GTC AAC CAT G
Rspo1-for	CGA CAT GAA CAA ATG CAT CA
Rspo1-rev	CTC CTG ACA CTT GGT GCA GA
Rspo2-for	GCC CAT CAG GGT ATT ATG GA
Rspo2-rev	TCA CAG TTT TCT ATT CTG CAT CG
Rspo3-for	GCG AAG AAT GCA TCC TAA TGT
Rspo3-rev	CAG CCA TTG TAA TCT GAA CAC G
Rspo4-for	TCT GAG TCC AGG AAG TGT CCT AT
Rspo4-rev	TGT TCT TCT GCC TGG GAT TT
Rtp3-for	CGC AAT ACC AGC AAA AGA CC
Rtp3-rev	CTA TCA GAA CTC GGC CAG ATG
S100a9-for	GAC ACC CTG ACA CCC TGA G
S100a9-rev	TGA GGG CTT CAT TTC TCT TCT C
Sdc1-For	AAG TGC TGG GAG GTG TCA TT
Sdc1-Rev	TGC TTG GTG GGT TTC TGG TA
Sema3c-for	ATA TCC TCG CCC AGG AAC TT
Sema3c-rev	TGG GAA GTC CTT GGT GGT T
Serpinb6c-for	CTG CTC ATC TTG CTA TCT CCA G
Serpinb6c-rev	AAA GGT TTA AGG CAA AAG TAG CAT T
Smpd3-for	TCT ACC TCC TCG ACC AGC AC
Smpd3-rev	TGC TGC TCC AGT TTG TCA TC
Sparcl1-for	GGC AAT CCC GAC AAG TAC AAG
Sparcl1-rev	TGG TTT TCT ATG TCT GCT GTA GC
Spink2-for	TGT TCG CTG CAC AGG AGA
Spink2-for	ATG GGA ACA ACC AGA GCA AC
Spink2-rev	TGA GAA TCG GAA GAG TCG AGA
Spink2-rev	TTT GCC TCT TCC CTT TAA GTC A
Tspan11-for	ATC ACA GCG TCG GTG GAC
Tspan11-rev	GCT GAG CTG TTA CTG CCA CA
Vap1-for	ACC CAC AGC GCT CAC TTC
Vap1-rev	GGG GAC AAA AGC CAT ATC CT
Vit-for	AAA CGC CAA AGA AGA CTA AAA GG
Vit-rev	AGC TTT GAC ATC GCA GTT GA
Recmbined NucRed-	TTGATTTGATACCGCGGGC

Recmbined NucRed-	GCGCATGAACTCTTTGATGAC
ApoB For	GTCCAGGTTGAATCACGGGT
ApoB Rev	AGGATCCTGCAAGGTCAAGC
