# Supplementary figures and images for: Identification of a regulatory pathway inhibiting adipogenesis via RSPO2
Source: Nat Metab. 2022 Jan 13;4(1):90–105. doi: 10.1038/s42255-021-00509-1 (PMC8803606; doi:10.1038/s42255-021-00509-1)

Fig. 4h

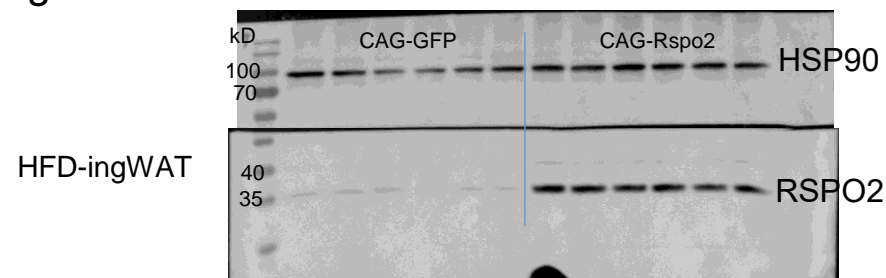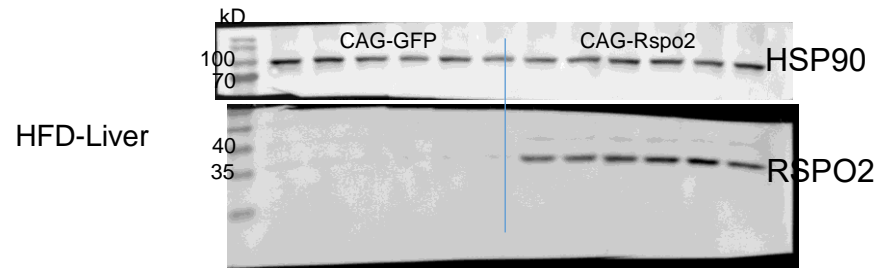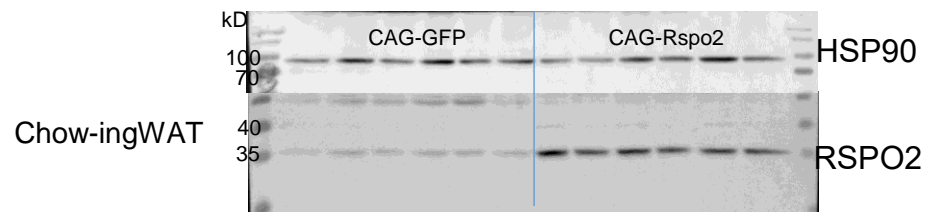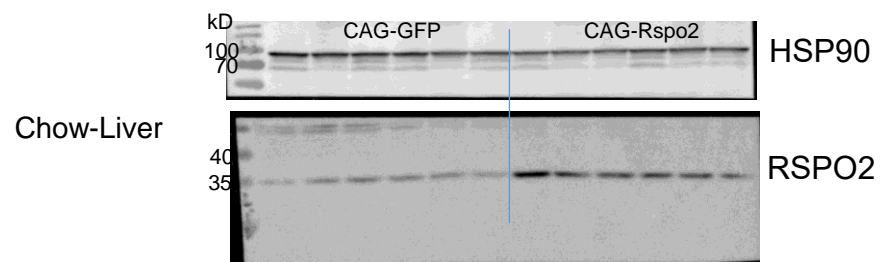

Fig. 4m

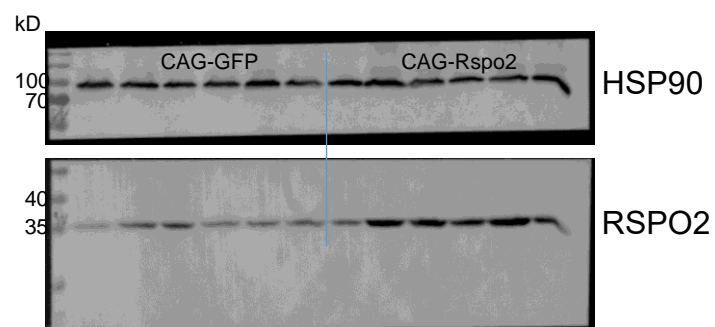

Supplement: Source Data Fig. 4 — Uncropped western blots. [file 42255_2021_509_MOESM6_ESM.pdf]

Fig. 5h

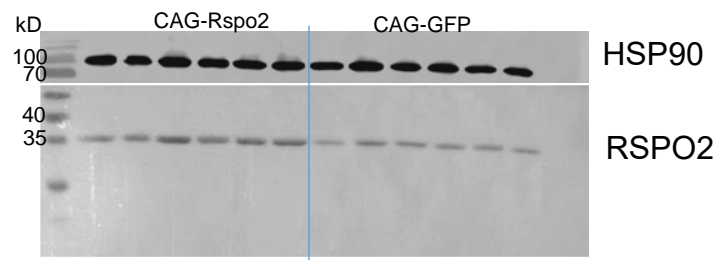

Supplement: Source Data Fig. 5 — Uncropped western blots. [file 42255_2021_509_MOESM8_ESM.pdf]

Fig. 6a

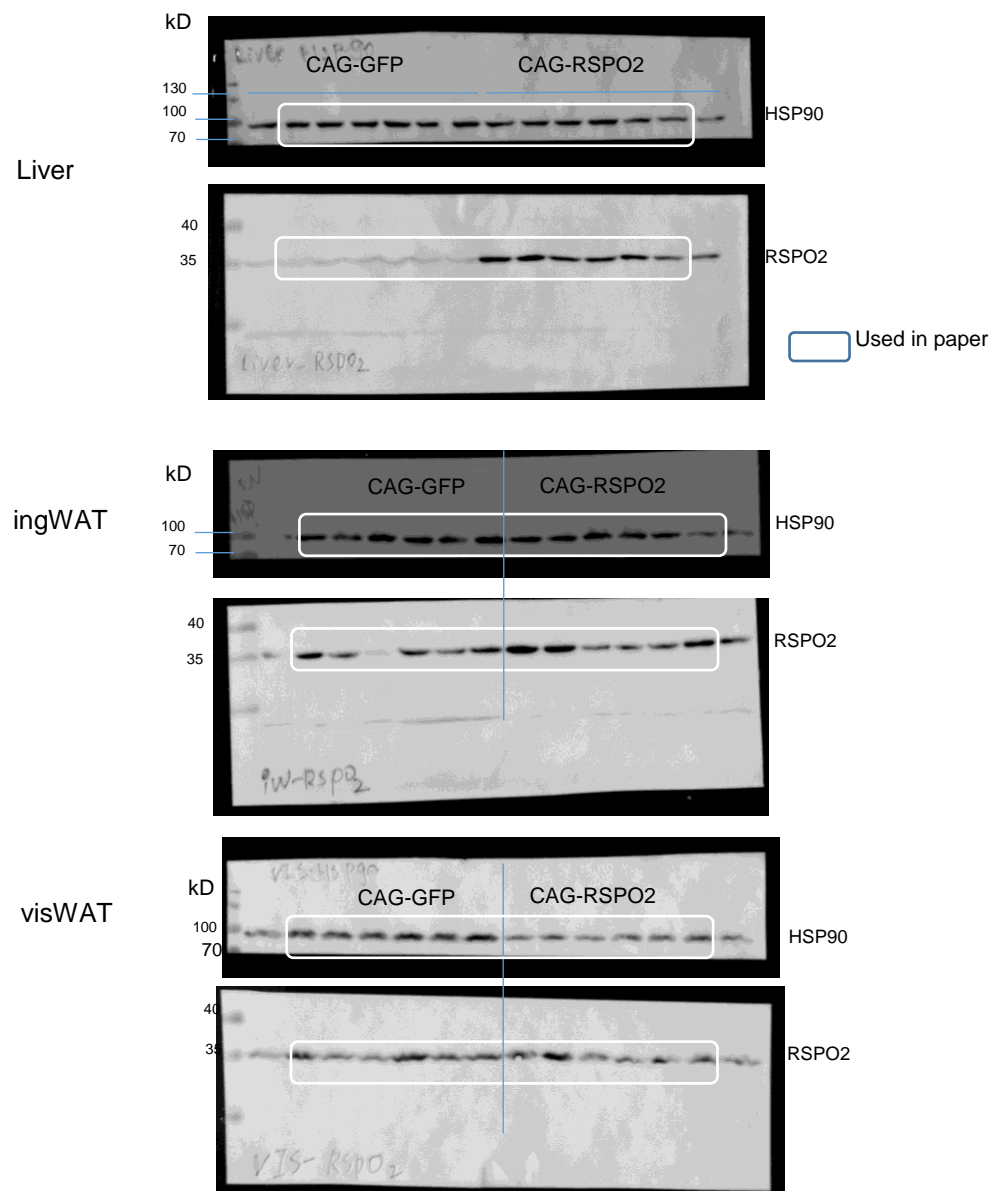

Supplement: Source Data Fig. 6 — Uncropped western blots. [file 42255_2021_509_MOESM10_ESM.pdf]

Extended Data Fig.3t

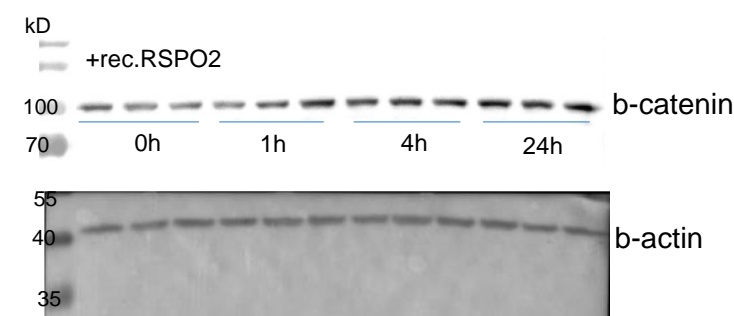

Extended Data Fig.3x

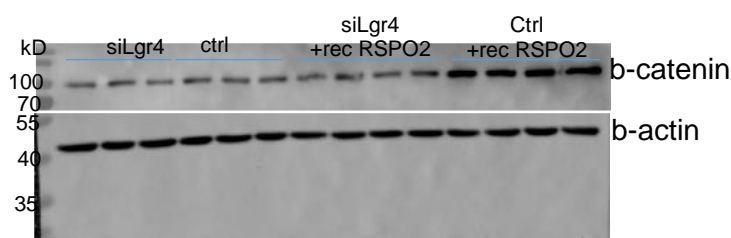

Supplement: Source Data Extended Data Fig. 3 — Uncropped western blots. [file 42255_2021_509_MOESM14_ESM.pdf]

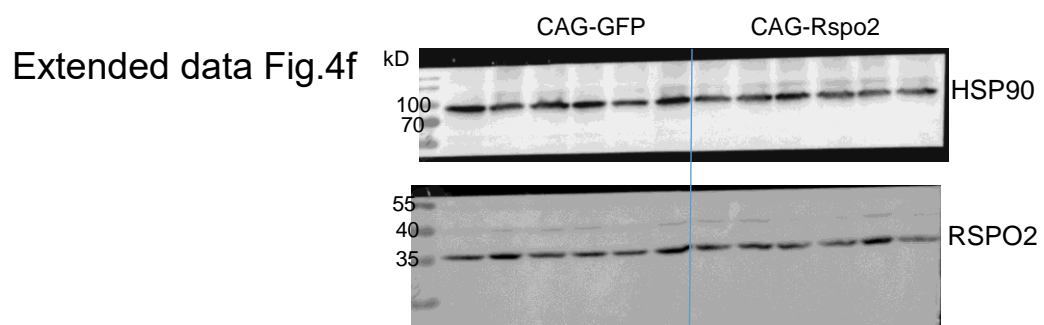

Supplement: Source Data Extended Data Fig. 4 — Uncropped western blots. [file 42255_2021_509_MOESM16_ESM.pdf]

Extended Data Fig.5I

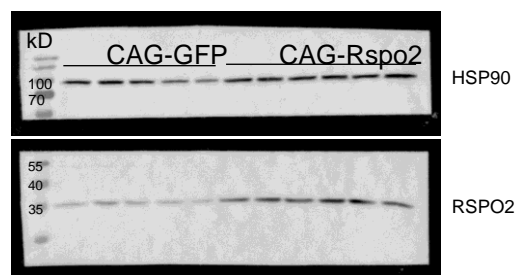

Supplement: Source Data Extended Data Fig. 5 — Uncropped western blots. [file 42255_2021_509_MOESM18_ESM.pdf]

Extended Data Fig.7d

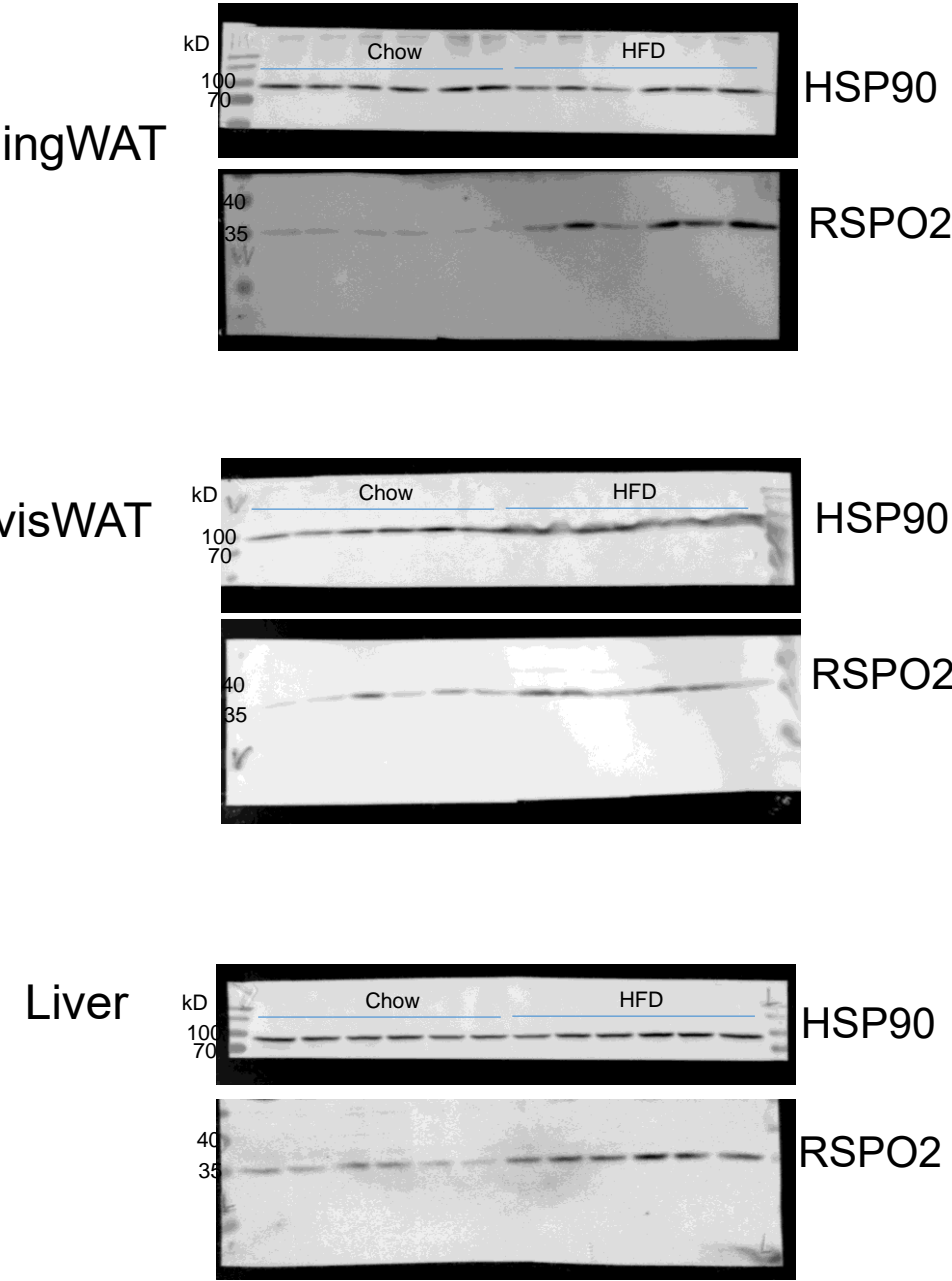

Supplement: Source Data Extended Data Fig. 7 — Uncropped western blots. [file 42255_2021_509_MOESM20_ESM.pdf]
